# Understanding palladium–tellurium cluster formation on WTe_2_: From a kinetically hindered distribution to thermodynamically controlled monodispersity

**DOI:** 10.1093/pnasnexus/pgad212

**Published:** 2023-06-28

**Authors:** Prescott E Evans, Yang Wang, Peter V Sushko, Zdenek Dohnálek

**Affiliations:** Physical and Computational Sciences Directorate, Pacific Northwest National Laboratory, Richland, WA 99352, USA; Physical and Computational Sciences Directorate, Pacific Northwest National Laboratory, Richland, WA 99352, USA; Physical and Computational Sciences Directorate, Pacific Northwest National Laboratory, Richland, WA 99352, USA; Physical and Computational Sciences Directorate, Pacific Northwest National Laboratory, Richland, WA 99352, USA; Voiland School of Chemical Engineering and Bioengineering, Washington State University, Pullman, WA 99163, USA

**Keywords:** transition metal dichalcogenides, nanoclusters, growth, scanning tunneling microscopy, ab initio modeling

## Abstract

A fundamental understanding of the transition metal dichalcogenide (TMDC)–metal interface is critical for their utilization in a broad range of applications. We investigate how the deposition of palladium (Pd), as a model metal, on WTe_2_(001), leads to the assembly of Pd into clusters and nanoparticles. Using X-ray photoemission spectroscopy, scanning tunneling microscopy imaging, and ab initio simulations, we find that Pd nucleation is driven by the interaction with and the availability of mobile excess tellurium (Te) leading to the formation of Pd-Te clusters at room temperature. Surprisingly, the nucleation of Pd-Te clusters is not affected by intrinsic surface defects, even at elevated temperatures. Upon annealing, the Pd-Te nanoclusters adopt an identical nanostructure and are stable up to ∼523 K. Density functional theory calculations provide a foundation for our understanding of the mobility of Pd and Te atoms, preferential nucleation of Pd-Te clusters, and the origin of their annealing-induced monodispersity. These results highlight the role the excess chalcogenide atoms may play in the metal deposition process. More broadly, the discoveries of synthetic pathways yielding thermally robust monodispersed nanostructures on TMDCs are critical to the manufacturing of novel quantum and microelectronics devices and catalytically active nano-alloy centers.

**Significance Statement**

Atomic-scale control of the functionalization of 2D materials, such as WTe_2_(001), is critical for the development and deployment of next-generation catalysts and quantum devices. We show palladium (Pd), a common catalyst and device contact metal, is captured at room temperature by mobile intercalated tellurium (Te). The assembled Pd-Te clusters do not nucleate on intrinsic surface defects, suggesting the defects are more complex than putative Te vacancies. Annealing the polydisperse nanoclusters converts them to a thermally stable monodisperse configuration. We demonstrate that the mismatch between cluster structure and the WTe_2_(001) lattice generates strain and leads to the formation of monodisperse Pd-Te nanoclusters. Forming monodisperse Pd-Te-based structures provides a path toward the realization of size-selected nanostructures for quantum devices and selective catalytic conversions.

## Introduction

Recent attention to topologically interesting materials, the transition metal dichalcogenides in particular, has led to numerous studies of their unique properties (1–4). 2D WTe_2_(001), with its topologically insulating surface and protected quantized conducting edge states, has been at the center of attention of spintronic device applications (5–11). Equally as fascinating, bulk WTe_2_ is a type-II Weyl semimetal (12, 13) and has been shown to exhibit large magnetoresistance (14, 15). With quantum devices and integrated quantum/conventional microelectronics systems in mind, there is a significant interest in understanding how the structure and electronic properties of WTe_2_ are influenced by intrinsic surface defects and deposited metals (16–20). Understanding these interactions is critical to integrating WTe_2_ with other device components. Theoretical studies point to the key role of surface vacancies in influencing the topological properties of WTe_2_ (17). While these studies also conclude that excess tellurium (Te) adatoms have little effect on surface electronic properties (17), the effect excess Te may have on the deposition of metals can be significant and must be understood.

Recent studies have demonstrated that palladium (Pd) contacts exhibit superconductivity at the interface with WTe_2_ (20). In the search to fabricate a topological Josephson junction, further studies demonstrated spontaneous nucleation of superconducting PdTe at the Pd-WTe_2_ interface after moderate annealing (21). Within the scope of producing layered nanodevices employing WTe_2_, the study of the initial stages of atomic interactions between contact metals, Pd in particular, with WTe_2_(001) is of great consequence toward tailoring and functionalizing this topologically exotic surface.

In addition to device application, Pd, one of the platinum group elements, is known for its catalytic properties critical to energy conversion and capture applications (22–28). There is a significant body of work exploring relationships between catalytic activity and the size of Pd cluster/nanoparticles size, electronic properties of the supporting substrate, and strain (29, 30). Van der Waals materials, such as WTe_2_, represent a new type of catalytic support, where a fundamentally 2D electronic structure and inherent lattice strain, as well as their ability to bend and twist, provide new opportunities to control the behavior of the supported catalysts. Utilizing these opportunities is predicated on the ability to grow thermally stable, well-defined clusters, which can be challenging due to a weak interaction of Van der Waals materials in the out-of-plane direction.

In this study, scanning tunneling microscopy (STM), X-ray photoemission spectroscopy (XPS), and density functional theory (DFT) were utilized to examine the initial nucleation and growth of Pd and its interactions with Te on WTe_2_(001) as a function of coverage and temperature. Experimental and theoretical findings show that deposited Pd interacts with excess Te readily at room temperature resulting in the formation of clusters of mixed Pd and Te composition we denote as Pd-Te clusters. STM studies demonstrate that the nucleation of the Pd-Te clusters surprisingly does not occur on intrinsic surface defects. Further, the annealing of submonolayer Pd on WTe_2_ at temperatures between 373 and 523 K leads to monodispersed, identically oriented Pd-Te nanoclusters. Theoretical calculations provide a framework for understanding the Pd and Te surface mobility, nucleation of the Pd-Te clusters at room temperature, and the origin of their annealing-induced monodispersity. The described synthesis of Pd-Te clusters on WTe_2_(001) provides a unique pathway for the formation of monodispersed chalcogen-containing metal nanostructures on 2D transition metal dichalcogenides.

## Results and discussion

### Unexpected initial surface interactions of deposited Pd

**Fig. 1.**
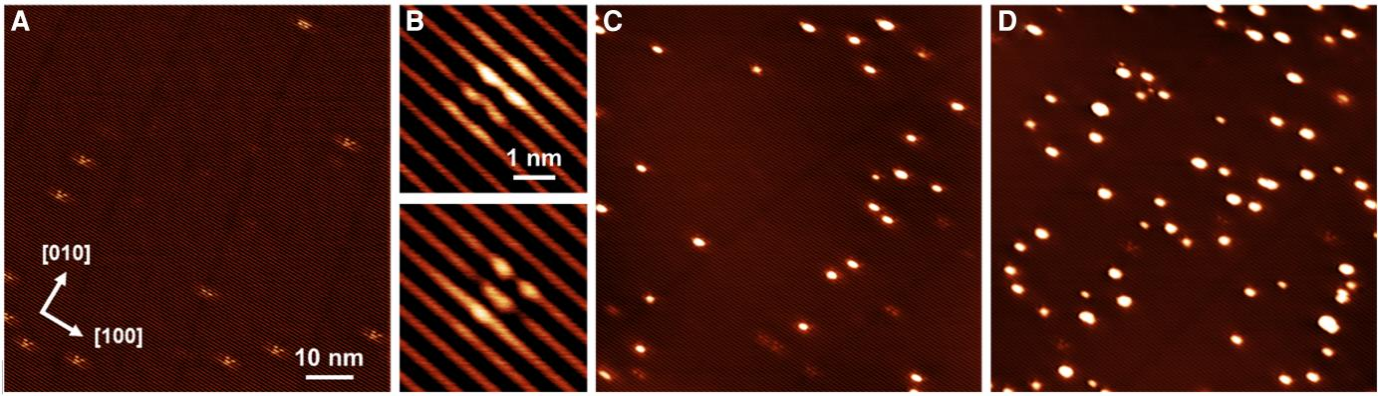
STM images of a) clean WTe_2_(001) and b) the two types of intrinsic defects previously assigned to Te vacancies (31–36). The images of WTe_2_(001) after the deposition of c) 0.003 and d) 0.017 ML of Pd at 293 K; 1 ML of Pd is defined relative to the density of Pd atoms on Pd(111). Imaging conditions: *T*_imaging_ = 80 K, *V*_gap_ = +0.50 V, *I* = 40 pA, a, c, and d) 80 × 80 nm^2^, b) 4.5 × 4.5 nm^2^.

To understand the initial stages of the nucleation and growth of Pd clusters on WTe_2_(001), we deposited Pd at two submonolayer coverages [0.003 and 0.017 ML (monolayers)] at 293 K. STM images for the resulting surfaces are compared with that of bare WTe_2_(001) in Fig. 1. The bare WTe_2_(001) (Figs. 1a and S1a) exhibits large, highly ordered terraces with extremely low defect densities (3 × 10^−4^/unit cell, u.c.). Two types of defects, previously assigned to outer and inner Te vacancies (V_Te_'s) (Fig. 1b, top and bottom, respectively), are observed (31–36).

After deposition, the WTe_2_(001) surfaces covered with 0.003 and 0.017 ML of Pd, defined relative to the density of Pd atoms on Pd(111) (Fig. 1c and d), both show a spatially random distribution of various sized nanometer-scale clusters. From the quantitative analysis of the STM images, cluster densities of 7.5 and 25 × 10^−4^ clusters/u.c. were determined for the 0.003 and 0.017 ML Pd coverages, respectively. While the amount of deposited Pd increased more than five times, the cluster density increased by only a factor of 3.5, which indicates that the nucleation of new clusters is strongly preferred over the growth of the existing ones. This is consistent with the slight increase in the average cluster diameter (from 1.9 to 2.1 nm), as observed by comparing Fig. 1d and c. The deposited Pd amount and cluster density in Fig. 1c allow us to estimate, as deposited, there are an average of ∼16 Pd atoms per cluster. The size dependence of Pd clusters on the initially deposited Pd amount demonstrates that Pd diffusion at 293 K is rather limited. In both Pd deposition cases, the observed cluster density is larger than the density of intrinsic defects (3 × 10^−4^ clusters/u.c.), yet defects remain uncovered. Hence, at 293 K, defects do not appear to participate in Pd nucleation. This is surprising, as chalcogenide defects often serve as primary nucleation centers for metal atoms on TMD surfaces (37–40). The involvement of these intrinsic defects in Pd nucleation is addressed below in the discussion of the temperature-dependent studies.

**Fig. 2.**
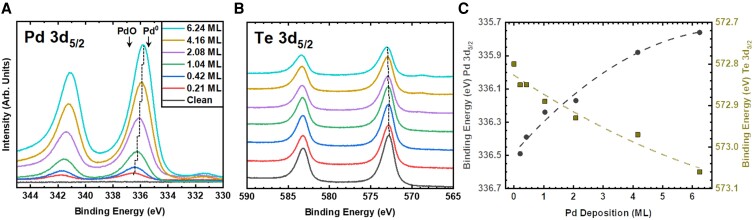
Core-level XPS spectra of WTe_2_ as a function of sequential Pd deposition at 293 K for the a) Pd 3d region and b) Te 3d region. The complementary W 4d region is shown in Fig. S2. The c) changes in the 3d_5/2_ peak binding energy for Pd (circles) and Te (squares) as a function of deposited Pd amount, lines provided to guide the eye.

To further examine the properties of Pd on WTe_2_(001), higher Pd coverages (0.21 to 6.24 ML) were sequentially deposited on WTe_2_(001) at 293 K. These larger quantities of Pd allow for a detailed compositional analysis of the system via XPS. The deposition-dependent Pd 3d and Te 3d XPS spectra are shown in Fig. 2, and the complementary W 4d XPS spectra are displayed in Fig. S2. The reference spectra for bare WTe_2_(001) place the core-level maxima of the Te 3d_5/2_ and W 4d_5/2_ at 572.8 and 243.0 eV, respectively, in good agreement with previously reported values (41–43). The lack of additional Te-O peaks commonly observed on air-oxidized WTe_2_(001) surfaces at 576.1 and 586.6 eV (41) attests to the atomic cleanliness of the surface as observed in the STM images (Figs. 1a and S1a).

As the Pd coverage increases from 0.21 to 6.24 ML, the Pd 3d_5/2_ binding energy shifts continuously from 336.5 to 335.8 eV (Fig. 2a). This shift suggests that there are significant changes in the Pd oxidation state. For the lowest Pd deposition of 0.21 ML, the Pd 3d_5/2_ binding energy value of 336.5 eV is close to the 336.8 and 336.7 eV values reported for oxidized Pd in PdO and PdTe_2_, respectively (44, 45). For the highest deposited amount of Pd, 6.24 ML, the 335.8 eV binding energy of the Pd 3d_5/2_ approaches 335.4 eV reported for metallic Pd (44), which indicates the formation of large metallic Pd particles.

The examination of Te 3d (Fig. 2b) and W 4d (Fig. S2) spectra provides further insight into the origin of observed Pd oxidation. With increasing Pd coverage, the Te 3d_5/2_ peak broadens and its peak position shifts from 572.8 to 573.0 eV approaching the 573.1 eV value reported for Te 3d_5/2_ in PdTe_2_ single crystal (45). This shift points to charge transfer between Te and the deposited Pd. While XPS indicates the interaction of Te at the WTe_2_(001) surface, the W 4d XPS core-level region spectra show only the expected exponential decay in intensity as a function of Pd deposition with no changes in either W 4d_5/2_ peak position or shape (Figs. S2 and S3). This lack of a shift in the W 4d_5/2_ suggests that the chemical interactions are limited to those between surface Pd and Te, and changes in the electronic structure of the underlying bulk WTe_2_(001) are negligible.

The concomitant changes in Pd 3d and Te 3d are consistent with the formation of a Pd telluride compound (45–47). The oxidized Pd component dominates the Pd 3d XPS at small deposited amounts where a negligible fraction of metallic Pd is present. Further shifts toward oxidized Te dominate the Te 3d XPS at large Pd deposited amounts, where Pd attenuates the contribution from the underlying WTe_2_. These observations suggest that surface Te can bind a small amount of Pd, but the interaction is quenched with larger amounts of deposited Pd. Overall, the evolution of the XPS suggests that the small Pd clusters observed with STM, which are the primary focus of this work, react with Te at room temperature, forming Pd-Te clusters.

### The transport of excess Te on and to the surface

The formation of Pd-Te clusters, as discussed, is predicated on the availability of Te. Prior studies (19, 48) have demonstrated that chemical vapor transport (CVT)–grown WTe_2_ single crystals contain excess Te intercalated between the WTe_2_(001) layers. To assess the feasibility of the release of intercalated Te, a DFT analysis of Te diffusion pathways along and across WTe_2_ sheets was performed.

**Fig. 3.**
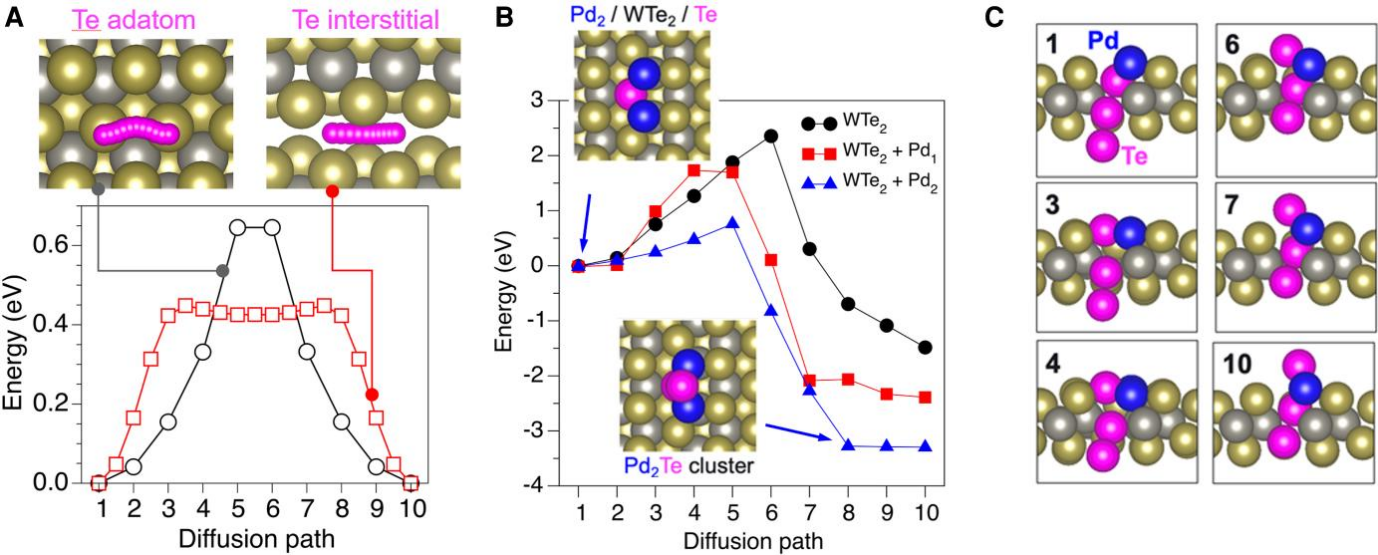
a) The diffusion of Te_ad_ on WTe_2_ surface (left schematic, top view) and Te_int_ between the WTe_2_ layers (right schematic, side view) with corresponding potential energy profiles. b) Potential energy profile for the diffusion of Te atoms through a bare (circles) and Pd covered (one and two Pd atoms shown with squares and triangles, respectively) calculated in the bilayer slab model, where the distance between two WTe_2_ layers was fixed at the corresponding bulk value (7.03 Å). The insets provide top views of the initial and final structure for Pd_2_/WTe_2_ case. c) Initial (1), final (10), and several intermediate configurations (images 3, 4, 6, and 7) illustrate the Te_int_ diffusion path. The stationary W and Te atoms in the lattice are shown in gray and gold, respectively. The Te atoms participating in the diffusion process and Pd atoms are shown in magenta and blue, respectively.

The prerequisite for the formation of Pd-Te clusters on WTe_2_(001) is the facile diffusion of Te adatoms (Te_ad_) on the surface terraces. The lowest energy pathway is found to be along the rows of surface Te_in_ atoms with a barrier of 0.62 eV, as shown in Fig. 3a. Here and below, Te_in_ and Te_out_ refer to the positions of Te atoms along the lower and upper Te chain of WTe_2_(001), respectively. Using the diffusion barrier of 0.62 eV and the standard value of the kinetic preexponential factor of ∼10^13^ s^−1^ (49), the Te diffusion rate is estimated to be ∼200 sites per second at 293 K. With respect to the slow Pd deposition rate (0.0035 ML/s), the reaction of diffusing Te adatoms with the deposited Pd atoms is plausible.

Considering the excess Te in the WTe_2_ bulk (19), the most likely source of the Te_ad_ is Te intercalated between the WTe_2_ layers. This excess Te can reach the surface via diffusion out at the step edges or a direct diffusion through the topmost WTe_2_ layer. To illustrate the former mechanism, the diffusion path of the interstitial Te atom (Te_int_) trapped between two WTe_2_ layers, and the corresponding barrier, was calculated using a bulk WTe_2_ model. Here, we used the supercell formed by the 4 × 2 × 1 extension of the crystallographic WTe_2_ cell corresponding to the vendor sample specification (13.9 × 12.5 × 14.1 Å^3^) and 2 × 2 × 2 *k*-mesh. Alignment of the Te_in_ and Te_out_ in the neighboring layers (Fig. 3a, right schematic) creates a different local environment for Te_int_ than for Te_ad_, even though the diffusion direction along the Te rows is the same. While the site located between two Te_in_ atoms at the surface corresponds to the barrier for Te_ad_ diffusion, a similar site in the bulk corresponds to a shallow energy minimum. As a result, the distance between the energy minimum and the barrier shortens by approximately a factor of 2. The calculated barrier is 0.44 eV, indicating that Te is more mobile when sandwitched between the WTe_2_ layers than on the surface. This suggests that Te can escape onto the surface at the sheet boundaries, such as step edges.

In contrast to the facile diffusion along the WTe_2_ surfaces and interfaces, the Te_int_ diffusion through the top layer is hindered by a large energy barrier of 2.35 eV (black trace, Fig. 3b) and is, therefore, an unlikely origin of Te_ad_. However, the Te_int_ diffusion barrier decreases dramatically in the presence of Pd surface atoms. For the case of one and two neighboring Pd atoms on the surface, the barrier decreases to 1.73 and 0.76 eV, respectively (Fig. 3a). The Pd-assisted diffusion path (Fig. 3b) can be separated into two stages. First, the surface Te atom leaves its lattice site and binds to two neighboring Pd atoms, thus creating a precursor Te vacancy (V_Te_) configuration and the Pd_2_Te cluster (image 3 in Fig. 3c). Then, this vacancy is backfilled via a concerted displacement of another lattice Te and Te_ad_, coupled with further outward displacement of the surface Te that completes the formation of the Pd_2_Te cluster. These results suggest that the deposition of Pd can promote the diffusion of interstitial Te atoms from the subsurface to the surface, where in addition to the other discussed diffusion pathways, Te_ad_ can participate in the formation of Pd-Te nanoclusters.

### Room temperature nucleation of Pd-Te nanoclusters

To understand the initial stages of the formation of Pd-Te clusters, we examined the stability of these clusters depending on the nucleation site and composition using ab initio simulations. Our initial investigations centered on the nucleation of pure Pd clusters on an ideal WTe_2_(001) terrace and on V_Te_ defects. Since we have already established that the observed clusters contain both Pd and Te, an in-depth analysis of the growth of pure Pd clusters is provided in Section S3 and Figs. S4 and S5. We conclude that the isolated Pd atom binds preferentially above W atoms, between two Te_in_ and one Te_out_ atoms, with the binding energy of 3.4 eV as calculated relative to the gas-phase Pd atom. The calculated diffusion barriers of such isolated Pd atoms along and across the Te rows are 0.66 and 0.82 eV (Fig. S4), respectively. The clustering of Pd atoms leads to the formation of Pd chain clusters along the Te rows (Fig. S5a) with a small Pd-Pd binding energy (<0.15 eV/Pd) (Fig. S5b). Hence, the formation of pure, stable Pd clusters at room temperature on pristine WTe_2_(001) is unlikely.

**Fig. 4.**
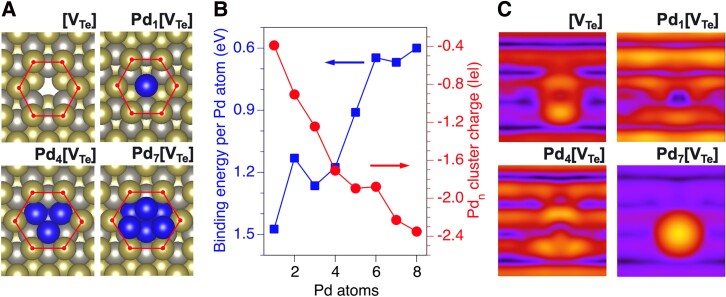
Pd anchored at Te vacancy (V_Te_). a) Arrangement of the Pd atoms (dark spheres) at the V_Te_ site for several Pd*_n_* clusters (*n* = 1, 4, and 7). Te atoms near the vacancy site are indicated with the red hexagon; W and Te atoms in the lattice are shown in gray and gold, respectively. b) Formation energy per Pd atom for *n* = 1–8. The decreased cluster stability between *n* = 4 and *n* = 5 indicates that the transition from Pd_4_[V_Te_] to Pd_5_[V_Te_] is thermodynamically unfavorable. c) Simulated STM images for the Te vacancy and selected Pd*_n_*[V_Te_] configurations are shown in a); the STM images show the entire area of the WTe_2_ supercell.

Contrary to the interaction of Pd on pristine WTe_2_, simulations of Pd binding at the most stable Te vacancy site (Te_in_ vacancy, Fig. 4a) on WTe_2_ show that a single Pd atom binds to V_Te_ with an energy gain of 1.5 eV (Fig. 4b). Pd occupies V_Te_, effectively forming a substitutional defect, PdTe. This defect can trap up to three additional Pd atoms with average binding energies of ∼1 eV/Pd, thus forming a Pd_4_ cluster anchored at V_Te_ (Fig. 4a). Accordingly, the electron charge associated with the vacancy is trapped at the Pd atoms, as shown in Fig. 4b. The attachment of additional Pd adatoms to this cluster is thermodynamically less favorable, as reflected by the decrease of the average Pd binding energies for *n* > 4. This propensity to nucleate Pd on V_Te_ defects contrasts with the surprising lack of Pd nucleation on defects (previously attributed to Te vacancies) observed at 293 K (see Fig. 1).

The much higher stability of Pd*_n_*[V_Te_] (*n* ≤ 4) clusters compared with that of Pd*_n_* clusters on stoichiometric WTe_2_ (Fig. S5) implies that if V_Te_'s are present at the surface, Pd anchoring at the V_Te_ sites would dominate the initial stages of Pd growth. This high stability would result in the formation of Pd*_n_*[V_Te_] (*n* ≤ 4) clusters. However, this theoretical prediction is in contrast with the experimental observations (see Fig. 1), where defects, previously attributed to the V_Te_'s, remain uncovered after Pd deposition at 293 K. The theoretically determined properties of the Pd clusters in V_Te_'s are also inconsistent with XPS and STM data. First, the localization of two electrons formally associated with V_Te_ is predicted to shift the Pd 4p levels to lower binding energies, which is opposite to the shift observed in XPS. Second, the simulated images of the Pd*_n_* clusters (*n* ≤ 4) in the V_Te_'s (Fig. 4c) show no resemblance to the Pd clusters in the experimental STM images (Fig. 1). While larger clusters, such as Pd_7_[V_Te_], have simulated STM images (Fig. 4c) qualitatively matching the experimental ones, the formation of these clusters is predicted to be thermodynamically much less favorable than the formation of Pd_4_[V_Te_] clusters.

**Fig. 5.**
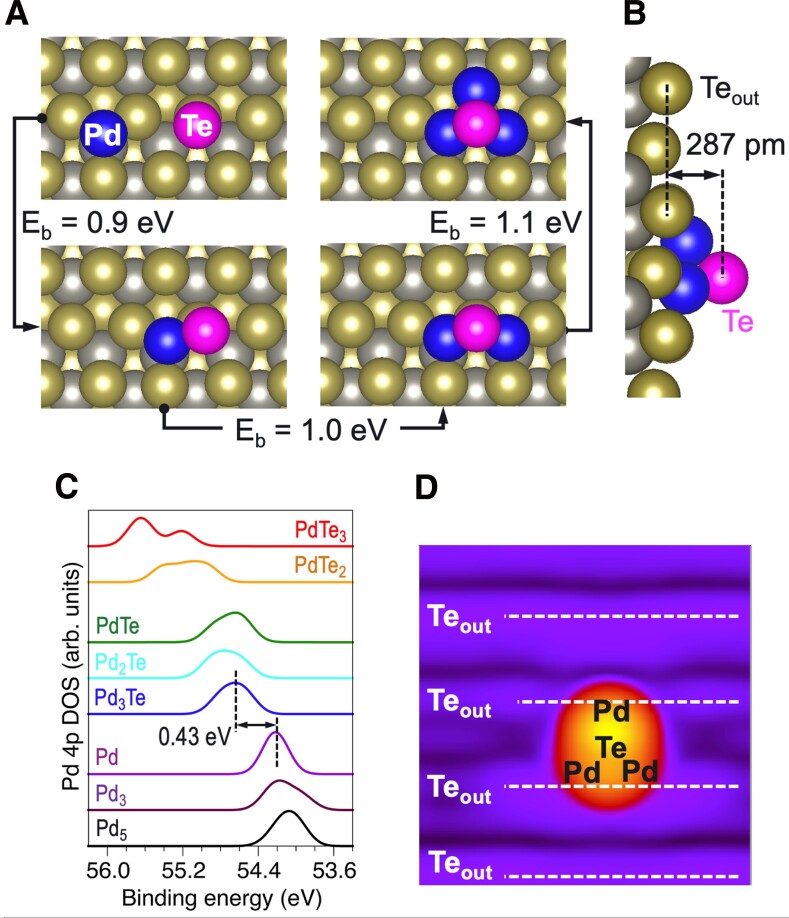
a) The interaction of deposited Pd atoms with a Te adatom on a pristine WTe_2_ surface leads to the formation of a Pd_3_Te cluster with the binding energy E_b_ of ∼1 eV per Pd atom. b) Side view and nominal height of the Pd_3_Te_1_ cluster. c) One-electron DOS of the Pd 4p^6^ states for clusters containing three and five Pd atoms (see Fig. S5a for the cluster structure) representing Pd-rich limit, Pd adatom, and selected Pd*_n_*Te*_m_* clusters with *n*/*m* ratio ranging from 3 to 1/3, corresponding to progressively higher Pd oxidation states (Fig. S6). In all cases, the DOS is aligned so that the electrostatic potential in the vacuum gap is 0. d) Simulated STM image of the Pd_3_Te cluster.

We now turn to the nucleation of mixed Pd-Te clusters on pristine WTe_2_. The calculated binding energy of the Pd_1_–Te_1_ pair from isolated Pd and Te adatoms is 0.9 eV (Fig. 5a). Adding a second Pd atom to this Pd_1_–Te_1_ pair leads to another 1.0 eV gain, and finally, trapping of a third Pd atom, thus forming a Pd_3_Te cluster, provides an additional 1.1 eV energy gain. Interestingly, the Pd-Pd distances in the Pd_3_Te cluster (2.87 and 3.15 Å) are close to the interatomic distances in bulk Pd (2.73 Å). The nominal height of this Pd_3_Te cluster, defined as the distance between the plane of Te_out_ atoms and the apex Te, as shown in Fig. 5b, is ∼2.9 Å. The binding energy of an additional (fourth) Pd atom is negligibly small, which suggests that further growth of this cluster is unfavorable unless additional Te atoms are present. The simulated STM image of the Pd_3_Te_1_ cluster (shown in Fig. 5d) is qualitatively similar to the experimentally observed STM images (Fig. 1), which, together with its high stability relative to the isolated Pd and Te adatoms, suggests that Pd clusters capped with Te atoms can be a characteristic structural motif of the deposited Pd clusters.

The one-electron Pd 4p densities of states (DOS) calculated for the Pd*_n_*Te (*n* = 1–3) clusters shown in Fig. 5a are displayed in Fig. 5c. For comparison, Pd 4p DOS spectra of two pure Pd chain clusters (Pd_3_ and Pd_5_ from Fig. S5a) are also shown. As expected, the peak of the Pd 4p DOS shifts to higher binding energies with increasing Te content. The magnitude of the binding energy shift relative to the Pd adatom varies between 0.43 eV for the Pd_3_Te cluster and 1.2 eV for the PdTe_2_ and PdTe_3_ clusters (Fig. 5c), which is in qualitative agreement with the experimentally observed shift (0.7 eV) of the Pd 4d_5/2_ between the nearly metallic state and the low deposition peak maximum shown in Fig. 2a and c.

The high stability of Pd_3_Te clusters suggests that sequential binding of Pd and Te atoms will result in the formation of larger, thermodynamically stable Pd*_n_*Te*_m_* clusters, in which Te adatoms bind Pd atoms together upon deposition. Since the CVT-grown WTe_2_ single crystals are known to be Te-rich with the W:Te ratio reported to be as high as 1:2.8 (19), and as interstitial Te, Te_int_, can readily diffuse to the surface and react with the deposited Pd, we find this model to be consistent with the formation of clusters shown in Fig. 1c and d.

### Temperature-dependent evolution of Pd-Te nanoclusters

The lack of Pd nucleation on intrinsic surface defects at room temperature poses a question: is Pd nucleation on defects limited by Pd mobility or by weak Pd-defect interactions? The observed Pd clustering at 293 K indicates that the Pd is sufficiently mobile on the surface to form nanometer-sized clusters. This is consistent with the moderate calculated diffusion barrier of 0.66 eV. To address this question, annealing- and deposition–temperature-dependent STM studies were performed.

**Fig. 6.**
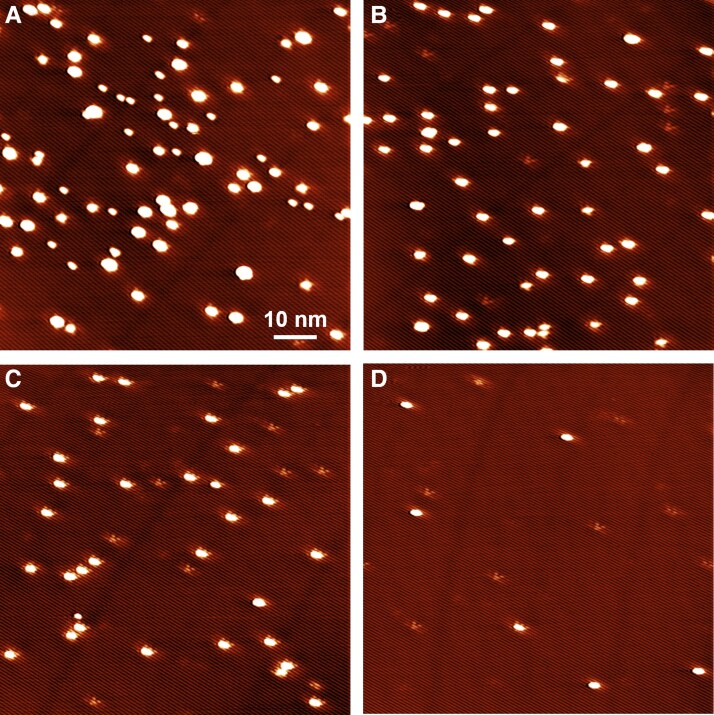
Annealing-dependent STM images of WTe_2_(001) with a 0.017 ML of Pd. a) As deposited at 293 K and annealed to b) 373, c) 523, and d) 553 K. Imaging conditions: *T*_imaging_ = 80 K, 80 × 80 nm^2^, *V*_gap_ = +0.50 V, *I* = 40 pA.

**Fig. 7.**
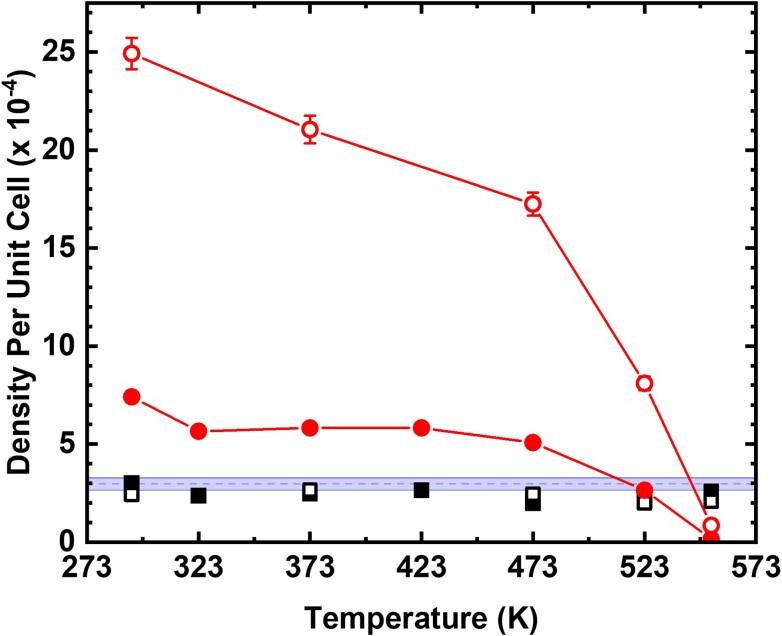
The densities of Pd clusters (circles) and intrinsic surface defects (squares) as a function of annealing temperature for 0.003 (closed symbols) and 0.017 ML (open symbols) of Pd deposited at 293 K. Lines are provided to guide the eye.

The clusters deposited at 293 K show a distribution of sizes, with intrinsic defects remaining bare, as illustrated in Fig. 6a for 0.017 ML Pd. However, the structure and the distribution of these clusters change with annealing. The changes induced by the annealing at 373 and 523 K are illustrated in Fig. 6b and c. Surprisingly, the initial 293 K deposited clusters with a wide distribution of sizes (Fig. 6a) are transformed into mostly monodispersed nanometer-sized clusters (Fig. 6b and c). This is in contrast with expected ripening processes, generally induced by annealing (50). The cluster density decreases slightly, indicating relatively strong binding and stability of Pd-Te clusters on the surface. Importantly, despite the cluster density decrease and disappearance of very small clusters, the formation of intermediate larger clusters is not observed, contrary to the expected annealing-induced sintering. A similar annealing-induced evolution is observed for 0.003 ML Pd (Fig. S1). The changes in Pd-Te cluster densities as a function of annealing temperature are further quantified in Fig. 7 for both 0.003 ML (solid circles) and 0.017 ML (open circles) of deposited Pd.

Ultimately, all nanoclusters disappear after annealing above 553 K (Fig. 6d), and a mostly clean surface is recovered. Sparse large nanoparticles (10–50 nm), such as those shown in Fig. S7, are observed due to sintering in which the intermediate steps are not immediately observed, indicating that the Pd is still on the surface. A detailed analysis of these large nanoparticles and their structure is provided in Section S5 and Figs. S7–S9.

Note that intrinsic defects (fainter features in Fig. 6b–d) remain uncovered after the annealing, i.e. the annealing-induced Pd diffusion and Pd-Te cluster restructuring do not lead to nucleation on defects. The persistence of bare intrinsic defects across the whole range of annealing temperatures is quantified in Fig. 7 (square symbols). The absence of any significant interaction between Pd atoms and intrinsic defects is further confirmed by the lack of Pd cluster nucleation during the deposition at elevated temperatures (see 423 K deposition in Fig. S1d). Here, at elevated deposition temperature, the cluster density decreased by a factor of 7 relative to the deposition at 293 K, with larger clusters indicating facile Pd diffusion. Since the calculations show a strong propensity for Pd nucleation on V_Te_'s, we conclude that the nature of observed defects is more complex than simply V_Te_'s.

A detailed comparison of the temperature-dependent distributions of the apparent cluster heights and diameters is provided in Fig. S10. While the STM measurements suggest that the average apparent cluster size increases from 1.9 nm (2.1 nm) at 293 K to 2.1 nm (2.3 nm) at 523 K for 0.003 ML Pd (0.017 ML Pd), the measured values are affected by the convolution with the tip apex. Since the tip varies from experiment to experiment, caution must be exercised when using these values as the true cluster sizes. However, when the dispersity of the nanocluster diameter within each image is normalized with respect to the average image diameter, the influence of the tip apex convolution can be removed. This analysis shows that there is significant tightening in the distribution of cluster diameters upon annealing for both 0.003 and 0.017 ML Pd depositions, and the full width at half maximum (FWHM) of these distributions decreases by a factor of 1.6 and 2.6, respectively.

In contrast to cluster diameter, the height distributions are mostly unaffected by the tip shape. Therefore, they can be directly used to compare the changes in the apparent height distributions during the annealing sequences. The distributions of as-deposited cluster heights for both coverages show a substantial narrowing after annealing at 373 K, and the clusters appear to converge to a single stable apparent height of 2.2–2.5 Å after annealing at 523 K (see Fig. S10). Accordingly, the FWHM of the height distribution decreases by more than a factor of 5 for both the 0.003 and 0.017 ML Pd coverages.

### Monodispersed Pd-Te clusters

**Fig. 8.**
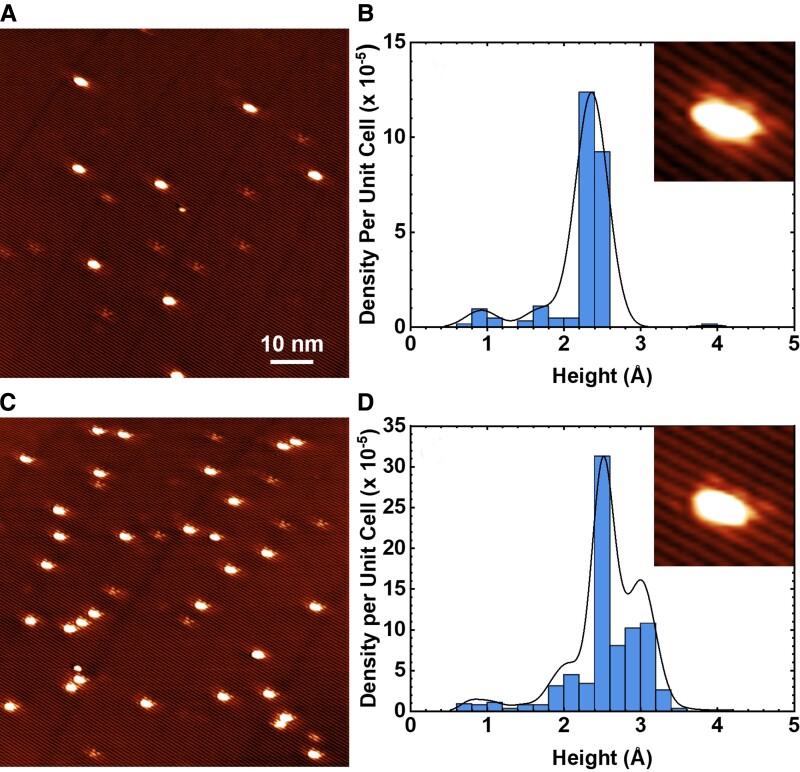
STM images WTe_2_ after a) 0.003 and c) 0.017 ML deposition of Pd at 293 K and annealing at 523 K. b and d) The statistical distribution of measured Pd-Te cluster heights from ∼1 × 10^6^ nm^2^ area. Insets show high-resolution STM images of typical Pd-Te clusters with the modulation of contrast in the surrounding surface structure. Imaging conditions: *T*_imaging_ = 80 K, 80 × 80 nm^2^, 5.5 × 5.5 nm^2^ (inset), *V*_gap_ = +0.50 V, and *I* = 40 pA.

The high magnification images of the monodispersed clusters (insets in Fig. 8) show that each cluster spans over two grooves (Te rows, where upper and lower Te positions are not separately resolved) and generates an extended electronic modulation within the WTe_2_(001) surface. This monodisperse asymmetric cluster configuration (Fig. 8c) has an average apparent FWHM width of 1.5 nm along the [010] direction and a length of 2.4 nm along the [100] direction, with an asymmetry ratio of 1.6. The substrate charge modulation is reminiscent of that of bare intrinsic defects (see Fig. 1b).

**Fig. 9.**
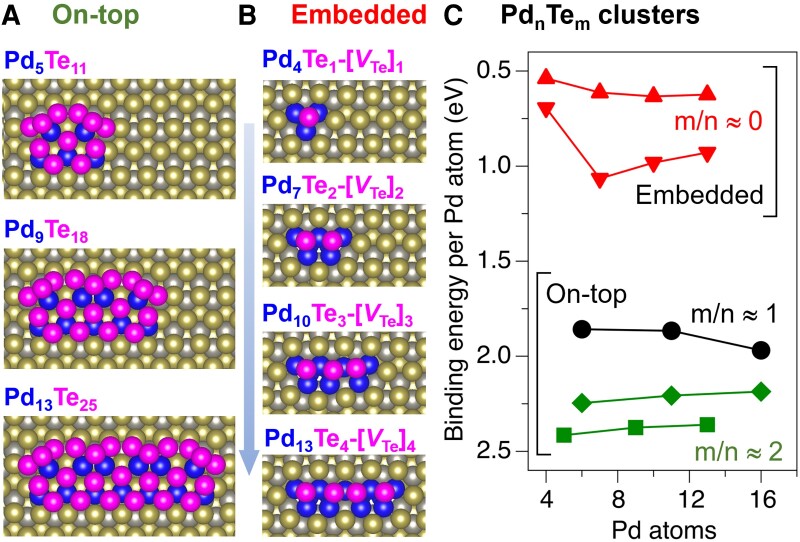
a and b) Structures of selected Pd-Te clusters on the WTe_2_(001) surface. a) Clusters on top of the surface, formed by reacting Pd and Te adatoms, as a function of increasing length along the WTe_2_ grooves. b) Clusters embedded in the surface, formed by displacing the lattice Te by Pd into the adatom configuration, as a function of increasing length along the WTe_2_ grooves. c) Binding energies calculated relative to isolated Pd and Te adatoms and normalized to the number of Pd atoms for three series of on top (circles, rhombuses, and squares) and two series of embedded (triangles up and triangles down) clusters. Energies corresponding to clusters in a) and b) are shown using squares (m/n ≈ 2) and triangled down (m/n ≈ 0), respectively.

To illustrate the possible structural motifs associated with monodispersed Pd-Te clusters observed after annealing, we carried out DFT simulations of the cluster size-dependent stability. Two types of Pd-Te clusters growing along the WTe_2_ grooves were considered: the first type (Fig. 9a) is formed on top of the pristine WTe_2_(001), while the second type (Fig. 9b) is embedded in the WTe_2_(001) by exchanging the Te lattice atoms with Pd.

In the first case, we constructed a series of Pd-Te cluster structures based on one of the most thermodynamically stable structural motifs (see the top panel in Fig. 9a) that can form upon the interaction of deposited Pd atoms and excess Te atoms. For example, the binding energy (E_b_) of a Pd_5_Te_11_ cluster, calculated with respect to isolated Pd and Te adatoms on the surface, is ∼2.40 eV per Pd atom. By extending this structural motif along the WTe_2_ grooves, we formed clusters of Pd_9_Te_18_ (E_b_ ∼2.35 eV/Pd) and Pd_13_Te_25_ (E_b_ ∼2.20 eV/Pd) that have gradually decreasing binding energy (green squares, Fig. 9c). We note that the smallest cluster, Pd_5_Te_11_, is the most stable in this series due to the strain that develops due to lattice mismatch between the WTe_2_ substrate and the Pd-Te cluster. Decreasing the fraction of Te adatoms relative to Pd decreases the cluster stability on a per Pd atom basis, as shown in two additional dependencies (green diamonds and black circles, Fig. 9c, cluster structures in Fig. S11a). Formation of the clusters of these types requires facile diffusion of both Pd and excess Te and reorganization of these atoms at the surface.

In contrast, the second type of considered clusters—embedded clusters—requires reorganization of the surface structure and incorporation of Pd into the WTe_2_ layers. We note that in the presence of surface Pd, the energy cost to displace a surface Te to produce a V_Te_–Te_ad_ defect pair decreases from 2.9 to 2.2 eV (as discussed in Section S3 and Fig. S5). Further, intercalation of Pd into the WTe_2_ has already been observed at 453 K (21), indicating the feasibility of such Pd/Te exchange processes.

Given the strong binding of Pd atoms to both V_Te_ (Fig. 4) and Te_ad_ (Fig. 5) and the effect of Pd on the V_Te_–Te_ad_ pair formation energy, it can be expected that as the local Pd concentration increases or local Te concentration deceases, Pd atoms can displace surface Te atoms and occupy V_Te_ sites. Each cluster in this family is created by forming *k* V_Te_ defects, depositing Pd atoms on the V_Te_ site, and capping them the Te atoms. This family of structurally similar clusters is represented by the formula Pd_3*k*+1_Te*_k_*[V_Te_]*_k_* and shown in Fig. 9b. Other similar cluster series with lower thermodynamic stabilities are shown in Fig. S11b. Importantly, such embedded clusters are not expected to form upon Pd deposition at room temperature but may form during annealing. Thermodynamic stability of the embedded Pd-Te clusters as a function of the number of Pd atoms is ascertained in terms of the E_b_ per Pd atom in Fig. 9c (red). The calculated dependence of E_b_ also suggests that lattice strain induced by these clusters increases with the number of atoms, thus limiting the cluster growth.

To conclude, we note that while E_b_ per Pd atom values show that the embedded clusters are significantly less stable than the surface Pd-Te clusters, their stability depends on the local Te chemical potential, as discussed in detail below, which can be a complex function of the WTe_2_ chemical composition, excess Te transport, and annealing protocols. Importantly, based on the calculated binding energies for both embedded and on-top cluster configurations, the interactions between the Pd-Te clusters and the WTe_2_ surface provide the driving force for the formation of monodisperse size-selected clusters, as experimentally observed.

### Te chemical potential-dependent stability of Pd-Te clusters

As Pd-Te clusters appear to form on the pristine WTe_2_(001) surface in the presence of excess Te and form surface embedded clusters when Te is limited, the role of Te availability as a binding species was examined. To this end, ∼100 nonequivalent Pd*_n_*Te*_m_* clusters (both on-top and embedded) with the *m*/*n* ratio ranging from 3 (in PdTe_3_) to 0 (pure Pd) were constructed (see Materials and Methods) to demonstrate cluster stability as a function of Te availability.

**Fig. 10.**
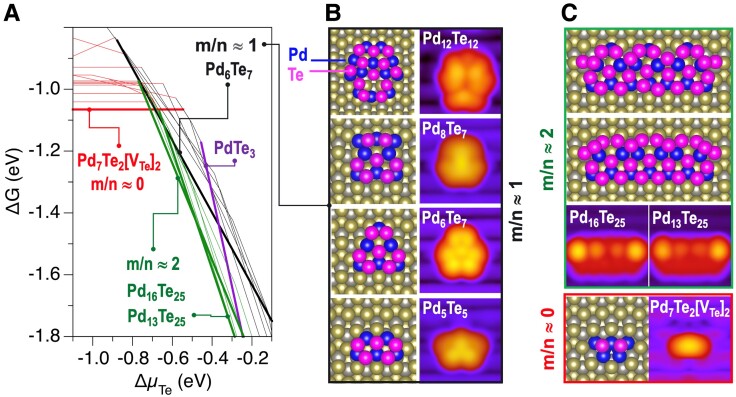
a) Thermodynamic stability of Pd*_n_*Te*_m_* clusters on the WTe_2_ surface as determined using Gibbs free energies ΔG versus Te chemical potential, Δ*μ*_Te_, depending on the relative Te/Pd content: *m*/*n* ≈ 1 (as deposited), *m*/*n* ≈ 2 (Te-rich regime), *m*/*n* ≈ 0 (embedded clusters, Te-deficient regime). ΔG(Δ*μ*_Te_) for the most stable and for other similar configurations are shown with bold and thin lines, respectively. The densely packed lines in the range of Δ*μ*_Te_ from −0.7 to −0.3 eV suggests a wide range of nearly isoenergetic clusters. b) Examples of atomic configurations and the corresponding simulated STM images for the some of most stable Pd-Te clusters formed upon Pd deposition. c) Configurations and STM images for several low-energy Pd-Te configurations that can form after annealing under Te-rich (*m*/*n* ≈ 2) and Te-deficient (*m*/*n* ≈ 0) conditions. All clusters have approximately the same height, defined as shown in Fig. 5B.

The dependence of the Gibbs free energies (ΔG) of Pd*_n_*Te*_m_* clusters on the Te chemical potential (Δ*μ*_Te_), accounting for local Te availability, can be described within four regimes. Configurations of selected clusters and corresponding simulated STM images are shown in Fig. 10. In the limit of low Δ*μ*_Te_ (Δ*μ*_Te_ < −3 eV, not shown), only Pd[V_Te_] defects form (see Fig. 4); i.e. Pd atoms occupying Te lattice sites are stable as the ratio of deposited Pd to V_Te_ is low. At higher Δ*μ*_Te_ (−3 eV < Δ*μ*_Te_ < −1.7 eV, not shown), where the V_Te_ concentration is lower and the Pd:V_Te_ ratio is higher, a larger amount of Pd gets trapped at each vacancy site. The most stable cluster considered here can be viewed as a superposition of two Pd_4_[V_Te_] clusters shown in Fig. 4a at the neighboring V_Te_ sites (Pd_7_[V_Te_]_2_). At yet higher Δ*μ*_Te_ (−1.7 eV < Δ*μ*_Te_ < −0.7 eV), this cluster is stabilized with the addition of two Te adatoms. This resulting configuration can be viewed as Pd_7_Te_2_[V_Te_]_2_; this is the most stable cluster of the Pd_3*k*+1_Te*_k_*[V_Te_]*_k_* series for *k* = 2, shown in Fig. 9b. Finally, at Δ*μ*_Te_ > −0.7 eV, i.e. where significant excess Te is available, a variety of surface Pd*_n_*Te*_m_* clusters (*m*/*n* ≈ 1 and *m*/*n* ≈ 2) that have nearly identical stability exist. Several examples of these clusters and the corresponding calculated STM images are shown in Fig. 10b and c. Note that all the stable on-top clusters give rise to qualitatively similar calculated STM images that are consistent with experimental observation. The likelihood of realization of specific cluster configurations would be therefore determined by kinetic processes responsible for the delivery of the Pd and availability of excess Te atoms. This contrasts with the experimentally observed stability of monodispersed Pd-Te clusters over a broad range of annealing temperatures (293–523 K) and Pd coverages (0.003–0.017 ML). Therefore, we conclude that the observed annealed clusters are more likely to be embedded in the WTe_2_(001) layer rather than grow on top of the surface.

## Conclusion

Using high-resolution imaging, spectroscopic studies, and computational simulations, we have demonstrated that the deposition of Pd on the WTe_2_(001) at room temperature leads to the formation of nanometer-sized Pd-Te clusters that nucleate on ideal terrace sites and not on intrinsic defect sites. Given that ab initio simulations predict strong Pd binding on the Te vacancy sites, which is not observed experimentally, and that WTe_2_ samples have excess Te that do not heal the intrinsic defects, we conclude that the structure of the experimentally observed intrinsic surface defects is likely more complex than a simple Te vacancy.

We show spectroscopically that the Pd-Te clusters and larger structures that form upon room temperature deposition contain both Pd and Te. Upon annealing to moderate temperatures (373–523 K), the Pd-Te clusters transform into a single, thermodynamically stable type that is monodispersed on the surface. The clusters are elongated along the WTe_2_ grooves with an apparent width and length of 1.5 and 2.4 nm, respectively. They have an apparent height of 2.2–2.5 Å, indicating that they are only a single layer high.

Our simulations show that while supported Pd atoms do not exhibit a thermodynamic driving force toward nucleation into Pd clusters, they readily interact with excess Te atoms. These interactions lead to the nucleation of Pd-Te clusters. Regardless of whether the clusters are on top of the surface (in the Te-rich case) or embedded (in the Te-deficient case), the binding energy per Pd atom decreases with the increasing cluster size due to lattice mismatch, thus providing a driving force for limiting cluster size. The stain dependence is manifested more strongly in the case of the embedded clusters, which suggests that observed monodispersity arises due to incorporation of Pd atoms into the WTe_2_ layers.

Finally, annealing at temperatures above ∼553 K results in the sintering of the monodispersed clusters and formation of large (>10 nm) Pd-Te nanoparticles and the restoration of bare WTe_2_(001) surface.

Overall, we conclude that the excess Te present within the Te-based TMDs can dramatically influence the TMD interactions with deposited metals readily at room temperature. Due to high Te mobility and reactivity, the formation of telluride-based compounds can be expected. With the current interest in WTe_2_(001) and the manipulation of its electronic and topological properties, the ability to form monodispersed Te-based structures can provide a unique path toward the realization of size-selected nanostructures for novel quantum devices and selective catalytic applications.

## Materials and methods

### Sample preparation

All WTe_2_(001) sourced samples were grown using CVT (HQ graphene, >99.995% purity). The WTe_2_(001) samples were mounted to sample plates with tantalum foil strips and exfoliated in situ using clear adhesive tape within a load lock with base pressure of better than 1 × 10^−9^ Torr. Following exfoliation, samples were transferred in situ into a modular ultrahigh-vacuum (UHV) chamber with a base pressure better than 2 × 10^−11^ Torr where samples were cleaned via annealing at 553 K with surface contamination monitored using STM. Pd (Alfa Aesar, 99.9% purity) was deposited on the WTe_2_(001) surface using a high-temperature crucible evaporator (Createc) with a source temperature of 1373 K. The Pd deposition rate was calibrated using quartz crystal microbalance (Inficon) and fixed at 0.94 ± 0.17 ng/(s·cm^2^). Using a density of Pd atoms on Pd(111) surface, this deposition flux equates to 0.21 ± 0.04 ML per minute.

### Spectroscopic measurements

The WTe_2_(001) surface was characterized using XPS and low-temperature STM housed within the same UHV system where sample preparation was carried out. STM imaging was performed with a low-temperature (80 K) scanning tunneling microscope (Omicron LT-STM) utilizing electrochemically etched tungsten tips. All imaging shown was performed at 80 K using a constant current mode with a 0.50-V tip bias and a constant current setpoint of 40 pA. X-ray core-level photoemission spectra were acquired using a SPECS X-ray Al anode (hν = 1,486.6 eV) source with an Omicron EA 125 hemispherical energy analyzer with an angular acceptance of ±8° and a collection angle of 64° with respect to surface normal.

### Ab initio simulations

The WTe_2_ surface was represented using the periodic model. A monolayer slab and the lateral supercells of 7 × 4 and 9 × 5 crystallographic cells were used; the interslab distance was >25 Å. Structural configurations of the lattice defects, adsorbed atoms, and clusters and the corresponding formation energies and diffusion barriers were calculated using the DFT method, as implemented in the VASP package (51, 52). The Perdew–Burke–Ernzerhof exchange correlation functional adjusted for solids (PBEsol) (53) and plane-wave–augmented potential (54) representing atomic cores were used throughout. The bulk WTe_2_ structure was optimized using the crystallographic cell (Monkhorst–Pack k-mesh 8 × 8 × 4); the optimized lateral lattice cell parameters (*a* = 3.455 Å; *b* = 6.256 Å, and *c* = 14.060) were used in all subsequent slab simulations. The WTe_2_ slab calculations were performed for the gamma point only; 314 eV plane wave cutoff was used throughout. Surfaces of large Pd clusters were represented using a slab terminated with Pd(111) surfaces. The slab was four atomic planes thick. Atoms of one of the surfaces were fixed at the ideal bulk positions, while all other atoms and the supercell shape were relaxed under the constant volume constraint. The 6 × 6 hexagonal lateral supercell was used to model surface terraces; to model Te adsorption at the surface steps and kinks, several Pd atoms were removed from the top layer of this slab. The lateral supercell parameter corresponds to the preoptimized Pd bulk face-centered cubic (FCC) cell parameter (*a*_Pd-FCC_ = 3.869 Å). The vacuum gap was ∼20 Å. The charge population analysis was performed using the Bader method (55, 56). The projected one-electron DOS were smeared using Gaussian-type functions with the FWHM of 0.1 eV. Diffusion barriers were calculated using the climbing image nudged elastic band (CI-NEB) method and eight image configurations (57). To generate constant charge density STM images, first the charge density distribution associated with one-electron states in the range between the Fermi energy (E_F_) and E_F_ + 0.5 eV was calculated. Then, this charge density was convoluted with a spherical Gaussian-type function with the FWHM of 1.0 Å. The Gibbs free energies of Pd*_n_*Te*_m_* per Pd atom versus Te chemical potential (Δ*μ*_Te_) were calculated using the following equation where *E*(Pd*_n_*Te*_m_*), *E*(Pd), and *E*(Te) are the total energies of the Pd*_n_*Te*_m_* cluster and isolated Pd and Te adatoms, respectively, supported on WTe_2_, and *E*(WTe_2_) is the energy of the defect-free WTe_2_ surface:


$$\begin{array}{rl} \Delta G(\Delta \mu_{\mathrm{Te}})=\frac{1}{n}\{ & \left[E(Pd_{n}Te_{m})+(m+n-1)E(\mathrm{WT}e_{2})]-[nE(\mathrm{Pd})-mE(\mathrm{Te})\right]\}\\ & -\Delta \mu_{\mathrm{Te}}\frac{m}{n}. \end{array}$$

### Generating Pd*_n_*Te*_m_* clusters

To generate the initial configurations of the surface Pd-Te clusters, two rules were adopted: (i) a Te atom can be bound to one to three Pd atoms, and (ii) each Pd atom should be connected to the rest of the cluster by at least one bridging Te atom. To quantify the relative stability of these clusters, Pd supply during deposition was assumed to be unlimited. However, binding energies of individual Pd atoms to preexisting Pd*_n_*Te*_m_* clusters depend on the availability of excess Te that serves as a binding agent. 3D structures are illustrated using VESTA visualization (58).

## Supplementary Material

pgad212_Supplementary_Data

## Data Availability

All experimental data are provided in the manuscript and the supporting information. Theoretically determined structural configurations are combined into a separate supporting information file in VASP POSCAR format.
