# Supplementary material for: Understanding palladium–tellurium cluster formation on WTe2: From a kinetically hindered distribution to thermodynamically controlled monodispersity
Source: PNAS Nexus. 2023 Jun 28;2(7):pgad212. doi: 10.1093/pnasnexus/pgad212 (PMC10321376; doi:10.1093/pnasnexus/pgad212)
Supplement: pgad212_Supplementary_Data [file pgad212_supplementary_data.zip › PNASNEXUS-PNASNEXUS-2023-00358R-s01.pdf]

Supporting Information for

Understanding Pd-Te Cluster Formation on WTe_2: From a Kinetically Hindered Distribution to Thermodynamically Controlled Monodispersity

Prescott E. Evans,[†] Yang Wang,[†] Peter V. Sushko,[†,*] and Zdenek Dohnálek[†,‡,*]

[†]Physical and Computational Sciences Directorate, Pacific Northwest National Laboratory, P.O. Box 999, Richland, Washington 99352, United States

[‡]Voiland School of Chemical Engineering and Bioengineering, Washington State University, Pullman, Washington 99163, United States

*Corresponding Authors: Peter V. Sushko and Zdenek Dohnálek
Emails: peter.sushko@pnnl.gov, zdenek.dohnalek@pnnl.gov

This PDF file includes:

Supplementary text (Sections S1 – S7)
Figures S1 to S11

Section S1. STM images for low coverage annealing sequence and high temperature deposition

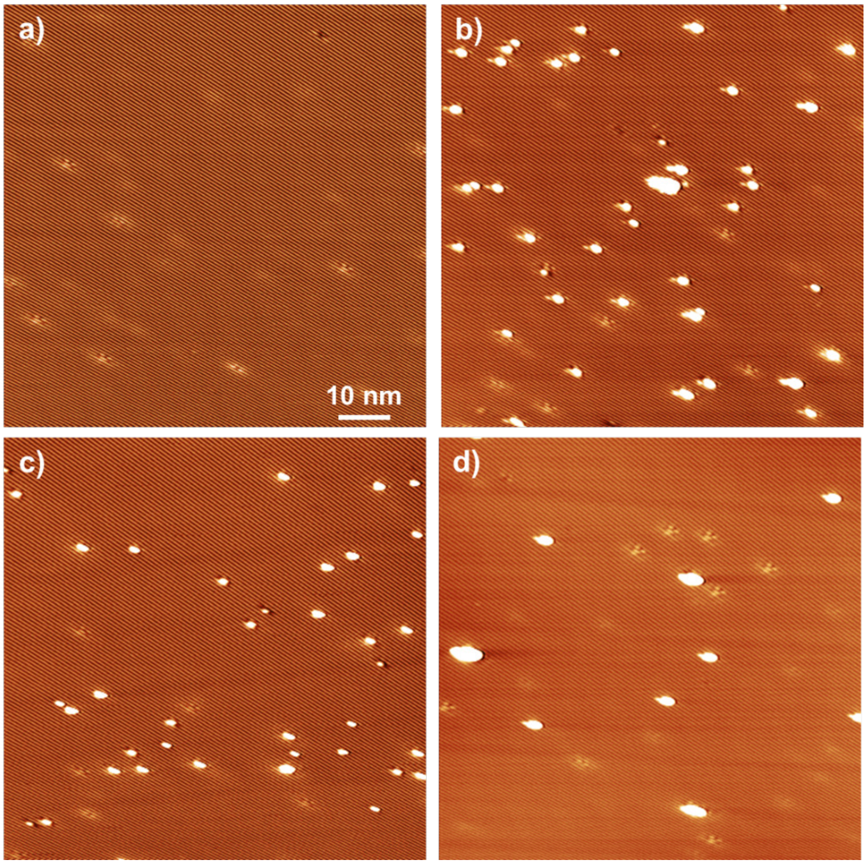

Fig S1. (*A*) Bare $WTe_2(001)$ before Pd deposition. $WTe_2(001)$ after (*B*) 0.003 ML deposition of Palladium at 293 K and (*C*) annealing at 423 K. $WTe_2(001)$ after (*D*) 0.003 ML deposition at 423 K. Imaging conditions: 80 K, 80 x 80 nm^2, V_{gap} = +0.50 V, I = 40 pA.

Section S2. Pd coverage-dependent XPS spectra and deposition evaluation

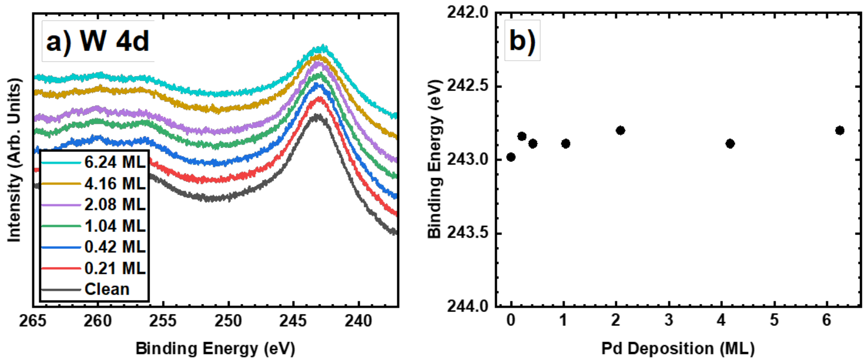

Fig. S2. (*A*) Core level XPS spectra of palladium exposed WTe_2 centered on the W 4d region for deposited palladium amounts and (*B*) the subsequent measured shifts in the W $4d_{5/2}$ peak binding energy.

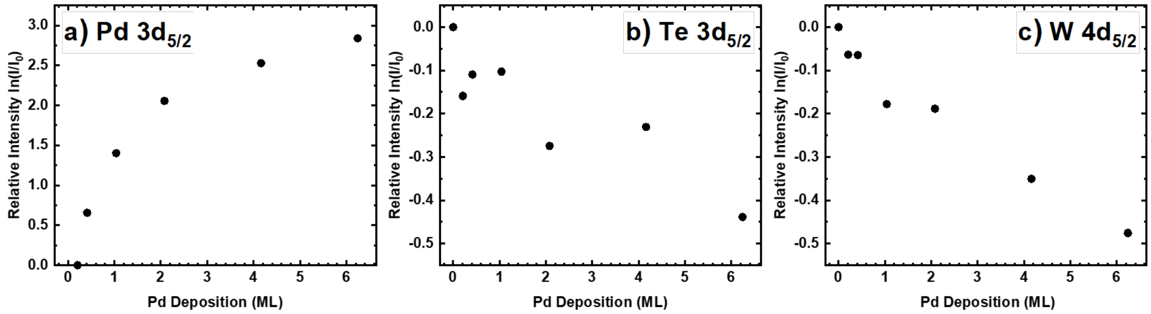

Fig. S3. The natural log of the relative integrated intensity area of the (*A*) Pd $3d_{5/2}$, (*B*) Te $3d_{5/2}$, (*C*) W $4d_{5/2}$ core level XPS, as a function of deposited palladium

Section S3. Pd coverage-dependent XPS spectra and deposition evaluation

Upon initial deposition from the gas-phase, isolated Pd atoms bind to the WTe_2(001) surface with an energy gain of 3.4 eV per atom. The most stable Pd site is above a W atom, between two Te_{in} and one Te_{out} atoms (B1 in Fig. S4*A*), while the second most stable site (B2) is also above a W atom, between two Te_{out} and one Te_{in}. The stability of Pd at the sites A1 and A2 (Fig. S5) are comparable to that at B2; however, the negligibly low barriers for the A1→B1 and A2→B1 interconversion suggest that Pd can occupy these sites only transiently during the surface diffusion processes. Neglecting these configurations puts the barriers for Pd atom diffusion along (B1→B1) and across (B1→B2) the surface rows at ~1.0 and ~0.8 eV, respectively. Hence, the expected diffusion of Pd atoms on the WTe_2(001) surface at room temperature is expected to be rather slow.

Increasing the number of palladium atoms on the surface, two adjacent Pd atoms on two neighboring B1 sites, have a distance of 348 pm, which is too large to form a Pd-Pd bond, and the interaction between Pd-induced strain fields is weak, as manifested by the Pd–Pd binding energy of only 0.08 eV. Instead, the most stable arrangement of the two adjacent Pd atoms occupies B1 and B2 sites (B1-B2 chains thereafter), shown in Fig. S5*A*. The Pd–Pd distance in this chain is ~280 pm, which is close to the interatomic distance in the Pd bulk (273 pm). Accordingly, the relative cost of Pd occupying the 2nd most stable site (B2) is offset by the gain due to the Pd–Pd interactions. The adsorption energy in the Pd clusters forming B1-B2 chains (relative to isolated Pd at B1 site) is shown in Fig. S5*B*. The formation energy increases linearly with the length of the chain and is modulated by the location of the end members. Specifically, the transition of the B1…B1 termination to the B1…B2 termination is associated with a noticeable decrease in the binding energy (Fig. S5*B*), i.e., B1-B2 chains can grow only by adding two Pd atoms at once. Considering this kinetic requirement and the average binding energy of < 0.15 eV per atom, the formation of pure, stable Pd clusters at room temperature are unlikely in accordance with the experimental conclusions

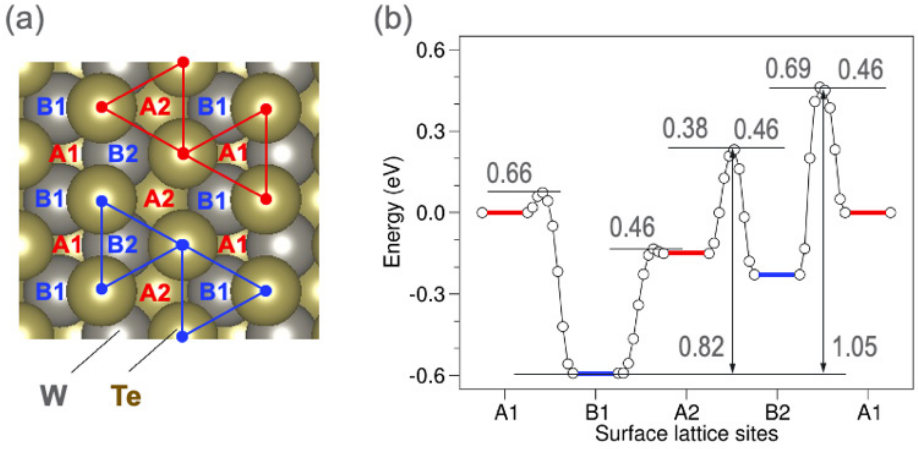

Fig. S4. (*A*) The four Pd adsorption sites on the WTe_2 surface. A1 and A2 are located above Te sites, B1 and B2 are located above W sites. (*B*) Stability of the Pd at each site and the calculated barriers for the site-to-site diffusion. The energy scale is selected relative to Pd at the A1 site.

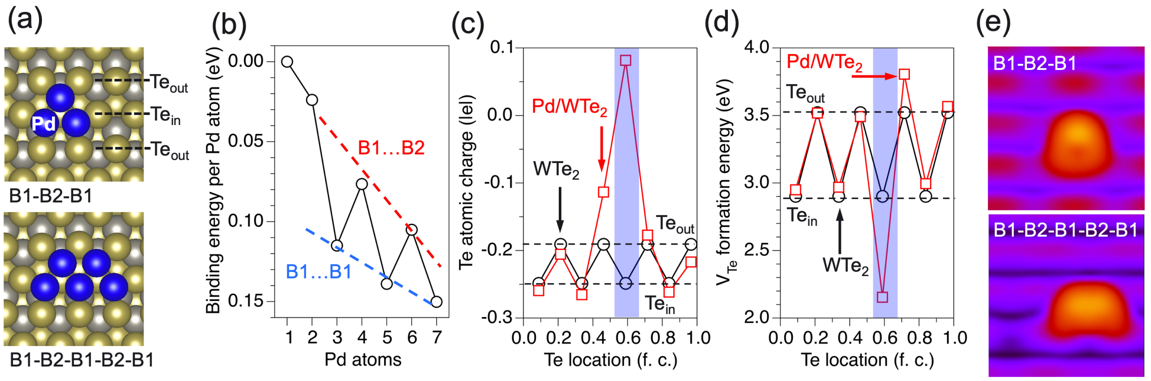

Fig S5. (*A*) Chain-like configurations of Pd atoms located at the B1 and B2 sites along the Te rows on the WTe2 surface. (*B*) The binding energy (per Pd atom) of the B1-B2 chains depending on the location of the terminating Pd. (*C*) Te atomic charges on the pristine WTe2 surface (black) and on the surface containing B1-B2 Pd chain (red). Te atoms located near the B1-B2 chain (blue rectangle) loose some of its electron charge and become relatively unstable, as indicated by the ~0.8 eV decrease in the VTe formation energy in the vicinity of the B1-B2 chain (*D*). (*E*) Simulated STM images for the Pd3 and Pd5 clusters shown in panel (a).

Section S4. Modeling of mixed clusters in the low limit of Pd coverage

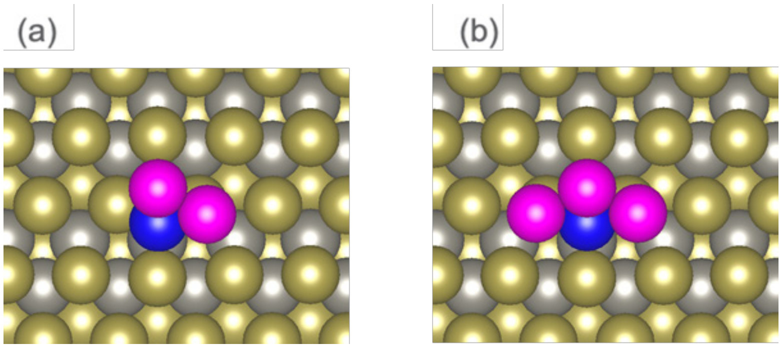

Fig. S6. Clusters used to model Pd (blue) deposited on the WTe_2 (001) surface in the limit of low coverage in the presence of excess Te (magenta): $PdTe_2$ (*A*) and $PdTe_3$ (*B*). See also Fig. 5*C* for the dependence of one-electrons density of states on the Te/Pd ratio.

Section S5. Imaging and Structure of Pd-Te Superstructures and DFT calculations of Te adatoms and substitutional Te on Pd(111)

Annealing dependent palladium deposition studies show the development of large Pd-based nanoparticles after annealing of Pd deposits to 553 K. While extensive structural studies of such nanoparticles, were not carried out, several examples with superstructures on the top terrace were observed (Fig. S7). The example in Fig. S7*A* shows an ordered hexagonal array of bright features that can be due to either Te incorporated into the Pd lattice or Te adatoms sitting on top of the Pd(111) terrace. Line profiles of the spacing on ordered clusters measure between 8 and 10 Å (Fig. S7 *A* and *D*), indicating large clusters are not pure palladium in composition. A second example in Fig. S7*C* is selected to illustrate a structure with more dilute ordered arrangements of the bright features along the step edges and disorder across the top terrace.

The atomic-scale origin of the structural arrangements on the Pd-Te superstructure surface were further examined using ab initio modeling. We evaluated the stability of Te in both substitutional and adatom positions on the Pd(111) surface by comparing the energies of Te adsorption on the defect-free surface and the formation of a substitutional Te_{Pd} defect whereby the Pd is displaced into an adatom configuration. Assuming that the displaced Pd remains at the surface, the substitutional Te_{Pd} was found to be less stable than the adsorbed one by 0.4 eV. However, if the displaced Pd binds to a step edge or a kink at the Pd (111) surface, the formation of substitutional Te_{Pd} becomes thermodynamically preferred. We propose that the latter scenario is unlikely because our calculations suggest that excess Te strongly binds to low-coordinated Pd atoms. Indeed, we find that Te atom binding energy to step edges on Pd(111) surface is 0.85 eV larger than to the terrace. Therefore, we conclude that excess Te preferentially decorates step edges on the periphery of the large Pd particles. Accordingly, displacing surface Pd to these sites is unlikely.

For Te adatoms adsorbed on the Pd (111) surface, it is found that Te is nearly equally (within 0.01 eV) stable at the face-centered cubic (FCC) and hexagonal closest packed (HCP) hollow sites. To establish the most likely Te configurations, energies of the Te-Te interactions

depending on th distance between the Te atoms were calculated relative to the energies of isolated adsorbed Te (see Fig. S8).

According to the Bader analysis, Te donates as much as 0.5 electrons to the surface Pd atoms located in the immediate vicinity of the adsorbed Te atoms, which form a "solvation shell" around it. First, we found that positively charged Te adatoms repel each other if they are close to each other. As the Te-Te distance increases, the repulsive interactions become efficiently screened by surrounding Pd. These calculations indicate that the Te-Te interaction energy becomes negligible when the Te-Te distance is ~0.7 nm or larger. Therefore, at maximum packing density, Te adatoms form a hexagonal arrangement with ~0.7 – 0.9 nm spacing between Te which is consistent with the superstructure spacing observed using STM.

A constant-current STM image (bias +0.5 eV) was also simulated for the close-packed arrangement of Te atoms adsorbed at the FCC hollow sites (Fig. S9 *A* and *B*). The bright spots correspond to the unoccupied states of Te adatoms. Their arrangement and intensities are consistent with the experimental data, further supporting the proposed Te superstructure. An analogous image is found for the Te at the HCP hollow sites (not shown). For comparison, the STM image for the substitutional Te under the same conditions was also simulated (Fig. S9 *C* and *D*). In this case, the simulated pattern is very different from that observed experimentally, which further suggests that substitutional Te is unlikely.

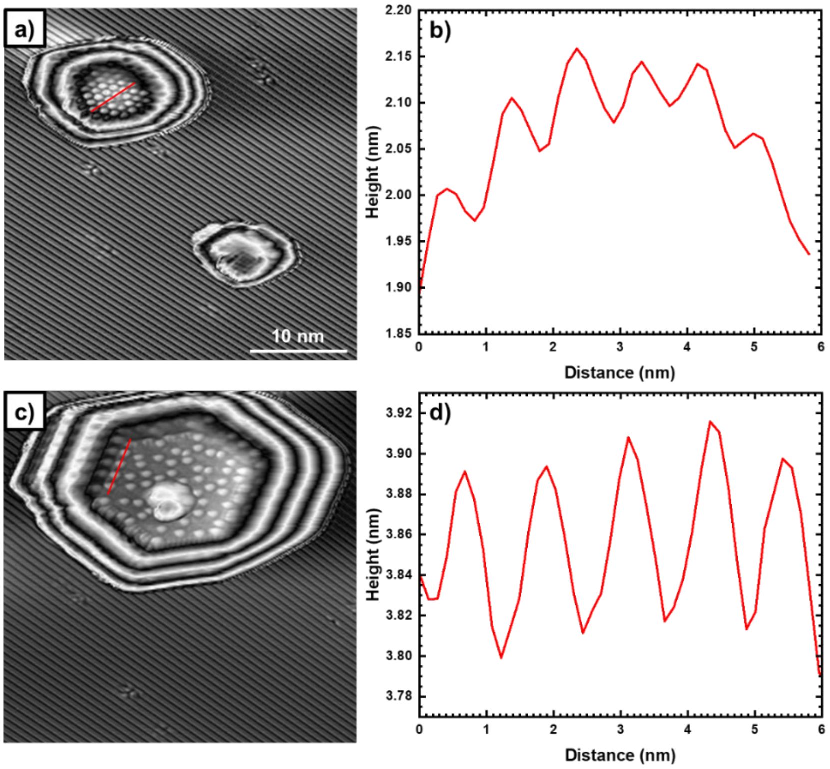

Fig. S7. (*A* and *C*) STM imaging of WTe_2 with a 0.017 ML Pd deposition at 293 K followed by annealing at 553 K as shown in Fig. 6. (*B* and *D*) surface profiles across the corresponding ordered structures marked in red (35 x 35 nm, *Vgap* = +0.500 V, *I* = 40 pA)

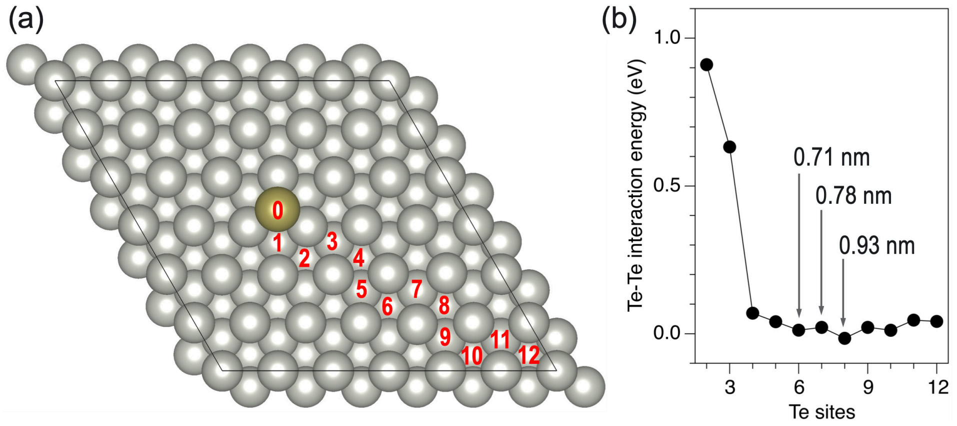

Fig. S8. Estimates of the highest surface density of Te atoms adsorbed on the surface of large Pd clusters. (*A*) Supercell used to calculate the interaction energies of two Te atoms adsorbed on the Pd (111) surface. One of the Te atoms (large gold sphere) was placed at the FCC hollow site "0"; the other Te atom was positioned at the FCC or HCP hollow sites labeled 1–12. (*B*) Comparison of the Te–Te interaction energies calculated for the selected sites shows that this interaction is negligible at distances exceeding ~0.7 nm; at shorter distances they repel each other. Since Te binding energies at the FCC and HCP sites are nearly identical, the adsorbed Te have freedom for form a variety of closed packed configurations with the Te-Te distances in the 0.7–0.9 nm rage depending on the Te availability. The arrangement used for STM simulations (see Fig. S9) corresponds to the Te–Te distance of 0.81 nm.

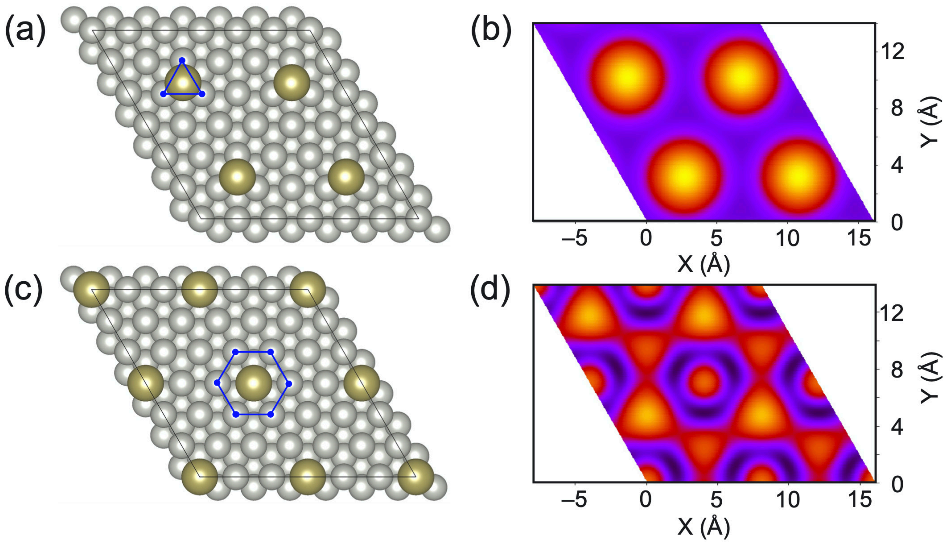

Fig. S9. Selected close-packed configuration of Te on Pd (111) surface and the corresponding simulated STM images; Te–Te distance is 0.8 nm (see Fig. S8). (*A* and *B*) Te adsorbed at the FCC hollow surface sites; (*C* and *D*) substitutional Te sites. Te atoms acquire a charge of ~ +0.5 |*e*| in both configurations. Blue triangle in (*A*) and blue hexagon in (*C*) show surface Pd atoms that acquire negative charge. The simulated STM image in (*B*) is consistent with the experimentally observed superstructure on the surface of large Pd clusters (see Fig. S7).

Section S6. Cluster size distribution as a function of annealing

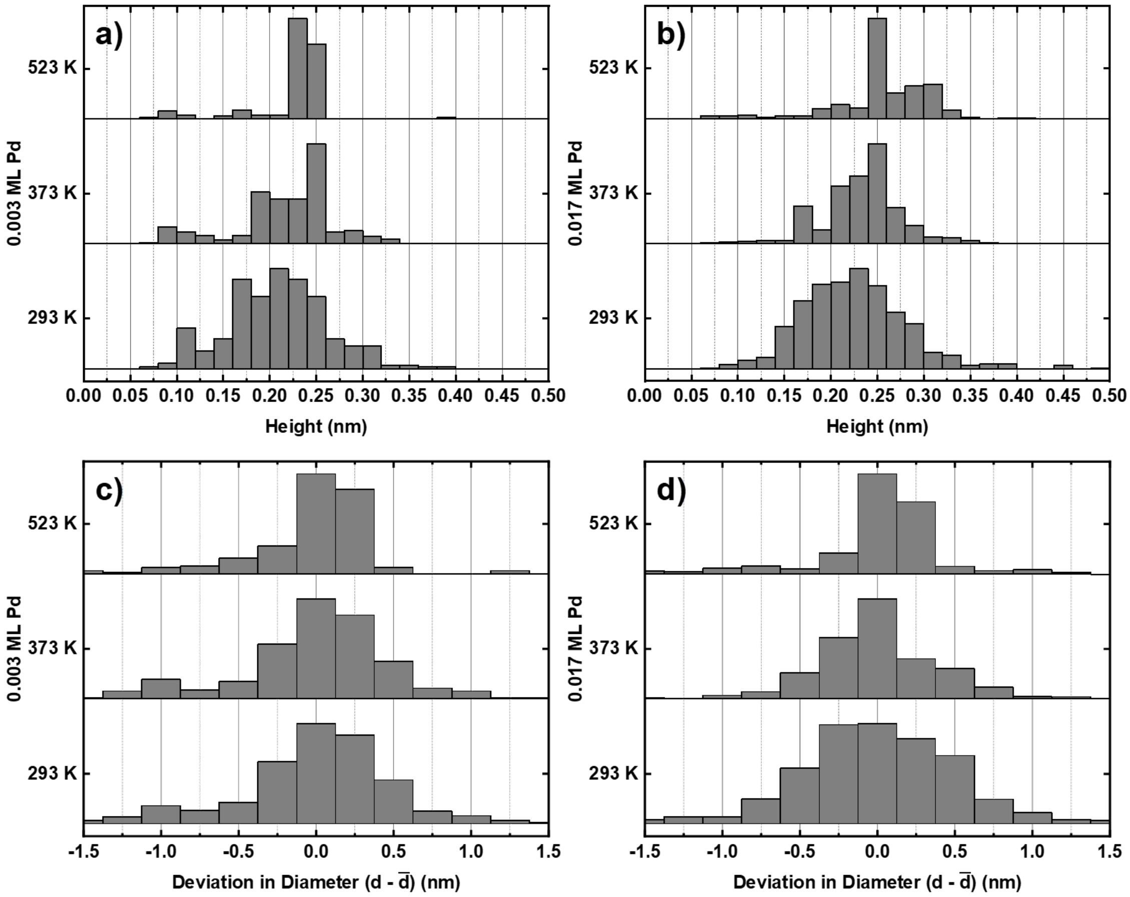

Fig. S10. Height distributions of clusters for (*A*) 0.003 ML Pd deposition for 293 K, 393 K, 523 K and height distributions for (*B*) 0.017 ML Pd deposition for 293 K, 393 K, 523 K. Deviation in diameter of clusters for (*C*) 0.003 ML Pd deposition for 293 K, 393 K, 523 K and for (*D*) 0.017 ML Pd deposition for 293 K, 393 K, 523 K. Deviation in diameter (d-d̄) is used to remove tip convolution within imaging sequencing by centering the distribution about the frame mean diameter.

Section S7. Examples of Pd clusters on the WTe_2 surface and anchored at Te vacancies and capped with Te adatoms

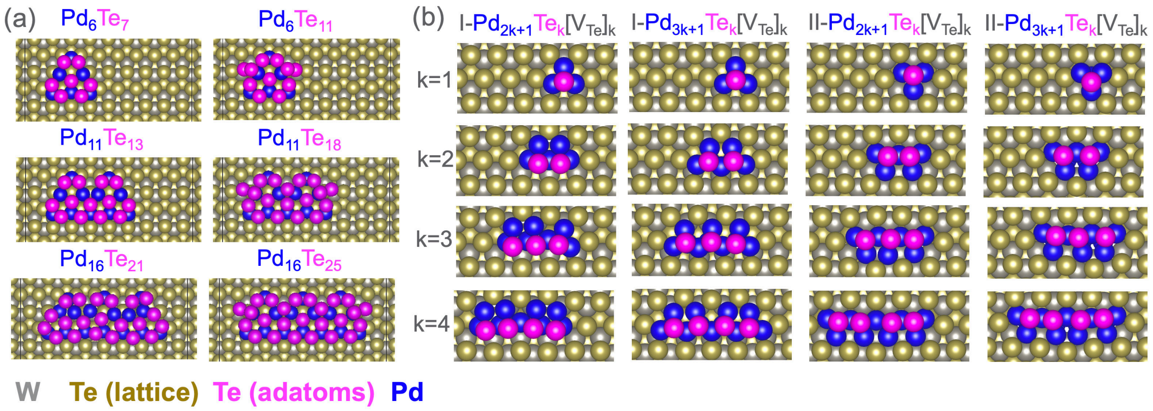

Fig. S11. Examples of Pd_mTe_n clusters located (*A*) on the WTe_2 surface and (*B*) anchored at Te vacancies (V_{Te}) and stabilized by Te adatoms (Te_{ad}). Pd_mT_n clusters with m/n ≈ 1 become disordered with increasing size but retain ordered structure for m/n ≈ 1.5. Clusters of types I and II differ by orientation of the Pd_3 structural units. Value of *k* indicates the number of V_{Te} and Te_{ad}. Te vacancies remain unoccupied in the $Pd_{2k+1}Te_k[V_{Te}]_k$ series and occupied by a single Pd in the $Pd_{3k+1}Te_k[V_{Te}]_k$ series. See also Fig. 9.
