# Supplementary material for: Understanding palladium–tellurium cluster formation on WTe2: From a kinetically hindered distribution to thermodynamically controlled monodispersity
Source: PNAS Nexus. 2023 Jun 28;2(7):pgad212. doi: 10.1093/pnasnexus/pgad212 (PMC10321376; doi:10.1093/pnasnexus/pgad212)
Supplement: pgad212_Supplementary_Data [file pgad212_supplementary_data.zip › PNASNEXUS-PNASNEXUS-2023-00358R-s02.pdf]

Supporting Information for

Understanding Pd-Te Cluster Formation on WTe_2: From a Kinetically Hindered Distribution to Thermodynamically Controlled Monodispersity

Prescott E. Evans,[†] Yang Wang,[†] Peter V. Sushko,[†,*] and Zdenek Dohnálek[†,‡,*]

[†]Physical and Computational Sciences Directorate, Pacific Northwest National Laboratory, P.O. Box 999, Richland, Washington 99352, United States

[‡]Voiland School of Chemical Engineering and Bioengineering, Washington State University, Pullman, Washington 99163, United States

*Corresponding Authors: Peter V. Sushko and Zdenek Dohnálek
Emails: peter.sushko@pnnl.gov, zdenek.dohnalek@pnnl.gov

This PDF file includes:

Structural configurations of the adsorbed Te, Pd, and Pd-Te clusters (VASP POSCAR format) discussed in the manuscript. Each configuration begins with a marker "==" followed by a brief description of the configuration in the same line.

```
== Te atom on WTe2 (001)
   1.00000000000000
    13.8189816962433802    0.0000000000000000    0.0000000000000000
     0.0000000000000000   12.5126822235751405    0.0000000000000000
     0.0000000000000000    0.0000000000000000   34.0000000000000000
   W    Te
    32    65
Selective dynamics
Direct
  0.9998929761688800  0.0112328479828392  0.4211532338114381   F   F   F
  0.0001563611665708  0.5116606917122321  0.4218262813495763   T   T   T
  0.2504182263474942  0.0129115931017208  0.4227310841633203   T   T   T
  0.2501438100902914  0.5140165654068324  0.4220946577878121   T   T   T
  0.5013970774580871  0.0106519311010068  0.4211985402847745   T   T   T
  0.5002240163934504  0.5115287429405686  0.4218555664818952   T   T   T
  0.7503652712648686  0.0114846904568127  0.4211497307722216   T   T   T
  0.7501053980990087  0.5114361086942769  0.4216806927259289   T   T   T
  0.0001010279842077  0.3078678304799922  0.2058549390229558   F   F   F
  0.0004923774747979  0.8090179029004588  0.2049946400916010   T   T   T
  0.2504806272611681  0.3090063867383261  0.2050421214377258   T   T   T
  0.2505130047195860  0.8088517392291545  0.2048824331166033   T   T   T
  0.5004299561175241  0.3090578570781846  0.2049446383679539   T   T   T
  0.5004618114250573  0.8089736912916682  0.2049077585628740   T   T   T
  0.7503068945289944  0.3091217749748371  0.2050779737630639   T   T   T
  0.7504332186174849  0.8090167244775864  0.2049874250350688   T   T   T
  0.1250846172568614  0.4872931678933880  0.2111055370914574   T   T   T
  0.1259389047117717  0.9874053842723183  0.2108828785458345   T   T   T
  0.3757965027573881  0.4876065494594665  0.2107660328632188   T   T   T
  0.3759802453145831  0.9875953535486413  0.2107605893893842   T   T   T
  0.6256330362667047  0.4876491520972889  0.2108565738556570   T   T   T
  0.6256982614199962  0.9876815635949395  0.2108396940588410   T   T   T
  0.8760486330434970  0.4873836668227803  0.2111710288805402   T   T   T
  0.8756442499145171  0.9875234477196851  0.2110230812388661   T   T   T
  0.1194924964727271  0.1885087736263878  0.4157019010596809   T   T   T
  0.1243348824181946  0.6911238270940723  0.4163120097084752   T   T   T
  0.3809428370084840  0.1883125965393249  0.4156507787401512   T   T   T
  0.3762702815221241  0.6909410660655606  0.4163393742980832   T   T   T
  0.6258766680527872  0.1902370251131773  0.4157236531042685   T   T   T
  0.6254063190738912  0.6901839634669967  0.4162248971845658   T   T   T
  0.8744940681677066  0.1903984455885452  0.4157173459979055   T   T   T
  0.8753447252937842  0.6903809784417239  0.4161276659899540   T   T   T
  0.9993287774402262  0.3146774182567795  0.4623893591160797   T   T   T
  0.9998822266530514  0.8165145068115397  0.4622035078015880   T   T   T
  0.2513155261456785  0.2840791362039692  0.5346855269213061   T   T   T
  0.2502810487733950  0.8158547156874058  0.4624372285499608   T   T   T
  0.5008641535123944  0.3144573471296071  0.4623640242285635   T   T   T
  0.5006087379069671  0.8159383394942396  0.4623806476320267   T   T   T
  0.7500847573241549  0.3151937942951601  0.4622180028914320   T   T   T
  0.7502712493307832  0.8158166412155324  0.4621396920656426   T   T   T
  0.1240340508017978  0.0628880555271981  0.4804316669097381   T   T   T
  0.1249713023365085  0.5612100159834361  0.4802075182065776   T   T   T
  0.3771786653694744  0.0628912868335536  0.4803800943739173   T   T   T
  0.3754265156205668  0.5610015204061736  0.4802221943541035   T   T   T
  0.6257350178050203  0.0600695877233970  0.4795657846384920   T   T   T
```

```
  0.6252099862001160  0.5598787298625508  0.4800437693435887   T   T   T
  0.8750107127384218  0.0601983235755576  0.4795655389635224   T   T   T
  0.8750134656623693  0.5602185897836456  0.4799494701912508   T   T   T
  0.1244486777823360  0.3573195842421050  0.1471579197266498   T   T   T
  0.1252790872318502  0.8580745343365997  0.1467068894636178   T   T   T
  0.3751743459030267  0.3578803125004785  0.1467140063677679   T   T   T
  0.3753648846570256  0.8580505355288827  0.1466746154092610   T   T   T
  0.6254284258079809  0.3580543430163828  0.1467410909085736   T   T   T
  0.6253584943618486  0.8580765293178424  0.1467062211302092   T   T   T
  0.8758349052710538  0.3574863345959561  0.1471378157407913   T   T   T
  0.8753401299130128  0.8581332138711365  0.1467887340028791   T   T   T
  0.0005690051886218  0.1129543455821596  0.1649752861463131   T   T   T
  0.0006948639382697  0.6127418112523175  0.1647103636008332   T   T   T
  0.2507642640601200  0.1129850383633222  0.1645807374993385   T   T   T
  0.2501412599645064  0.6123960210810202  0.1646023835308049   T   T   T
  0.5008399323407314  0.1129255338198184  0.1645229078377545   T   T   T
  0.5007916265489035  0.6128825806147206  0.1645176220830764   T   T   T
  0.7508179255731324  0.1129177765915950  0.1646848612681375   T   T   T
  0.7510896670616996  0.6126450265104592  0.1647388397657625   T   T   T
  0.1260521560184451  0.1837299483332871  0.2509263610783141   T   T   T
  0.1254107052655383  0.6841510296016471  0.2507027956788695   T   T   T
  0.3754871234185155  0.1841104528874667  0.2506991617775334   T   T   T
  0.3754155339706556  0.6841176932900712  0.2506368296630118   T   T   T
  0.6252257857810954  0.1842192829238410  0.2506933722577923   T   T   T
  0.6253428210464781  0.6841642983859249  0.2506608318866283   T   T   T
  0.8749149719236672  0.1839773245446645  0.2510134450356925   T   T   T
  0.8753810621505930  0.6845063615591548  0.2507791614273057   T   T   T
  0.0006320386568114  0.4383621199818614  0.2697177962699142   T   T   T
  0.0011475077209492  0.9377058018214256  0.2692112689877565   T   T   T
  0.2510102152555219  0.4380952060233723  0.2691715216229263   T   T   T
  0.2514007313380297  0.9375902633401401  0.2691014023587547   T   T   T
  0.5007804149588608  0.4380513666290934  0.2691550827576940   T   T   T
  0.5010468505065295  0.9376819468681653  0.2691258088599972   T   T   T
  0.7506507658903685  0.4379690515145799  0.2692819937602037   T   T   T
  0.7507112794183859  0.9376799502821330  0.2692429144793371   T   T   T
  0.9982898822356813  0.1413351736836778  0.3566595784841817   T   T   T
  0.0003854691152793  0.6426584438170320  0.3578226188331003   T   T   T
  0.2501132132275422  0.1409796071039172  0.3586156457636112   T   T   T
  0.2503398538837144  0.6431555576661777  0.3581808570599482   T   T   T
  0.5020687253016559  0.1408528780636862  0.3566824504157953   T   T   T
  0.5004043197463965  0.6426265833956198  0.3578787161980247   T   T   T
  0.7502090607299268  0.1423284988248836  0.3571333452433360   T   T   T
  0.7503684871354099  0.6424189480026544  0.3578160537615536   T   T   T
  0.2501628556943170  0.3152773308703079  0.4571989505490756   T   T   T
  0.1259199443480386  0.8871045287467891  0.3760665450473912   T   T   T
  0.3756735726855969  0.3866139992798872  0.3760024535631331   T   T   T
  0.3747072946387103  0.8870473940585618  0.3761139896008805   T   T   T
  0.6252175332899198  0.3866452623770046  0.3758848803021712   T   T   T
  0.6253418414279410  0.8858894261053839  0.3756132273525385   T   T   T
  0.8750167890214439  0.3867916014257976  0.3758638115526306   T   T   T
  0.8752200967570047  0.8862996539528698  0.3756005526119693   T   T   T
  0.1246160351355485  0.3866847853638984  0.3760262103980934   T   T   T
== Te interstitial in the bulk WTe2
    1.000000000000000
```

```
     13.8511690800000000    0.0000000000000000    0.0000000000000000
      0.0000000000000000   12.4846387199999995    0.0000000000000000
      0.0000000000000000    0.0000000000000000   14.0994563300000006
    W    Te
    32    65
Selective dynamics
Direct
  0.9972776454254592  0.0160388727225910  0.0234561482185121   T   T   T
  0.9993598493412356  0.5145520442340389  0.0228937473953825   T   T   T
  0.2471401085839683  0.0152177373330632  0.0296847931564950   T   T   T
  0.2508720617855845  0.5089618026486045  0.0339148604625607   T   T   T
  0.5050227691620001  0.0168467934230981  0.0247298511040975   T   T   T
  0.5003510446627119  0.5143034416262806  0.0241540002636647   T   T   T
  0.7508098096035963  0.0156000973742104  0.0184344451668800   T   T   T
  0.7504693536373165  0.5160385651029648  0.0193077352859895   T   T   T
  0.9976249594354851  0.3063324393144472  0.4925239856944824   T   T   T
  0.9947865493505612  0.8026465228588657  0.4930083987478426   T   T   T
  0.2491253205027151  0.2995195719903362  0.4916180304522590   T   T   T
  0.2483998132913336  0.8110365601305147  0.4837940256179297   T   T   T
  0.5034195498774520  0.3058144964024446  0.4903926557043476   T   T   T
  0.5058359702902077  0.8024336905973394  0.4915323034283878   T   T   T
  0.7500271056576459  0.3044313699062838  0.4948513509354748   T   T   T
  0.7498322069210106  0.8045420890072502  0.4953404374479392   T   T   T
  0.1256825206460949  0.4812830129131950  0.5050941986256052   T   T   T
  0.1040773239466979  0.9842578535722903  0.5002835091489441   T   T   T
  0.3736425265611958  0.4789012883487732  0.5018316563803645   T   T   T
  0.3956980995352068  0.9841037600780466  0.4987607172503752   T   T   T
  0.6267570765306705  0.4825802755936023  0.5083972854653653   T   T   T
  0.6282133852320477  0.9831214401902025  0.5089623995226205   T   T   T
  0.8738664323078377  0.4829675397887821  0.5096579928114850   T   T   T
  0.8721167627439581  0.9830596803511280  0.5096874428442615   T   T   T
  0.1232245997638464  0.1914942571304848  0.0109365116856985   T   T   T
  0.1239808293700583  0.6912978026470111  0.0110772304446174   T   T   T
  0.3763021746293145  0.1906183464839457  0.0111972213838505   T   T   T
  0.3735519342060397  0.6897712755226154  0.0128173016739472   T   T   T
  0.6277049503626133  0.1946101488377149  0.0054345401701111   T   T   T
  0.6260575548236561  0.6931883731805730  0.0046797772993499   T   T   T
  0.8736040393817807  0.1941804032716453  0.0051030170638865   T   T   T
  0.8743660427215378  0.6936529130092516  0.0042934412541872   T   T   T
  0.9972570647258048  0.3175992848561592  0.1174529803643958   T   T   T
  0.9974346980298050  0.8176891303525196  0.1156829060819646   T   T   T
  0.2497050168245441  0.3114716179912957  0.1238181894713709   T   T   T
  0.2486432719502157  0.8148889215391738  0.1227202048259571   T   T   T
  0.5025851872877544  0.3170055154536478  0.1177228651581733   T   T   T
  0.5027830596939267  0.8172987476661056  0.1158479737332620   T   T   T
  0.7507604075454088  0.3199099786768885  0.1148491208605721   T   T   T
  0.7504074787704437  0.8187914506635859  0.1132077450524137   T   T   T
  0.1209775902133009  0.0656141304125135  0.1670383407114902   T   T   T
  0.1214794554966651  0.5653527301364123  0.1670433582964328   T   T   T
  0.3776821775102684  0.0643922272488427  0.1661493909800657   T   T   T
  0.3783672606141649  0.5651405774391043  0.1687494384374434   T   T   T
  0.6292695810242097  0.0688378543840666  0.1618534541006778   T   T   T
  0.6270777750864821  0.5680049653413050  0.1611257201900820   T   T   T
  0.8722853449537307  0.0676176922606190  0.1612008855478851   T   T   T
```

```
  0.8736640522508199  0.5685091605563938  0.1608861382003115   T   T   T
  0.1240127574162298  0.3497498223322850  0.3517474186625266   T   T   T
  0.1185102099878285  0.8522563286113898  0.3483525224072039   T   T   T
  0.3749492347366855  0.3458874629034402  0.3503946637919369   T   T   T
  0.3801791571339931  0.8513682353028907  0.3481992288718896   T   T   T
  0.6282436748300555  0.3559848275353746  0.3515518168987493   T   T   T
  0.6281407841544459  0.8546198282441316  0.3519763890294213   T   T   T
  0.8731876424472005  0.3562964502903671  0.3530730412542662   T   T   T
  0.8723217727699784  0.8541855897388776  0.3528635182163557   T   T   T
  0.9920796319676768  0.1104264686935879  0.3923002001742887   T   T   T
  0.9983465087520959  0.6064038582733773  0.3994222210677426   T   T   T
  0.2493344635462905  0.0978307921879282  0.4013554149420704   T   T   T
  0.2484459749122628  0.6072155803701249  0.3939068148129414   T   T   T
  0.5078346683033959  0.1101179268754728  0.3909137975404511   T   T   T
  0.5025096117159917  0.6052774491732484  0.3974854811927717   T   T   T
  0.7501624317561093  0.1089154307364334  0.3986418619851680   T   T   T
  0.7505900159127684  0.6079164112324413  0.3999821245330312   T   T   T
  0.1183744844252855  0.1806354228123645  0.6023122270977721   T   T   T
  0.1266955733473866  0.6816292851124405  0.5972495434303958   T   T   T
  0.3821926762394925  0.1805469492340649  0.6002204868406491   T   T   T
  0.3739220861726676  0.6775894776239819  0.5879150756354377   T   T   T
  0.6251862615582277  0.1797513052494492  0.6054436969209105   T   T   T
  0.6263657217314994  0.6780548489187405  0.6067863525128251   T   T   T
  0.8741003390319866  0.1800174597176764  0.6061167586969436   T   T   T
  0.8738974658903850  0.6786160487589492  0.6074310827400229   T   T   T
  0.0018379704732088  0.4349929932697125  0.6482766072740201   T   T   T
  0.9967700690436789  0.9290202663546040  0.6505111270919591   T   T   T
  0.2507296380050005  0.4303819670896284  0.6450461287995100   T   T   T
  0.2508340110564575  0.9667749201978096  0.6229848144554372   T   T   T
  0.4976589119154566  0.4342326002300962  0.6457362588977859   T   T   T
  0.5025399552046808  0.9286864503451383  0.6490042970493416   T   T   T
  0.7497289716380569  0.4316171354070242  0.6505428945926095   T   T   T
  0.7496678874385359  0.9320908083300885  0.6513297921004734   T   T   T
  0.0009120893121031  0.1436884136427468  0.8672341499179167   T   T   T
  0.0016655477386657  0.6435840644755879  0.8670006802549798   T   T   T
  0.2498921024777606  0.1406499173829064  0.8724556696236477   T   T   T
  0.2479382045141037  0.6231554675241112  0.8764575634113548   T   T   T
  0.4996798140293623  0.1432155149437541  0.8682890728031709   T   T   T
  0.4966288085497753  0.6431473159350848  0.8685356614978531   T   T   T
  0.7502314144126275  0.1455115150081098  0.8631513791405936   T   T   T
  0.7499469143542701  0.6448291390346789  0.8632881469077872   T   T   T
  0.1261871558264304  0.3881056104785058  0.9162789131726291   T   T   T
  0.1265532049100454  0.8899783190269530  0.9168724296833998   T   T   T
  0.3738764176248346  0.3874321185947872  0.9171476571711804   T   T   T
  0.3730675946323910  0.8896663323210109  0.9262082373290855   T   T   T
  0.6239789144671114  0.3918032870690796  0.9091719256990058   T   T   T
  0.6248556725228873  0.8915056513802180  0.9087560887877071   T   T   T
  0.8759909102330916  0.3914197680428551  0.9083081375823068   T   T   T
  0.8765775081800679  0.8916237675830045  0.9078866504851105   T   T   T
  0.1260918737296621  0.7909029098891249  0.7540592430090809   T   T   T
== Te adatom bound to Pd1
   1.00000000000000
    13.8189816962433802    0.0000000000000000    0.0000000000000000
     0.0000000000000000   12.5126822235751405    0.0000000000000000
```

```
     0.0000000000000000    0.0000000000000000   34.0000000000000000
   W    Te   Pd
    32    65     1
Selective dynamics
Direct
  0.9998929761688800  0.0112328479828392  0.4211532338114381   F   F   F
  0.9984990335404154  0.5117591799069995  0.4217427857627710   T   T   T
  0.2519662694301890  0.0121895024729459  0.4230064356570873   T   T   T
  0.2492213377228007  0.5157013613775162  0.4222887407698086   T   T   T
  0.5000175134477298  0.0094879813513644  0.4216297260823522   T   T   T
  0.5004077616894793  0.5118100555446186  0.4221837294963577   T   T   T
  0.7515550358272741  0.0103879917447294  0.4205012774416019   T   T   T
  0.7507643515671955  0.5110533503495817  0.4215230394511325   T   T   T
  0.0001010279842077  0.3078678304799922  0.2058549390229558   F   F   F
  0.0010100750455821  0.8066932209726094  0.2060567652470098   T   T   T
  0.2508441664353848  0.3065730150737455  0.2060381722807090   T   T   T
  0.2508416366541382  0.8066781315201107  0.2060609182382862   T   T   T
  0.5006984433492079  0.3062945913686741  0.2060517087194725   T   T   T
  0.5007575650920785  0.8063830201217038  0.2061266372958931   T   T   T
  0.7509490073687325  0.3065232614281734  0.2060523988652826   T   T   T
  0.7510617950708681  0.8065243607119159  0.2060972997086902   T   T   T
  0.1260460906609911  0.4853550385860277  0.2120029675754012   T   T   T
  0.1258088123214285  0.9854337089745729  0.2119576590072955   T   T   T
  0.3760720958031219  0.4849578145765468  0.2120147486104963   T   T   T
  0.3760161364106988  0.9849876304773120  0.2120185402820266   T   T   T
  0.6258934489277658  0.4848084865459731  0.2121244228146406   T   T   T
  0.6259055130736371  0.9848342001924989  0.2121515460765180   T   T   T
  0.8758985076450724  0.4853444951001671  0.2120309324475262   T   T   T
  0.8761976599522228  0.9851613053559680  0.2120945410411820   T   T   T
  0.1196964551748011  0.1882325712769297  0.4159014787130348   T   T   T
  0.1230533700381339  0.6915798731931516  0.4155863810598651   T   T   T
  0.3797865663058970  0.1868766466967382  0.4180760620276512   T   T   T
  0.3769703505072320  0.6903508727630848  0.4162000478041141   T   T   T
  0.6287894173166433  0.1885436867207804  0.4157322527804196   T   T   T
  0.6257409490085079  0.6895296279686125  0.4153404018612304   T   T   T
  0.8745904680175108  0.1894914091528142  0.4150209067816930   T   T   T
  0.8750534365856398  0.6898836622763321  0.4154936870757139   T   T   T
  0.9965890130201375  0.3143790814804512  0.4619108657461103   T   T   T
  0.9989533790684845  0.8161905496370886  0.4617015741697652   T   T   T
  0.2123695759465977  0.3059133541009391  0.5408306947699445   T   T   T
  0.2499665946074036  0.8147213797439019  0.4619793943870514   T   T   T
  0.5097006540539789  0.3169894129186416  0.4631242978862661   T   T   T
  0.5020913732692831  0.8142487654364381  0.4619992954191983   T   T   T
  0.7519260217543927  0.3145695864471194  0.4619419564894700   T   T   T
  0.7508120427335908  0.8156576275356832  0.4612104905496251   T   T   T
  0.1237759943588846  0.0619775488478193  0.4802682302342082   T   T   T
  0.1233421261490366  0.5646542380047810  0.4799953941099644   T   T   T
  0.3785056684700169  0.0495449214267945  0.4820182954567398   T   T   T
  0.3752566806418347  0.5608856247739188  0.4803632508240347   T   T   T
  0.6273852224276015  0.0591272828714628  0.4793822916026962   T   T   T
  0.6263281089996806  0.5629360669731741  0.4799188355165132   T   T   T
  0.8747380167673753  0.0603992258122480  0.4792916304423490   T   T   T
  0.8743724423333475  0.5614924952674037  0.4800265697436696   T   T   T
  0.1258481491696010  0.3563388826825590  0.1478117369434091   T   T   T
```

```
  0.1258875618797019  0.8561917523658840  0.1478406619184710   T   T   T
  0.3757779862580803  0.3559886521837622  0.1478368216711184   T   T   T
  0.3758640352405144  0.8559648519111502  0.1478501258317136   T   T   T
  0.6257226834562745  0.3560180033645387  0.1479023372644134   T   T   T
  0.6257907154145594  0.8560528420365139  0.1479414987054059   T   T   T
  0.8753570947424825  0.3564708339843539  0.1476811111824744   T   T   T
  0.8758703313282920  0.8561769071528923  0.1478854006690233   T   T   T
  0.0009334791925250  0.1107970809444288  0.1657357265051609   T   T   T
  0.0011724911130140  0.6102575801639345  0.1657310614835702   T   T   T
  0.2511001947341330  0.1100849004786873  0.1656943387261977   T   T   T
  0.2511713209374079  0.6100941746621222  0.1657390454555507   T   T   T
  0.5010549982771646  0.1097879688869409  0.1657798180742222   T   T   T
  0.5010931444027336  0.6099251903364302  0.1658269857399584   T   T   T
  0.7510196395500010  0.1098440494933342  0.1658632610726759   T   T   T
  0.7509940441400268  0.6099608529971278  0.1658219915653398   T   T   T
  0.1255381815306352  0.1822191127774440  0.2516619846370017   T   T   T
  0.1259815579916615  0.6818179954036080  0.2518503455324900   T   T   T
  0.3756870016242005  0.1815798153876769  0.2517578129523295   T   T   T
  0.3758058815549347  0.6815016819945369  0.2517607693073636   T   T   T
  0.6258452000500714  0.1815636953334391  0.2517531312445840   T   T   T
  0.6259410389572824  0.6816407571294463  0.2517600971571463   T   T   T
  0.8761017129588703  0.1820321076601248  0.2517657197336322   T   T   T
  0.8760924738486674  0.6818421694186332  0.2518358925481668   T   T   T
  0.0009663376404187  0.4353886033787217  0.2703010559458117   T   T   T
  0.0011604407935078  0.9349537762927616  0.2703538396980611   T   T   T
  0.2509395254839190  0.4351685982238654  0.2703029511964133   T   T   T
  0.2507548462490353  0.9355493149932859  0.2702917691787273   T   T   T
  0.5008377982748877  0.4346050798612698  0.2703201976217252   T   T   T
  0.5007042976412345  0.9350641444637197  0.2704237662796536   T   T   T
  0.7510040707666470  0.4348428286722431  0.2703467975918975   T   T   T
  0.7511042650589584  0.9349297054095340  0.2703833911488988   T   T   T
  0.9996579450453451  0.1399529644791153  0.3564621604094679   T   T   T
  0.9998458798794778  0.6402098506314559  0.3571404879625586   T   T   T
  0.2522337544037424  0.1424066057130272  0.3594985856155544   T   T   T
  0.2504484147305374  0.6429553150733507  0.3578575843795534   T   T   T
  0.5001029333880759  0.1421294853640286  0.3582319380703787   T   T   T
  0.5000145496688443  0.6397445338408204  0.3573391257117530   T   T   T
  0.7502399510174720  0.1405535675258186  0.3563604885557428   T   T   T
  0.7503264764134057  0.6397237354614348  0.3571289829394219   T   T   T
  0.2408204878076975  0.3202553538300595  0.4580765432676291   T   T   T
  0.1263500331618837  0.8880197816161496  0.3758958990985118   T   T   T
  0.3751953898240811  0.3846792096158052  0.3781670279055360   T   T   T
  0.3749282623433766  0.8865140813466689  0.3760718844430070   T   T   T
  0.6254411413562836  0.3860199134959620  0.3761379272112418   T   T   T
  0.6250368725450808  0.8860018141178041  0.3751521364001206   T   T   T
  0.8748954349062601  0.3866123648648607  0.3754381419269359   T   T   T
  0.8762863291581914  0.8857455081945197  0.3748167118067189   T   T   T
  0.1242068943197538  0.3874669636459183  0.3762893326889881   T   T   T
  0.3675883183409392  0.2499529707421353  0.5059433305203993   T   T   T
== Te adatom bound to Pd2
   1.00000000000000
    13.8189816962433802    0.0000000000000000    0.0000000000000000
     0.0000000000000000   12.5126822235751405    0.0000000000000000
     0.0000000000000000    0.0000000000000000   34.0000000000000000
```

```
   W    Te   Pd
    32    65     2
Selective dynamics
Direct
  0.9998929761688800  0.0112328479828392  0.4211532338114381   F   F   F
  0.9984785314827176  0.5137892529945488  0.4217265027241958   T   T   T
  0.2499181596129289  0.0131884539728606  0.4231259813939531   T   T   T
  0.2498391755387121  0.5187333586284738  0.4219710124999382   T   T   T
  0.4999662812350513  0.0110371004120026  0.4213477372649245   T   T   T
  0.5012853986803093  0.5136295042854033  0.4218349050475938   T   T   T
  0.7497718811813828  0.0116087346034059  0.4202483840093887   T   T   T
  0.7498634520380184  0.5127795741032387  0.4213544350932392   T   T   T
  0.0001010279842077  0.3078678304799922  0.2058549390229558   F   F   F
  0.0007567777558978  0.8069620671814308  0.2059361786070563   T   T   T
  0.2501744873443832  0.3071999918361557  0.2056596193508792   T   T   T
  0.2501757043758256  0.8073027630636187  0.2056844783093739   T   T   T
  0.4996196879538718  0.3069018994352300  0.2056785031139924   T   T   T
  0.4996699883084711  0.8070335308720160  0.2057293007084721   T   T   T
  0.7503157908993672  0.3067492787168859  0.2058460062732681   T   T   T
  0.7504201866600347  0.8068979756096080  0.2058929594076779   T   T   T
  0.1252185569324656  0.4857510534240687  0.2117544295392914   T   T   T
  0.1250170628467440  0.9858801595418067  0.2115991003779987   T   T   T
  0.3751088010883434  0.4856904020095398  0.2115661965918045   T   T   T
  0.3751360340436892  0.9857297060916279  0.2115334333697765   T   T   T
  0.6248450678646553  0.4852915327151139  0.2118106643953488   T   T   T
  0.6248657945595052  0.9852964630346075  0.2118229149095623   T   T   T
  0.8755464345308953  0.4855377882756419  0.2119520941911632   T   T   T
  0.8758640762378149  0.9853719310238026  0.2119164740808855   T   T   T
  0.1188558794977609  0.1884754362166218  0.4175822644242086   T   T   T
  0.1219495257446511  0.6926721381982305  0.4157399996122035   T   T   T
  0.3808552899479489  0.1882125245242780  0.4177431835522131   T   T   T
  0.3777668298137350  0.6925280346744969  0.4157489392199287   T   T   T
  0.6271596177498627  0.1899643868492036  0.4153522645255210   T   T   T
  0.6252923492499550  0.6910674841736858  0.4150550617342452   T   T   T
  0.8725330479321262  0.1900232703224675  0.4152365707811980   T   T   T
  0.8744230594514970  0.6911692912872992  0.4150618487009248   T   T   T
  0.9905930376026972  0.3189480217659812  0.4630781412872242   T   T   T
  0.9978548674718442  0.8163834994816079  0.4617129316338968   T   T   T
  0.2498092803245223  0.3107106770134132  0.5618946484376879   T   T   T
  0.2498279552947480  0.8151717187114412  0.4618085352704219   T   T   T
  0.5091876692758951  0.3187903199172520  0.4632877879302739   T   T   T
  0.5019210456721306  0.8159940580033113  0.4617920117593545   T   T   T
  0.7499440830862532  0.3154643929166307  0.4618629288994114   T   T   T
  0.7498808580756924  0.8171573847234683  0.4607937443016784   T   T   T
  0.1220372614735406  0.0528883825542546  0.4814907697436491   T   T   T
  0.1236954684643491  0.5642191798630325  0.4799732826112320   T   T   T
  0.3775316074679492  0.0524688932560776  0.4816484869259579   T   T   T
  0.3760545179171237  0.5641612521761498  0.4800072255346414   T   T   T
  0.6263574295476413  0.0607427764325239  0.4792299365913013   T   T   T
  0.6260913101329918  0.5644452076879970  0.4798631395920332   T   T   T
  0.8734300670227505  0.0607981160435581  0.4791002205997318   T   T   T
  0.8737852517212847  0.5644692618169153  0.4798416587903304   T   T   T
  0.1249701476971366  0.3566742175612546  0.1475596591168894   T   T   T
  0.1251531035353555  0.8565154538227217  0.1475259386336380   T   T   T
```

```
  0.3750250774018590  0.3566584180564540  0.1473881648918156   T   T   T
  0.3751026564012793  0.8565786421542571  0.1474033030890227   T   T   T
  0.6252778424015807  0.3562642081277170  0.1476713431307478   T   T   T
  0.6253371467139895  0.8561965144515534  0.1476862892013096   T   T   T
  0.8751048171771393  0.3566983741525515  0.1476421380072589   T   T   T
  0.8755218776577323  0.8562850865579300  0.1477701603803196   T   T   T
  0.0000140851961462  0.1110978931543859  0.1653615665298933   T   T   T
  0.0002602356680408  0.6107678215089384  0.1655136111190953   T   T   T
  0.2500332138423285  0.1108787795824286  0.1652346206722945   T   T   T
  0.2500444035998718  0.6108804201411422  0.1652868973984578   T   T   T
  0.5002827637189255  0.1105732727021775  0.1652906115338529   T   T   T
  0.5002982703734966  0.6106288676140760  0.1653181680110641   T   T   T
  0.7504924128514292  0.1100300298575111  0.1655828212838568   T   T   T
  0.7504613340785772  0.6102535526050936  0.1656404819848242   T   T   T
  0.1252672079302437  0.1822414269144358  0.2514499112183355   T   T   T
  0.1255991051552829  0.6822014339380451  0.2516563991315488   T   T   T
  0.3749023810073362  0.1821360842935624  0.2514217690304911   T   T   T
  0.3749433488746973  0.6821224000298943  0.2514290197207433   T   T   T
  0.6248665888226798  0.1821034751852954  0.2514098741912288   T   T   T
  0.6249301674539521  0.6821464285764788  0.2514150452508175   T   T   T
  0.8755070764729826  0.1822399307209627  0.2515557820657415   T   T   T
  0.8754272723197920  0.6822734762486645  0.2516434281941433   T   T   T
  0.0005827937282852  0.4356481666024680  0.2702419326697774   T   T   T
  0.0007933306927871  0.9355563349825589  0.2702078062021990   T   T   T
  0.2503673494980163  0.4355092473553633  0.2699818304344399   T   T   T
  0.2502570479542565  0.9361285890902526  0.2699171492781427   T   T   T
  0.4997229403047646  0.4351654218441091  0.2699960544038861   T   T   T
  0.4996179199218281  0.9354604076500422  0.2700573531371148   T   T   T
  0.7500348640438108  0.4350510802912929  0.2700609796953229   T   T   T
  0.7502453894927114  0.9354074727872748  0.2701011395346455   T   T   T
  0.0000064940919011  0.1429225637299986  0.3573932982402374   T   T   T
  0.9996276157187384  0.6422498882017874  0.3568952212338535   T   T   T
  0.2499583964821107  0.1443570562792350  0.3601477871552355   T   T   T
  0.2497937370922112  0.6449359443978376  0.3577289801785319   T   T   T
  0.4997099983890130  0.1427436773548945  0.3575564161539890   T   T   T
  0.5000906305390513  0.6418444142951722  0.3568976656389408   T   T   T
  0.7497647533876483  0.1414277064582097  0.3561669854975251   T   T   T
  0.7499019343720941  0.6415235583222367  0.3567788144797987   T   T   T
  0.2497925376355651  0.3223059615404152  0.4554001998932297   T   T   T
  0.1257203476680817  0.8885100326756884  0.3757484038540525   T   T   T
  0.3761239059136711  0.3877092602488471  0.3774101354311911   T   T   T
  0.3741539767937318  0.8883370340632443  0.3757897945648189   T   T   T
  0.6250991138835609  0.3873349997142725  0.3758704545998311   T   T   T
  0.6242171679542383  0.8874700087769769  0.3745993474000588   T   T   T
  0.8744809076853266  0.3875150896040417  0.3757812373759470   T   T   T
  0.8753411846301239  0.8874117544801061  0.3745626807182095   T   T   T
  0.1235729702699613  0.3879277533542456  0.3772475742791450   T   T   T
  0.1346082612125000  0.2538054114162209  0.5075154824255103   T   T   T
  0.3648003040523544  0.2535233183814849  0.5074911967749928   T   T   T
== Te-in vacancy on WTe2 (001)
   1.00000000000000
     14.0906791687999995    0.0000000000000000    0.0000000000000000
      0.0000000000000000   25.2576961516000011    0.0000000000000000
      0.0000000000000000    0.0000000000000000   27.7237830675911070
```

```
   W    Te
    64   127
Selective dynamics
Direct
  0.3748008104701092  0.8516438280321132  0.3738530604647529   T   T   T
  0.6249973897505229  0.8516233099362089  0.3738485305544358   T   T   T
  0.1248990853416167  0.8516023206706635  0.3738166204945806   T   T   T
  0.3748973959550350  0.3512947279355748  0.3714341869407626   T   T   T
  0.8749581722741718  0.1016058032211903  0.3720036238176209   T   T   T
  0.6250052905138344  0.1016099819295317  0.3720175866959672   T   T   T
  0.1248587020864959  0.6011975448574541  0.3730035446832181   T   T   T
  0.8749343095719737  0.3512742395769729  0.3713514982576753   T   T   T
  0.1249829962271234  0.1015995247477390  0.3719939239137651   T   T   T
  0.6249672834425836  0.3512777377705388  0.3714064059967338   T   T   T
  0.8751624912940060  0.6013323782464464  0.3733209330017858   T   T   T
  0.3747401410668098  0.6013046043198707  0.3735283612654447   T   T   T
  0.3750153794766613  0.1015955761810637  0.3720276046678818   T   T   T
  0.8750482328584379  0.8517158399060168  0.3737968427326202   T   T   T
  0.6249945791995982  0.6013915359083872  0.3738114101883623   T   T   T
  0.1249415971171370  0.3512734856103465  0.3713284706837068   T   T   T
  0.7499806312853150  0.0128734179889507  0.3805106716476951   T   T   T
  0.4998962886232101  0.2625525649822160  0.3792234375514058   T   T   T
  0.2499986739853264  0.2625514466342275  0.3792138814102152   T   T   T
  0.2499937158326770  0.7626520712689387  0.3815313899560039   T   T   T
  0.7500476446478460  0.2625578505668176  0.3791950890702238   T   T   T
  0.9997561510510354  0.5124021557940341  0.3804008161241203   T   T   T
  0.4998138022712239  0.5124733151679186  0.3808623388729045   T   T   T
  0.2499826538926067  0.0128314559059914  0.3805632258608476   T   T   T
  0.7500116126860078  0.5124915932410000  0.3807844163770877   T   T   T
  0.9997803210838242  0.7627209960332683  0.3814332029912698   T   T   T
  0.4999424273858847  0.0128464136866044  0.3805471163416803   T   T   T
  0.4997903896990472  0.7627788763538214  0.3816787432434770   T   T   T
  0.7502027275398561  0.7628190865093279  0.3815986861372392   T   T   T
  0.2500404907926412  0.5123982313988539  0.3805028734164542   T   T   T
  0.9999762567160714  0.2625480073309255  0.3791890507677736   T   T   T
  0.9999340164693100  0.0128572711512098  0.3805252666183709   T   T   T
  0.5006215670424397  0.8978801274954469  0.6212899429369930   T   T   T
  0.2484727730424617  0.6481877832778603  0.6179140264803333   T   T   T
  0.0015504703461313  0.6482550873363356  0.6181061850738616   T   T   T
  0.4895904800423664  0.6475241606774008  0.6156397389486827   T   T   T
  0.2501362378617807  0.8988613650851208  0.6213014340559081   T   T   T
  0.7492844217145993  0.3988013746627517  0.6192978304859682   T   T   T
  0.5007151191792002  0.3987901886596869  0.6192701302400371   T   T   T
  0.5002100461707119  0.1483254502486680  0.6222782353749070   T   T   T
  0.7495656294187102  0.8980101430743653  0.6213554137367457   T   T   T
  0.9997243271659921  0.1484083536725371  0.6223150861122133   T   T   T
  0.9996770928723324  0.3978103167719829  0.6193029252517722   T   T   T
  0.7498081056965977  0.1483413649284540  0.6222905432302432   T   T   T
  0.2504808859463417  0.3977786315420943  0.6192085606001135   T   T   T
  0.9999290719861083  0.8989588809863794  0.6214168957495582   T   T   T
  0.2503110037619225  0.1483314130062819  0.6222642423924323   T   T   T
  0.7606362393606521  0.6475427407108959  0.6158453942014539   T   T   T
  0.1250911267128927  0.2370934239368807  0.6291946228371851   T   T   T
  0.1249059218613528  0.7374910258592602  0.6258665402172909   T   T   T
```

0.8748828769272067	0.4863540414614492	0.6254194172647765	T	T	T
0.3723720380142171	0.7374250815966056	0.6253804657478045	T	T	T
0.1250420185336942	0.9875631176457753	0.6298495001314246	T	T	T
0.1250846217333667	0.4865379364933190	0.6260894318419413	T	T	T
0.8745803667464889	0.2373256160506164	0.6291855397519910	T	T	T
0.8743878679251292	0.9872171518841740	0.6299076435635212	T	T	T
0.3756625573463858	0.9871074915983952	0.6298292601535427	T	T	T
0.3752467148666323	0.4863893521349517	0.6252523632465190	T	T	T
0.6250530770501775	0.9868729176383124	0.6298304676829328	T	T	T
0.6250156135934726	0.2374547926135079	0.6292120650147097	T	T	T
0.6253869150317839	0.7336769341721161	0.6257811842300280	T	T	T
0.8775628930947961	0.7375422687399659	0.6256172714699991	T	T	T
0.6250367222519434	0.4897578093119151	0.6224749677583155	T	T	T
0.3754420088998247	0.2372792706388658	0.6291899714157829	T	T	T
0.9999705122599784	0.0764342865203687	0.3016563655470592	T	T	T
0.9999122927528489	0.3269305675043896	0.3007349698381110	T	T	T
0.9997478111242778	0.5772405524392986	0.3024221125711438	T	T	T
0.9999529720431390	0.8271693199101685	0.3032404906602262	T	T	T
0.2500409435199989	0.0763509160797874	0.3016947242338303	T	T	T
0.2500015613119555	0.3269394751213898	0.3007767036872790	T	T	T
0.2502288286530619	0.5772501908657970	0.3025570023714816	T	T	T
0.2499286210824342	0.8270372882033611	0.3032964780347186	T	T	T
0.5000021158144353	0.0764010622734875	0.3017028230713449	T	T	T
0.4998632907524983	0.3269656097903457	0.3008508646378351	T	T	T
0.5001162982634079	0.5773601356502351	0.3030234300184310	T	T	T
0.4999241848498459	0.8270294655474143	0.3033331864730571	T	T	T
0.7499558196935469	0.0764357125944141	0.3016493882818367	T	T	T
0.7498540792685763	0.3269702164428648	0.3007712722042931	T	T	T
0.7496879472290463	0.5773273576700669	0.3029113335232727	T	T	T
0.7499720352746960	0.8271181046962695	0.3032920298438739	T	T	T
0.6249766458624062	0.2003216675939180	0.3233466619433940	T	T	T
0.3749653097549481	0.2002769077100476	0.3233198500764404	T	T	T
0.6249459738656914	0.4510950776061148	0.3242164591190109	T	T	T
0.1249473436300007	0.7009030040508978	0.3252843457485056	T	T	T
0.6249474894078095	0.7008256290077608	0.3257499526029729	T	T	T
0.3751355750375078	0.4510097813703977	0.3240829336330455	T	T	T
0.6249903040230407	0.9504718980630079	0.3249847729167173	T	T	T
0.1249754789799599	0.4510063445524218	0.3239146191233530	T	T	T
0.8750005298902541	0.2002801828624114	0.3233142933581139	T	T	T
0.3750154181975447	0.7007621544726638	0.3255684500409275	T	T	T
0.8747102602649982	0.4510180941420638	0.3239905056527994	T	T	T
0.1249975300472628	0.9505032613829322	0.3249795906445480	T	T	T
0.8748605343887907	0.7008701568602066	0.3253630758417825	T	T	T
0.3749867289034629	0.9504803395590804	0.3249843260399008	T	T	T
0.8749800771500945	0.9505481177656466	0.3248878743872863	T	T	T
0.1250059331805562	0.2002752188726266	0.3233135338652478	T	T	T
0.7500045519160865	0.1637296616475099	0.4266598382046226	T	T	T
0.2495685425845882	0.6631564408969449	0.4274569497108657	T	T	T
0.9999880400997282	0.9140321958912531	0.4280909060141160	T	T	T
0.4997492243512480	0.6633601363652862	0.4279169776508503	T	T	T
0.4999843221125759	0.1637247451065177	0.4266531224886276	T	T	T
0.7499922678117078	0.4127481906888301	0.4264646350959649	T	T	T
0.2497957690377758	0.4127958232657147	0.4264269233673941	T	T	T
0.2499959631660277	0.1637340277359879	0.4266480159615757	T	T	T

0.9999452316966192	0.4127858597486815	0.4263846204277927	T	T	T
0.4999932822577478	0.9139610976040879	0.4280364698474961	T	T	T
0.7502015832987625	0.6633870823674011	0.4277706961373557	T	T	T
0.2498887494791807	0.9139994477946852	0.4281044965631706	T	T	T
0.4999746594071807	0.4127458138212473	0.4264741173500164	T	T	T
0.0001374430530533	0.6632599629642398	0.4272811725380966	T	T	T
-0.0000094312231353	0.1637382558661351	0.4266517558277191	T	T	T
0.7500082337729144	0.9140304690779070	0.4280345376207890	T	T	T
0.3748250592237010	0.7875619434841020	0.4522708044461650	T	T	T
0.3749486994862530	0.0385205318280976	0.4509739252375849	T	T	T
0.3746798754335807	0.5367311052786592	0.4515852657060364	T	T	T
0.8750254834006097	0.2874546459255036	0.4498403811242823	T	T	T
0.6249956043616417	0.2874285541283254	0.4498366400002448	T	T	T
0.6250252834655375	0.7873609930753316	0.4522448166737104	T	T	T
0.3749695138772269	0.2874354101164688	0.4498732581590196	T	T	T
0.1249751965425437	0.2874651061391567	0.4498234707833941	T	T	T
0.1249367415322014	0.0386307022539232	0.4509697329421447	T	T	T
0.8751936484296277	0.5368733081041653	0.4514423021119872	T	T	T
0.6249585297944629	0.0385205253922792	0.4509633819162990	T	T	T
0.1248865807265798	0.7874225275025153	0.4521204827607790	T	T	T
0.8749344104922132	0.0385301036584466	0.4509549817864300	T	T	T
0.6249855348739648	0.5366670429656417	0.4517662364362017	T	T	T
0.1247897237211310	0.5367221039719250	0.4511333023933257	T	T	T
0.8750904016243847	0.7876042855112396	0.4522280192131866	T	T	T
0.6250082360574843	0.1724309699229776	0.5513702757180298	T	T	T
0.3750405015603187	0.4205255536568632	0.5479466914374905	T	T	T
0.3756741181158263	0.9234711630867471	0.5509711757323202	T	T	T
0.3705292352440487	0.6749543164451887	0.5454601881470597	T	T	T
0.6252938173047964	0.6705264300896771	0.5481198132659011	T	T	T
0.1249896870987763	0.9243182883661782	0.5509209691812710	T	T	T
0.8796210951834201	0.6750530115384732	0.5456351172229914	T	T	T
0.8748333749116713	0.1722418966599652	0.5513412852682351	T	T	T
0.1250056993689837	0.1721643529571522	0.5513629707804287	T	T	T
0.1249948233369636	0.4212341472636282	0.5483310889411478	T	T	T
0.6250269020643477	0.4208082499923373	0.5476501782196402	T	T	T
0.3751922925487223	0.1722365515786244	0.5513368307031076	T	T	T
0.1249360448638748	0.6735993583027774	0.5471701001574064	T	T	T
0.8745402481936168	0.9235948190129679	0.5510509013454755	T	T	T
0.6251362034853529	0.9233326107285398	0.5508007462128497	T	T	T
0.8750115785549004	0.4205393628779093	0.5479752750088184	T	T	T
0.7496796021902300	0.0493474854525804	0.5752698251031404	T	T	T
0.2490528343494464	0.5490080373204602	0.5714282584387532	T	T	T
0.7527621587299448	0.5493714597274256	0.5699199987521638	T	T	T
0.5003943505591258	0.0493133055006950	0.5752623572001053	T	T	T
0.2490465426391664	0.8012559709851880	0.5722580691895754	T	T	T
0.9999496665303563	0.2980741274078996	0.5734874968159087	T	T	T
0.5004109409588389	0.7994126368025354	0.5729576678179047	T	T	T
0.2504730366225070	0.0494410459344810	0.5753529285715672	T	T	T
0.0007588552275838	0.8013747582851103	0.5724073610870163	T	T	T
0.7498625724511507	0.2986598631526039	0.5735467432741983	T	T	T
0.7499841589041782	0.7996213461604320	0.5730119508118071	T	T	T
0.9996011272994264	0.0495109752592560	0.5753695026214853	T	T	T
0.4973717492082174	0.5493444010508778	0.5698123642872039	T	T	T
0.2501283592149081	0.2980428396665324	0.5734613103003091	T	T	T

```
  0.0007393141934242  0.5491019966262493  0.5717211194787959   T   T   T
  0.5001678315509992  0.2986317945670022  0.5735631345916982   T   T   T
  0.3752274896408363  0.0862169612534649  0.6783476950126238   T   T   T
  0.3761768135802305  0.3372290556748040  0.6759550992824821   T   T   T
  0.1250091165895206  0.0864342986497910  0.6782695403224015   T   T   T
  0.8721837081241957  0.5859983745209077  0.6742785274053273   T   T   T
  0.3747988942535745  0.8349523400043102  0.6759575694102755   T   T   T
  0.1250364934336061  0.8356293291655176  0.6760322693323599   T   T   T
  0.1251619087605726  0.3367820033427175  0.6758493074400748   T   T   T
  0.8739082604391289  0.3372906734406891  0.6759646110565124   T   T   T
  0.6250235422565651  0.0861735662514577  0.6783673084684331   T   T   T
  0.1252026143819867  0.5863617208316275  0.6743655598668813   T   T   T
  0.8748596956896048  0.0863098179302597  0.6784196815325703   T   T   T
  0.6250024362547871  0.3377356894408661  0.6760664498688989   T   T   T
  0.6251266006176014  0.8333035834381359  0.6754490069896208   T   T   T
  0.3779875695428126  0.5859467515623650  0.6740115783570971   T   T   T
  0.8752401555410918  0.8352949212885974  0.6762329378772360   T   T   T
  0.2509509472441674  0.4636500184308897  0.6965298999403211   T   T   T
  0.0007339199599514  0.7125780863914046  0.6966400815506192   T   T   T
  0.7515956999295638  0.7098850794249013  0.6955344506751501   T   T   T
  0.5032074932487848  0.4680951094803860  0.6953654294063908   T   T   T
  0.9993963063762041  0.4637830581755327  0.6966641558663386   T   T   T
  0.7467922277329998  0.4680602815681991  0.6954205202553823   T   T   T
  0.4985647298702681  0.7098965366151264  0.6952520321794216   T   T   T
  0.7497999279667853  0.2134795120097748  0.7000429532452881   T   T   T
  0.5003056770657435  0.9613954087903288  0.7001672245996485   T   T   T
  0.9998563200770290  0.2137888021253924  0.6999952362016072   T   T   T
  0.2502598587894782  0.9623968639482319  0.7002094413307548   T   T   T
  0.2502983431843531  0.2134616798117489  0.6999989699125783   T   T   T
  0.9999045003431823  0.9626193115465346  0.7002700664092586   T   T   T
  0.2493344243923984  0.7123931204512109  0.6964724885824009   T   T   T
  0.7497425857406033  0.9614954065180861  0.7002594137277534   T   T   T
  0.5002410970983319  0.2134624580589666  0.7000352810266374   T   T   T
== Te-in vacancy containing Pd1
   1.00000000000000
    14.0906791687999995    0.0000000000000000    0.0000000000000000
     0.0000000000000000   25.2576961516000011    0.0000000000000000
     0.0000000000000000    0.0000000000000000   27.7237830675911070
   W    Te   Pd
    64   127     1
Selective dynamics
Direct
  0.3748008104701092  0.8516438280321132  0.3738530604647529   T   T   T
  0.6249973897505229  0.8516233099362089  0.3738485305544358   T   T   T
  0.1248990853416167  0.8516023206706635  0.3738166204945806   T   T   T
  0.3748973959550350  0.3512947279355748  0.3714341869407626   T   T   T
  0.8749581722741718  0.1016058032211903  0.3720036238176209   T   T   T
  0.6250052905138344  0.1016099819295317  0.3720175866959672   T   T   T
  0.1248587020864959  0.6011975448574541  0.3730035446832181   T   T   T
  0.8749343095719737  0.3512742395769729  0.3713514982576753   T   T   T
  0.1249829962271234  0.1015995247477390  0.3719939239137651   T   T   T
  0.6249672834425836  0.3512777377705388  0.3714064059967338   T   T   T
  0.8751624912940060  0.6013323782464464  0.3733209330017858   T   T   T
  0.3747401410668098  0.6013046043198707  0.3735283612654447   T   T   T
```

0.3750153794766613	0.1015955761810637	0.3720276046678818	T	T	T
0.8750482328584379	0.8517158399060168	0.3737968427326202	T	T	T
0.6249945791995982	0.6013915359083872	0.3738114101883623	T	T	T
0.1249415971171370	0.3512734856103465	0.3713284706837068	T	T	T
0.7499806312853150	0.0128734179889507	0.3805106716476951	T	T	T
0.4998962886232101	0.2625525649822160	0.3792234375514058	T	T	T
0.2499986739853264	0.2625514466342275	0.3792138814102152	T	T	T
0.2499937158326770	0.7626520712689387	0.3815313899560039	T	T	T
0.7500476446478460	0.2625578505668176	0.3791950890702238	T	T	T
0.9997561510510354	0.5124021557940341	0.3804008161241203	T	T	T
0.4998138022712239	0.5124733151679186	0.3808623388729045	T	T	T
0.2499826538926067	0.0128314559059914	0.3805632258608476	T	T	T
0.7500116126860078	0.5124915932410000	0.3807844163770877	T	T	T
0.9997803210838242	0.7627209960332683	0.3814332029912698	T	T	T
0.4999424273858847	0.0128464136866044	0.3805471163416803	T	T	T
0.4997903896990472	0.7627788763538214	0.3816787432434770	T	T	T
0.7502027275398561	0.7628190865093279	0.3815986861372392	T	T	T
0.2500404907926412	0.5123982313988539	0.3805028734164542	T	T	T
0.9999762567160714	0.2625480073309255	0.3791890507677736	T	T	T
0.9999340164693100	0.0128572711512098	0.3805252666183709	T	T	T
0.5006215670424397	0.8978801274954469	0.6212899429369930	T	T	T
0.2484727730424617	0.6481877832778603	0.6179140264803333	T	T	T
0.0015504703461313	0.6482550873363356	0.6181061850738616	T	T	T
0.4895904800423664	0.6475241606774008	0.6156397389486827	T	T	T
0.2501362378617807	0.8988613650851208	0.6213014340559081	T	T	T
0.7492844217145993	0.3988013746627517	0.6192978304859682	T	T	T
0.5007151191792002	0.3987901886596869	0.6192701302400371	T	T	T
0.5002100461707119	0.1483254502486680	0.6222782353749070	T	T	T
0.7495656294187102	0.8980101430743653	0.6213554137367457	T	T	T
0.9997243271659921	0.1484083536725371	0.6223150861122133	T	T	T
0.9996770928723324	0.3978103167719829	0.6193029252517722	T	T	T
0.7498081056965977	0.1483413649284540	0.6222905432302432	T	T	T
0.2504808859463417	0.3977786315420943	0.6192085606001135	T	T	T
0.9999290719861083	0.8989588809863794	0.6214168957495582	T	T	T
0.2503110037619225	0.1483314130062819	0.6222642423924323	T	T	T
0.7606362393606521	0.6475427407108959	0.6158453942014539	T	T	T
0.1250911267128927	0.2370934239368807	0.6291946228371851	T	T	T
0.1249059218613528	0.7374910258592602	0.6258665402172909	T	T	T
0.8748828769272067	0.4863540414614492	0.6254194172647765	T	T	T
0.3723720380142171	0.7374250815966056	0.6253804657478045	T	T	T
0.1250420185336942	0.9875631176457753	0.6298495001314246	T	T	T
0.1250846217333667	0.4865379364933190	0.6260894318419413	T	T	T
0.8745803667464889	0.2373256160506164	0.6291855397519910	T	T	T
0.8743878679251292	0.9872171518841740	0.6299076435635212	T	T	T
0.3756625573463858	0.9871074915983952	0.6298292601535427	T	T	T
0.3752467148666323	0.4863893521349517	0.6252523632465190	T	T	T
0.6250530770501775	0.9868729176383124	0.6298304676829328	T	T	T
0.6250156135934726	0.2374547926135079	0.6292120650147097	T	T	T
0.6253869150317839	0.7336769341721161	0.6257811842300280	T	T	T
0.8775628930947961	0.7375422687399659	0.6256172714699991	T	T	T
0.6250367222519434	0.4897578093119151	0.6224749677583155	T	T	T
0.3754420088998247	0.2372792706388658	0.6291899714157829	T	T	T
0.9999705122599784	0.0764342865203687	0.3016563655470592	T	T	T
0.9999122927528489	0.3269305675043896	0.3007349698381110	T	T	T

0.9997478111242778	0.5772405524392986	0.3024221125711438	T	T	T
0.9999529720431390	0.8271693199101685	0.3032404906602262	T	T	T
0.2500409435199989	0.0763509160797874	0.3016947242338303	T	T	T
0.2500015613119555	0.3269394751213898	0.3007767036872790	T	T	T
0.2502288286530619	0.5772501908657970	0.3025570023714816	T	T	T
0.2499286210824342	0.8270372882033611	0.3032964780347186	T	T	T
0.5000021158144353	0.0764010622734875	0.3017028230713449	T	T	T
0.4998632907524983	0.3269656097903457	0.3008508646378351	T	T	T
0.5001162982634079	0.5773601356502351	0.3030234300184310	T	T	T
0.4999241848498459	0.8270294655474143	0.3033331864730571	T	T	T
0.7499558196935469	0.0764357125944141	0.3016493882818367	T	T	T
0.7498540792685763	0.3269702164428648	0.3007712722042931	T	T	T
0.7496879472290463	0.5773273576700669	0.3029113335232727	T	T	T
0.7499720352746960	0.8271181046962695	0.3032920298438739	T	T	T
0.6249766458624062	0.2003216675939180	0.3233466619433940	T	T	T
0.3749653097549481	0.2002769077100476	0.3233198500764404	T	T	T
0.6249459738656914	0.4510950776061148	0.3242164591190109	T	T	T
0.1249473436300007	0.7009030040508978	0.3252843457485056	T	T	T
0.6249474894078095	0.7008256290077608	0.3257499526029729	T	T	T
0.3751355750375078	0.4510097813703977	0.3240829336330455	T	T	T
0.6249903040230407	0.9504718980630079	0.3249847729167173	T	T	T
0.1249754789799599	0.4510063445524218	0.3239146191233530	T	T	T
0.8750005298902541	0.2002801828624114	0.3233142933581139	T	T	T
0.3750154181975447	0.7007621544726638	0.3255684500409275	T	T	T
0.8747102602649982	0.4510180941420638	0.3239905056527994	T	T	T
0.1249975300472628	0.9505032613829322	0.3249795906445480	T	T	T
0.8748605343887907	0.7008701568602066	0.3253630758417825	T	T	T
0.3749867289034629	0.9504803395590804	0.3249843260399008	T	T	T
0.8749800771500945	0.9505481177656466	0.3248878743872863	T	T	T
0.1250059331805562	0.2002752188726266	0.3233135338652478	T	T	T
0.7500045519160865	0.1637296616475099	0.4266598382046226	T	T	T
0.2495685425845882	0.6631564408969449	0.4274569497108657	T	T	T
0.9999880400997282	0.9140321958912531	0.4280909060141160	T	T	T
0.4997492243512480	0.6633601363652862	0.4279169776508503	T	T	T
0.4999843221125759	0.1637247451065177	0.4266531224886276	T	T	T
0.7499922678117078	0.4127481906888301	0.4264646350959649	T	T	T
0.2497957690377758	0.4127958232657147	0.4264269233673941	T	T	T
0.2499959631660277	0.1637340277359879	0.4266480159615757	T	T	T
0.9999452316966192	0.4127858597486815	0.4263846204277927	T	T	T
0.4999932822577478	0.9139610976040879	0.4280364698474961	T	T	T
0.7502015832987625	0.6633870823674011	0.4277706961373557	T	T	T
0.2498887494791807	0.9139994477946852	0.4281044965631706	T	T	T
0.4999746594071807	0.4127458138212473	0.4264741173500164	T	T	T
0.0001374430530533	0.6632599629642398	0.4272811725380966	T	T	T
-0.0000094312231353	0.1637382558661351	0.4266517558277191	T	T	T
0.7500082337729144	0.9140304690779070	0.4280345376207890	T	T	T
0.3748250592237010	0.7875619434841020	0.4522708044461650	T	T	T
0.3749486994862530	0.0385205318280976	0.4509739252375849	T	T	T
0.3746798754335807	0.5367311052786592	0.4515852657060364	T	T	T
0.8750254834006097	0.2874546459255036	0.4498403811242823	T	T	T
0.6249956043616417	0.2874285541283254	0.4498366400002448	T	T	T
0.6250252834655375	0.7873609930753316	0.4522448166737104	T	T	T
0.3749695138772269	0.2874354101164688	0.4498732581590196	T	T	T
0.1249751965425437	0.2874651061391567	0.4498234707833941	T	T	T

0.1249367415322014	0.0386307022539232	0.4509697329421447	T	T	T
0.8751936484296277	0.5368733081041653	0.4514423021119872	T	T	T
0.6249585297944629	0.0385205253922792	0.4509633819162990	T	T	T
0.1248865807265798	0.7874225275025153	0.4521204827607790	T	T	T
0.8749344104922132	0.0385301036584466	0.4509549817864300	T	T	T
0.6249855348739648	0.5366670429656417	0.4517662364362017	T	T	T
0.1247897237211310	0.5367221039719250	0.4511333023933257	T	T	T
0.8750904016243847	0.7876042855112396	0.4522280192131866	T	T	T
0.6250082360574843	0.1724309699229776	0.5513702757180298	T	T	T
0.3750405015603187	0.4205255536568632	0.5479466914374905	T	T	T
0.3756741181158263	0.9234711630867471	0.5509711757323202	T	T	T
0.3705292352440487	0.6749543164451887	0.5454601881470597	T	T	T
0.6252938173047964	0.6705264300896771	0.5481198132659011	T	T	T
0.1249896870987763	0.9243182883661782	0.5509209691812710	T	T	T
0.8796210951834201	0.6750530115384732	0.5456351172229914	T	T	T
0.8748333749116713	0.1722418966599652	0.5513412852682351	T	T	T
0.1250056993689837	0.1721643529571522	0.5513629707804287	T	T	T
0.1249948233369636	0.4212341472636282	0.5483310889411478	T	T	T
0.6250269020643477	0.4208082499923373	0.5476501782196402	T	T	T
0.3751922925487223	0.1722365515786244	0.5513368307031076	T	T	T
0.1249360448638748	0.6735993583027774	0.5471701001574064	T	T	T
0.8745402481936168	0.9235948190129679	0.5510509013454755	T	T	T
0.6251362034853529	0.9233326107285398	0.5508007462128497	T	T	T
0.8750115785549004	0.4205393628779093	0.5479752750088184	T	T	T
0.7496796021902300	0.0493474854525804	0.5752698251031404	T	T	T
0.2490528343494464	0.5490080373204602	0.5714282584387532	T	T	T
0.7527621587299448	0.5493714597274256	0.5699199987521638	T	T	T
0.5003943505591258	0.0493133055006950	0.5752623572001053	T	T	T
0.2490465426391664	0.8012559709851880	0.5722580691895754	T	T	T
0.9999496665303563	0.2980741274078996	0.5734874968159087	T	T	T
0.5004109409588389	0.7994126368025354	0.5729576678179047	T	T	T
0.2504730366225070	0.0494410459344810	0.5753529285715672	T	T	T
0.0007588552275838	0.8013747582851103	0.5724073610870163	T	T	T
0.7498625724511507	0.2986598631526039	0.5735467432741983	T	T	T
0.7499841589041782	0.7996213461604320	0.5730119508118071	T	T	T
0.9996011272994264	0.0495109752592560	0.5753695026214853	T	T	T
0.4973717492082174	0.5493444010508778	0.5698123642872039	T	T	T
0.2501283592149081	0.2980428396665324	0.5734613103003091	T	T	T
0.0007393141934242	0.5491019966262493	0.5717211194787959	T	T	T
0.5001678315509992	0.2986317945670022	0.5735631345916982	T	T	T
0.3752274896408363	0.0862169612534649	0.6783476950126238	T	T	T
0.3761768135802305	0.3372290556748040	0.6759550992824821	T	T	T
0.1250091165895206	0.0864342986497910	0.6782695403224015	T	T	T
0.8721837081241957	0.5859983745209077	0.6742785274053273	T	T	T
0.3747988942535745	0.8349523400043102	0.6759575694102755	T	T	T
0.1250364934336061	0.8356293291655176	0.6760322693323599	T	T	T
0.1251619087605726	0.3367820033427175	0.6758493074400748	T	T	T
0.8739082604391289	0.3372906734406891	0.6759646110565124	T	T	T
0.6250235422565651	0.0861735662514577	0.6783673084684331	T	T	T
0.1252026143819867	0.5863617208316275	0.6743655598668813	T	T	T
0.8748596956896048	0.0863098179302597	0.6784196815325703	T	T	T
0.6250024362547871	0.3377356894408661	0.6760664498688989	T	T	T
0.6251266006176014	0.8333035834381359	0.6754490069896208	T	T	T
0.3779875695428126	0.5859467515623650	0.6740115783570971	T	T	T

```
  0.8752401555410918  0.8352949212885974  0.6762329378772360   T   T   T
  0.2509509472441674  0.4636500184308897  0.6965298999403211   T   T   T
  0.0007339199599514  0.7125780863914046  0.6966400815506192   T   T   T
  0.7515956999295638  0.7098850794249013  0.6955344506751501   T   T   T
  0.5032074932487848  0.4680951094803860  0.6953654294063908   T   T   T
  0.9993963063762041  0.4637830581755327  0.6966641558663386   T   T   T
  0.7467922277329998  0.4680602815681991  0.6954205202553823   T   T   T
  0.4985647298702681  0.7098965366151264  0.6952520321794216   T   T   T
  0.7497999279667853  0.2134795120097748  0.7000429532452881   T   T   T
  0.5003056770657435  0.9613954087903288  0.7001672245996485   T   T   T
  0.9998563200770290  0.2137888021253924  0.6999952362016072   T   T   T
  0.2502598587894782  0.9623968639482319  0.7002094413307548   T   T   T
  0.2502983431843531  0.2134616798117489  0.6999989699125783   T   T   T
  0.9999045003431823  0.9626193115465346  0.7002700664092586   T   T   T
  0.2493344243923984  0.7123931204512109  0.6964724885824009   T   T   T
  0.7497425857406033  0.9614954065180861  0.7002594137277534   T   T   T
  0.5002410970983319  0.2134624580589666  0.7000352810266374   T   T   T
  0.6249960442602497  0.5905191205920090  0.6578666518512856   T   T   T
== Te-in vacancy containing Pd4
   1.00000000000000
    14.0906791687999995    0.0000000000000000    0.0000000000000000
     0.0000000000000000   25.2576961516000011    0.0000000000000000
     0.0000000000000000    0.0000000000000000   27.7237830675911070
   W    Te   Pd
    64   127     4
Selective dynamics
Direct
  0.3751389712273488  0.8511708448027639  0.3738056579930180   T   T   T
  0.6250362170721542  0.8510915704758863  0.3737419805307908   T   T   T
  0.1250499542567144  0.8511829550350964  0.3738415136086837   T   T   T
  0.3749722341324349  0.3514995209568710  0.3734841432372535   T   T   T
  0.8750447249005096  0.1013825989576160  0.3726501318427396   T   T   T
  0.6249910156596759  0.1014093443032629  0.3726436289348214   T   T   T
  0.1250346411046639  0.6014232283848870  0.3736240016981697   T   T   T
  0.8750572261118174  0.3514989904919230  0.3735112439849472   T   T   T
  0.1249952659936863  0.1013886712836363  0.3726218944377154   T   T   T
  0.6250024248519434  0.3515295868433540  0.3736476448954549   T   T   T
  0.8751854180751829  0.6012942726021409  0.3737600658611458   T   T   T
  0.3748572105932286  0.6012901796748062  0.3737822062334058   T   T   T
  0.3749641966487124  0.1013812133276307  0.3726518861127942   T   T   T
  0.8749454189612490  0.8511681997513585  0.3738083350322922   T   T   T
  0.6250219598292142  0.6012814463098216  0.3741302380728920   T   T   T
  0.1250044270650799  0.3513797828702527  0.3732435822472043   T   T   T
  0.7500600934065825  0.0125884858803532  0.3807411323493826   T   T   T
  0.4998739039375177  0.2625808925375883  0.3808609125206205   T   T   T
  0.2499789494642421  0.2625539468190971  0.3807808644521107   T   T   T
  0.2501527250905151  0.7623939547468793  0.3819815462656605   T   T   T
  0.7501413586126910  0.2625771169212536  0.3808757504959496   T   T   T
  0.0008450365808774  0.5123943112370367  0.3809396449379236   T   T   T
  0.4999632024403137  0.5125339153457905  0.3818099702898868   T   T   T
  0.2500539824085943  0.0125771501236991  0.3807892132282523   T   T   T
  0.7500546895198131  0.5125362951837985  0.3818075621919202   T   T   T
  0.9998752150874485  0.7623917433606190  0.3819809893486806   T   T   T
  0.4999524771714874  0.0125874003537063  0.3807392829954804   T   T   T
```

0.4999030784885114	0.7622428060037020	0.3819241459834781	T	T	T
0.7501279001045217	0.7622588057120475	0.3819266240105349	T	T	T
0.2492121401162164	0.5123929479495452	0.3809468148688253	T	T	T
0.0000318349073580	0.2625488794569728	0.3807803949394639	T	T	T
0.9999546896959832	0.0125785463518736	0.3807837412312101	T	T	T
0.5003791593215606	0.8994236204970264	0.6218495792628917	T	T	T
0.2457831633887765	0.6505554108608522	0.6263771918755666	T	T	T
0.0042076889974587	0.6505609831853298	0.6263644142156819	T	T	T
0.4757130989967997	0.6493056749653465	0.6296255863329635	T	T	T
0.2496727622013022	0.9003792735562779	0.6207684614070679	T	T	T
0.7512663031732875	0.3965564207108129	0.6252882910349217	T	T	T
0.4985699327594350	0.3965746835761604	0.6252838897662064	T	T	T
0.4997255363186383	0.1485529385581169	0.6195686670526677	T	T	T
0.7496914941218141	0.8994163113262806	0.6218693347353588	T	T	T
0.9998680467748510	0.1489269039086537	0.6199595346526426	T	T	T
0.9993889973205327	0.3981776200563024	0.6276610454319563	T	T	T
0.7502539570955614	0.1485529254112040	0.6195999501543575	T	T	T
0.2504459208639225	0.3981977426779711	0.6276938517514037	T	T	T
0.0003881549849086	0.9003830010035675	0.6207693052512445	T	T	T
0.2501226626804670	0.1489266059371470	0.6199424314301735	T	T	T
0.7743084832085909	0.6492974841197086	0.6296161599759162	T	T	T
0.1249713305307155	0.2373981999391853	0.6294516409971170	T	T	T
0.1250331159682538	0.7397502652948174	0.6304018834570060	T	T	T
0.8746750427766697	0.4861515546351393	0.6373068773524928	T	T	T
0.3700574154243492	0.7404171328194616	0.6327277972492497	T	T	T
0.1250158044468131	0.9888023681461131	0.6275065734644142	T	T	T
0.1249491715045188	0.4873138214164193	0.6365896109537463	T	T	T
0.8752177912167673	0.2369530743993103	0.6290561140772136	T	T	T
0.8747683205022569	0.9884178538087186	0.6275085472853514	T	T	T
0.3752599490591859	0.9884141063783164	0.6274919309593311	T	T	T
0.3751892265557796	0.4861775693934066	0.6373662641607685	T	T	T
0.6250116718294902	0.9881182796351828	0.6276171762780857	T	T	T
0.6249955887037828	0.2366376292870914	0.6283003005574357	T	T	T
0.6250541227261249	0.7375471352187382	0.6383875528939887	T	T	T
0.8799293377682402	0.7404166849868540	0.6327350172950915	T	T	T
0.6248986103239225	0.4812512580522875	0.6341000712088563	T	T	T
0.3747093575599517	0.2369680333004232	0.6290149362860130	T	T	T
0.9999979111656221	0.0766321412062791	0.3021881152480759	T	T	T
0.9997497233182324	0.3272313573626768	0.3027889170937690	T	T	T
0.0000142135301400	0.5771770640752293	0.3030256130481769	T	T	T
0.0000568298252370	0.8263623446733399	0.3033784959021569	T	T	T
0.2500041640717372	0.0766356746301556	0.3021980201916543	T	T	T
0.2502501309989004	0.3272271620784543	0.3027782434790266	T	T	T
0.2500862237097902	0.5771776527929160	0.3030356590880194	T	T	T
0.2500551144193351	0.8263630970961282	0.3033742170264365	T	T	T
0.4999929607007934	0.0766515232390830	0.3022067302188906	T	T	T
0.5001668121508258	0.3273402966775224	0.3029749540210125	T	T	T
0.5001739305744789	0.5768871398491052	0.3034643722094642	T	T	T
0.5000306912347284	0.8263185144755234	0.3033299072728599	T	T	T
0.7500000717313773	0.0766650328705838	0.3021890681924794	T	T	T
0.7499063621842383	0.3273602937778115	0.3030085780885882	T	T	T
0.7498796428373263	0.5768898735949228	0.3034538732664886	T	T	T
0.7500752364547941	0.8263165074064008	0.3033378303427081	T	T	T
0.6250009354370304	0.2008201169705616	0.3245325962957423	T	T	T

0.3750080401972615	0.2007450246568290	0.3244329815085222	T	T	T
0.6250208919339844	0.4507410052132765	0.3257336057660209	T	T	T
0.1250227533629028	0.7005391260523194	0.3258571965620728	T	T	T
0.6250142723029216	0.7002661891345604	0.3261348122035455	T	T	T
0.3752704631935027	0.4506806820904787	0.3254295389151903	T	T	T
0.6250045116338476	0.9501961208709989	0.3251697596112682	T	T	T
0.1250104110832944	0.4504000838488893	0.3247915166310630	T	T	T
0.8749942933188949	0.2007420985029381	0.3244403199163198	T	T	T
0.3750166700295750	0.7004040778917734	0.3259236248957340	T	T	T
0.8747481741315407	0.4506788306264660	0.3254311594621787	T	T	T
0.1250071915072969	0.9501713602712186	0.3251979455998629	T	T	T
0.8750030893660135	0.7004248677798826	0.3259189938450229	T	T	T
0.3750070189744052	0.9501873320546490	0.3251526987781575	T	T	T
0.8749952267198126	0.9501674936661647	0.3251544126172303	T	T	T
0.1250033558481183	0.2007477937240243	0.3244380746509192	T	T	T
0.7500514324824157	0.1633437918072997	0.4272126616914731	T	T	T
0.2499470379801207	0.6631631001339400	0.4288699210632050	T	T	T
0.9999425687371445	0.9135558050132834	0.4280651976532195	T	T	T
0.4998583906027598	0.6630634412347680	0.4294762680016329	T	T	T
0.4999485798065650	0.1633419585326588	0.4272042447482420	T	T	T
0.7501206677631463	0.4133603244004155	0.4288760625876873	T	T	T
0.2495396767777674	0.4133700670487421	0.4283357023929789	T	T	T
0.2499704877486772	0.1633402874040724	0.4271945572548445	T	T	T
0.0004670352951163	0.4133722181940454	0.4283394492819331	T	T	T
0.5001588738484151	0.9135798536215997	0.4279881053604843	T	T	T
0.7502183782400962	0.6630891556915672	0.4294526052115749	T	T	T
0.2501626634149489	0.9135362038613246	0.4280881418597718	T	T	T
0.4998614327410754	0.4133701893122449	0.4288511687283604	T	T	T
0.0001079852590727	0.6631484091972567	0.4288533674465044	T	T	T
0.0000325704358930	0.1633329464249187	0.4271961288168759	T	T	T
0.7498954877392338	0.9135878221763417	0.4279767456742573	T	T	T
0.3753082121730201	0.7874061361297329	0.4525587676173025	T	T	T
0.3750580999163748	0.0377620760038581	0.4512969292283214	T	T	T
0.3734696657233528	0.5366732389473589	0.4517274453393874	T	T	T
0.8751799305170634	0.2870045408940189	0.4516246676502680	T	T	T
0.6249994163723924	0.2869088465996002	0.4516329382948349	T	T	T
0.6250116949981988	0.7872732408623420	0.4524242133718866	T	T	T
0.3748632962382348	0.2870516914746821	0.4515998167124963	T	T	T
0.1249875849236610	0.2871615506235065	0.4515443338701913	T	T	T
0.1250013318361496	0.0378464896258579	0.4512646683215127	T	T	T
0.8765717108057496	0.5366773937099462	0.4517042358339051	T	T	T
0.6250041719488614	0.0378261673383134	0.4512279259838587	T	T	T
0.1250085962171039	0.7873034570175470	0.4524999401603027	T	T	T
0.8749506795944985	0.0377615727975285	0.4512946916217858	T	T	T
0.6250109278887798	0.5373544796495595	0.4526857758847828	T	T	T
0.1250370560873665	0.5371505703089055	0.4519666965643525	T	T	T
0.8747114061695417	0.7874227453737747	0.4525615099191739	T	T	T
0.6250051787075359	0.1736716064027554	0.5489708800834555	T	T	T
0.3726913935695424	0.4246789057241917	0.5568023398933655	T	T	T
0.3760550250932634	0.9222143799611862	0.5501566165074868	T	T	T
0.3687671516759486	0.6762992589587696	0.5544245384009385	T	T	T
0.6250212588870447	0.6746908680622491	0.5616384410752377	T	T	T
0.1250142728359393	0.9238403719533159	0.5495579682647062	T	T	T
0.8812414796210029	0.6763110562025978	0.5544144849337979	T	T	T

0.8752593722741439	0.1743171845828745	0.5492930237291465	T	T	T
0.1249878446414524	0.1748765244387563	0.5496325343385038	T	T	T
0.1249216486324847	0.4242849891758413	0.5575006703006860	T	T	T
0.6249096632124570	0.4218081205799118	0.5543126235945385	T	T	T
0.3747397940198046	0.1742965597141565	0.5492684923168409	T	T	T
0.1250069503515215	0.6727050587711910	0.5537360655136636	T	T	T
0.8739911880222052	0.9222185176081562	0.5501697762057711	T	T	T
0.6250491173303583	0.9219780214091121	0.5503804296321791	T	T	T
0.8770632853071861	0.4245731570858756	0.5567648768682067	T	T	T
0.7499196692329362	0.0497892768195271	0.5724162005178545	T	T	T
0.2478034447351568	0.5500616549094280	0.5819717250717444	T	T	T
0.7754376409159977	0.5483480088294047	0.5726461989337633	T	T	T
0.5001057152531694	0.0497938202551490	0.5724031509932603	T	T	T
0.2498056898411081	0.8010583953231061	0.5744986179509526	T	T	T
0.0000916613358412	0.3012788396250417	0.5767074052941287	T	T	T
0.5016961061984606	0.7984050830564788	0.5789303115780514	T	T	T
0.2502608334930642	0.0501665786742176	0.5726670228378018	T	T	T
0.0001779740802310	0.8010454010558061	0.5745359570400749	T	T	T
0.7509370012351001	0.2999288829604083	0.5749851898792067	T	T	T
0.7483183118183433	0.7984085446029157	0.5789355873011965	T	T	T
0.9997421935616703	0.0501693414231212	0.5726871212690957	T	T	T
0.4745010422975733	0.5483909028648325	0.5727106680780406	T	T	T
0.2497532037660316	0.3013333378914286	0.5767002174020219	T	T	T
0.0020934154392748	0.5500510790220675	0.5819440291699501	T	T	T
0.4990263334730897	0.2999273732633089	0.5749790092073634	T	T	T
0.3751452641855377	0.0869444536619039	0.6757335651942745	T	T	T
0.3751543834251492	0.3334158482366886	0.6808434362192367	T	T	T
0.1249975418493294	0.0871711546296998	0.6757481433623839	T	T	T
0.8931929196485853	0.5907595501130506	0.6920291538359555	T	T	T
0.3734818663325996	0.8399382513141819	0.6791068172306771	T	T	T
0.1250466940477893	0.8393941335052384	0.6770813686846857	T	T	T
0.1249549404441006	0.3342183983134891	0.6814515686512865	T	T	T
0.8747977027233501	0.3334199566021636	0.6808547265824192	T	T	T
0.6249873714118162	0.0868179370726579	0.6754658143502552	T	T	T
0.1249778748312654	0.5875432062687126	0.6841617001310555	T	T	T
0.8748224247028406	0.0869553079117491	0.6757599980428000	T	T	T
0.6249844475478186	0.3328735279182432	0.6789388938941796	T	T	T
0.6250201861467991	0.8397954663745019	0.6798378131052749	T	T	T
0.3568030977151299	0.5907451824657851	0.6920374350874319	T	T	T
0.8767177889986161	0.8399640648409948	0.6791314206255731	T	T	T
0.2492953525438832	0.4609562992927180	0.7073123950082505	T	T	T
0.0043502826870195	0.7175013445783806	0.7033631374381895	T	T	T
0.7579866429411484	0.7186898347855105	0.7074046683218382	T	T	T
0.5008844565863069	0.4551007822221078	0.7078883638073652	T	T	T
0.0005441240357079	0.4609489129643575	0.7072708634825614	T	T	T
0.7490271404761523	0.4551082664692125	0.7078747634112657	T	T	T
0.4920738470315526	0.7186943184197206	0.7073946311623541	T	T	T
0.7495971452532582	0.2111563605873511	0.6988105195067891	T	T	T
0.4998799726240146	0.9655011890598172	0.6986066918094634	T	T	T
0.9999208200411864	0.2112158638584414	0.6993954840345509	T	T	T
0.2499578471216566	0.9654568194091431	0.6983910170414501	T	T	T
0.2500503207868754	0.2112151429206040	0.6993826901868350	T	T	T
0.0000946555304111	0.9654608769972070	0.6983903032184051	T	T	T
0.2456827383308538	0.7175115908195083	0.7033816872134329	T	T	T

```
  0.7501226391217366  0.9655018146349309  0.6986144484128344   T   T   T
  0.5002731637757823  0.2111647406358112  0.6987508770590607   T   T   T
  0.6250043980577406  0.5850545355868274  0.6091792214677153   T   T   T
  0.5248023319981282  0.5545637198015689  0.6845247305075625   T   T   T
  0.7252259486461700  0.5545179441210820  0.6844653867560546   T   T   T
  0.6250625924290332  0.6442292313678947  0.6929242322827940   T   T   T
== Te-in vacancy containing Pd7
   1.00000000000000
    24.6586885454000111    0.0000000000000000    0.0000000000000000
     0.0000000000000000   25.2576961516000082    0.0000000000000000
     0.0000000000000000    0.0000000000000000   30.0000000000007034
   W    Te   Pd
    56   111    7
Selective dynamics
Direct
  0.0715865027584490  0.0977159101246328  0.4919206054572614   T   T   T
  0.2139276696133562  0.0975473159177348  0.4928378062132715   T   T   T
  0.3566480774927769  0.0976908888087290  0.4949662086659630   T   T   T
  0.5005481952761627  0.0985108569814859  0.4965077682399988   T   T   T
  0.6424250580212928  0.0983366169462388  0.4963251816202082   T   T   T
  0.7861828060330651  0.0974326104638926  0.4945521673630843   T   T   T
  0.9291124028096026  0.0973443929861196  0.4923959902134836   T   T   T
  0.0713454919649967  0.3484913146979046  0.4946556591913338   T   T   T
  0.2117345613894062  0.3481585790644686  0.4949107831006364   T   T   T
  0.3511347548329509  0.3460415009919922  0.4953757269461933   T   T   T
  0.4850610849684711  0.3491554049521304  0.4953053548111278   T   T   T
  0.6577082601625345  0.3489506707520473  0.4956513134800125   T   T   T
  0.7916752908193977  0.3457264340168825  0.4958511719416790   T   T   T
  0.9311422402666392  0.3480588716054924  0.4951253463415285   T   T   T
  0.0714973482305915  0.5996786266659574  0.4958479618692108   T   T   T
  0.2135445113295628  0.5998406062060593  0.4964911948288341   T   T   T
  0.3534960163034042  0.6006853184879029  0.4978963955257065   T   T   T
  0.4915700165986062  0.5998194773123158  0.4980873071479640   T   T   T
  0.6512108409970678  0.5997434784854430  0.4983471317737561   T   T   T
  0.7892661311803375  0.6004716549465450  0.4982830223949943   T   T   T
  0.9292422603202913  0.5998074069187821  0.4967981166213676   T   T   T
  0.0716120292686717  0.8487935193409496  0.4930249068179530   T   T   T
  0.2145966298434537  0.8490131385777111  0.4936152129797509   T   T   T
  0.3571308472156973  0.8491797275090506  0.4944213321922816   T   T   T
  0.4999147008283792  0.8485064161390220  0.4943277003504104   T   T   T
  0.6426973860536657  0.8482883348527726  0.4943512198136271   T   T   T
  0.7856113660580522  0.8490116104424447  0.4941196483929180   T   T   T
  0.9283563765375724  0.8488459932850134  0.4934045744971698   T   T   T
  0.0000795202915708  0.0089244874638178  0.4988050403520249   T   T   T
  0.1430749601378719  0.0089941273202481  0.4990513851881250   T   T   T
  0.2857993566921442  0.0090513657347649  0.5003396692553433   T   T   T
  0.4287811233828566  0.0093975871035145  0.5015229030528128   T   T   T
  0.5715253339082930  0.0093172534010357  0.5020828419664012   T   T   T
  0.7141010914945329  0.0091333333592598  0.5011637302108153   T   T   T
  0.8570869741228516  0.0088057093035814  0.4998408462409702   T   T   T
  0.0007882583787611  0.2585280609313377  0.4997591298131316   T   T   T
  0.1423103758896209  0.2586390839655116  0.4997718070196450   T   T   T
  0.2827579388957608  0.2576795006645357  0.5004341648216079   T   T   T
  0.4251637705709384  0.2570986735232711  0.5026329805583831   T   T   T
```

0.5715969499617638	0.2617421716152160	0.5069872661656631	T	T	T
0.7178309203271048	0.2568051415375339	0.5028649860082464	T	T	T
0.8602170999111058	0.2574940705894087	0.5005779519496546	T	T	T
0.0007176650528756	0.5101391023538026	0.5025101665569947	T	T	T
0.1422295254616844	0.5100897942558966	0.5022290278978475	T	T	T
0.2826103605891729	0.5103261530668382	0.5034641625127740	T	T	T
0.4200548762049766	0.5116115993183907	0.5050956029111123	T	T	T
0.5714876363077445	0.5211596063700539	0.5112411263585178	T	T	T
0.7225434638157481	0.5114008725518694	0.5054913145079656	T	T	T
0.8600583072257496	0.5101508771707874	0.5039660923007458	T	T	T
0.9999176267580634	0.7603739282368708	0.5009586644228005	T	T	T
0.1431737512287555	0.7603228578619751	0.5010115142236555	T	T	T
0.2854028416563337	0.7605876265090666	0.5016815068008126	T	T	T
0.4276016850249046	0.7605353781781869	0.5019499749808061	T	T	T
0.5712460858514165	0.7590313460569000	0.5011492970658206	T	T	T
0.7150810547306968	0.7603977665818508	0.5020689334988429	T	T	T
0.8573267075622275	0.7604516380863096	0.5016642555912154	T	T	T
0.1432458832464948	0.0736102214224940	0.4268937952003239	T	T	T
0.2866272079987508	0.0740508993271237	0.4284145073353459	T	T	T
0.4296291827227559	0.0757956754475533	0.4303674089634918	T	T	T
0.5713110081430038	0.0752408728729384	0.4307485963161927	T	T	T
0.7131826121935251	0.0755342394151712	0.4299814213291621	T	T	T
0.8564258177771790	0.0739056718364015	0.4280304588516481	T	T	T
0.0001721414956801	0.0736623370418458	0.4265773708136971	T	T	T
0.1414551858454974	0.3253553050257570	0.4286639476883142	T	T	T
0.2813940364956472	0.3234821783051666	0.4288349041654964	T	T	T
0.4209192472919076	0.3175755424398179	0.4288677418545338	T	T	T
0.5714448254681050	0.3253709843007568	0.4354207282799261	T	T	T
0.7218223739167728	0.3174606999337056	0.4292386560324741	T	T	T
0.8613092662927490	0.3234242373410978	0.4291399403895998	T	T	T
0.0011984343244324	0.3252173284080089	0.4287209832323529	T	T	T
0.1426876526312235	0.5756391256270977	0.4306301646273331	T	T	T
0.2843231705489867	0.5753302830726695	0.4313407106619187	T	T	T
0.4238918293051445	0.5747591375383510	0.4318995082827625	T	T	T
0.5714216680377167	0.5769098097635591	0.4367113895745281	T	T	T
0.7191193426867496	0.5744358856860858	0.4322870152285012	T	T	T
0.8584556952800039	0.5749049508025569	0.4318007119734147	T	T	T
0.9999732648564813	0.5755450028128344	0.4308694358141077	T	T	T
0.1433914040327019	0.8238598680403969	0.4282094924080995	T	T	T
0.2862502480253913	0.8244118389554357	0.4288103032185721	T	T	T
0.4284551215532152	0.8243796086481340	0.4291545578544519	T	T	T
0.5712660011695526	0.8237285849865124	0.4291359737256470	T	T	T
0.7141061183814934	0.8240020857515262	0.4291195086748846	T	T	T
0.8566507064065593	0.8239085044366964	0.4286187696235548	T	T	T
0.9998199937261736	0.8240312768911918	0.4281379381444480	T	T	T
0.0715870809889269	0.1966565103942164	0.4474466417170491	T	T	T
0.2127018521888912	0.1960896488322269	0.4477141161346698	T	T	T
0.3553435165643514	0.1953977150265477	0.4501243571316439	T	T	T
0.5010165346590316	0.1982118800607220	0.4535352190338592	T	T	T
0.6419944477280611	0.1982123403314767	0.4535401335022964	T	T	T
0.7875813531679020	0.1954104226790913	0.4500915037181965	T	T	T
0.9303073020480591	0.1961187961290905	0.4476317018488255	T	T	T
0.0713126621295151	0.4479386201132709	0.4504766192568714	T	T	T
0.2124968881698059	0.4476607440139835	0.4507099470257575	T	T	T

0.3511222447897807	0.4461368663425253	0.4523603607271066	T	T	T
0.4763576349482571	0.4462957850763634	0.4433940333652717	T	T	T
0.6667321383743146	0.4460385141153253	0.4437079609255498	T	T	T
0.7917316725012808	0.4456766840855962	0.4528754617295607	T	T	T
0.9303005123393120	0.4476874971306183	0.4512398470483314	T	T	T
0.0714970718761509	0.6979533756495195	0.4501117192114358	T	T	T
0.2141594571689046	0.6978494043503669	0.4504804938537025	T	T	T
0.3560436741240467	0.6979216876418870	0.4510429791465170	T	T	T
0.4978812227118120	0.6966875129318355	0.4511003019954736	T	T	T
0.6448499788312958	0.6965202611973168	0.4513838437361499	T	T	T
0.7867055075044880	0.6976416068694513	0.4512844176829335	T	T	T
0.9287116968569211	0.6977271679481352	0.4506507257827296	T	T	T
0.0716634646556807	0.9469764785429656	0.4475474710185965	T	T	T
0.2150635478437584	0.9472325666073916	0.4481637456588391	T	T	T
0.3580326695448373	0.9475610121147275	0.4493114747370778	T	T	T
0.5004218169253322	0.9476618611749040	0.4499686555700566	T	T	T
0.6424215486659696	0.9472351778499897	0.4498943892733185	T	T	T
0.7848440271595679	0.9474158289652440	0.4486878847944124	T	T	T
0.9280976584624870	0.9469781850051583	0.4478892966419147	T	T	T
0.0008228501552592	0.1593888908954138	0.5437338002844287	T	T	T
0.1421714966292792	0.1596837143729716	0.5440238211768954	T	T	T
0.2836507827342307	0.1592812388981739	0.5453405218850131	T	T	T
0.4274282597553336	0.1592177076163131	0.5477271157392902	T	T	T
0.5716122705168800	0.1605533476390938	0.5486051364941987	T	T	T
0.7156479672425575	0.1586390488897915	0.5476470586684691	T	T	T
0.8593086542267058	0.1588815169413563	0.5449688064855499	T	T	T
0.0015420926534225	0.4112053671503994	0.5470245381825377	T	T	T
0.1412540896213127	0.4112139480465192	0.5467676723857430	T	T	T
0.2797660153717948	0.4107576114707526	0.5476649668805978	T	T	T
0.4088096063409919	0.4080236564117664	0.5572989929026575	T	T	T
0.7337540908806617	0.4075608024524625	0.5577990335762691	T	T	T
0.8630169520396723	0.4104141023158484	0.5481066792920258	T	T	T
0.0007417693242130	0.6627006991507842	0.5471236043815427	T	T	T
0.1421796937186235	0.6626131226948857	0.5471297223946899	T	T	T
0.2831739751269147	0.6633338322647655	0.5485907763393689	T	T	T
0.4252636571050781	0.6633527369004104	0.5493582198975391	T	T	T
0.5712895752864379	0.6601543545164491	0.5456313506444435	T	T	T
0.7176463585647403	0.6633093870636189	0.5496509442057815	T	T	T
0.8594723008777425	0.6633225828641196	0.5488149028948019	T	T	T
0.0001508711746396	0.9109845775786380	0.5440892458904665	T	T	T
0.1427285062848053	0.9110007725526185	0.5442707639969867	T	T	T
0.2853907916215335	0.9110200572872992	0.5452574934954422	T	T	T
0.4286682630621366	0.9106969188051927	0.5456577106552620	T	T	T
0.5713654182965017	0.9100706863314028	0.5458320748717335	T	T	T
0.7141222638898602	0.9105284862008980	0.5454485067942387	T	T	T
0.8574380170289319	0.9108558062296520	0.5448259453836178	T	T	T
0.0713964154998935	0.0333010901720687	0.5640939422596001	T	T	T
0.2135902814962094	0.0330540332470345	0.5650227939624929	T	T	T
0.3564573122087059	0.0321940926828423	0.5665465195480806	T	T	T
0.4998548697369873	0.0321521862658739	0.5675290039751306	T	T	T
0.6433631350392469	0.0320788097863729	0.5673667067967630	T	T	T
0.7865310030505261	0.0319098044907504	0.5661038795567858	T	T	T
0.9292341028883562	0.0329572358926301	0.5645709870627228	T	T	T
0.0715288820015642	0.2819872209345663	0.5656607127199513	T	T	T

```
  0.2120677889521078  0.2816271809610583  0.5661349242467548   T   T   T
  0.3525613779866564  0.2806237498793344  0.5671064708979663   T   T   T
  0.4950976373659912  0.2811341599402949  0.5710283732469725   T   T   T
  0.6480275632587423  0.2809583669469993  0.5711670666155019   T   T   T
  0.7905281018542628  0.2799926986808273  0.5674205165960087   T   T   T
  0.9311942655997775  0.2814069494831065  0.5661760406266030   T   T   T
  0.0715778335824777  0.5349328743202166  0.5678455954042555   T   T   T
  0.2113072447367342  0.5349782353218850  0.5685964137752179   T   T   T
  0.3504456079361731  0.5366690725033626  0.5707722908808865   T   T   T
  0.4930623003917122  0.5394888243464961  0.5741113488998539   T   T   T
  0.6496643796148815  0.5394492273473825  0.5744383365086431   T   T   T
  0.7921365945748133  0.5364862459107010  0.5711845420573937   T   T   T
  0.9315865591676867  0.5350747314756367  0.5689061516038224   T   T   T
  0.0715069738977497  0.7856014821584181  0.5658711798765026   T   T   T
  0.2136793362091330  0.7857551976127781  0.5665339172733500   T   T   T
  0.3563588928697731  0.7853453691041637  0.5671500463129604   T   T   T
  0.5001605286568365  0.7839262465746102  0.5665363886979782   T   T   T
  0.6424564189336648  0.7835968533070490  0.5666120864044067   T   T   T
  0.7864820108899657  0.7855866533120612  0.5670450869417195   T   T   T
  0.9291707498501612  0.7857050246093477  0.5664171580094866   T   T   T
  0.5712019588705843  0.3602886287466202  0.5466682187864306   T   T   T
  0.6402143573426438  0.4437341843836262  0.5337426107620623   T   T   T
  0.5024319623389737  0.4439238842224177  0.5334287701205460   T   T   T
  0.5714589411610886  0.4229912285076314  0.4695038196383811   T   T   T
  0.5711399049572896  0.4630465863928137  0.5995307325289435   T   T   T
  0.6494451215298057  0.3769447364627598  0.6048412271178841   T   T   T
  0.4927390411100435  0.3772799278263794  0.6046594961265765   T   T   T
== Separated Te adatom and Pd adatom
   1.00000000000000
    24.6586885454000111    0.0000000000000000    0.0000000000000000
     0.0000000000000000   25.2576961516000082    0.0000000000000000
     0.0000000000000000    0.0000000000000000   22.0000000000007034
   W    Te   Pd
    56   113     1
Selective dynamics
Direct
  0.0711591688119252  0.0990515191369577  0.4951508367701988   T   T   T
  0.2145065488063408  0.0987619385073427  0.4949312880713848   T   T   T
  0.3566906880368012  0.0987247200158596  0.4952374196018834   T   T   T
  0.4997944929876913  0.0986285965985599  0.4949378207122221   T   T   T
  0.6434716070361278  0.0989053777435794  0.4951666860861438   T   T   T
  0.7863493485989834  0.0993262846494836  0.4953455698709401   T   T   T
  0.9281122377271628  0.0992188625602372  0.4953127661671444   T   T   T
  0.0709523493798908  0.3492068177130181  0.4958973628432487   T   T   T
  0.2126703597548567  0.3490326372532835  0.4970706667727002   T   T   T
  0.3563346398178920  0.3478953460696727  0.5001530036635352   T   T   T
  0.4979551108139356  0.3477165128345390  0.4950956840492006   T   T   T
  0.6470593058616604  0.3480031407993484  0.4936988573101896   T   T   T
  0.7868407537665703  0.3491579547090060  0.4944739746434388   T   T   T
  0.9282409673251888  0.3493933550428504  0.4956355976441610   T   T   T
  0.0709506157617229  0.5995996655133025  0.4960160089643164   T   T   T
  0.2144385609123893  0.5998533131447764  0.4960927767864451   T   T   T
  0.3567259317998842  0.5994290977479825  0.4968710845702544   T   T   T
  0.4991938850729020  0.6000393114568955  0.4953461517344394   T   T   T
```

0.6441396154270468	0.5999242332213874	0.4952567054014528	T	T	T
0.7861599895449898	0.5995810397824487	0.4955979396311849	T	T	T
0.9278219301662787	0.5994039971550140	0.4957872768490094	T	T	T
0.0709505974639686	0.8491774127500206	0.4955273804702562	T	T	T
0.2147238795249311	0.8491863404570921	0.4955181938914026	T	T	T
0.3568115280141413	0.8492707352061448	0.4956082830232110	T	T	T
0.4993699028285271	0.8491124254469932	0.4954895608491370	T	T	T
0.6436198921484801	0.8492928187637434	0.4957089401909590	T	T	T
0.7863893603504009	0.8495324211764943	0.4959045143265526	T	T	T
0.9278141219353628	0.8493721010010946	0.4957906899987656	T	T	T
0.9994526070247572	0.0101046973160785	0.5042858825640804	T	T	T
0.1430566827615188	0.0099502552759621	0.5039571433222945	T	T	T
0.2858456218712631	0.0097891428744124	0.5038209466438176	T	T	T
0.4278847955845250	0.0098525032326843	0.5038844008062158	T	T	T
0.5715939295394263	0.0099205852779755	0.5042455628771741	T	T	T
0.7151954418791089	0.0101681565990051	0.5043729820400202	T	T	T
0.8571860209982025	0.0101261096334283	0.5043196627429475	T	T	T
0.9995881824846324	0.2601062495465460	0.5041843998117733	T	T	T
0.1429847414483834	0.2597697026524076	0.5046077785249111	T	T	T
0.2858063505734980	0.2593785453088441	0.5052942929676780	T	T	T
0.4274663300678534	0.2588932887425466	0.5046962085553868	T	T	T
0.5716969634146291	0.2601408966882068	0.5052783264589346	T	T	T
0.7153015098259528	0.2594261843248624	0.5025472690401310	T	T	T
0.8575586762521628	0.2600077977747119	0.5033643051016163	T	T	T
0.9992940346139887	0.5103859640308817	0.5047891297783462	T	T	T
0.1426593068724203	0.5107127798457518	0.5055052740851868	T	T	T
0.2856630962386771	0.5109814855050766	0.5057667217753579	T	T	T
0.4281287981828709	0.5110346463230910	0.5054831076092812	T	T	T
0.5720213265868561	0.5117244523167780	0.5044438860633660	T	T	T
0.7152815411867283	0.5103933729530610	0.5040769070489918	T	T	T
0.8568871519628788	0.5101964444214627	0.5042513550219613	T	T	T
0.9990867041545973	0.7602521951770557	0.5045502279769221	T	T	T
0.1430510113866681	0.7604062768404095	0.5045693738264118	T	T	T
0.2859434831918621	0.7601954760428131	0.5046074219126239	T	T	T
0.4276676220600009	0.7603215091458021	0.5046062817213097	T	T	T
0.5715505376323169	0.7605295497828262	0.5047572927258935	T	T	T
0.7154317101075681	0.7605330222733171	0.5047173737660369	T	T	T
0.8570851435883352	0.7602123388955503	0.5044098886249062	T	T	T
0.1427672996960605	0.0752934110651219	0.4060434103880649	T	T	T
0.2857031621073602	0.0749515529285424	0.4056249769751721	T	T	T
0.4283569120951024	0.0752446182189719	0.4058963977572519	T	T	T
0.5717738254367193	0.0749953862011040	0.4062769468572011	T	T	T
0.7148875833965325	0.0751665068931250	0.4061806786843222	T	T	T
0.8570903543521475	0.0750867148219808	0.4059287927430589	T	T	T
0.9996880062821724	0.0751255458673437	0.4061986576424713	T	T	T
0.1426875190451260	0.3251402254875915	0.4068755133574229	T	T	T
0.2857623649480461	0.3265804670788637	0.4093613448569291	T	T	T
0.4255644861785234	0.3255688540131189	0.4078228005645732	T	T	T
0.5715156221888023	0.3241328840526197	0.4071194460167487	T	T	T
0.7165248416563015	0.3246820336766449	0.4039215270585664	T	T	T
0.8582965733122826	0.3254087213977868	0.4053694974735018	T	T	T
0.9999553260384724	0.3253853477505763	0.4063845247984414	T	T	T
0.1427590612699568	0.5755629989270947	0.4073074237691992	T	T	T
0.2857845961753074	0.5757027375428130	0.4071182992387376	T	T	T

0.4276995334670175	0.5757818785195813	0.4068423981383685	T	T	T
0.5715255667513527	0.5765844519967739	0.4066970743167713	T	T	T
0.7149859020139309	0.5759353555894176	0.4061066490335403	T	T	T
0.8571061818850031	0.5755447098825707	0.4062003315496596	T	T	T
0.9997165825915770	0.5756816113835259	0.4068780149969091	T	T	T
0.1428280434538572	0.8254823130054262	0.4066513136023158	T	T	T
0.2857836247394307	0.8250499692215010	0.4062252026299346	T	T	T
0.4282216131437034	0.8251830030560511	0.4063038084566052	T	T	T
0.5715953319873837	0.8255278659641290	0.4068953995200510	T	T	T
0.7148291853620136	0.8254464968261692	0.4065989644092220	T	T	T
0.8569884398074636	0.8254449352502712	0.4062510918019164	T	T	T
0.9994323493359816	0.8253208319393001	0.4066448783228563	T	T	T
0.0711375628194412	0.1977610954026503	0.4343094340914764	T	T	T
0.2142426384674874	0.1977011026694183	0.4342129783889420	T	T	T
0.3566849629395026	0.1973445558622673	0.4341998401231338	T	T	T
0.5000125236978802	0.1977675140212768	0.4346882868748947	T	T	T
0.6427800940048544	0.1975042927514465	0.4338872435510134	T	T	T
0.7865086933299473	0.1973290714673659	0.4329418338968911	T	T	T
0.9286068808359098	0.1974797830939582	0.4334928771245603	T	T	T
0.0712592511032449	0.4480553524136258	0.4352506754624904	T	T	T
0.2137736095156294	0.4480933421848159	0.4356583852879007	T	T	T
0.3565781386159187	0.4464602197811439	0.4386844965635151	T	T	T
0.4993168986993373	0.4483626011492816	0.4349873317585805	T	T	T
0.6440269333885564	0.4482824199946960	0.4338688041338509	T	T	T
0.7864029485459508	0.4478464366149191	0.4335689441375648	T	T	T
0.9285117124270958	0.4477531044960507	0.4345160895934304	T	T	T
0.0710985242604352	0.6982146053455375	0.4348148280275483	T	T	T
0.2144840977112537	0.6981523243107124	0.4346509775169954	T	T	T
0.3568966024330646	0.6976302572076694	0.4345125267438345	T	T	T
0.4997691050977719	0.6987085955448078	0.4346023993699924	T	T	T
0.6434992713297063	0.6989165329638201	0.4347201935241392	T	T	T
0.7860534383864625	0.6980789747819997	0.4340875908171145	T	T	T
0.9281883803747596	0.6977807191679410	0.4341990275045841	T	T	T
0.0711679773443880	0.9477166634627758	0.4344297510146009	T	T	T
0.2143369711042641	0.9474985907474536	0.4338857343128302	T	T	T
0.3569938153938185	0.9473607705976892	0.4337132364147579	T	T	T
0.4998482484327441	0.9477713573427432	0.4343696424609578	T	T	T
0.6433878914148634	0.9477720975833712	0.4347122360710046	T	T	T
0.7860863882884180	0.9477217468876536	0.4341733874440553	T	T	T
0.9282424520032512	0.9475795026214129	0.4343162885174178	T	T	T
0.9997876807483169	0.1614485343917466	0.5652418597943597	T	T	T
0.1429956271389443	0.1608784642374236	0.5651552406084325	T	T	T
0.2853900456537639	0.1606042197536223	0.5658030175472953	T	T	T
0.4287605761438824	0.1604235072783178	0.5654619554939249	T	T	T
0.5716022378255208	0.1609965768489081	0.5650015014635794	T	T	T
0.7145670747430402	0.1617075668849108	0.5649495908263733	T	T	T
0.8571736505027513	0.1619029126854824	0.5653652133598519	T	T	T
0.9994369047348119	0.4116151248315880	0.5657804657770957	T	T	T
0.1413154627130917	0.4116541388962925	0.5668908757358397	T	T	T
0.2791783817028994	0.4142983233826288	0.5683435763434966	T	T	T
0.4347529342889193	0.4135539484789824	0.5673521016548121	T	T	T
0.5732558649276783	0.4114167388570314	0.5571024249138454	T	T	T
0.7152618699527592	0.4109704232820274	0.5649233139588721	T	T	T
0.8569374815040673	0.4116458366757379	0.5657185911664899	T	T	T

```
  0.9994028931306495  0.6617700122470914  0.5656591500656706   T   T   T
  0.1425931321754926  0.6620783741165241  0.5656676586533534   T   T   T
  0.2856027242577981  0.6621670584652684  0.5664658025712024   T   T   T
  0.4281262471629034  0.6621909850717029  0.5660894041435262   T   T   T
  0.5716047830341142  0.6616777532801698  0.5648527685812023   T   T   T
  0.7149121647629554  0.6619419078529321  0.5656510406911086   T   T   T
  0.8569849421712660  0.6619611525157219  0.5660419169642192   T   T   T
  0.9996818367262813  0.9115181531497231  0.5654772728428303   T   T   T
  0.1429194355291428  0.9113838671799447  0.5652547423628606   T   T   T
  0.2856874691969999  0.9117335836334552  0.5655761207259904   T   T   T
  0.4282884499877226  0.9116097023895620  0.5655341461962630   T   T   T
  0.5713031968177709  0.9112551213901900  0.5652877672616418   T   T   T
  0.7147149221261353  0.9116531287018468  0.5657355029398805   T   T   T
  0.8572053240595822  0.9118712256053315  0.5662985522442847   T   T   T
  0.0714559515261471  0.0338758955398250  0.5930205342536610   T   T   T
  0.2143969823788847  0.0337091657378781  0.5930738893244308   T   T   T
  0.3570419672452548  0.0337270031325161  0.5932902263514405   T   T   T
  0.4997960920718698  0.0336476076793646  0.5928880214794882   T   T   T
  0.6431170463649855  0.0339356943242021  0.5931797713943272   T   T   T
  0.7860488481289071  0.0343161225562875  0.5937094491254737   T   T   T
  0.9284351715899510  0.0342183125723413  0.5935884788677939   T   T   T
  0.0711229640090564  0.2834796698779506  0.5934743006872453   T   T   T
  0.2139745620635982  0.2833356517446269  0.5942922248935024   T   T   T
  0.3570248723432274  0.2763431097388405  0.5970166389597071   T   T   T
  0.4999798426101293  0.2847842056456235  0.5938859211842142   T   T   T
  0.6440433855053976  0.2849606876275181  0.5929219288279098   T   T   T
  0.7859183572232842  0.2838082842081139  0.5924111799967867   T   T   T
  0.9282604094936312  0.2836631444402994  0.5933647524256075   T   T   T
  0.0706090183915031  0.5345076256599449  0.5940757131491892   T   T   T
  0.2137426083793793  0.5357836241834947  0.5945240943602567   T   T   T
  0.3571069394812968  0.5334677255109551  0.5952339710851909   T   T   T
  0.5007563051109343  0.5359569245237112  0.5935288931434156   T   T   T
  0.6437252181138634  0.5347598524724709  0.5931451511014797   T   T   T
  0.7859846028625931  0.5342593248340112  0.5937080023063405   T   T   T
  0.9279798447114017  0.5341860446311834  0.5938309703161807   T   T   T
  0.0710860801853490  0.7840171397026786  0.5933910169917728   T   T   T
  0.2142917821329608  0.7843133908229054  0.5937245313069784   T   T   T
  0.3567912521340858  0.7845743201691071  0.5940904434076500   T   T   T
  0.4997422285872938  0.7841304868799043  0.5935518869466346   T   T   T
  0.6434158689202625  0.7842813212426205  0.5936649098728035   T   T   T
  0.7863460404204872  0.7844251292254126  0.5940963112181418   T   T   T
  0.9282415711370272  0.7842656962793341  0.5938862878377846   T   T   T
  0.5720699845831945  0.3942125646057069  0.6767574200463794   T   T   T
  0.3572121232866570  0.3756131062657602  0.6286223409405618   T   T   T
== Te adatom bound to Pd1
   1.00000000000000
     24.6586885454000111    0.0000000000000000    0.0000000000000000
      0.0000000000000000   25.2576961516000082    0.0000000000000000
      0.0000000000000000    0.0000000000000000   22.0000000000007034
   W    Te   Pd
    56   113     1
Selective dynamics
Direct
  0.0711095899010736  0.0990290537124709  0.4950978056236087   T   T   T
```

0.2137814447715019	0.0987441933325912	0.4946575405216891	T	T	T
0.3559345978431616	0.0987487246132065	0.4947006236196766	T	T	T
0.4991123933549493	0.0985263562258861	0.4953136674151898	T	T	T
0.6432134386532892	0.0990086087818892	0.4953315301183534	T	T	T
0.7867494751626036	0.0992166391284487	0.4953000093824707	T	T	T
0.9286126046794317	0.0992126637422713	0.4951990869483821	T	T	T
0.0709657180879152	0.3494689456286871	0.4958638606086467	T	T	T
0.2129144883740819	0.3493662944156496	0.4956824874788740	T	T	T
0.3535626291017389	0.3482804547586335	0.4956833924311625	T	T	T
0.4964866157249703	0.3473062975233497	0.4992408925897533	T	T	T
0.6469350704365244	0.3480084374298380	0.4949326409141901	T	T	T
0.7873185249254566	0.3488738435579390	0.4944409129253428	T	T	T
0.9289295827124169	0.3492928047975326	0.4953984994459704	T	T	T
0.0708570260670575	0.5994173545567161	0.4959473513171940	T	T	T
0.2141685792986335	0.5994246546028111	0.4960965632308456	T	T	T
0.3562330915595108	0.5993963714551070	0.4958582245999040	T	T	T
0.4981883691948424	0.5995771471537418	0.4962512804599744	T	T	T
0.6445102537984474	0.6000699637464734	0.4952248803697224	T	T	T
0.7868257148244359	0.5995950613692375	0.4957189462054029	T	T	T
0.9281426291809285	0.5994547818014014	0.4958435192718944	T	T	T
0.0708939713641799	0.8490142768481171	0.4956537073162451	T	T	T
0.2141294048763061	0.8489900114168312	0.4955552065909556	T	T	T
0.3563462209753850	0.8491510596040588	0.4954497812882944	T	T	T
0.4991160310904860	0.8490413575658408	0.4954301730495290	T	T	T
0.6434261536829859	0.8492977345359132	0.4956160900150028	T	T	T
0.7866758339697468	0.8495895290080262	0.4958384800520819	T	T	T
0.9282197098620265	0.8493777106869581	0.4957885961955122	T	T	T
0.9997007007384026	0.0099779411562731	0.5041036147391176	T	T	T
0.1425706588608387	0.0097311777543260	0.5038881604852911	T	T	T
0.2850135266045365	0.0096691670184158	0.5036343747376765	T	T	T
0.4274309263248558	0.0098213481308443	0.5039930623077206	T	T	T
0.5710916657033437	0.0100157884564367	0.5045420563088748	T	T	T
0.7153348655695080	0.0103068755371023	0.5044227045043373	T	T	T
0.8576903107817371	0.0101613222789580	0.5041131609505008	T	T	T
0.0000290072652618	0.2599896496578511	0.5038387183366680	T	T	T
0.1424166589026154	0.2599655605748925	0.5037981812458142	T	T	T
0.2837265321999874	0.2594412243698668	0.5032483807279342	T	T	T
0.4283649015337475	0.2587953452636389	0.5044020283576099	T	T	T
0.5700787805876163	0.2603150914399378	0.5067667603743412	T	T	T
0.7156931210669406	0.2594385680650945	0.5034742513434030	T	T	T
0.8583004453093719	0.2598751788727831	0.5032796824871294	T	T	T
0.9994061632411835	0.5102893080495370	0.5045912564349694	T	T	T
0.1427054205224645	0.5103453131753088	0.5049081518144242	T	T	T
0.2851539144364207	0.5102550528802554	0.5051250671812015	T	T	T
0.4272416400423653	0.5103917278061852	0.5056988620516665	T	T	T
0.5716383478907883	0.5123896475501933	0.5056666484122623	T	T	T
0.7162670115189654	0.5105859604608980	0.5046887726379685	T	T	T
0.8575311515609860	0.5100590362070712	0.5041876827666818	T	T	T
0.9993729502639827	0.7601285757317425	0.5044497008030940	T	T	T
0.1427638657770897	0.7600565783920038	0.5045099289067123	T	T	T
0.2853892634370667	0.7600615961018842	0.5043660370912616	T	T	T
0.4274157809534219	0.7602022387004385	0.5045324547493757	T	T	T
0.5712266877128007	0.7604262085079485	0.5047059607978975	T	T	T
0.7155942130974179	0.7606224447765982	0.5047604648439793	T	T	T

0.8575142341896127	0.7603417408127041	0.5043682062472540	T	T	T
0.1422373664467619	0.0748030135389352	0.4057819807942060	T	T	T
0.2850738208193911	0.0748356691578710	0.4053748615460732	T	T	T
0.4279056008104578	0.0751377046191156	0.4058978721049176	T	T	T
0.5713017891347671	0.0752049491595851	0.4067018322025651	T	T	T
0.7147444297449843	0.0754587089864837	0.4064081508982528	T	T	T
0.8574365370534606	0.0752892336301313	0.4058039015863528	T	T	T
0.9998113630288262	0.0750601314878391	0.4060241022633723	T	T	T
0.1418355543547953	0.3256807374464097	0.4062145532421011	T	T	T
0.2836263983195226	0.3250201204244851	0.4055336804002282	T	T	T
0.4274342361322392	0.3258079839779119	0.4078849428680430	T	T	T
0.5700027504678240	0.3258843274416536	0.4100539078699856	T	T	T
0.7158631663222302	0.3240742690197415	0.4046999312140192	T	T	T
0.8585325357594735	0.3252657203991480	0.4052983154631083	T	T	T
0.0002101091752641	0.3254264850706935	0.4061159556412508	T	T	T
0.1425681435700659	0.5755659837767558	0.4070739644707391	T	T	T
0.2851194297035907	0.5751229320036474	0.4067354321523950	T	T	T
0.4278037927010232	0.5750245745832554	0.4068729195824732	T	T	T
0.5710888212266371	0.5764606755407947	0.4074480718975091	T	T	T
0.7153261179415714	0.5754855488180844	0.4063484216544814	T	T	T
0.8575017323108557	0.5755457608551057	0.4061988611441822	T	T	T
0.9997135808215261	0.5757429051482026	0.4066851521110062	T	T	T
0.1425214453058393	0.8251961847613123	0.4066505868336576	T	T	T
0.2852844029398475	0.8251620723968727	0.4061272485037898	T	T	T
0.4280197645713746	0.8250786459194781	0.4062976817629371	T	T	T
0.5714489904052992	0.8254316895699518	0.4068684227979312	T	T	T
0.7148664750348820	0.8255050814149579	0.4067326365808965	T	T	T
0.8572467840252143	0.8256563842464953	0.4062288052418735	T	T	T
0.9995690104468525	0.8253309953385721	0.4064897759157063	T	T	T
0.0712592232492100	0.1974159624679062	0.4335318461210688	T	T	T
0.2129530336534483	0.1972642188370134	0.4328269729916007	T	T	T
0.3562056419445319	0.1975899276731591	0.4336085991478585	T	T	T
0.4999611102382533	0.1976207817624246	0.4347126093938007	T	T	T
0.6421590498368518	0.1982006430836638	0.4351439193506246	T	T	T
0.7866584422107218	0.1974718968370556	0.4333809985012234	T	T	T
0.9290915510264288	0.1974695815608302	0.4330873786106418	T	T	T
0.0710827424566003	0.4479594329404851	0.4349398379325308	T	T	T
0.2136101887898125	0.4479454935626982	0.4348149666679426	T	T	T
0.3558867214172017	0.4477322009460180	0.4349932482812844	T	T	T
0.4991396710008527	0.4471400446153586	0.4386086823235821	T	T	T
0.6440715187945629	0.4488161463399823	0.4350286682125121	T	T	T
0.7865831136617906	0.4478668758797563	0.4337221282693484	T	T	T
0.9287039719754462	0.4477044940974950	0.4341879174102594	T	T	T
0.0710662539320858	0.6978511288052865	0.4346612817711306	T	T	T
0.2139907368105617	0.6977590607594186	0.4344547747678182	T	T	T
0.3565993598452739	0.6977908654014445	0.4341276322599758	T	T	T
0.4993500051981471	0.6982612583456838	0.4348931344536586	T	T	T
0.6434578381361672	0.6989911308906008	0.4347752818287512	T	T	T
0.7862154643471267	0.6981362736771868	0.4341931272782279	T	T	T
0.9283459645733921	0.6977939668197877	0.4341418476703908	T	T	T
0.0710783267940848	0.9473121611973350	0.4342395857007716	T	T	T
0.2139739085703790	0.9472105730009286	0.4338213922602832	T	T	T
0.3566624118186708	0.9473919148574670	0.4338219989834186	T	T	T
0.4996053193090326	0.9479083188853481	0.4345465764699482	T	T	T

0.6431988468924973	0.9480795911610236	0.4348871940574869	T	T	T
0.7861692588286550	0.9479108799569660	0.4341897510341646	T	T	T
0.9284334338821483	0.9475110224049640	0.4341234864270258	T	T	T
0.9999714945305888	0.1615828508925405	0.5651804855069527	T	T	T
0.1427670557987085	0.1612643623207484	0.5649071033671090	T	T	T
0.2847332395122064	0.1612309720623230	0.5647437005182224	T	T	T
0.4274541631521764	0.1602484031667178	0.5651092630096129	T	T	T
0.5711426089839302	0.1603297457572659	0.5656957935939948	T	T	T
0.7148288752668456	0.1613226267508981	0.5650635526287112	T	T	T
0.8576431942785682	0.1618716039922757	0.5652697099448618	T	T	T
0.9996300015709171	0.4117080586313939	0.5657995600402810	T	T	T
0.1422634721975199	0.4116929557410491	0.5661757945027559	T	T	T
0.2840541652796040	0.4111286933381455	0.5664535262397730	T	T	T
0.4223046819208558	0.4120887130207025	0.5675695437561674	T	T	T
0.5773296065897515	0.4138313862926126	0.5586323477722471	T	T	T
0.7166351276725742	0.4109671668097225	0.5650787705661647	T	T	T
0.8576106162242088	0.4113999550144222	0.5655153382343263	T	T	T
0.9995413858885638	0.6617767544204619	0.5657658489595349	T	T	T
0.1425686664466417	0.6616398589356067	0.5658044567672003	T	T	T
0.2853740322825173	0.6618888060470051	0.5658793635274223	T	T	T
0.4276592146848041	0.6619643561979777	0.5661910915721797	T	T	T
0.5714249719688753	0.6615229274501783	0.5647025866572798	T	T	T
0.7153099892858584	0.6619182287696677	0.5655495520205233	T	T	T
0.8574695895763395	0.6622735419383867	0.5661736935063971	T	T	T
0.9997926619576867	0.9116073938629172	0.5655749716306948	T	T	T
0.1426135301684190	0.9112322233339625	0.5654378193278761	T	T	T
0.2852925079809545	0.9114881635038273	0.5655241837853197	T	T	T
0.4278683723175378	0.9113320425768101	0.5654686141315642	T	T	T
0.5711358946911981	0.9111480236388245	0.5652556893274019	T	T	T
0.7147404926201589	0.9116407471758250	0.5655699242827691	T	T	T
0.8574613002273128	0.9119980334591903	0.5661617076656065	T	T	T
0.0713352121921841	0.0338579453271032	0.5930854239548998	T	T	T
0.2139116377670877	0.0337715908710054	0.5929237025897008	T	T	T
0.3561983374609091	0.0338925144110882	0.5930035752575710	T	T	T
0.4991558695320381	0.0335697378452041	0.5932380220901183	T	T	T
0.6430216651486124	0.0338906664028696	0.5931405329712242	T	T	T
0.7863769174964028	0.0341808011547893	0.5935394980947620	T	T	T
0.9287001037112986	0.0341626908207567	0.5934299390084311	T	T	T
0.0712519751503351	0.2835295182255913	0.5933256870688820	T	T	T
0.2134127047974734	0.2835073465827925	0.5933225162756106	T	T	T
0.3553229093810734	0.2827255986850724	0.5927056680651567	T	T	T
0.4977029314394992	0.2783180992981053	0.5967826814299071	T	T	T
0.6440047480951724	0.2845022633849841	0.5934028109749879	T	T	T
0.7868672081227963	0.2837228473642930	0.5925896904393591	T	T	T
0.9288522553874129	0.2836636329090437	0.5931972293113730	T	T	T
0.0709020588343649	0.5341004131673003	0.5937591303208126	T	T	T
0.2136913945374102	0.5341958432847255	0.5942479433667651	T	T	T
0.3560465181054035	0.5352074440574283	0.5945492290739253	T	T	T
0.4994645744122919	0.5344144289043727	0.5944064737107762	T	T	T
0.6442583501592682	0.5361720753287070	0.5935111821528570	T	T	T
0.7869729283933331	0.5345069787984984	0.5940374608175999	T	T	T
0.9284011537952344	0.5341887317056224	0.5938714585603283	T	T	T
0.0711617803778443	0.7836910634052671	0.5935253948257203	T	T	T
0.2139339166215243	0.7838458550118799	0.5937109164560725	T	T	T

```
  0.3564232317976722  0.7843415407403470  0.5937909643357115   T   T   T
  0.4993833628680610  0.7840215101260063  0.5934878638678815   T   T   T
  0.6432095314726980  0.7843055260137731  0.5934780946162088   T   T   T
  0.7864546767776451  0.7844875230381732  0.5940099955499831   T   T   T
  0.9285790459401789  0.7841938029911892  0.5938915891224553   T   T   T
  0.5917406135116730  0.4018909547988633  0.6860306793821801   T   T   T
  0.5041061528649182  0.3789919632180214  0.6303814515792859   T   T   T
== Te adatom bound to Pd2
   1.00000000000000
    24.6586885454000111    0.0000000000000000    0.0000000000000000
     0.0000000000000000   25.2576961516000082    0.0000000000000000
     0.0000000000000000    0.0000000000000000   22.0000000000007034
   W    Te   Pd
    56   113     2
Selective dynamics
Direct
  0.0716250222769121  0.0988575036348045  0.4946753009405243   T   T   T
  0.2137624375524975  0.0986806717927864  0.4947062519125140   T   T   T
  0.3557553683698688  0.0987025816995856  0.4947817740129797   T   T   T
  0.4991935422824412  0.0986174817645853  0.4952936481785344   T   T   T
  0.6436522933708972  0.0986888337817038  0.4951155705926242   T   T   T
  0.7872385254512002  0.0988007495472306  0.4943499667734864   T   T   T
  0.9295038862331124  0.0987967638294884  0.4943331736741248   T   T   T
  0.0715415418389017  0.3495492254382884  0.4957873204266879   T   T   T
  0.2127451212695787  0.3492959176964110  0.4957222315978671   T   T   T
  0.3538087266673290  0.3481056511890397  0.4953702046651169   T   T   T
  0.4955859833820833  0.3475715967660522  0.4989281045423813   T   T   T
  0.6473728132764190  0.3476040678387279  0.4984672692840856   T   T   T
  0.7891390715852998  0.3481196736897004  0.4942447622558175   T   T   T
  0.9302399206014271  0.3492686289602238  0.4947403562187662   T   T   T
  0.0712735372819530  0.5994703786350627  0.4958072102620661   T   T   T
  0.2140155167946935  0.5994383090717640  0.4959607500248274   T   T   T
  0.3555264315375715  0.5997021319349094  0.4956319264524485   T   T   T
  0.4974783094883818  0.6001766181381609  0.4955088914875543   T   T   T
  0.6453314841325286  0.6001462118885207  0.4951330979859074   T   T   T
  0.7871837669844213  0.5997502304459941  0.4950605226573196   T   T   T
  0.9285786487636287  0.5995592827191368  0.4955328272576917   T   T   T
  0.0713841236486387  0.8489523197803261  0.4953182889700962   T   T   T
  0.2139104211750979  0.8490175537428207  0.4953640614836046   T   T   T
  0.3558857692635309  0.8492625926500572  0.4952118444346302   T   T   T
  0.4990440639891280  0.8491632289601110  0.4953083740319004   T   T   T
  0.6437128732742423  0.8492253261110326  0.4953334021324492   T   T   T
  0.7868108625959375  0.8494954155427822  0.4954086720045235   T   T   T
  0.9288534917308804  0.8492701564630897  0.4954104576476641   T   T   T
  0.0003125673967160  0.0096538245921707  0.5033345812639003   T   T   T
  0.1425711596066370  0.0095445204273673  0.5033986420035202   T   T   T
  0.2846692066524519  0.0096282698238656  0.5035418798420935   T   T   T
  0.4271651557950124  0.0099134179315445  0.5040663080863916   T   T   T
  0.5713408170295927  0.0099945767877842  0.5045483539586779   T   T   T
  0.7155981767704240  0.0100671630732161  0.5040692897414166   T   T   T
  0.8583097417252148  0.0098909258607745  0.5034976499314789   T   T   T
  0.0008017904902005  0.2598776903475015  0.5031766731570886   T   T   T
  0.1424285080491506  0.2598560035232141  0.5036768381722816   T   T   T
  0.2837111059986070  0.2593575686275136  0.5035420330764870   T   T   T
```

0.4279789679386685	0.2587805444278103	0.5045954555588984	T	T	T
0.5715146081858994	0.2601658787163796	0.5074095760940824	T	T	T
0.7149069510710567	0.2588559750818596	0.5037559144012199	T	T	T
0.8594560853459670	0.2594039423173408	0.5025436083775485	T	T	T
0.9999200974872974	0.5103441225770665	0.5042144747701598	T	T	T
0.1427210605823264	0.5102944383941631	0.5046899193152278	T	T	T
0.2846724876149788	0.5102142085292998	0.5047671107963674	T	T	T
0.4258942340076620	0.5109406552594224	0.5052882426012261	T	T	T
0.5712622519282667	0.5135109298694724	0.5053626589776306	T	T	T
0.7168766089140298	0.5109437543786333	0.5046101159266509	T	T	T
0.8580406753317675	0.5101760710282490	0.5039360063626407	T	T	T
0.9998523063768152	0.7601520627032886	0.5041739898746727	T	T	T
0.1426874563238622	0.7600058209781736	0.5042630202612191	T	T	T
0.2848040213806720	0.7601818965155391	0.5041332380500287	T	T	T
0.4270222002139039	0.7605113374085565	0.5044005682374295	T	T	T
0.5712696903838169	0.7605875842769704	0.5045573794056057	T	T	T
0.7156814233921110	0.7606076090926704	0.5044559114672185	T	T	T
0.8577638437782382	0.7603814388674236	0.5041395266503578	T	T	T
0.1428414367229272	0.0748163609087785	0.4053893573689679	T	T	T
0.2851047320013430	0.0748370712671542	0.4053505079350357	T	T	T
0.4279365935976865	0.0751514614275584	0.4060394913526890	T	T	T
0.5713762263723264	0.0751031977380217	0.4067446197612870	T	T	T
0.7149715376507456	0.0750628683682028	0.4058748632313872	T	T	T
0.8581735424039302	0.0747165239286479	0.4050663250337682	T	T	T
0.0007009502917234	0.0747420219448950	0.4052477355279128	T	T	T
0.1420785659194000	0.3255082446007369	0.4059935221976593	T	T	T
0.2835125880117210	0.3246750108834348	0.4057071779269021	T	T	T
0.4273389434422702	0.3251852799463800	0.4074037405763447	T	T	T
0.5713615647115157	0.3265006092715907	0.4117216944580316	T	T	T
0.7153149076799612	0.3252644299697443	0.4064943893016496	T	T	T
0.8594890989113567	0.3247929666171493	0.4046726441403552	T	T	T
0.0012692816407576	0.3254599654350832	0.4054999984130984	T	T	T
0.1426475000888235	0.5755002641492202	0.4067110357356299	T	T	T
0.2847468820067522	0.5751745217873463	0.4063550144314473	T	T	T
0.4270581771528431	0.5755260805533686	0.4064191881408277	T	T	T
0.5713144056374693	0.5770512364082100	0.4072856616033240	T	T	T
0.7156168572209274	0.5755120393149772	0.4057680214024562	T	T	T
0.8580493334157850	0.5753808894926015	0.4057935613449078	T	T	T
0.0001558342612010	0.5758645579188284	0.4064464640064334	T	T	T
0.1427832771661275	0.8249794814359253	0.4061271423629315	T	T	T
0.2850683155573848	0.8251854863926674	0.4057923772388258	T	T	T
0.4278037292405359	0.8253988192514307	0.4062394990171145	T	T	T
0.5714503798122365	0.8255426900812767	0.4067669420371811	T	T	T
0.7150884884947363	0.8254616681451176	0.4062772636764520	T	T	T
0.8576437053206943	0.8256662175678975	0.4059950865313363	T	T	T
0.0000226119596105	0.8252951826943080	0.4061410905461130	T	T	T
0.0718266151472337	0.1971674092335343	0.4329282839403008	T	T	T
0.2131002077884742	0.1971484990182933	0.4328952648244176	T	T	T
0.3561193516068619	0.1975641388692214	0.4338558477594836	T	T	T
0.5003658399246552	0.1978824856914299	0.4352710734861220	T	T	T
0.6423792578429283	0.1978157123463999	0.4348023422037394	T	T	T
0.7870020639027203	0.1975123523458319	0.4332418362147845	T	T	T
0.9303122810559590	0.1970407247482018	0.4321493798624343	T	T	T
0.0715176659544872	0.4477606447195766	0.4346325670704596	T	T	T

0.2134932318592175	0.4476801157609634	0.4345489697169132	T	T	T
0.3555524548134893	0.4476311732187772	0.4347158820233384	T	T	T
0.4982572114099930	0.4483922295290180	0.4376577579403818	T	T	T
0.6442680651773710	0.4484565869832074	0.4373417504611486	T	T	T
0.7872462617935938	0.4477206432589493	0.4337295209567636	T	T	T
0.9293256370263456	0.4477836088630921	0.4335489062342637	T	T	T
0.0712771379521082	0.6977599148911000	0.4343197685813557	T	T	T
0.2139091818080304	0.6976495636124453	0.4341962516463758	T	T	T
0.3561963333812189	0.6981559723308027	0.4339313665102508	T	T	T
0.4991384471467181	0.6988167900851514	0.4346212095646322	T	T	T
0.6436170828654089	0.6990243124159935	0.4346574442733840	T	T	T
0.7864705163462721	0.6984510541845746	0.4338052164221922	T	T	T
0.9286447262350908	0.6979399454927127	0.4339893950572329	T	T	T
0.0714788262850080	0.9470206730477349	0.4332490412491471	T	T	T
0.2139741406722860	0.9470500497994141	0.4334257946304019	T	T	T
0.3565004165539152	0.9476063459485008	0.4336382307008407	T	T	T
0.4995697452100664	0.9481442617682269	0.4346311819021020	T	T	T
0.6432490068903042	0.9480485997448491	0.4347346372647191	T	T	T
0.7862919344484209	0.9478364561647847	0.4337271177435396	T	T	T
0.9289002481242806	0.9472530183469832	0.4332262477557542	T	T	T
0.0004543192054046	0.1614355586022708	0.5646671812322596	T	T	T
0.1428115828977533	0.1612382768401285	0.5649264626446220	T	T	T
0.2846778844182430	0.1611325234588608	0.5650286723692273	T	T	T
0.4274736984508049	0.1600931903340683	0.5651917068765118	T	T	T
0.5714808324395499	0.1598729392689215	0.5657201174567219	T	T	T
0.7155852085644422	0.1604309229775049	0.5647427210388595	T	T	T
0.8584638994847086	0.1613144130365883	0.5641967033439990	T	T	T
0.0000965261631347	0.4117989017879929	0.5655808115975856	T	T	T
0.1425596214676117	0.4118607566751004	0.5663417413754477	T	T	T
0.2841252616762238	0.4112304643674444	0.5661966677617570	T	T	T
0.4219064326907990	0.4127064233692987	0.5673640803382977	T	T	T
0.5715644419136278	0.4144288911002751	0.5551822008902495	T	T	T
0.7212851244538169	0.4128121119671981	0.5664254470177766	T	T	T
0.8587835170065120	0.4111913166667343	0.5651721063039810	T	T	T
0.9998089763304824	0.6617369793881388	0.5655474261978263	T	T	T
0.1426100221437454	0.6617433548834833	0.5658070353877385	T	T	T
0.2849882299458763	0.6621009622718750	0.5659447707979973	T	T	T
0.4271066156378953	0.6622254363986499	0.5658484157285933	T	T	T
0.5714545363032448	0.6614817134863531	0.5639730265863111	T	T	T
0.7156698829201031	0.6620278201513625	0.5654520415449834	T	T	T
0.8576067402653567	0.6621935356241171	0.5657817882278668	T	T	T
0.0000745189348479	0.9115986676420770	0.5652060430883090	T	T	T
0.1425971683678120	0.9112907678783593	0.5653267518983381	T	T	T
0.2850813458342729	0.9114896511505942	0.5654100277106978	T	T	T
0.4276948846302033	0.9112985676389845	0.5652939570214494	T	T	T
0.5713095276436072	0.9110336491974340	0.5650535579999769	T	T	T
0.7149580888044131	0.9115061929220398	0.5653476083566529	T	T	T
0.8577009195070397	0.9118128084151276	0.5655018306538990	T	T	T
0.0714035256181799	0.0335172375739290	0.5926647529981023	T	T	T
0.2135584236846634	0.0334302032641636	0.5927847236730605	T	T	T
0.3558957136413097	0.0337882647433532	0.5930012258357539	T	T	T
0.4992282520363555	0.0335322587146520	0.5931272958663606	T	T	T
0.6434727732752307	0.0337460063381242	0.5930332937407594	T	T	T
0.7869793714460064	0.0342105977155616	0.5928046600753690	T	T	T

```
  0.9292972938412499  0.0340261009936172  0.5927542671863715   T   T   T
  0.0713739237915354  0.2834374388796413  0.5931803488193664   T   T   T
  0.2133368612143428  0.2834837437864257  0.5933869729502227   T   T   T
  0.3553094873076465  0.2830059443971704  0.5929446769406493   T   T   T
  0.4980532331022818  0.2794650370591249  0.5963144033878507   T   T   T
  0.6452773460714497  0.2794653364240756  0.5958729223642053   T   T   T
  0.7878557199119056  0.2832225981289725  0.5918492679865944   T   T   T
  0.9296323301126193  0.2836684801845779  0.5925342472999392   T   T   T
  0.0711615012568144  0.5340788273394302  0.5936508822693028   T   T   T
  0.2135645358774453  0.5341955123823602  0.5941468901126481   T   T   T
  0.3552265894746433  0.5356310095312683  0.5945385834752226   T   T   T
  0.4985425109364680  0.5355301176379755  0.5936319342056491   T   T   T
  0.6443056412873905  0.5355614725289117  0.5932317516937179   T   T   T
  0.7875754048581678  0.5354934248669468  0.5937644215604381   T   T   T
  0.9289453303846247  0.5341298224420300  0.5934858287901241   T   T   T
  0.0712292709647172  0.7837088696227125  0.5934470783613238   T   T   T
  0.2137022776156493  0.7839236928477966  0.5936374000322702   T   T   T
  0.3559292157911239  0.7844944877535627  0.5935910956902281   T   T   T
  0.4992291420858851  0.7840707382843176  0.5932943829867039   T   T   T
  0.6433329075113724  0.7842485436893312  0.5932356660794676   T   T   T
  0.7866122047420935  0.7845601176931839  0.5936932925213259   T   T   T
  0.9288528591703864  0.7841701040004571  0.5936188976863852   T   T   T
  0.5721670388508004  0.4021451515351541  0.7157727625677929   T   T   T
  0.5040173643325921  0.3802686949180333  0.6330558774961763   T   T   T
  0.6396729513327896  0.3803494035941552  0.6324765025777116   T   T   T
== Te adatom bound to Pd3
   1.00000000000000
     24.6586885454000111    0.0000000000000000    0.0000000000000000
      0.0000000000000000   25.2576961516000082    0.0000000000000000
      0.0000000000000000    0.0000000000000000   22.0000000000007034
   W    Te   Pd
    56   113     3
Selective dynamics
Direct
  0.0717111122401160  0.0990924307121142  0.4943160327504275   T   T   T
  0.2134358198089571  0.0990218751938489  0.4947985443747650   T   T   T
  0.3555430892905390  0.0989315290941677  0.4950524615417068   T   T   T
  0.4989895349159367  0.0988493139345638  0.4952660122231540   T   T   T
  0.6438419506458910  0.0989072684362549  0.4949571522779280   T   T   T
  0.7874527678423359  0.0988641773668418  0.4944187971615837   T   T   T
  0.9299516694272105  0.0989581376587968  0.4942062994955759   T   T   T
  0.0715371178053479  0.3497419573933255  0.4959469076015877   T   T   T
  0.2125305475421611  0.3495789329830288  0.4962373007142349   T   T   T
  0.3535748006850940  0.3484726293609071  0.4963172479811709   T   T   T
  0.4923728865307383  0.3473135727173408  0.4980887684335595   T   T   T
  0.6503441146085472  0.3472315950644811  0.4977456755948000   T   T   T
  0.7890823638725774  0.3482460251549269  0.4956699506832441   T   T   T
  0.9305594456099145  0.3493890667631575  0.4955213754738581   T   T   T
  0.0714034373136238  0.5996017374926308  0.4958552564838228   T   T   T
  0.2137714346105112  0.5996629354570437  0.4964247098333027   T   T   T
  0.3554664432791124  0.5999921608772542  0.4967678771402235   T   T   T
  0.4977355363008452  0.6009298395599199  0.4969896999259210   T   T   T
  0.6448205360274708  0.6007775553658704  0.4969647477502658   T   T   T
  0.7871908155728387  0.5997598414119675  0.4965668054284797   T   T   T
```

0.9289217382735508	0.5995840800230250	0.4960244424951694	T	T	T
0.0715319813772288	0.8491485618094218	0.4948175122913966	T	T	T
0.2136754108556873	0.8493024808086916	0.4951895139053402	T	T	T
0.3557639103378682	0.8497021887211549	0.4952145613979761	T	T	T
0.4991096196394905	0.8496995653913291	0.4948120548349672	T	T	T
0.6437006879944398	0.8496636618852634	0.4947131634024124	T	T	T
0.7870759695072247	0.8496324560024833	0.4949689293463553	T	T	T
0.9294039510494106	0.8493288320652982	0.4949523422850148	T	T	T
0.0007286434897863	0.0098027521176682	0.5029433248075347	T	T	T
0.1425734736837992	0.0097911340931817	0.5031787370301142	T	T	T
0.2843127920266899	0.0100035533227979	0.5037800693136628	T	T	T
0.4270568720242288	0.0102094300470089	0.5040533703678470	T	T	T
0.5714157695594636	0.0103943560451321	0.5042869432377103	T	T	T
0.7157336559313076	0.0102648397010862	0.5037253342637411	T	T	T
0.8586076791479692	0.0099688241013765	0.5033446015308374	T	T	T
0.0009520615403856	0.2600209611479098	0.5032332319447479	T	T	T
0.1423541492983781	0.2601397918506323	0.5036827706069300	T	T	T
0.2836239006958858	0.2596385407057076	0.5040244683322141	T	T	T
0.4270302844499592	0.2587574587408374	0.5050771723466388	T	T	T
0.5711260859137374	0.2600502154750616	0.5074326156089352	T	T	T
0.7158577584999207	0.2586841261729070	0.5045474360130373	T	T	T
0.8593443850578762	0.2594592935608747	0.5032863546359201	T	T	T
0.0002484319992427	0.5104529838162085	0.5047400806552098	T	T	T
0.1425469433472615	0.5104333371393359	0.5049572445194148	T	T	T
0.2845883758461581	0.5105072851357373	0.5057439569104114	T	T	T
0.4259837532581561	0.5112616045594811	0.5072560102495884	T	T	T
0.5714236688557131	0.5135840219493458	0.5077926288561372	T	T	T
0.7166039106345837	0.5110787263515194	0.5070848001724049	T	T	T
0.8580410954390927	0.5102826467284511	0.5052097639559970	T	T	T
0.0001915678109840	0.7601628484244242	0.5038659419538548	T	T	T
0.1425452965137623	0.7602416241650604	0.5040729831447593	T	T	T
0.2846293061736483	0.7604839090811026	0.5043326060176548	T	T	T
0.4269892189975285	0.7611983204881133	0.5044508917165001	T	T	T
0.5713145063996885	0.7612560863164962	0.5044057590134085	T	T	T
0.7157171714951704	0.7609973814000568	0.5044681940722403	T	T	T
0.8581449857025678	0.7604777129324629	0.5041013134301551	T	T	T
0.1428933782381747	0.0749528310425649	0.4051742581649920	T	T	T
0.2848568578806358	0.0750981653339499	0.4055806540663714	T	T	T
0.4276646792351829	0.0755602688973152	0.4061671138993824	T	T	T
0.5712929365832047	0.0756205538961837	0.4066095134300053	T	T	T
0.7151739645196152	0.0757036327999355	0.4057497148871413	T	T	T
0.8584471714366788	0.0748825577364183	0.4051388122567184	T	T	T
0.0008367955632928	0.0750239535464907	0.4048116960841038	T	T	T
0.1421617369729466	0.3259492204347090	0.4061437915460642	T	T	T
0.2834048838646521	0.3252136305995090	0.4064317477008012	T	T	T
0.4253741948792968	0.3242272494671599	0.4066810896159553	T	T	T
0.5712287807059534	0.3265174510573348	0.4128969638404030	T	T	T
0.7170802499836677	0.3241221162798785	0.4060974332629261	T	T	T
0.8593860759510769	0.3251809095556606	0.4058160084387296	T	T	T
0.0011557899769790	0.3257768161784281	0.4056650613961838	T	T	T
0.1428495429684807	0.5754828918532946	0.4068832753990141	T	T	T
0.2850614796636286	0.5754969858372321	0.4073496923741804	T	T	T
0.4272966337366461	0.5753060628984655	0.4080341319719825	T	T	T
0.5712619315537353	0.5763178941127653	0.4092138171215934	T	T	T

0.7152535772777628	0.5751495054151963	0.4079741248933821	T	T	T
0.8576407758339040	0.5754246833498678	0.4069712187051506	T	T	T
0.0000806911133832	0.5757512257527716	0.4066729632040844	T	T	T
0.1429095600228583	0.8250621203352905	0.4057355668853679	T	T	T
0.2850014134334370	0.8253535856937718	0.4059104523822065	T	T	T
0.4275488568390498	0.8257963575020241	0.4060592003404210	T	T	T
0.5714118403268890	0.8259103626159133	0.4062127469266720	T	T	T
0.7152342417762831	0.8254416611069942	0.4060270254579239	T	T	T
0.8580627778420405	0.8253884303958338	0.4057396478262059	T	T	T
0.0003867604614730	0.8251632348527077	0.4056093493774068	T	T	T
0.0719396894976477	0.1975069915583275	0.4326727972481089	T	T	T
0.2130406367373520	0.1974608462880591	0.4329973549843604	T	T	T
0.3555519313458572	0.1976538035462189	0.4341969505494283	T	T	T
0.4997619730070333	0.1980833114908531	0.4353261551464490	T	T	T
0.6427129776236656	0.1982191702941827	0.4352811738392797	T	T	T
0.7873865943997642	0.1975552070357063	0.4334573982666616	T	T	T
0.9302408856511717	0.1975195424253740	0.4323828909035975	T	T	T
0.0714853695179310	0.4478758203538895	0.4347711998840758	T	T	T
0.2136212749983608	0.4479541753746371	0.4351381725806511	T	T	T
0.3558772627261120	0.4480870348484077	0.4363451366625690	T	T	T
0.4973266061704993	0.4498282595369986	0.4379315610776275	T	T	T
0.6453310133502893	0.4498032711107275	0.4376158945726352	T	T	T
0.7867659609802288	0.4480102852304257	0.4359546299085107	T	T	T
0.9292944931950629	0.4479502234503542	0.4347544623036728	T	T	T
0.0714922880300524	0.6977563288138422	0.4340019338525669	T	T	T
0.2139385598123986	0.6977991142476142	0.4344004879914701	T	T	T
0.3561994259862302	0.6984119218580951	0.4345499439844801	T	T	T
0.4989592701568495	0.6991447386077443	0.4349528864221173	T	T	T
0.6435342409928761	0.6991026382926904	0.4349992050568126	T	T	T
0.7864609983177872	0.6982580644245240	0.4344851263221894	T	T	T
0.9288178863340638	0.6978396608722781	0.4340133604217984	T	T	T
0.0717014616663936	0.9472885076563088	0.4327001929797859	T	T	T
0.2140122883591008	0.9473396670539438	0.4332497669691779	T	T	T
0.3562872822419441	0.9481976368506402	0.4336338627357385	T	T	T
0.4994908790529492	0.9486601870032199	0.4343206711358668	T	T	T
0.6432209896229386	0.9488013219330937	0.4340045057676080	T	T	T
0.7864896459463466	0.9480852151581318	0.4333341535833502	T	T	T
0.9291543718052369	0.9474404784083015	0.4329158818646145	T	T	T
0.0007238506641123	0.1615895574134329	0.5645516147107310	T	T	T
0.1424642799198594	0.1616293089794729	0.5648562563484449	T	T	T
0.2844495077388495	0.1613530413976519	0.5652409280029362	T	T	T
0.4273831780960454	0.1601549119869252	0.5657968232382815	T	T	T
0.5714802867354003	0.1600599583717769	0.5655438224126301	T	T	T
0.7156786529921459	0.1601453580928668	0.5651645425691073	T	T	T
0.8587328666151665	0.1610888282268480	0.5645260302665031	T	T	T
0.0004202581400823	0.4119376945553120	0.5663824183870531	T	T	T
0.1423435178145432	0.4121048289079317	0.5665924997017963	T	T	T
0.2840275139935723	0.4114254243823835	0.5672187488991077	T	T	T
0.4227814879765938	0.4122829377338191	0.5691149538323192	T	T	T
0.5714773134905695	0.4115186935211176	0.5482765489651530	T	T	T
0.7200076752956220	0.4120051262477228	0.5687078354439382	T	T	T
0.8588674327206229	0.4110382997105301	0.5663963646281104	T	T	T
0.0000584210291096	0.6619783253993698	0.5656228932909100	T	T	T
0.1424189051693879	0.6622567040460179	0.5659853141272636	T	T	T

```
  0.2847031565644357  0.6624997995660609  0.5665366341631243   T   T   T
  0.4269609525609775  0.6631178131926015  0.5662488194234080   T   T   T
  0.5712787448589070  0.6626277624416939  0.5647474644100619   T   T   T
  0.7156050597010243  0.6627463490557631  0.5661406809150751   T   T   T
  0.8578046030417443  0.6624839378864523  0.5662808275750928   T   T   T
  0.0004554398902434  0.9116965404367096  0.5648855540699983   T   T   T
  0.1424946634593904  0.9116797594289324  0.5649405445302190   T   T   T
  0.2848143338432460  0.9117366065926797  0.5653442223798607   T   T   T
  0.4278116299837603  0.9116613297758573  0.5649481840668994   T   T   T
  0.5715237477476999  0.9114547361623534  0.5643826623044372   T   T   T
  0.7150868152048561  0.9116066875041383  0.5647910305497451   T   T   T
  0.8582032644635357  0.9118639666552131  0.5649798895479469   T   T   T
  0.0715737241652606  0.0339684685883938  0.5923766704259178   T   T   T
  0.2132625523171154  0.0339342867627384  0.5929854905660186   T   T   T
  0.3558197675201100  0.0338543489857203  0.5931573579344026   T   T   T
  0.4992930371580349  0.0336809150195093  0.5929879241734832   T   T   T
  0.6436067543808511  0.0338860729134142  0.5927580622401413   T   T   T
  0.7871335521323850  0.0340187355682470  0.5926221669686315   T   T   T
  0.9296668471388579  0.0340773670497262  0.5924637834560497   T   T   T
  0.0714104094058933  0.2835499370791558  0.5932956989822704   T   T   T
  0.2130493577566311  0.2835174178614366  0.5936692860799538   T   T   T
  0.3548569762023158  0.2832041172584056  0.5937266985764642   T   T   T
  0.4979618671969013  0.2801004979053671  0.5963007526916334   T   T   T
  0.6447821825582045  0.2800145648039023  0.5957834116289386   T   T   T
  0.7881680291757405  0.2828899630726668  0.5930728928608050   T   T   T
  0.9299610711505932  0.2834616996569308  0.5929216374628102   T   T   T
  0.0713333042274400  0.5346261374929278  0.5940779377319142   T   T   T
  0.2131024587533386  0.5345871179635266  0.5946840709909995   T   T   T
  0.3544000551707238  0.5362385359686567  0.5958546244026033   T   T   T
  0.4960048695301684  0.5390551737799827  0.5973385704208360   T   T   T
  0.6467734730475807  0.5388725955000953  0.5973844333624865   T   T   T
  0.7883352844350110  0.5357152262826409  0.5955764154536295   T   T   T
  0.9294394258334433  0.5344492608727128  0.5942404952766704   T   T   T
  0.0712387136180043  0.7843180899594352  0.5931992346513322   T   T   T
  0.2135145827064587  0.7845015926919647  0.5935942177799741   T   T   T
  0.3558563262495435  0.7849215888538211  0.5936416983514203   T   T   T
  0.4993650026644482  0.7851874773656498  0.5930962071751810   T   T   T
  0.6433557439208470  0.7851281248235074  0.5930218496597347   T   T   T
  0.7869493664617192  0.7850323711505559  0.5934709761908752   T   T   T
  0.9291899928265145  0.7844851335955425  0.5933258901724804   T   T   T
  0.5717655075703728  0.4004287853030633  0.7242728099107619   T   T   T
  0.5076209133113955  0.3799525664116097  0.6338010537208613   T   T   T
  0.6354018077956466  0.3798112698188469  0.6336287722857146   T   T   T
  0.5712521311168774  0.4747361168428006  0.6420463618935996   T   T   T
== On-top cluster Pd05-Te11
    1.0000000000000000
    31.7040281100000030    0.0000000000000000    0.0000000000000000
     0.0000000000000000   31.5721201999999970    0.0000000000000000
     0.0000000000000000    0.0000000000000000   22.0000000000000000
   W    Te   Te   Pd
    90   180    11     5
Selective dynamics
Direct
  0.0547934805196575  0.0780744436570090  0.4921840920657118   T   T   T
```

0.0539137249973355	0.2779170928048512	0.4952632749567784	T	T	T
0.0554755413911918	0.4786225415453125	0.4925997295395739	T	T	T
0.0552575222827449	0.6785493579017378	0.4911635513737571	T	T	T
0.0552439433303783	0.8784486018952370	0.4916324374001745	T	T	T
0.1664489989923370	0.0779415392831519	0.4919628653602762	T	T	T
0.1673765348678836	0.2774321809470090	0.4982990340184455	T	T	T
0.1668529064908784	0.4793512258992607	0.4912325475648272	T	T	T
0.1664801076895621	0.6789238216060814	0.4898141915488768	T	T	T
0.1664290384741245	0.8784767103078553	0.4905613731854623	T	T	T
0.2777753938465283	0.0777067111091495	0.4914461165565818	T	T	T
0.2776129661708676	0.2774043359511444	0.4958775539142329	T	T	T
0.2775443474540546	0.4800920944986812	0.4894968719362184	T	T	T
0.2778352099025357	0.6792363115524171	0.4892705273067622	T	T	T
0.2777011151900679	0.8784512938588424	0.4898584161352765	T	T	T
0.3891665549103174	0.0778956417804300	0.4915775977720677	T	T	T
0.3881003387981825	0.2773239666010873	0.4982512994332587	T	T	T
0.3885851748124817	0.4791103949614128	0.4910902144721465	T	T	T
0.3890727948577841	0.6789420087731377	0.4898942923670419	T	T	T
0.3891342199979437	0.8784619885185789	0.4899387717441941	T	T	T
0.5004682743217921	0.0780707814032295	0.4915341826545200	T	T	T
0.5016496984361198	0.2778000836700469	0.4951707364566048	T	T	T
0.4999248199048065	0.4785805462066859	0.4928925021123653	T	T	T
0.5002014054167210	0.6785354024950017	0.4909888497445650	T	T	T
0.5003256227015295	0.8782808705698085	0.4906570231787660	T	T	T
0.6113878814522250	0.0780892578548078	0.4921436986455634	T	T	T
0.6121347822698067	0.2782010254116287	0.4936668141527421	T	T	T
0.6113700710854990	0.4785506042976214	0.4935341251219297	T	T	T
0.6113915438060463	0.6783814007857514	0.4924529460558218	T	T	T
0.6113533931653744	0.8782814917604965	0.4918375189894115	T	T	T
0.7222620250185646	0.0781483835371889	0.4925968125979063	T	T	T
0.7225035523828491	0.2782670762354789	0.4930554107453524	T	T	T
0.7222877510751203	0.4785147187348514	0.4934881717241004	T	T	T
0.7222666139383259	0.6784579339973359	0.4934095618475475	T	T	T
0.7223599031194765	0.8782763051274413	0.4928290287615816	T	T	T
0.8331499781744012	0.0780870603999945	0.4927350034722359	T	T	T
0.8330207341103371	0.2783371052552188	0.4929191637499107	T	T	T
0.8331223576970298	0.4785479883084788	0.4932540871094927	T	T	T
0.8331952278400270	0.6784488333217215	0.4934737283723348	T	T	T
0.8332579489119295	0.8782758548932033	0.4932035665219406	T	T	T
0.9439380347787086	0.0781139735861159	0.4926290596148257	T	T	T
0.9433526641646813	0.2782716679349519	0.4932856764715711	T	T	T
0.9439054560057523	0.4785876597456208	0.4930797157338671	T	T	T
0.9441413652682257	0.6784045482905753	0.4926996532724861	T	T	T
0.9441042441111751	0.8782646977240264	0.4927101361832106	T	T	T
0.9994736314453575	0.0070167363826297	0.5019276615082108	T	T	T
0.9994008642880950	0.2068909288572598	0.5032647334479418	T	T	T
0.9989836953203418	0.4074361440182182	0.5033381332540965	T	T	T
0.9995975975602653	0.6073629785772420	0.5017034124614040	T	T	T
0.9997135389743043	0.8072589638948902	0.5018196712677190	T	T	T
0.1108104685287599	0.0070708546194467	0.5014251363585194	T	T	T
0.1107889751862866	0.2065317174651645	0.5035868951213173	T	T	T
0.1118858310699758	0.4080033861294892	0.5035257523912138	T	T	T
0.1109269433312661	0.6076781270452402	0.5003873778033987	T	T	T
0.1108133776968781	0.8074456533215377	0.5005599998554365	T	T	T

0.2221035308041529	0.0068826977191166	0.5007592772134009	T	T	T
0.2223842548847576	0.2061267262555644	0.5042839348536944	T	T	T
0.2231585292151750	0.4088023355619520	0.5020910973246312	T	T	T
0.2221281617597954	0.6083038653630528	0.4989134509869834	T	T	T
0.2220502726438933	0.8075227253833469	0.4996336508127285	T	T	T
0.3334250215431810	0.0068343824562393	0.5004103371664501	T	T	T
0.3330830756299585	0.2060788488987242	0.5043758677188334	T	T	T
0.3320327501863551	0.4087608362353771	0.5021938188746204	T	T	T
0.3334403939155590	0.6082405207999932	0.4991625868544617	T	T	T
0.3334497164025572	0.8075705856251628	0.4994871879829815	T	T	T
0.4447545530563151	0.0069633405390585	0.5005753312697099	T	T	T
0.4446150763574504	0.2065055205984278	0.5032199671394225	T	T	T
0.4438174036382090	0.4078629172135974	0.5035010759454183	T	T	T
0.4445028345212948	0.6076608680726486	0.5002265205026242	T	T	T
0.4447074830955073	0.8073830993652900	0.4999476302288802	T	T	T
0.5558656532446916	0.0069696534423262	0.5012547164776072	T	T	T
0.5560968331457263	0.2067882295786308	0.5032946085006933	T	T	T
0.5562997079198667	0.4073401433604245	0.5037997081805713	T	T	T
0.5557320053234180	0.6073994372404109	0.5017374618431987	T	T	T
0.5559145736832936	0.8071713713952552	0.5010530298629254	T	T	T
0.6667837048385610	0.0069663773428111	0.5018961027935064	T	T	T
0.6667895199992706	0.2069260645058832	0.5024569591340968	T	T	T
0.6670303724865939	0.4071548825690273	0.5030263296596668	T	T	T
0.6667702357839272	0.6072651134084182	0.5027499912143011	T	T	T
0.6667941633512582	0.8071758448601636	0.5021726392928599	T	T	T
0.7777009298741808	0.0069987137511572	0.5024902412897444	T	T	T
0.7777850199638341	0.2069092291821067	0.5020727109989721	T	T	T
0.7776250049483286	0.4071307899591404	0.5025991681165507	T	T	T
0.7777198750840875	0.6072542345537636	0.5030964636824281	T	T	T
0.7778100686520520	0.8071732884278442	0.5028607319801028	T	T	T
0.8885917826984211	0.0069860857257017	0.5025627099275674	T	T	T
0.8885361721723508	0.2069341665369614	0.5021798581408773	T	T	T
0.8883201164557454	0.4072089188713264	0.5025599766290144	T	T	T
0.8885536308294694	0.6072656439616989	0.5027533934838321	T	T	T
0.8886659391760255	0.8071323960409890	0.5028328212506616	T	T	T
0.9992025580859047	0.0586695981485289	0.4033775447997983	T	T	T
0.9997669271010640	0.2587409547336786	0.4049698301997292	T	T	T
0.9995238919816267	0.4585385094580811	0.4043518537772128	T	T	T
0.9992041429617793	0.6588632374043287	0.4031033663151047	T	T	T
0.9993497858007964	0.8588980590850211	0.4033353313899947	T	T	T
0.1104181855878273	0.0589466907957157	0.4032031482613199	T	T	T
0.1117378237397035	0.2596573593290209	0.4081203697708756	T	T	T
0.1107478899665008	0.4583215065312546	0.4038010750799357	T	T	T
0.1104261210947541	0.6591233099073481	0.4016820762125238	T	T	T
0.1104443047923624	0.8591641123633642	0.4021469859688566	T	T	T
0.2218398911172557	0.0588170899556514	0.4025963009575903	T	T	T
0.2218867944949049	0.2588415564803154	0.4074574536970151	T	T	T
0.2216811132458089	0.4589126236237211	0.4018879786815214	T	T	T
0.2220071963425549	0.6600019351551486	0.4005521235035711	T	T	T
0.2218098025053177	0.8592489264327857	0.4012346559456604	T	T	T
0.3335870127104323	0.0589227004298148	0.4024180390448643	T	T	T
0.3334178734666711	0.2587926428814078	0.4075924766958318	T	T	T
0.3334475882061093	0.4588189019486313	0.4019714448967408	T	T	T
0.3336675625926222	0.6597414539512408	0.4006250339719440	T	T	T

0.3334590542218228	0.8592544606880396	0.4010533108374383	T	T	T
0.4449416649669486	0.0589779732376001	0.4024385635366050	T	T	T
0.4437425310934860	0.2598371853822389	0.4078694865095916	T	T	T
0.4447874066185093	0.4582060520686419	0.4037917645743094	T	T	T
0.4449964312342426	0.6592353042527864	0.4015799997820582	T	T	T
0.4449539065303827	0.8589722302460056	0.4013524754277458	T	T	T
0.5561424320767043	0.0586555662404202	0.4028279191291929	T	T	T
0.5558938911998630	0.2585838245714702	0.4050780558822502	T	T	T
0.5558673526490866	0.4584061615280176	0.4047729554269270	T	T	T
0.5563249940019879	0.6588103986196695	0.4029956476300888	T	T	T
0.5562221749725313	0.8586162299301611	0.4023716172691753	T	T	T
0.6669759678855541	0.0586445872819817	0.4033768769492608	T	T	T
0.6669898623485124	0.2588112221964740	0.4040355380351821	T	T	T
0.6667962468935849	0.4588366605743468	0.4045432632179751	T	T	T
0.6672089988630455	0.6587565846511183	0.4040168872483559	T	T	T
0.6672081803637249	0.8586667231840400	0.4034857874874902	T	T	T
0.7777573761130431	0.0584334383342522	0.4037154963895173	T	T	T
0.7777235206734749	0.2588365856831629	0.4038114266410067	T	T	T
0.7776448271325083	0.4590404653298213	0.4043450418859252	T	T	T
0.7776877280772474	0.6588521116677010	0.4046037039222479	T	T	T
0.7779547808372325	0.8586354380824190	0.4040832064850350	T	T	T
0.8884704967669212	0.0583703161357295	0.4037666939930598	T	T	T
0.8884881121029177	0.2589967781958110	0.4038111711531778	T	T	T
0.8884686541480382	0.4589540711946276	0.4041160488015958	T	T	T
0.8884008691551801	0.6588564072797810	0.4042857402133165	T	T	T
0.8885345106353549	0.8585593178908640	0.4040689891187634	T	T	T
0.0550619919120604	0.1575946232952706	0.4324498587689359	T	T	T
0.0554201795604942	0.3576528702155307	0.4351202652116116	T	T	T
0.0550778364553733	0.5575559120735768	0.4314157615152873	T	T	T
0.0549645140789694	0.7577663446944031	0.4306609620397981	T	T	T
0.0550295910566684	0.9575338251530688	0.4313940418060449	T	T	T
0.1665124843891465	0.1575099237682862	0.4328248602597015	T	T	T
0.1671693636131424	0.3566264758028857	0.4352168737465417	T	T	T
0.1662892143865983	0.5581953024405706	0.4294586780234183	T	T	T
0.1662852326097621	0.7581292873926042	0.4295239171264665	T	T	T
0.1662484338941459	0.9576907276767881	0.4305154196989850	T	T	T
0.2777846405928510	0.1572492317302420	0.4321179984810780	T	T	T
0.2775554744821983	0.3568980618348342	0.4326637025743759	T	T	T
0.2777589061595797	0.5589704469855470	0.4283106042508535	T	T	T
0.2778321800678375	0.7582229241107150	0.4290189898040867	T	T	T
0.2777452541067356	0.9576323674754027	0.4297366666769962	T	T	T
0.3889098944388767	0.1574258151339081	0.4326241416730957	T	T	T
0.3883074370918003	0.3565989956168332	0.4356776592085621	T	T	T
0.3890682209148707	0.5580465170069421	0.4295720078400417	T	T	T
0.3891056836280176	0.7580047367479424	0.4293492152193240	T	T	T
0.3891255571340878	0.9577062172381089	0.4297812653418179	T	T	T
0.5004255990982106	0.1577508658600197	0.4321345196162268	T	T	T
0.5003167996610026	0.3574677646298876	0.4351253170954026	T	T	T
0.5004113868589795	0.5574419546560194	0.4314428929871613	T	T	T
0.5004920074782838	0.7575739355114639	0.4302897107538601	T	T	T
0.5004248415249269	0.9574834594712703	0.4304204645886617	T	T	T
0.6113690517366930	0.1573831585593084	0.4320279859439333	T	T	T
0.6114266436357901	0.3575744778373589	0.4332179206032311	T	T	T
0.6115003007817997	0.5574334244640863	0.4323141240465908	T	T	T

0.6116380614994097	0.7573125369624528	0.4313415265295748	T	T	T
0.6115316791530333	0.9572933498734194	0.4312965917506905	T	T	T
0.7221949075627614	0.1572362008135711	0.4319685908462332	T	T	T
0.7222925944468095	0.3574621967130522	0.4323382578511687	T	T	T
0.7222549992305716	0.5574861072968478	0.4326934023694558	T	T	T
0.7224935023492136	0.7573593237850240	0.4323873243251828	T	T	T
0.7223525888577662	0.9571807362436896	0.4320422195538897	T	T	T
0.8332217238770665	0.1570356034345678	0.4318620708638609	T	T	T
0.8330206484110191	0.3574416475878047	0.4322496337487091	T	T	T
0.8329886063716767	0.5575673843926358	0.4325147924840018	T	T	T
0.8331996040943793	0.7574014628484589	0.4325470298667154	T	T	T
0.8331867680107410	0.9572153667664525	0.4323209221286655	T	T	T
0.9441673512479967	0.1572995372034082	0.4322442969033731	T	T	T
0.9439158278178408	0.3576464742235601	0.4325712817890316	T	T	T
0.9437590469321637	0.5574915219543267	0.4320596300203515	T	T	T
0.9438935005190471	0.7574466713371607	0.4320595299968676	T	T	T
0.9439246760067329	0.9572834559900264	0.4320645169668149	T	T	T
0.9993343332306904	0.1276084371204662	0.5631285952876671	T	T	T
0.9984376699941691	0.3280650537697494	0.5641004832700502	T	T	T
0.9998727766614584	0.5284375990000949	0.5624915319186908	T	T	T
0.0000735040087408	0.7281354170898071	0.5621679201264326	T	T	T
0.9999142773410868	0.9279770430689465	0.5625121746160233	T	T	T
0.1104326191146166	0.1269558727247198	0.5628223479845367	T	T	T
0.1094058458977075	0.3288855574010606	0.5630966592868097	T	T	T
0.1113879661148040	0.5289179746496969	0.5617109738183144	T	T	T
0.1112016388258529	0.7283256395496747	0.5607268740875501	T	T	T
0.1111330280401334	0.9279129077544458	0.5616202428600455	T	T	T
0.2222315883754316	0.1263268629650158	0.5630926750717838	T	T	T
0.2238609153114198	0.3307662434140371	0.5625488746423306	T	T	T
0.2224245486343060	0.5300528101697710	0.5598654914496998	T	T	T
0.2221845382915456	0.7285288303508743	0.5598965597235526	T	T	T
0.2222348802950234	0.9277750121300414	0.5607641212657790	T	T	T
0.3333304730718987	0.1261802373787510	0.5631140748069582	T	T	T
0.3315772518443738	0.3306224739868321	0.5625717659199402	T	T	T
0.3329355411600641	0.5297853794448873	0.5599036180780682	T	T	T
0.3333560714406821	0.7286513155406258	0.5599921393393034	T	T	T
0.3333690677896994	0.9276923393169780	0.5604470295844473	T	T	T
0.4451253330665050	0.1270583144490766	0.5624455774282821	T	T	T
0.4461051396936678	0.3286707709834915	0.5629335772754711	T	T	T
0.4440177462184329	0.5289606905385950	0.5615456762174875	T	T	T
0.4444412113216512	0.7284413258636679	0.5605804256892335	T	T	T
0.4445930613850402	0.9278407709027490	0.5606488288486744	T	T	T
0.5559107606074342	0.1274297524169588	0.5627410144953502	T	T	T
0.5568811346525371	0.3278277155071043	0.5645756578354940	T	T	T
0.5552821538530411	0.5285086605193484	0.5627945645881076	T	T	T
0.5554248090722639	0.7281990165678204	0.5617975028201715	T	T	T
0.5555071754424896	0.9278659446485196	0.5616455738389269	T	T	T
0.6667040908459874	0.1277574265051895	0.5628621045438835	T	T	T
0.6672276680550212	0.3279653529206473	0.5638870750388717	T	T	T
0.6667426690042410	0.5282415130305880	0.5636282714975378	T	T	T
0.6665207480775464	0.7281912210309706	0.5631400790837008	T	T	T
0.6665822678350370	0.9279789465954303	0.5626432470913300	T	T	T
0.7777497731978185	0.1278566596984894	0.5628505252515207	T	T	T
0.7776862467612022	0.3280557953244533	0.5634879841053138	T	T	T

0.7777415667898506	0.5281325824376273	0.5637075561236691	T	T	T
0.7777333924729843	0.7281619053436573	0.5637196252116120	T	T	T
0.7777397513254225	0.9280028304908863	0.5633004307993908	T	T	T
0.8884750895077198	0.1278334144070091	0.5629106175770241	T	T	T
0.8882990529355335	0.3280958336962678	0.5635076112762301	T	T	T
0.8885360884303550	0.5282462045270074	0.5633154563303804	T	T	T
0.8888649058361301	0.7280358709454211	0.5634795897509302	T	T	T
0.8888007087502490	0.9279463944376402	0.5633063186432862	T	T	T
0.0552980939744875	0.0263330212904904	0.5905420166255643	T	T	T
0.0547618408871623	0.2258701999834176	0.5926979163013888	T	T	T
0.0552781141727803	0.4279229411432202	0.5915952915161515	T	T	T
0.0557173803054266	0.6271002524413171	0.5898412215442591	T	T	T
0.0556867916571540	0.8268854071541958	0.5901196366793993	T	T	T
0.1666740819314905	0.0259805241616333	0.5901563536197558	T	T	T
0.1657082031347812	0.2203822020089829	0.5951476466468643	T	T	T
0.1675548950365352	0.4289210246205010	0.5908799167608701	T	T	T
0.1668844728755753	0.6275561106762073	0.5885097502125781	T	T	T
0.1667656818507547	0.8268014510970833	0.5889851816186472	T	T	T
0.2779135750000385	0.0257415796968619	0.5895348111211441	T	T	T
0.2777491939496318	0.2222984885488872	0.5944131974372540	T	T	T
0.2776718267355524	0.4317550515606963	0.5905878829527480	T	T	T
0.2776945647329634	0.6279021295650545	0.5878163714813803	T	T	T
0.2778250768914648	0.8268833767280233	0.5883778572719315	T	T	T
0.3890469824598229	0.0257580383634494	0.5894687296541454	T	T	T
0.3898849151517280	0.2202670958768367	0.5950473175755752	T	T	T
0.3878311071283110	0.4286403011399946	0.5907418172647080	T	T	T
0.3888549910998353	0.6275551140466322	0.5886579184057158	T	T	T
0.3889330532432694	0.8269861684571347	0.5885332571823677	T	T	T
0.5000910605764223	0.0263344356571146	0.5898904202365400	T	T	T
0.5005671297618753	0.2257233071404162	0.5924195771584145	T	T	T
0.4999996028002900	0.4277169824596426	0.5919455762175458	T	T	T
0.4995844954526190	0.6272325125284777	0.5897834233294414	T	T	T
0.4999368621590836	0.8267906829896765	0.5892818985564797	T	T	T
0.6111188065264147	0.0263338274985134	0.5905829484716352	T	T	T
0.6117495517022473	0.2263998033180684	0.5921294614356811	T	T	T
0.6117965652541378	0.4270783992086112	0.5923188636523629	T	T	T
0.6108472027268329	0.6268373720720697	0.5912097888999128	T	T	T
0.6109386926406080	0.8268975154273204	0.5905863147180698	T	T	T
0.7219941196988194	0.0264561301260850	0.5911493039556786	T	T	T
0.7225240806560403	0.2262076357842770	0.5912495399254327	T	T	T
0.7224510398001793	0.4267987661950523	0.5919493446919544	T	T	T
0.7220999748461876	0.6267278189306302	0.5919037851738899	T	T	T
0.7220472293845175	0.8267501086907354	0.5914456352348609	T	T	T
0.8331185892047805	0.0265651926203435	0.5914790349116537	T	T	T
0.8330928049652903	0.2263823079449332	0.5911479629501442	T	T	T
0.8329476517524586	0.4267272650557201	0.5916211574471856	T	T	T
0.8332761375125392	0.6267506655876997	0.5920059081439611	T	T	T
0.8332568279187618	0.8267026490313265	0.5919274199865072	T	T	T
0.9442657967100797	0.0265145351580792	0.5912403385405339	T	T	T
0.9435606657859265	0.2264401923017370	0.5916773727200449	T	T	T
0.9434719328376127	0.4271481532474195	0.5918452752643295	T	T	T
0.9444671716008752	0.6267274715583955	0.5912420783627185	T	T	T
0.9445578904728877	0.8266871585947247	0.5913380166187976	T	T	T
0.2200018666271906	0.2738429672280664	0.7220048825380587	T	T	T

```
  0.3354680292004751  0.2744352422767405  0.7218025561508206   T   T   T
  0.1622544101370073  0.3419349049530900  0.7441104527251188   T   T   T
  0.2795658641623193  0.3721831172175541  0.7316442449203647   T   T   T
  0.3955661447013902  0.3410442151501092  0.7446400912974060   T   T   T
  0.2370904571758947  0.4870016781211393  0.7500037735809115   T   T   T
  0.3232290597561494  0.4874244105071623  0.7389989669407794   T   T   T
  0.1230019099620549  0.4309566862947824  0.7442297413720946   T   T   T
  0.4369029536934468  0.4282891470610504  0.7463829533144472   T   T   T
  0.1727465564412301  0.4456726701929403  0.8414634509551042   T   T   T
  0.3828718722791444  0.4516273627263900  0.8358446642368220   T   T   T
  0.1641419203091225  0.2997012696195183  0.6409776351144093   T   T   T
  0.2776876557579814  0.3080770304812658  0.6503269144836231   T   T   T
  0.3916956570201664  0.2995656204662834  0.6411194004194344   T   T   T
  0.2085382407453262  0.4115447767351774  0.7442267455452175   T   T   T
  0.3512527931408539  0.4113609466077849  0.7394760492932034   T   T   T
== On-top cluster Pd09-Te18
     1.0000000000000000
    31.7040281100000030    0.0000000000000000    0.0000000000000000
     0.0000000000000000   31.5721201999999970    0.0000000000000000
     0.0000000000000000    0.0000000000000000   22.0000000000000000
   W    Te   Te   Pd
    90   180    18     9
Selective dynamics
Direct
  0.0550404159389524  0.0776172939778648  0.4878162978425948   T   T   T
  0.0541485097461686  0.2773976188116071  0.4916550141656519   T   T   T
  0.0550686404725548  0.4785372816838436  0.4923252723092266   T   T   T
  0.0552399111975544  0.6784885952587978  0.4901061781086851   T   T   T
  0.0554280743350566  0.8781905529623802  0.4885699517425644   T   T   T
  0.1664683657237959  0.0774421908860690  0.4887962413288258   T   T   T
  0.1651463478414792  0.2765652201943994  0.4958573853078430   T   T   T
  0.1662218049877940  0.4787385906917650  0.4913133107151563   T   T   T
  0.1661687067042703  0.6785349721056559  0.4889316576782302   T   T   T
  0.1664814571077591  0.8779005596024574  0.4879324242838694   T   T   T
  0.2777646172928476  0.0769125244090315  0.4899712814281263   T   T   T
  0.2776041709666923  0.2763072545206512  0.4985435205415383   T   T   T
  0.2770490952641793  0.4792468425233903  0.4910556930905852   T   T   T
  0.2776518034187276  0.6788303567776269  0.4883459281014885   T   T   T
  0.2777871603749277  0.8776994285593964  0.4877563761317579   T   T   T
  0.3888160496574619  0.0768971847629259  0.4908858894353451   T   T   T
  0.3881170990132174  0.2756094466824267  0.5015673193608789   T   T   T
  0.3832082946262534  0.4789804397298075  0.4954236300761501   T   T   T
  0.3896944913177668  0.6784473537986865  0.4885197921129413   T   T   T
  0.3890042030219903  0.8777500505949259  0.4881432491492165   T   T   T
  0.5001300700400371  0.0770016259860567  0.4903862967130393   T   T   T
  0.5007032504205419  0.2770835010060028  0.4998618595007212   T   T   T
  0.5073352579177501  0.4785990606271185  0.4882314193018206   T   T   T
  0.5006498751137627  0.6786439407641137  0.4884116084674429   T   T   T
  0.5002322663690690  0.8779494672838609  0.4885633991977875   T   T   T
  0.6112066100116352  0.0771954426503718  0.4893450554362205   T   T   T
  0.6118972778565114  0.2763766737860813  0.4958763905821162   T   T   T
  0.6136617233806301  0.4794692288988572  0.4899802109786722   T   T   T
  0.6113729153245744  0.6789104901653480  0.4890192697578631   T   T   T
  0.6112397571149082  0.8780708541623542  0.4885223661799267   T   T   T
```

0.7227395281318162	0.0774737764579891	0.4882124990019086	T	T	T
0.7243210325225999	0.2768481603542766	0.4913134990244849	T	T	T
0.7230095443640214	0.4786459051678864	0.4919988213693156	T	T	T
0.7227527452041412	0.6785786681814421	0.4899119538656359	T	T	T
0.7225653123325008	0.8780626663710539	0.4887760174512115	T	T	T
0.8336598499003623	0.0775462939062992	0.4878320852904435	T	T	T
0.8343191309471726	0.2778110418529474	0.4897373672176232	T	T	T
0.8339328972840898	0.4784832506220279	0.4924629013993226	T	T	T
0.8337813773749410	0.6784688639542028	0.4905798650120333	T	T	T
0.8336983421664761	0.8780522845380440	0.4891551761329453	T	T	T
0.9441597076501086	0.0776922342026909	0.4879331235922099	T	T	T
0.9433987882066227	0.2778710059764283	0.4897628719528287	T	T	T
0.9441151223136064	0.4785317360297429	0.4926303363385620	T	T	T
0.9444490830456264	0.6785253727833218	0.4908262273180091	T	T	T
0.9442712351360217	0.8781759352118359	0.4890369903857167	T	T	T
0.9996756063730167	0.0067289796978911	0.4975982317170451	T	T	T
0.9995044097766814	0.2064294550190009	0.4988718236021883	T	T	T
0.9990097952418749	0.4071061723624921	0.5018461292870222	T	T	T
0.9998173838780674	0.6073646011491252	0.5006415031560532	T	T	T
0.9998726612503940	0.8072585808236340	0.4990781966748359	T	T	T
0.1109510114866633	0.0066390456890116	0.4974637667227145	T	T	T
0.1102124453025330	0.2058217029924785	0.4994092340577079	T	T	T
0.1104782681990898	0.4075849458613491	0.5022531859089204	T	T	T
0.1107603161075931	0.6073999597842694	0.4997527114764795	T	T	T
0.1108459916560586	0.8071298163116646	0.4984203178728591	T	T	T
0.2221540809634294	0.0061820556334097	0.4979852647522670	T	T	T
0.2222720399087957	0.2055035231573165	0.5033857555386533	T	T	T
0.2223660634748949	0.4080764774997550	0.5017807036030149	T	T	T
0.2221387647252900	0.6079862363202948	0.4991373707226459	T	T	T
0.2221168969102752	0.8069767301119495	0.4976190019647123	T	T	T
0.3333749258521601	0.0059329580574592	0.4987934214343681	T	T	T
0.3328632550648858	0.2045196527827676	0.5056952956322651	T	T	T
0.3313069881867909	0.4076094655191018	0.5060752431491531	T	T	T
0.3333478013737624	0.6084657438619716	0.4995388097975802	T	T	T
0.3336203384441047	0.8068486541965274	0.4973549676286417	T	T	T
0.4445227763419395	0.0060174444922501	0.4990971727591358	T	T	T
0.4445892989240331	0.2047231294493665	0.5053249062516114	T	T	T
0.4380347196568925	0.4087625785035328	0.5068076354096346	T	T	T
0.4454582321301723	0.6072978774454988	0.4978733069584688	T	T	T
0.4447862621060112	0.8069621250014998	0.4979395390008389	T	T	T
0.5557473775643625	0.0062214957742175	0.4986759452715319	T	T	T
0.5559361683179901	0.2055723264587378	0.5033553238481631	T	T	T
0.5627247125473774	0.4081814310026242	0.5000266367238211	T	T	T
0.5563097906756701	0.6080998242640789	0.4983010033649330	T	T	T
0.5557302705507290	0.8071715216386962	0.4982221689721947	T	T	T
0.6668370664045984	0.0065414055590999	0.4981477416864087	T	T	T
0.6663531370638915	0.2055398458420718	0.5001488767033102	T	T	T
0.6676899877916784	0.4076289902576544	0.5013445060477240	T	T	T
0.6670980661043665	0.6077655493988885	0.4992885268719675	T	T	T
0.6668532099860667	0.8072217410101155	0.4985935846492308	T	T	T
0.7782961678313571	0.0065116728905213	0.4977111140116566	T	T	T
0.7792281525021282	0.2060788364406932	0.4984196622001629	T	T	T
0.7790641190736339	0.4070118385712295	0.5013249166720085	T	T	T
0.7785087859794525	0.6073850572102732	0.5006320481037599	T	T	T

0.7782708453209797	0.8070897637720165	0.4991054668353144	T	T	T
0.8889766679174688	0.0066111667767900	0.4976462135145571	T	T	T
0.8887647781590314	0.2063603530630449	0.4978684056979383	T	T	T
0.8889168172213422	0.4069463451129069	0.5011624699922349	T	T	T
0.8891401439774476	0.6073539710033569	0.5009080775120244	T	T	T
0.8890285957555136	0.8070803718193981	0.4991744796891825	T	T	T
0.9996767140325883	0.0581759029619220	0.3988568953614889	T	T	T
0.9998314723077263	0.2587220612834991	0.4012867877325285	T	T	T
0.9995778799449584	0.4587498851290970	0.4036215561048184	T	T	T
0.9996397762922541	0.6585737484639403	0.4015925685968054	T	T	T
0.9997179466198605	0.8583987824506178	0.4000616234097440	T	T	T
0.1110240968373293	0.0587138000441849	0.3992370846987680	T	T	T
0.1114838943298356	0.2593789540787861	0.4038046693364134	T	T	T
0.1104902095922375	0.4584191492964452	0.4033440383992796	T	T	T
0.1103253948704393	0.6585642192516404	0.4006855821062743	T	T	T
0.1107041371063718	0.8583450318558209	0.3994452842318971	T	T	T
0.2223163990821488	0.0585172304460279	0.4001965617091355	T	T	T
0.2225085187763964	0.2589555246275667	0.4079984790644702	T	T	T
0.2216888765068686	0.4588164033170893	0.4023949318705822	T	T	T
0.2217541575048602	0.6588588810483912	0.3998306618649125	T	T	T
0.2219731108994925	0.8583982873978440	0.3989608711059096	T	T	T
0.3335088401254361	0.0584516513951117	0.4011372819297780	T	T	T
0.3337616436017587	0.2579583209632289	0.4102921101786114	T	T	T
0.3323996349229498	0.4565063319164440	0.4035946892457870	T	T	T
0.3336029366399380	0.6589752353969561	0.3999408491857805	T	T	T
0.3334609271622533	0.8584400282552996	0.3989330304369069	T	T	T
0.4443335806059240	0.0584315941767508	0.4014198780022665	T	T	T
0.4441978333032670	0.2596585065437745	0.4115731373349208	T	T	T
0.4432354742610338	0.4587113651641664	0.4090646813367462	T	T	T
0.4450846226381206	0.6586693244622915	0.3994482396706784	T	T	T
0.4447370720645480	0.8584938684569573	0.3994249297772994	T	T	T
0.5553388926853479	0.0583804877096679	0.4006590733138521	T	T	T
0.5544917781966029	0.2597877816364677	0.4081738604378795	T	T	T
0.5598279907522361	0.4567902229803409	0.3985234411062010	T	T	T
0.5562803944824680	0.6594651924972612	0.3995610093573205	T	T	T
0.5557602993773783	0.8586934531144775	0.3995602299817156	T	T	T
0.6667354413641340	0.0584685945256609	0.3998111868481454	T	T	T
0.6661384813904466	0.2585550022881301	0.4044438477004150	T	T	T
0.6688545116134077	0.4583419894920960	0.4018747058045909	T	T	T
0.6674175177258620	0.6592536837898566	0.4006413929670726	T	T	T
0.6670304679401435	0.8585912565000734	0.3998448424603614	T	T	T
0.7780623431054147	0.0580620729390588	0.3990199676116749	T	T	T
0.7783038789492398	0.2585942271826785	0.4006429918171523	T	T	T
0.7786966140944810	0.4589357752509549	0.4032870377138090	T	T	T
0.7784650337822382	0.6584563270815909	0.4016018915719978	T	T	T
0.7782434602055212	0.8583679983079551	0.4002233560615446	T	T	T
0.8889022935598665	0.0580730131765804	0.3988020597192380	T	T	T
0.8889189294360679	0.2587316343255689	0.3996695798909415	T	T	T
0.8891318231572141	0.4591016763408617	0.4032126353244663	T	T	T
0.8892323478492057	0.6585809999832700	0.4018113009113093	T	T	T
0.8889342003101416	0.8582092223261000	0.4001287968972083	T	T	T
0.0550719365626560	0.1570827977484423	0.4276680126723059	T	T	T
0.0549929074684230	0.3576045047026523	0.4324500687593764	T	T	T
0.0550088760846531	0.5572530846441150	0.4306307276901095	T	T	T

0.0550624958313700	0.7573461731951028	0.4289092318400100	T	T	T
0.0553221586155934	0.9570447409416171	0.4273878688873207	T	T	T
0.1669819783459160	0.1572127628204007	0.4297437524960302	T	T	T
0.1661388492558315	0.3565354359694895	0.4336638854861000	T	T	T
0.1662453235657849	0.5577046029938045	0.4300010628433526	T	T	T
0.1660722075169695	0.7574316250527947	0.4278012776350036	T	T	T
0.1664689155403981	0.9569516976498291	0.4272707934925478	T	T	T
0.2780667651063451	0.1568712856598463	0.4314829934428441	T	T	T
0.2780324886910070	0.3556740879160525	0.4352545649022889	T	T	T
0.2777115161268313	0.5582156885432739	0.4295703068628934	T	T	T
0.2776038390896577	0.7574675093689908	0.4271647700315319	T	T	T
0.2778672169415280	0.9568991108732742	0.4275277267693543	T	T	T
0.3887830662749985	0.1569223213564183	0.4329775699891052	T	T	T
0.3874953459581896	0.3553306947692957	0.4391736241316065	T	T	T
0.3878666727046033	0.5569173754467395	0.4313533355939044	T	T	T
0.3894373175383463	0.7572665937931936	0.4273608246694074	T	T	T
0.3889882558698595	0.9568838116990512	0.4280097988118557	T	T	T
0.4998315621832854	0.1569351330220797	0.4316575097702511	T	T	T
0.4991465893687557	0.3593227916354713	0.4401308463201215	T	T	T
0.5021020080295198	0.5576800111807159	0.4275576911313090	T	T	T
0.5004379075317025	0.7575494231709501	0.4274354531461169	T	T	T
0.5000539983603521	0.9568491590106328	0.4281544138033819	T	T	T
0.6104545112179258	0.1567741884768352	0.4298797974689959	T	T	T
0.6130343427071324	0.3557303271386345	0.4304776251241673	T	T	T
0.6125588123158965	0.5581570362327488	0.4282871775649513	T	T	T
0.6115299544987417	0.7577207632564434	0.4282786412389655	T	T	T
0.6113277624410893	0.9570732231711757	0.4278040275609652	T	T	T
0.7224881665891438	0.1570568977231941	0.4285724590059461	T	T	T
0.7231325617235957	0.3569830815655526	0.4318946773733410	T	T	T
0.7233184245784693	0.5574845240970037	0.4307636868373375	T	T	T
0.7227831913374145	0.7574712391474137	0.4289111863529829	T	T	T
0.7224398147772912	0.9569267515223820	0.4277317877423200	T	T	T
0.8337335520404076	0.1566101449431002	0.4268317537433475	T	T	T
0.8339198469499394	0.3573641268136680	0.4299533880027612	T	T	T
0.8339695991296504	0.5572771198839069	0.4310927246766966	T	T	T
0.8337263277590897	0.7572077351199856	0.4290504399280623	T	T	T
0.8336716074929292	0.9566824967340987	0.4275176791277915	T	T	T
0.9442955294738640	0.1568949246992305	0.4273592077657494	T	T	T
0.9442077906739866	0.3575240943530824	0.4302718889202971	T	T	T
0.9443322539299919	0.5572364417211347	0.4310766111484690	T	T	T
0.9445124408377757	0.7572085187724489	0.4291820118655216	T	T	T
0.9443644283305762	0.9567693171594382	0.4274425712544890	T	T	T
0.9994211031089910	0.1271943348631128	0.5584719911003565	T	T	T
0.9984159148258424	0.3272605889251926	0.5612572293905671	T	T	T
0.9999129309916904	0.5285058746281924	0.5622476925402620	T	T	T
0.0000521587981562	0.7284639929359407	0.5603794014070492	T	T	T
0.9999788618962555	0.9279369381344001	0.5586677855415911	T	T	T
0.1101218625818121	0.1263970879903373	0.5589426994187536	T	T	T
0.1083913563584047	0.3280271196343155	0.5608751427490161	T	T	T
0.1110231455202617	0.5287635166696363	0.5616023658679347	T	T	T
0.1111172815656098	0.7282714510327875	0.5595448068540682	T	T	T
0.1111091203669074	0.9277239967043559	0.5583149254510820	T	T	T
0.2217979082384095	0.1254461499985096	0.5609676646769589	T	T	T
0.2213307650588166	0.3295519241873673	0.5607946954943192	T	T	T

0.2219770853279961	0.5296201455052217	0.5606410164876787	T	T	T
0.2220678073317926	0.7283301407436988	0.5584151038782464	T	T	T
0.2221109905075343	0.9271807995624619	0.5581607581228029	T	T	T
0.3327831760740820	0.1246713336270099	0.5632501367457521	T	T	T
0.3311574485963351	0.3293879554636074	0.5667579412760569	T	T	T
0.3312229334366198	0.5304826966488266	0.5627102579381288	T	T	T
0.3336856641323641	0.7281559057604384	0.5580648195100090	T	T	T
0.3332151443053277	0.9268304967456734	0.5587401407979622	T	T	T
0.4446778078313534	0.1248731358401311	0.5627482692334346	T	T	T
0.4437925678816287	0.3302597551506999	0.5647159239822598	T	T	T
0.4462826280374790	0.5265054629855728	0.5533012668174911	T	T	T
0.4450003176254224	0.7281384568438065	0.5587008539558342	T	T	T
0.4444311151973939	0.9269023707888503	0.5591521407994438	T	T	T
0.5560158876055329	0.1254610004089690	0.5613973086827669	T	T	T
0.5578864599984177	0.3307382282013457	0.5588828654135366	T	T	T
0.5571226802138952	0.5293744907438347	0.5598842199676648	T	T	T
0.5556888940621454	0.7283941585031540	0.5590405200868754	T	T	T
0.5557393393759618	0.9273202111281806	0.5591157256446683	T	T	T
0.6675269951674304	0.1262193292281973	0.5594777635611924	T	T	T
0.6699383460308201	0.3279990488316063	0.5608047291744209	T	T	T
0.6676781393112723	0.5293609487089486	0.5615003624172322	T	T	T
0.6668760851073311	0.7284319641499309	0.5593800985338689	T	T	T
0.6668648506892436	0.9275961641792242	0.5587622774463415	T	T	T
0.7786896063979520	0.1269746425945502	0.5585596981978833	T	T	T
0.7794279959694346	0.3270944012303092	0.5611385147110945	T	T	T
0.7785515850751502	0.5286175198413126	0.5624305792727872	T	T	T
0.7781375173490060	0.7282934106655301	0.5602463304695142	T	T	T
0.7781392714179529	0.9277919156402915	0.5589375927410486	T	T	T
0.8889126242197953	0.1273659910879311	0.5585136677714857	T	T	T
0.8888066744617059	0.3274174454853844	0.5611904322540244	T	T	T
0.8889695318794273	0.5285352447090188	0.5627261450952201	T	T	T
0.8890222201904292	0.7284122816962737	0.5605894140698318	T	T	T
0.8889747904491595	0.9279715186127789	0.5592441399073406	T	T	T
0.0553705977020184	0.0260300869104649	0.5863202833482589	T	T	T
0.0544999427756273	0.2249452859581197	0.5886467743716830	T	T	T
0.0545796182380646	0.4272888913229319	0.5910513373497375	T	T	T
0.0556595181909176	0.6271886232209114	0.5889714229063429	T	T	T
0.0556005237065677	0.8269654076773987	0.5876185209289605	T	T	T
0.1663952481507443	0.0253403739902039	0.5867936200617416	T	T	T
0.1644720243395378	0.2193669976828003	0.5921040320692367	T	T	T
0.1662294860551936	0.4274729235493553	0.5902140230354145	T	T	T
0.1664467322147305	0.6276159612444513	0.5881445779899970	T	T	T
0.1667768377369367	0.8267026992916932	0.5868434459668003	T	T	T
0.2774912127271356	0.0245415844756201	0.5876250477266607	T	T	T
0.2766760979830123	0.2204315348313831	0.5956343355464166	T	T	T
0.2753261606222618	0.4302564110759782	0.5916280743851646	T	T	T
0.2775482973849421	0.6284919445678629	0.5879519737462394	T	T	T
0.2780306010279111	0.8260968387002735	0.5863044422126727	T	T	T
0.3888747263351208	0.0243752488382450	0.5882965236745115	T	T	T
0.3887796871400211	0.2181253061409102	0.5968073364976485	T	T	T
0.3858950680640216	0.4300762479261770	0.5976129076479946	T	T	T
0.3899712961838260	0.6269411890667415	0.5873725536681502	T	T	T
0.3889245494809157	0.8260966363006433	0.5866040032966128	T	T	T
0.5002614492833911	0.0246210794609376	0.5881231749269692	T	T	T

```
  0.5007241169514624  0.2200214806741360  0.5948724363586968   T   T   T
  0.5023439744216568  0.4277016909094714  0.5834709277292178   T   T   T
  0.5005316844250000  0.6270634973495298  0.5871432188733404   T   T   T
  0.5000873290265899  0.8264514037053341  0.5871567943191833   T   T   T
  0.6114844518135856  0.0252297270694323  0.5875784382025607   T   T   T
  0.6128378172491974  0.2195481899062783  0.5930540926112635   T   T   T
  0.6140032474092844  0.4295370665240522  0.5913482736100011   T   T   T
  0.6116016670137085  0.6274578206655017  0.5876927778738557   T   T   T
  0.6112213054471192  0.8267260308390600  0.5873310102371351   T   T   T
  0.7227518859468037  0.0259139995204570  0.5866947965585290   T   T   T
  0.7236318424555029  0.2244530454966537  0.5880574746382257   T   T   T
  0.7235976320966331  0.4271037632630206  0.5902795922602447   T   T   T
  0.7226043227220273  0.6272703552239547  0.5886554135591585   T   T   T
  0.7223916234703897  0.8267555583608246  0.5875956523256373   T   T   T
  0.8336622521566383  0.0261560911064045  0.5867000855876682   T   T   T
  0.8340390664389268  0.2256965240042324  0.5878622448258147   T   T   T
  0.8339130859982831  0.4264136482709048  0.5907443367425765   T   T   T
  0.8337385624459062  0.6273860752189028  0.5897077500708475   T   T   T
  0.8336058035011833  0.8267389096479962  0.5881614073478345   T   T   T
  0.9443591991516299  0.0261670096835275  0.5867374601189727   T   T   T
  0.9438035364856561  0.2257758318270456  0.5876172998614537   T   T   T
  0.9438266904224298  0.4265096714087823  0.5909362941291977   T   T   T
  0.9445714749410214  0.6271108036462272  0.5897738306753746   T   T   T
  0.9445223135597131  0.8269093487699278  0.5880755070216952   T   T   T
  0.2167010561770289  0.2703258459951970  0.7210561974776460   T   T   T
  0.3293220009946928  0.2761120005382338  0.7236225954453350   T   T   T
  0.4498525741479401  0.2771315540193217  0.7214248666412009   T   T   T
  0.5591288755700849  0.2712552516109988  0.7191004292967925   T   T   T
  0.1582413221185574  0.3382926604060899  0.7453052812818707   T   T   T
  0.2732876201207526  0.3726899879445650  0.7268395607806059   T   T   T
  0.3872978115718477  0.3537137549522097  0.7456052319844729   T   T   T
  0.5003997156986484  0.3753740233681784  0.7165816376513082   T   T   T
  0.6155944232119561  0.3405955900708886  0.7444381298317773   T   T   T
  0.2203517288178203  0.4884445659695469  0.7211372240721222   T   T   T
  0.3098169183731782  0.4960880742297343  0.7289483277892526   T   T   T
  0.4637377639764502  0.4960045022377498  0.7017712978962980   T   T   T
  0.5501794697432407  0.4901180434187046  0.7306508672055885   T   T   T
  0.1145475670686432  0.4237769557888069  0.7497737464534725   T   T   T
  0.6562166357867816  0.4281473115131461  0.7538578547894812   T   T   T
  0.1733658919835648  0.4581396156827138  0.8259054275146881   T   T   T
  0.3905074336915660  0.4732650817678722  0.7852050125762914   T   T   T
  0.5969961466554835  0.4540643670908814  0.8353060242966267   T   T   T
  0.1610924168375760  0.2977865555765389  0.6408759805364714   T   T   T
  0.2728060967707646  0.3068035573713072  0.6486244796091430   T   T   T
  0.3887607536843402  0.3021495612489141  0.6462587682241737   T   T   T
  0.5045539491370196  0.3076413826248921  0.6431296223349532   T   T   T
  0.6150824839625333  0.2990757170497040  0.6393879059238327   T   T   T
  0.1995436326089585  0.4090344662164377  0.7320159296364578   T   T   T
  0.3404804353588545  0.4199560109097938  0.7236838369961909   T   T   T
  0.4315050520241855  0.4203281799386603  0.7125968180276158   T   T   T
  0.5728183767407286  0.4113075707597843  0.7347176560222740   T   T   T
== On-top cluster Pd13-Te25
     1.0000000000000000
     31.7040281100000030    0.0000000000000000    0.0000000000000000
```

```
     0.0000000000000000   31.5721201999999970    0.0000000000000000
     0.0000000000000000    0.0000000000000000   22.0000000000000000
   W    Te   Te   Pd
    90   180    25    13
Selective dynamics
Direct
  0.0551629840568394  0.0769910007191088  0.4861095550782673   T   T   T
  0.0552348461517444  0.2769238162708265  0.4914975146829405   T   T   T
  0.0554372112866358  0.4784781683272100  0.4929193419039000   T   T   T
  0.0554741378065532  0.6782528013472880  0.4900691439402366   T   T   T
  0.0556584459412697  0.8776657947975020  0.4872446445876981   T   T   T
  0.1663721641287833  0.0771348228315965  0.4876645843783544   T   T   T
  0.1645935905929876  0.2762954771671530  0.4952085147669232   T   T   T
  0.1661183515292286  0.4785005291832756  0.4922966785169662   T   T   T
  0.1664151084111797  0.6783009122791089  0.4897926052823835   T   T   T
  0.1665970215792195  0.8776255307100436  0.4873801488837176   T   T   T
  0.2776882288460238  0.0766063298074716  0.4889878909820495   T   T   T
  0.2772461966540035  0.2760349868842449  0.4979091382905795   T   T   T
  0.2770112715934424  0.4789432821933487  0.4926052000965825   T   T   T
  0.2777667513662897  0.6786608431302535  0.4897825260443579   T   T   T
  0.2777598959289739  0.8775673204409405  0.4877665330753642   T   T   T
  0.3887825838938259  0.0768937617675623  0.4900576572394761   T   T   T
  0.3867876815493054  0.2752500202205564  0.5010508568086887   T   T   T
  0.3831665962651886  0.4791407256062309  0.4963854777316365   T   T   T
  0.3893769476357457  0.6785136964685039  0.4895999327843903   T   T   T
  0.3890013057814276  0.8777321928606207  0.4878606963669294   T   T   T
  0.5000704596666141  0.0768971622151236  0.4899779102577723   T   T   T
  0.4998679905816381  0.2767427125073961  0.5030585566257795   T   T   T
  0.5066556951279947  0.4782384510428495  0.4897292176917504   T   T   T
  0.5000186587890679  0.6789223380753615  0.4887934248199213   T   T   T
  0.5000402482270153  0.8778795152829572  0.4875243962113322   T   T   T
  0.6111489231403652  0.0768813414323682  0.4895489872225233   T   T   T
  0.6135521881682996  0.2755163772282521  0.5001684804405794   T   T   T
  0.6138472039605950  0.4797349331548151  0.4942968104122930   T   T   T
  0.6110561193679298  0.6790644384279658  0.4882188141242481   T   T   T
  0.6110859966622537  0.8777549390226429  0.4870023707786237   T   T   T
  0.7224898470085944  0.0767562092406227  0.4882898674578492   T   T   T
  0.7234204996719157  0.2764294006703276  0.4967345219116989   T   T   T
  0.7240389781598727  0.4795951114505608  0.4913738168690173   T   T   T
  0.7231256362234717  0.6789728108219633  0.4883132212019211   T   T   T
  0.7224989715778692  0.8777555136305872  0.4867673108899124   T   T   T
  0.8335986868040857  0.0771068429865634  0.4870011462750481   T   T   T
  0.8344253886990102  0.2765282032132930  0.4948616550620892   T   T   T
  0.8342710139605523  0.4788624683620701  0.4916325143023559   T   T   T
  0.8341260729986061  0.6786231543568966  0.4889598043791250   T   T   T
  0.8336122646623287  0.8778030902994987  0.4869022909947766   T   T   T
  0.9447443671835066  0.0771201725581932  0.4858328620156989   T   T   T
  0.9447578012291146  0.2766760633859567  0.4909720932641858   T   T   T
  0.9446453777369800  0.4784412428186191  0.4926165580106848   T   T   T
  0.9447974469171209  0.6782947059220145  0.4898509512740847   T   T   T
  0.9445010196651593  0.8777485241230686  0.4870637971946826   T   T   T
  0.0001360806700476  0.0060546006711507  0.4952836752806891   T   T   T
  0.0007579202537096  0.2056610577965635  0.4985358844388467   T   T   T
  0.0001089325855465  0.4068057268083699  0.5022355493053233   T   T   T
```

0.0002658573174434	0.6071701888644551	0.5003600372169045	T	T	T
0.0001802288782114	0.8068494904376095	0.4977891517644040	T	T	T
0.1110577621825905	0.0061766463998775	0.4960606399382892	T	T	T
0.1101026511198160	0.2053504126413174	0.4984925424068734	T	T	T
0.1107574459314675	0.4073416770689018	0.5025596384252845	T	T	T
0.1109182035876241	0.6072647424674070	0.5003903459209498	T	T	T
0.1111302511479957	0.8067842969581778	0.4978476618089177	T	T	T
0.2222015693269868	0.0059758968897709	0.4972694505686892	T	T	T
0.2225954689141755	0.2051809389226742	0.5025683824386235	T	T	T
0.2223218559525910	0.4077169444713366	0.5029997510104166	T	T	T
0.2222788339405607	0.6076325211738965	0.5003933007190424	T	T	T
0.2221661342487369	0.8067864969836179	0.4978993780892774	T	T	T
0.3334104844909298	0.0057513265974552	0.4981162918212323	T	T	T
0.3323709530529158	0.2042931480497137	0.5050833260312055	T	T	T
0.3307875211519644	0.4073167146443379	0.5070242854808634	T	T	T
0.3332384139790355	0.6081900057477636	0.5009041595859495	T	T	T
0.3334922564009470	0.8068781876883583	0.4978522873918877	T	T	T
0.4443834014278693	0.0060313127900555	0.4983056867081991	T	T	T
0.4445823309120031	0.2047381794056471	0.5057270279641142	T	T	T
0.4360970473175804	0.4084134975739580	0.5070668483666246	T	T	T
0.4452101820722199	0.6074671555425007	0.4988123812979712	T	T	T
0.4444523819079420	0.8070211486475664	0.4978146731821796	T	T	T
0.5554980199192970	0.0059928992664849	0.4979925515575935	T	T	T
0.5554627606859948	0.2047934905463644	0.5056434942476545	T	T	T
0.5637013362220527	0.4080631217771571	0.5050068273821092	T	T	T
0.5555110721679020	0.6086880739205519	0.4986886434045554	T	T	T
0.5553366531457673	0.8071178175435533	0.4971326574307625	T	T	T
0.6666491463163854	0.0059121236410658	0.4972172320972234	T	T	T
0.6676248860796029	0.2044335477160650	0.5041081832133947	T	T	T
0.6685128859629944	0.4084766064572199	0.5057999444893890	T	T	T
0.6672788358261377	0.6086314857006518	0.4989323844304856	T	T	T
0.6667408164399358	0.8070262245621366	0.4967606750605792	T	T	T
0.7779971437277921	0.0060584598285124	0.4964468687289524	T	T	T
0.7780925138492020	0.2053096995433877	0.5015525446017780	T	T	T
0.7788304524262163	0.4080517483576407	0.5018426748621356	T	T	T
0.7788372506580419	0.6080806592648413	0.4990494695278112	T	T	T
0.7782975513837769	0.8070402492437838	0.4971080528553222	T	T	T
0.8890021944720429	0.0062787579439163	0.4956133968110835	T	T	T
0.8886716361351249	0.2053794901864474	0.4981316314208544	T	T	T
0.8889019440083125	0.4073550573426878	0.5021529015184600	T	T	T
0.8894745264145348	0.6073656169108951	0.4996813508368778	T	T	T
0.8891889529497946	0.8069040788367661	0.4975723474705543	T	T	T
0.0000424344954300	0.0577399588341518	0.3966217247001631	T	T	T
0.0001027799443747	0.2584410899327365	0.4013708645979508	T	T	T
0.0002459695265273	0.4583218686406578	0.4039940926417576	T	T	T
0.0002624503986360	0.6581738613370844	0.4011102799621289	T	T	T
0.0001381468207681	0.8576150125512240	0.3984663698144043	T	T	T
0.1113046013267612	0.0582274192703607	0.3977863142257061	T	T	T
0.1115985022371448	0.2588656924902458	0.4025688207350021	T	T	T
0.1107889750866224	0.4583639300316459	0.4037905119723887	T	T	T
0.1109269927243809	0.6582329426424184	0.4011708234212207	T	T	T
0.1111889701671975	0.8576599619684572	0.3985018144074311	T	T	T
0.2223592412621520	0.0581020967891729	0.3992466858961289	T	T	T
0.2221716680237281	0.2588629359200776	0.4073823533116436	T	T	T

0.2218622992415346	0.4585560962819930	0.4036930040801477	T	T	T
0.2222437890826477	0.6584033750129381	0.4011821856384464	T	T	T
0.2222624267909210	0.8578268103792294	0.3987794149960134	T	T	T
0.3335665965781890	0.0580940188395091	0.4003444072738453	T	T	T
0.3332323740136427	0.2576191301330812	0.4092658140024025	T	T	T
0.3322477440970321	0.4564207918829081	0.4047806133635070	T	T	T
0.3334431094231078	0.6586282241015021	0.4012127671379641	T	T	T
0.3333901142572924	0.8581579083438485	0.3989773933812083	T	T	T
0.4444615483368377	0.0586782797136321	0.4007127543466478	T	T	T
0.4445621415612394	0.2600645067398397	0.4127121504997945	T	T	T
0.4431305314971123	0.4587096734848691	0.4097838272241581	T	T	T
0.4443682578188579	0.6585463135862855	0.4002412374391639	T	T	T
0.4444514659563351	0.8580683654277087	0.3988202457499882	T	T	T
0.5553994071340489	0.0585957155586892	0.4004055287506185	T	T	T
0.5553276353247520	0.2601489565272291	0.4126855150236279	T	T	T
0.5605730384568669	0.4559987161967594	0.4023590574237545	T	T	T
0.5553814407295871	0.6593386630581323	0.3994102916928284	T	T	T
0.5553496293636818	0.8583702134126272	0.3982567519675659	T	T	T
0.6663766955263437	0.0584930500391438	0.3996650265913191	T	T	T
0.6671563824605053	0.2577485156196069	0.4085124595837971	T	T	T
0.6680965524938342	0.4580202311610299	0.4046796569240276	T	T	T
0.6671260819194929	0.6593275331436707	0.3997395386642758	T	T	T
0.6667211050908513	0.8583612142608378	0.3979840864644140	T	T	T
0.7777101831768759	0.0581985315578484	0.3983899025473593	T	T	T
0.7779221140651349	0.2589354522640582	0.4059816154803674	T	T	T
0.7788831249921269	0.4589696753752590	0.4025009357277372	T	T	T
0.7786863941831560	0.6588566055359342	0.3996383789548920	T	T	T
0.7780953450902944	0.8580168915261898	0.3980065982867351	T	T	T
0.8888275234055424	0.0582156341575864	0.3972103187482733	T	T	T
0.8880291305496605	0.2589969418386761	0.4025538933532249	T	T	T
0.8895920590911236	0.4583363367475332	0.4033303992823067	T	T	T
0.8897096406404890	0.6584619021221418	0.4005518546081078	T	T	T
0.8890808355561445	0.8577051272674259	0.3981913604452023	T	T	T
0.0553792265437371	0.1565986657074714	0.4261972176452210	T	T	T
0.0556505821491230	0.3572162717088582	0.4322671350985022	T	T	T
0.0555772642183002	0.5570324281028107	0.4308797066377462	T	T	T
0.0555801670072269	0.7568124846894838	0.4281509160757826	T	T	T
0.0557287725519842	0.9562828117058314	0.4255398569865589	T	T	T
0.1669483609767151	0.1568953408019899	0.4287517054853677	T	T	T
0.1661766311575453	0.3563889621888567	0.4337765144451465	T	T	T
0.1664527028176867	0.5574719847010503	0.4310016067637655	T	T	T
0.1665602307154723	0.7568816938462057	0.4280913631261352	T	T	T
0.1667390913646273	0.9565521508154414	0.4264143755305553	T	T	T
0.2779292805532723	0.1565962399591331	0.4304502472580161	T	T	T
0.2777626755081172	0.3558184253972133	0.4355535439641051	T	T	T
0.2776492261724142	0.5578707384824443	0.4310044286897101	T	T	T
0.2777380072909423	0.7571535720127148	0.4280831846743452	T	T	T
0.2778122727944403	0.9565858402213605	0.4271937330093044	T	T	T
0.3889799151303982	0.1571620498156198	0.4325733185304501	T	T	T
0.3865396079191035	0.3553385752803193	0.4387561616914751	T	T	T
0.3875723572523619	0.5569851981975985	0.4321949067931371	T	T	T
0.3890180852220718	0.7570841772546525	0.4278946899553164	T	T	T
0.3889815601221126	0.9566944171933961	0.4273379120228682	T	T	T
0.5000456428710550	0.1572518433885831	0.4319384464590637	T	T	T

0.4996089309191527	0.3596674149553826	0.4458434396312149	T	T	T
0.5015377806266178	0.5574473202658770	0.4285177207359457	T	T	T
0.4997758339553078	0.7574308974738764	0.4270661974353606	T	T	T
0.4998904260851449	0.9569104003845497	0.4271222305548954	T	T	T
0.6108945230907018	0.1571462899573888	0.4321301925415154	T	T	T
0.6139802498067607	0.3554320908443001	0.4364105522087716	T	T	T
0.6122962376529356	0.5580365221132910	0.4308348424949323	T	T	T
0.6109361251561899	0.7576114992583898	0.4267854557326821	T	T	T
0.6109358841511119	0.9568403966871645	0.4267825500107864	T	T	T
0.7221912761213927	0.1566014042252129	0.4296993175262848	T	T	T
0.7225757653101414	0.3566070954035083	0.4348718826285349	T	T	T
0.7234694263543454	0.5583592054323137	0.4294463537225188	T	T	T
0.7227368326765068	0.7575130057537562	0.4269766272251100	T	T	T
0.7222998103141905	0.9567998533985883	0.4261999803867749	T	T	T
0.8329246693525411	0.1566470422155096	0.4275266801140242	T	T	T
0.8338838620144154	0.3563788812815300	0.4327204955097306	T	T	T
0.8343481458503801	0.5575442289537809	0.4296265031120324	T	T	T
0.8339635996777829	0.7572061463811116	0.4273637698791957	T	T	T
0.8335512500207866	0.9565895687851180	0.4256598101454333	T	T	T
0.9446398430915957	0.1569781406169424	0.4268147474043986	T	T	T
0.9443709583046606	0.3571971502756345	0.4322726332271465	T	T	T
0.9450133175271015	0.5570157462991081	0.4305191786324467	T	T	T
0.9448003040177012	0.7568094252066970	0.4280440891095939	T	T	T
0.9444547299081255	0.9564043735094907	0.4254352785296371	T	T	T
0.0000618142142496	0.1261317491354460	0.5572180730415693	T	T	T
0.9999165050455958	0.3266507899997005	0.5614327288853898	T	T	T
0.0000873242144750	0.5285674737628338	0.5624300224790003	T	T	T
0.0001322532520982	0.7283059520568785	0.5597170100508984	T	T	T
0.0000754580362057	0.9275068724779645	0.5568533827167592	T	T	T
0.1100937765731695	0.1258746126635148	0.5578180755939230	T	T	T
0.1087811522593372	0.3276918309984332	0.5609726595103022	T	T	T
0.1111267122954183	0.5286903236006266	0.5624300177747450	T	T	T
0.1111155451540255	0.7282156742555081	0.5595587901203642	T	T	T
0.1110482376546498	0.9273969059524925	0.5572121493359737	T	T	T
0.2217025470741154	0.1250984392825955	0.5599320010146330	T	T	T
0.2211167242339933	0.3288050357250329	0.5611907216468695	T	T	T
0.2218218680775967	0.5291980740023832	0.5619968940089238	T	T	T
0.2220403908411319	0.7282976631870999	0.5594088208263720	T	T	T
0.2220172648805228	0.9270697817664891	0.5577339685851772	T	T	T
0.3326178684636037	0.1244731995586663	0.5622643307601081	T	T	T
0.3304377248300277	0.3287958049650979	0.5674085394509968	T	T	T
0.3310560979226535	0.5302991504771436	0.5645990014791337	T	T	T
0.3335642170000382	0.7283568260519089	0.5591901710478141	T	T	T
0.3332858432266131	0.9268078365844098	0.5584058599425213	T	T	T
0.4445648810516648	0.1247978617271087	0.5623812421159254	T	T	T
0.4413998648547551	0.3301543571943740	0.5649148585481900	T	T	T
0.4460142928311632	0.5267724034739092	0.5540948968095256	T	T	T
0.4447488062388431	0.7284684910035095	0.5592826190086381	T	T	T
0.4445994564231137	0.9269851618738143	0.5582692889713589	T	T	T
0.5555434069350562	0.1247645682857196	0.5623464681231313	T	T	T
0.5588099394598995	0.3301211269645785	0.5639736019138584	T	T	T
0.5564969204198842	0.5299838773332806	0.5584933447484201	T	T	T
0.5556345179979599	0.7286976261760487	0.5583522629043495	T	T	T
0.5557305599905210	0.9269593347927556	0.5579258152499924	T	T	T

0.6673789889318735	0.1245867305536328	0.5614012583583817	T	T	T
0.6695831252150168	0.3293328839574736	0.5651798520853017	T	T	T
0.6688983463955278	0.5306988810956491	0.5603609594274658	T	T	T
0.6669124235841036	0.7285662313325062	0.5577903023972387	T	T	T
0.6668688447660812	0.9269448382696336	0.5572936803446681	T	T	T
0.7783581682928020	0.1251561075730346	0.5593953457749735	T	T	T
0.7787905339946950	0.3294218552521454	0.5604842156657299	T	T	T
0.7789452338178177	0.5298471594408815	0.5609917605581928	T	T	T
0.7784115194294705	0.7284994103867366	0.5584671975435691	T	T	T
0.7780954596068994	0.9272005933473519	0.5571177801965403	T	T	T
0.8896415693828842	0.1258818660221891	0.5574087570642784	T	T	T
0.8910613553496016	0.3275934536039355	0.5605031455748968	T	T	T
0.8893156897383214	0.5289079442532956	0.5619096449822056	T	T	T
0.8891891544520402	0.7282869118957903	0.5593733324123156	T	T	T
0.8890810920488332	0.9275492980172094	0.5568245387214663	T	T	T
0.0553152919010279	0.0253757987510758	0.5846223708874022	T	T	T
0.0551827856692005	0.2244415081253325	0.5887127692800563	T	T	T
0.0551172611010324	0.4272016545995270	0.5915976302921349	T	T	T
0.0555889890456336	0.6272456870048557	0.5893156151111952	T	T	T
0.0555844427081226	0.8267297176929859	0.5865787771160187	T	T	T
0.1662504857732190	0.0250913838187626	0.5856823234218724	T	T	T
0.1645483891197944	0.2188499093583733	0.5912284914308207	T	T	T
0.1660894298127982	0.4272574350974133	0.5911789206193553	T	T	T
0.1664001416734286	0.6274815957435612	0.5889808921606470	T	T	T
0.1666074155962900	0.8267769684641200	0.5866620510794287	T	T	T
0.2774962110989310	0.0243801184667297	0.5868498068357676	T	T	T
0.2764681256399187	0.2202360290947312	0.5951374879456582	T	T	T
0.2751148238398513	0.4299281021984784	0.5932958474120092	T	T	T
0.2774163535003147	0.6282137710833426	0.5893143412408320	T	T	T
0.2777969269825822	0.8262950107904637	0.5866661288071749	T	T	T
0.3887765678353308	0.0243669016637885	0.5875848145800374	T	T	T
0.3883343762279766	0.2181505167308494	0.5966518285233714	T	T	T
0.3849127483941868	0.4301937103797330	0.5985470225666844	T	T	T
0.3898192609920219	0.6271069777841621	0.5884013036000425	T	T	T
0.3889294738863232	0.8264912681869893	0.5867911637479614	T	T	T
0.5000910887955567	0.0243847083276710	0.5874990740270050	T	T	T
0.5000900087187932	0.2193936713281461	0.5968894167482118	T	T	T
0.5005965028713255	0.4268431104276062	0.5840686560711003	T	T	T
0.5002921104637339	0.6278726887197974	0.5880428908971986	T	T	T
0.5001218355784468	0.8267382730267739	0.5865172415710133	T	T	T
0.6114365477194177	0.0242450122047157	0.5868772771310763	T	T	T
0.6119876872843364	0.2183204720928935	0.5959617165390234	T	T	T
0.6143082812179602	0.4305018964556880	0.5970881944543898	T	T	T
0.6115950839475208	0.6284076161297996	0.5874501881987199	T	T	T
0.6112807791550757	0.8264093863480072	0.5857870429822041	T	T	T
0.7225752592557650	0.0243834456462847	0.5860220229348873	T	T	T
0.7237015283708292	0.2205081272264848	0.5937034676486370	T	T	T
0.7251009540008989	0.4302147960311248	0.5912398960270212	T	T	T
0.7232066724209469	0.6284628151163401	0.5876584871064723	T	T	T
0.7223855579348907	0.8265183659841077	0.5856038907171289	T	T	T
0.8337657630032848	0.0251006846850265	0.5850996415913579	T	T	T
0.8351814708194635	0.2189868823199522	0.5912664538932938	T	T	T
0.8340691182542193	0.4277347226050411	0.5909399141794901	T	T	T
0.8341255652459577	0.6277794924064352	0.5882782776353144	T	T	T

```
  0.8336035348624806  0.8269187038892788  0.5861968595834843   T   T   T
  0.9447214196760952  0.0254954562708231  0.5842703233179988   T   T   T
  0.9447604852997513  0.2241045934485758  0.5874939315541057   T   T   T
  0.9447216384331477  0.4271512877797504  0.5911888627896387   T   T   T
  0.9446432304903800  0.6271904321448434  0.5889191255827427   T   T   T
  0.9446186468424194  0.8268055267894059  0.5864108414335620   T   T   T
  0.2160318810256610  0.2693965006148129  0.7215012642669699   T   T   T
  0.3285951538780254  0.2743632126496584  0.7244903277624130   T   T   T
  0.4502064030692777  0.2751975400282637  0.7244763725171824   T   T   T
  0.5516107738916100  0.2734076365289754  0.7229480307524502   T   T   T
  0.6715760700084771  0.2756055910946500  0.7243011210031145   T   T   T
  0.7831796511947841  0.2697476859732695  0.7213693059462878   T   T   T
  0.1567139211026715  0.3368838059953510  0.7463843127637235   T   T   T
  0.2710677031001902  0.3711167865112899  0.7294370861366989   T   T   T
  0.3857198447217846  0.3518586897533693  0.7471837606505879   T   T   T
  0.4986006521612491  0.3761353349777155  0.7143557278843857   T   T   T
  0.6119568659220053  0.3526152047071658  0.7461737123999649   T   T   T
  0.7264807273159029  0.3723436555311101  0.7265812215896587   T   T   T
  0.8411859175319537  0.3379075728436686  0.7449286342723554   T   T   T
  0.1124901743265764  0.4215719694828638  0.7499909278245257   T   T   T
  0.8850260395839428  0.4236946813105551  0.7483432205853492   T   T   T
  0.2193409122268978  0.4872042438748855  0.7206796887766936   T   T   T
  0.3080775196522257  0.4938348637439362  0.7392923117829542   T   T   T
  0.4523206755875885  0.4980870626581382  0.7004798168972169   T   T   T
  0.5421919044332661  0.4981509959774987  0.7091516452585458   T   T   T
  0.6893273674659399  0.4947886200667447  0.7244809793061018   T   T   T
  0.7782566793082215  0.4881095881762286  0.7278141115032869   T   T   T
  0.1708552271316174  0.4585390108508435  0.8246179448181385   T   T   T
  0.3896049728904278  0.4671904722004946  0.7938039246236774   T   T   T
  0.6119113257419295  0.4685622164100847  0.7914943793742242   T   T   T
  0.8279639983616633  0.4539951334247543  0.8300594881679333   T   T   T
  0.1603850975743830  0.2967864728119838  0.6418276726613052   T   T   T
  0.2722328121129324  0.3059390427597519  0.6497563134822706   T   T   T
  0.3881001896958431  0.3010117034781696  0.6474790906412830   T   T   T
  0.5006207432386257  0.3075659517110735  0.6410237214397168   T   T   T
  0.6126499040642256  0.3015614935607063  0.6462486776183074   T   T   T
  0.7275350531693223  0.3064572869550138  0.6487136683281960   T   T   T
  0.8383756266409920  0.2972181249245422  0.6405574152189939   T   T   T
  0.1976562664112516  0.4078870819366491  0.7328989986232239   T   T   T
  0.3380443486170551  0.4179195865286655  0.7287784583619865   T   T   T
  0.4283069305209000  0.4189541335369607  0.7124970416762105   T   T   T
  0.5683611528479600  0.4199375442182660  0.7153819626445608   T   T   T
  0.6589436083754390  0.4186886548434720  0.7219960103162408   T   T   T
  0.7996359422753494  0.4090988829249838  0.7340021240810698   T   T   T
== Embedded cluster Pd04-Te01-VTe01
   1.00000000000000
     24.6586885454000111    0.0000000000000000    0.0000000000000000
      0.0000000000000000   25.2576961516000082    0.0000000000000000
      0.0000000000000000    0.0000000000000000   30.0000000000007034
   W     Te    Pd
    56   112     4
Selective dynamics
Direct
  0.0712997413344209  0.0979235527091584  0.4912694572436215   T   T   T
```

0.2123970346905407	0.0977564908373472	0.4910576142256666	T	T	T
0.3550065109669184	0.0974141955088480	0.4920366031975019	T	T	T
0.4997841730672669	0.0976637932573296	0.4942661803584591	T	T	T
0.6428679881504995	0.0979289172654768	0.4948158190118441	T	T	T
0.7874455768898561	0.0973361659696297	0.4929829315472452	T	T	T
0.9297778327748011	0.0976108597963835	0.4919896528957438	T	T	T
0.0717503920253281	0.3486456956160587	0.4933272359160273	T	T	T
0.2119660209168054	0.3485597443899217	0.4930244623260844	T	T	T
0.3513884467321513	0.3469339010102214	0.4938389060285469	T	T	T
0.4843624019021350	0.3458718112053166	0.4995938858238841	T	T	T
0.6571867543377785	0.3471499157078664	0.4972617459573640	T	T	T
0.7922120880085481	0.3467482046625123	0.4947985097097201	T	T	T
0.9311632462872870	0.3482687219621586	0.4940831527439388	T	T	T
0.0712650845512035	0.5992152384133269	0.4953284373722307	T	T	T
0.2127336051588836	0.5993597258986406	0.4958461277542900	T	T	T
0.3557165259621646	0.5995897551606817	0.4970445752698979	T	T	T
0.4972860427459651	0.6036817896726444	0.4945695693482646	T	T	T
0.6393630069910562	0.6000185414432695	0.4922661057483111	T	T	T
0.7905415061092598	0.5997167030160567	0.4968573076626416	T	T	T
0.9305675681302755	0.5988591451514583	0.4955922689897672	T	T	T
0.0711997780680306	0.8486001837146961	0.4931696704472140	T	T	T
0.2127082404235476	0.8487427040777947	0.4930417493746549	T	T	T
0.3558839405980226	0.8491021942154451	0.4927993601861008	T	T	T
0.4996434739736378	0.8494278180653293	0.4923007310909723	T	T	T
0.6432552344184698	0.8489437190900362	0.4923500091014351	T	T	T
0.7867964518735945	0.8484499303892807	0.4929791922965874	T	T	T
0.9294116967378581	0.8485404170841356	0.4931467903878034	T	T	T
0.0005019062453398	0.0088253163722208	0.4982529171179991	T	T	T
0.1419286484958382	0.0089659916527095	0.4979504177831836	T	T	T
0.2839052970120245	0.0090107045204540	0.4983848203531124	T	T	T
0.4278770597502755	0.0093091420316196	0.4993223746197016	T	T	T
0.5713371010704793	0.0091967812762980	0.5001581377680691	T	T	T
0.7149964496260890	0.0090998798647562	0.4997967670887103	T	T	T
0.8584154588898757	0.0086518329301471	0.4991096912503138	T	T	T
0.0012303458426863	0.2586939723887788	0.4984587343600091	T	T	T
0.1419906875655824	0.2587555561905462	0.4977640750899567	T	T	T
0.2828022494154958	0.2582627779904970	0.4978462012336274	T	T	T
0.4238689651995415	0.2561885358857847	0.5006583692490131	T	T	T
0.5688203624552020	0.2589992686382723	0.5077694182389905	T	T	T
0.7206877626431354	0.2563771190926482	0.5013519127071533	T	T	T
0.8604436604009724	0.2580906968035434	0.4994139743634880	T	T	T
0.0013431679287727	0.5094819226034075	0.5012385699222367	T	T	T
0.1420754659992172	0.5099101350252365	0.5013038031906628	T	T	T
0.2834948615266883	0.5098094458717318	0.5023707073130136	T	T	T
0.4288981889930585	0.5110477336887014	0.5043198345225565	T	T	T
0.5604386947710958	0.5143992360468979	0.4995825697194105	T	T	T
0.7224454184349142	0.5112644887407710	0.5060891937328105	T	T	T
0.8604757763382066	0.5094422947826439	0.5030170344711329	T	T	T
0.0004157376035656	0.7596479415321313	0.5004470029257376	T	T	T
0.1418096577056573	0.7597371079371757	0.5004921174356729	T	T	T
0.2838566547960312	0.7602579588823448	0.5007483311586688	T	T	T
0.4274195103086909	0.7612269407141127	0.5001813582890123	T	T	T
0.5713461547246206	0.7613252016244247	0.4991241868735882	T	T	T
0.7150821512288000	0.7599683060039535	0.4996005294296809	T	T	T

0.8585895574854263	0.7598116099594170	0.5004197720508308	T	T	T
0.1418459416942585	0.0735308042480999	0.4254253356799622	T	T	T
0.2845787808531474	0.0734668464605790	0.4261016724637333	T	T	T
0.4286697270770076	0.0752025734764974	0.4279036433480771	T	T	T
0.5716835834376360	0.0753692097683493	0.4289500830889579	T	T	T
0.7139778222950225	0.0754943952706903	0.4286055050212888	T	T	T
0.8576999654782157	0.0733925156654261	0.4270893255742018	T	T	T
0.0000249046609641	0.0736658121753342	0.4259560514098065	T	T	T
0.1415718995141743	0.3256621816142333	0.4268870044054512	T	T	T
0.2823701145869399	0.3254311055373704	0.4268847798378956	T	T	T
0.4243235878222016	0.3219665087367232	0.4288933131791440	T	T	T
0.5728895004549794	0.3196164723579537	0.4359835372008586	T	T	T
0.7210134207848549	0.3207116238779858	0.4293847999106371	T	T	T
0.8610605060253587	0.3242788256664899	0.4279655730182043	T	T	T
0.0010657572033359	0.3254934114627919	0.4275267029329307	T	T	T
0.1424726555121046	0.5757123113217594	0.4297585772714930	T	T	T
0.2848038892723845	0.5756328229722646	0.4310870626972654	T	T	T
0.4253907212158203	0.5756699860272244	0.4313705527782558	T	T	T
0.5678099198312283	0.5771496870382182	0.4277069159052196	T	T	T
0.7170085016092409	0.5728358845122044	0.4327096545671749	T	T	T
0.8590527083623787	0.5741045061585488	0.4305322476065914	T	T	T
0.0007195200414556	0.5750339674867712	0.4295237922156707	T	T	T
0.1420836561045511	0.8235629196819476	0.4276974384837730	T	T	T
0.2844817755247177	0.8239943371065592	0.4279346235965183	T	T	T
0.4276153792905547	0.8250292867557153	0.4274924816789668	T	T	T
0.5715001115679954	0.8257004551319754	0.4269749227416524	T	T	T
0.7152316770766005	0.8244076744539570	0.4275320182193456	T	T	T
0.8580845274910849	0.8239304923627998	0.4278100034822737	T	T	T
0.0002314597451914	0.8238493505893395	0.4277846663322544	T	T	T
0.0710883839778397	0.1963410692632306	0.4458375982916777	T	T	T
0.2123492049321739	0.1963041888827889	0.4455497169084697	T	T	T
0.3548480413285722	0.1956748314448109	0.4469956690599419	T	T	T
0.5003642487263162	0.1976213875976495	0.4520162426042052	T	T	T
0.6422416097111610	0.1985951809820594	0.4538350301377162	T	T	T
0.7882796745593307	0.1956671191145179	0.4476964431636063	T	T	T
0.9299541657184265	0.1960329611212612	0.4466734055854849	T	T	T
0.0717830629887833	0.4476064934983859	0.4490266210125093	T	T	T
0.2128941814981664	0.4481855729543074	0.4494792275234162	T	T	T
0.3548614762050571	0.4480408411130529	0.4524163700572348	T	T	T
0.4891329228740245	0.4574416346115076	0.4416917082876410	T	T	T
0.6547114026569700	0.4485693540548583	0.4499365411603848	T	T	T
0.7891996095904079	0.4473278284310085	0.4527718679870763	T	T	T
0.9299943928068325	0.4474319292318930	0.4500671370321315	T	T	T
0.0711575595437141	0.6968718373347313	0.4493831371823658	T	T	T
0.2132445824979845	0.6970952892611845	0.4497545859272159	T	T	T
0.3557192472555860	0.6974448456721569	0.4506441699517654	T	T	T
0.4984717485663079	0.7000907357499707	0.4480821893458484	T	T	T
0.6428274424829509	0.6983866832018294	0.4480488212428339	T	T	T
0.7871823433445496	0.6967546439324014	0.4496885853728793	T	T	T
0.9291627741947907	0.6967353084952677	0.4494847625945837	T	T	T
0.0711623976512300	0.9461674659487527	0.4469502907691881	T	T	T
0.2132917132538028	0.9464923083219138	0.4469989691534894	T	T	T
0.3565440473107827	0.9473393429274642	0.4475147372834767	T	T	T
0.4999935223887367	0.9481907762010641	0.4478173397985929	T	T	T

0.6431015902660222	0.9479464458586100	0.4481410912912956	T	T	T
0.7860654988229174	0.9469905966096079	0.4479612763091932	T	T	T
0.9287609083858412	0.9463938940755489	0.4473173750818168	T	T	T
0.0010542216414980	0.1601808796430299	0.5431652716950232	T	T	T
0.1420639755541828	0.1603669385041177	0.5426804809512370	T	T	T
0.2832359523474279	0.1597585595539731	0.5426449036573177	T	T	T
0.4260224747777211	0.1580503858256667	0.5451983098792327	T	T	T
0.5707873747427096	0.1579752291755404	0.5473667761220096	T	T	T
0.7166353200418013	0.1583606347720759	0.5459241748020323	T	T	T
0.8592683071643699	0.1593869768246351	0.5439400904035625	T	T	T
0.0017862963702262	0.4108399951857979	0.5457778491186450	T	T	T
0.1420387491656224	0.4109214402366576	0.5455587498806495	T	T	T
0.2815050490278002	0.4100929862570679	0.5461414713693604	T	T	T
0.4169033912170728	0.4058151988075103	0.5529871724378436	T	T	T
0.7274606255909559	0.4067568474817658	0.5541516557924308	T	T	T
0.8617811453390820	0.4100658513058227	0.5473397577883583	T	T	T
0.0005447501949431	0.6620704578546609	0.5468653828485168	T	T	T
0.1420059531837596	0.6621572233446317	0.5467889390100812	T	T	T
0.2839504326236961	0.6624430270086391	0.5472122304432701	T	T	T
0.4267631852074210	0.6640499663280598	0.5470563455129343	T	T	T
0.5710197821349005	0.6636618566524959	0.5438713367198685	T	T	T
0.7141704343189984	0.6610960615998563	0.5438835434270610	T	T	T
0.8598183448709876	0.6624250570595497	0.5474728040806449	T	T	T
0.0002146970138989	0.9112624372148338	0.5442332348405061	T	T	T
0.1421543821144097	0.9114378897117684	0.5441395910905716	T	T	T
0.2845600918963037	0.9112381207583239	0.5438935558103540	T	T	T
0.4279745672654792	0.9108688733254732	0.5438055207884959	T	T	T
0.5715334652033860	0.9103013984051298	0.5439388840723033	T	T	T
0.7147933973223424	0.9103528281685469	0.5439939107036739	T	T	T
0.8578572695342742	0.9105561944517994	0.5443752230604789	T	T	T
0.0713890051603372	0.0333797598116326	0.5636514777481546	T	T	T
0.2128920848807934	0.0334350894245581	0.5635806928485713	T	T	T
0.3554497889708428	0.0326736764350309	0.5640199684656524	T	T	T
0.4991600926659219	0.0316745236142377	0.5653783443048042	T	T	T
0.6434318106793631	0.0316153396866213	0.5656150275175041	T	T	T
0.7869900645546478	0.0321871202459040	0.5647820833936323	T	T	T
0.9296829479075016	0.0328853959617394	0.5641682825923208	T	T	T
0.0719313927426753	0.2818449341591822	0.5642603518396162	T	T	T
0.2122113941619735	0.2814948869343850	0.5638500682883389	T	T	T
0.3517613579475441	0.2805250504489211	0.5652275215942107	T	T	T
0.4924194031132973	0.2779749488761104	0.5712606694534360	T	T	T
0.6486747358769411	0.2791413790724431	0.5695173961310754	T	T	T
0.7910267941523338	0.2808653011567570	0.5666121365433016	T	T	T
0.9313941934358726	0.2813702681899544	0.5650194686311224	T	T	T
0.0717189358064781	0.5337471418466826	0.5671666431455776	T	T	T
0.2122823984016772	0.5338727349905368	0.5676062068478829	T	T	T
0.3543346026559492	0.5341686238689262	0.5684625693511706	T	T	T
0.4973202600840906	0.5446608065672764	0.5706544241904736	T	T	T
0.6440441402300674	0.5444361401429604	0.5672524076699174	T	T	T
0.7920830121385847	0.5367013905606516	0.5704160727546188	T	T	T
0.9319178561133363	0.5340188456006174	0.5678182151670382	T	T	T
0.0710628879027551	0.7847340621942929	0.5659251139151267	T	T	T
0.2129185663780240	0.7851377251751916	0.5659298283977421	T	T	T
0.3559908603265555	0.7855873414517840	0.5654624173677140	T	T	T

```
  0.4999070133702875  0.7853119736428026  0.5646026989218265   T   T   T
  0.6432435138455669  0.7845157199772889  0.5644927745003540   T   T   T
  0.7862231567013597  0.7836236832792975  0.5651967812334459   T   T   T
  0.9292148842303123  0.7847002378775515  0.5658212842124298   T   T   T
  0.5880653767970385  0.4234822270452038  0.6177245161169349   T   T   T
  0.5696946818894760  0.3501403754948565  0.5599165300694926   T   T   T
  0.6322098865830055  0.4480823412695808  0.5398042750535925   T   T   T
  0.5138285422327606  0.4429902089824169  0.5578646941148268   T   T   T
  0.5649918310380132  0.4071976190876504  0.4801466488130081   T   T   T
== Embedded cluster Pd07-Te02-VTe02
   1.00000000000000
    24.6586885454000111    0.0000000000000000    0.0000000000000000
     0.0000000000000000   25.2576961516000082    0.0000000000000000
     0.0000000000000000    0.0000000000000000   30.0000000000007034
   W    Te   Pd
    56   112     7
Selective dynamics
Direct
  0.0700633590886485  0.0984277711727274  0.4940678290638749   T   T   T
  0.2107983279735320  0.0980169047366587  0.4945009511384918   T   T   T
  0.3528652936770766  0.0970337618024984  0.4952332388312735   T   T   T
  0.5000236734359829  0.1018604267492488  0.4997031155495529   T   T   T
  0.6472101374639875  0.0970771904683106  0.4952980223343387   T   T   T
  0.7893074162271433  0.0980609859593416  0.4945354367293408   T   T   T
  0.9300243748007374  0.0984036128822523  0.4940542476267205   T   T   T
  0.0693270556747001  0.3497656442744624  0.4984312490408543   T   T   T
  0.2067460723074761  0.3484589620734164  0.4987480726135276   T   T   T
  0.3430448986071105  0.3478123924898839  0.4991617274264442   T   T   T
  0.5000337966721341  0.3083991345475733  0.4902805050642851   T   T   T
  0.6570807898765271  0.3478251852002500  0.4992605204513831   T   T   T
  0.7934188894956842  0.3484428741461440  0.4988090516092344   T   T   T
  0.9309384246592046  0.3498215389674951  0.4985069308413759   T   T   T
  0.0702628529209026  0.6000604495466194  0.4979914762578977   T   T   T
  0.2093355231646740  0.6004603617832808  0.4986820529475021   T   T   T
  0.3625118107798063  0.6021818671808802  0.4935465760827336   T   T   T
  0.5000273672714433  0.6090215720932342  0.4905631987256467   T   T   T
  0.6375895080702173  0.6021725162608766  0.4935650658951838   T   T   T
  0.7906790056991413  0.6004462596908988  0.4986684668723010   T   T   T
  0.9297767776655671  0.6000599374757138  0.4979872422963725   T   T   T
  0.0706790676923120  0.8489011027608461  0.4933926113795978   T   T   T
  0.2122107824478792  0.8490874276987024  0.4933708568306509   T   T   T
  0.3555848759127298  0.8510479413810944  0.4931054162667631   T   T   T
  0.5000232793110770  0.8525796331916761  0.4926388769049769   T   T   T
  0.6444787028070178  0.8510627888797073  0.4931406939224105   T   T   T
  0.7878204142897456  0.8491243652313580  0.4934031753221940   T   T   T
  0.9293648536714297  0.8489120469060787  0.4934290363928271   T   T   T
  0.0000449182456936  0.0089326591893241  0.4992906287206369   T   T   T
  0.1406443848666431  0.0090286545524630  0.4997148226620125   T   T   T
  0.2824087164253442  0.0093521331337191  0.5006311614578788   T   T   T
  0.4284208796166921  0.0116379736910811  0.5020899484024255   T   T   T
  0.5716610959325048  0.0116592901888115  0.5021225155079334   T   T   T
  0.7176020146589932  0.0093954962232452  0.5006435550876014   T   T   T
  0.8594094070130289  0.0090445101915134  0.4997243832251493   T   T   T
  0.0000734009009971  0.2595896893602891  0.5022673511456541   T   T   T
```

0.1392637960743877	0.2590362674898262	0.5023149658657783	T	T	T
0.2783330646532018	0.2574306937761640	0.5037841904441069	T	T	T
0.4113584335088013	0.2549562883885187	0.5078844177453850	T	T	T
0.5887248933537647	0.2549571138822612	0.5079764831496121	T	T	T
0.7218409038480240	0.2574665780187916	0.5039266895994680	T	T	T
0.8609779946770174	0.2590989408921586	0.5024588016316924	T	T	T
0.0000835446259447	0.5108718632709393	0.5049901991251128	T	T	T
0.1400046496052419	0.5107811643964952	0.5058589173894571	T	T	T
0.2787892781544837	0.5122309079757243	0.5081390145188857	T	T	T
0.4391167025502927	0.5168333996905572	0.4956430910753641	T	T	T
0.5610269133385951	0.5168423520204267	0.4956816327818506	T	T	T
0.7213267337417568	0.5121548107326298	0.5081003201715851	T	T	T
0.8601106194902717	0.5107822826511841	0.5058584462979001	T	T	T
0.0000220559940779	0.7601888977021874	0.5010186695759153	T	T	T
0.1416007408096702	0.7603812130888323	0.5011471747829903	T	T	T
0.2843692096872273	0.7612603404619912	0.5005384100974362	T	T	T
0.4279104627380969	0.7645239260140466	0.4993916464579175	T	T	T
0.5721330500120263	0.7645332289566351	0.4994040920894572	T	T	T
0.7156872302905392	0.7612860326453159	0.5005638716713718	T	T	T
0.8584319508462561	0.7604078233599058	0.5011635658988557	T	T	T
0.1409560431371824	0.0749156138168129	0.4281995406858888	T	T	T
0.2826900648752874	0.0748596820892285	0.4289804961512833	T	T	T
0.4291738586296484	0.0798900536526769	0.4322658799880002	T	T	T
0.5709095137579768	0.0798746198600321	0.4322980081113489	T	T	T
0.7173729090938200	0.0749794043788008	0.4290112676934423	T	T	T
0.8591194461617909	0.0749179283600104	0.4281879681762376	T	T	T
0.0000498070430683	0.0750585998510413	0.4278535046865273	T	T	T
0.1385269261108032	0.3264994409388797	0.4316689041094388	T	T	T
0.2761898178177237	0.3233440996203423	0.4325889086013960	T	T	T
0.4151745221919930	0.3169284256239597	0.4319495210572604	T	T	T
0.5849645273862096	0.3169072866379801	0.4320787437264406	T	T	T
0.7238867097459076	0.3233438520406380	0.4326798406782529	T	T	T
0.8616723889770614	0.3264822946394256	0.4317569153672925	T	T	T
0.0000796021778078	0.3269616425560231	0.4316808622617893	T	T	T
0.1410963551515038	0.5742433068507070	0.4325369075284231	T	T	T
0.2835360403330604	0.5739338327478927	0.4350831652502510	T	T	T
0.4288087684153787	0.5833343154198136	0.4251794316057838	T	T	T
0.5712442097118882	0.5833452641037337	0.4252181630693864	T	T	T
0.7164715973477263	0.5738955059786712	0.4350663035999053	T	T	T
0.8589235202390980	0.5742347257141809	0.4325385297213359	T	T	T
0.0000105975153901	0.5748869746366712	0.4323195124228559	T	T	T
0.1417101556722827	0.8237525972040762	0.4279530425971187	T	T	T
0.2841975562215188	0.8255408127823456	0.4281465373340014	T	T	T
0.4278467970313099	0.8294086294762928	0.4275570583156346	T	T	T
0.5722482939942695	0.8294464402483125	0.4275911089002655	T	T	T
0.7158488501607478	0.8255652839325851	0.4281629246802937	T	T	T
0.8583229327054133	0.8238283359105805	0.4279875114290446	T	T	T
0.9999983150435231	0.8239073648893515	0.4279633378104973	T	T	T
0.0697094308739934	0.1971278114214478	0.4491592600370289	T	T	T
0.2098805057936213	0.1970020744249613	0.4497975794574650	T	T	T
0.3502973596846660	0.1959979599905258	0.4504341464632867	T	T	T
0.5000123802076974	0.2041823023671476	0.4563554961788401	T	T	T
0.6498234906091302	0.1960089011311320	0.4505317355750287	T	T	T
0.7903168877659981	0.1971303104348347	0.4500002958765804	T	T	T

0.9303924891008160	0.1971238099837842	0.4491976125246941	T	T	T
0.0706365903436690	0.4483282591839210	0.4536621933393089	T	T	T
0.2117459970195889	0.4488727169620065	0.4556188740405991	T	T	T
0.3515279536056081	0.4509616025296456	0.4543097666701240	T	T	T
0.5000802979635618	0.4690744746942055	0.4298815840109957	T	T	T
0.6485840387223650	0.4508919405988946	0.4542778157326041	T	T	T
0.7885166978252901	0.4488032688813006	0.4555640387736306	T	T	T
0.9295686705535140	0.4483200371548444	0.4536886955235139	T	T	T
0.0710502601811001	0.6967262550218120	0.4506750770465289	T	T	T
0.2127634913146157	0.6969423396551333	0.4508655018643970	T	T	T
0.3564247003421663	0.7005654450260023	0.4491867594663546	T	T	T
0.5000174520658414	0.7061006146187429	0.4462963750668579	T	T	T
0.6436301451677129	0.7005978733520579	0.4492194830094577	T	T	T
0.7872752230684866	0.6969492090485924	0.4508668468883245	T	T	T
0.9289658076969771	0.6967343862812289	0.4506859953443066	T	T	T
0.0709412067665146	0.9467008288559898	0.4474783021061230	T	T	T
0.2127149004466763	0.9474073766941433	0.4481199441891338	T	T	T
0.3565724613736978	0.9500697475384833	0.4496324598536879	T	T	T
0.5000392704779526	0.9519359024655929	0.4493030734938613	T	T	T
0.6434389902030391	0.9500620168035389	0.4496397689657176	T	T	T
0.7873010314007562	0.9474537000540096	0.4481781164608741	T	T	T
0.9291138092248697	0.9467009382588171	0.4475381774271071	T	T	T
0.0000511318775559	0.1606088783360967	0.5464961964588683	T	T	T
0.1402211210505776	0.1600819312124107	0.5466090339427222	T	T	T
0.2813350819667182	0.1588473264084866	0.5474247275167298	T	T	T
0.4217049314073891	0.1567860178473008	0.5507877845293664	T	T	T
0.5783368670885523	0.1567778677476853	0.5508177876957749	T	T	T
0.7188669758961415	0.1588666988919550	0.5475613112044494	T	T	T
0.8600342590953168	0.1600316373687252	0.5466787950242160	T	T	T
0.0001966879015296	0.4126813052324630	0.5507074068590251	T	T	T
0.1395319289806277	0.4121229766926537	0.5515397799096139	T	T	T
0.2754577943926849	0.4104528894994201	0.5561371743558277	T	T	T
0.7246629436266189	0.4104518848528082	0.5561785735202882	T	T	T
0.8606033252332255	0.4121312271856252	0.5515571167988852	T	T	T
0.0000132311197509	0.6634476128944026	0.5486665104959060	T	T	T
0.1411579613522701	0.6636610853928135	0.5494235702728973	T	T	T
0.2866895248052683	0.6620479534159674	0.5454370797268915	T	T	T
0.4295110290785485	0.6671993483888442	0.5447698352615292	T	T	T
0.5705306587340759	0.6672123859141299	0.5447772279489768	T	T	T
0.7133735097532918	0.6620695061267199	0.5454145836634227	T	T	T
0.8588949565530390	0.6636641643433568	0.5494089121581708	T	T	T
0.0000352120253740	0.9111946253964656	0.5447197068481502	T	T	T
0.1416301186328044	0.9109906139574322	0.5448295603871657	T	T	T
0.2837327958699272	0.9109507119032807	0.5449813811356428	T	T	T
0.4279155192742879	0.9122410996231535	0.5449120699900557	T	T	T
0.5720922134111269	0.9122409319756634	0.5449190113258313	T	T	T
0.7163125687494721	0.9110040383576495	0.5450222633782439	T	T	T
0.8584183843191675	0.9110349366323419	0.5448730802986811	T	T	T
0.0702488944371452	0.0323485245082001	0.5654906108670906	T	T	T
0.2111870572720100	0.0322012064943698	0.5660086897286178	T	T	T
0.3543187976781865	0.0313963096720017	0.5665772583678422	T	T	T
0.5000054943279589	0.0326025991467891	0.5681953404499689	T	T	T
0.6457505624667276	0.0314182645133037	0.5666214763684364	T	T	T
0.7888345839189240	0.0322027237510067	0.5659997503843215	T	T	T

```
  0.9298250507822136  0.0323397328281167  0.5654770287610648   T   T   T
  0.0697128140032259  0.2821272381570840  0.5689907053147949   T   T   T
  0.2083646132435046  0.2817313892764426  0.5695847974483984   T   T   T
  0.3459201918762647  0.2858063765172705  0.5738868720632461   T   T   T
  0.5000516293653826  0.2620358659547445  0.5717742374808341   T   T   T
  0.6541882297496201  0.2858289005041183  0.5739855695551108   T   T   T
  0.7918442513474099  0.2817674508219171  0.5697131763342015   T   T   T
  0.9305903731735622  0.2822139257060169  0.5690973875198813   T   T   T
  0.0697247684569095  0.5365046169841863  0.5711235719235923   T   T   T
  0.2095541608790821  0.5385967381536857  0.5726162770122358   T   T   T
  0.3590768674594862  0.5429525758028074  0.5671439790286660   T   T   T
  0.5000182306997918  0.5477790092334690  0.5653207683906998   T   T   T
  0.6410835692782572  0.5429251535739759  0.5671470037945852   T   T   T
  0.7905251113760898  0.5385687534986520  0.5726045396384533   T   T   T
  0.9304069938922256  0.5365033068561251  0.5711089169385076   T   T   T
  0.0708752539952046  0.7853964251020642  0.5663533081061729   T   T   T
  0.2134151442339467  0.7848072210800927  0.5660315741388461   T   T   T
  0.3561721320365111  0.7865764136817471  0.5651773235370469   T   T   T
  0.5000087241312494  0.7878449439285623  0.5644099986257305   T   T   T
  0.6438652517780452  0.7865748817109497  0.5652008909447122   T   T   T
  0.7866192486490953  0.7848530930855949  0.5660614512832153   T   T   T
  0.9291947196717329  0.7853622750450837  0.5663580908156384   T   T   T
  0.4288012687374980  0.4234228118705481  0.6149309959181695   T   T   T
  0.5712261636316753  0.4234150399083508  0.6149182152468352   T   T   T
  0.3755752412060710  0.4444388964981750  0.5418277522760908   T   T   T
  0.5000311233931206  0.4414433455347313  0.5519072270694274   T   T   T
  0.6245389918012423  0.4444283466341293  0.5418569752836723   T   T   T
  0.4380008750872361  0.3461193975165467  0.5579620698696621   T   T   T
  0.5620625247629388  0.3461187533681484  0.5579892102002825   T   T   T
  0.4445545608582255  0.4020259531939186  0.4778334670438286   T   T   T
  0.5555693136645993  0.4020355831742095  0.4778724602702988   T   T   T
== Embedded cluster Pd10-Te03-VTe03
   1.00000000000000
     24.6586885454000111    0.0000000000000000    0.0000000000000000
      0.0000000000000000   25.2576961516000082    0.0000000000000000
      0.0000000000000000    0.0000000000000000   30.0000000000007034
   W    Te   Pd
    56   112    10
Selective dynamics
Direct
  0.0680664072728176  0.0983104665865370  0.4964362149680788   T   T   T
  0.2090385845568951  0.0981046456061839  0.4977583824661981   T   T   T
  0.3468901748466916  0.0954788993785870  0.4983211090498698   T   T   T
  0.5103342796584237  0.1005094534761697  0.5022981057716733   T   T   T
  0.6497674886875653  0.0966362493206197  0.4962909235069534   T   T   T
  0.7892624347151288  0.0977376468684952  0.4953643969885359   T   T   T
  0.9287813502834324  0.0982107061510433  0.4953320855680223   T   T   T
  0.0655928515360450  0.3493072150437046  0.5002365652512644   T   T   T
  0.1991495992381254  0.3488312979040599  0.5011021791211746   T   T   T
  0.3594714103060533  0.3365664691816488  0.5029079622486125   T   T   T
  0.4990328517966307  0.3067412653006906  0.4899969728125229   T   T   T
  0.6567566339397192  0.3477731474620278  0.4998475424098461   T   T   T
  0.7923995677388608  0.3486054613309005  0.5000624311959335   T   T   T
  0.9289674197042872  0.3501631490651237  0.5002558925383344   T   T   T
```

0.0656354015242447	0.6006904363413488	0.5009811497856308	T	T	T
0.2218558444760008	0.6022648601708372	0.4947978091179184	T	T	T
0.3598102016429496	0.6091430869936365	0.4914343818299656	T	T	T
0.4980302284561267	0.6095466460731127	0.4914254202093142	T	T	T
0.6383285448216405	0.6020552274623657	0.4945479635620113	T	T	T
0.7890645555014870	0.6001827781387131	0.4996865027626290	T	T	T
0.9280559442904565	0.6000303329283155	0.4999541961031585	T	T	T
0.0702119836447023	0.8486743178591174	0.4949408605516962	T	T	T
0.2120162343165944	0.8502104892857222	0.4946283257708531	T	T	T
0.3564460856583176	0.8540576069772646	0.4952244024273477	T	T	T
0.5009948380336903	0.8536896902104559	0.4947026440571440	T	T	T
0.6451692950401592	0.8503089588873786	0.4942724735218437	T	T	T
0.7877404334415614	0.8486194013115119	0.4944187636361940	T	T	T
0.9289859043066098	0.8486465895300899	0.4946581643426225	T	T	T
0.9988925388424338	0.0087131322944047	0.5010732911580013	T	T	T
0.1392453730444147	0.0092971429911336	0.5022879419029388	T	T	T
0.2784372935477629	0.0091468823505133	0.5033579743121673	T	T	T
0.4327102353758580	0.0142794359589092	0.5071385453819164	T	T	T
0.5757013991084980	0.0104092413376623	0.5037760159631426	T	T	T
0.7183095185440953	0.0084756938013101	0.5011294346680358	T	T	T
0.8587736737038307	0.0085677231453797	0.5005026190359843	T	T	T
0.9977786604416204	0.2594912762327731	0.5037302126047599	T	T	T
0.1362425146055657	0.2580666347444071	0.5052335142651799	T	T	T
0.2803517794547948	0.2576630353711832	0.5115402850943580	T	T	T
0.4143051462273704	0.2475968578918504	0.5074967252736601	T	T	T
0.5900556264674569	0.2553287057874211	0.5087852434447369	T	T	T
0.7214999850850731	0.2576024840502425	0.5048796487278442	T	T	T
0.8594533040620199	0.2590685359739506	0.5035660141234278	T	T	T
0.9979624819934713	0.5110364549551868	0.5080914527132437	T	T	T
0.1366138375240822	0.5136005168495834	0.5102160850750874	T	T	T
0.2967819852483680	0.5168122894780260	0.4971904706602543	T	T	T
0.4307107886701981	0.5195499887135048	0.4936821276295613	T	T	T
0.5602357576442640	0.5170475518491854	0.4957429158805548	T	T	T
0.7195912089610047	0.5118535864820282	0.5084205370980179	T	T	T
0.8584545781443335	0.5108133325776472	0.5073534622273360	T	T	T
0.9992151311547800	0.7602922629523787	0.5027944742819822	T	T	T
0.1418701431197950	0.7604488200198047	0.5017843438882400	T	T	T
0.2850754455542081	0.7647583667608563	0.5010785754182768	T	T	T
0.4285443959646374	0.7669756663076613	0.5006218260830734	T	T	T
0.5720363769719713	0.7646372927670886	0.5009854682336861	T	T	T
0.7158001309925851	0.7607755016683402	0.5017842317535266	T	T	T
0.8580240928045704	0.7601360144854692	0.5024151381324757	T	T	T
0.1395018904618181	0.0755439004320601	0.4309212610304949	T	T	T
0.2797543134742749	0.0731995595256610	0.4308688002478042	T	T	T
0.4307194266578674	0.0815278875811676	0.4386969216376698	T	T	T
0.5744415369706648	0.0770908804831489	0.4325668155132836	T	T	T
0.7183936391625364	0.0740753975719009	0.4293970825021807	T	T	T
0.8588675304043087	0.0745452208186405	0.4289263979198606	T	T	T
0.9992336984557862	0.0746232674264464	0.4295248359071167	T	T	T
0.1348731351875047	0.3229077604010519	0.4334333010567116	T	T	T
0.2781257321173599	0.3186603567191897	0.4382636313115532	T	T	T
0.4190806556386257	0.3127817370066098	0.4287333534475520	T	T	T
0.5843044946855059	0.3166889005936566	0.4326464750810892	T	T	T
0.7234589879896215	0.3232016992266977	0.4333757256136768	T	T	T

0.8605445127263235	0.3267153183386199	0.4330142046530237	T	T	T
0.9975038664076212	0.3270248129689073	0.4331167278825793	T	T	T
0.1407732719190289	0.5754396609943362	0.4374637297549226	T	T	T
0.2878641881932753	0.5822707761087583	0.4264884636675980	T	T	T
0.4288333132380207	0.5893902347104680	0.4243566457614100	T	T	T
0.5708638561661241	0.5850867981795721	0.4266604082860982	T	T	T
0.7160686791882779	0.5738314531616491	0.4352770546185568	T	T	T
0.8583070713757711	0.5743438340840885	0.4340237992726738	T	T	T
0.9986334516087393	0.5740786126732209	0.4344273981071928	T	T	T
0.1414694781344513	0.8244838502386450	0.4293349151268228	T	T	T
0.2851938141517358	0.8303952762773426	0.4295219065103247	T	T	T
0.4283553817391061	0.8328876675875939	0.4293830149014291	T	T	T
0.5722471003689400	0.8296317869074210	0.4290904753713732	T	T	T
0.7160470406547237	0.8247656692136532	0.4291120354962064	T	T	T
0.8583665455179315	0.8233183602180848	0.4290707603835520	T	T	T
0.9998367037341014	0.8234138921112880	0.4293934807174385	T	T	T
0.0679704142827493	0.1966242834610375	0.4513656014058481	T	T	T
0.2104061821001896	0.1975491301956470	0.4554580627747903	T	T	T
0.3473029328164329	0.1928675016479186	0.4536268552392626	T	T	T
0.5041368366418827	0.2013452679442352	0.4577961468895344	T	T	T
0.6506997528252284	0.1956268283472511	0.4513818293807818	T	T	T
0.7897655572260815	0.1966705333261061	0.4507501007138811	T	T	T
0.9288541727810122	0.1969054942265142	0.4502973586439559	T	T	T
0.0688977307617441	0.4492086250201988	0.4576057914186428	T	T	T
0.2018229704629600	0.4515343652282409	0.4529386748001482	T	T	T
0.3615913078362722	0.4663548234444356	0.4347714474921856	T	T	T
0.5001810119498049	0.4706075449291574	0.4309870331603616	T	T	T
0.6472725109578229	0.4503400450299803	0.4539421522787970	T	T	T
0.7876011494113742	0.4485917054654196	0.4564619407902373	T	T	T
0.9286157054406680	0.4486041527389952	0.4556291538570166	T	T	T
0.0697992900216263	0.6964921887787779	0.4525491773898423	T	T	T
0.2147144023654904	0.7004501238937610	0.4501765381678368	T	T	T
0.3571498735637932	0.7070969436154619	0.4476138026138320	T	T	T
0.5000710102973094	0.7073560082026251	0.4476635294682302	T	T	T
0.6435216327759405	0.7006930868018690	0.4504240712355244	T	T	T
0.7870980838305324	0.6967593236996680	0.4519046450747906	T	T	T
0.9287827761868570	0.6965694037971687	0.4521811027682716	T	T	T
0.0703622226744496	0.9467489649595704	0.4494526724337926	T	T	T
0.2109948011430913	0.9484044403087244	0.4495331905069571	T	T	T
0.3576121808343500	0.9540332721262360	0.4538318299303062	T	T	T
0.5006711222513850	0.9533410869557192	0.4520191113458244	T	T	T
0.6446361483580384	0.9487095674939161	0.4499743796297094	T	T	T
0.7878926077697563	0.9464635640459743	0.4485940979710716	T	T	T
0.9292441321496236	0.9462399478945500	0.4486748246497237	T	T	T
0.9978131718951605	0.1604734644138623	0.5482581629774924	T	T	T
0.1381635984982076	0.1593635842815118	0.5499548182561912	T	T	T
0.2808543217139160	0.1565610740160726	0.5521744671158546	T	T	T
0.4255096843430761	0.1496566287040653	0.5511756401856910	T	T	T
0.5831089077565623	0.1570110690387193	0.5535520550612888	T	T	T
0.7196267946131564	0.1589672317972922	0.5489897828237379	T	T	T
0.8588403891004580	0.1601042406069510	0.5477948464252357	T	T	T
0.9974429704299553	0.4124574898854373	0.5534543575684161	T	T	T
0.1328738460950501	0.4102242764316580	0.5580615171431726	T	T	T
0.7228987519820516	0.4105003726815347	0.5566693673494837	T	T	T

0.8587880415938395	0.4124278752082299	0.5532387709705248	T	T	T
0.9981357181252620	0.6639602818192016	0.5516411768364666	T	T	T
0.1445610979334683	0.6613651966669414	0.5462549032008595	T	T	T
0.2882800374102744	0.6666226559909991	0.5460201894225979	T	T	T
0.4287092145679006	0.6694525844881980	0.5451418853283997	T	T	T
0.5701857174567593	0.6664487389374664	0.5455589966529300	T	T	T
0.7131789900724319	0.6620757899486301	0.5468403872398360	T	T	T
0.8576841157758570	0.6637653565490985	0.5506386942840394	T	T	T
0.9995148860109518	0.9107696931824989	0.5463805758140298	T	T	T
0.1408501081271578	0.9107952070750756	0.5467190582265430	T	T	T
0.2826948061435011	0.9115847361193340	0.5475308445370158	T	T	T
0.4295557454134711	0.9138724268777090	0.5473494656359872	T	T	T
0.5742537614756932	0.9118632658465580	0.5467644755618937	T	T	T
0.7168364857916327	0.9106385355628630	0.5459499166133214	T	T	T
0.8581992164653337	0.9108455206448989	0.5458663981144080	T	T	T
0.0683968655227841	0.0321150167971939	0.5678518148669036	T	T	T
0.2089597848564652	0.0323434959389520	0.5694562598950607	T	T	T
0.3534442471551949	0.0303620395773097	0.5690709783831565	T	T	T
0.5059068446018284	0.0324696442717561	0.5718996327079120	T	T	T
0.6487344218814423	0.0315418674459438	0.5682346915948554	T	T	T
0.7888607556815987	0.0316933106226207	0.5668920984261600	T	T	T
0.9284972981019303	0.0321487705630825	0.5668422084104565	T	T	T
0.0663882055049582	0.2821194070466284	0.5711105511130682	T	T	T
0.2047303979391673	0.2800448275685259	0.5743556691682734	T	T	T
0.3548621599319111	0.2757062858222002	0.5805475143171379	T	T	T
0.5043113140561460	0.2591525251082517	0.5714017077971713	T	T	T
0.6541566162371181	0.2874975224692980	0.5750763809124719	T	T	T
0.7907451890285987	0.2819881414232996	0.5711095221269710	T	T	T
0.9284075241746129	0.2818310315587250	0.5705637185999309	T	T	T
0.0672044932842219	0.5389750851201779	0.5751475966090280	T	T	T
0.2174024300621722	0.5448259912620598	0.5691441441597405	T	T	T
0.3618539991350579	0.5442681772953295	0.5636346694805598	T	T	T
0.4973358491301572	0.5454004349261240	0.5646502128440194	T	T	T
0.6402128714513537	0.5426697916456922	0.5681331695455127	T	T	T
0.7881790657841959	0.5382246986279099	0.5736112650938298	T	T	T
0.9276523470638137	0.5370350049330949	0.5734196371185326	T	T	T
0.0711648483922442	0.7842727465389079	0.5674906692945818	T	T	T
0.2131399280764718	0.7854509481602585	0.5667912186799596	T	T	T
0.3566748523716851	0.7886008065512006	0.5663949040662621	T	T	T
0.5005332636388332	0.7887237129472211	0.5663204319898023	T	T	T
0.6441277315300016	0.7862370952527229	0.5665959910880506	T	T	T
0.7864739763672486	0.7848586804189958	0.5673448901081686	T	T	T
0.9284157165496876	0.7856436625876757	0.5678365842713485	T	T	T
0.2986600850808130	0.4308847825058473	0.6164262941978566	T	T	T
0.4215768866018047	0.4223382722973756	0.6180417495916505	T	T	T
0.5659199174162781	0.4259474290479679	0.6126642360251048	T	T	T
0.2320355753045870	0.4478858792541212	0.5440300753772946	T	T	T
0.3659725272906387	0.4341417085755022	0.5430815235539638	T	T	T
0.4919414854080403	0.4420425414249581	0.5481499597832642	T	T	T
0.6224222652746482	0.4451139102604855	0.5412572076335240	T	T	T
0.2834937999367838	0.3527124915547752	0.5626986230729247	T	T	T
0.4419279202745108	0.3453863531948076	0.5623824412654054	T	T	T
0.5603915238516654	0.3475356259098665	0.5574793110051062	T	T	T
0.2937569077558982	0.4099115706732908	0.4781133890915223	T	T	T

```
  0.4368954188365001  0.4032976106828086  0.4755104022051277   T   T   T
  0.5528071883156830  0.4020114113544510  0.4778816914154650   T   T   T
== Embedded cluster Pd13-Te04-VTe04
   1.00000000000000
    24.6586885454000111    0.0000000000000000    0.0000000000000000
     0.0000000000000000   25.2576961516000082    0.0000000000000000
     0.0000000000000000    0.0000000000000000   30.0000000000007034
   W    Te   Pd
    56   112    13
Selective dynamics
Direct
  0.0684874192833047  0.0976571472173669  0.4994949180516463   T   T   T
  0.2083972954313702  0.0958819109874425  0.4999996580309843   T   T   T
  0.3484447494415015  0.0991784822455377  0.5034979298123704   T   T   T
  0.5123853229272848  0.0949084963455758  0.4987599907549627   T   T   T
  0.6485708869707054  0.0979999200029433  0.4984186801990519   T   T   T
  0.7882603982947644  0.0981947906799106  0.4978519841527399   T   T   T
  0.9273280233312876  0.0979691648928785  0.4981615956966675   T   T   T
  0.0570412256236979  0.3491422872117419  0.5020719353077561   T   T   T
  0.2155151442872886  0.3376485922512263  0.5035540645171048   T   T   T
  0.3573472412207646  0.3055135793857708  0.4905034214352120   T   T   T
  0.4990387547801974  0.3365502943528029  0.5022855231470595   T   T   T
  0.6572412888848981  0.3491153146307779  0.5010125554155187   T   T   T
  0.7896591964722176  0.3493892226659847  0.5008593569096085   T   T   T
  0.9248539655542388  0.3492947339383906  0.5013799906036537   T   T   T
  0.0791063314201286  0.6016754282730248  0.4960654617548412   T   T   T
  0.2176714238319891  0.6091593524301760  0.4923276021029085   T   T   T
  0.3577132974013595  0.6102504071270866  0.4920355931012931   T   T   T
  0.4968527662299804  0.6092943022923405  0.4918487383848945   T   T   T
  0.6354912001806816  0.6020650263940671  0.4956415491968530   T   T   T
  0.7893607164320138  0.6007423392443172  0.5032783833599472   T   T   T
  0.9245566035893747  0.6006407490222950  0.5036218702156386   T   T   T
  0.0695681068729403  0.8497779374003842  0.4968266758390083   T   T   T
  0.2122864907767086  0.8521571255279929  0.4957719298775959   T   T   T
  0.3571854150079127  0.8543126884948545  0.4955609409350771   T   T   T
  0.5014979060063409  0.8540509603291194  0.4960445447884819   T   T   T
  0.6450648039380434  0.8500502665180932  0.4958840467794564   T   T   T
  0.7864938781975761  0.8486145278156527  0.4970233970586855   T   T   T
  0.9279448891207113  0.8485853741441916  0.4975156399795221   T   T   T
  0.9980249756988143  0.0089552898045031  0.5044058980099311   T   T   T
  0.1390665580361884  0.0086368799324630  0.5050842684907880   T   T   T
  0.2822655497889107  0.0100303776474229  0.5058125291133304   T   T   T
  0.4260009251744359  0.0139797080102750  0.5082470112341022   T   T   T
  0.5801541773590614  0.0086616690240090  0.5039059629878325   T   T   T
  0.7178081283443946  0.0090703314451089  0.5035720536640137   T   T   T
  0.8575273253012962  0.0086693006008796  0.5037740664045982   T   T   T
  0.9951139609933014  0.2579074101314205  0.5060637076517859   T   T   T
  0.1379472535744532  0.2578310891274850  0.5126208649639330   T   T   T
  0.2707092755173656  0.2492161576900333  0.5092762265015608   T   T   T
  0.4435940120009676  0.2476771668537309  0.5077865279076212   T   T   T
  0.5775062201270744  0.2579190398495130  0.5115059909986098   T   T   T
  0.7199951236659865  0.2582186581636416  0.5054258964074370   T   T   T
  0.8575785062269562  0.2591743874398629  0.5046128944080909   T   T   T
  0.9942608465081249  0.5132999574278806  0.5117749246674825   T   T   T
```

0.1554494958829846	0.5168714451338945	0.4981015450686434	T	T	T
0.2898568023961987	0.5203302568695981	0.4943071364244012	T	T	T
0.4247016591071350	0.5201116352479440	0.4939164077397740	T	T	T
0.5590440799216663	0.5168477463302955	0.4974585737782800	T	T	T
0.7194796579741762	0.5133947056346168	0.5109864425043641	T	T	T
0.8570976247048221	0.5112723340258080	0.5111232030107472	T	T	T
0.9995468802530496	0.7602895417936800	0.5043420518171534	T	T	T
0.1417559911476065	0.7635287443187125	0.5024231740785408	T	T	T
0.2857576379637169	0.7667157499721268	0.5013131921214040	T	T	T
0.4292397728091344	0.7674433695086177	0.5013149856491903	T	T	T
0.5722905593097718	0.7644676977569719	0.5019069457910490	T	T	T
0.7147638807294238	0.7604693842535734	0.5036083576130530	T	T	T
0.8572072728693774	0.7605717683309138	0.5056221369028915	T	T	T
0.1388160011706835	0.0733453220933586	0.4333421398326956	T	T	T
0.2825793965684436	0.0771073173200061	0.4346896058846266	T	T	T
0.4279762819995555	0.0804470323963292	0.4394590581826473	T	T	T
0.5788285949820952	0.0722606610919910	0.4310920002479021	T	T	T
0.7179473189741314	0.0748105651885964	0.4318216008232886	T	T	T
0.8580542469390512	0.0736304193127319	0.4317494396133542	T	T	T
0.9986244278007930	0.0748655767368432	0.4328049472062465	T	T	T
0.1352664158211013	0.3188793269779547	0.4390315734100281	T	T	T
0.2760896169389701	0.3133447751938858	0.4304208676569462	T	T	T
0.4377209872274896	0.3119018821972412	0.4294623463380296	T	T	T
0.5790575676578543	0.3185276606483233	0.4376711479356528	T	T	T
0.7212171732590896	0.3228710082410923	0.4334374151214559	T	T	T
0.8574922515388381	0.3263768173641194	0.4337265251228001	T	T	T
0.9935252892227020	0.3227106598181371	0.4342084777015698	T	T	T
0.1454843867160512	0.5824198161190289	0.4275839768325392	T	T	T
0.2871950349518207	0.5903787575195129	0.4255666929620521	T	T	T
0.4275005258147663	0.5901442289352762	0.4251183498277454	T	T	T
0.5691461629979858	0.5827698763012128	0.4271457872448169	T	T	T
0.7163307825519212	0.5757714754015089	0.4383064434254237	T	T	T
0.8571801985986318	0.5740038070131321	0.4368451805181280	T	T	T
0.9979987935816491	0.5752482930311973	0.4389169395632226	T	T	T
0.1407386966618144	0.8282259706859663	0.4304904864982506	T	T	T
0.2850473781475696	0.8324769894272189	0.4299911208182432	T	T	T
0.4295015890518876	0.8333512567030188	0.4300462171129908	T	T	T
0.5725878772480281	0.8301959362115161	0.4303384666885960	T	T	T
0.7158960480155058	0.8243431764676311	0.4309668139812136	T	T	T
0.8574532641707926	0.8233218435051928	0.4319568864620125	T	T	T
0.9986540312879396	0.8241010313054640	0.4317483159524275	T	T	T
0.0685930997943077	0.1969005068376001	0.4565863171340682	T	T	T
0.2052023896577847	0.1933290116427817	0.4550857054201488	T	T	T
0.3558243307091426	0.1989893568835068	0.4581931764313006	T	T	T
0.5110166847894486	0.1921785722766984	0.4541695941552382	T	T	T
0.6471068833019449	0.1969467594637646	0.4553402220417326	T	T	T
0.7882518865177364	0.1959296709210960	0.4519308580827647	T	T	T
0.9270923555452963	0.1958210451188929	0.4522337773194779	T	T	T
0.0590708996143965	0.4522568781107945	0.4536360536804915	T	T	T
0.2201990264094921	0.4665146540317536	0.4358732590040995	T	T	T
0.3570537525675382	0.4702949925281391	0.4329654978884836	T	T	T
0.4942160298352720	0.4664573605674995	0.4352843324106088	T	T	T
0.6548827193499742	0.4524765715205351	0.4527855278558176	T	T	T
0.7878771613566330	0.4497993806135164	0.4586834554683527	T	T	T

0.9265809342274963	0.4495181146175917	0.4593052594759422	T	T	T
0.0712003398043700	0.6998402956689784	0.4519160643114613	T	T	T
0.2141375026561048	0.7068071545797072	0.4485308489927512	T	T	T
0.3576075451954615	0.7084982428147427	0.4485535259574412	T	T	T
0.5001177340981608	0.7073768255664126	0.4480918077350063	T	T	T
0.6427505367885414	0.7003039627435612	0.4513308266314467	T	T	T
0.7870075447046155	0.6966098804649345	0.4549408081704474	T	T	T
0.9274257065268452	0.6964588023205373	0.4553019280780666	T	T	T
0.0690581204587995	0.9476566003610974	0.4521516851097365	T	T	T
0.2120438134574302	0.9500396903346717	0.4523535576826800	T	T	T
0.3571914533939811	0.9538480741472377	0.4533633663241853	T	T	T
0.5007125504190629	0.9539449975949913	0.4545053932297434	T	T	T
0.6469948246362400	0.9477646114007412	0.4501653004706443	T	T	T
0.7871931523768522	0.9465558475382956	0.4513296298821298	T	T	T
0.9281642094138653	0.9464463043813151	0.4521033921053610	T	T	T
0.9970484413400837	0.1596708639166934	0.5512166205589992	T	T	T
0.1386679214058764	0.1571677894747071	0.5533603902198453	T	T	T
0.2777897180158630	0.1534408208499721	0.5573434852675077	T	T	T
0.4343364459987031	0.1486700358723504	0.5510197570995317	T	T	T
0.5769996255179964	0.1568169878628253	0.5528121425908703	T	T	T
0.7180844584760883	0.1598870005462611	0.5508125051030262	T	T	T
0.8575977563914338	0.1606055229504178	0.5500007368824076	T	T	T
0.9914353835239251	0.4102077279117153	0.5596360853101282	T	T	T
0.7223324163354774	0.4102104300990975	0.5585823786573041	T	T	T
0.8568821538445681	0.4114041774662926	0.5554320876474110	T	T	T
0.0027034803130042	0.6612733773828997	0.5490092047661035	T	T	T
0.1459451716737668	0.6658152928055030	0.5473136324111398	T	T	T
0.2869695595020114	0.6692699524641366	0.5455916721228899	T	T	T
0.4282529566782885	0.6697615861691252	0.5454490194619263	T	T	T
0.5683286070724655	0.6665093106164051	0.5466623642408270	T	T	T
0.7113317392758476	0.6617549087757052	0.5484812979256282	T	T	T
0.8569251007596387	0.6640768370092552	0.5553854772667003	T	T	T
0.9986416392411884	0.9104942437039774	0.5492060886114423	T	T	T
0.1406549080630851	0.9105491812197422	0.5490634141394831	T	T	T
0.2840931142019921	0.9118114568371767	0.5485765239268323	T	T	T
0.4288080570045541	0.9137449183320113	0.5484288119436529	T	T	T
0.5749177792292304	0.9117180902172651	0.5486617769905321	T	T	T
0.7158327823722412	0.9108043162126960	0.5483290929830783	T	T	T
0.8570558274651899	0.9104955851499154	0.5488814375077694	T	T	T
0.0681621044166746	0.0316385456292880	0.5708551215066776	T	T	T
0.2100801189686256	0.0300700027727207	0.5714984788560248	T	T	T
0.3532667917403179	0.0316586257513413	0.5733356366189956	T	T	T
0.5055333392118200	0.0304085687539098	0.5698805992128968	T	T	T
0.6482628264276437	0.0327642205608715	0.5705493925077674	T	T	T
0.7875569814747653	0.0327397061322801	0.5697706353646088	T	T	T
0.9274022189062954	0.0325472857229803	0.5700164237237393	T	T	T
0.0626903885409472	0.2807949873634177	0.5755146156868921	T	T	T
0.2116099184781882	0.2779554045998646	0.5821960846227721	T	T	T
0.3584093643543595	0.2566959021390783	0.5719658349182309	T	T	T
0.5032191982772640	0.2763231167994311	0.5805937043419773	T	T	T
0.6521567890097775	0.2807264226907561	0.5745771693357237	T	T	T
0.7890387153957070	0.2819436770197927	0.5717756563521033	T	T	T
0.9257011559663404	0.2818130623991786	0.5721297761810421	T	T	T
0.0756752923978981	0.5447037394352817	0.5704105860123723	T	T	T

```
  0.2209312592579180  0.5447914340732423  0.5643605759276761   T   T   T
  0.3574538646881608  0.5433304494815766  0.5640970333654043   T   T   T
  0.4936346303920172  0.5446692889362472  0.5637605784335923   T   T   T
  0.6383221410223626  0.5445934377105991  0.5697987341166016   T   T   T
  0.7869391487101807  0.5388349877760678  0.5774115657313904   T   T   T
  0.9264782034135827  0.5390361389965399  0.5779000346799809   T   T   T
  0.0711670667103825  0.7851817594083971  0.5689914261137595   T   T   T
  0.2139372015892704  0.7872093829771292  0.5673602951732554   T   T   T
  0.3573755375812939  0.7894328411114030  0.5670515964426815   T   T   T
  0.5008955257615203  0.7885026920512958  0.5672337815098977   T   T   T
  0.6433187815977528  0.7855595444522812  0.5682384800668078   T   T   T
  0.7849268943603097  0.7845420118018462  0.5697819148676677   T   T   T
  0.9290310280373142  0.7845145986192981  0.5702536150599143   T   T   T
  0.1589404318682183  0.4325944102564087  0.6166575116800099   T   T   T
  0.2826072177963518  0.4232034690577857  0.6160479531313102   T   T   T
  0.4325995650813481  0.4231531571914650  0.6156882523874737   T   T   T
  0.5553823319717385  0.4317278896602826  0.6162502699683202   T   T   T
  0.0901784723897968  0.4481051602319210  0.5447688865476192   T   T   T
  0.2239592933026668  0.4344875294231380  0.5426095795935403   T   T   T
  0.3576436874136041  0.4412047204562540  0.5468369255692925   T   T   T
  0.4908894353289059  0.4339333643176315  0.5418390581097701   T   T   T
  0.6236704631650944  0.4479898756695473  0.5439365568379224   T   T   T
  0.1409174084985986  0.3539661169367164  0.5642699836238665   T   T   T
  0.2999764602559920  0.3454332841785948  0.5617496258991528   T   T   T
  0.4159777887969765  0.3453489893752197  0.5610376573260606   T   T   T
  0.5735493205885276  0.3534622688996325  0.5634836475405275   T   T   T
  0.1510427977918229  0.4107706122328045  0.4779773954128674   T   T   T
  0.2968273038257952  0.4025273327781805  0.4772398559907284   T   T   T
  0.4175984227025561  0.4019935781655953  0.4765159407440284   T   T   T
  0.5632279770218477  0.4101594435253044  0.4771275325888114   T   T   T
== On-top cluster Pd12-Te12
   1.00000000000000
     24.6586885454000111    0.0000000000000000    0.0000000000000000
      0.0000000000000000   25.2576961516000082    0.0000000000000000
      0.0000000000000000    0.0000000000000000   30.0000000000007034
   W    Te   Pd
    56   124    12
Selective dynamics
Direct
  0.0717555734729931  0.0971695293855103  0.4908791346583410   T   T   T
  0.2131195316435160  0.0975521848350873  0.4915464131595511   T   T   T
  0.3555239271473062  0.0979208280392763  0.4928851482843259   T   T   T
  0.4978425847935840  0.0981547252828769  0.4920381299885279   T   T   T
  0.6457489552826625  0.0991912158347675  0.4929466632432847   T   T   T
  0.7882175735376241  0.0980213407212120  0.4929353523447826   T   T   T
  0.9301748833948300  0.0977997161459174  0.4918528404848944   T   T   T
  0.0717933645542522  0.3476391734093060  0.4916004300250532   T   T   T
  0.2095902299377033  0.3465785084681215  0.4923764821654794   T   T   T
  0.3572922052150979  0.3480275067267845  0.4945503479576055   T   T   T
  0.4994232145977724  0.3469756600655755  0.4827346614229859   T   T   T
  0.6432514584782373  0.3467874422087269  0.4815592538102310   T   T   T
  0.7865749634904394  0.3487564577947432  0.4939997384341916   T   T   T
  0.9335132740373200  0.3470262090456782  0.4920240083715461   T   T   T
  0.0716164238987324  0.5991928924827472  0.4943297323094659   T   T   T
```

0.2093733066250124	0.5992074458504573	0.4945952312024108	T	T	T
0.3630804620633254	0.5991656523565604	0.4932857273207519	T	T	T
0.5015362237391738	0.6005972501484785	0.4910641400288286	T	T	T
0.6419128718928477	0.6002138549248884	0.4910895675529555	T	T	T
0.7827584348926281	0.5987915728470107	0.4937905665361380	T	T	T
0.9329708945538804	0.5995338350197524	0.4944393196168905	T	T	T
0.0717664562318118	0.8485083999293800	0.4930809604184571	T	T	T
0.2146134270979101	0.8481855310763685	0.4932340705600951	T	T	T
0.3573391068790289	0.8489728801221417	0.4933439975420998	T	T	T
0.5003048781177865	0.8510422475285534	0.4936224921489141	T	T	T
0.6436656591390022	0.8508010740007819	0.4936056851505711	T	T	T
0.7860009129590668	0.8488861811375835	0.4935248605477541	T	T	T
0.9288978285452055	0.8482537295512184	0.4933154009679789	T	T	T
0.0003280494964891	0.0085989081083227	0.4985526494580644	T	T	T
0.1428163934043833	0.0085299078161983	0.4983650552291429	T	T	T
0.2858626887094257	0.0090863870738451	0.4995300729612487	T	T	T
0.4268368435856087	0.0099467034163318	0.4999497314776951	T	T	T
0.5729096865726210	0.0112046241001423	0.5005113941149830	T	T	T
0.7157435227142658	0.0101260181773022	0.5004937076736082	T	T	T
0.8574843177889128	0.0091245597455502	0.4997712937306363	T	T	T
0.0028693763537001	0.2579600339780282	0.4973281221737867	T	T	T
0.1405461387306456	0.2577220536471049	0.4972464375357260	T	T	T
0.2849872575930122	0.2577449212454665	0.4985906461396963	T	T	T
0.4262035222228904	0.2590757183355148	0.4974800605028807	T	T	T
0.5702443698157508	0.2588152530764225	0.4897597361246830	T	T	T
0.7189783795688246	0.2599708354646589	0.4965664226333818	T	T	T
0.8585097011133418	0.2583078430503598	0.4987568021149981	T	T	T
0.0024422016838105	0.5095143157215164	0.5003090274347778	T	T	T
0.1409146403747408	0.5093202364041514	0.5005937757824677	T	T	T
0.2778720979683295	0.5100159475765506	0.5009532294044355	T	T	T
0.4355173811459513	0.5107774676192987	0.4964518662206375	T	T	T
0.5715335008262552	0.5111963159539765	0.4927126172078858	T	T	T
0.7091627969367247	0.5105537013704551	0.4966128205735291	T	T	T
0.8647930322564389	0.5102039137988735	0.5002209859072324	T	T	T
0.0007294840239769	0.7599726612196975	0.5006532334060421	T	T	T
0.1429607045934667	0.7599454253715735	0.5006187127305181	T	T	T
0.2861775839404294	0.7593026689219852	0.5001600496708131	T	T	T
0.4300044454397305	0.7618911740901038	0.5004512285378545	T	T	T
0.5719348354987341	0.7631673702205150	0.4996183780388657	T	T	T
0.7136755085525709	0.7613698769046180	0.5004748158651553	T	T	T
0.8574658903065497	0.7595323881788940	0.5004882683387311	T	T	T
0.1430248784447336	0.0727890419995043	0.4256524722580843	T	T	T
0.2854102635653388	0.0729911517486310	0.4269611298941212	T	T	T
0.4270182906953226	0.0731973827172029	0.4271467157772162	T	T	T
0.5723093138007271	0.0750190678888903	0.4284025920764130	T	T	T
0.7162176444819180	0.0736186145911113	0.4277330341335638	T	T	T
0.8580954243064490	0.0729424314158865	0.4271539708569887	T	T	T
0.0000996549371349	0.0727025131595273	0.4259190702515609	T	T	T
0.1420176892293013	0.3232063949788194	0.4252694243062044	T	T	T
0.2846505989548470	0.3256690911020407	0.4291081182346359	T	T	T
0.4219053517544610	0.3224976801754227	0.4242931356762822	T	T	T
0.5707771077612163	0.3220903389033607	0.4167781540405608	T	T	T
0.7216405702676355	0.3233299860474335	0.4234974248137334	T	T	T
0.8590149016248939	0.3256397305493984	0.4286388760809093	T	T	T

0.0016439385887144	0.3233089524350784	0.4253401316110393	T	T	T
0.1416241322564348	0.5744019322489069	0.4280379027150754	T	T	T
0.2852757072608485	0.5757158153045012	0.4311124967311907	T	T	T
0.4303741174687217	0.5764471679081482	0.4254274007927759	T	T	T
0.5717871744827918	0.5800855689977593	0.4242279082220164	T	T	T
0.7143232412706831	0.5769528081066690	0.4261799901147273	T	T	T
0.8583175984443363	0.5761042162010543	0.4302126042737844	T	T	T
0.0015017545167137	0.5750427672614224	0.4280816306456497	T	T	T
0.1432031121489108	0.8238752866401053	0.4279521888826923	T	T	T
0.2861810003184272	0.8237405145355202	0.4281167621641906	T	T	T
0.4292145286885000	0.8262242554901963	0.4282033911071103	T	T	T
0.5718753328830997	0.8280589690518910	0.4280669512547240	T	T	T
0.7144723650346078	0.8256547736614445	0.4281892965183542	T	T	T
0.8572906597308622	0.8237064620069704	0.4282320398307164	T	T	T
0.0001775524423930	0.8240949971822128	0.4280227063946632	T	T	T
0.0716180356581089	0.1951474387372566	0.4446576039611463	T	T	T
0.2134865176782945	0.1964062496367642	0.4464482903154658	T	T	T
0.3551141973488707	0.1961663778571861	0.4467714459089736	T	T	T
0.4963584666504642	0.1954731401717454	0.4438833700793885	T	T	T
0.6467787939997089	0.1963375184089030	0.4442920521203517	T	T	T
0.7890076204662201	0.1962023791920208	0.4463739352167633	T	T	T
0.9298433570864036	0.1964749538903505	0.4468090021854018	T	T	T
0.0718434610533251	0.4473429228196359	0.4474807354238458	T	T	T
0.2118858960656649	0.4471556739872148	0.4484489283535921	T	T	T
0.3554399970695321	0.4501031923725027	0.4521675069571810	T	T	T
0.4993386624675289	0.4499716673140877	0.4396936984715236	T	T	T
0.6439694184066234	0.4502495779332018	0.4398555965658683	T	T	T
0.7879651952017532	0.4497888134333411	0.4512893091493355	T	T	T
0.9318654076610707	0.4477097314806153	0.4476136238703278	T	T	T
0.0717841451074169	0.6977461789855218	0.4493683752792125	T	T	T
0.2135567175485726	0.6972605468265494	0.4491981105772115	T	T	T
0.3587880520578132	0.6980840915164164	0.4496259702448224	T	T	T
0.5004067048456139	0.7009551718666478	0.4476313913742031	T	T	T
0.6433920809871233	0.7005793563639346	0.4475480877738049	T	T	T
0.7853389420254138	0.6977595513768964	0.4499418906039791	T	T	T
0.9296564332760160	0.6976656422761393	0.4493639133059935	T	T	T
0.0716246769922310	0.9464657059537052	0.4474462913879004	T	T	T
0.2148761985018216	0.9463931694714314	0.4479823777727326	T	T	T
0.3569657077635357	0.9471547571215896	0.4479566447603332	T	T	T
0.5001809824029009	0.9496194348978900	0.4490520191504437	T	T	T
0.6439377478055414	0.9492862726341574	0.4488278290371947	T	T	T
0.7861407467364617	0.9473454293107002	0.4485155911437006	T	T	T
0.9284106852357421	0.9465673501208208	0.4480212825459151	T	T	T
0.0016044739585100	0.1597580451669718	0.5425745273548453	T	T	T
0.1417731846479443	0.1595887129823319	0.5424470639210249	T	T	T
0.2835332307659476	0.1596255533882449	0.5433344870592012	T	T	T
0.4279924197886901	0.1608935875582097	0.5410691459793071	T	T	T
0.5711049800032462	0.1645071585023192	0.5383376218268301	T	T	T
0.7165831295149795	0.1625939906915174	0.5404430005957561	T	T	T
0.8596199421327115	0.1601534341855718	0.5435276780084846	T	T	T
0.0030995634590275	0.4102104054292567	0.5447075616040091	T	T	T
0.1405779243301481	0.4099434712711522	0.5451179112064873	T	T	T
0.2790473912189057	0.4110091038748460	0.5427025405089009	T	T	T
0.4375832605794956	0.4123276887281315	0.5380399098911877	T	T	T

0.5718674305946673	0.4102699250432848	0.5264383996971644	T	T	T
0.7054492845494572	0.4116220578290260	0.5381420296782171	T	T	T
0.8651763097542092	0.4118697801437267	0.5420358008855195	T	T	T
0.0015464168275269	0.6619167698018666	0.5467024488756799	T	T	T
0.1417485818558841	0.6619436531264810	0.5469335999577497	T	T	T
0.2860855204223886	0.6590678618544179	0.5435043185814021	T	T	T
0.4297133604159328	0.6631496916091570	0.5449272405592155	T	T	T
0.5722372738859594	0.6649134974674092	0.5403960636793657	T	T	T
0.7139803508976587	0.6623755067825086	0.5450940368759886	T	T	T
0.8577478808565412	0.6597531216904676	0.5444417034075157	T	T	T
0.0002897770963587	0.9107110785591666	0.5441807486667569	T	T	T
0.1432094099062624	0.9105543324076905	0.5442046362723876	T	T	T
0.2856868170181457	0.9106463234560985	0.5445975998744766	T	T	T
0.4280940065673426	0.9117685921182737	0.5449510354353517	T	T	T
0.5721374511841639	0.9128412678008614	0.5447805631428149	T	T	T
0.7153811983214715	0.9116345894271891	0.5452753608429590	T	T	T
0.8578488221801737	0.9105691004279346	0.5446821334265658	T	T	T
0.0717276301717790	0.0332818927366830	0.5635896047831566	T	T	T
0.2133715248314193	0.0334109763152510	0.5640301663224865	T	T	T
0.3559602752747461	0.0337293880625913	0.5655179667976360	T	T	T
0.4995111201430490	0.0352065833719178	0.5647048323207633	T	T	T
0.6441719002089002	0.0358185345703440	0.5656839684875372	T	T	T
0.7873225158979279	0.0342003127878442	0.5656481577502717	T	T	T
0.9297962444391978	0.0337091619515183	0.5642691887681477	T	T	T
0.0718573402840460	0.2823989710703221	0.5637515615658260	T	T	T
0.2117834487353064	0.2810140893629745	0.5632373370949859	T	T	T
0.3537379355168086	0.2798359048344372	0.5658376202599767	T	T	T
0.5050530659850774	0.2882246107355594	0.5572270479925672	T	T	T
0.6398072948098178	0.2880760197589239	0.5550713453614962	T	T	T
0.7905112611709180	0.2822533706090773	0.5661315473663542	T	T	T
0.9318962610550952	0.2817425222805799	0.5632121685114267	T	T	T
0.0716585232045675	0.5343951423835241	0.5666072898056624	T	T	T
0.2106337203970046	0.5361580444092329	0.5674947185783970	T	T	T
0.3583548375001914	0.5312593948505054	0.5603082992403418	T	T	T
0.5039928217602441	0.5306105949814650	0.5625347592532443	T	T	T
0.6395717118407640	0.5299305471103221	0.5621941026274810	T	T	T
0.7857222083190475	0.5299505947774990	0.5611650258487512	T	T	T
0.9324716018511235	0.5356281470739115	0.5669647668096701	T	T	T
0.0718236577906267	0.7850462293661420	0.5659157621101075	T	T	T
0.2149708797153234	0.7833970034305634	0.5655444390049051	T	T	T
0.3572059212437460	0.7843937838305326	0.5655151947613304	T	T	T
0.5008318724427371	0.7863979635438298	0.5656647273286044	T	T	T
0.6431956917472119	0.7862320326855398	0.5657343998539973	T	T	T
0.7862256938259242	0.7843414492826678	0.5657037170638173	T	T	T
0.9288621122084870	0.7836998176357123	0.5657274577897478	T	T	T
0.4812723145774166	0.2037778963152722	0.6827162729418307	T	T	T
0.6406567371919557	0.1941180045277791	0.6631568611560457	T	T	T
0.4421459156291574	0.3072080147972392	0.6693067071031709	T	T	T
0.5707046133360926	0.3882176722436567	0.6226281398519312	T	T	T
0.6873206234918404	0.2965250357908725	0.6713846522504767	T	T	T
0.3651619283916127	0.4222590406581137	0.6680713884706271	T	T	T
0.4717019922269831	0.4901551486533329	0.6927884079577816	T	T	T
0.6614217197567954	0.4798787775052204	0.6877145662210914	T	T	T
0.7701162092567553	0.4093709446526927	0.6724352509569848	T	T	T

```
  0.4417283384730408  0.6024734706127456  0.6686897257707861   T   T   T
  0.5709902415964014  0.5913757050476475  0.6672547267181967   T   T   T
  0.6999980805549433  0.5912260483509739  0.6684996495432100   T   T   T
  0.4362218881643625  0.2295220025286967  0.6076304937091495   T   T   T
  0.5501828005996122  0.1941402187651023  0.6212793025257725   T   T   T
  0.7116349381398199  0.2294276586005836  0.6066489206299442   T   T   T
  0.3616232672538796  0.3812976320968381  0.5897046083036180   T   T   T
  0.4634693928630298  0.4115302214664145  0.6318127885270183   T   T   T
  0.6784101567459925  0.3990838043207298  0.6285419520446621   T   T   T
  0.7837115379985755  0.3845409255275405  0.5899497115032153   T   T   T
  0.3956930103995546  0.5158908205464878  0.6407778844796781   T   T   T
  0.5652593890614290  0.4886409433441894  0.6494252947663062   T   T   T
  0.7410099416568350  0.5026695420556210  0.6381492887200375   T   T   T
  0.5068385617186388  0.6276026595488182  0.6035566174248951   T   T   T
  0.6368286744220324  0.6258719055122055  0.6042194884578778   T   T   T
== On-top cluster Pd08-Te07
   1.00000000000000
    24.6586885454000111    0.0000000000000000    0.0000000000000000
     0.0000000000000000   25.2576961516000082    0.0000000000000000
     0.0000000000000000    0.0000000000000000   30.0000000000007034
   W    Te   Pd
    56   119     8
Selective dynamics
Direct
  0.0706833256229011  0.0970635312334087  0.4904884696736657   T   T   T
  0.2130182173079814  0.0976615421917147  0.4911259817758386   T   T   T
  0.3562227325180620  0.0971936250474243  0.4916760099726132   T   T   T
  0.5001493850067110  0.0980953095435403  0.4924399451603994   T   T   T
  0.6439515412273511  0.0972092939231760  0.4917338549072147   T   T   T
  0.7871375619906111  0.0977084126989784  0.4911331093045809   T   T   T
  0.9294598688860236  0.0970857847045223  0.4904597657097711   T   T   T
  0.0690199898939222  0.3476266043519149  0.4925933669292423   T   T   T
  0.2084159874794458  0.3451672936624899  0.4932111369220415   T   T   T
  0.3554707722606113  0.3459570481753089  0.4987907723645036   T   T   T
  0.5000259222694335  0.3424307874357044  0.4920104862659374   T   T   T
  0.6446255359081013  0.3459463507067791  0.4987499501944437   T   T   T
  0.7917892903269070  0.3451445670398520  0.4930077507376529   T   T   T
  0.9310831140361274  0.3476244176099869  0.4925143121300655   T   T   T
  0.0702382716710063  0.5983402048703363  0.4958270931589777   T   T   T
  0.2106315930191247  0.5981702878183273  0.4960278397767307   T   T   T
  0.3518578959781674  0.5956066579211953  0.4942659660595435   T   T   T
  0.5000116797100950  0.5981254230144081  0.4943100836504040   T   T   T
  0.6486123675128815  0.5956104483345858  0.4942652632597707   T   T   T
  0.7894484536553268  0.5981671010492264  0.4959186861574863   T   T   T
  0.9298312219373726  0.5983500070067759  0.4958089110662516   T   T   T
  0.0707401798949102  0.8481115122710549  0.4929705347151845   T   T   T
  0.2131177185302326  0.8483042197602360  0.4925379189367042   T   T   T
  0.3561036726746187  0.8494281890747353  0.4912715125031401   T   T   T
  0.5000701232594448  0.8498827966722463  0.4904440563498318   T   T   T
  0.6440420240974272  0.8494464629629033  0.4912920665088703   T   T   T
  0.7869434793129422  0.8483251227943477  0.4925227865464720   T   T   T
  0.9293290947817260  0.8481295476748153  0.4929603961266798   T   T   T
  0.0000439127944395  0.0082718743813169  0.4976680405129587   T   T   T
  0.1416875609537793  0.0086914619380040  0.4977417672185874   T   T   T
```

0.2846290520746829	0.0090874066187914	0.4981860889589403	T	T	T
0.4281214866775474	0.0094277977093194	0.4982602745553797	T	T	T
0.5721383019619062	0.0094378822362667	0.4983157856115877	T	T	T
0.7155043887423623	0.0091198257732831	0.4982210553557814	T	T	T
0.8583953482733135	0.0087333132995669	0.4977677413304875	T	T	T
0.0001315623968508	0.2577823825216747	0.4972879704628728	T	T	T
0.1383292538666824	0.2573431181146676	0.4978661269154750	T	T	T
0.2873352128207620	0.2573992290536253	0.5003656284611949	T	T	T
0.4227859875911167	0.2573258968870664	0.5015289306163836	T	T	T
0.5773571207887912	0.2573081749000001	0.5015695889108294	T	T	T
0.7127010023231716	0.2574204112455989	0.5002661165736886	T	T	T
0.8619023436368503	0.2573330200242704	0.4977196497223216	T	T	T
0.0000151001411523	0.5085931861294070	0.5012704289342959	T	T	T
0.1401077842533296	0.5086169766225320	0.5023188681951495	T	T	T
0.2760409865041554	0.5071411322286584	0.5022505026939027	T	T	T
0.4365902276388336	0.5076312926164439	0.5005956833908046	T	T	T
0.5636151787774150	0.5076425718058022	0.5005953416580644	T	T	T
0.7239679459975930	0.5071356567288708	0.5020317299817592	T	T	T
0.8599226656267890	0.5086100589438662	0.5022342758243287	T	T	T
0.0000236116835031	0.7591320829829972	0.5005436362399472	T	T	T
0.1415085643757947	0.7593364733706491	0.5005281974595328	T	T	T
0.2842315879156279	0.7600897709305456	0.5001974408939497	T	T	T
0.4271233960201269	0.7626667469612128	0.4974633938070979	T	T	T
0.5729838120107810	0.7626542704481317	0.4974656421778622	T	T	T
0.7158796611554918	0.7601071298754981	0.5001602474443830	T	T	T
0.8586209028227226	0.7593310998636914	0.5005177019598516	T	T	T
0.1425039624239652	0.0733915043532313	0.4253671503682447	T	T	T
0.2849557023420690	0.0739407722521422	0.4260807063903113	T	T	T
0.4287988443321334	0.0750808931538216	0.4267714419666054	T	T	T
0.5714912545410578	0.0750370223846646	0.4267448065814389	T	T	T
0.7152297286867216	0.0739634760572822	0.4261336507888415	T	T	T
0.8576417723265176	0.0733952782594743	0.4253505680889034	T	T	T
0.0000949360471292	0.0723520861307013	0.4249681301033944	T	T	T
0.1400698482842332	0.3227894797159274	0.4263900827156302	T	T	T
0.2853055432459101	0.3251668864212773	0.4309336357245477	T	T	T
0.4243005838994421	0.3228868027264910	0.4301736718811850	T	T	T
0.5757747625356872	0.3227849269526767	0.4301875622141502	T	T	T
0.7147110709443176	0.3252167536251143	0.4308425400112142	T	T	T
0.8601663300353024	0.3227295398266428	0.4261963287553471	T	T	T
0.0001174159303395	0.3240109055164288	0.4256329614044524	T	T	T
0.1407557114392975	0.5733487069710959	0.4302441971269328	T	T	T
0.2810613097958204	0.5711917184060800	0.4300512875157141	T	T	T
0.4269248961008524	0.5733688204045511	0.4300353006493123	T	T	T
0.5733459312221678	0.5735069441770221	0.4301525533780944	T	T	T
0.7191141193980038	0.5713224859961763	0.4298450073899076	T	T	T
0.8593966741808846	0.5733364877237124	0.4301782125907576	T	T	T
0.0000493299408965	0.5743071322728999	0.4298025821558972	T	T	T
0.1418009319456083	0.8230838540841917	0.4277068640381457	T	T	T
0.2841782137533239	0.8233233461807105	0.4270604786672276	T	T	T
0.4278095959879609	0.8262503203680663	0.4253305461494194	T	T	T
0.5723213095041035	0.8262993456990212	0.4253490389123817	T	T	T
0.7158897282897067	0.8233319212191356	0.4270404337452164	T	T	T
0.8582508793668004	0.8230801897505883	0.4277111560935697	T	T	T
0.0000475856408037	0.8227970736665566	0.4276407081144256	T	T	T

0.0702919044145842	0.1954711833810963	0.4445994001749771	T	T	T
0.2138270185209216	0.1975119772172846	0.4477340392695397	T	T	T
0.3561500625526617	0.1961859289222089	0.4467597573240978	T	T	T
0.5000948283412374	0.1983893824116147	0.4505671821328600	T	T	T
0.6439418840389173	0.1961641297872887	0.4467414394177443	T	T	T
0.7862852478526725	0.1975799597738587	0.4476911244791435	T	T	T
0.9300667790566091	0.1955308068889082	0.4445439833425115	T	T	T
0.0706494453510903	0.4467394913616743	0.4489111009657307	T	T	T
0.2100340903760737	0.4454654710489910	0.4493276758973356	T	T	T
0.3555508929991221	0.4475299799962759	0.4561465267536634	T	T	T
0.5000544430893884	0.4434560670028460	0.4455951937506812	T	T	T
0.6445399523142380	0.4474263696973988	0.4559304755998019	T	T	T
0.7902159007627787	0.4454506293122517	0.4490861717498492	T	T	T
0.9294558493007841	0.4467397037796484	0.4488578355098405	T	T	T
0.0707939173508132	0.6959828682473872	0.4496959038103217	T	T	T
0.2125396185402693	0.6960629332757015	0.4501813656706903	T	T	T
0.3537994358338658	0.6967693331677957	0.4487041784939351	T	T	T
0.5000304042312749	0.6989897467810833	0.4479901714871178	T	T	T
0.6463507452239952	0.6967408193336713	0.4487167831107701	T	T	T
0.7875884562036213	0.6961021641508202	0.4501486789427067	T	T	T
0.9292988964817831	0.6959887223763919	0.4497048747360649	T	T	T
0.0711951407382906	0.9457302754396525	0.4466257103266214	T	T	T
0.2134398949619476	0.9466133775971355	0.4468629533179519	T	T	T
0.3563746773935929	0.9479269388816258	0.4465297185391046	T	T	T
0.5002281688089152	0.9487266535961034	0.4463323786804667	T	T	T
0.6437949422399496	0.9479634128199818	0.4465555599499249	T	T	T
0.7866398507313418	0.9466315639945878	0.4468750221222818	T	T	T
0.9289311950859845	0.9457745755205297	0.4466177009838482	T	T	T
0.0000844846576129	0.1594076478392324	0.5421200935548909	T	T	T
0.1410772894819964	0.1592588401948371	0.5422196314510921	T	T	T
0.2846803925286780	0.1582152623084498	0.5433944889036765	T	T	T
0.4273768114017277	0.1578172246094985	0.5444431416098037	T	T	T
0.5729055923449151	0.1577867125064017	0.5445207279331884	T	T	T
0.7155228716061001	0.1583103284081354	0.5434144603685621	T	T	T
0.8591086901147683	0.1593333943549343	0.5421279087544203	T	T	T
0.0000126988809578	0.4095945011759718	0.5456481350607822	T	T	T
0.1396640696162264	0.4081208093072748	0.5458894381894250	T	T	T
0.2767102428846354	0.4085681541038074	0.5464807742106694	T	T	T
0.4341746544290087	0.4097609569537886	0.5428727303826323	T	T	T
0.5659800859229055	0.4097414710941884	0.5428414313326425	T	T	T
0.7235131475530534	0.4085665453466707	0.5462577137274424	T	T	T
0.8604056167030431	0.4081041994720771	0.5457835979524905	T	T	T
0.0000254857780556	0.6616829722710064	0.5471335570190327	T	T	T
0.1405884269766716	0.6617037929375684	0.5471176129823317	T	T	T
0.2821922973526673	0.6611662431721521	0.5468290294286946	T	T	T
0.4251908355654698	0.6646172357002841	0.5358578652308666	T	T	T
0.5749060768945345	0.6645737220187902	0.5358499387932538	T	T	T
0.7180042759325578	0.6612265997327438	0.5467870161429099	T	T	T
0.8594406010337675	0.6616884951627461	0.5471053481582725	T	T	T
0.0000236251281666	0.9109760616135444	0.5439766983347928	T	T	T
0.1422834703185738	0.9109737877194176	0.5435359964635007	T	T	T
0.2851192563401114	0.9108820097568935	0.5430174121830776	T	T	T
0.4284455793318430	0.9109956560935821	0.5422707387164762	T	T	T
0.5716486547328606	0.9109688805394728	0.5423092645618255	T	T	T

```
  0.7149776932300287  0.9109148592419662  0.5430600660266223   T   T   T
  0.8577810876675365  0.9109733581897509  0.5435403231530405   T   T   T
  0.0708775995360322  0.0329989427663992  0.5631651810155823   T   T   T
  0.2129415887351622  0.0330876916598389  0.5632426201151735   T   T   T
  0.3563579055376906  0.0325235426295095  0.5636564133594717   T   T   T
  0.5000873283679726  0.0323510025043499  0.5637081833548704   T   T   T
  0.6438530080727385  0.0324986771987606  0.5637378939670791   T   T   T
  0.7871394681064545  0.0331253195272031  0.5632938766235599   T   T   T
  0.9292226242568523  0.0330600021808892  0.5631643782242903   T   T   T
  0.0695005226477981  0.2816501576873863  0.5641294630393141   T   T   T
  0.2110418622027428  0.2793907245913725  0.5636229883616117   T   T   T
  0.3541638556572713  0.2752521006922188  0.5697604860462911   T   T   T
  0.5000530142283380  0.2790061435424451  0.5650751458452939   T   T   T
  0.6460544983070746  0.2752174293114430  0.5697620003546752   T   T   T
  0.7891066777119450  0.2794312685388743  0.5634469833086359   T   T   T
  0.9305941439047645  0.2816888066138339  0.5640539939834811   T   T   T
  0.0696502686634816  0.5323302698481335  0.5677188098521044   T   T   T
  0.2099182198948807  0.5345191220290889  0.5690031256174359   T   T   T
  0.3570327366950923  0.5254943834375687  0.5619192194216143   T   T   T
  0.5000744273860497  0.5342869421781202  0.5682798491361346   T   T   T
  0.6431213532795136  0.5254310568684643  0.5619076236948057   T   T   T
  0.7900357371813078  0.5344725731496004  0.5688941977608380   T   T   T
  0.9303219050602085  0.5323033129043360  0.5676753522606280   T   T   T
  0.0708261154356815  0.7845141842064282  0.5658619713745542   T   T   T
  0.2130609228233141  0.7849230182453866  0.5654626561411145   T   T   T
  0.3565981182877676  0.7861685111599671  0.5639809925152507   T   T   T
  0.5000491557682943  0.7850545648311692  0.5622935340528632   T   T   T
  0.6435073875294759  0.7861493581963863  0.5639815471007241   T   T   T
  0.7870469963084016  0.7849628763247387  0.5654312806554992   T   T   T
  0.9292273633386473  0.7845585691057508  0.5658725924274477   T   T   T
  0.4209504355345694  0.3583138602249786  0.6661396926246387   T   T   T
  0.5794112759717248  0.3580130128335579  0.6659711795970892   T   T   T
  0.3523324685061981  0.4445843254017012  0.6635695466239414   T   T   T
  0.5001710271951115  0.4784511645578032  0.6664660821174165   T   T   T
  0.6478496051652510  0.4444735285323871  0.6634876443837725   T   T   T
  0.4343875120746071  0.6260723762466964  0.6614856707280917   T   T   T
  0.5659141426569293  0.6262535418985579  0.6612936733729952   T   T   T
  0.3555199076090950  0.3759938981315415  0.5974305684667442   T   T   T
  0.5001192810513739  0.3879187070090324  0.6114928046131139   T   T   T
  0.6448155704146179  0.3759923312802377  0.5973178916350532   T   T   T
  0.4092604810606483  0.5282018047079232  0.6461718179681581   T   T   T
  0.5910106909835923  0.5282665342659822  0.6461879144814549   T   T   T
  0.5000180979884230  0.6356347458752624  0.5926059172652601   T   T   T
  0.3659710230413208  0.6202467658167544  0.5960359224734948   T   T   T
  0.6344229931871603  0.6202496883497259  0.5958958877454055   T   T   T
== On-top cluster Pd06-Te07
   1.00000000000000
    24.6586885454000111    0.0000000000000000    0.0000000000000000
     0.0000000000000000   25.2576961516000082    0.0000000000000000
     0.0000000000000000    0.0000000000000000   30.0000000000007034
   W    Te   Pd
    56   119     6
Selective dynamics
Direct
```

0.0709638499750877	0.0978522474749827	0.4902342002808406	T	T	T
0.2131483711346107	0.0980286797973475	0.4910763715772522	T	T	T
0.3564710104234098	0.0979733517279249	0.4921158371397460	T	T	T
0.5000220107450870	0.0983650952259579	0.4925026989221093	T	T	T
0.6435627225867357	0.0979713700925431	0.4921368046784280	T	T	T
0.7869563472628315	0.0979786045104927	0.4910772680795131	T	T	T
0.9291865492247927	0.0978677181216685	0.4902886497167572	T	T	T
0.0697706105648855	0.3483463847085735	0.4928882780124234	T	T	T
0.2106001396969061	0.3469417635369167	0.4940950058105152	T	T	T
0.3568683335183591	0.3467150641276172	0.4982875185615353	T	T	T
0.5001055490267551	0.3455262965799326	0.4934418750409299	T	T	T
0.6432874954406437	0.3466757126158145	0.4981611696063538	T	T	T
0.7894627034702303	0.3469375117782472	0.4940893329706654	T	T	T
0.9302705465397387	0.3483145184402393	0.4928936583795727	T	T	T
0.0707594086958533	0.5988185046466908	0.4946086199229152	T	T	T
0.2127949006836327	0.5985984660222288	0.4947496904557804	T	T	T
0.3563717487644377	0.5992944206706731	0.4947151022333939	T	T	T
0.5000944403162995	0.5995508429600669	0.4901995283220938	T	T	T
0.6437322872674650	0.5993613603027519	0.4946839348921456	T	T	T
0.7873312942851589	0.5986304204040407	0.4947758400447026	T	T	T
0.9293164811763575	0.5988071716118407	0.4946386190249164	T	T	T
0.0709611506153146	0.8484371794516588	0.4917344799919805	T	T	T
0.2134637632963509	0.8485278682058716	0.4915704869709043	T	T	T
0.3569060056126549	0.8491228548878534	0.4907749605912982	T	T	T
0.5000311060720949	0.8502660030849298	0.4907414394994460	T	T	T
0.6431651054876523	0.8491721267264857	0.4907914007695862	T	T	T
0.7866735892290916	0.8485517983657498	0.4916060340859055	T	T	T
0.9293465297745536	0.8485071946960190	0.4918822760950513	T	T	T
0.0001395610454616	0.0090025246687729	0.4969090079170165	T	T	T
0.1419862659749073	0.0090463279881026	0.4972970270036827	T	T	T
0.2848569376183797	0.0093757120578466	0.4980644386693761	T	T	T
0.4278953779845991	0.0097458525119550	0.4984375552796400	T	T	T
0.5721287089677405	0.0097519387899300	0.4984459929240019	T	T	T
0.7152409613159061	0.0094043918315287	0.4980918241630254	T	T	T
0.8581867236156490	0.0090603738403190	0.4973497904896096	T	T	T
0.0000266987816798	0.2585606255161294	0.4977189840453692	T	T	T
0.1405031824210644	0.2584957917442465	0.4985070131009065	T	T	T
0.2865861646681812	0.2582717335999620	0.5001845052945638	T	T	T
0.4249570470864097	0.2585162748625501	0.5014868813893516	T	T	T
0.5751265454878353	0.2584948014293658	0.5014133913902883	T	T	T
0.7138012323164398	0.2581785708878578	0.5001306909154090	T	T	T
0.8594116628371247	0.2584708807621901	0.4985378470939192	T	T	T
0.0000239584603872	0.5094056959749803	0.5010587315672170	T	T	T
0.1413017832716012	0.5095260488195071	0.5017417676158662	T	T	T
0.2853512553373183	0.5098066941075254	0.5031278253245102	T	T	T
0.4297842489433193	0.5107972530900545	0.5007151594013134	T	T	T
0.5704213540997904	0.5108120702388115	0.5006627934299515	T	T	T
0.7147472107420679	0.5098228144256768	0.5030627602404470	T	T	T
0.8587647958829058	0.5094926622890628	0.5017341077828951	T	T	T
0.0000899909517736	0.7595591484164255	0.4993966073786372	T	T	T
0.1419549978639790	0.7596262436114962	0.4993641005491861	T	T	T
0.2845829888493462	0.7600262840180378	0.4989692457141305	T	T	T
0.4296692858937146	0.7618398072354252	0.4971787939351651	T	T	T
0.5704680174796957	0.7619041856855535	0.4972349407077388	T	T	T

0.7155234448246680	0.7600946055084665	0.4990389885169836	T	T	T
0.8583303802395041	0.7596804186318141	0.4995256733734785	T	T	T
0.1427906969568315	0.0737922427131216	0.4251459609625748	T	T	T
0.2854575765545673	0.0746357409858714	0.4262960178725600	T	T	T
0.4285966056786625	0.0753377137522939	0.4269666047097332	T	T	T
0.5714428416774477	0.0753430839066738	0.4269701737208643	T	T	T
0.7145731165392804	0.0746357142071762	0.4263095621838376	T	T	T
0.8573566691539073	0.0737840311817615	0.4251909253643064	T	T	T
0.0000302643318789	0.0738920438692373	0.4246669512086768	T	T	T
0.1417728988391665	0.3247134563981902	0.4272612895899068	T	T	T
0.2862727097038683	0.3257643984469595	0.4307949729871761	T	T	T
0.4264023909109114	0.3235526603576449	0.4301550674439006	T	T	T
0.5736741217780831	0.3235717044871474	0.4300834425282419	T	T	T
0.7139152572189901	0.3256110107782663	0.4307469137730629	T	T	T
0.8583036459736545	0.3246950446399342	0.4272721714551379	T	T	T
0.0000187700494392	0.3252503694654431	0.4264310989228333	T	T	T
0.1419152738453790	0.5737674978694738	0.4294613655496147	T	T	T
0.2846115073332732	0.5728577070473870	0.4302368863492065	T	T	T
0.4266839957210146	0.5737532334338517	0.4278150068717092	T	T	T
0.5734023596991905	0.5737826501003790	0.4277807959615285	T	T	T
0.7154955903594801	0.5728940667868164	0.4302355170480728	T	T	T
0.8581897775626661	0.5737443831617106	0.4294812087362140	T	T	T
0.0000322287296745	0.5743706566930998	0.4290954112356751	T	T	T
0.1421428810183118	0.8233622903201174	0.4265697199752479	T	T	T
0.2847937792998373	0.8236138463802906	0.4263111700485634	T	T	T
0.4284208814045174	0.8264029679806922	0.4252287798235442	T	T	T
0.5716598966066105	0.8264602524111730	0.4252506214546226	T	T	T
0.7153119452894616	0.8236047613799059	0.4263364481705759	T	T	T
0.8581383940039281	0.8234020575125439	0.4267442543233576	T	T	T
0.0000341841235224	0.8233361982995581	0.4264923963700423	T	T	T
0.0711257111865791	0.1969692897295866	0.4455480306222035	T	T	T
0.2143456792723472	0.1978814102721322	0.4475649473264722	T	T	T
0.3569040756490746	0.1969947917275825	0.4475235886170514	T	T	T
0.5000097024669526	0.1983334172754957	0.4500629066412716	T	T	T
0.6431746301035335	0.1969597533756817	0.4475857144958486	T	T	T
0.7858938975829689	0.1977126399912032	0.4474412969359210	T	T	T
0.9289641468773724	0.1969766699662522	0.4456266138209765	T	T	T
0.0710728502937547	0.4476751468038658	0.4491970286497363	T	T	T
0.2134633234958881	0.4476693362815845	0.4512894868040234	T	T	T
0.3566485735213440	0.4462606117055412	0.4532939113969999	T	T	T
0.5000825376224651	0.4464340059385459	0.4484565251100764	T	T	T
0.6434585780846722	0.4462951315010363	0.4531742027677308	T	T	T
0.7866367899289997	0.4476582018601960	0.4512449256452711	T	T	T
0.9290492551000380	0.4476286905109748	0.4491694742136739	T	T	T
0.0710336495315417	0.6966234209827185	0.4485931582497699	T	T	T
0.2132439771611218	0.6966727883695797	0.4486706792649060	T	T	T
0.3562824559263945	0.6977206362142522	0.4480564548250801	T	T	T
0.5002076874172614	0.6992032993244952	0.4445951098313881	T	T	T
0.6438703570791090	0.6978566372490336	0.4480698530882472	T	T	T
0.7869508751261840	0.6967664456993538	0.4488552354544714	T	T	T
0.9290726722738241	0.6966226727795546	0.4486461586638228	T	T	T
0.0713462629872762	0.9465067544676142	0.4457791812176423	T	T	T
0.2139322361236901	0.9470593037479154	0.4463247116515345	T	T	T
0.3566752275294470	0.9480787267003349	0.4465175366232769	T	T	T

0.5000371405622906	0.9490668359516982	0.4466024181669913	T	T	T
0.6434493240461783	0.9481587878212540	0.4465339740441304	T	T	T
0.7862117308694222	0.9470655997673585	0.4463689601406011	T	T	T
0.9288183640419679	0.9465100512934255	0.4458611973440496	T	T	T
0.0000946523941724	0.1597917326759443	0.5421599157781720	T	T	T
0.1413300037628044	0.1597242676656448	0.5424052735325348	T	T	T
0.2841055822182315	0.1586610790144966	0.5435592757286475	T	T	T
0.4278864311334718	0.1583765065342170	0.5447256318643998	T	T	T
0.5721525628573867	0.1583910403147741	0.5447347077059335	T	T	T
0.7160267459904828	0.1585762922257349	0.5435708451314321	T	T	T
0.8587129871208252	0.1596995456853799	0.5424246749920035	T	T	T
0.0000088638513789	0.4101105702715142	0.5455643001933008	T	T	T
0.1404313423638967	0.4095585784621714	0.5457709184376550	T	T	T
0.2806483425381285	0.4102450433426316	0.5453965983106303	T	T	T
0.4321560969869584	0.4120142114253726	0.5430684678572965	T	T	T
0.5681396868736940	0.4120381405102280	0.5430272215102322	T	T	T
0.7194152526438515	0.4102225612303168	0.5451976854612698	T	T	T
0.8596364544330523	0.4095475647345615	0.5457756657073162	T	T	T
0.0000792659816027	0.6617427716747303	0.5455914543517369	T	T	T
0.1419935473686386	0.6618672904976796	0.5454334386906292	T	T	T
0.2845363896575899	0.6619575963220324	0.5449084691143162	T	T	T
0.4286545564133055	0.6645627227483449	0.5401300989672170	T	T	T
0.5714776782817849	0.6645875268069230	0.5401487827973948	T	T	T
0.7155609318180991	0.6620012094768956	0.5449262154275624	T	T	T
0.8582008454302422	0.6617775989030278	0.5455931718249045	T	T	T
0.0002953839439055	0.9113008362625410	0.5426251817697882	T	T	T
0.1423588793567428	0.9108777391972063	0.5426180473594651	T	T	T
0.2851636013996709	0.9107076066412896	0.5424243874881328	T	T	T
0.4281861746546176	0.9110456105230806	0.5424317562508407	T	T	T
0.5718722095554172	0.9110697995294201	0.5424431139024437	T	T	T
0.7149232986543873	0.9107380140393655	0.5424080866349384	T	T	T
0.8578219905261609	0.9109041061511557	0.5426979776838202	T	T	T
0.0709323498999986	0.0333368511565189	0.5625118261027994	T	T	T
0.2129973913256334	0.0331110110649339	0.5630424026686623	T	T	T
0.3561674719188915	0.0327620679923643	0.5637956997567534	T	T	T
0.5000007997483471	0.0324963524774785	0.5637423542514853	T	T	T
0.6438623271444260	0.0327515557274111	0.5637944520195151	T	T	T
0.7871434942958400	0.0330817017790811	0.5630830689735159	T	T	T
0.9293004909501785	0.0333229241167452	0.5625449780243359	T	T	T
0.0699872400323105	0.2821095460440348	0.5641564652894901	T	T	T
0.2121677155899709	0.2807786198234425	0.5644149079874260	T	T	T
0.3545459346508101	0.2741407778413047	0.5689810707993144	T	T	T
0.5000977121888148	0.2798675455543719	0.5663179157043253	T	T	T
0.6456804871650362	0.2740204675610015	0.5687787669074835	T	T	T
0.7879380315798733	0.2808641085209382	0.5644978141321775	T	T	T
0.9300381319482883	0.2820870151008620	0.5641515210441874	T	T	T
0.0705021270795119	0.5339827808250849	0.5669369372216919	T	T	T
0.2126325029930044	0.5350722048874395	0.5673036202595354	T	T	T
0.3586075885170042	0.5353983439516568	0.5670449087382372	T	T	T
0.5001624058874049	0.5375595939947064	0.5648047150668279	T	T	T
0.6415839492206422	0.5354581491635950	0.5669754789621565	T	T	T
0.7874191760003356	0.5350637703982799	0.5672753766653988	T	T	T
0.9295332671876344	0.5339370033303873	0.5669404178350287	T	T	T
0.0711218823102300	0.7848783534355931	0.5646174815854879	T	T	T

```
  0.2136638786626341  0.7848746607634218  0.5643478113088675   T   T   T
  0.3573712529077593  0.7853239154719927  0.5629160808767857   T   T   T
  0.5000151409727372  0.7856857029832659  0.5631060226685743   T   T   T
  0.6427479564782433  0.7854425052696479  0.5629841677782369   T   T   T
  0.7865269068499506  0.7849868276781160  0.5643813519034506   T   T   T
  0.9291624827539374  0.7848567757857878  0.5647516932749556   T   T   T
  0.4218849692772648  0.3537081107687056  0.6653691840309228   T   T   T
  0.5784071388891174  0.3537459800495521  0.6654091496312247   T   T   T
  0.3497940798393714  0.4375334345108851  0.6660931528843839   T   T   T
  0.5004038588332216  0.4673153696173367  0.6668029622132251   T   T   T
  0.6508540522622014  0.4372901636687909  0.6659347588223775   T   T   T
  0.4445906580360704  0.6098250833869737  0.6709562637441415   T   T   T
  0.5555988544638805  0.6096848698058249  0.6708415922452369   T   T   T
  0.3550037756406339  0.3730070355280432  0.5983356649598224   T   T   T
  0.5001670104755960  0.3838803212228381  0.6089802831714783   T   T   T
  0.6452423364654252  0.3728150711500976  0.5982305671647055   T   T   T
  0.4098126325848203  0.5179675604079651  0.6475719354108996   T   T   T
  0.5908361105842312  0.5178726120182346  0.6475173808154204   T   T   T
  0.5000177894115044  0.6404910798131461  0.6017310410761684   T   T   T
== On-top cluster Pd05-Te05
     1.0000000000000000
    31.7040281100000030    0.0000000000000000    0.0000000000000000
     0.0000000000000000   31.5721201999999970    0.0000000000000000
     0.0000000000000000    0.0000000000000000   22.0000000000000000
   W    Te   Pd
    90   185     5
Selective dynamics
Direct
  0.0545900677739099  0.0780320182857851  0.4918140051114345   T   T   T
  0.0506608087182911  0.2773000502803855  0.4958852380895198   T   T   T
  0.0520313060679485  0.4801331029307083  0.4976484360468286   T   T   T
  0.0547943847736519  0.6793660370637113  0.4935357319813150   T   T   T
  0.0550523706696805  0.8787134819392270  0.4926831979053872   T   T   T
  0.1658724453595722  0.0776961454346189  0.4926238472795270   T   T   T
  0.1640965968671067  0.2773899345548319  0.5059250474524902   T   T   T
  0.1655366682094036  0.4792654209304703  0.4984730823316991   T   T   T
  0.1661722398142442  0.6793859628077545  0.4924430717655422   T   T   T
  0.1664337119730668  0.8787961812369082  0.4922859290726089   T   T   T
  0.2779512426757806  0.0787572313976099  0.4938778089129089   T   T   T
  0.2778496420467992  0.2741702348505867  0.4992720761834455   T   T   T
  0.2777173610918027  0.4812824010259762  0.5030394979712828   T   T   T
  0.2775870298887712  0.6794186058150827  0.4919973384835117   T   T   T
  0.2777752095430756  0.8789308725345573  0.4920819718300362   T   T   T
  0.3898786506610882  0.0777288038269385  0.4922485597110402   T   T   T
  0.3911978013355006  0.2774840857893937  0.5056358178566810   T   T   T
  0.3901002658906967  0.4792594553533615  0.4980579247834927   T   T   T
  0.3892981747488193  0.6793205083655970  0.4918093856833027   T   T   T
  0.3891556706687391  0.8787307881944573  0.4919636234016949   T   T   T
  0.5009145299470911  0.0781954907837235  0.4916404916122404   T   T   T
  0.5047364676827644  0.2773364299675473  0.4955075995728130   T   T   T
  0.5032827404307639  0.4800294468424899  0.4968304600914086   T   T   T
  0.5007387264645302  0.6793619137491209  0.4926778400303218   T   T   T
  0.5005816639498668  0.8787576658060472  0.4922022857859767   T   T   T
  0.6118188070071434  0.0781053608338765  0.4916446984151637   T   T   T
```

0.6133633418458558	0.2784071202518238	0.4928892524730146	T	T	T
0.6123392805458345	0.4793846878298020	0.4950674881662161	T	T	T
0.6115304509308489	0.6793958125527915	0.4937988850836880	T	T	T
0.6115106956712558	0.8788085446633280	0.4928866553873047	T	T	T
0.7224537316484118	0.0784117397956431	0.4922494126135644	T	T	T
0.7226501389286097	0.2786299275338646	0.4925643238606786	T	T	T
0.7223364157166167	0.4792081742976351	0.4942019605346089	T	T	T
0.7221209417670353	0.6793696152791898	0.4945337001948916	T	T	T
0.7222223027380765	0.8788507025955409	0.4933227635904811	T	T	T
0.8332203050186832	0.0783026111646010	0.4921729060108057	T	T	T
0.8331697426904828	0.2786540419184855	0.4927525708326190	T	T	T
0.8331474108672445	0.4793500440654336	0.4946948504028087	T	T	T
0.8331796190953578	0.6793987339530210	0.4948188981764420	T	T	T
0.8333212002421504	0.8788576122937554	0.4933729119595300	T	T	T
0.9436358001014528	0.0780197524329266	0.4916305514983117	T	T	T
0.9422572136692159	0.2784364245329967	0.4931676464349026	T	T	T
0.9430917758253383	0.4795905080740449	0.4958558026121481	T	T	T
0.9439131891314767	0.6794060674963390	0.4943071060775083	T	T	T
0.9440430057385404	0.8787195638899251	0.4930474027224024	T	T	T
0.9992106729166099	0.0070492993655428	0.5015829082635630	T	T	T
0.9972675739045183	0.2063299100406376	0.5017485499247757	T	T	T
0.9967070314080689	0.4078940029239937	0.5057667329198177	T	T	T
0.9990158685889078	0.6084352799382575	0.5040136583871326	T	T	T
0.9995552813822045	0.8077327128293373	0.5027564473942725	T	T	T
0.1106009591510813	0.0070582767331748	0.5017410251458092	T	T	T
0.1111800658088794	0.2062866438865532	0.5051703991707264	T	T	T
0.1024131710796549	0.4081481760939246	0.5104400680097978	T	T	T
0.1100887501217987	0.6085562399181431	0.5032229422819238	T	T	T
0.1107739978391772	0.8078958768538329	0.5023935328100069	T	T	T
0.2221089906805182	0.0073763387498224	0.5022988044794260	T	T	T
0.2164524548996836	0.2060626462251377	0.5079812500309933	T	T	T
0.2283950561584206	0.4090437855904163	0.5187866926725713	T	T	T
0.2217826362288769	0.6088596701851224	0.5030224614032804	T	T	T
0.2221378273630985	0.8079555540049653	0.5019516858360176	T	T	T
0.3336756063565505	0.0073292717252704	0.5020186726392009	T	T	T
0.3391097829907764	0.2060243477414125	0.5077684519679934	T	T	T
0.3270654486649222	0.4091054899978194	0.5186408675769941	T	T	T
0.3336261629573263	0.6088411091707664	0.5028511010055489	T	T	T
0.3333131113272227	0.8078987428994164	0.5016194531051348	T	T	T
0.4450395884581944	0.0070934462972727	0.5014158017411650	T	T	T
0.4443409644335141	0.2064489974328094	0.5049543217520731	T	T	T
0.4527956395601132	0.4081081144132579	0.5098483022061679	T	T	T
0.4454524820139638	0.6085426400454910	0.5023813502402049	T	T	T
0.4447902415790224	0.8078383017097033	0.5018625799023317	T	T	T
0.5563105506153332	0.0071948926230002	0.5013845094753385	T	T	T
0.5583856908488559	0.2064640500986958	0.5018442068972171	T	T	T
0.5586370809846518	0.4078054642922879	0.5052083501544271	T	T	T
0.5565602246143440	0.6083332392537814	0.5032333490501163	T	T	T
0.5559823347437641	0.8078310061305458	0.5023891265733572	T	T	T
0.6669913610117084	0.0072421203874691	0.5019744345377147	T	T	T
0.6674284108187966	0.2069747673592998	0.5011465203871088	T	T	T
0.6675793005000499	0.4075577291888253	0.5027703183851725	T	T	T
0.6668801260592643	0.6082097826458593	0.5037681591896527	T	T	T
0.6667844113933470	0.8078175345882314	0.5029709945095657	T	T	T

0.7778113279660021	0.0073862586473701	0.5023554558289332	T	T	T
0.7779799950731244	0.2073411755479109	0.5014175331105081	T	T	T
0.7778535946940237	0.4076871363879346	0.5026173969484533	T	T	T
0.7776061186068446	0.6081994186686863	0.5043799184747094	T	T	T
0.7776977074740106	0.8079582937041297	0.5035090212037253	T	T	T
0.8885348881042929	0.0071896073496986	0.5020670721485215	T	T	T
0.8882706046926967	0.2069578231733795	0.5012520695642444	T	T	T
0.8879527483153575	0.4076816149293165	0.5032712430912292	T	T	T
0.8884157622780460	0.6082668141528995	0.5044070414595815	T	T	T
0.8886688054872680	0.8078269585108357	0.5032724041804655	T	T	T
0.9991666332234815	0.0585510845948888	0.4027690114282743	T	T	T
0.9981954302353238	0.2588641226104099	0.4040439262060941	T	T	T
0.9985382398411653	0.4597487485174023	0.4070835363651412	T	T	T
0.9991163297049604	0.6597085981653370	0.4049278180615972	T	T	T
0.9994582207480036	0.8590996051347933	0.4039450527453480	T	T	T
0.1104159971308313	0.0588466276443859	0.4032362842258070	T	T	T
0.1110993934514728	0.2619964381496752	0.4117390753439648	T	T	T
0.1094722385343405	0.4576569790737559	0.4110301804275026	T	T	T
0.1101527813487445	0.6595547102980694	0.4041731954132732	T	T	T
0.1106253655274390	0.8593387902208893	0.4036386718430069	T	T	T
0.2224908441513689	0.0598444316831630	0.4044990017947455	T	T	T
0.2192564552240365	0.2606939260467930	0.4133383939650859	T	T	T
0.2230216030025430	0.4556842257442437	0.4155507452849049	T	T	T
0.2217319259951577	0.6598336699668522	0.4035067571299841	T	T	T
0.2220226545832220	0.8594002178252583	0.4033590801397087	T	T	T
0.3332908598628886	0.0597815510711332	0.4041546435839177	T	T	T
0.3361570552341667	0.2605412548759867	0.4130583737594348	T	T	T
0.3323033532350680	0.4557579063641134	0.4153951625083465	T	T	T
0.3334014697570331	0.6594522992853719	0.4033352458118887	T	T	T
0.3334157981197815	0.8595482322387629	0.4031808245173661	T	T	T
0.4452793296315553	0.0588262973644399	0.4028948804465414	T	T	T
0.4442711671072704	0.2620592523220072	0.4114599897318081	T	T	T
0.4458185467534604	0.4575864779313596	0.4103241584825556	T	T	T
0.4452547996340821	0.6598683332768659	0.4034211603313931	T	T	T
0.4449519427953578	0.8593301960194245	0.4032887188321616	T	T	T
0.5563430135768015	0.0587816763564684	0.4026216145289168	T	T	T
0.5573693591702020	0.2586079950555218	0.4038593788120024	T	T	T
0.5568285893966504	0.4593864581485743	0.4063775484179807	T	T	T
0.5564904952463186	0.6595929352361437	0.4042340495183890	T	T	T
0.5562585307148107	0.8593344707284721	0.4036044499059410	T	T	T
0.6673704023927075	0.0584965598984461	0.4030434235092959	T	T	T
0.6678124376311828	0.2591212602167651	0.4029045763748375	T	T	T
0.6670613237237726	0.4600312021153923	0.4049902993157212	T	T	T
0.6671071520244969	0.6597215444503637	0.4050100716881487	T	T	T
0.6670533691446435	0.8591090131528132	0.4040727982587913	T	T	T
0.7777627316651702	0.0584471337974800	0.4033493200616837	T	T	T
0.7779557111946116	0.2593954593595543	0.4033927204113095	T	T	T
0.7779293112599690	0.4603229338402160	0.4051990511274596	T	T	T
0.7778023747317778	0.6595948584289193	0.4058399428955877	T	T	T
0.7778275547670456	0.8591077385856405	0.4045220189467440	T	T	T
0.8881890939330165	0.0582264684284058	0.4029128581231974	T	T	T
0.8879284775647569	0.2592154133675300	0.4030525326534394	T	T	T
0.8884706129378751	0.4604143490907109	0.4056824891054390	T	T	T
0.8883666044255641	0.6595250331700354	0.4056280373065114	T	T	T

0.8885171015547605	0.8589649471212744	0.4042109814387717	T	T	T
0.0549000728978838	0.1577863065957044	0.4325687329496948	T	T	T
0.0529926108599176	0.3579966372144320	0.4362022073377124	T	T	T
0.0542748481477158	0.5580928821763167	0.4342229558084535	T	T	T
0.0550007454870458	0.7582288831992652	0.4325659092425336	T	T	T
0.0550152817395848	0.9574794982688607	0.4316549746867696	T	T	T
0.1656209439267883	0.1575148182083913	0.4321187215037500	T	T	T
0.1665523024241710	0.3614866701434932	0.4531547505210093	T	T	T
0.1659127523052198	0.5572947237193104	0.4352992179180223	T	T	T
0.1662001392516010	0.7584293396399890	0.4318620689036856	T	T	T
0.1665924881518493	0.9577959219881386	0.4317689694927397	T	T	T
0.2777993571939670	0.1599349968449871	0.4384826991120616	T	T	T
0.2777172345390007	0.3586933583061228	0.4425012581867779	T	T	T
0.2777308148397057	0.5575776284094205	0.4348889037049584	T	T	T
0.2775932599775924	0.7584581933920782	0.4312792185070433	T	T	T
0.2778081353553214	0.9580831351691612	0.4316440565383366	T	T	T
0.3900420165664632	0.1576362479687907	0.4317071146255592	T	T	T
0.3886040027565988	0.3615496170549061	0.4527337711455646	T	T	T
0.3895839166423508	0.5572881418216682	0.4348726261940644	T	T	T
0.3891413356247883	0.7584428227622451	0.4313795275569712	T	T	T
0.3890599482066178	0.9577669581384045	0.4315007636660047	T	T	T
0.5007117833252281	0.1580786530957267	0.4325321703656721	T	T	T
0.5022949943245415	0.3579908009226123	0.4357507778372655	T	T	T
0.5012675351979926	0.5579799443218283	0.4333946934724910	T	T	T
0.5006543882311946	0.7582683539040752	0.4318579363524295	T	T	T
0.5005939866783873	0.9576312759948573	0.4312036205696776	T	T	T
0.6124496916362389	0.1569557732061928	0.4300331531333809	T	T	T
0.6121664778059647	0.3578967442779823	0.4325429798161358	T	T	T
0.6119551799266405	0.5581380317379578	0.4335523413432074	T	T	T
0.6115103646120020	0.7581082712831941	0.4325956745874565	T	T	T
0.6116586004602110	0.9574341975993482	0.4315941531027102	T	T	T
0.7227212531150659	0.1571307914008226	0.4308643504656179	T	T	T
0.7226486749238933	0.3577728052960766	0.4320649698693341	T	T	T
0.7223822192882730	0.5584046552190008	0.4338132923807467	T	T	T
0.7224798389680429	0.7580251312861608	0.4332227111783715	T	T	T
0.7224122948051104	0.9574513796209490	0.4320806500935072	T	T	T
0.8330479821422235	0.1571164735723270	0.4307734949625701	T	T	T
0.8330927722441447	0.3578196028002940	0.4322271485611908	T	T	T
0.8330169289070415	0.5584822225069738	0.4341619787830322	T	T	T
0.8330315102756001	0.7579478834582293	0.4334094614776384	T	T	T
0.8331478877383408	0.9574877301204469	0.4320071212647102	T	T	T
0.9431019910824869	0.1569130937197363	0.4300855554143469	T	T	T
0.9432664641783711	0.3579343270049575	0.4329993413450331	T	T	T
0.9435945370257973	0.5583697801656886	0.4342718988962034	T	T	T
0.9438474784784215	0.7579817360487733	0.4329473812487859	T	T	T
0.9438580316192322	0.9573585725663003	0.4317101310440110	T	T	T
0.9984018417967359	0.1276719907603383	0.5622178891293922	T	T	T
0.9961936760208968	0.3278549281596232	0.5660571797129194	T	T	T
0.9976658740221462	0.5303993350058518	0.5669086932651108	T	T	T
0.9996303723196398	0.7291234945767623	0.5641901712819998	T	T	T
0.9997580956172992	0.9284626375478465	0.5630791712097716	T	T	T
0.1094816134556835	0.1264856978246977	0.5633682315139057	T	T	T
0.1021824625976446	0.3288466274202165	0.5698415606177963	T	T	T
0.1092093359711183	0.5303838539533247	0.5651464030526906	T	T	T

0.1107738692498905	0.7290322624597815	0.5630494198741068	T	T	T
0.1108946410523667	0.9283278009279538	0.5627118704930384	T	T	T
0.2204886391121803	0.1257331873200089	0.5651081851721032	T	T	T
0.2247487110034497	0.3270232566071560	0.5653286764358170	T	T	T
0.2207301750762560	0.5317363007651984	0.5675537919971348	T	T	T
0.2220364634343949	0.7289631336258811	0.5623144759980958	T	T	T
0.2220947224688543	0.9282677661199131	0.5625206523510775	T	T	T
0.3353890522677336	0.1257245203986282	0.5649428641510693	T	T	T
0.3306644425585257	0.3271658626583660	0.5651873733424759	T	T	T
0.3350211724530650	0.5317566559943854	0.5672749788970667	T	T	T
0.3334293893027614	0.7289160758472928	0.5617784821015213	T	T	T
0.3335615643580654	0.9281880845849578	0.5623409153685371	T	T	T
0.4460185516061549	0.1267297486057839	0.5630013859452793	T	T	T
0.4530144623099806	0.3288346733741685	0.5694367506220069	T	T	T
0.4463893401632557	0.5304458047521088	0.5645811731367277	T	T	T
0.4447125848431356	0.7289530901823660	0.5623604542033045	T	T	T
0.4447951798963074	0.9283227073918511	0.5622045963589586	T	T	T
0.5570612728426402	0.1278095579042400	0.5621884703756932	T	T	T
0.5593007631769500	0.3278339822322243	0.5655681758779956	T	T	T
0.5577192639030356	0.5301939521220523	0.5659950553296220	T	T	T
0.5557071028973130	0.7291263740249071	0.5635606146882324	T	T	T
0.5557369329208472	0.9286117787970654	0.5627580565650636	T	T	T
0.6670947707784348	0.1280963904930755	0.5621973817941224	T	T	T
0.6678226099757723	0.3283745153468112	0.5637308703783358	T	T	T
0.6674229417341203	0.5293043486590817	0.5651411548773113	T	T	T
0.6666283182599796	0.7291599983507244	0.5644502719167496	T	T	T
0.6667150286603704	0.9285202879216716	0.5633802792384736	T	T	T
0.7779473677489515	0.1281992791236626	0.5621103161227187	T	T	T
0.7778372587471609	0.3284337962040693	0.5631072441430551	T	T	T
0.7775099226108882	0.5290784290898998	0.5649483006636032	T	T	T
0.7776227278597260	0.7290710456511443	0.5647430254926471	T	T	T
0.7777917568350823	0.9285552626762426	0.5633579116010851	T	T	T
0.8884043780858786	0.1279533522413271	0.5622276836132508	T	T	T
0.8877931131241219	0.3284179594865811	0.5641320370624804	T	T	T
0.8878993331533942	0.5296004748579516	0.5658490141216511	T	T	T
0.8886751793342256	0.7291663664386356	0.5648402312898486	T	T	T
0.8888240273139248	0.9285430676400036	0.5633865112965092	T	T	T
0.0548236516535721	0.0265851279613497	0.5903816604715375	T	T	T
0.0525322853757234	0.2243472669332280	0.5918095244231207	T	T	T
0.0485352677752329	0.4302558017098942	0.5983361669336485	T	T	T
0.0548788304291703	0.6280228914627337	0.5925944593877006	T	T	T
0.0552637721317687	0.8273361418181970	0.5914739691027735	T	T	T
0.1661423434709299	0.0258961460706465	0.5910083169659902	T	T	T
0.1628406243856931	0.2185566624683938	0.6002969119218068	T	T	T
0.1586596212560940	0.4331042975659533	0.5975894489834345	T	T	T
0.1659928478105449	0.6285432299511106	0.5915891261575906	T	T	T
0.1665859708895119	0.8274349326792343	0.5910352384025982	T	T	T
0.2780331799255005	0.0261038533803499	0.5911432388652711	T	T	T
0.2778592748605389	0.2173195380674182	0.5947456683675838	T	T	T
0.2779523427650089	0.4422613325108364	0.6136065315800745	T	T	T
0.2777855628252701	0.6284703120557270	0.5912883221992336	T	T	T
0.2778633862319541	0.8273389701876930	0.5907024708814685	T	T	T
0.3896227285368011	0.0259979951433308	0.5907436161446926	T	T	T
0.3927025458919328	0.2186581910127740	0.6000537917960583	T	T	T

```
  0.3967995804522308  0.4331460295889564  0.5973082283817622   T   T   T
  0.3896762538912185  0.6285898947148000  0.5909916179640975   T   T   T
  0.3889748657209022  0.8271928241999635  0.5905864987079459   T   T   T
  0.5006462677729716  0.0266334088891601  0.5901893946672593   T   T   T
  0.5029533984488446  0.2247052991660502  0.5917129429396352   T   T   T
  0.5067152988067083  0.4301953661652496  0.5976392362343209   T   T   T
  0.5007251873576533  0.6280402606537439  0.5915722208024274   T   T   T
  0.5001740213789398  0.8274812857949644  0.5910436358089795   T   T   T
  0.6114095547726335  0.0268489711745662  0.5907017615416981   T   T   T
  0.6131173995584783  0.2264158796713795  0.5912279006200828   T   T   T
  0.6142458858816170  0.4274321261391528  0.5934925368714467   T   T   T
  0.6115641302207189  0.6280361649315120  0.5927183888701977   T   T   T
  0.6112051008975830  0.8274186573724007  0.5916845157896519   T   T   T
  0.7222388378756334  0.0269977809492568  0.5911191035721874   T   T   T
  0.7226998219443086  0.2264803631568248  0.5905569331518277   T   T   T
  0.7229582829630470  0.4269003375185187  0.5919638349810594   T   T   T
  0.7219985764670501  0.6277385095141015  0.5931440838204015   T   T   T
  0.7220726673231308  0.8275053124505481  0.5921701316942889   T   T   T
  0.8332898375310877  0.0268997809270886  0.5912357540327177   T   T   T
  0.8331333605005651  0.2263777190229735  0.5907396962458089   T   T   T
  0.8324746836276127  0.4269508021443484  0.5923298763428716   T   T   T
  0.8329755372988704  0.6278656146563152  0.5934502779915078   T   T   T
  0.8332592521169218  0.8275379252951516  0.5922269109979392   T   T   T
  0.9441336336855667  0.0268801536048082  0.5908212040582869   T   T   T
  0.9427161393627074  0.2262985335267919  0.5912369282223774   T   T   T
  0.9411615229267606  0.4274366997710463  0.5941936095153405   T   T   T
  0.9438452798734301  0.6282298555499378  0.5932142789893785   T   T   T
  0.9443463834299012  0.8274312343771028  0.5919532068083724   T   T   T
  0.2115185267935839  0.2751985604738748  0.7289026062737710   T   T   T
  0.3433340709952390  0.2745735009795187  0.7286238529032072   T   T   T
  0.1669779857020289  0.3514509643719478  0.7296800451255516   T   T   T
  0.2776629940689149  0.3625506475043148  0.7227475154012034   T   T   T
  0.3880479333162347  0.3507098116821060  0.7305223599146212   T   T   T
  0.1625115220124597  0.2989764197509577  0.6378916011140890   T   T   T
  0.2776725992643617  0.2964299435978778  0.6506592680693294   T   T   T
  0.3926316379171157  0.2990129217849814  0.6378950483650043   T   T   T
  0.2174971959509287  0.3897529107449843  0.6533399577956713   T   T   T
  0.3382009203954915  0.3894588043175330  0.6535918921710510   T   T   T
On-top cluster Pd16-Te25
    1.000000000000000
    31.7040281099999994    0.0000000000000000    0.0000000000000000
     0.0000000000000000   31.5721202000000005    0.0000000000000000
     0.0000000000000000    0.0000000000000000   30.0000000000000000
   W    Te   Pd
    90   205    16
Selective dynamics
Direct
  0.0554990292949259  0.0764878616300088  0.3559307328009556   T   T   T
  0.0547106534536538  0.2754259881253587  0.3607552307015144   T   T   T
  0.0544882176516464  0.4783955090000043  0.3660592658261804   T   T   T
  0.0560025511654239  0.6781668756607544  0.3600672140441284   T   T   T
  0.0557679849528831  0.8773751378574786  0.3563597092930014   T   T   T
  0.1667272637387541  0.0767045897818448  0.3570907600141865   T   T   T
  0.1667804335421602  0.2760149917159537  0.3654876293811031   T   T   T
```

0.1619964085001832	0.4777145774852505	0.3653702819681536	T	T	T
0.1671055569273417	0.6783147899135755	0.3590984705685681	T	T	T
0.1667645488798092	0.8776320039741278	0.3563166210800955	T	T	T
0.2781189450844649	0.0767011547806704	0.3581775844357192	T	T	T
0.2786496091571961	0.2748933042331547	0.3642722685465825	T	T	T
0.2826547827604999	0.4786558508916090	0.3627620564376817	T	T	T
0.2781977980020435	0.6791812672467373	0.3581065985267406	T	T	T
0.2779391053951557	0.8778904332339007	0.3560490743314951	T	T	T
0.3889329094250223	0.0769903873907715	0.3586504324582758	T	T	T
0.3883447918755070	0.2749875707844465	0.3650332896404830	T	T	T
0.3851288841308605	0.4788020502323581	0.3633730775488034	T	T	T
0.3895824914143389	0.6792159394648480	0.3559903317434002	T	T	T
0.3891989059705416	0.8781715411232508	0.3556370696072892	T	T	T
0.5002565163841145	0.0773724104769461	0.3586243455072102	T	T	T
0.4990843234176594	0.2766510597873860	0.3663938176656391	T	T	T
0.5070661424018883	0.4794770584594238	0.3588484205006192	T	T	T
0.5005574067765503	0.6795882100996864	0.3554829621240406	T	T	T
0.5001021637690747	0.8784863680147882	0.3554492112683781	T	T	T
0.6111605745681510	0.0771464001713849	0.3584414393102927	T	T	T
0.6123566155734329	0.2753390845536498	0.3640159864808492	T	T	T
0.6137038877651539	0.4794809404185157	0.3604840854930658	T	T	T
0.6116250966860851	0.6798224824316338	0.3555049936667869	T	T	T
0.6111705351112650	0.8782334962897106	0.3552179710749335	T	T	T
0.7220312597324702	0.0768865844706901	0.3577968516413559	T	T	T
0.7212673509123008	0.2755935910453572	0.3643977552076085	T	T	T
0.7185861559278476	0.4792822984566944	0.3602539718902145	T	T	T
0.7229902340711483	0.6795010949467746	0.3570637076746052	T	T	T
0.7226211018784277	0.8780819991438705	0.3557093325643641	T	T	T
0.8333501308575377	0.0771504730514064	0.3568565240909669	T	T	T
0.8336555973552158	0.2762602007932505	0.3654303124583483	T	T	T
0.8379860158341510	0.4777373756206698	0.3639140290081581	T	T	T
0.8336914250299907	0.6785827573964367	0.3583291246095499	T	T	T
0.8337242629534846	0.8779287299549859	0.3562717469663441	T	T	T
0.9447070785385451	0.0765276488736529	0.3558405286771391	T	T	T
0.9453857562345804	0.2756225299632715	0.3608932836208041	T	T	T
0.9460745730109836	0.4784905900120456	0.3656938543212807	T	T	T
0.9445691778614992	0.6782047799863798	0.3599133475746872	T	T	T
0.9445969161434407	0.8775021739046055	0.3563769164072250	T	T	T
0.0001445775255252	0.0056460237866656	0.3625398955176226	T	T	T
0.9995183308513522	0.2044632435776458	0.3655331390802871	T	T	T
0.0002962161569625	0.4064345135365010	0.3725915641863236	T	T	T
0.0003342292560221	0.6072569679910753	0.3690205100218510	T	T	T
0.0001831873733417	0.8065620827306109	0.3642925206844722	T	T	T
0.1113123115604610	0.0057795712542171	0.3629370204125755	T	T	T
0.1119478507193478	0.2047380023337841	0.3657520203681103	T	T	T
0.1067898539958131	0.4062276691349353	0.3727332065709288	T	T	T
0.1110912297079148	0.6073986703294781	0.3686300054868197	T	T	T
0.1113784575669071	0.8067354457977470	0.3641918171006976	T	T	T
0.2221697124941344	0.0058700212066093	0.3636558552166871	T	T	T
0.2211864607675858	0.2044433837510868	0.3678862826746906	T	T	T
0.2297563866445493	0.4072994236126882	0.3706896626892652	T	T	T
0.2233501472856293	0.6079176834348935	0.3665722155884680	T	T	T
0.2222673315486325	0.8070226924056972	0.3636156244408260	T	T	T
0.3334469434665239	0.0059590147500377	0.3642121960056005	T	T	T

0.3333414865486622	0.2045658116931668	0.3704414880235057	T	T	T
0.3342404094349486	0.4059167397222451	0.3716742728122141	T	T	T
0.3330859438779021	0.6090882064546120	0.3660525278961962	T	T	T
0.3335501210363296	0.8073370022705300	0.3627911620201631	T	T	T
0.4445945261461245	0.0063544808793864	0.3641855673009525	T	T	T
0.4446067020233099	0.2048933374128603	0.3685568360830604	T	T	T
0.4382379618083700	0.4074853227437578	0.3693239233189939	T	T	T
0.4459326308781626	0.6084468492477419	0.3629655182725495	T	T	T
0.4446976916974538	0.8075533568137930	0.3620952039739941	T	T	T
0.5555581859849572	0.0063186981462746	0.3640055175316728	T	T	T
0.5549806756600855	0.2054479742623864	0.3693339950141622	T	T	T
0.5627303564109526	0.4078894752241174	0.3674770696458408	T	T	T
0.5561554655472003	0.6093604759459386	0.3630970907550460	T	T	T
0.5554435113252053	0.8076571533245108	0.3619862166955273	T	T	T
0.6667812689637598	0.0060789238592174	0.3637298148177419	T	T	T
0.6673912794945136	0.2046100429838835	0.3701522976091609	T	T	T
0.6658710742354776	0.4074311073991133	0.3695201636588188	T	T	T
0.6680789456525063	0.6093268874534203	0.3643563783918538	T	T	T
0.6670138955366969	0.8075459733300057	0.3622953674871192	T	T	T
0.7780187152828331	0.0061533971914392	0.3633436993618274	T	T	T
0.7778454582413906	0.2050305543735599	0.3681527351049311	T	T	T
0.7707034265700324	0.4078965904793696	0.3690389368971676	T	T	T
0.7779301725901027	0.6081613083316184	0.3650405769261673	T	T	T
0.7783788187924849	0.8072819775982264	0.3633932653500505	T	T	T
0.8889279519336306	0.0060344015798973	0.3628710665381571	T	T	T
0.8886317773008227	0.2049310050000408	0.3657714121121756	T	T	T
0.8938648834629029	0.4063032845949796	0.3721051840545806	T	T	T
0.8896150520124524	0.6074311572489021	0.3679178266979111	T	T	T
0.8890217754646899	0.8068904758217194	0.3640531602466418	T	T	T
0.0001135254462048	0.0573970145799997	0.2903271920382420	T	T	T
0.0000011824253098	0.2578568215784435	0.2944559197434156	T	T	T
0.0004665920761699	0.4578883750529535	0.3003111033109626	T	T	T
0.0003695373159130	0.6571799474699727	0.2954130517471081	T	T	T
0.0001537600985630	0.8573137639009648	0.2913673365191933	T	T	T
0.1117015098363085	0.0580458857943659	0.2911086715918604	T	T	T
0.1131613099884617	0.2603175529575292	0.2970262325893917	T	T	T
0.1088460234828937	0.4566390598813794	0.2998349458511416	T	T	T
0.1110491752853885	0.6572362337540353	0.2948907611346877	T	T	T
0.1111896757412492	0.8575826363364797	0.2912489775178926	T	T	T
0.2228959328085109	0.0584718285963766	0.2921342190317678	T	T	T
0.2221982955267572	0.2596178460920588	0.2988829036881808	T	T	T
0.2218381059947122	0.4597156349795562	0.3014194248329416	T	T	T
0.2221069986144730	0.6581784419665438	0.2936579259023442	T	T	T
0.2222297379528048	0.8582038947817271	0.2910090067611221	T	T	T
0.3337783202466419	0.0585718704599048	0.2927357736948607	T	T	T
0.3338730608081996	0.2566081998127151	0.2989154896551391	T	T	T
0.3338974076955158	0.4551693679960210	0.2965156201571120	T	T	T
0.3327872928217242	0.6588500980276604	0.2922311131979990	T	T	T
0.3333577265397141	0.8589176323825372	0.2905010880528162	T	T	T
0.4446403139951037	0.0594923763329825	0.2930601962765594	T	T	T
0.4447801489932864	0.2593965383824688	0.2995358680229868	T	T	T
0.4445675391771747	0.4594441205551865	0.2993761162637772	T	T	T
0.4447359832917632	0.6594075871923130	0.2904752745572973	T	T	T
0.4445836898309402	0.8593915034244790	0.2900141885000354	T	T	T

0.5555428260337175	0.0594038994539669	0.2928691506073712	T	T	T
0.5545837198385601	0.2594802619003797	0.2999924750461279	T	T	T
0.5594198253024745	0.4568531333030100	0.2935881774429339	T	T	T
0.5562149290533357	0.6600282923730457	0.2902557484232398	T	T	T
0.5555122081808432	0.8594196843739711	0.2899099126888053	T	T	T
0.6662184066896224	0.0588953940994200	0.2924301503054336	T	T	T
0.6667768006781377	0.2564154134799491	0.2983516873998804	T	T	T
0.6670368070417338	0.4565557348301797	0.2944305522712667	T	T	T
0.6679397753679349	0.6599464486641599	0.2912813482536096	T	T	T
0.6670723577296492	0.8592004199844270	0.2901454541137840	T	T	T
0.7772676582996371	0.0587043500709454	0.2918184710744456	T	T	T
0.7778623658992433	0.2601321917030817	0.2992147540427154	T	T	T
0.7792536884408002	0.4595947090057955	0.2991001941836665	T	T	T
0.7788719873791018	0.6588587611503958	0.2925328895383979	T	T	T
0.7784543826253283	0.8584319446165254	0.2908441697630155	T	T	T
0.8884765933615075	0.0581834570240841	0.2910009310655496	T	T	T
0.8871081222336323	0.2604585490306144	0.2969793545349854	T	T	T
0.8921819296718155	0.4567047144453527	0.2992122416989960	T	T	T
0.8899521078440407	0.6573829083803666	0.2943706642577481	T	T	T
0.8892257393484643	0.8578418038185073	0.2912133178157527	T	T	T
0.0560167813298894	0.1566354995287472	0.3131504678516179	T	T	T
0.0547170519536016	0.3573981523282766	0.3187556247491192	T	T	T
0.0553712362570623	0.5562824604529153	0.3191670359010108	T	T	T
0.0557511662264987	0.7563483881038755	0.3137079017046958	T	T	T
0.0558529336800149	0.9561239330964206	0.3112282696795086	T	T	T
0.1673003804666564	0.1563864357386464	0.3131593197483761	T	T	T
0.1674351659911605	0.3586173894798568	0.3242725632205878	T	T	T
0.1656010611569649	0.5562443836159088	0.3183674740337404	T	T	T
0.1665925245629844	0.7566374262172562	0.3131158207845359	T	T	T
0.1669795532034219	0.9565137140674670	0.3114731015458438	T	T	T
0.2782205161045972	0.1566191863065541	0.3157870446705478	T	T	T
0.2803078674764483	0.3551871263988113	0.3188040918744810	T	T	T
0.2790357101957222	0.5570128644285240	0.3157528473659121	T	T	T
0.2776653470926823	0.7574421012464605	0.3120356925350821	T	T	T
0.2779855707019054	0.9569418849154682	0.3119140777065566	T	T	T
0.3887997956975532	0.1568124514212354	0.3160676443391470	T	T	T
0.3877612666701065	0.3548453872754673	0.3190384361148045	T	T	T
0.3877669197104597	0.5570630419350397	0.3155777530356884	T	T	T
0.3888431828783111	0.7579575120168960	0.3108796435544789	T	T	T
0.3890392655019423	0.9574745377397815	0.3119117818645960	T	T	T
0.5001348479488874	0.1571480889834037	0.3150855298433362	T	T	T
0.4997903208362335	0.3584898556413880	0.3231399761087296	T	T	T
0.5022110490397289	0.5578105224232531	0.3122868728810311	T	T	T
0.5001160974504664	0.7583182008956026	0.3101900739515279	T	T	T
0.5000562015116971	0.9577310520187063	0.3116011780435112	T	T	T
0.6110350006800420	0.1572966220662387	0.3163292556327711	T	T	T
0.6134410611840614	0.3550516121189671	0.3164169922667147	T	T	T
0.6129348424107486	0.5582688623993776	0.3139426271834130	T	T	T
0.6115688285855971	0.7584974728511632	0.3105530711569298	T	T	T
0.6111431669265582	0.9575769201814169	0.3114957808172947	T	T	T
0.7219037194681404	0.1568732296739737	0.3153755274284630	T	T	T
0.7201970690386099	0.3554020073699017	0.3178591887800581	T	T	T
0.7225564843520813	0.5577465301458311	0.3133982421962184	T	T	T
0.7232033438723457	0.7579396039969548	0.3114826336176100	T	T	T

0.7223499747904241	0.9570963278328357	0.3115142328533739	T	T	T
0.8326002496212531	0.1570384559738106	0.3136486393243642	T	T	T
0.8332249770549666	0.3588822330719217	0.3237973016105762	T	T	T
0.8355694018730027	0.5562003692840622	0.3168164076772990	T	T	T
0.8340627506785141	0.7569954751805559	0.3125945700681563	T	T	T
0.8333917860617868	0.9568278513844369	0.3112629489727347	T	T	T
0.9440413110793026	0.1565267459763565	0.3128209826261080	T	T	T
0.9458346464096357	0.3574125882729792	0.3184996867818564	T	T	T
0.9454375608639525	0.5563025686726992	0.3187777841479049	T	T	T
0.9447110987666776	0.7562632080850699	0.3136244654498229	T	T	T
0.9444768201199554	0.9562995877046266	0.3112763452170349	T	T	T
0.0000261769127540	0.1251107487559693	0.4083220572985662	T	T	T
0.0000927556594571	0.3245154796457289	0.4138932490067481	T	T	T
0.0001604908816668	0.5297680969542259	0.4168562775965622	T	T	T
0.0001885024771349	0.7285406733313512	0.4103271168760549	T	T	T
0.0001962853592364	0.9270894604030977	0.4075376701171287	T	T	T
0.1104841164087101	0.1250066062794314	0.4087565640742000	T	T	T
0.1068267260467022	0.3261574163361635	0.4122840010066873	T	T	T
0.1106164539514760	0.5291154980535994	0.4163670622501326	T	T	T
0.1116373614218667	0.7285754354149203	0.4100586646520177	T	T	T
0.1111890891638042	0.9270812504269478	0.4076426404041029	T	T	T
0.2218074653792639	0.1245843743225378	0.4103205682325584	T	T	T
0.2250845306789871	0.3282410166370588	0.4114531163070662	T	T	T
0.2226379064451063	0.5286346572272616	0.4082162380049351	T	T	T
0.2226921847314472	0.7289211812470338	0.4091763834941383	T	T	T
0.2222779309502633	0.9269299455776407	0.4077801664468613	T	T	T
0.3332297328501564	0.1243595314164303	0.4118768024494464	T	T	T
0.3332142834508075	0.3273590046470152	0.4172197178355486	T	T	T
0.3352199027886461	0.5320902300756158	0.4149304813101042	T	T	T
0.3342376883735138	0.7291268210042875	0.4078480416595365	T	T	T
0.3335670454632700	0.9268528794517865	0.4078631722066848	T	T	T
0.4446114175741521	0.1253036738086848	0.4112726278568788	T	T	T
0.4413081864013774	0.3294240491877066	0.4135575466982230	T	T	T
0.4458529073841108	0.5292987975650346	0.4050902671311643	T	T	T
0.4451330077371411	0.7291555878570981	0.4067520298509120	T	T	T
0.4446416853722414	0.9271507496155154	0.4076734950603855	T	T	T
0.5557340226050478	0.1254405465452995	0.4113535988060378	T	T	T
0.5597587106984586	0.3304062744266818	0.4109378688499182	T	T	T
0.5587461223387203	0.5319477505320058	0.4092746984673994	T	T	T
0.5556732048020036	0.7292965762092806	0.4066618434632868	T	T	T
0.5557008916757175	0.9271300585315522	0.4075028053420777	T	T	T
0.6669113568714862	0.1245781669821948	0.4117240461291927	T	T	T
0.6660454212668964	0.3293479858447376	0.4168668364505392	T	T	T
0.6664399799685793	0.5322324222596779	0.4117073406324982	T	T	T
0.6669287683525634	0.7293804818621666	0.4071534802704638	T	T	T
0.6667771130934765	0.9270308839612329	0.4072449463677693	T	T	T
0.7779260889137816	0.1249086602325778	0.4100749801116873	T	T	T
0.7746588529792980	0.3292664126729802	0.4105762924617669	T	T	T
0.7784200686935578	0.5290928708893919	0.4065593308773626	T	T	T
0.7780624712894132	0.7289683946954859	0.4086516328037700	T	T	T
0.7780440494684181	0.9272644174315294	0.4075860679096608	T	T	T
0.8896695962666351	0.1250944549036526	0.4086243629846370	T	T	T
0.8934695894323430	0.3265270857000034	0.4122073776760070	T	T	T
0.8896449213623832	0.5291433721222485	0.4152994692290055	T	T	T

0.8887813465140159	0.7286248605539967	0.4097346682963792	T	T	T
0.8891123538141323	0.9273414021184139	0.4076344115375703	T	T	T
0.0554920438565249	0.0247195182698748	0.4280365379049250	T	T	T
0.0553134588629688	0.2224441175358803	0.4311132437002974	T	T	T
0.0546782837049877	0.4273266421164388	0.4392275369517400	T	T	T
0.0558815628893138	0.6283277480363887	0.4336733324105018	T	T	T
0.0557399958233801	0.8267252398081208	0.4292730071659465	T	T	T
0.1664003397640494	0.0243488846747250	0.4288419893182555	T	T	T
0.1652114036974932	0.2165770971862005	0.4343896585757266	T	T	T
0.1693822510277896	0.4234119247877750	0.4325703540729232	T	T	T
0.1677890656707156	0.6280841336328585	0.4321908925949548	T	T	T
0.1669970018576684	0.8266920903425339	0.4291146119100445	T	T	T
0.2775294254890220	0.0240592389185001	0.4295142354159203	T	T	T
0.2762510168494642	0.2200911955212401	0.4355229304677496	T	T	T
0.2807313545798907	0.4276827580682381	0.4371643281836973	T	T	T
0.2783975637505351	0.6286298712838556	0.4315736107688477	T	T	T
0.2781921297233786	0.8265906370625532	0.4284433791184018	T	T	T
0.3889815965935369	0.0242029420064225	0.4298575289847688	T	T	T
0.3899690910419272	0.2203293897145578	0.4368266014974590	T	T	T
0.3889275108801253	0.4300330433678695	0.4370272972856327	T	T	T
0.3908909121877703	0.6291016921341238	0.4288914760479567	T	T	T
0.3893601347684105	0.8266188896285444	0.4278048575969926	T	T	T
0.5002070445277238	0.0244306980437878	0.4298401522537547	T	T	T
0.4996195536616804	0.2197264565545235	0.4358576428953785	T	T	T
0.5015046197777218	0.4253786851404504	0.4272377543027934	T	T	T
0.5008743954580945	0.6283295224377997	0.4283581600930682	T	T	T
0.5001379714526895	0.8268216475433771	0.4275739271415632	T	T	T
0.6113514799816171	0.0242220106559353	0.4294884557123994	T	T	T
0.6106260535882930	0.2214181832824929	0.4359253893648282	T	T	T
0.6120842556427823	0.4312627813911490	0.4355029873734108	T	T	T
0.6116475642530576	0.6299634555544157	0.4284746004240020	T	T	T
0.6111640183374697	0.8267362681653775	0.4273694342983549	T	T	T
0.7226196815575534	0.0241703453591304	0.4291330579484181	T	T	T
0.7232742077791944	0.2211644963444811	0.4360684983379348	T	T	T
0.7195916828414490	0.4289754936991005	0.4353260338378428	T	T	T
0.7228792404048817	0.6284883478232018	0.4300981375271060	T	T	T
0.7222404948843120	0.8267842250165989	0.4280997520216349	T	T	T
0.8337363199871175	0.0249135062379274	0.4285398916741608	T	T	T
0.8346594184616696	0.2166911894898408	0.4341445786607820	T	T	T
0.8306862670102063	0.4234924104717870	0.4314051813304430	T	T	T
0.8328636844161854	0.6281412662427435	0.4312347011133078	T	T	T
0.8334630574862987	0.8268621569583611	0.4290313161833644	T	T	T
0.9447807599440765	0.0248823525686774	0.4279593917068033	T	T	T
0.9446411805033563	0.2226325628695314	0.4315609966241905	T	T	T
0.9455280000431456	0.4275554746392460	0.4388818335888319	T	T	T
0.9444600955078831	0.6282101229614465	0.4333980565272901	T	T	T
0.9446008337489095	0.8267503909413652	0.4292139563945455	T	T	T
0.2117878431418571	0.2768329469709025	0.5331314127932435	T	T	T
0.3304395541818046	0.2743287099370156	0.5318774939906176	T	T	T
0.4654883466547478	0.2904673050349873	0.5457432165918694	T	T	T
0.5527808507709697	0.2686511542714539	0.5342419119996638	T	T	T
0.6679736258563368	0.2787024643860406	0.5319617601326588	T	T	T
0.7864865570822203	0.2776340585601773	0.5316342540255687	T	T	T
0.1498151047556839	0.3417718749292143	0.5360867266994571	T	T	T

```
  0.2614274740461818  0.3741732935527766  0.5333474041596311   T   T   T
  0.3788949736576526  0.3503545498414000  0.5527518373438149   T   T   T
  0.4875944163653659  0.3766997509951814  0.5158372682388640   T   T   T
  0.6142967776678321  0.3589419606203972  0.5549461893930379   T   T   T
  0.7364073062287555  0.3740981651802807  0.5339397205737469   T   T   T
  0.8489274236805225  0.3419529091114513  0.5354865327445670   T   T   T
  0.1055729503469061  0.4342763318396766  0.5437464817290568   T   T   T
  0.8939690168080245  0.4340093505175417  0.5417066312274961   T   T   T
  0.2160454980222205  0.4943271959677288  0.5304118890071189   T   T   T
  0.3020079171423230  0.4914494886793987  0.5543379886225343   T   T   T
  0.4403962032430838  0.5001650966934209  0.5268657425887998   T   T   T
  0.5272043437410149  0.4927952972542268  0.5475548552844655   T   T   T
  0.7005989626845763  0.4935041807425491  0.5523096422879784   T   T   T
  0.7859860671477101  0.4946877917901905  0.5288995981956160   T   T   T
  0.1690564297390960  0.4297486850375630  0.6043887080286650   T   T   T
  0.3893404215817077  0.4517259214246416  0.5971932901486271   T   T   T
  0.6093721236597504  0.4528543154251198  0.5927314741108238   T   T   T
  0.8305129110537836  0.4311550314764238  0.6027430519535327   T   T   T
  0.1634042543423101  0.2960728660281727  0.4648600459608211   T   T   T
  0.2731582347466536  0.3062435419477582  0.4788436220905263   T   T   T
  0.3920920173911194  0.3013502806508975  0.4787651390712593   T   T   T
  0.5033772607690342  0.3036144167508672  0.4678799322444754   T   T   T
  0.6075070450182497  0.3075468766653450  0.4786816299180252   T   T   T
  0.7263444368450561  0.3082143775700965  0.4775248054560378   T   T   T
  0.8362367189772346  0.2959720826090282  0.4639491329503511   T   T   T
  0.1883237281916135  0.4163557855513192  0.5203499187467110   T   T   T
  0.3283068247445963  0.4149983496816549  0.5513849418812229   T   T   T
  0.4164216434456035  0.4207453563677414  0.5209298226194725   T   T   T
  0.5515769620325802  0.4143422523944682  0.5474185344430508   T   T   T
  0.6708103121622232  0.4186018348537995  0.5477982405075792   T   T   T
  0.8108183552345136  0.4155140772321884  0.5191113132658328   T   T   T
  0.2747407931501744  0.5075110912272409  0.4713120481233120   T   T   T
  0.5001767308970504  0.5036802631687205  0.4649685840327615   T   T   T
  0.7265667387617474  0.5090637892587444  0.4702661965928598   T   T   T
== On-top cluster Pd13-Te25
    1.0000000000000000
    31.7040281100000030    0.0000000000000000    0.0000000000000000
     0.0000000000000000   31.5721201999999970    0.0000000000000000
     0.0000000000000000    0.0000000000000000   22.0000000000000000
   W    Te   Te   Pd
    90   180    25    13
Selective dynamics
Direct
  0.0551629840568394  0.0769910007191088  0.4861095550782673   T   T   T
  0.0552348461517444  0.2769238162708265  0.4914975146829405   T   T   T
  0.0554372112866358  0.4784781683272100  0.4929193419039000   T   T   T
  0.0554741378065532  0.6782528013472880  0.4900691439402366   T   T   T
  0.0556584459412697  0.8776657947975020  0.4872446445876981   T   T   T
  0.1663721641287833  0.0771348228315965  0.4876645843783544   T   T   T
  0.1645935905929876  0.2762954771671530  0.4952085147669232   T   T   T
  0.1661183515292286  0.4785005291832756  0.4922966785169662   T   T   T
  0.1664151084111797  0.6783009122791089  0.4897926052823835   T   T   T
  0.1665970215792195  0.8776255307100436  0.4873801488837176   T   T   T
  0.2776882288460238  0.0766063298074716  0.4889878909820495   T   T   T
```

0.2772461966540035	0.2760349868842449	0.4979091382905795	T	T	T
0.2770112715934424	0.4789432821933487	0.4926052000965825	T	T	T
0.2777667513662897	0.6786608431302535	0.4897825260443579	T	T	T
0.2777598959289739	0.8775673204409405	0.4877665330753642	T	T	T
0.3887825838938259	0.0768937617675623	0.4900576572394761	T	T	T
0.3867876815493054	0.2752500202205564	0.5010508568086887	T	T	T
0.3831665962651886	0.4791407256062309	0.4963854777316365	T	T	T
0.3893769476357457	0.6785136964685039	0.4895999327843903	T	T	T
0.3890013057814276	0.8777321928606207	0.4878606963669294	T	T	T
0.5000704596666141	0.0768971622151236	0.4899779102577723	T	T	T
0.4998679905816381	0.2767427125073961	0.5030585566257795	T	T	T
0.5066556951279947	0.4782384510428495	0.4897292176917504	T	T	T
0.5000186587890679	0.6789223380753615	0.4887934248199213	T	T	T
0.5000402482270153	0.8778795152829572	0.4875243962113322	T	T	T
0.6111489231403652	0.0768813414323682	0.4895489872225233	T	T	T
0.6135521881682996	0.2755163772282521	0.5001684804405794	T	T	T
0.6138472039605950	0.4797349331548151	0.4942968104122930	T	T	T
0.6110561193679298	0.6790644384279658	0.4882188141242481	T	T	T
0.6110859966622537	0.8777549390226429	0.4870023707786237	T	T	T
0.7224898470085944	0.0767562092406227	0.4882898674578492	T	T	T
0.7234204996719157	0.2764294006703276	0.4967345219116989	T	T	T
0.7240389781598727	0.4795951114505608	0.4913738168690173	T	T	T
0.7231256362234717	0.6789728108219633	0.4883132212019211	T	T	T
0.7224989715778692	0.8777555136305872	0.4867673108899124	T	T	T
0.8335986868040857	0.0771068429865634	0.4870011462750481	T	T	T
0.8344253886990102	0.2765282032132930	0.4948616550620892	T	T	T
0.8342710139605523	0.4788624683620701	0.4916325143023559	T	T	T
0.8341260729986061	0.6786231543568966	0.4889598043791250	T	T	T
0.8336122646623287	0.8778030902994987	0.4869022909947766	T	T	T
0.9447443671835066	0.0771201725581932	0.4858328620156989	T	T	T
0.9447578012291146	0.2766760633859567	0.4909720932641858	T	T	T
0.9446453777369800	0.4784412428186191	0.4926165580106848	T	T	T
0.9447974469171209	0.6782947059220145	0.4898509512740847	T	T	T
0.9445010196651593	0.8777485241230686	0.4870637971946826	T	T	T
0.0001360806700476	0.0060546006711507	0.4952836752806891	T	T	T
0.0007579202537096	0.2056610577965635	0.4985358844388467	T	T	T
0.0001089325855465	0.4068057268083699	0.5022355493053233	T	T	T
0.0002658573174434	0.6071701888644551	0.5003600372169045	T	T	T
0.0001802288782114	0.8068494904376095	0.4977891517644040	T	T	T
0.1110577621825905	0.0061766463998775	0.4960606399382892	T	T	T
0.1101026511198160	0.2053504126413174	0.4984925424068734	T	T	T
0.1107574459314675	0.4073416770689018	0.5025596384252845	T	T	T
0.1109182035876241	0.6072647424674070	0.5003903459209498	T	T	T
0.1111302511479957	0.8067842969581778	0.4978476618089177	T	T	T
0.2222015693269868	0.0059758968897709	0.4972694505686892	T	T	T
0.2225954689141755	0.2051809389226742	0.5025683824386235	T	T	T
0.2223218559525910	0.4077169444713366	0.5029997510104166	T	T	T
0.2222788339405607	0.6076325211738965	0.5003933007190424	T	T	T
0.2221661342487369	0.8067864969836179	0.4978993780892774	T	T	T
0.3334104844909298	0.0057513265974552	0.4981162918212323	T	T	T
0.3323709530529158	0.2042931480497137	0.5050833260312055	T	T	T
0.3307875211519644	0.4073167146443379	0.5070242854808634	T	T	T
0.3332384139790355	0.6081900057477636	0.5009041595859495	T	T	T
0.3334922564009470	0.8068781876883583	0.4978522873918877	T	T	T

0.4443834014278693	0.0060313127900555	0.4983056867081991	T	T	T
0.4445823309120031	0.2047381794056471	0.5057270279641142	T	T	T
0.4360970473175804	0.4084134975739580	0.5070668483666246	T	T	T
0.4452101820722199	0.6074671555425007	0.4988123812979712	T	T	T
0.4444523819079420	0.8070211486475664	0.4978146731821796	T	T	T
0.5554980199192970	0.0059928992664849	0.4979925515575935	T	T	T
0.5554627606859948	0.2047934905463644	0.5056434942476545	T	T	T
0.5637013362220527	0.4080631217771571	0.5050068273821092	T	T	T
0.5555110721679020	0.6086880739205519	0.4986886434045554	T	T	T
0.5553366531457673	0.8071178175435533	0.4971326574307625	T	T	T
0.6666491463163854	0.0059121236410658	0.4972172320972234	T	T	T
0.6676248860796029	0.2044335477160650	0.5041081832133947	T	T	T
0.6685128859629944	0.4084766064572199	0.5057999444893890	T	T	T
0.6672788358261377	0.6086314857006518	0.4989323844304856	T	T	T
0.6667408164399358	0.8070262245621366	0.4967606750605792	T	T	T
0.7779971437277921	0.0060584598285124	0.4964468687289524	T	T	T
0.7780925138492020	0.2053096995433877	0.5015525446017780	T	T	T
0.7788304524262163	0.4080517483576407	0.5018426748621356	T	T	T
0.7788372506580419	0.6080806592648413	0.4990494695278112	T	T	T
0.7782975513837769	0.8070402492437838	0.4971080528553222	T	T	T
0.8890021944720429	0.0062787579439163	0.4956133968110835	T	T	T
0.8886716361351249	0.2053794901864474	0.4981316314208544	T	T	T
0.8889019440083125	0.4073550573426878	0.5021529015184600	T	T	T
0.8894745264145348	0.6073656169108951	0.4996813508368778	T	T	T
0.8891889529497946	0.8069040788367661	0.4975723474705543	T	T	T
0.0000424344954300	0.0577399588341518	0.3966217247001631	T	T	T
0.0001027799443747	0.2584410899327365	0.4013708645979508	T	T	T
0.0002459695265273	0.4583218686406578	0.4039940926417576	T	T	T
0.0002624503986360	0.6581738613370844	0.4011102799621289	T	T	T
0.0001381468207681	0.8576150125512240	0.3984663698144043	T	T	T
0.1113046013267612	0.0582274192703607	0.3977863142257061	T	T	T
0.1115985022371448	0.2588656924902458	0.4025688207350021	T	T	T
0.1107889750866224	0.4583639300316459	0.4037905119723887	T	T	T
0.1109269927243809	0.6582329426424184	0.4011708234212207	T	T	T
0.1111889701671975	0.8576599619684572	0.3985018144074311	T	T	T
0.2223592412621520	0.0581020967891729	0.3992466858961289	T	T	T
0.2221716680237281	0.2588629359200776	0.4073823533116436	T	T	T
0.2218622992415346	0.4585560962819930	0.4036930040801477	T	T	T
0.2222437890826477	0.6584033750129381	0.4011821856384464	T	T	T
0.2222624267909210	0.8578268103792294	0.3987794149960134	T	T	T
0.3335665965781890	0.0580940188395091	0.4003444072738453	T	T	T
0.3332323740136427	0.2576191301330812	0.4092658140024025	T	T	T
0.3322477440970321	0.4564207918829081	0.4047806133635070	T	T	T
0.3334431094231078	0.6586282241015021	0.4012127671379641	T	T	T
0.3333901142572924	0.8581579083438485	0.3989773933812083	T	T	T
0.4444615483368377	0.0586782797136321	0.4007127543466478	T	T	T
0.4445621415612394	0.2600645067398397	0.4127121504997945	T	T	T
0.4431305314971123	0.4587096734848691	0.4097838272241581	T	T	T
0.4443682578188579	0.6585463135862855	0.4002412374391639	T	T	T
0.4444514659563351	0.8580683654277087	0.3988202457499882	T	T	T
0.5553994071340489	0.0585957155586892	0.4004055287506185	T	T	T
0.5553276353247520	0.2601489565272291	0.4126855150236279	T	T	T
0.5605730384568669	0.4559987161967594	0.4023590574237545	T	T	T
0.5553814407295871	0.6593386630581323	0.3994102916928284	T	T	T

0.5553496293636818	0.8583702134126272	0.3982567519675659	T	T	T
0.6663766955263437	0.0584930500391438	0.3996650265913191	T	T	T
0.6671563824605053	0.2577485156196069	0.4085124595837971	T	T	T
0.6680965524938342	0.4580202311610299	0.4046796569240276	T	T	T
0.6671260819194929	0.6593275331436707	0.3997395386642758	T	T	T
0.6667211050908513	0.8583612142608378	0.3979840864644140	T	T	T
0.7777101831768759	0.0581985315578484	0.3983899025473593	T	T	T
0.7779221140651349	0.2589354522640582	0.4059816154803674	T	T	T
0.7788831249921269	0.4589696753752590	0.4025009357277372	T	T	T
0.7786863941831560	0.6588566055359342	0.3996383789548920	T	T	T
0.7780953450902944	0.8580168915261898	0.3980065982867351	T	T	T
0.8888275234055424	0.0582156341575864	0.3972103187482733	T	T	T
0.8880291305496605	0.2589969418386761	0.4025538933532249	T	T	T
0.8895920590911236	0.4583363367475332	0.4033303992823067	T	T	T
0.8897096406404890	0.6584619021221418	0.4005518546081078	T	T	T
0.8890808355561445	0.8577051272674259	0.3981913604452023	T	T	T
0.0553792265437371	0.1565986657074714	0.4261972176452210	T	T	T
0.0556505821491230	0.3572162717088582	0.4322671350985022	T	T	T
0.0555772642183002	0.5570324281028107	0.4308797066377462	T	T	T
0.0555801670072269	0.7568124846894838	0.4281509160757826	T	T	T
0.0557287725519842	0.9562828117058314	0.4255398569865589	T	T	T
0.1669483609767151	0.1568953408019899	0.4287517054853677	T	T	T
0.1661766311575453	0.3563889621888567	0.4337765144451465	T	T	T
0.1664527028176867	0.5574719847010503	0.4310016067637655	T	T	T
0.1665602307154723	0.7568816938462057	0.4280913631261352	T	T	T
0.1667390913646273	0.9565521508154414	0.4264143755305553	T	T	T
0.2779292805532723	0.1565962399591331	0.4304502472580161	T	T	T
0.2777626755081172	0.3558184253972133	0.4355535439641051	T	T	T
0.2776492261724142	0.5578707384824443	0.4310044286897101	T	T	T
0.2777380072909423	0.7571535720127148	0.4280831846743452	T	T	T
0.2778122727944403	0.9565858402213605	0.4271937330093044	T	T	T
0.3889799151303982	0.1571620498156198	0.4325733185304501	T	T	T
0.3865396079191035	0.3553385752803193	0.4387561616914751	T	T	T
0.3875723572523619	0.5569851981975985	0.4321949067931371	T	T	T
0.3890180852220718	0.7570841772546525	0.4278946899553164	T	T	T
0.3889815601221126	0.9566944171933961	0.4273379120228682	T	T	T
0.5000456428710550	0.1572518433885831	0.4319384464590637	T	T	T
0.4996089309191527	0.3596674149553826	0.4458434396312149	T	T	T
0.5015377806266178	0.5574473202658770	0.4285177207359457	T	T	T
0.4997758339553078	0.7574308974738764	0.4270661974353606	T	T	T
0.4998904260851449	0.9569104003845497	0.4271222305548954	T	T	T
0.6108945230907018	0.1571462899573888	0.4321301925415154	T	T	T
0.6139802498067607	0.3554320908443001	0.4364105522087716	T	T	T
0.6122962376529356	0.5580365221132910	0.4308348424949323	T	T	T
0.6109361251561899	0.7576114992583898	0.4267854557326821	T	T	T
0.6109358841511119	0.9568403966871645	0.4267825500107864	T	T	T
0.7221912761213927	0.1566014042252129	0.4296993175262848	T	T	T
0.7225757653101414	0.3566070954035083	0.4348718826285349	T	T	T
0.7234694263543454	0.5583592054323137	0.4294463537225188	T	T	T
0.7227368326765068	0.7575130057537562	0.4269766272251100	T	T	T
0.7222998103141905	0.9567998533985883	0.4261999803867749	T	T	T
0.8329246693525411	0.1566470422155096	0.4275266801140242	T	T	T
0.8338838620144154	0.3563788812815300	0.4327204955097306	T	T	T
0.8343481458503801	0.5575442289537809	0.4296265031120324	T	T	T

0.8339635996777829	0.7572061463811116	0.4273637698791957	T	T	T
0.8335512500207866	0.9565895687851180	0.4256598101454333	T	T	T
0.9446398430915957	0.1569781406169424	0.4268147474043986	T	T	T
0.9443709583046606	0.3571971502756345	0.4322726332271465	T	T	T
0.9450133175271015	0.5570157462991081	0.4305191786324467	T	T	T
0.9448003040177012	0.7568094252066970	0.4280440891095939	T	T	T
0.9444547299081255	0.9564043735094907	0.4254352785296371	T	T	T
0.0000618142142496	0.1261317491354460	0.5572180730415693	T	T	T
0.9999165050455958	0.3266507899997005	0.5614327288853898	T	T	T
0.0000873242144750	0.5285674737628338	0.5624300224790003	T	T	T
0.0001322532520982	0.7283059520568785	0.5597170100508984	T	T	T
0.0000754580362057	0.9275068724779645	0.5568533827167592	T	T	T
0.1100937765731695	0.1258746126635148	0.5578180755939230	T	T	T
0.1087811522593372	0.3276918309984332	0.5609726595103022	T	T	T
0.1111267122954183	0.5286903236006266	0.5624300177747450	T	T	T
0.1111155451540255	0.7282156742555081	0.5595587901203642	T	T	T
0.1110482376546498	0.9273969059524925	0.5572121493359737	T	T	T
0.2217025470741154	0.1250984392825955	0.5599320010146330	T	T	T
0.2211167242339933	0.3288050357250329	0.5611907216468695	T	T	T
0.2218218680775967	0.5291980740023832	0.5619968940089238	T	T	T
0.2220403908411319	0.7282976631870999	0.5594088208263720	T	T	T
0.2220172648805228	0.9270697817664891	0.5577339685851772	T	T	T
0.3326178684636037	0.1244731995586663	0.5622643307601081	T	T	T
0.3304377248300277	0.3287958049650979	0.5674085394509968	T	T	T
0.3310560979226535	0.5302991504771436	0.5645990014791337	T	T	T
0.3335642170000382	0.7283568260519089	0.5591901710478141	T	T	T
0.3332858432266131	0.9268078365844098	0.5584058599425213	T	T	T
0.4445648810516648	0.1247978617271087	0.5623812421159254	T	T	T
0.4413998648547551	0.3301543571943740	0.5649148585481900	T	T	T
0.4460142928311632	0.5267724034739092	0.5540948968095256	T	T	T
0.4447488062388431	0.7284684910035095	0.5592826190086381	T	T	T
0.4445994564231137	0.9269851618738143	0.5582692889713589	T	T	T
0.5555434069350562	0.1247645682857196	0.5623464681231313	T	T	T
0.5588099394598995	0.3301211269645785	0.5639736019138584	T	T	T
0.5564969204198842	0.5299838773332806	0.5584933447484201	T	T	T
0.5556345179979599	0.7286976261760487	0.5583522629043495	T	T	T
0.5557305599905210	0.9269593347927556	0.5579258152499924	T	T	T
0.6673789889318735	0.1245867305536328	0.5614012583583817	T	T	T
0.6695831252150168	0.3293328839574736	0.5651798520853017	T	T	T
0.6688983463955278	0.5306988810956491	0.5603609594274658	T	T	T
0.6669124235841036	0.7285662313325062	0.5577903023972387	T	T	T
0.6668688447660812	0.9269448382696336	0.5572936803446681	T	T	T
0.7783581682928020	0.1251561075730346	0.5593953457749735	T	T	T
0.7787905339946950	0.3294218552521454	0.5604842156657299	T	T	T
0.7789452338178177	0.5298471594408815	0.5609917605581928	T	T	T
0.7784115194294705	0.7284994103867366	0.5584671975435691	T	T	T
0.7780954596068994	0.9272005933473519	0.5571177801965403	T	T	T
0.8896415693828842	0.1258818660221891	0.5574087570642784	T	T	T
0.8910613553496016	0.3275934536039355	0.5605031455748968	T	T	T
0.8893156897383214	0.5289079442532956	0.5619096449822056	T	T	T
0.8891891544520402	0.7282869118957903	0.5593733324123156	T	T	T
0.8890810920488332	0.9275492980172094	0.5568245387214663	T	T	T
0.0553152919010279	0.0253757987510758	0.5846223708874022	T	T	T
0.0551827856692005	0.2244415081253325	0.5887127692800563	T	T	T

0.0551172611010324	0.4272016545995270	0.5915976302921349	T	T	T
0.0555889890456336	0.6272456870048557	0.5893156151111952	T	T	T
0.0555844427081226	0.8267297176929859	0.5865787771160187	T	T	T
0.1662504857732190	0.0250913838187626	0.5856823234218724	T	T	T
0.1645483891197944	0.2188499093583733	0.5912284914308207	T	T	T
0.1660894298127982	0.4272574350974133	0.5911789206193553	T	T	T
0.1664001416734286	0.6274815957435612	0.5889808921606470	T	T	T
0.1666074155962900	0.8267769684641200	0.5866620510794287	T	T	T
0.2774962110989310	0.0243801184667297	0.5868498068357676	T	T	T
0.2764681256399187	0.2202360290947312	0.5951374879456582	T	T	T
0.2751148238398513	0.4299281021984784	0.5932958474120092	T	T	T
0.2774163535003147	0.6282137710833426	0.5893143412408320	T	T	T
0.2777969269825822	0.8262950107904637	0.5866661288071749	T	T	T
0.3887765678353308	0.0243669016637885	0.5875848145800374	T	T	T
0.3883343762279766	0.2181505167308494	0.5966518285233714	T	T	T
0.3849127483941868	0.4301937103797330	0.5985470225666844	T	T	T
0.3898192609920219	0.6271069777841621	0.5884013036000425	T	T	T
0.3889294738863232	0.8264912681869893	0.5867911637479614	T	T	T
0.5000910887955567	0.0243847083276710	0.5874990740270050	T	T	T
0.5000900087187932	0.2193936713281461	0.5968894167482118	T	T	T
0.5005965028713255	0.4268431104276062	0.5840686560711003	T	T	T
0.5002921104637339	0.6278726887197974	0.5880428908971986	T	T	T
0.5001218355784468	0.8267382730267739	0.5865172415710133	T	T	T
0.6114365477194177	0.0242450122047157	0.5868772771310763	T	T	T
0.6119876872843364	0.2183204720928935	0.5959617165390234	T	T	T
0.6143082812179602	0.4305018964556880	0.5970881944543898	T	T	T
0.6115950839475208	0.6284076161297996	0.5874501881987199	T	T	T
0.6112807791550757	0.8264093863480072	0.5857870429822041	T	T	T
0.7225752592557650	0.0243834456462847	0.5860220229348873	T	T	T
0.7237015283708292	0.2205081272264848	0.5937034676486370	T	T	T
0.7251009540008989	0.4302147960311248	0.5912398960270212	T	T	T
0.7232066724209469	0.6284628151163401	0.5876584871064723	T	T	T
0.7223855579348907	0.8265183659841077	0.5856038907171289	T	T	T
0.8337657630032848	0.0251006846850265	0.5850996415913579	T	T	T
0.8351814708194635	0.2189868823199522	0.5912664538932938	T	T	T
0.8340691182542193	0.4277347226050411	0.5909399141794901	T	T	T
0.8341255652459577	0.6277794924064352	0.5882782776353144	T	T	T
0.8336035348624806	0.8269187038892788	0.5861968595834843	T	T	T
0.9447214196760952	0.0254954562708231	0.5842703233179988	T	T	T
0.9447604852997513	0.2241045934485758	0.5874939315541057	T	T	T
0.9447216384331477	0.4271512877797504	0.5911888627896387	T	T	T
0.9446432304903800	0.6271904321448434	0.5889191255827427	T	T	T
0.9446186468424194	0.8268055267894059	0.5864108414335620	T	T	T
0.2160318810256610	0.2693965006148129	0.7215012642669699	T	T	T
0.3285951538780254	0.2743632126496584	0.7244903277624130	T	T	T
0.4502064030692777	0.2751975400282637	0.7244763725171824	T	T	T
0.5516107738916100	0.2734076365289754	0.7229480307524502	T	T	T
0.6715760700084771	0.2756055910946500	0.7243011210031145	T	T	T
0.7831796511947841	0.2697476859732695	0.7213693059462878	T	T	T
0.1567139211026715	0.3368838059953510	0.7463843127637235	T	T	T
0.2710677031001902	0.3711167865112899	0.7294370861366989	T	T	T
0.3857198447217846	0.3518586897533693	0.7471837606505879	T	T	T
0.4986006521612491	0.3761353349777155	0.7143557278843857	T	T	T
0.6119568659220053	0.3526152047071658	0.7461737123999649	T	T	T

```
  0.7264807273159029  0.3723436555311101  0.7265812215896587   T   T   T
  0.8411859175319537  0.3379075728436686  0.7449286342723554   T   T   T
  0.1124901743265764  0.4215719694828638  0.7499909278245257   T   T   T
  0.8850260395839428  0.4236946813105551  0.7483432205853492   T   T   T
  0.2193409122268978  0.4872042438748855  0.7206796887766936   T   T   T
  0.3080775196522257  0.4938348637439362  0.7392923117829542   T   T   T
  0.4523206755875885  0.4980870626581382  0.7004798168972169   T   T   T
  0.5421919044332661  0.4981509959774987  0.7091516452585458   T   T   T
  0.6893273674659399  0.4947886200667447  0.7244809793061018   T   T   T
  0.7782566793082215  0.4881095881762286  0.7278141115032869   T   T   T
  0.1708552271316174  0.4585390108508435  0.8246179448181385   T   T   T
  0.3896049728904278  0.4671904722004946  0.7938039246236774   T   T   T
  0.6119113257419295  0.4685622164100847  0.7914943793742242   T   T   T
  0.8279639983616633  0.4539951334247543  0.8300594881679333   T   T   T
  0.1603850975743830  0.2967864728119838  0.6418276726613052   T   T   T
  0.2722328121129324  0.3059390427597519  0.6497563134822706   T   T   T
  0.3881001896958431  0.3010117034781696  0.6474790906412830   T   T   T
  0.5006207432386257  0.3075659517110735  0.6410237214397168   T   T   T
  0.6126499040642256  0.3015614935607063  0.6462486776183074   T   T   T
  0.7275350531693223  0.3064572869550138  0.6487136683281960   T   T   T
  0.8383756266409920  0.2972181249245422  0.6405574152189939   T   T   T
  0.1976562664112516  0.4078870819366491  0.7328989986232239   T   T   T
  0.3380443486170551  0.4179195865286655  0.7287784583619865   T   T   T
  0.4283069305209000  0.4189541335369607  0.7124970416762105   T   T   T
  0.5683611528479600  0.4199375442182660  0.7153819626445608   T   T   T
  0.6589436083754390  0.4186886548434720  0.7219960103162408   T   T   T
  0.7996359422753494  0.4090988829249838  0.7340021240810698   T   T   T
== Embedded cluster Pd07-Te02-VTe02
   1.00000000000000
     24.6586885454000111    0.0000000000000000    0.0000000000000000
      0.0000000000000000   25.2576961516000082    0.0000000000000000
      0.0000000000000000    0.0000000000000000   30.0000000000007034
   W    Te   Pd
    56   112     7
Selective dynamics
Direct
  0.0700633590886485  0.0984277711727274  0.4940678290638749   T   T   T
  0.2107983279735320  0.0980169047366587  0.4945009511384918   T   T   T
  0.3528652936770766  0.0970337618024984  0.4952332388312735   T   T   T
  0.5000236734359829  0.1018604267492488  0.4997031155495529   T   T   T
  0.6472101374639875  0.0970771904683106  0.4952980223343387   T   T   T
  0.7893074162271433  0.0980609859593416  0.4945354367293408   T   T   T
  0.9300243748007374  0.0984036128822523  0.4940542476267205   T   T   T
  0.0693270556747001  0.3497656442744624  0.4984312490408543   T   T   T
  0.2067460723074761  0.3484589620734164  0.4987480726135276   T   T   T
  0.3430448986071105  0.3478123924898839  0.4991617274264442   T   T   T
  0.5000337966721341  0.3083991345475733  0.4902805050642851   T   T   T
  0.6570807898765271  0.3478251852002500  0.4992605204513831   T   T   T
  0.7934188894956842  0.3484428741461440  0.4988090516092344   T   T   T
  0.9309384246592046  0.3498215389674951  0.4985069308413759   T   T   T
  0.0702628529209026  0.6000604495466194  0.4979914762578977   T   T   T
  0.2093355231646740  0.6004603617832808  0.4986820529475021   T   T   T
  0.3625118107798063  0.6021818671808802  0.4935465760827336   T   T   T
  0.5000273672714433  0.6090215720932342  0.4905631987256467   T   T   T
```

0.6375895080702173	0.6021725162608766	0.4935650658951838	T	T	T
0.7906790056991413	0.6004462596908988	0.4986684668723010	T	T	T
0.9297767776655671	0.6000599374757138	0.4979872422963725	T	T	T
0.0706790676923120	0.8489011027608461	0.4933926113795978	T	T	T
0.2122107824478792	0.8490874276987024	0.4933708568306509	T	T	T
0.3555848759127298	0.8510479413810944	0.4931054162667631	T	T	T
0.5000232793110770	0.8525796331916761	0.4926388769049769	T	T	T
0.6444787028070178	0.8510627888797073	0.4931406939224105	T	T	T
0.7878204142897456	0.8491243652313580	0.4934031753221940	T	T	T
0.9293648536714297	0.8489120469060787	0.4934290363928271	T	T	T
0.0000449182456936	0.0089326591893241	0.4992906287206369	T	T	T
0.1406443848666431	0.0090286545524630	0.4997148226620125	T	T	T
0.2824087164253442	0.0093521331337191	0.5006311614578788	T	T	T
0.4284208796166921	0.0116379736910811	0.5020899484024255	T	T	T
0.5716610959325048	0.0116592901888115	0.5021225155079334	T	T	T
0.7176020146589932	0.0093954962232452	0.5006435550876014	T	T	T
0.8594094070130289	0.0090445101915134	0.4997243832251493	T	T	T
0.0000734009009971	0.2595896893602891	0.5022673511456541	T	T	T
0.1392637960743877	0.2590362674898262	0.5023149658657783	T	T	T
0.2783330646532018	0.2574306937761640	0.5037841904441069	T	T	T
0.4113584335088013	0.2549562883885187	0.5078844177453850	T	T	T
0.5887248933537647	0.2549571138822612	0.5079764831496121	T	T	T
0.7218409038480240	0.2574665780187916	0.5039266895994680	T	T	T
0.8609779946770174	0.2590989408921586	0.5024588016316924	T	T	T
0.0000835446259447	0.5108718632709393	0.5049901991251128	T	T	T
0.1400046496052419	0.5107811643964952	0.5058589173894571	T	T	T
0.2787892781544837	0.5122309079757243	0.5081390145188857	T	T	T
0.4391167025502927	0.5168333996905572	0.4956430910753641	T	T	T
0.5610269133385951	0.5168423520204267	0.4956816327818506	T	T	T
0.7213267337417568	0.5121548107326298	0.5081003201715851	T	T	T
0.8601106194902717	0.5107822826511841	0.5058584462979001	T	T	T
0.0000220559940779	0.7601888977021874	0.5010186695759153	T	T	T
0.1416007408096702	0.7603812130888323	0.5011471747829903	T	T	T
0.2843692096872273	0.7612603404619912	0.5005384100974362	T	T	T
0.4279104627380969	0.7645239260140466	0.4993916464579175	T	T	T
0.5721330500120263	0.7645332289566351	0.4994040920894572	T	T	T
0.7156872302905392	0.7612860326453159	0.5005638716713718	T	T	T
0.8584319508462561	0.7604078233599058	0.5011635658988557	T	T	T
0.1409560431371824	0.0749156138168129	0.4281995406858888	T	T	T
0.2826900648752874	0.0748596820892285	0.4289804961512833	T	T	T
0.4291738586296484	0.0798900536526769	0.4322658799880002	T	T	T
0.5709095137579768	0.0798746198600321	0.4322980081113489	T	T	T
0.7173729090938200	0.0749794043788008	0.4290112676934423	T	T	T
0.8591194461617909	0.0749179283600104	0.4281879681762376	T	T	T
0.0000498070430683	0.0750585998510413	0.4278535046865273	T	T	T
0.1385269261108032	0.3264994409388797	0.4316689041094388	T	T	T
0.2761898178177237	0.3233440996203423	0.4325889086013960	T	T	T
0.4151745221919930	0.3169284256239597	0.4319495210572604	T	T	T
0.5849645273862096	0.3169072866379801	0.4320787437264406	T	T	T
0.7238867097459076	0.3233438520406380	0.4326798406782529	T	T	T
0.8616723889770614	0.3264822946394256	0.4317569153672925	T	T	T
0.0000796021778078	0.3269616425560231	0.4316808622617893	T	T	T
0.1410963551515038	0.5742433068507070	0.4325369075284231	T	T	T
0.2835360403330604	0.5739338327478927	0.4350831652502510	T	T	T

0.4288087684153787	0.5833343154198136	0.4251794316057838	T	T	T
0.5712442097118882	0.5833452641037337	0.4252181630693864	T	T	T
0.7164715973477263	0.5738955059786712	0.4350663035999053	T	T	T
0.8589235202390980	0.5742347257141809	0.4325385297213359	T	T	T
0.0000105975153901	0.5748869746366712	0.4323195124228559	T	T	T
0.1417101556722827	0.8237525972040762	0.4279530425971187	T	T	T
0.2841975562215188	0.8255408127823456	0.4281465373340014	T	T	T
0.4278467970313099	0.8294086294762928	0.4275570583156346	T	T	T
0.5722482939942695	0.8294464402483125	0.4275911089002655	T	T	T
0.7158488501607478	0.8255652839325851	0.4281629246802937	T	T	T
0.8583229327054133	0.8238283359105805	0.4279875114290446	T	T	T
0.9999983150435231	0.8239073648893515	0.4279633378104973	T	T	T
0.0697094308739934	0.1971278114214478	0.4491592600370289	T	T	T
0.2098805057936213	0.1970020744249613	0.4497975794574650	T	T	T
0.3502973596846660	0.1959979599905258	0.4504341464632867	T	T	T
0.5000123802076974	0.2041823023671476	0.4563554961788401	T	T	T
0.6498234906091302	0.1960089011311320	0.4505317355750287	T	T	T
0.7903168877659981	0.1971303104348347	0.4500002958765804	T	T	T
0.9303924891008160	0.1971238099837842	0.4491976125246941	T	T	T
0.0706365903436690	0.4483282591839210	0.4536621933393089	T	T	T
0.2117459970195889	0.4488727169620065	0.4556188740405991	T	T	T
0.3515279536056081	0.4509616025296456	0.4543097666701240	T	T	T
0.5000802979635618	0.4690744746942055	0.4298815840109957	T	T	T
0.6485840387223650	0.4508919405988946	0.4542778157326041	T	T	T
0.7885166978252901	0.4488032688813006	0.4555640387736306	T	T	T
0.9295686705535140	0.4483200371548444	0.4536886955235139	T	T	T
0.0710502601811001	0.6967262550218120	0.4506750770465289	T	T	T
0.2127634913146157	0.6969423396551333	0.4508655018643970	T	T	T
0.3564247003421663	0.7005654450260023	0.4491867594663546	T	T	T
0.5000174520658414	0.7061006146187429	0.4462963750668579	T	T	T
0.6436301451677129	0.7005978733520579	0.4492194830094577	T	T	T
0.7872752230684866	0.6969492090485924	0.4508668468883245	T	T	T
0.9289658076969771	0.6967343862812289	0.4506859953443066	T	T	T
0.0709412067665146	0.9467008288559898	0.4474783021061230	T	T	T
0.2127149004466763	0.9474073766941433	0.4481199441891338	T	T	T
0.3565724613736978	0.9500697475384833	0.4496324598536879	T	T	T
0.5000392704779526	0.9519359024655929	0.4493030734938613	T	T	T
0.6434389902030391	0.9500620168035389	0.4496397689657176	T	T	T
0.7873010314007562	0.9474537000540096	0.4481781164608741	T	T	T
0.9291138092248697	0.9467009382588171	0.4475381774271071	T	T	T
0.0000511318775559	0.1606088783360967	0.5464961964588683	T	T	T
0.1402211210505776	0.1600819312124107	0.5466090339427222	T	T	T
0.2813350819667182	0.1588473264084866	0.5474247275167298	T	T	T
0.4217049314073891	0.1567860178473008	0.5507877845293664	T	T	T
0.5783368670885523	0.1567778677476853	0.5508177876957749	T	T	T
0.7188669758961415	0.1588666988919550	0.5475613112044494	T	T	T
0.8600342590953168	0.1600316373687252	0.5466787950242160	T	T	T
0.0001966879015296	0.4126813052324630	0.5507074068590251	T	T	T
0.1395319289806277	0.4121229766926537	0.5515397799096139	T	T	T
0.2754577943926849	0.4104528894994201	0.5561371743558277	T	T	T
0.7246629436266189	0.4104518848528082	0.5561785735202882	T	T	T
0.8606033252332255	0.4121312271856252	0.5515571167988852	T	T	T
0.0000132311197509	0.6634476128944026	0.5486665104959060	T	T	T
0.1411579613522701	0.6636610853928135	0.5494235702728973	T	T	T

```
  0.2866895248052683  0.6620479534159674  0.5454370797268915   T   T   T
  0.4295110290785485  0.6671993483888442  0.5447698352615292   T   T   T
  0.5705306587340759  0.6672123859141299  0.5447772279489768   T   T   T
  0.7133735097532918  0.6620695061267199  0.5454145836634227   T   T   T
  0.8588949565530390  0.6636641643433568  0.5494089121581708   T   T   T
  0.0000352120253740  0.9111946253964656  0.5447197068481502   T   T   T
  0.1416301186328044  0.9109906139574322  0.5448295603871657   T   T   T
  0.2837327958699272  0.9109507119032807  0.5449813811356428   T   T   T
  0.4279155192742879  0.9122410996231535  0.5449120699900557   T   T   T
  0.5720922134111269  0.9122409319756634  0.5449190113258313   T   T   T
  0.7163125687494721  0.9110040383576495  0.5450222633782439   T   T   T
  0.8584183843191675  0.9110349366323419  0.5448730802986811   T   T   T
  0.0702488944371452  0.0323485245082001  0.5654906108670906   T   T   T
  0.2111870572720100  0.0322012064943698  0.5660086897286178   T   T   T
  0.3543187976781865  0.0313963096720017  0.5665772583678422   T   T   T
  0.5000054943279589  0.0326025991467891  0.5681953404499689   T   T   T
  0.6457505624667276  0.0314182645133037  0.5666214763684364   T   T   T
  0.7888345839189240  0.0322027237510067  0.5659997503843215   T   T   T
  0.9298250507822136  0.0323397328281167  0.5654770287610648   T   T   T
  0.0697128140032259  0.2821272381570840  0.5689907053147949   T   T   T
  0.2083646132435046  0.2817313892764426  0.5695847974483984   T   T   T
  0.3459201918762647  0.2858063765172705  0.5738868720632461   T   T   T
  0.5000516293653826  0.2620358659547445  0.5717742374808341   T   T   T
  0.6541882297496201  0.2858289005041183  0.5739855695551108   T   T   T
  0.7918442513474099  0.2817674508219171  0.5697131763342015   T   T   T
  0.9305903731735622  0.2822139257060169  0.5690973875198813   T   T   T
  0.0697247684569095  0.5365046169841863  0.5711235719235923   T   T   T
  0.2095541608790821  0.5385967381536857  0.5726162770122358   T   T   T
  0.3590768674594862  0.5429525758028074  0.5671439790286660   T   T   T
  0.5000182306997918  0.5477790092334690  0.5653207683906998   T   T   T
  0.6410835692782572  0.5429251535739759  0.5671470037945852   T   T   T
  0.7905251113760898  0.5385687534986520  0.5726045396384533   T   T   T
  0.9304069938922256  0.5365033068561251  0.5711089169385076   T   T   T
  0.0708752539952046  0.7853964251020642  0.5663533081061729   T   T   T
  0.2134151442339467  0.7848072210800927  0.5660315741388461   T   T   T
  0.3561721320365111  0.7865764136817471  0.5651773235370469   T   T   T
  0.5000087241312494  0.7878449439285623  0.5644099986257305   T   T   T
  0.6438652517780452  0.7865748817109497  0.5652008909447122   T   T   T
  0.7866192486490953  0.7848530930855949  0.5660614512832153   T   T   T
  0.9291947196717329  0.7853622750450837  0.5663580908156384   T   T   T
  0.4288012687374980  0.4234228118705481  0.6149309959181695   T   T   T
  0.5712261636316753  0.4234150399083508  0.6149182152468352   T   T   T
  0.3755752412060710  0.4444388964981750  0.5418277522760908   T   T   T
  0.5000311233931206  0.4414433455347313  0.5519072270694274   T   T   T
  0.6245389918012423  0.4444283466341293  0.5418569752836723   T   T   T
  0.4380008750872361  0.3461193975165467  0.5579620698696621   T   T   T
  0.5620625247629388  0.3461187533681484  0.5579892102002825   T   T   T
  0.4445545608582255  0.4020259531939186  0.4778334670438286   T   T   T
  0.5555693136645993  0.4020355831742095  0.4778724602702988   T   T   T
== On-top cluster Pd03 (B1-B2-B1)
   1.00000000000000
     24.6586885454000111    0.0000000000000000    0.0000000000000000
      0.0000000000000000   25.2576961516000082    0.0000000000000000
      0.0000000000000000    0.0000000000000000   30.0000000000007034
```

```
   W    Te   Pd
    56   112     3
Selective dynamics
Direct
  0.0715392992474149  0.0997616029439553  0.4969666356712175   T   T   T
  0.2140848303770761  0.0995681658693305  0.4978336820025719   T   T   T
  0.3575160436799898  0.0993307816566746  0.4983107936532396   T   T   T
  0.5004754437422377  0.0990245339718124  0.4982982823612132   T   T   T
  0.6434700407612745  0.0990364773360468  0.4975648568994285   T   T   T
  0.7864950330723665  0.0994721602849082  0.4966967825467287   T   T   T
  0.9293379880191347  0.0997499589330698  0.4964155810422638   T   T   T
  0.0715074988646864  0.3496708465166588  0.4976795019009834   T   T   T
  0.2139022365422910  0.3495530284641414  0.4989816527836941   T   T   T
  0.3570500855352409  0.3490255686742360  0.5000425805923421   T   T   T
  0.4985731237681086  0.3475205237268081  0.4989490454909995   T   T   T
  0.6452491796047130  0.3477043576973974  0.4983434863697761   T   T   T
  0.7871107341842041  0.3492540463540116  0.4985793230497939   T   T   T
  0.9295398447580400  0.3497397679795982  0.4975320427457932   T   T   T
  0.0714934493206995  0.5996836167493058  0.4967371467970506   T   T   T
  0.2141359152647019  0.5996173967894667  0.4977741721198181   T   T   T
  0.3578618299133473  0.5998058789745392  0.4985243067808818   T   T   T
  0.5007305396489933  0.6012699099782242  0.4984546267158932   T   T   T
  0.6427398384722196  0.6013654283250910  0.4980446562318770   T   T   T
  0.7860069428526399  0.5999818101054667  0.4973055583250156   T   T   T
  0.9292834438203539  0.5998108386709751  0.4965693585207109   T   T   T
  0.0716362181484861  0.8497811741385599  0.4960823255506799   T   T   T
  0.2139629792907297  0.8497659490717374  0.4966881949970762   T   T   T
  0.3573015560527520  0.8498224405160025  0.4968744729027297   T   T   T
  0.5005115213668843  0.8498889398439942  0.4966331671163861   T   T   T
  0.6432766363355548  0.8498601304291207  0.4961651317833509   T   T   T
  0.7867983266853235  0.8499182459813154  0.4958602789471382   T   T   T
  0.9296172886232373  0.8498441154054562  0.4956850819338815   T   T   T
  0.0005458498623692  0.0106011011446357  0.5025582295780583   T   T   T
  0.1426239014760871  0.0105461592204732  0.5033594353400392   T   T   T
  0.2856866065682253  0.0105171503790305  0.5040487977226976   T   T   T
  0.4292974869924988  0.0103363511513221  0.5040467249939711   T   T   T
  0.5720241177570565  0.0103019888882608  0.5036993769305010   T   T   T
  0.7145994576338678  0.0103434740596121  0.5029227681442495   T   T   T
  0.8580705624468630  0.0106157031840423  0.5023067645969851   T   T   T
  0.0004170349709843  0.2605445039250265  0.5036343939803177   T   T   T
  0.1428523393820160  0.2604535684702446  0.5045938695008123   T   T   T
  0.2857122624913302  0.2602468005173016  0.5057335863723369   T   T   T
  0.4292389747943984  0.2594587125805503  0.5060762535246358   T   T   T
  0.5718984900711471  0.2595776603429910  0.5053757197846903   T   T   T
  0.7147373888225835  0.2596987838926755  0.5049577827727856   T   T   T
  0.8581077596092060  0.2603492828949574  0.5039850539580167   T   T   T
  0.0004432929465864  0.5105817801770617  0.5033360380032027   T   T   T
  0.1427725287758298  0.5104793810757303  0.5041003767895214   T   T   T
  0.2859226716651735  0.5106454042677588  0.5054403752559734   T   T   T
  0.4301286765426520  0.5112024645709422  0.5060012362653975   T   T   T
  0.5716523393946208  0.5138610253725912  0.5036842185287265   T   T   T
  0.7136191674282639  0.5113268675877063  0.5050412129490129   T   T   T
  0.8578549962819749  0.5108111355557503  0.5039842916970130   T   T   T
  0.0005120706140176  0.7605708468544289  0.5022529404030239   T   T   T
```

0.1427786670898501	0.7606367071630512	0.5028092854484618	T	T	T
0.2856882349580968	0.7607297775691630	0.5036775879601313	T	T	T
0.4287633474020536	0.7611203533407336	0.5037827284712908	T	T	T
0.5717652595140679	0.7612724458539953	0.5032748260326946	T	T	T
0.7150958645235234	0.7612620032417384	0.5030173809878429	T	T	T
0.8581375521120072	0.7608905179378482	0.5023995769263022	T	T	T
0.1434637968115876	0.0762443268056606	0.4318507253976853	T	T	T
0.2861636739118274	0.0763382201767622	0.4326898569588618	T	T	T
0.4288081912057436	0.0764066215076507	0.4327237960938362	T	T	T
0.5714996024197034	0.0763718910743114	0.4323496160144298	T	T	T
0.7146249240388091	0.0763424052945530	0.4315332463086300	T	T	T
0.8577332157782729	0.0765788567055867	0.4308716074287047	T	T	T
0.0007257906601041	0.0763887886678984	0.4309685706547892	T	T	T
0.1435527274842499	0.3257529354917664	0.4328089532783435	T	T	T
0.2861298938001273	0.3255207284749347	0.4341957865018505	T	T	T
0.4278125481908184	0.3245832529021564	0.4340398006243810	T	T	T
0.5716469938913256	0.3252600967938656	0.4340793725124975	T	T	T
0.7156114316767227	0.3249186866282690	0.4330646937372130	T	T	T
0.8576438139531842	0.3258239397227028	0.4324675146332971	T	T	T
0.0005934039796111	0.3259555345109311	0.4318515380854782	T	T	T
0.1435581367473117	0.5751871738523671	0.4319869198944660	T	T	T
0.2865074409320890	0.5751349806039505	0.4332548329692882	T	T	T
0.4291500291593723	0.5754700905869754	0.4334642981918561	T	T	T
0.5715264918218329	0.5782781539550770	0.4321209052850545	T	T	T
0.7139853956291363	0.5758780997636062	0.4327284643870883	T	T	T
0.8570947518551854	0.5754596807201262	0.4318796302730904	T	T	T
0.0004356092713935	0.5754765977989557	0.4311450482226130	T	T	T
0.1433310913271377	0.8259590473659354	0.4308792868897451	T	T	T
0.2858469734309130	0.8255657579549567	0.4316571876399010	T	T	T
0.4287416564466421	0.8256317662199416	0.4315240800053914	T	T	T
0.5716590590073944	0.8259837643098363	0.4309649953932298	T	T	T
0.7148884962599028	0.8258160076855343	0.4308176098042955	T	T	T
0.8579708172887779	0.8259956214478043	0.4304179817541648	T	T	T
0.0007792644685877	0.8257863797331817	0.4303278291570072	T	T	T
0.0720336293424982	0.1984918607020635	0.4523445656574254	T	T	T
0.2146921728239651	0.1983862924958720	0.4534425106299019	T	T	T
0.3571837751339767	0.1981662675434362	0.4543523960416743	T	T	T
0.4999898296818951	0.1975927423240577	0.4539909924198306	T	T	T
0.6433910701228148	0.1978241959387349	0.4534112643244880	T	T	T
0.7863049158892474	0.1984420497884538	0.4527561909103189	T	T	T
0.9292499119010954	0.1985875860470331	0.4518586341316417	T	T	T
0.0719760261837854	0.4478382002297679	0.4523964372855989	T	T	T
0.2148433036862163	0.4478233899680331	0.4537790078001347	T	T	T
0.3580031024645988	0.4478997829796670	0.4552054930813175	T	T	T
0.4988511450617308	0.4485394870018454	0.4533797713312718	T	T	T
0.6444103401519885	0.4487384925753192	0.4528965909096173	T	T	T
0.7856044158846839	0.4482000231899558	0.4538955621246879	T	T	T
0.9289004682739593	0.4480602714256546	0.4523595422706348	T	T	T
0.0719505711893912	0.6979778862297827	0.4511862949158247	T	T	T
0.2147823474285807	0.6979285467369257	0.4523109515539341	T	T	T
0.3574274708418320	0.6980642104740382	0.4529311337043210	T	T	T
0.4999808379025130	0.6988547699817967	0.4524506346726345	T	T	T
0.6433441861618132	0.6991011576650558	0.4521697820978025	T	T	T
0.7861467789285030	0.6984007915297301	0.4517581984910956	T	T	T

0.9290877235110707	0.6981420818111921	0.4509677993914870	T	T	T
0.0719071868151783	0.9484476527216568	0.4512222459867387	T	T	T
0.2145898931347938	0.9486245268817523	0.4520863175488332	T	T	T
0.3574416308937254	0.9487847261020732	0.4523573501939037	T	T	T
0.5003535808694335	0.9486445996234090	0.4521031691666394	T	T	T
0.6430994905843426	0.9485749667169756	0.4514573511440113	T	T	T
0.7862474949668515	0.9486108956400705	0.4509487262061258	T	T	T
0.9293305701460296	0.9485019542937078	0.4507041342327827	T	T	T
0.0003310543856728	0.1618511319359526	0.5483447279715119	T	T	T
0.1425450880580499	0.1616064036765887	0.5492465060026008	T	T	T
0.2855826142491718	0.1610838491458436	0.5498480715249933	T	T	T
0.4290106739088793	0.1605986254926046	0.5503788435610701	T	T	T
0.5723332682694457	0.1607580549586909	0.5498549559769903	T	T	T
0.7153661446399989	0.1608400968410487	0.5490319417096714	T	T	T
0.8581415031008321	0.1614644519694182	0.5483167910423589	T	T	T
0.0004330134503201	0.4122943912925416	0.5488416251206319	T	T	T
0.1423524999809939	0.4121879463585431	0.5496152371924147	T	T	T
0.2854072201944173	0.4117490269626872	0.5507244863247744	T	T	T
0.4279802828149801	0.4124393634221666	0.5521768996709017	T	T	T
0.5719751633132286	0.4157750340332679	0.5394671354762200	T	T	T
0.7163455134273535	0.4124683858794885	0.5510103676464808	T	T	T
0.8584473202085835	0.4121072697070989	0.5493386695886723	T	T	T
0.0002559746380309	0.6624244471662804	0.5476246742207771	T	T	T
0.1425070025526539	0.6624112735241140	0.5481723151004620	T	T	T
0.2856121615001574	0.6622364584255922	0.5489757282543719	T	T	T
0.4288506004504101	0.6631976554082046	0.5493666263576565	T	T	T
0.5719001935251032	0.6638223968332960	0.5492525635739625	T	T	T
0.7151365158087567	0.6632066471186272	0.5484899436514888	T	T	T
0.8579835379475004	0.6626127815157061	0.5477182849922173	T	T	T
0.0003381814230020	0.9120307171237266	0.5474217611895980	T	T	T
0.1425474802105380	0.9119028165955553	0.5479667899842628	T	T	T
0.2855705976265830	0.9116510045872294	0.5482916637576218	T	T	T
0.4290048305856980	0.9116176600674496	0.5482825957996021	T	T	T
0.5721729219070373	0.9116958988000953	0.5479450287467700	T	T	T
0.7151558983712032	0.9116696131822033	0.5473158396427167	T	T	T
0.8581557988568618	0.9121916002638069	0.5469840528130059	T	T	T
0.0711751910514429	0.0341347622981320	0.5686090721015755	T	T	T
0.2137649384299808	0.0336975595210677	0.5692472192853616	T	T	T
0.3574403386480491	0.0333006420271797	0.5694878508777971	T	T	T
0.5008866159038439	0.0329786713967282	0.5696334182216206	T	T	T
0.6438349584870401	0.0333188620175717	0.5690751265249657	T	T	T
0.7866602020861839	0.0335319968104099	0.5680183631940466	T	T	T
0.9291022725321055	0.0341008649395180	0.5679822402802035	T	T	T
0.0711027008854922	0.2845091619482061	0.5695801288005651	T	T	T
0.2136420035698505	0.2839016941493006	0.5706617643564875	T	T	T
0.3570543754492017	0.2832960768592747	0.5713676535944415	T	T	T
0.5008555576155339	0.2811013799039524	0.5723473189788618	T	T	T
0.6437361178008830	0.2814164045026786	0.5717028457824838	T	T	T
0.7872303858366994	0.2836385479756846	0.5699432939053789	T	T	T
0.9294418141581293	0.2842665394863061	0.5693177719787904	T	T	T
0.0712374034703748	0.5349088354885008	0.5689874928869232	T	T	T
0.2136147112170916	0.5346927815611258	0.5699167290443604	T	T	T
0.3571919643854013	0.5353901666621517	0.5707266821910393	T	T	T
0.4999956034291984	0.5371200240526440	0.5719312532282023	T	T	T

```
  0.6440608569295622  0.5368025530160977  0.5713516451079119   T   T   T
  0.7866901381004570  0.5353452359211470  0.5694078414142565   T   T   T
  0.9294845740217643  0.5351760348185267  0.5688452457264462   T   T   T
  0.0713792098631641  0.7846086519587737  0.5679527836362616   T   T   T
  0.2138499901688518  0.7847385699460090  0.5685503826901523   T   T   T
  0.3570124595256783  0.7851750145413185  0.5689648733581991   T   T   T
  0.5003922470652602  0.7854552795252127  0.5688307366254516   T   T   T
  0.6437141573440421  0.7853798450132243  0.5683715257527577   T   T   T
  0.7869789969078769  0.7852078000325300  0.5679395722125161   T   T   T
  0.9293395711224705  0.7849386166283207  0.5676929404363401   T   T   T
  0.5126031736982113  0.3802126987329539  0.6004420969996234   T   T   T
  0.5720684916788371  0.4736640474410173  0.6104347372392700   T   T   T
  0.6322129950988418  0.3804644598174383  0.5999360757341963   T   T   T
== On-top cluster Pd05 (B1-B2-B1-B2-B1)
   1.00000000000000
    24.6586885454000111    0.0000000000000000    0.0000000000000000
     0.0000000000000000   25.2576961516000082    0.0000000000000000
     0.0000000000000000    0.0000000000000000   30.0000000000007034
   W    Te   Pd
    56   112     5
Selective dynamics
Direct
  0.0719417407930088  0.0994528881455440  0.4978908280642498   T   T   T
  0.2145329841324366  0.0994193150479132  0.4981629231045867   T   T   T
  0.3565141793573313  0.0992143714557064  0.4981879391474426   T   T   T
  0.4982494330086961  0.0988523740576743  0.4976161639905015   T   T   T
  0.6433979213918398  0.0998588974935607  0.4974122612758550   T   T   T
  0.7881226429381910  0.0987969785584226  0.4971849568130399   T   T   T
  0.9299495086008311  0.0993260279665148  0.4975854228872750   T   T   T
  0.0721868491445331  0.3499254014220503  0.4995679048010180   T   T   T
  0.2141088541272368  0.3498411391605148  0.4996887047087447   T   T   T
  0.3555377349702311  0.3490379022882686  0.4997283416236877   T   T   T
  0.4959851681920217  0.3477779689972073  0.4976188236494873   T   T   T
  0.6434913756445175  0.3451682277203795  0.4926978278540133   T   T   T
  0.7903019526683859  0.3479340206493324  0.4971352866369693   T   T   T
  0.9308762275350357  0.3491903982288468  0.4992859652988774   T   T   T
  0.0715902030809032  0.5998768650904922  0.4990541256832816   T   T   T
  0.2148198204246444  0.5998200375618016  0.4990095696751281   T   T   T
  0.3571796851519978  0.5996454040027401  0.4991266608882102   T   T   T
  0.4993854295483398  0.6005128298375186  0.4990961136557437   T   T   T
  0.6432489683657133  0.6021469338541000  0.4966829409522457   T   T   T
  0.7868701754471241  0.6007727339023867  0.4987412681322843   T   T   T
  0.9290836673130748  0.5999368273533036  0.4989090701236515   T   T   T
  0.0719599238477703  0.8495313114566244  0.4973585295110312   T   T   T
  0.2147340790700460  0.8495144824168120  0.4975704118865460   T   T   T
  0.3568048399741090  0.8495036967060180  0.4976568110585552   T   T   T
  0.4991010928992889  0.8498633897687554  0.4973414845675577   T   T   T
  0.6430600265100145  0.8502683174641620  0.4966016049838132   T   T   T
  0.7873060176891701  0.8499408890080895  0.4967127981517132   T   T   T
  0.9296863449310111  0.8497133605723475  0.4971078317919051   T   T   T
  0.0007654637492236  0.0101993871085025  0.5036544869811455   T   T   T
  0.1431749438231033  0.0102394931718028  0.5039367001433026   T   T   T
  0.2855898611772443  0.0101146999651835  0.5041177780669447   T   T   T
  0.4276384393071656  0.0100268918716080  0.5040850124112897   T   T   T
```

0.5708319842089453	0.0109465136621143	0.5040613475490919	T	T	T
0.7155358264621166	0.0108792089962865	0.5037568504828090	T	T	T
0.8585945235367332	0.0100833109953363	0.5035639911720891	T	T	T
0.0011537468185044	0.2600479735263786	0.5049006787459990	T	T	T
0.1431737946213403	0.2603710792144291	0.5052440234649452	T	T	T
0.2853291631340759	0.2599424834546745	0.5054233540111570	T	T	T
0.4275976628512939	0.2596258786737285	0.5053747884025768	T	T	T
0.5664458144029195	0.2595186765423576	0.5033809561057228	T	T	T
0.7201974233230691	0.2595967425156224	0.5031508047641288	T	T	T
0.8587919646914931	0.2596889827159923	0.5048176000223195	T	T	T
0.0003482327627721	0.5109096434088423	0.5059288584765232	T	T	T
0.1431789241498986	0.5108972258950564	0.5059630773414582	T	T	T
0.2858782660560690	0.5106980847936424	0.5060125167939874	T	T	T
0.4284680950718596	0.5106174214358670	0.5060943987529883	T	T	T
0.5707915489679642	0.5130113701808536	0.5035316716011363	T	T	T
0.7155919787288155	0.5131459029489884	0.5033234297315289	T	T	T
0.8577182292967332	0.5109658261201467	0.5056142134149310	T	T	T
0.0006171101467472	0.7605965271978539	0.5041131666997463	T	T	T
0.1434423477282439	0.7605603865384396	0.5042799841786264	T	T	T
0.2860041219907576	0.7604063334818290	0.5043264278758393	T	T	T
0.4279087173615673	0.7606854190465834	0.5043020081287186	T	T	T
0.5709819034667369	0.7615794922258196	0.5038712484537589	T	T	T
0.7153279635640495	0.7617838820447289	0.5037556460134771	T	T	T
0.8585593997986091	0.7609987597479314	0.5038659297703827	T	T	T
0.1434082612312713	0.0760983380948883	0.4325075725242803	T	T	T
0.2855342771628426	0.0758651391583409	0.4324993574633245	T	T	T
0.4273560770744596	0.0753846281013503	0.4322564152213310	T	T	T
0.5711741520478136	0.0765800575973700	0.4325632817254104	T	T	T
0.7151724383600123	0.0764901006294192	0.4322064861106014	T	T	T
0.8590194966114101	0.0753347297546714	0.4317600907707068	T	T	T
0.0011671094123836	0.0759629316920115	0.4320377628667492	T	T	T
0.1432310563514765	0.3262322354629719	0.4339168996767743	T	T	T
0.2847439783958456	0.3254684480636920	0.4339758340240831	T	T	T
0.4253056213588311	0.3246199723077805	0.4329463375728762	T	T	T
0.5677021122021838	0.3235204935683131	0.4310857256755736	T	T	T
0.7187142872773546	0.3234678940465774	0.4306474759322764	T	T	T
0.8611034931726662	0.3248361853915471	0.4325217431356026	T	T	T
0.0017779885686814	0.3259015318222706	0.4335802184590044	T	T	T
0.1432186311483221	0.5755052443857210	0.4338381172903275	T	T	T
0.2859654721062354	0.5752034363419369	0.4337039721657167	T	T	T
0.4282693912766480	0.5753529169217902	0.4337932631704030	T	T	T
0.5704938222589453	0.5775861051530199	0.4323577869614860	T	T	T
0.7158548041851603	0.5777030828887514	0.4320921129109924	T	T	T
0.8580978236991206	0.5760091107217384	0.4334024006826959	T	T	T
0.0004771889771010	0.5754899735608420	0.4336576309117925	T	T	T
0.1434924794367244	0.8254068976058286	0.4321791581516648	T	T	T
0.2858159300013126	0.8252271166055951	0.4321346072687267	T	T	T
0.4281030598766608	0.8255625327847009	0.4321327598463453	T	T	T
0.5709401399603971	0.8262753086897741	0.4318552664972587	T	T	T
0.7150484332281056	0.8262440806801618	0.4315780608337251	T	T	T
0.8585040283132377	0.8256804224096944	0.4314839385035780	T	T	T
0.0009378597592653	0.8253324926442809	0.4318229348558916	T	T	T
0.0722960491253574	0.1981738619956137	0.4531484952579542	T	T	T
0.2142903264631617	0.1980727611590387	0.4534270553993363	T	T	T

0.3564155121411375	0.1977276052384505	0.4535622673752274	T	T	T
0.4974654769811024	0.1970701883190764	0.4522024937401299	T	T	T
0.6432877178489069	0.1992767342490849	0.4535182526852177	T	T	T
0.7887669320049414	0.1971455515694487	0.4517343149088131	T	T	T
0.9300777616095099	0.1979029595314472	0.4530060897501804	T	T	T
0.0718417350043935	0.4482749662184382	0.4547504200704414	T	T	T
0.2143461345261058	0.4482175379924773	0.4547181484752143	T	T	T
0.3568284938243073	0.4478807639862570	0.4549794306805111	T	T	T
0.4979790005995750	0.4488793124567060	0.4532799987641785	T	T	T
0.6431505485623723	0.4505948475983612	0.4501823990806218	T	T	T
0.7881897257045853	0.4492056352630938	0.4528546296664492	T	T	T
0.9294186610684118	0.4481900877627510	0.4547109530903559	T	T	T
0.0719372520854824	0.6978381423585885	0.4533024814956274	T	T	T
0.2147915741244809	0.6977990534489346	0.4531679855956616	T	T	T
0.3571376414219974	0.6976107948395555	0.4532192429970044	T	T	T
0.4994699080547170	0.6984509428299095	0.4531542266680119	T	T	T
0.6431741106889862	0.7003222208030087	0.4523084591290778	T	T	T
0.7868082322553706	0.6989149580504812	0.4528660656233089	T	T	T
0.9294318537411962	0.6979611320697815	0.4529468863685716	T	T	T
0.0720509651671015	0.9479000971321349	0.4522358785386544	T	T	T
0.2145988302892591	0.9479655521040634	0.4524436467045320	T	T	T
0.3567927205879936	0.9477811856933620	0.4524618791845416	T	T	T
0.4996172323203869	0.9485554224527653	0.4525611943940656	T	T	T
0.6430346049383673	0.9494278177531899	0.4524107925756275	T	T	T
0.7866007453559758	0.9485767252997750	0.4520954058948094	T	T	T
0.9295871364026155	0.9480322846326019	0.4518287469015239	T	T	T
0.0007719492338475	0.1614431477176835	0.5494802082609278	T	T	T
0.1431665523987807	0.1615057870114476	0.5496390957723083	T	T	T
0.2856863532618662	0.1612948088796968	0.5499722460846460	T	T	T
0.4282711631614252	0.1610518584447352	0.5499387691919472	T	T	T
0.5707270223911556	0.1614254834101739	0.5483497779285621	T	T	T
0.7161509409083264	0.1615089287109001	0.5482312231998712	T	T	T
0.8582967494920063	0.1610511394412782	0.5493241678827253	T	T	T
0.0007199727999929	0.4120664144831685	0.5512362030350428	T	T	T
0.1432398410800565	0.4122795059672136	0.5511318423958885	T	T	T
0.2855296738869783	0.4119899594905450	0.5513777785856572	T	T	T
0.4269340693916820	0.4121822115573904	0.5518648007436249	T	T	T
0.5740081615887919	0.4124681610114873	0.5383550065756008	T	T	T
0.7126508527412067	0.4127003551781421	0.5383052135848575	T	T	T
0.8593305398249809	0.4124556271958225	0.5511519889079551	T	T	T
0.0002420897337317	0.6626227198291923	0.5499246622883487	T	T	T
0.1432822598211555	0.6624524997438135	0.5497701726002473	T	T	T
0.2860595166045594	0.6625143742998510	0.5500508221430397	T	T	T
0.4283591722724578	0.6627816476458436	0.5502344480721979	T	T	T
0.5716310275286979	0.6632914865532784	0.5487966837397542	T	T	T
0.7149122602192450	0.6634133739136997	0.5486113333823185	T	T	T
0.8578143200761277	0.6631280551784188	0.5498532244850080	T	T	T
0.0006563643031698	0.9118903420284200	0.5486582566243046	T	T	T
0.1432962472431877	0.9117624917763905	0.5487433309578195	T	T	T
0.2858639315098941	0.9117778207993692	0.5491245104948406	T	T	T
0.4282597068201908	0.9117961431927135	0.5491229797031282	T	T	T
0.5714147703448982	0.9120139045644235	0.5481604689451733	T	T	T
0.7150333443931720	0.9119695871193642	0.5477699943989047	T	T	T
0.8581951141160438	0.9118884190700678	0.5484619222490631	T	T	T

```
  0.0717935958418879  0.0336690169009754  0.5694017983135475   T   T   T
  0.2141759803060836  0.0336332216745398  0.5696545910275957   T   T   T
  0.3566543089135659  0.0334864192747862  0.5697679816321621   T   T   T
  0.4994359113614417  0.0338661087917752  0.5695275340555840   T   T   T
  0.6433712908920017  0.0342423011311141  0.5689095783098408   T   T   T
  0.7870075857812784  0.0337211369031022  0.5691559039738348   T   T   T
  0.9294465506270529  0.0337890846935547  0.5692419146770975   T   T   T
  0.0719362895002116  0.2836070208514395  0.5708422288269901   T   T   T
  0.2141018147016673  0.2836301174736081  0.5710246383597744   T   T   T
  0.3564519656684142  0.2832922633165159  0.5711698061717206   T   T   T
  0.4995130604868915  0.2825803272672588  0.5717209683102840   T   T   T
  0.6434189393602188  0.2832472795090982  0.5670393347071279   T   T   T
  0.7872333775271146  0.2829420041634830  0.5713328380872496   T   T   T
  0.9299571697599782  0.2832745815194257  0.5705632258890556   T   T   T
  0.0717148162949000  0.5347891030743027  0.5712257927174209   T   T   T
  0.2145167516658559  0.5349622675019324  0.5712194146456709   T   T   T
  0.3570246627572211  0.5352371275093145  0.5714350482484826   T   T   T
  0.4996104040969316  0.5347328737417671  0.5722099084951583   T   T   T
  0.6432200734956922  0.5422046038634987  0.5709125337407362   T   T   T
  0.7867799032604471  0.5350170552270248  0.5718949173162172   T   T   T
  0.9289287422452619  0.5353781516714103  0.5711735473280971   T   T   T
  0.0719674800945670  0.7848179844046325  0.5694743832959691   T   T   T
  0.2147154642806640  0.7846532882866724  0.5696469302043622   T   T   T
  0.3569916216413772  0.7848560747280346  0.5698444251383218   T   T   T
  0.4997814729199903  0.7850169817502539  0.5693757882902140   T   T   T
  0.6432231094341624  0.7859810391097973  0.5687664173034080   T   T   T
  0.7867959064260692  0.7857077751928855  0.5690228372605522   T   T   T
  0.9294685579609540  0.7852590667600399  0.5694143704079571   T   T   T
  0.5120255363971856  0.3824092985315910  0.5992794030966399   T   T   T
  0.5780751362320985  0.4734806779673231  0.6073586436475031   T   T   T
  0.6434853759874111  0.3824023702742624  0.5994655760826568   T   T   T
  0.7085635076204999  0.4736980376298083  0.6073648570321072   T   T   T
  0.7747400083967162  0.3827051907909725  0.5991447226074080   T   T   T
== On-top cluster Pd01-Te02
   1.00000000000000
    24.6586885454000111    0.0000000000000000    0.0000000000000000
     0.0000000000000000   25.2576961516000082    0.0000000000000000
     0.0000000000000000    0.0000000000000000   22.0000000000007034
   W    Te   Pd
    56   114     1
Selective dynamics
Direct
  0.0706931384524465  0.0991043878843524  0.4948510726810304   T   T   T
  0.2124958251893916  0.0988699385714093  0.4946211206708450   T   T   T
  0.3553578184582006  0.0989370539337539  0.4945907481730998   T   T   T
  0.4994022814416290  0.0987916445063719  0.4949948802705849   T   T   T
  0.6440371449690698  0.0991180898070845  0.4947917139691145   T   T   T
  0.7874757304103127  0.0989803969472980  0.4947764494124560   T   T   T
  0.9292921989815911  0.0990889552354319  0.4948477318088151   T   T   T
  0.0708034695194721  0.3492578724988184  0.4955841916058901   T   T   T
  0.2116832067894649  0.3490450235540959  0.4954748351836611   T   T   T
  0.3531594115451721  0.3478555067374744  0.4960215486277196   T   T   T
  0.4989999563101878  0.3478940154880441  0.4996746533478745   T   T   T
  0.6464168599214392  0.3478091063426864  0.4951208457642753   T   T   T
```

0.7880506270964680	0.3489222312692885	0.4951028245411209	T	T	T
0.9294272806510619	0.3492000673187928	0.4953474458857137	T	T	T
0.0707967751692181	0.5992519424395356	0.4958179479826990	T	T	T
0.2130537590928843	0.5994007444991903	0.4957819569389718	T	T	T
0.3557060804035948	0.5997129068977016	0.4947867437842302	T	T	T
0.4983787099120424	0.6007182284935931	0.4940230295841949	T	T	T
0.6446214719188417	0.6004277675754213	0.4946931635347572	T	T	T
0.7872866802808404	0.5995474135441909	0.4952980757584279	T	T	T
0.9288835338967540	0.5992240939872381	0.4955995485544707	T	T	T
0.0708333438027157	0.8490958914856446	0.4955907626834954	T	T	T
0.2128924241971415	0.8491974542274199	0.4951930099231263	T	T	T
0.3555917753062516	0.8495529687403118	0.4944306136171399	T	T	T
0.4995096162896597	0.8496819590627697	0.4938061600088165	T	T	T
0.6438264558581779	0.8495524730034286	0.4940156041204239	T	T	T
0.7872362718038023	0.8494168600408606	0.4950460519472500	T	T	T
0.9290379910378913	0.8491676332073630	0.4955622109683954	T	T	T
0.9999809687982938	0.0098130485449672	0.5035735491806870	T	T	T
0.1415942578013210	0.0098188171718542	0.5036261379897072	T	T	T
0.2838209843902327	0.0100280735101497	0.5036392222008431	T	T	T
0.4275015013185912	0.0102132316477175	0.5038141561763396	T	T	T
0.5715782120513633	0.0104232100060030	0.5040115792885825	T	T	T
0.7158262651887164	0.0102573412052888	0.5039179956922786	T	T	T
0.8584171795163517	0.0099156212042864	0.5037069913387731	T	T	T
0.0001868063003066	0.2598392712335207	0.5035079891081397	T	T	T
0.1414452017755755	0.2597324153767777	0.5035260584093815	T	T	T
0.2829074860575055	0.2593861142526612	0.5038678294195694	T	T	T
0.4292124533522258	0.2593550064182634	0.5051768489628464	T	T	T
0.5704338697976027	0.2597996061504212	0.5056487150232503	T	T	T
0.7165607701878475	0.2595896594377091	0.5039463863229349	T	T	T
0.8589122155247058	0.2597169815938557	0.5034009095930279	T	T	T
0.9999583594012315	0.5099357087834754	0.5042672145270516	T	T	T
0.1420347131516923	0.5100762008839773	0.5046993195870421	T	T	T
0.2837926135429330	0.5103598646437105	0.5050626817781501	T	T	T
0.4278452845053220	0.5117630262645818	0.5056680815105912	T	T	T
0.5716773493900120	0.5132675264321563	0.5057889961802554	T	T	T
0.7164936667650873	0.5107965619699789	0.5048257477998963	T	T	T
0.8581181352933233	0.5100209888438483	0.5042943890698974	T	T	T
0.9999128193981360	0.7599442851713607	0.5041064386144612	T	T	T
0.1420088807126979	0.7600213529632990	0.5041729950689651	T	T	T
0.2842012930908093	0.7603583641611357	0.5037214815510468	T	T	T
0.4272051391810100	0.7610750356794420	0.5031988936773524	T	T	T
0.5715921554536849	0.7611584709154531	0.5029437378786229	T	T	T
0.7159522735095517	0.7607442567386689	0.5035605613097108	T	T	T
0.8582369598464297	0.7601506360153497	0.5039988622052339	T	T	T
0.1415862703719787	0.0748403642916137	0.4053598811643446	T	T	T
0.2842322188554386	0.0750873927453245	0.4054454054516876	T	T	T
0.4277097834216678	0.0754312550796165	0.4059861481479340	T	T	T
0.5716760783225519	0.0756037842675151	0.4062899045937272	T	T	T
0.7153772597244493	0.0753189236318370	0.4058668251624649	T	T	T
0.8581739678583711	0.0748699927486661	0.4054128924340164	T	T	T
0.9999638842165991	0.0748972845371925	0.4053124002888974	T	T	T
0.1413574308035214	0.3252176238222108	0.4055955685099202	T	T	T
0.2833633683083304	0.3247308022443348	0.4059840676888669	T	T	T
0.4282289381785192	0.3257700379411488	0.4089886627848924	T	T	T

0.5706982248076395	0.3254045480675874	0.4090471967857325	T	T	T
0.7163537287003402	0.3242700586834458	0.4054671262068091	T	T	T
0.8586638896227449	0.3251383990388670	0.4055300737725127	T	T	T
0.0002240772187067	0.3253332707631953	0.4056893072690135	T	T	T
0.1419383795254571	0.5751748648040601	0.4066117459847274	T	T	T
0.2841330721737411	0.5748126422187330	0.4064503830596184	T	T	T
0.4273515146697482	0.5748963783334917	0.4058402009743996	T	T	T
0.5715754583298651	0.5762996308874394	0.4066267394257269	T	T	T
0.7156678132950245	0.5751387826578815	0.4062134038848224	T	T	T
0.8581082982508590	0.5752199309147681	0.4060178395150795	T	T	T
0.9999887886392136	0.5751914521238741	0.4062675028178340	T	T	T
0.1417485787599326	0.8251030738253505	0.4060505494017857	T	T	T
0.2841693212554118	0.8253976653279499	0.4056018862426638	T	T	T
0.4274766442628523	0.8262332567693619	0.4052199926590200	T	T	T
0.5717649318102410	0.8261296277698020	0.4050777553620365	T	T	T
0.7157083107812489	0.8257145343021340	0.4055130936744100	T	T	T
0.8581865316642319	0.8253467491145022	0.4058425036945630	T	T	T
0.9999658939006035	0.8252969765279741	0.4061428581870105	T	T	T
0.0708651720794000	0.1972978441601567	0.4328266431681718	T	T	T
0.2126108527738062	0.1973231730972912	0.4329841368686444	T	T	T
0.3562906997315133	0.1983628275552493	0.4347277889412487	T	T	T
0.4998985852341122	0.1977975178542757	0.4342996603128935	T	T	T
0.6430864237265185	0.1982790581398488	0.4347556466807519	T	T	T
0.7870931118208429	0.1975443093494161	0.4333708385508591	T	T	T
0.9292729985510068	0.1973091400502384	0.4328058067171807	T	T	T
0.0711955810759589	0.4475439478784060	0.4344199578916963	T	T	T
0.2130559006696205	0.4476856134241980	0.4346375840031228	T	T	T
0.3556074598014057	0.4482288182646483	0.4360716253118632	T	T	T
0.4997027809555781	0.4483994797476777	0.4390621621046473	T	T	T
0.6444143211712865	0.4493970766433800	0.4356155185124283	T	T	T
0.7869907381362715	0.4477366459655863	0.4342257001415108	T	T	T
0.9289565762587342	0.4474505158193550	0.4340291920676637	T	T	T
0.0710309234106910	0.6975272335339369	0.4341371664883119	T	T	T
0.2132469911659819	0.6976739582301347	0.4339693179296549	T	T	T
0.3558775188713076	0.6985499603931303	0.4333798514846638	T	T	T
0.4990911075426396	0.6996520422326877	0.4330303891268057	T	T	T
0.6437193805082305	0.6992281094645497	0.4333472129532951	T	T	T
0.7868068060880614	0.6981486287633262	0.4335613054608047	T	T	T
0.9287991174711145	0.6975254359373374	0.4338262027939304	T	T	T
0.0708802515039775	0.9471124910702872	0.4334225438598251	T	T	T
0.2131173659860567	0.9475093685451963	0.4335286873958624	T	T	T
0.3560202206703182	0.9483549619369003	0.4336657167398539	T	T	T
0.4996856299464000	0.9488835290691092	0.4337229585972047	T	T	T
0.6434120113095697	0.9488102669509983	0.4338010760481939	T	T	T
0.7865457886862447	0.9478733980234588	0.4337150635735413	T	T	T
0.9289333250713608	0.9471770533184789	0.4333949445338380	T	T	T
0.9999937080771139	0.1616090437833288	0.5651749415873445	T	T	T
0.1417817110995372	0.1614145648334059	0.5650285831486014	T	T	T
0.2839128072237928	0.1610151578916486	0.5648313876951412	T	T	T
0.4274583662351858	0.1599894462756526	0.5650220740920634	T	T	T
0.5716796548076045	0.1599335201085289	0.5651116212074545	T	T	T
0.7156555179378732	0.1609478148955155	0.5648938861415562	T	T	T
0.8582424764236876	0.1614191251070529	0.5650622616785299	T	T	T
0.9999672180287813	0.4116863765100336	0.5657708410959937	T	T	T

```
  0.1417261200449902  0.4115239261907394  0.5662317839042976   T   T   T
  0.2828038204509998  0.4108569479928288  0.5662026762091008   T   T   T
  0.4233385790583576  0.4122205639606906  0.5636955253066325   T   T   T
  0.5765694978329429  0.4135096083310195  0.5562507293842920   T   T   T
  0.7168950868893964  0.4111038868279621  0.5652510027035527   T   T   T
  0.8583153639509820  0.4113559388220994  0.5658834510120603   T   T   T
  0.9997740594290855  0.6617121453884187  0.5657181574550461   T   T   T
  0.1421520450065846  0.6617660400890477  0.5658154968243524   T   T   T
  0.2849726106795659  0.6619420832625373  0.5651027699811535   T   T   T
  0.4276409832295007  0.6625196689545939  0.5642434192877388   T   T   T
  0.5712720782052144  0.6624452430559330  0.5629359576882791   T   T   T
  0.7154978300544219  0.6623022133555434  0.5646904048835516   T   T   T
  0.8578027301213291  0.6619386143933625  0.5657368595065657   T   T   T
  0.9998876630544367  0.9117138688932753  0.5655678933148404   T   T   T
  0.1421808722423889  0.9115685739424662  0.5655161256586309   T   T   T
  0.2846351204028148  0.9115916630250737  0.5650373033976311   T   T   T
  0.4279116293285663  0.9112294106223784  0.5642330927872862   T   T   T
  0.5715596183011279  0.9112382145790839  0.5638878905617295   T   T   T
  0.7149227334207585  0.9113994772623802  0.5646982319137831   T   T   T
  0.8577524396834464  0.9116877946593139  0.5656032452824526   T   T   T
  0.0708610922438484  0.0340429181340134  0.5930987505788061   T   T   T
  0.2128761610256208  0.0340345972055607  0.5929117343801035   T   T   T
  0.3557633273603847  0.0339965804837503  0.5925236261663852   T   T   T
  0.4994551475143740  0.0336341129510878  0.5928007980214767   T   T   T
  0.6435218247573359  0.0340951217422444  0.5926127342508261   T   T   T
  0.7869997183770435  0.0341400694658168  0.5929930670190314   T   T   T
  0.9291481567686609  0.0340576880480364  0.5931325866697400   T   T   T
  0.0708815706472399  0.2835046540680816  0.5933178768583107   T   T   T
  0.2124510710654303  0.2836490970484562  0.5933003404173294   T   T   T
  0.3553177732312926  0.2826863057152555  0.5928721313114226   T   T   T
  0.4992797044732289  0.2781219052901402  0.5965249463359078   T   T   T
  0.6441061499321823  0.2832377231287763  0.5929240137943409   T   T   T
  0.7877166219735683  0.2836933763757669  0.5929177105856585   T   T   T
  0.9292897036331309  0.2836468352491042  0.5931577873953673   T   T   T
  0.0708093792749773  0.5340560786617448  0.5938613127021963   T   T   T
  0.2128690525339413  0.5344842260465039  0.5942440815156059   T   T   T
  0.3555478823302913  0.5362342330777468  0.5937871997717150   T   T   T
  0.4993183999540148  0.5384663744414587  0.5939485342660588   T   T   T
  0.6444054419450067  0.5369825189801992  0.5934433839597020   T   T   T
  0.7873421612615473  0.5349132442834513  0.5939605429382528   T   T   T
  0.9290564045209744  0.5339945027881644  0.5937255625760586   T   T   T
  0.0710598564752309  0.7837226435335515  0.5936093065029446   T   T   T
  0.2134599930349504  0.7840300746073011  0.5933450614004580   T   T   T
  0.3560536530127872  0.7847055221591761  0.5925395714963827   T   T   T
  0.4995546025032532  0.7847602400077038  0.5917197308403170   T   T   T
  0.6433694951662144  0.7846256757028968  0.5919726558419702   T   T   T
  0.7866324538976422  0.7843639737671303  0.5931640085426988   T   T   T
  0.9289575938600483  0.7838257698945484  0.5936675106913171   T   T   T
  0.5926513281282501  0.3941408301035907  0.6901071756069873   T   T   T
  0.5003934675475266  0.4416498968342097  0.7291757430432007   T   T   T
  0.5011720759213607  0.3795570269877085  0.6323426680087976   T   T   T
== On-top cluster Pd01-Te03
   1.00000000000000
    24.6586885454000111    0.0000000000000000    0.0000000000000000
```

```
     0.0000000000000000   25.2576961516000082    0.0000000000000000
     0.0000000000000000    0.0000000000000000   30.0000000000007034
   W    Te   Pd
    56   115     1
Selective dynamics
Direct
  0.0706586210737328  0.0991495964507983  0.4962565846290212   T   T   T
  0.2124328718075578  0.0988503932184939  0.4965857779872484   T   T   T
  0.3557304194113187  0.0987110970119549  0.4968746829147960   T   T   T
  0.4999249815196278  0.0984288823574618  0.4971965053596003   T   T   T
  0.6439346915208459  0.0987622930248970  0.4967305012846007   T   T   T
  0.7871426198577096  0.0989091445736142  0.4962696194288265   T   T   T
  0.9291990252599409  0.0991389455121562  0.4961628432906600   T   T   T
  0.0706244573821945  0.3493150906473513  0.4975541570987118   T   T   T
  0.2113982633897945  0.3488456116131154  0.4978500038220975   T   T   T
  0.3531453720557817  0.3477147232168980  0.4986988614344142   T   T   T
  0.4997794005811532  0.3479866513541007  0.5016590663596044   T   T   T
  0.6466370263859178  0.3477628689393985  0.4987293153597934   T   T   T
  0.7883489902008022  0.3488893461701162  0.4976262905816058   T   T   T
  0.9292690655748400  0.3493085645164828  0.4974522934424727   T   T   T
  0.0708016825006544  0.5991691816026530  0.4969120356421883   T   T   T
  0.2125869334568038  0.5993262987120389  0.4972221365550253   T   T   T
  0.3556278587612170  0.5997435458576899  0.4971859802147504   T   T   T
  0.4998667619667979  0.6002088449758484  0.4982867153023665   T   T   T
  0.6439491431839020  0.5997221700760105  0.4971831879665557   T   T   T
  0.7870815242449870  0.5993555895309839  0.4971265644280767   T   T   T
  0.9289832360662235  0.5991361785096495  0.4968482970947597   T   T   T
  0.0707094348501349  0.8491586251824252  0.4957440158941541   T   T   T
  0.2124914816606406  0.8492525463400383  0.4958865640046776   T   T   T
  0.3557474030240346  0.8492350471239829  0.4961144064250670   T   T   T
  0.4997948606991185  0.8492769750216318  0.4964312669859661   T   T   T
  0.6438557515564051  0.8492092405163372  0.4959330503864368   T   T   T
  0.7871785519207827  0.8492326575808639  0.4957116775642689   T   T   T
  0.9290763448523759  0.8491817112236296  0.4956680259657950   T   T   T
  0.9998709307396030  0.0098682383454826  0.5020854234014904   T   T   T
  0.1414752371086938  0.0098960188017584  0.5023031030825581   T   T   T
  0.2838813953896496  0.0099516968225226  0.5029632124090250   T   T   T
  0.4279091297805568  0.0099465064798115  0.5035380849678632   T   T   T
  0.5718592274915587  0.0099221404453248  0.5033999764699451   T   T   T
  0.7156724983624505  0.0099650109627079  0.5026770208540663   T   T   T
  0.8582080247083631  0.0099453856952893  0.5021738858830761   T   T   T
  0.9999761632753310  0.2599541689493517  0.5030918079019577   T   T   T
  0.1412191294494028  0.2597117426355009  0.5035795667628465   T   T   T
  0.2833659087043947  0.2592636915833952  0.5043304027910540   T   T   T
  0.4296349446839202  0.2591284991541083  0.5055805751166631   T   T   T
  0.5700945696427004  0.2591858765071937  0.5056603910090042   T   T   T
  0.7162935454248646  0.2593266304051154  0.5041986249010174   T   T   T
  0.8586081909654365  0.2597109718478841  0.5032767271781088   T   T   T
  0.9999405295173723  0.5099149166826933  0.5035815624518454   T   T   T
  0.1418066641854597  0.5100180072858782  0.5039848316926073   T   T   T
  0.2834950641725871  0.5105056865028990  0.5046980430143424   T   T   T
  0.4287770940614909  0.5120372623070902  0.5053500134806819   T   T   T
  0.5708027550123425  0.5119922649326794  0.5052914836132693   T   T   T
  0.7161715550838496  0.5105140896871037  0.5046387257152337   T   T   T
```

0.8580006035807999	0.5100261420331421	0.5039000712545598	T	T	T
0.9999029894449065	0.7598251999181009	0.5021486397462817	T	T	T
0.1415824958748820	0.7600384097839120	0.5023157929050448	T	T	T
0.2838437803226378	0.7603731747824126	0.5028168958905530	T	T	T
0.4277794961983626	0.7607568377631571	0.5032824098791047	T	T	T
0.5717441506089080	0.7607391309077225	0.5033688336461816	T	T	T
0.7157336874712042	0.7604375652636327	0.5027817970812400	T	T	T
0.8581962322942687	0.7600645922409037	0.5022611819812859	T	T	T
0.1419722068295517	0.0756997237373768	0.4306574687762242	T	T	T
0.2845353953159571	0.0755430548700377	0.4312923467718265	T	T	T
0.4280092462521577	0.0754150913807368	0.4319709358188221	T	T	T
0.5716482229853757	0.0754186418918546	0.4318632716443288	T	T	T
0.7150307327999357	0.0756010152857615	0.4310471135559554	T	T	T
0.8578710631264712	0.0757078368299544	0.4305366339394151	T	T	T
0.9999845373358083	0.0756076787404270	0.4304187692929168	T	T	T
0.1413953450778523	0.3252621512972051	0.4317944098956050	T	T	T
0.2837045793658982	0.3245038224011038	0.4325904411120403	T	T	T
0.4286324061423701	0.3254105476472291	0.4352300660395061	T	T	T
0.5711034324339700	0.3254687734468312	0.4353334575711319	T	T	T
0.7158732687369032	0.3246898112854269	0.4324557700433938	T	T	T
0.8584555793898513	0.3254066954628974	0.4315946111551338	T	T	T
0.9999930580111007	0.3258414726021929	0.4315601516453016	T	T	T
0.1420672846462344	0.5746537579008240	0.4316535041740202	T	T	T
0.2844008705866941	0.5747334643917300	0.4322144266834297	T	T	T
0.4284046876243178	0.5757659020266466	0.4328801419246719	T	T	T
0.5712294922589426	0.5757399073323975	0.4328143568270229	T	T	T
0.7151265762583041	0.5747285020162748	0.4321760947953684	T	T	T
0.8576343519536646	0.5747055019054603	0.4315766660721425	T	T	T
0.9999039841167022	0.5747489724951834	0.4313939881582818	T	T	T
0.1419177967937986	0.8252038927777504	0.4301989358390630	T	T	T
0.2845481281341361	0.8251093795062946	0.4307552077419641	T	T	T
0.4280781358609240	0.8255499616132641	0.4312149092057316	T	T	T
0.5714220470531837	0.8252904268542923	0.4312182906886719	T	T	T
0.7151263858280098	0.8250005842763198	0.4306231228232534	T	T	T
0.8578696262301085	0.8251016659923405	0.4301237018742276	T	T	T
0.9999373179981813	0.8249440578797360	0.4301628928011089	T	T	T
0.0709317063341678	0.1976399421979193	0.4512412620291213	T	T	T
0.2129120060794143	0.1977036804086706	0.4520111303377125	T	T	T
0.3567954515488617	0.1983811573236665	0.4533949473359651	T	T	T
0.4999665601727092	0.1976822943196853	0.4529842187051813	T	T	T
0.6428871765964458	0.1984727639995177	0.4533624350449886	T	T	T
0.7867318436713822	0.1978398694852370	0.4517055720381865	T	T	T
0.9290387018699845	0.1976436128174144	0.4511213451496047	T	T	T
0.0711320544680509	0.4474160092844344	0.4524473120997802	T	T	T
0.2129871512754157	0.4474406109471420	0.4529690576533005	T	T	T
0.3557337503612430	0.4482997429107990	0.4546785525318394	T	T	T
0.4997497275273596	0.4472354242031477	0.4560824632301436	T	T	T
0.6438783999541244	0.4483364246095992	0.4546437334624124	T	T	T
0.7867670402513982	0.4475467453728260	0.4528338636371691	T	T	T
0.9287582976009406	0.4474062622789556	0.4524065731845924	T	T	T
0.0711325897826044	0.6970899877812252	0.4509906809488668	T	T	T
0.2133337917434993	0.6975715832053607	0.4513306742247779	T	T	T
0.3562402871620353	0.6984607666893380	0.4520294867627053	T	T	T
0.4998076464296689	0.6986001901436530	0.4525161553887281	T	T	T

0.6432740683172412	0.6984769303295413	0.4520881203508134	T	T	T
0.7863497234027903	0.6976633699497102	0.4513147154557716	T	T	T
0.9287212187486259	0.6970730041112300	0.4509489506678096	T	T	T
0.0709702451402224	0.9475771988946217	0.4504418653863160	T	T	T
0.2132550966826485	0.9478370692058131	0.4509444266735233	T	T	T
0.3563349327853689	0.9482588313054700	0.4517586672426354	T	T	T
0.4997964223330244	0.9483468159559083	0.4521244105602439	T	T	T
0.6432735192127034	0.9482494348923853	0.4514642208371454	T	T	T
0.7863635696416619	0.9479600688244170	0.4507587750974534	T	T	T
0.9287429178466877	0.9476372118365483	0.4503870765873057	T	T	T
0.9998652853315028	0.1614349682632860	0.5478824174747999	T	T	T
0.1416175933473086	0.1611022830985575	0.5483211867316161	T	T	T
0.2840463725913390	0.1604389275807777	0.5483637395996724	T	T	T
0.4278845177492565	0.1595450058071137	0.5489615895962991	T	T	T
0.5719483294203973	0.1595275403981549	0.5489953988107490	T	T	T
0.7156208176164948	0.1604868384203759	0.5482291941679759	T	T	T
0.8580108989737907	0.1610842645870654	0.5479432646611736	T	T	T
0.9999324297677612	0.4118425270717661	0.5490756753940302	T	T	T
0.1414220216570572	0.4116340699121524	0.5495192352053102	T	T	T
0.2824202515539868	0.4108644702403839	0.5497287263575811	T	T	T
0.4235738931679768	0.4131210804105329	0.5461568415264431	T	T	T
0.5760623753500819	0.4130690342334818	0.5461350143535719	T	T	T
0.7173540833378990	0.4109012620616843	0.5496207661224717	T	T	T
0.8583990318013637	0.4115774745989221	0.5493867051639619	T	T	T
0.9997769375960424	0.6620832612681011	0.5479108907004349	T	T	T
0.1417616911292030	0.6621410738337631	0.5481516895380760	T	T	T
0.2842998677140375	0.6621415706845750	0.5479246708617781	T	T	T
0.4276960842438713	0.6624068018670820	0.5482082179115578	T	T	T
0.5720204854717202	0.6623695357698547	0.5482850991376320	T	T	T
0.7153983886594408	0.6622014792996520	0.5478378880034630	T	T	T
0.8579179336820647	0.6621789365561044	0.5480163592151961	T	T	T
0.9998553384833462	0.9115816249117908	0.5472266670136634	T	T	T
0.1417287446738525	0.9115881969197336	0.5472925497409111	T	T	T
0.2843892895142952	0.9111410559985079	0.5474346655342270	T	T	T
0.4278093214461952	0.9108110490718713	0.5476423338748436	T	T	T
0.5719964342466319	0.9108331864316733	0.5475415298487533	T	T	T
0.7152893518874484	0.9111317515599259	0.5471229868792453	T	T	T
0.8578490078876700	0.9115809335741701	0.5471134154457017	T	T	T
0.0706538915243690	0.0334567039109969	0.5679469275809774	T	T	T
0.2125959282651819	0.0333525788337977	0.5683044838915572	T	T	T
0.3557751511745688	0.0333370417901034	0.5684412580422040	T	T	T
0.4999911998331559	0.0328660472280722	0.5686963977526551	T	T	T
0.6439592757935403	0.0333299425748504	0.5682444478364792	T	T	T
0.7869292828926979	0.0334852925470232	0.5679722125663484	T	T	T
0.9289666560985730	0.0334178945086499	0.5678673830038944	T	T	T
0.0703766846268387	0.2835403828615659	0.5691823399714786	T	T	T
0.2121618516727914	0.2834031346518457	0.5695980543038980	T	T	T
0.3555480031520826	0.2823093995273648	0.5698361832939587	T	T	T
0.4998145831784451	0.2775131496308501	0.5726887485673661	T	T	T
0.6442716794515841	0.2823341810106616	0.5698844722189431	T	T	T
0.7877869934141417	0.2834194677802180	0.5693033066586585	T	T	T
0.9293913452597278	0.2834369463329785	0.5690126129957199	T	T	T
0.0706626194455864	0.5345941434185113	0.5692457547988791	T	T	T
0.2124201571137105	0.5347578445007402	0.5698031068773852	T	T	T

```
  0.3552999642617134  0.5362500069883100  0.5695764103629324   T   T   T
  0.4998338797409485  0.5352027234643197  0.5710697755756088   T   T   T
  0.6442183360741182  0.5360424848811756  0.5695217173068048   T   T   T
  0.7873434788437506  0.5347739126655755  0.5696786186771557   T   T   T
  0.9291756926935615  0.5345479582626073  0.5691833100790583   T   T   T
  0.0707543915876706  0.7841427743213347  0.5677866972523373   T   T   T
  0.2126781996334092  0.7845207713470778  0.5679686114798896   T   T   T
  0.3557161103914429  0.7845886064253740  0.5681103866824885   T   T   T
  0.4996895073790251  0.7846693092744581  0.5684997632444757   T   T   T
  0.6438676806042261  0.7848583917121432  0.5680802594866234   T   T   T
  0.7869958234510855  0.7845800696238235  0.5678821819609743   T   T   T
  0.9290272025925590  0.7842527877471224  0.5677458642559595   T   T   T
  0.5749558604475935  0.4053375341097705  0.6564429412047483   T   T   T
  0.4246280068553620  0.4055045524625944  0.6562952989870718   T   T   T
  0.4997778779042447  0.4453431574457912  0.7145999103061571   T   T   T
  0.4998459420069746  0.3813744200127716  0.5998483179550528   T   T   T
== Te x4 on Pd(111)
   1.00000000000000
     16.1454760000000412    0.0000000000000066    0.0000000000006193
     -8.0727380000000792   13.9823920000000115    0.0000000000008174
      0.0000000000005753    0.0000000000009511   30.0000000000008242
   Pd   Te
   144     4
Selective dynamics
Direct
  0.0000000000009734  0.0000000000010019  0.0000050000008116   F   F   F
  0.1666666666666359  0.0000000000001350  0.0000050000002219   F   F   F
  0.3333333333333357  0.0000000000009379  0.0000050000000371   F   F   F
  0.5000000000004405  0.0000000000006679  0.0000050000009679   F   F   F
  0.6666666666666927  0.0000000000006395  0.0000050000001792   F   F   F
  0.8333333333332646  0.0000000000008455  0.0000050000009892   F   F   F
  0.0000000000007745  0.1666666666666288  0.0000050000009395   F   F   F
  0.1666666666666075  0.1666666666666075  0.0000050000005487   F   F   F
  0.3333333333331510  0.1666666666666075  0.0000050000009182   F   F   F
  0.5000000000001918  0.1666666666666146  0.0000050000005842   F   F   F
  0.6666666666666927  0.1666666666666501  0.0000050000007121   F   F   F
  0.8333333333339823  0.1666666666666785  0.0000050000005700   F   F   F
  0.0000000000004974  0.3333333333330586  0.0000050000006198   F   F   F
  0.1666666666665719  0.3333333333336697  0.0000050000009537   F   F   F
  0.3333333333335275  0.3333333333332362  0.0000050000003569   F   F   F
  0.5000000000004903  0.3333333333331439  0.0000050000000869   F   F   F
  0.6666666666661598  0.3333333333331439  0.0000050000008045   F   F   F
  0.8333333333331439  0.3333333333338899  0.0000050000002147   F   F   F
  0.0000000000009166  0.5000000000006466  0.0000050000005345   F   F   F
  0.1666666666661314  0.5000000000009308  0.0000050000005132   F   F   F
  0.3333333333338118  0.5000000000004476  0.0000050000008329   F   F   F
  0.5000000000008029  0.5000000000009592  0.0000050000007121   F   F   F
  0.6666666666661030  0.5000000000001350  0.0000050000002787   F   F   F
  0.8333333333333783  0.5000000000004903  0.0000050000003213   F   F   F
  0.0000000000004334  0.6666666666666430  0.0000050000006198   F   F   F
  0.1666666666668135  0.6666666666666501  0.0000050000006766   F   F   F
  0.3333333333338828  0.6666666666666430  0.0000050000001934   F   F   F
  0.5000000000009379  0.6666666666666359  0.0000050000000655   F   F   F
  0.6665283418613441  0.6666666666666146  0.0000050000001437   F   F   F
```

0.8333333333334281	0.6666666666666856	0.0000050000007121	F	F	F
0.1666666666666217	0.8333333333331865	0.0000050000003782	F	F	F
0.0000000000003268	0.8333333333332362	0.0000050000004848	F	F	F
0.3333333333334920	0.8333333333337052	0.0000050000005061	F	F	F
0.5000000000000142	0.8333333333339397	0.0000050000000371	F	F	F
0.6666666666667069	0.8333333333338615	0.0000050000004279	F	F	F
0.8333333333333286	0.8333333333332931	0.0000050000001011	F	F	F
0.1110073154275289	0.0554537864781905	0.0755690003810836	T	T	T
0.2777319744381870	0.0554449216937746	0.0755796120545230	T	T	T
0.4443938737213278	0.0554994244630979	0.0744099824184245	T	T	T
0.6110119920408633	0.0554585528120307	0.0755668351314740	T	T	T
0.7777339432152495	0.0554459053894830	0.0755802933972349	T	T	T
0.9443918074881325	0.0554966272184578	0.0744114918215555	T	T	T
0.1110154647231134	0.2221588792704743	0.0755792288632062	T	T	T
0.2777411519303696	0.2221678306680346	0.0761332855836343	T	T	T
0.4444591002548946	0.2221652345309439	0.0755770281950662	T	T	T
0.6110178789904276	0.2221647943419693	0.0755772762459453	T	T	T
0.7777427598529648	0.2221697492914675	0.0761338985901672	T	T	T
0.9444591077315269	0.2221647367436186	0.0755782575140928	T	T	T
0.1110711706021730	0.3888443913097283	0.0757928473933081	T	T	T
0.2777307868375233	0.3888755762327559	0.0755748765084707	T	T	T
0.4444535998409207	0.3888946676456955	0.0755608729448781	T	T	T
0.6110711056190337	0.3888462374739815	0.0757931562334397	T	T	T
0.7777329583471734	0.3888793344830695	0.0755750279865703	T	T	T
0.9444527290331467	0.3888933678418716	0.0755619537517626	T	T	T
0.1110097692789501	0.5554564617140721	0.0755662287259765	T	T	T
0.2777320958348947	0.5554448876033579	0.0755793042705747	T	T	T
0.4443887343877735	0.5554971012138502	0.0744114623414903	T	T	T
0.6110083991009632	0.5554579527014577	0.0755674640689450	T	T	T
0.7777295311367675	0.5554456404149831	0.0755785747134318	T	T	T
0.9443905197443365	0.5554964777200502	0.0744102320228192	T	T	T
0.1110148178379854	0.7221630907164781	0.0755768477511344	T	T	T
0.2777396451588973	0.7221709837528177	0.0761309554955288	T	T	T
0.4444549603831755	0.7221659047928162	0.0755777439196957	T	T	T
0.6110005999656406	0.7221610975203382	0.0755942506068307	T	T	T
0.7777285364374205	0.7221657896404930	0.0761237586973537	T	T	T
0.9444544028208479	0.7221633185354462	0.0755769101454477	T	T	T
0.1110691510646118	0.8888444842966218	0.0757929080539965	T	T	T
0.2777327834409668	0.8888810256716255	0.0755736145756806	T	T	T
0.4444534268510568	0.8888954291587167	0.0755612804819275	T	T	T
0.6110703800104045	0.8888479346175649	0.0757941733833705	T	T	T
0.7777264239493655	0.8888757344947572	0.0755758475712429	T	T	T
0.9444478457023766	0.8888899849687073	0.0755613259737984	T	T	T
0.0550297053820131	0.1107807501707846	0.1511860447265548	T	T	T
0.2219649838749907	0.1106023241203808	0.1515432536900029	T	T	T
0.3890807145482827	0.1107663685490526	0.1511749311562010	T	T	T
0.5550253773672269	0.1107776035409983	0.1511799940293374	T	T	T
0.7219665828636818	0.1106017656042135	0.1515423735211982	T	T	T
0.8890824867833441	0.1107677400761652	0.1511774036151062	T	T	T
0.0555346619889871	0.2777340147265594	0.1516104425417455	T	T	T
0.2219665070366125	0.2778931136014921	0.1515559603457091	T	T	T
0.3892596150191424	0.2778932984028627	0.1515412515939905	T	T	T
0.5555343921420860	0.2777337518365511	0.1516099933874321	T	T	T
0.7219670728423779	0.2778952162861112	0.1515550071195504	T	T	T

0.8892578876762659	0.2778928589915953	0.1515443860642612	T	T	T
0.0555193387263627	0.4443311732650829	0.1515959290564292	T	T	T
0.2221249395359406	0.4443214304772731	0.1516101956202458	T	T	T
0.3890690449120098	0.4448134730796358	0.1511783709527150	T	T	T
0.5555192036119407	0.4443316552046073	0.1515965207111010	T	T	T
0.7221242632799095	0.4443220819733660	0.1516105115367755	T	T	T
0.8890670083494930	0.4448111896574853	0.1511803818997819	T	T	T
0.0550248608127248	0.6107764934954152	0.1511795686174399	T	T	T
0.2219682104391025	0.6106049204255224	0.1515394379936464	T	T	T
0.3890830691221525	0.6107687942317073	0.1511758666815298	T	T	T
0.5550272468380912	0.6107783206795881	0.1511871715545077	T	T	T
0.7219677686206819	0.6106067406376288	0.1515400642307069	T	T	T
0.8890798243255741	0.6107655947469620	0.1511750750711965	T	T	T
0.0555326172702162	0.7777323553580480	0.1516089110144823	T	T	T
0.2219657798624610	0.7778957068932086	0.1515526899951541	T	T	T
0.3892581423420223	0.7778944684855059	0.1515420047875047	T	T	T
0.5555338760731082	0.7777357668061559	0.1516131038987215	T	T	T
0.7219697509252425	0.7778950398181278	0.1515571182078683	T	T	T
0.8892572289961004	0.7778906961152763	0.1515407679089859	T	T	T
0.0555173341773515	0.9443298492129351	0.1515970563705510	T	T	T
0.2221226838862854	0.9443219686979100	0.1516090676341317	T	T	T
0.3890664442625322	0.9448122258204104	0.1511787102469241	T	T	T
0.5555188943886372	0.9443322986094022	0.1515969826627097	T	T	T
0.7221249637861548	0.9443211593717792	0.1516122814091110	T	T	T
0.8890692998530123	0.9448136923850954	0.1511797102434642	T	T	T
0.9998319232278661	0.9999510122398155	0.2270381950950857	T	T	T
0.1660096048541213	0.9986553756810385	0.2279134604266793	T	T	T
0.3334718652959233	0.9999355663301305	0.2270287742960680	T	T	T
0.4998274351625809	0.9999471134201064	0.2270361162278638	T	T	T
0.6660094732226475	0.9986569606316242	0.2279150338889733	T	T	T
0.8334748468605059	0.9999383669998330	0.2270308225921697	T	T	T
0.9998710920157193	0.1663560477561341	0.2270354472920896	T	T	T
0.1620392321080029	0.1642306446522344	0.2276096773715422	T	T	T
0.3355945067787552	0.1642172237270621	0.2275651859579965	T	T	T
0.4998654534385788	0.1663536493338595	0.2270330602590538	T	T	T
0.6620262249807851	0.1642288116677701	0.2275934892819204	T	T	T
0.8355954937505056	0.1642203988300997	0.2275694263602578	T	T	T
0.0011562037843798	0.3338035784893756	0.2279025942378772	T	T	T
0.1660033905769463	0.3338099894659979	0.2279086230849323	T	T	T
0.3355423032836485	0.3377151993693896	0.2276342497851586	T	T	T
0.5011530390010775	0.3338035035615884	0.2279040594437324	T	T	T
0.6660002979606877	0.3338095283804069	0.2279094109223602	T	T	T
0.8355408804250399	0.3377096321238323	0.2276433855893940	T	T	T
0.9998273321274233	0.4999489674990098	0.2270365996044488	T	T	T
0.1660091548837633	0.4986586900071246	0.2279125970812634	T	T	T
0.3334740302829340	0.4999427280838951	0.2270286874574599	T	T	T
0.4998304870255545	0.4999517306036070	0.2270378063302561	T	T	T
0.6660084783615547	0.4986564429607473	0.2279127682272669	T	T	T
0.8334717006217267	0.4999383211855506	0.2270298635548018	T	T	T
0.9998672675372434	0.6663553371824462	0.2270326441414070	T	T	T
0.1620300048304352	0.6642317924691975	0.2275929591468657	T	T	T
0.3355968392967290	0.6642213039215019	0.2275653899492087	T	T	T
0.4998713182006853	0.6663562102484714	0.2270359835687985	T	T	T
0.6620345273995863	0.6642291394430989	0.2276039644655516	T	T	T

```
  0.8355968279708578  0.6642194818167106  0.2275656228755241   T   T   T
  0.0011579863740774  0.8338037874128765  0.2279033277518278   T   T   T
  0.1660029106771650  0.8338101548075089  0.2279077432263351   T   T   T
  0.3355433789214329  0.8377097319234532  0.2276385361656045   T   T   T
  0.5011589102162134  0.8338029465914447  0.2279035474100739   T   T   T
  0.6660049212841973  0.8338098491215794  0.2279108018535005   T   T   T
  0.8355468140116901  0.8377152475911633  0.2276385522681834   T   T   T
  0.2777266585280379  0.2218287527019987  0.2912630672503009   T   T   T
  0.7776376516076275  0.2217841875190866  0.2912578110132404   T   T   T
  0.2776585156719936  0.7217962339762714  0.2912536971284661   T   T   T
  0.7776920205088635  0.7218055566394398  0.2912673403503769   T   T   T
== Substitutional Te x4 on Pd(111)
   1.00000000000000
    16.1454760000000412    0.0000000000000066    0.0000000000006193
    -8.0727380000000792   13.9823920000000115    0.0000000000008174
     0.0000000000005753    0.0000000000009511   30.0000000000008242
   Pd   Te
   140     4
Selective dynamics
Direct
  0.0000000000009734  0.0000000000010019  0.0000050000008116   F   F   F
  0.1666666666666359  0.0000000000001350  0.0000050000002219   F   F   F
  0.3333333333333357  0.0000000000009379  0.0000050000000371   F   F   F
  0.5000000000004405  0.0000000000006679  0.0000050000009679   F   F   F
  0.6666666666666927  0.0000000000006395  0.0000050000001792   F   F   F
  0.8333333333332646  0.0000000000008455  0.0000050000009892   F   F   F
  0.0000000000007745  0.1666666666666288  0.0000050000009395   F   F   F
  0.1666666666666075  0.1666666666666075  0.0000050000005487   F   F   F
  0.3333333333331510  0.1666666666666075  0.0000050000009182   F   F   F
  0.5000000000001918  0.1666666666666146  0.0000050000005842   F   F   F
  0.6666666666666927  0.1666666666666501  0.0000050000007121   F   F   F
  0.8333333333339823  0.1666666666666785  0.0000050000005700   F   F   F
  0.0000000000004974  0.3333333333330586  0.0000050000006198   F   F   F
  0.1666666666665719  0.3333333333336697  0.0000050000009537   F   F   F
  0.3333333333335275  0.3333333333332362  0.0000050000003569   F   F   F
  0.5000000000004903  0.3333333333331439  0.0000050000000869   F   F   F
  0.6666666666661598  0.3333333333331439  0.0000050000008045   F   F   F
  0.8333333333331439  0.3333333333338899  0.0000050000002147   F   F   F
  0.0000000000009166  0.5000000000006466  0.0000050000005345   F   F   F
  0.1666666666661314  0.5000000000009308  0.0000050000005132   F   F   F
  0.3333333333338118  0.5000000000004476  0.0000050000008329   F   F   F
  0.5000000000008029  0.5000000000009592  0.0000050000007121   F   F   F
  0.6666666666661030  0.5000000000001350  0.0000050000002787   F   F   F
  0.8333333333333783  0.5000000000004903  0.0000050000003213   F   F   F
  0.0000000000004334  0.6666666666666430  0.0000050000006198   F   F   F
  0.1666666666668135  0.6666666666666501  0.0000050000006766   F   F   F
  0.3333333333338828  0.6666666666666430  0.0000050000001934   F   F   F
  0.5000000000009379  0.6666666666666359  0.0000050000000655   F   F   F
  0.6665283418613441  0.6666666666666146  0.0000050000001437   F   F   F
  0.8333333333334281  0.6666666666666856  0.0000050000007121   F   F   F
  0.1666666666666217  0.8333333333331865  0.0000050000003782   F   F   F
  0.0000000000003268  0.8333333333332362  0.0000050000004848   F   F   F
  0.3333333333334920  0.8333333333337052  0.0000050000005061   F   F   F
  0.5000000000000142  0.8333333333339397  0.0000050000000371   F   F   F
```

0.6666666666667069	0.8333333333338615	0.0000050000004279	F	F	F
0.8333333333333286	0.8333333333332931	0.0000050000001011	F	F	F
0.1108900226613059	0.0553692702790298	0.0755496194225168	T	T	T
0.2775329439748477	0.0550513761784218	0.0752674459848448	T	T	T
0.4444945198360559	0.0553727885189342	0.0755506550752166	T	T	T
0.6108886821886151	0.0553696306654460	0.0755501792257963	T	T	T
0.7775314342189158	0.0550522925190113	0.0752663970813271	T	T	T
0.9444925969039877	0.0553704225418470	0.0755488728074533	T	T	T
0.1110402621245939	0.2220713950360750	0.0755640668628704	T	T	T
0.2775345692014733	0.2223363535610026	0.0752633610769048	T	T	T
0.4448127148328472	0.2223353877839718	0.0752628316393965	T	T	T
0.6110413436687659	0.2220707775499522	0.0755658385102445	T	T	T
0.7775342528718828	0.2223370102038713	0.0752636436902323	T	T	T
0.9448114076870411	0.2223364481455319	0.0752617572041570	T	T	T
0.1110431448013077	0.3888101668040379	0.0755627682335828	T	T	T
0.2777792606623640	0.3888115049719804	0.0755636040206768	T	T	T
0.4445004748846368	0.3889860514397439	0.0755392588359229	T	T	T
0.6110453853244957	0.3888093500598844	0.0755627263144966	T	T	T
0.7777800156052308	0.3888116954755681	0.0755640224757968	T	T	T
0.9444994664995912	0.3889898236972172	0.0755411442469665	T	T	T
0.1108898607603682	0.5553715416137375	0.0755475946588724	T	T	T
0.2775281523463662	0.5550482884611352	0.0752672910697534	T	T	T
0.4444898956028018	0.5553679885609183	0.0755465110755277	T	T	T
0.6108915749786591	0.5553699361697360	0.0755467613170627	T	T	T
0.7775299925150485	0.5550487027257484	0.0752667296387666	T	T	T
0.9444915111509697	0.5553712795291209	0.0755481960349353	T	T	T
0.1110441285567868	0.7220773563335559	0.0755641146368777	T	T	T
0.2775363778128854	0.7223393298448028	0.0752638012332790	T	T	T
0.4448108497314679	0.7223363420336122	0.0752619787850469	T	T	T
0.6110402010090813	0.7220792826775665	0.0755716562145330	T	T	T
0.7775291036840041	0.7223343470493915	0.0752520155534885	T	T	T
0.9448125768811459	0.7223374559579243	0.0752628168501246	T	T	T
0.1110486680397996	0.8888145175668526	0.0755635719398534	T	T	T
0.2777857291995107	0.8888174993077369	0.0755650756443705	T	T	T
0.4445015290721754	0.8889895099619405	0.0755432272234519	T	T	T
0.6110465853674938	0.8888149553472889	0.0755629424481347	T	T	T
0.7777821255806532	0.8888161712885218	0.0755630101857114	T	T	T
0.9445005581711777	0.8889877611374745	0.0755427824253296	T	T	T
0.0555696282861108	0.1111192210705875	0.1518559202265704	T	T	T
0.2223301405068855	0.1110730189616060	0.1510538732662730	T	T	T
0.3887651215740211	0.1110735445984401	0.1510584205228734	T	T	T
0.5555670053820012	0.1111185996159753	0.1518621081990729	T	T	T
0.7223283807932609	0.1110717250172463	0.1510537049398821	T	T	T
0.8887630094231599	0.1110704873239061	0.1510538351072541	T	T	T
0.0553219560927367	0.2775598049498609	0.1515959931517914	T	T	T
0.2222482582924006	0.2775607110211208	0.1515966223680351	T	T	T
0.3887552896749596	0.2774987569739499	0.1510445335945284	T	T	T
0.5553267110496150	0.2775658979091986	0.1515971631612584	T	T	T
0.7222498928005974	0.2775608111686698	0.1515983259305827	T	T	T
0.8887559306898808	0.2774985409280785	0.1510442860123095	T	T	T
0.0555647660142355	0.4442555081478618	0.1518555690063730	T	T	T
0.2222515526759370	0.4444925102922572	0.1516043717125591	T	T	T
0.3887037379943230	0.4442512016794432	0.1518553939858398	T	T	T
0.5555628310627991	0.4442549974370925	0.1518526379538684	T	T	T

0.7222544309574570	0.4444941125852229	0.1516047831147462	T	T	T
0.8887061532427891	0.4442531676241151	0.1518563362729497	T	T	T
0.0555715302569557	0.6111243735028313	0.1518544454876319	T	T	T
0.2223266294869146	0.6110713650459559	0.1510531092594279	T	T	T
0.3887593821676661	0.6110708632758943	0.1510529670481435	T	T	T
0.5555689779411630	0.6111213452071738	0.1518527172047500	T	T	T
0.7223281167993600	0.6110725082689141	0.1510511073210676	T	T	T
0.8887622173838988	0.6110717522894044	0.1510545069756369	T	T	T
0.0553310708927074	0.7775721070142101	0.1515966172709601	T	T	T
0.2222569048975868	0.7775718534835993	0.1515966001699812	T	T	T
0.3887582385984099	0.7775013618326559	0.1510472174853093	T	T	T
0.5553285985530249	0.7775706847045758	0.1515950420035589	T	T	T
0.7222578891695762	0.7775697187220691	0.1515944100961875	T	T	T
0.8887585691504960	0.7774996345934040	0.1510468110056749	T	T	T
0.0555683553209324	0.9442571179225248	0.1518600484450749	T	T	T
0.2222644376211316	0.9445070611296589	0.1516044586616063	T	T	T
0.3887093656350971	0.9442537857204834	0.1518598054780245	T	T	T
0.5555679667183593	0.9442575353130588	0.1518602112702267	T	T	T
0.7222627464710420	0.9445044812928404	0.1516023999697523	T	T	T
0.8887086875483760	0.9442519662046542	0.1518577935852534	T	T	T
0.1668219574482618	0.9999794060433219	0.2276272125581606	T	T	T
0.3332051696411981	0.9999816358329529	0.2276319355012921	T	T	T
0.6668233913765753	0.9999786625478972	0.2276264772584897	T	T	T
0.8331997815290815	0.9999741086353149	0.2276291343144286	T	T	T
0.9998532599054269	0.1666397689323848	0.2276298399405773	T	T	T
0.1668035940294994	0.1666353828112030	0.2276299006451465	T	T	T
0.3332742193136091	0.1665296144649565	0.2257813567805215	T	T	T
0.4998581241002254	0.1666447068425269	0.2276323942065720	T	T	T
0.6668085026710255	0.1666341884783055	0.2276310888832518	T	T	T
0.8332779367643557	0.1665270526247716	0.2257782588417505	T	T	T
0.9998194950461119	0.3329669254813206	0.2276163286237674	T	T	T
0.1666025771515785	0.3331999889438938	0.2269091337726123	T	T	T
0.3331557823268108	0.3329704114429521	0.2276157412589607	T	T	T
0.4998173079983243	0.3329768995202679	0.2276150752950913	T	T	T
0.6666108614054694	0.3332037267669647	0.2269098087581807	T	T	T
0.8331662428132302	0.3329730330371502	0.2276174607707798	T	T	T
0.1667919560763186	0.4999783737843996	0.2276269180567840	T	T	T
0.3331852581281535	0.4999780725737618	0.2276258666905360	T	T	T
0.6667953915029093	0.4999793228075233	0.2276247915066798	T	T	T
0.8331901875169075	0.4999795900882794	0.2276282042943745	T	T	T
0.9998537462346864	0.6666411505271285	0.2276274254183735	T	T	T
0.1668015098786908	0.6666420033197122	0.2276268425317929	T	T	T
0.3332685058316328	0.6665281462584910	0.2257799668775358	T	T	T
0.4998488407984570	0.6666388942329102	0.2276243855339918	T	T	T
0.6667982966018285	0.6666384687895662	0.2276241328979433	T	T	T
0.8332692544433583	0.6665270735946756	0.2257804755908539	T	T	T
0.9998340689461295	0.8329838848279063	0.2276191849523563	T	T	T
0.1666194162029128	0.8332068322111649	0.2269096419448172	T	T	T
0.3331749252029151	0.8329782799283871	0.2276203555787209	T	T	T
0.4998344487042480	0.8329795846779033	0.2276191386706203	T	T	T
0.6666164244812043	0.8332018424176547	0.2269069024656501	T	T	T
0.8331734730007154	0.8329751398331005	0.2276177988770821	T	T	T
0.4999446189129723	0.4998821063622502	0.2321007170715540	T	T	T
0.9999412717857009	0.9998572697234361	0.2321064784886399	T	T	T

```
  0.4999527470383648  0.9998820560799484  0.2321107267838693   T   T   T
  0.9999370463440550  0.4998652863981791  0.2320993860438589   T   T   T
== On-top cluster Pd06-Te07
   1.00000000000000
    31.7040281100000030    0.0000000000000000    0.0000000000000000
     0.0000000000000000   31.5721201999999970    0.0000000000000000
     0.0000000000000000    0.0000000000000000   22.0000000000000000
   W    Te   Pd
    90   187     6
Selective dynamics
Direct
  0.0545293013356735  0.0780575145248989  0.4919126424239145   T   T   T
  0.0518973290690161  0.2773557078675634  0.4946801440383768   T   T   T
  0.0543966291281570  0.4787227132409983  0.4950363561218685   T   T   T
  0.0551522097439888  0.6788892385669535  0.4935187131551144   T   T   T
  0.0550719640333626  0.8786300133711824  0.4929714386370286   T   T   T
  0.1662793373135611  0.0778505656216541  0.4921345085824966   T   T   T
  0.1655999022825108  0.2770843700889433  0.5003381576936562   T   T   T
  0.1669399583662909  0.4793269363415919  0.4953477687289519   T   T   T
  0.1667494303219419  0.6793980890313451  0.4915893068925040   T   T   T
  0.1664823736690250  0.8789137185034303  0.4924041061444781   T   T   T
  0.2778514110726448  0.0786709861664089  0.4926479235830968   T   T   T
  0.2776954765387326  0.2753053371323042  0.4913553417075285   T   T   T
  0.2776272386822490  0.4796467448063726  0.4892426545903975   T   T   T
  0.2776896940399821  0.6804026990483412  0.4911102023666251   T   T   T
  0.2777328936329855  0.8791174973084882  0.4920706270305143   T   T   T
  0.3893797879713456  0.0779012824530415  0.4920100114226639   T   T   T
  0.3897879777805169  0.2771471934474313  0.5003743473494408   T   T   T
  0.3884241193733440  0.4793098011620807  0.4951548712523612   T   T   T
  0.3888300871551731  0.6794995758372780  0.4912979679456915   T   T   T
  0.3890203115994160  0.8789321275879528  0.4922209164298048   T   T   T
  0.5008187114978871  0.0782322797890023  0.4921315397495418   T   T   T
  0.5036691089782689  0.2775020272013681  0.4950386126534216   T   T   T
  0.5012519913732408  0.4786600687539532  0.4946761387170252   T   T   T
  0.5004583917317148  0.6789350773379385  0.4927866497253328   T   T   T
  0.5003932007547638  0.8787724289050455  0.4926823037327987   T   T   T
  0.6119947115122006  0.0781414850041379  0.4923967807381628   T   T   T
  0.6130560105084633  0.2782721185734597  0.4934900281023933   T   T   T
  0.6118799557831954  0.4788867413651725  0.4946414291630264   T   T   T
  0.6113407073746614  0.6787649350196125  0.4938722832343124   T   T   T
  0.6114712101538857  0.8785645088452553  0.4931914190273991   T   T   T
  0.7225442597153079  0.0781950376084581  0.4929824962088144   T   T   T
  0.7225836172371076  0.2783936925469737  0.4931588958709388   T   T   T
  0.7223152735138010  0.4788166802037178  0.4945150336089918   T   T   T
  0.7222768823316733  0.6787014434714702  0.4945855613931290   T   T   T
  0.7222479300855558  0.8784127314244684  0.4936484740633592   T   T   T
  0.8332273288131106  0.0780487587036827  0.4927860316496064   T   T   T
  0.8332971071425560  0.2784575738731619  0.4931916514229296   T   T   T
  0.8333601801067403  0.4789371230078101  0.4948331766840212   T   T   T
  0.8333055893571270  0.6786856238835970  0.4951464723385965   T   T   T
  0.8332880772463436  0.8783279872193198  0.4938640836323462   T   T   T
  0.9435059199602615  0.0779393448720276  0.4921508773285932   T   T   T
  0.9426935269150629  0.2782749356164956  0.4933340396009639   T   T   T
  0.9439293624234003  0.4788987478622151  0.4948518414822285   T   T   T
```

0.9440643750462104	0.6787842501183822	0.4946303468801869	T	T	T
0.9440090130275199	0.8783703386697805	0.4935379211763908	T	T	T
0.9989788776464152	0.0069969264748980	0.5020673872461575	T	T	T
0.9979140948718525	0.2065369973235582	0.5022978270877703	T	T	T
0.9985318082909501	0.4074769871534116	0.5046265624243813	T	T	T
0.9993348767264884	0.6077089297983220	0.5038472881690319	T	T	T
0.9994931469997403	0.8074544377970645	0.5030568122551342	T	T	T
0.1105885705265147	0.0072363809275770	0.5020552197727367	T	T	T
0.1111358633954110	0.2064160251575504	0.5037528406205282	T	T	T
0.1109616783021250	0.4075860684619751	0.5069059105759127	T	T	T
0.1104497359302655	0.6079630196752762	0.5026608542927372	T	T	T
0.1107273196494359	0.8078658045150426	0.5026116928303913	T	T	T
0.2220993060663713	0.0074577065394614	0.5020122361113629	T	T	T
0.2165761203955749	0.2063154649855202	0.5040180137616446	T	T	T
0.2233822203385508	0.4081822689235489	0.5033808417902159	T	T	T
0.2232952180852182	0.6095174142579651	0.5000161596985421	T	T	T
0.2220588400921289	0.8081676064895875	0.5017839677366036	T	T	T
0.3334310980443602	0.0074483697185762	0.5018805623811575	T	T	T
0.3389750214571720	0.2062831955928609	0.5040840460630558	T	T	T
0.3317734185874722	0.4082067604135652	0.5035152580049918	T	T	T
0.3323341350414058	0.6095961051309964	0.4999377775866672	T	T	T
0.3334139053820556	0.8082126914640880	0.5015894484974013	T	T	T
0.4448410606933457	0.0073034770979613	0.5020106294847039	T	T	T
0.4443939601383574	0.2065785181201825	0.5039804125442989	T	T	T
0.4446082871910591	0.4076310773788067	0.5069625469917541	T	T	T
0.4451725759900707	0.6080459933943775	0.5018440595426528	T	T	T
0.4448159368589489	0.8079126026251848	0.5022397065457729	T	T	T
0.5565045355174741	0.0072576569302924	0.5021579503731121	T	T	T
0.5577853984458565	0.2067172615628526	0.5026728648876574	T	T	T
0.5572600989401891	0.4075165439762815	0.5046139028686526	T	T	T
0.5562612738901018	0.6076155080005038	0.5030610812092182	T	T	T
0.5560082948957343	0.8076118309965129	0.5028344499004215	T	T	T
0.6671913145271393	0.0070770226165144	0.5025167243181770	T	T	T
0.6672839503670408	0.2069977226977723	0.5021740830677417	T	T	T
0.6672614679022992	0.4073307249315610	0.5034510056590648	T	T	T
0.6669387915198148	0.6075732231872979	0.5038261516045168	T	T	T
0.6668780613137096	0.8073412468716294	0.5030628399381758	T	T	T
0.7778166564719311	0.0070056558615010	0.5028758183519986	T	T	T
0.7779891844412187	0.2071129402545415	0.5020759566343500	T	T	T
0.7778913452585228	0.4073757808541579	0.5032467970621973	T	T	T
0.7777480497385553	0.6075239479244088	0.5045395959241608	T	T	T
0.7778118115204712	0.8073604087798039	0.5038215094622185	T	T	T
0.8883945523836820	0.0068843577104984	0.5025298117343300	T	T	T
0.8885069379817431	0.2069178848997042	0.5018961153493187	T	T	T
0.8885877199129733	0.4074362379812681	0.5035433727647591	T	T	T
0.8887413486007392	0.6075177885381955	0.5045622406264559	T	T	T
0.8886566773341169	0.8073232061884306	0.5038148776614352	T	T	T
0.9990054451628505	0.0583526163654996	0.4031586521829231	T	T	T
0.9985347213262097	0.2585298280234358	0.4040714302275800	T	T	T
0.9992907140275830	0.4588872040902661	0.4058317282186070	T	T	T
0.9991995360684366	0.6593067510875887	0.4051775754315299	T	T	T
0.9994514333507885	0.8589575659386346	0.4043100299672907	T	T	T
0.1103799227082648	0.0586906180877119	0.4033844663973186	T	T	T
0.1106410864921716	0.2599977056419750	0.4085623759314694	T	T	T

0.1107068998842509	0.4581572529877941	0.4076796062784050	T	T	T
0.1102925636134837	0.6592612925967352	0.4040148836560917	T	T	T
0.1105708790672915	0.8593070892543668	0.4039030355887012	T	T	T
0.2224003206111776	0.0594178963852634	0.4036162615699374	T	T	T
0.2192372101159479	0.2583147623997185	0.4066056748828380	T	T	T
0.2209153122826627	0.4586186019773121	0.4038452555307592	T	T	T
0.2220129974238974	0.6611125646326570	0.4018299021243860	T	T	T
0.2219377480145115	0.8597832626444620	0.4033153452202697	T	T	T
0.3333013983259392	0.0595060182310687	0.4035211223587830	T	T	T
0.3361003545281617	0.2581425300324370	0.4065820993409833	T	T	T
0.3343233480569999	0.4584657092647855	0.4038847863657237	T	T	T
0.3333271947127855	0.6612521308962368	0.4018103043454173	T	T	T
0.3334429439656110	0.8598640976880247	0.4031610204224961	T	T	T
0.4452131282775739	0.0587255998532875	0.4033004006467785	T	T	T
0.4449009833345271	0.2602105319498292	0.4088622097742535	T	T	T
0.4447357588734481	0.4580028916161089	0.4075701598631619	T	T	T
0.4451093987664657	0.6598304104058064	0.4034028280574053	T	T	T
0.4448754948586496	0.8593986007132179	0.4036615966370393	T	T	T
0.5564093447548895	0.0587333554755065	0.4033966284648053	T	T	T
0.5570053659890144	0.2586254645937202	0.4044299296871516	T	T	T
0.5564534706848721	0.4587706824283410	0.4056647574922876	T	T	T
0.5562721983599078	0.6591688468789734	0.4043419661331694	T	T	T
0.5560470714215812	0.8590175779161936	0.4040876065280347	T	T	T
0.6674026444947796	0.0585374957179369	0.4036552210460659	T	T	T
0.6675881187822443	0.2589326379220955	0.4036047826904068	T	T	T
0.6670659262655162	0.4592991067095690	0.4052854675960232	T	T	T
0.6670961587172309	0.6592381673938407	0.4052459403878234	T	T	T
0.6669414729587321	0.8588749771736551	0.4043710351504280	T	T	T
0.7778093042101871	0.0583492503421879	0.4039626971270556	T	T	T
0.7779670749597283	0.2592377594117564	0.4039870808068274	T	T	T
0.7779467141271270	0.4596666101105028	0.4055503188724612	T	T	T
0.7780702132437558	0.6589822399290358	0.4060262757730072	T	T	T
0.7778485194713415	0.8586172240523033	0.4048833906776128	T	T	T
0.8881601813844274	0.0580768849135076	0.4034105920801553	T	T	T
0.8881413982858534	0.2591168230385459	0.4035327526652937	T	T	T
0.8886349802182671	0.4596372006206974	0.4055933364797751	T	T	T
0.8884010440779857	0.6589554497998574	0.4059906271272806	T	T	T
0.8885575505530220	0.8585071945604321	0.4047316314956598	T	T	T
0.0547933099153969	0.1574971966637984	0.4320956416804632	T	T	T
0.0547661353128985	0.3579557972175121	0.4363258317742051	T	T	T
0.0546591260930798	0.5577948186719612	0.4336035349753732	T	T	T
0.0551319565735391	0.7579996507272101	0.4327564455243109	T	T	T
0.0549445417118202	0.9574839924970048	0.4320414926175817	T	T	T
0.1653374611895201	0.1569236014998228	0.4303727552488377	T	T	T
0.1661721869266264	0.3565958807549058	0.4388063165112984	T	T	T
0.1662368705168221	0.5582590287552470	0.4326430423226074	T	T	T
0.1661778694651823	0.7586247263802670	0.4316905017494130	T	T	T
0.1663615246353342	0.9578932813207169	0.4318309024864613	T	T	T
0.2777654413049393	0.1595448628929750	0.4359294854155389	T	T	T
0.2776715602685216	0.3567000531634278	0.4312892286313575	T	T	T
0.2776585580896426	0.5593905124367199	0.4275857235517683	T	T	T
0.2777265560347749	0.7592798220930985	0.4306634849678703	T	T	T
0.2777386776735939	0.9581123283645314	0.4314514528830636	T	T	T
0.3903587123474049	0.1571213062342525	0.4303629614291017	T	T	T

0.3891924666047338	0.3567466423266236	0.4390419491322390	T	T	T
0.3891052618271342	0.5582533031141728	0.4323947800596995	T	T	T
0.3892201492702948	0.7586705493699478	0.4314471776146324	T	T	T
0.3890587390377243	0.9578826266918359	0.4317041170600500	T	T	T
0.5008013172933975	0.1577759851554043	0.4324794914027636	T	T	T
0.5007983278304733	0.3580562422541820	0.4364939030150899	T	T	T
0.5008467173563667	0.5575466192001253	0.4328812619342232	T	T	T
0.5004827407488961	0.7580845959040703	0.4322605401452592	T	T	T
0.5004768065606610	0.9578181696125762	0.4320018893301930	T	T	T
0.6121960220588849	0.1570273300492670	0.4312802192355733	T	T	T
0.6119715543985650	0.3578192664317425	0.4333272113623706	T	T	T
0.6118012009442826	0.5578005224835582	0.4332742343834488	T	T	T
0.6113435605555513	0.7576490748855672	0.4328543744142822	T	T	T
0.6116438125266976	0.9573733530337730	0.4320147955885704	T	T	T
0.7225253460745769	0.1570744842408796	0.4318213087508668	T	T	T
0.7226378910361816	0.3576671394991211	0.4327446637933075	T	T	T
0.7224704535772766	0.5577902323748991	0.4337757006673637	T	T	T
0.7225555391745396	0.7574852319240840	0.4333577880764539	T	T	T
0.7224228832674112	0.9571759548084124	0.4325350605286399	T	T	T
0.8332083197004447	0.1569204982880958	0.4316563146721669	T	T	T
0.8332925696998862	0.3576916533811857	0.4327674077049021	T	T	T
0.8332360123741535	0.5578901260280543	0.4342630087747785	T	T	T
0.8332254621284978	0.7573779434363479	0.4340123295219052	T	T	T
0.8330836721319259	0.9569660636590233	0.4325465496434067	T	T	T
0.9434065413177695	0.1568348286696189	0.4307621165104709	T	T	T
0.9437468368589942	0.3578184323483073	0.4332238851892784	T	T	T
0.9439543081763933	0.5578661074826103	0.4338354289114983	T	T	T
0.9438801699350398	0.7575622179897209	0.4333742390446751	T	T	T
0.9437610917548008	0.9569644397446107	0.4322703401757907	T	T	T
0.9986492603926709	0.1276425018528071	0.5624631086626811	T	T	T
0.9977559393679956	0.3276506266676917	0.5651067181728360	T	T	T
0.9993085156955462	0.5287957686760488	0.5649933730453719	T	T	T
0.9998892643175282	0.7286154941040797	0.5642225929704153	T	T	T
0.9996742521456542	0.9282436093039794	0.5634456606013379	T	T	T
0.1098483482566569	0.1269008933121922	0.5625316802241556	T	T	T
0.1067508366564930	0.3282629196823774	0.5650738808866737	T	T	T
0.1103687097873883	0.5290747029610983	0.5639322695571771	T	T	T
0.1112044391622656	0.7286833356672673	0.5628250214026279	T	T	T
0.1109156373133872	0.9284314287108851	0.5628425298367586	T	T	T
0.2211085691902752	0.1269821925410489	0.5632886675562393	T	T	T
0.2253574392838828	0.3286993032940687	0.5605349848229206	T	T	T
0.2225315689490023	0.5315180153167730	0.5581374401401201	T	T	T
0.2220903605420971	0.7292787990097007	0.5620349883734609	T	T	T
0.2221389499321518	0.9285655570421247	0.5625907046593811	T	T	T
0.3346067662318700	0.1268911880094433	0.5634440644732862	T	T	T
0.3300749410716445	0.3287533933156431	0.5605333840006718	T	T	T
0.3329997916817427	0.5314954279733534	0.5579444007073934	T	T	T
0.3333732642180556	0.7293440121431227	0.5617398561381870	T	T	T
0.3333459984965374	0.9284813383513256	0.5625082450491914	T	T	T
0.4454821629687543	0.1270951514288507	0.5626730999537907	T	T	T
0.4487057221077667	0.3282804638711214	0.5651280534005073	T	T	T
0.4452566435388620	0.5291102414282014	0.5631487983227093	T	T	T
0.4445645208451846	0.7287767905093532	0.5623403038663638	T	T	T
0.4446410047521075	0.9284585551034454	0.5627329864065707	T	T	T

0.5567603761487503	0.1278186533275007	0.5626475099466259	T	T	T
0.5579020495044401	0.3277728973619762	0.5651419009628186	T	T	T
0.5563846645165624	0.5288089081248718	0.5645301649375514	T	T	T
0.5556619287195187	0.7285915657637206	0.5636529562377485	T	T	T
0.5558158596498721	0.9284500904427048	0.5631167290271301	T	T	T
0.6672071667499012	0.1280616647697481	0.5630862872486880	T	T	T
0.6674435286735331	0.3281343838271644	0.5643782759650885	T	T	T
0.6670492516516315	0.5285971661436981	0.5649444578054872	T	T	T
0.6666441110396154	0.7285555444892123	0.5643287102984228	T	T	T
0.6668016005978997	0.9282780205272084	0.5637104196398646	T	T	T
0.7780533346257522	0.1280877974537716	0.5629694614771301	T	T	T
0.7778975549074382	0.3281962271586111	0.5637328485713594	T	T	T
0.7775946196925122	0.5283860689648295	0.5651164481806803	T	T	T
0.7776677599011984	0.7284364747555074	0.5650005597912634	T	T	T
0.7778220170872551	0.9281550823220205	0.5639801092449506	T	T	T
0.8884523802336591	0.1279076191515161	0.5628432295369409	T	T	T
0.8883286545051383	0.3282267854537213	0.5643583758623458	T	T	T
0.8888154973915554	0.5284985495217194	0.5653698255631410	T	T	T
0.8887765249642033	0.7285011042680632	0.5651445300662599	T	T	T
0.8886667756882629	0.9281670084267970	0.5640076393654024	T	T	T
0.0547841840221913	0.0267177534084997	0.5906525552559140	T	T	T
0.0537569939621280	0.2251912058300583	0.5915167053307842	T	T	T
0.0540837008507269	0.4280850441924991	0.5939373489780019	T	T	T
0.0554388324253712	0.6274029151160242	0.5922374709464622	T	T	T
0.0552510089507864	0.8272059761649616	0.5916591931763933	T	T	T
0.1663078491128978	0.0263722306551788	0.5908741152382422	T	T	T
0.1640140528039570	0.2194520244581819	0.5976053712191610	T	T	T
0.1680339390004923	0.4278775933725570	0.5937991246152018	T	T	T
0.1671429388524446	0.6279903065138613	0.5898096634731250	T	T	T
0.1665706932645178	0.8275688257432435	0.5911046237730171	T	T	T
0.2778088328553804	0.0268098419040429	0.5908228152802797	T	T	T
0.2777181325806774	0.2234809181154211	0.5898065805504469	T	T	T
0.2775150682903558	0.4299154764157981	0.5912638747474328	T	T	T
0.2778653111564321	0.6286938959547173	0.5899606618598897	T	T	T
0.2778144122656488	0.8274787473661872	0.5906124672461202	T	T	T
0.3891068586227026	0.0265230031634192	0.5907918431015912	T	T	T
0.3914443279494909	0.2196439744210571	0.5976917168019200	T	T	T
0.3874030078689440	0.4280578170079534	0.5936699309760850	T	T	T
0.3888390971914183	0.6280261221220097	0.5894082509135643	T	T	T
0.3889194740541699	0.8274123377234243	0.5908040493059032	T	T	T
0.5005424827304448	0.0267037829589848	0.5907630503969793	T	T	T
0.5019332480177172	0.2251898779036391	0.5917819698418064	T	T	T
0.5016519547177877	0.4282386890114521	0.5938183662770843	T	T	T
0.5002933681615208	0.6274060850587578	0.5913164686048350	T	T	T
0.5001951538883305	0.8274341855078605	0.5914129530927175	T	T	T
0.6116780142389813	0.0268146633937035	0.5913761520790374	T	T	T
0.6126243350096710	0.2264392692533711	0.5920828267978484	T	T	T
0.6126189802735430	0.4270653109212733	0.5932935535249396	T	T	T
0.6113016672857540	0.6272073856272378	0.5925844230893276	T	T	T
0.6113711687229927	0.8270886914986315	0.5919150273925443	T	T	T
0.7223810816708862	0.0266975568704911	0.5917812500853452	T	T	T
0.7227413696267108	0.2263485473099192	0.5912946337812033	T	T	T
0.7226958918435166	0.4266871137105039	0.5924795263387449	T	T	T
0.7220242373261598	0.6271264878142586	0.5932294721572614	T	T	T

```
  0.7220791492204398  0.8269169535255827  0.5923682523526717   T   T   T
  0.8332716785737337  0.0266487612015417  0.5917151236526542   T   T   T
  0.8332693692472547  0.2263379963377964  0.5911955590809570   T   T   T
  0.8330543970178684  0.4266013937543272  0.5925266375223323   T   T   T
  0.8332274201377652  0.6270228009509288  0.5936304888194200   T   T   T
  0.8332564439710931  0.8269741842080663  0.5927993594932314   T   T   T
  0.9439224613882218  0.0267133093919603  0.5912712114423346   T   T   T
  0.9432549995481706  0.2263447912026023  0.5916988010689138   T   T   T
  0.9432788933185172  0.4270272613041046  0.5933524472840898   T   T   T
  0.9443324490543612  0.6272603651951678  0.5933189556440382   T   T   T
  0.9443738885455306  0.8270305747458353  0.5924052086767060   T   T   T
  0.2168423214039606  0.2821995813812928  0.7278805292599987   T   T   T
  0.3385551677705267  0.2824600589511073  0.7279705009922245   T   T   T
  0.1612989454804419  0.3497762295717032  0.7290381859460131   T   T   T
  0.2778991711600938  0.3733655485607003  0.7294842152845731   T   T   T
  0.3943526569233445  0.3498871783956286  0.7292831380336170   T   T   T
  0.2349498749026323  0.4872122152198125  0.7367675374009794   T   T   T
  0.3212819075230985  0.4874207540854852  0.7362630187870541   T   T   T
  0.1652172778480202  0.2985977268202303  0.6361775186411346   T   T   T
  0.2777344824138659  0.3063518774429924  0.6511804474498417   T   T   T
  0.3901777796106207  0.2987781240398296  0.6362890995369753   T   T   T
  0.2077981693436441  0.4140731620550678  0.7036116172096581   T   T   T
  0.3479950304047419  0.4141085977882013  0.7034085670401043   T   T   T
  0.2778470875425039  0.5122747223624340  0.6423671367056110   T   T   T
== On-top cluster Pd06-Te11
   1.00000000000000
    31.7040281100000030    0.0000000000000000    0.0000000000000000
     0.0000000000000000   31.5721201999999970    0.0000000000000000
     0.0000000000000000    0.0000000000000000   22.0000000000000000
   W    Te   Pd
    90   191     6
Selective dynamics
Direct
  0.0548083657878537  0.0779995663290048  0.4919177640422505   T   T   T
  0.0539812839897515  0.2777697022014854  0.4946550179719690   T   T   T
  0.0549913971545099  0.4786585173383631  0.4926275508577826   T   T   T
  0.0552437755895782  0.6786515909421938  0.4916582536222839   T   T   T
  0.0551782724738756  0.8785078608351149  0.4917757948307358   T   T   T
  0.1664565878908618  0.0777668558662606  0.4914427205037328   T   T   T
  0.1668749118682476  0.2771444433203232  0.4970840497474234   T   T   T
  0.1659897330232898  0.4788035532210373  0.4916891481779476   T   T   T
  0.1664279968607660  0.6790764435540988  0.4899056663683232   T   T   T
  0.1663057710866505  0.8785090095483686  0.4903892216776554   T   T   T
  0.2777142470491809  0.0775667342644110  0.4908199140099434   T   T   T
  0.2777523419604868  0.2766493276418788  0.4941463719249845   T   T   T
  0.2777430020631348  0.4793836702277303  0.4903468326969183   T   T   T
  0.2776569056260569  0.6796809779279804  0.4896847382780277   T   T   T
  0.2776908655588879  0.8785054911184226  0.4897147641784276   T   T   T
  0.3892098915817574  0.0777886270899916  0.4909403410560471   T   T   T
  0.3885430289366432  0.2771132695494254  0.4967401100147385   T   T   T
  0.3892769002070394  0.4788616114597132  0.4916694548962189   T   T   T
  0.3890067706059133  0.6792487290733510  0.4897358153952786   T   T   T
  0.3891470597049785  0.8785181751617427  0.4899022156472543   T   T   T
  0.5005179012308664  0.0780215877696902  0.4913377118232982   T   T   T
```

0.5015465564303119	0.2777644755107767	0.4948206479514632	T	T	T
0.5001451001799888	0.4785834268719439	0.4929449325756847	T	T	T
0.5003102177764019	0.6785986883169498	0.4912827682288972	T	T	T
0.5003367928120865	0.8783700402185538	0.4908137881568605	T	T	T
0.6114653303801462	0.0780718660049769	0.4921885318131605	T	T	T
0.6121644698671211	0.2781477996162365	0.4937232666512538	T	T	T
0.6116083969830656	0.4784888653110183	0.4936973736517115	T	T	T
0.6114559745304805	0.6784003321465751	0.4927247254739548	T	T	T
0.6113623406958657	0.8783365569502416	0.4920180320537967	T	T	T
0.7223474708358586	0.0781315521268903	0.4927987624675663	T	T	T
0.7225552363023576	0.2782318698134013	0.4932441122246884	T	T	T
0.7223976730284160	0.4785170619166201	0.4937331178033824	T	T	T
0.7223418199203319	0.6785299124711572	0.4938246480593939	T	T	T
0.7223494185853248	0.8782894808445464	0.4929737230667154	T	T	T
0.8331398907130624	0.0780894218469320	0.4928607929710772	T	T	T
0.8329347357634734	0.2783921751666397	0.4930441545879655	T	T	T
0.8331411363730479	0.4786368713008048	0.4937202024512837	T	T	T
0.8332178776248196	0.6785177710300645	0.4941264416204731	T	T	T
0.8332023670035117	0.8782744775500130	0.4933400880478206	T	T	T
0.9438486491486888	0.0781163056734635	0.4925722130026282	T	T	T
0.9433465209787413	0.2782571830699462	0.4931602302369978	T	T	T
0.9439127878373527	0.4786127251070944	0.4933063279786869	T	T	T
0.9441630472907117	0.6785781051426065	0.4933776650210778	T	T	T
0.9440537576855337	0.8783234348085761	0.4928482250220794	T	T	T
0.9994335052121518	0.0070478925144309	0.5019532278561470	T	T	T
0.9994065088791892	0.2068281860047247	0.5028706189079447	T	T	T
0.9990687976146656	0.4073584706266007	0.5031663257768969	T	T	T
0.9996231909252373	0.6074702740036579	0.5023811269507874	T	T	T
0.9997673885182566	0.8073098823612915	0.5020355464122869	T	T	T
0.1108002352490510	0.0070160887994114	0.5011922654794482	T	T	T
0.1107485923344456	0.2063300284659469	0.5027191908422607	T	T	T
0.1108615819042632	0.4077526332748919	0.5027691063895636	T	T	T
0.1107385008471952	0.6077286184347891	0.5008401594265213	T	T	T
0.1106842463497872	0.8075123779070994	0.5005421285321828	T	T	T
0.2221191395988966	0.0067519547467805	0.5002613405302314	T	T	T
0.2221320608038187	0.2058138820292606	0.5034555213790834	T	T	T
0.2231692086698220	0.4078897345889249	0.5019059108262044	T	T	T
0.2226373142777481	0.6088145544509678	0.4991327470958642	T	T	T
0.2219409250719133	0.8076790296231773	0.4995589702268770	T	T	T
0.3334741371767432	0.0067827891073345	0.4999070984534932	T	T	T
0.3333443455856078	0.2057872705434349	0.5031474591963960	T	T	T
0.3323767016815988	0.4078678560352484	0.5016558458427951	T	T	T
0.3329124909042449	0.6090203988795220	0.4991987127935977	T	T	T
0.3334654420774101	0.8077013129995422	0.4993503709586746	T	T	T
0.4447651817182405	0.0069204070123267	0.5003695958506850	T	T	T
0.4447776038306863	0.2063497747550035	0.5026438963838397	T	T	T
0.4441873806650423	0.4077255894649194	0.5030033816733723	T	T	T
0.4447959391933103	0.6078364101143814	0.5006992668526029	T	T	T
0.4447802864614504	0.8075291181054505	0.5000485492330122	T	T	T
0.5558661617608686	0.0069941868629526	0.5012392387402390	T	T	T
0.5560542062392192	0.2067426397956041	0.5030660191707634	T	T	T
0.5562934616199602	0.4073360683830252	0.5037925024478987	T	T	T
0.5558591754239789	0.6073332122081992	0.5019896014265878	T	T	T
0.5558668852475535	0.8072244773530208	0.5012621393751059	T	T	T

0.6668275573780075	0.0069517206457431	0.5019424345549350	T	T	T
0.6668972938169733	0.2068473270341768	0.5024830990072447	T	T	T
0.6671463296983421	0.4071248478678572	0.5031989410642608	T	T	T
0.6669025944186981	0.6072420313493604	0.5030537284559343	T	T	T
0.6667683793589907	0.8071889900893008	0.5022226777685432	T	T	T
0.7776877606157744	0.0069461985756206	0.5025754616206061	T	T	T
0.7777705576343060	0.2068821624925243	0.5022060841675587	T	T	T
0.7777194446340581	0.4071794779920809	0.5029082257510730	T	T	T
0.7777463097605863	0.6072995819725532	0.5035760731234143	T	T	T
0.7777739087012593	0.8071703739096698	0.5030015791697692	T	T	T
0.8885258855999713	0.0069974261970914	0.5026073127156789	T	T	T
0.8884700336948199	0.2069765170070779	0.5021730676459732	T	T	T
0.8883777408201226	0.4072500985824654	0.5027273067345593	T	T	T
0.8887358109328443	0.6073302862007508	0.5033457053448032	T	T	T
0.8886641640863781	0.8071980612278553	0.5029986007577403	T	T	T
0.9991166106078148	0.0585970225609963	0.4032852246700511	T	T	T
0.9995291098481358	0.2586094947572495	0.4046147572610179	T	T	T
0.9992539789956484	0.4585738951932607	0.4042730944700275	T	T	T
0.9991714167441038	0.6588796141589927	0.4037141264949344	T	T	T
0.9992953143792764	0.8588921179770672	0.4034934791117408	T	T	T
0.1103670101501421	0.0587916254243223	0.4028344656763689	T	T	T
0.1112978246461411	0.2594186548388931	0.4069314288976453	T	T	T
0.1103828177207386	0.4583732588382377	0.4035943212337930	T	T	T
0.1102633912805147	0.6590076812510788	0.4020412495871673	T	T	T
0.1102616736545897	0.8592275825634632	0.4020826846082441	T	T	T
0.2217899061201452	0.0586048160351138	0.4020158438141217	T	T	T
0.2214174486150034	0.2581498662049411	0.4063401429745264	T	T	T
0.2216435323861395	0.4589080026722975	0.4028554301722945	T	T	T
0.2219617940205574	0.6602501460760335	0.4006606247449825	T	T	T
0.2217386339550734	0.8592785567053105	0.4011028928164567	T	T	T
0.3335446448945977	0.0588267763508149	0.4018000244520496	T	T	T
0.3338390982763865	0.2583385871670751	0.4060883623365281	T	T	T
0.3335856121973853	0.4589575529235564	0.4027063188280531	T	T	T
0.3333695986427538	0.6604227268994515	0.4006278687046141	T	T	T
0.3334838696829884	0.8593447271309130	0.4009283854190837	T	T	T
0.4451041406674574	0.0587817066213707	0.4020909458678666	T	T	T
0.4443434460747511	0.2593196529207360	0.4068565470474911	T	T	T
0.4449171138840757	0.4583727514743016	0.4037095920367496	T	T	T
0.4451130860871758	0.6592023618959253	0.4018420074822978	T	T	T
0.4450039253901680	0.8590551093451154	0.4013878893171365	T	T	T
0.5562577797963398	0.0586646135919439	0.4027504273684671	T	T	T
0.5560890456622258	0.2585721240315338	0.4049455536999344	T	T	T
0.5560666774560397	0.4584782165535266	0.4048373787821438	T	T	T
0.5564093262335740	0.6588066119893677	0.4032579012956540	T	T	T
0.5562281095704289	0.8586251386221617	0.4025690771710033	T	T	T
0.6670920362659591	0.0586939142131156	0.4034764473466010	T	T	T
0.6670920318339520	0.2588166810840311	0.4041606384626673	T	T	T
0.6669596785765387	0.4588430503636236	0.4046791584550488	T	T	T
0.6673346060394710	0.6587455659741090	0.4043720443452498	T	T	T
0.6671625078268708	0.8587933212867095	0.4036207839395286	T	T	T
0.7778076272570554	0.0584515993562814	0.4038790498544911	T	T	T
0.7777173980846469	0.2588061526217945	0.4039043048944030	T	T	T
0.7777667092163130	0.4590765203457559	0.4046001599274622	T	T	T
0.7779022387771914	0.6589416923987286	0.4051551000108849	T	T	T

0.7779203645041379	0.8586162607933405	0.4042012776945108	T	T	T
0.8884120670409366	0.0583605084398846	0.4037648067904463	T	T	T
0.8883165046212266	0.2590524199855942	0.4038285219754031	T	T	T
0.8883681825231726	0.4591370673875811	0.4043766943145192	T	T	T
0.8883734365744819	0.6589607973198353	0.4049691673773765	T	T	T
0.8884835284890121	0.8585711150916651	0.4041636891744560	T	T	T
0.0549150208592984	0.1573719961843890	0.4319308700682650	T	T	T
0.0548013523697527	0.3573810887707063	0.4340180354521425	T	T	T
0.0547527219853547	0.5577287879314524	0.4317858009669492	T	T	T
0.0547674204265814	0.7577592733626044	0.4308307764535143	T	T	T
0.0549069057034558	0.9575350351706035	0.4314120243363013	T	T	T
0.1663817700365466	0.1571205058074336	0.4320318371703260	T	T	T
0.1667106559332778	0.3565481305617031	0.4346109318735482	T	T	T
0.1661814768754003	0.5581783317641070	0.4305608525657074	T	T	T
0.1660690636377901	0.7582139566371211	0.4295007516041161	T	T	T
0.1662027291152194	0.9576174669682727	0.4302072706028586	T	T	T
0.2777304738612225	0.1569894158164565	0.4314142634664336	T	T	T
0.2775995589566236	0.3564850942886666	0.4318355093754804	T	T	T
0.2778146320575414	0.5587232161089760	0.4287416691009448	T	T	T
0.2776914170619507	0.7585444246417083	0.4289250871109497	T	T	T
0.2777522932724944	0.9575542467942849	0.4294183295891958	T	T	T
0.3891919384080849	0.1572146694998108	0.4318014223476521	T	T	T
0.3888340045770079	0.3565069560693065	0.4343094581171602	T	T	T
0.3892367457591869	0.5583184168020439	0.4305865950323450	T	T	T
0.3892566114922366	0.7582799681016572	0.4291986563971264	T	T	T
0.3891697029718206	0.9576308944146045	0.4295380019868841	T	T	T
0.5006050665299864	0.1575873399897354	0.4317298989065450	T	T	T
0.5005994230791029	0.3573932198620068	0.4346110038235633	T	T	T
0.5005513533997660	0.5575229164464103	0.4317623734370747	T	T	T
0.5005388013655544	0.7576543342310432	0.4304406953618194	T	T	T
0.5005096139953823	0.9574682427003028	0.4303785558957818	T	T	T
0.6114932365159975	0.1572922954406685	0.4320406292379920	T	T	T
0.6115938767379424	0.3575796435397209	0.4333652160719885	T	T	T
0.6116178150775348	0.5573905494481858	0.4325472570800332	T	T	T
0.6116050811889332	0.7573055842212878	0.4314753571034288	T	T	T
0.6115471692517821	0.9573096774028402	0.4313583386975958	T	T	T
0.7223102583926864	0.1571317160908168	0.4320937265258333	T	T	T
0.7224519647029691	0.3573896470766564	0.4326205261251884	T	T	T
0.7224266103710160	0.5575691727126202	0.4330803418681472	T	T	T
0.7225088373722288	0.7573292230623987	0.4325787583659352	T	T	T
0.7223785655491529	0.9571469282232724	0.4321057116006596	T	T	T
0.8331895461217014	0.1570270998076160	0.4319183399551259	T	T	T
0.8330051686849278	0.3575243691464016	0.4323875258997606	T	T	T
0.8331689459191683	0.5576902673078045	0.4331108702946792	T	T	T
0.8332342053047024	0.7573933763256034	0.4328861849059191	T	T	T
0.8331645414385760	0.9571563954738699	0.4324188613204626	T	T	T
0.9439933459661034	0.1572595564028214	0.4320911265934707	T	T	T
0.9437670983959691	0.3576101890499910	0.4325724430823352	T	T	T
0.9438834476287481	0.5576671785041052	0.4325564063104564	T	T	T
0.9439358342983352	0.7574713180825460	0.4323735033549040	T	T	T
0.9438646142100539	0.9572729990854757	0.4321277432973087	T	T	T
0.9994530175314154	0.1275842439757780	0.5627179607215537	T	T	T
0.9985420491243974	0.3279662729987455	0.5638517176811337	T	T	T
0.9997388899282268	0.5284070619921549	0.5630423959279900	T	T	T

0.0001293755247214	0.7282313438936946	0.5627405940714166	T	T	T
0.9999023809330060	0.9280277546619331	0.5626570582465801	T	T	T
0.1106503723281395	0.1269149643225285	0.5622377532376704	T	T	T
0.1092480506420239	0.3288401442521164	0.5623266672012683	T	T	T
0.1106309080054305	0.5288334655062540	0.5620992124503047	T	T	T
0.1111683266553202	0.7284943005262239	0.5609009813642359	T	T	T
0.1111510330374457	0.9279517025906532	0.5615462254875911	T	T	T
0.2222578938584615	0.1262741734686836	0.5624744471934419	T	T	T
0.2240333528510962	0.3294973970075175	0.5618029712549190	T	T	T
0.2214582318545356	0.5305838468212095	0.5586653188568745	T	T	T
0.2219916246128547	0.7288813677330025	0.5599869103038214	T	T	T
0.2222051636262926	0.9277977236934943	0.5605498572643404	T	T	T
0.3333367274679832	0.1261616223767856	0.5621254066972289	T	T	T
0.3316359565016403	0.3295308382414347	0.5617593984451860	T	T	T
0.3339353380350366	0.5308150541670124	0.5585367205786892	T	T	T
0.3333508597637433	0.7290022575016292	0.5598665397776551	T	T	T
0.3333504259070320	0.9277730634905306	0.5603421267762764	T	T	T
0.4449324525323308	0.1269523453290005	0.5620195649842772	T	T	T
0.4460112209668776	0.3285725239104625	0.5622502390478064	T	T	T
0.4448296454764337	0.5289262196475880	0.5621999886209635	T	T	T
0.4445091954260794	0.7285744307364113	0.5605587049868094	T	T	T
0.4445249798065299	0.9279157525643765	0.5607111727285988	T	T	T
0.5558204596637809	0.1273969970213668	0.5625823039709249	T	T	T
0.5568825590162632	0.3278574897628302	0.5643827179059810	T	T	T
0.5556267826200384	0.5284059688590732	0.5630137967142632	T	T	T
0.5554594893647009	0.7282369371362878	0.5620361981795334	T	T	T
0.5554681692566540	0.9279492081012369	0.5617365495885585	T	T	T
0.6667234612037666	0.1277256814469573	0.5629456580816050	T	T	T
0.6672595401472109	0.3279010314265637	0.5640484548200135	T	T	T
0.6668733696936266	0.5281849668515973	0.5639922660541422	T	T	T
0.6665116874251185	0.7282965182642600	0.5633625682175848	T	T	T
0.6665583525267227	0.9279960037972409	0.5627949290952863	T	T	T
0.7778519670502944	0.1278353312168663	0.5630736780310419	T	T	T
0.7776975627285675	0.3280149736143702	0.5636453214799211	T	T	T
0.7777158145597334	0.5281645675536998	0.5641289291615038	T	T	T
0.7777592551828252	0.7282842516288037	0.5642413184996983	T	T	T
0.7776710256085296	0.9279612771783246	0.5635014699405394	T	T	T
0.8885039596698805	0.1278483384310838	0.5629134359730635	T	T	T
0.8883237596646448	0.3280801646787480	0.5634928563885940	T	T	T
0.8887548595847969	0.5282210997654143	0.5638045459107160	T	T	T
0.8888314040840033	0.7282341806299051	0.5640154413536831	T	T	T
0.8887562374186289	0.9279747820010018	0.5634557071182403	T	T	T
0.0553585557935398	0.0263986565026940	0.5904232765136370	T	T	T
0.0549261256172976	0.2255534275404162	0.5920553090474102	T	T	T
0.0550550205492332	0.4278183745512897	0.5915847050234532	T	T	T
0.0557557446909709	0.6272931702991359	0.5904740401668779	T	T	T
0.0557214851459196	0.8269359211444679	0.5903183851747484	T	T	T
0.1668268327960662	0.0259457525733488	0.5897165525335929	T	T	T
0.1655986776771135	0.2207096194828048	0.5942116185417763	T	T	T
0.1668089061162829	0.4278629689474715	0.5901661777309494	T	T	T
0.1668510496436890	0.6279932360453270	0.5886044840538714	T	T	T
0.1666612450019313	0.8270103557435911	0.5889069830342539	T	T	T
0.2779678952714467	0.0256946509593235	0.5890160813017096	T	T	T
0.2778174335518599	0.2224682437059755	0.5930481948676033	T	T	T

```
  0.2778285151295133  0.4277346477454558  0.5905592069132616   T   T   T
  0.2776542020183025  0.6280584307909313  0.5885176686377740   T   T   T
  0.2778116983672272  0.8270279060474235  0.5882664942136646   T   T   T
  0.3889369813665389  0.0259464694128589  0.5890624957568682   T   T   T
  0.3897694728819201  0.2206415261303434  0.5939634627673912   T   T   T
  0.3881777269284128  0.4279677224806098  0.5903348040246085   T   T   T
  0.3888581436402251  0.6283659977155129  0.5885164762929785   T   T   T
  0.3888938392543873  0.8271264089249610  0.5885359480671808   T   T   T
  0.5000099870993384  0.0263764268241012  0.5897731472174128   T   T   T
  0.5005077327336216  0.2256442592244927  0.5920904388348471   T   T   T
  0.5000300136102501  0.4276880802408070  0.5918891148505361   T   T   T
  0.4999576444565799  0.6273093445922142  0.5902817896207908   T   T   T
  0.4999212903751988  0.8269667011843571  0.5895615430377503   T   T   T
  0.6111094385676971  0.0263231155611588  0.5905905008580979   T   T   T
  0.6116757010871510  0.2260405268467926  0.5919861633037590   T   T   T
  0.6118004892326796  0.4269688808828214  0.5924329905658060   T   T   T
  0.6109881651337511  0.6268150233411243  0.5914286296854405   T   T   T
  0.6109543856574391  0.8269133402634301  0.5907026899003475   T   T   T
  0.7219659372050276  0.0263794241276508  0.5912505947794366   T   T   T
  0.7225048792088336  0.2261413340447669  0.5914190517720295   T   T   T
  0.7224853494895940  0.4268212908664655  0.5921965053585407   T   T   T
  0.7221100795315677  0.6268883352732789  0.5923926452794810   T   T   T
  0.7219953754422507  0.8267329877571145  0.5915514840481584   T   T   T
  0.8330637989991915  0.0265263886659781  0.5915646746488440   T   T   T
  0.8330903048837771  0.2263811787169376  0.5912077863759078   T   T   T
  0.8331183679468578  0.4266299149771359  0.5919456289297570   T   T   T
  0.8333071806839344  0.6266365606705686  0.5924544876858533   T   T   T
  0.8332216198228857  0.8266894200464967  0.5920480863516209   T   T   T
  0.9442050753112872  0.0265960769091398  0.5912645415503761   T   T   T
  0.9436710739111287  0.2264405336579851  0.5915462113625951   T   T   T
  0.9436224055939175  0.4270038516333934  0.5919042544996957   T   T   T
  0.9444925644908198  0.6268880864966591  0.5919438906194722   T   T   T
  0.9445761359898779  0.8267507950701447  0.5914361257425252   T   T   T
  0.2187967835412427  0.2752545619152710  0.7228080001977139   T   T   T
  0.3366372943628847  0.2749187078373628  0.7228108301358501   T   T   T
  0.1627287479687060  0.3450366501182934  0.7430291884549221   T   T   T
  0.2792372132791743  0.3764956433163051  0.7244320781346502   T   T   T
  0.3945284730485086  0.3435986487842212  0.7436718522527148   T   T   T
  0.2362125322573181  0.4900482135662062  0.7430283640397084   T   T   T
  0.3232527600551796  0.4901471239021239  0.7403989126438643   T   T   T
  0.1268795388615463  0.4362776179470338  0.7443843525729940   T   T   T
  0.4332833074476299  0.4330102987355945  0.7454466121128656   T   T   T
  0.1705438254245580  0.4345061337397426  0.8496902202500572   T   T   T
  0.3859651783744946  0.4378960684368849  0.8474153075344244   T   T   T
  0.1640259598034832  0.3006382431829368  0.6408506676472120   T   T   T
  0.2778081307791567  0.3078455508256241  0.6512916432286097   T   T   T
  0.3916879651119028  0.3002990817713240  0.6412067940591466   T   T   T
  0.2099857040110012  0.4135691322526298  0.7449005908761646   T   T   T
  0.3491151273500895  0.4133348815805177  0.7424957107556573   T   T   T
  0.2779324909835488  0.5062832535664672  0.6407314617197464   T   T   T
== On-top cluster Pd11-Te13
   1.00000000000000
     31.7040281100000030    0.0000000000000000    0.0000000000000000
      0.0000000000000000   31.5721201999999970    0.0000000000000000
```

```
     0.0000000000000000    0.0000000000000000   22.0000000000000000
   W    Te   Pd
    90   193    11
Selective dynamics
Direct
  0.0550775755205821  0.0776149098721424  0.4886601267265207   T   T   T
  0.0530491760547716  0.2770499961093616  0.4911068440387359   T   T   T
  0.0535184860162007  0.4787558816643412  0.4938475283043064   T   T   T
  0.0549789599541983  0.6789150612544107  0.4908491209209447   T   T   T
  0.0552348611156547  0.8783270056910085  0.4896798433359841   T   T   T
  0.1664496631883871  0.0775678735369032  0.4897358336509021   T   T   T
  0.1653852627132698  0.2766033251244363  0.4978778374108539   T   T   T
  0.1665679745529219  0.4794452580080565  0.4947032629192150   T   T   T
  0.1663507569859184  0.6793421072806431  0.4891401263448598   T   T   T
  0.1664547576432780  0.8785701579501719  0.4892964171430491   T   T   T
  0.2777541472852329  0.0778107771168302  0.4913368100905380   T   T   T
  0.2778324774417743  0.2760286135611635  0.4967807418967288   T   T   T
  0.2767902950168660  0.4796454628448547  0.4903326747162018   T   T   T
  0.2777078185049844  0.6803872628869595  0.4888178212274082   T   T   T
  0.2777103094959025  0.8787641038221692  0.4893108767530833   T   T   T
  0.3888938870140719  0.0777199842384842  0.4923910092116975   T   T   T
  0.3892234446211107  0.2766186123132808  0.5021771724094584   T   T   T
  0.3825590588595207  0.4785669758244319  0.4956397699931168   T   T   T
  0.3888868571832283  0.6796231092391877  0.4877185917769475   T   T   T
  0.3888997322218538  0.8788446122588222  0.4895232591867887   T   T   T
  0.5000758948885804  0.0776987786345148  0.4916033732551006   T   T   T
  0.5003616540359511  0.2762844489687483  0.4971869074998015   T   T   T
  0.5056560189125765  0.4797687613641129  0.4922790476046606   T   T   T
  0.4996807595010738  0.6797318769549623  0.4887195900615644   T   T   T
  0.4999330457339523  0.8787291253897100  0.4899619611704422   T   T   T
  0.6115099678426363  0.0773180158553575  0.4901537449480897   T   T   T
  0.6121433367739958  0.2762316868993354  0.4980492226837804   T   T   T
  0.6117891040055981  0.4793712172047235  0.4945205231214076   T   T   T
  0.6111027538106165  0.6793691758869144  0.4892684990835403   T   T   T
  0.6111509531427253  0.8783528095565001  0.4897575515179278   T   T   T
  0.7230720521812104  0.0773511231462656  0.4889029776687958   T   T   T
  0.7251230673354371  0.2770759905854921  0.4914346500724260   T   T   T
  0.7237757974232844  0.4785674762637813  0.4931402649294678   T   T   T
  0.7227999386262652  0.6787938715609906  0.4908138347455025   T   T   T
  0.7226857363751968  0.8781749334023339  0.4898766260737728   T   T   T
  0.8336987447897947  0.0774949931955683  0.4886244075945029   T   T   T
  0.8343440442026694  0.2777090187709456  0.4894080228405480   T   T   T
  0.8339493512473092  0.4787410401921900  0.4927317369176107   T   T   T
  0.8335550313428471  0.6786671733817933  0.4913514631586079   T   T   T
  0.8335530208894472  0.8781420211079458  0.4900383282503084   T   T   T
  0.9441989328655261  0.0774711451107097  0.4885207460632098   T   T   T
  0.9427972282755508  0.2776567020151740  0.4893172289360856   T   T   T
  0.9438250847447903  0.4787382364603035  0.4930140521997186   T   T   T
  0.9441445865013584  0.6787828604707390  0.4915748574035798   T   T   T
  0.9441655939327216  0.8781917228662601  0.4899790257624840   T   T   T
  0.9996553563170595  0.0066627217631729  0.4987077395078374   T   T   T
  0.9988977343652522  0.2060980819774603  0.4984677565608723   T   T   T
  0.9983565850046801  0.4069939425536739  0.5020734059782431   T   T   T
  0.9993178786137968  0.6077090013995962  0.5016036310156784   T   T   T
```

0.9996741919501270	0.8073760648943297	0.4999116057928638	T	T	T
0.1108234241848097	0.0068201991786463	0.4988992405413474	T	T	T
0.1112263939392314	0.2059088531000161	0.5000779109883943	T	T	T
0.1096197057819534	0.4077119571943145	0.5053754761517725	T	T	T
0.1100213138578660	0.6080018312543127	0.5006021861840431	T	T	T
0.1107667937985504	0.8075794758176409	0.4993364607187972	T	T	T
0.2221294137261755	0.0068473918880495	0.4996307489625209	T	T	T
0.2199431418746120	0.2057110895934046	0.5031043017610579	T	T	T
0.2231441887286705	0.4086528896071084	0.5043123604606196	T	T	T
0.2231653390306231	0.6094054078229684	0.4984529775358857	T	T	T
0.2220690873648077	0.8079130013113308	0.4985936005803573	T	T	T
0.3334477080856711	0.0069235236495733	0.5006103532709328	T	T	T
0.3344680818723901	0.2058141644004138	0.5057800399135259	T	T	T
0.3279923207650555	0.4077082899912089	0.5057628307937843	T	T	T
0.3318501542938491	0.6097301766813262	0.4980893449182299	T	T	T
0.3334627780358702	0.8080546987759613	0.4984343974102807	T	T	T
0.4443961304129923	0.0069057709023335	0.5008970296828890	T	T	T
0.4440540453848473	0.2058100725523096	0.5058057778469258	T	T	T
0.4502486562731516	0.4085781114518727	0.5069396126441436	T	T	T
0.4449469762371754	0.6085523151725921	0.4972006383904274	T	T	T
0.4444590513248460	0.8079461390835853	0.4987463233692495	T	T	T
0.5556553604253590	0.0067238999129251	0.5001772339358495	T	T	T
0.5574674306620788	0.2055765587127767	0.5034164725216800	T	T	T
0.5566590683692881	0.4081228877729268	0.5044099261613103	T	T	T
0.5548734196761268	0.6095554775400185	0.4989213936950586	T	T	T
0.5554430215130619	0.8076883579865768	0.4990779572972154	T	T	T
0.6671465220218005	0.0065729034945330	0.4994458674165047	T	T	T
0.6670545111211310	0.2056195510722458	0.5005686339142210	T	T	T
0.6670722365986289	0.4075199213325468	0.5049653500854540	T	T	T
0.6673727268364484	0.6079911928125745	0.5006598367313557	T	T	T
0.6669533333617917	0.8074591065652740	0.4996068883689352	T	T	T
0.7784994680875132	0.0065146797840484	0.4987089203726548	T	T	T
0.7789154889489709	0.2060423573716149	0.4983795283819293	T	T	T
0.7794699130361237	0.4070444361225597	0.5017879036720078	T	T	T
0.7784624850554852	0.6075801289590146	0.5010173991266086	T	T	T
0.7781231992735105	0.8072233527718355	0.4998677960634519	T	T	T
0.8888656350747820	0.0065381886054593	0.4985918683262190	T	T	T
0.8885312391661602	0.2060926831389867	0.4975177700688364	T	T	T
0.8887543364573720	0.4069159727651749	0.5009194307453894	T	T	T
0.8888320016342113	0.6075331943126633	0.5014500439582202	T	T	T
0.8887244725688839	0.8071790906432953	0.4999634677925765	T	T	T
0.9997825857347009	0.0579255631767517	0.3997209589950951	T	T	T
0.9992309503477219	0.2583187607796895	0.4006107600867367	T	T	T
0.9992404174261406	0.4588996385004778	0.4039142815935123	T	T	T
0.9994023106115986	0.6588078953220352	0.4024631521012991	T	T	T
0.9996793680626220	0.8586676721029073	0.4009559874227738	T	T	T
0.1111316271435975	0.0584168143447488	0.4003661194522824	T	T	T
0.1117683323055661	0.2598459009419054	0.4047890229755878	T	T	T
0.1104259869358560	0.4583930772821811	0.4067816863884134	T	T	T
0.1101966022943315	0.6588731683195114	0.4015289682511408	T	T	T
0.1107665946713625	0.8588966423295316	0.4006201698209773	T	T	T
0.2227181357450975	0.0588461442186856	0.4014661400509665	T	T	T
0.2217006908188284	0.2593124626574753	0.4079126583324024	T	T	T
0.2207167345170166	0.4583810946271763	0.4039860753537683	T	T	T

0.2219025772782972	0.6606515281081460	0.3999335864744250	T	T	T
0.2220565846719799	0.8596437166997859	0.4002285070159708	T	T	T
0.3336310123718533	0.0591642135955312	0.4027105910214611	T	T	T
0.3349347474625056	0.2590924169127376	0.4100914876166904	T	T	T
0.3318818504635973	0.4557116661654977	0.4035877957543537	T	T	T
0.3328806870926734	0.6608102108656326	0.3991306325597179	T	T	T
0.3333606392196495	0.8599164150089884	0.4002910898661032	T	T	T
0.4442638856729882	0.0589383996282674	0.4028660194597820	T	T	T
0.4434416284759348	0.2590448901073803	0.4101222363009599	T	T	T
0.4439352881600056	0.4596597901723706	0.4108761878222913	T	T	T
0.4446092701852306	0.6600340755891858	0.3989399318093358	T	T	T
0.4445714326767827	0.8598643851766794	0.4005311798860641	T	T	T
0.5553303221089019	0.0585748820348980	0.4018766193447846	T	T	T
0.5560237213336152	0.2590519561841745	0.4079349886332418	T	T	T
0.5579036535452319	0.4575652615423557	0.4032009724681392	T	T	T
0.5556741981596970	0.6605971892388601	0.3998598511598834	T	T	T
0.5554655451491540	0.8594305567461334	0.4008514476943137	T	T	T
0.6668878342612085	0.0579499171425619	0.4007413099841490	T	T	T
0.6662436118906199	0.2595062840041734	0.4054778353920868	T	T	T
0.6672997859042427	0.4581357460245605	0.4059060540320899	T	T	T
0.6674085355932615	0.6589038756550671	0.4016173762940690	T	T	T
0.6669472089510530	0.8589277318937489	0.4010136141933885	T	T	T
0.7781974183490732	0.0577708414306598	0.3997188656887477	T	T	T
0.7782925804678525	0.2584160365109322	0.4002761369469846	T	T	T
0.7785765274583270	0.4589277188875014	0.4036272653026907	T	T	T
0.7782438972558844	0.6589998415435999	0.4021034801005222	T	T	T
0.7781487199655787	0.8584793027283462	0.4009634738389531	T	T	T
0.8889532610129189	0.0578229291587922	0.3995691101158220	T	T	T
0.8885217473219015	0.2583196786529814	0.3990452151278754	T	T	T
0.8889558043151271	0.4593563613429609	0.4032778126692865	T	T	T
0.8889836289533757	0.6588368900144761	0.4024974996030363	T	T	T
0.8888507748464932	0.8584218801776525	0.4009943417922138	T	T	T
0.0553940180669124	0.1570960339382764	0.4284171727463468	T	T	T
0.0548396793352585	0.3580795353044289	0.4332615215057044	T	T	T
0.0543658741430118	0.5574897030639210	0.4317521898728536	T	T	T
0.0549115657794884	0.7577823013562842	0.4296501415921019	T	T	T
0.0553586256079701	0.9571103575069583	0.4286007083131469	T	T	T
0.1666620178097505	0.1568715720603659	0.4289452774819230	T	T	T
0.1661877861354568	0.3574032720443772	0.4378669787370566	T	T	T
0.1659375023425291	0.5580659730200958	0.4315552321621290	T	T	T
0.1661742132874229	0.7583500249602607	0.4285192573642485	T	T	T
0.1665911661062418	0.9575847527425199	0.4287194959309274	T	T	T
0.2781730370224457	0.1576703388498503	0.4327573460189775	T	T	T
0.2774594314120526	0.3563290060929700	0.4335455321068341	T	T	T
0.2769978422604201	0.5588351937452113	0.4269744274738964	T	T	T
0.2776886948992743	0.7590482816556471	0.4276184693387797	T	T	T
0.2779035651536045	0.9578845093121168	0.4293204903872032	T	T	T
0.3890326340850973	0.1572429235436472	0.4327909411133958	T	T	T
0.3891485197547018	0.3582144456266365	0.4444955149076276	T	T	T
0.3869827038143526	0.5573303094071150	0.4308332004271440	T	T	T
0.3890117423892830	0.7588464142750605	0.4276253281764794	T	T	T
0.3888586227090753	0.9579861631565599	0.4296726961540185	T	T	T
0.4998418996047183	0.1574264148856446	0.4327141567973805	T	T	T
0.5003322386107076	0.3569069393703463	0.4350742415409644	T	T	T

0.5017812430539081	0.5582580856980136	0.4278362252308762	T	T	T
0.5000079184853063	0.7586991975678832	0.4280149593931142	T	T	T
0.4998788026964734	0.9577125344354422	0.4298643969698518	T	T	T
0.6112891431880346	0.1566357162719287	0.4299539055394553	T	T	T
0.6117856944579982	0.3564675936602013	0.4369506364779029	T	T	T
0.6118564422645486	0.5581345730950208	0.4315970079256586	T	T	T
0.6115008438426488	0.7582866710834623	0.4290160475517479	T	T	T
0.6112168280627274	0.9573636542552794	0.4294692892524809	T	T	T
0.7225032690854443	0.1567028446969717	0.4285059932132430	T	T	T
0.7229040551709237	0.3579067797423567	0.4333644397317734	T	T	T
0.7231427411544021	0.5574688921334369	0.4316214173647251	T	T	T
0.7225175887893550	0.7576279583742467	0.4296615213376994	T	T	T
0.7224632547662091	0.9569225740572065	0.4290084223648487	T	T	T
0.8335237173830861	0.1562549332730404	0.4270127781518928	T	T	T
0.8336390289297640	0.3574220036397940	0.4298287976173109	T	T	T
0.8337229396207338	0.5575743745712795	0.4312740137094238	T	T	T
0.8334397434304255	0.7573235191519971	0.4297626677457189	T	T	T
0.8335719380923952	0.9566773559801385	0.4283629057045488	T	T	T
0.9440297419101890	0.1563949940760191	0.4271795487248208	T	T	T
0.9439453712088570	0.3575318038219945	0.4300898602597818	T	T	T
0.9441372488756620	0.5575857367234217	0.4316041976926993	T	T	T
0.9442599895481821	0.7574571096408047	0.4300146223867627	T	T	T
0.9443856562322076	0.9567777745854815	0.4286227272333544	T	T	T
0.9993261889822904	0.1272978287965048	0.5587781402797111	T	T	T
0.9977687174780053	0.3267357402373166	0.5612315676979891	T	T	T
0.9988519889472718	0.5289380080574374	0.5635812712921350	T	T	T
0.9998104067541360	0.7286260743560716	0.5610653147685425	T	T	T
0.9998573220627416	0.9278652328211766	0.5599879328892597	T	T	T
0.1100811464409287	0.1265339479087521	0.5596891065057216	T	T	T
0.1062517336316494	0.3279746123194296	0.5617678979272267	T	T	T
0.1095997024329436	0.5292384610363596	0.5623059997514795	T	T	T
0.1110615705394726	0.7286660870644062	0.5599438690655015	T	T	T
0.1108998251560436	0.9279861178554935	0.5596850206990367	T	T	T
0.2212281636685690	0.1260003186271419	0.5618667921828956	T	T	T
0.2228711704975449	0.3291713261767122	0.5600420550009435	T	T	T
0.2215999873356114	0.5320017521296592	0.5584023222933443	T	T	T
0.2219388748604441	0.7292301165647976	0.5591670459406590	T	T	T
0.2219462533099690	0.9278323770559367	0.5598918571632270	T	T	T
0.3330155722037721	0.1259605273287293	0.5637947749299443	T	T	T
0.3305221934274162	0.3289445494181645	0.5665968490589895	T	T	T
0.3315152171303988	0.5322789451401625	0.5595473591992570	T	T	T
0.3335503791433573	0.7293362199861686	0.5586357047315055	T	T	T
0.3332316211519495	0.9277353911262964	0.5604127394850587	T	T	T
0.4448843711009079	0.1259371030892909	0.5640088979172845	T	T	T
0.4473848665743062	0.3295598733056172	0.5664633717735817	T	T	T
0.4444965978660831	0.5292464224327060	0.5539264837962297	T	T	T
0.4442191656238746	0.7289573863779174	0.5588969342999599	T	T	T
0.4444432802953282	0.9277700038428595	0.5606938344189074	T	T	T
0.5565820091935617	0.1257651625981371	0.5621336554554055	T	T	T
0.5553658782238867	0.3290362881218695	0.5607478567739758	T	T	T
0.5580540496441342	0.5325759336444776	0.5613047678660866	T	T	T
0.5552787592007415	0.7288490529875608	0.5592611075174101	T	T	T
0.5557538160853051	0.9277135320405165	0.5605110206807804	T	T	T
0.6682244369062046	0.1261676510310202	0.5599341280174227	T	T	T

0.6712902247309496	0.3277425886673316	0.5616712013443663	T	T	T
0.6682530682550468	0.5292727039028794	0.5624318476535186	T	T	T
0.6667660981978613	0.7285555541475288	0.5601569602149135	T	T	T
0.6670446442090640	0.9276130084456735	0.5601199846165676	T	T	T
0.7787553434529886	0.1271664785150333	0.5590337551528795	T	T	T
0.7798714893016834	0.3270585285032628	0.5615911184665795	T	T	T
0.7786574619968586	0.5287542799064484	0.5626224831242506	T	T	T
0.7779484660547042	0.7284915540385093	0.5610097634138816	T	T	T
0.7781917785937892	0.9278795652131618	0.5601112165073807	T	T	T
0.8889827744692569	0.1273981810996907	0.5588405486668521	T	T	T
0.8886401742234412	0.3272707262262757	0.5609513792798108	T	T	T
0.8886497430514426	0.5286636283096821	0.5630273023264929	T	T	T
0.8887582652893172	0.7285733334104627	0.5614040159582359	T	T	T
0.8888512930515723	0.9279588364614854	0.5600796176975521	T	T	T
0.0551341437772595	0.0263924519554972	0.5876443793160966	T	T	T
0.0541221041286984	0.2248814887660494	0.5881210847968419	T	T	T
0.0530051160921554	0.4275874559145259	0.5925082888729900	T	T	T
0.0551468471605956	0.6278979508902290	0.5900295329759365	T	T	T
0.0553805887786642	0.8270538032473342	0.5885477051296188	T	T	T
0.1661391376518244	0.0258429723037329	0.5882041900311472	T	T	T
0.1640902838185788	0.2187044465796706	0.5937780527756988	T	T	T
0.1665099492181169	0.4284639532550524	0.5929189595223501	T	T	T
0.1666152605750090	0.6284572862928872	0.5876799032434172	T	T	T
0.1665642866014572	0.8272099914967465	0.5879170093646701	T	T	T
0.2773623983485478	0.0256701394622194	0.5892366552061222	T	T	T
0.2760317187315835	0.2212994572191277	0.5942770154914603	T	T	T
0.2760982905375620	0.4311016358809616	0.5945839425490232	T	T	T
0.2775196638977591	0.6290117120714975	0.5882127623322458	T	T	T
0.2777511424901443	0.8269973644788533	0.5876090758740340	T	T	T
0.3887744892902246	0.0254121851669302	0.5901770789105315	T	T	T
0.3891501384296350	0.2190751448478171	0.5980377666693310	T	T	T
0.3886276758436291	0.4273269655278817	0.5919199247825365	T	T	T
0.3888732203837873	0.6283099728928383	0.5859487996002979	T	T	T
0.3888174888757189	0.8271549692322375	0.5878395239787830	T	T	T
0.5003299850597411	0.0256012109891123	0.5896708795552306	T	T	T
0.5019139441851687	0.2213738580675325	0.5947146595898180	T	T	T
0.5029879993234138	0.4301621993757568	0.5947265635889827	T	T	T
0.4992407068565104	0.6276998526931045	0.5876369456408497	T	T	T
0.4999271685738532	0.8268707175139665	0.5881947047745161	T	T	T
0.6117383796786545	0.0256940736342325	0.5886663322707331	T	T	T
0.6137803918036964	0.2182174600251185	0.5941383890218573	T	T	T
0.6117793610392095	0.4288733031993974	0.5939738690936074	T	T	T
0.6111795527522182	0.6288045237681388	0.5881286037027714	T	T	T
0.6111378503234515	0.8268178544793097	0.5882111384801969	T	T	T
0.7231165437001534	0.0260676911574696	0.5878750811760148	T	T	T
0.7239866246274003	0.2247914416777021	0.5884038935396829	T	T	T
0.7243387283058326	0.4274755070956951	0.5916070247294426	T	T	T
0.7227940580739225	0.6275581568710342	0.5896776031988841	T	T	T
0.7224346275038497	0.8269194069059335	0.5886760845556047	T	T	T
0.8337264840186521	0.0262708919421408	0.5877958230480005	T	T	T
0.8340029742303819	0.2256266871373509	0.5875718473525248	T	T	T
0.8342118796493079	0.4265454905884355	0.5910410388336718	T	T	T
0.8334367623873505	0.6273564656670766	0.5904108522058188	T	T	T
0.8333631975090663	0.8268304610502908	0.5890050572297193	T	T	T

```
  0.9442577159658153  0.0262502180720486  0.5875862472188189   T   T   T
  0.9434411421882377  0.2256027764673225  0.5872920352339395   T   T   T
  0.9432667229864972  0.4264026552604953  0.5910893659871560   T   T   T
  0.9440104427337873  0.6275610512221078  0.5905978840215005   T   T   T
  0.9442212379963684  0.8269694125515711  0.5888947452357874   T   T   T
  0.2122027978306026  0.2815368484814549  0.7290126940288719   T   T   T
  0.3306301445966188  0.2785649331338729  0.7264795970631082   T   T   T
  0.4472458835770526  0.2791807004417475  0.7266783037337582   T   T   T
  0.5652350008420450  0.2811354777786823  0.7291211691876301   T   T   T
  0.1593650023277255  0.3509393972026336  0.7258083799280052   T   T   T
  0.2758085329517584  0.3703968670107305  0.7291600884041982   T   T   T
  0.3877660785469662  0.3546476975767469  0.7383793597632136   T   T   T
  0.5000654669666995  0.3705369289469591  0.7300384317087356   T   T   T
  0.6171489063910258  0.3511127955254060  0.7261084150498830   T   T   T
  0.2330550975878738  0.4881833838752492  0.7376817642960333   T   T   T
  0.3198539446986133  0.4911533914318812  0.7392849774550432   T   T   T
  0.4554485561760143  0.4921751083960146  0.7357000582159255   T   T   T
  0.5422034693412183  0.4886792430721251  0.7356419010429037   T   T   T
  0.1626502599102256  0.2975813069862118  0.6350349739632977   T   T   T
  0.2721610573038453  0.3044037392647613  0.6493809975205832   T   T   T
  0.3890100870349555  0.3006046285123045  0.6445835904856615   T   T   T
  0.5054416315439141  0.3048903513011650  0.6495767188461508   T   T   T
  0.6153375940193597  0.2970843837562752  0.6357040014883890   T   T   T
  0.2076560861299698  0.4146836756895171  0.7017499958342711   T   T   T
  0.3423791419038743  0.4169986626899776  0.7023316591232399   T   T   T
  0.4335031552214602  0.4169308536396798  0.7029265480211467   T   T   T
  0.5685135400604240  0.4146728357271589  0.7023184469641847   T   T   T
  0.2761729351704494  0.5148159450646249  0.6445176140309202   T   T   T
  0.4987504528773475  0.5127952765254005  0.6402470128434277   T   T   T
== On-top cluster Pd11-Te18
   1.00000000000000
    31.7040281100000030    0.0000000000000000    0.0000000000000000
     0.0000000000000000   31.5721201999999970    0.0000000000000000
     0.0000000000000000    0.0000000000000000   22.0000000000000000
   W    Te   Pd
    90   198    11
Selective dynamics
Direct
  0.0555888128670060  0.0774096287630259  0.4862319961344264   T   T   T
  0.0535150586403692  0.2764300195422094  0.4905829774948282   T   T   T
  0.0539077770354261  0.4787877047096868  0.4946937302295304   T   T   T
  0.0560467234531962  0.6786507995987863  0.4899315460774066   T   T   T
  0.0559437178403639  0.8779748894417870  0.4867611930810612   T   T   T
  0.1671576325284771  0.0773713565401384  0.4880231439929556   T   T   T
  0.1667977218823050  0.2767033572686112  0.4986575445210240   T   T   T
  0.1607900084443938  0.4780635006781583  0.4957164610965147   T   T   T
  0.1671562143541900  0.6785978349940711  0.4888787924663383   T   T   T
  0.1671825574618788  0.8781042820359201  0.4866805178175402   T   T   T
  0.2785900424358926  0.0770055821352481  0.4901767852687097   T   T   T
  0.2784134492194835  0.2756996944569398  0.5002779726118999   T   T   T
  0.2834375036210186  0.4793897440179283  0.4960507546119602   T   T   T
  0.2781330062975471  0.6791724826270756  0.4886683019385553   T   T   T
  0.2782955383659145  0.8779823558315429  0.4865656822063395   T   T   T
  0.3894955453153685  0.0771365821280249  0.4912484349066984   T   T   T
```

0.3891456492203100	0.2749859144978142	0.5020279319329926	T	T	T
0.3861832501576607	0.4786037119064550	0.4987912966514543	T	T	T
0.3899935982168342	0.6790671960599427	0.4873513841233059	T	T	T
0.3897289420325301	0.8779815557753022	0.4861052540527068	T	T	T
0.5007316075702789	0.0771649359846449	0.4895938268236945	T	T	T
0.5016299687696683	0.2767917781837015	0.5003666960882313	T	T	T
0.5075404928056573	0.4788037257168141	0.4917655959045242	T	T	T
0.5013316324160417	0.6792726401552374	0.4868669715950211	T	T	T
0.5008748184861055	0.8782383811578579	0.4857074729395006	T	T	T
0.6119556061039839	0.0773522251470858	0.4876804616003269	T	T	T
0.6129569548795990	0.2763061301520655	0.4952362413624740	T	T	T
0.6151994207778184	0.4794589236182958	0.4904360106259736	T	T	T
0.6121451578859193	0.6794027293456614	0.4861700211766658	T	T	T
0.6120036043397264	0.8784180860947546	0.4853679752787640	T	T	T
0.7238749128848333	0.0775753729964025	0.4862245437841409	T	T	T
0.7243767561431956	0.2772792717023475	0.4911479939025881	T	T	T
0.7239981575464226	0.4787421281650992	0.4903333583698050	T	T	T
0.7234548172457560	0.6788797445197247	0.4871745733414735	T	T	T
0.7233719467121090	0.8782590556390582	0.4855424057182915	T	T	T
0.8343739135256213	0.0776205470965672	0.4858973153047550	T	T	T
0.8348371659912782	0.2778814227015099	0.4891671741819667	T	T	T
0.8345775518783942	0.4785615483604229	0.4910735721091508	T	T	T
0.8342750599513586	0.6785901711264236	0.4886905308272576	T	T	T
0.8341859199143929	0.8780527843647105	0.4862212355070168	T	T	T
0.9446453367902485	0.0774579747755905	0.4857876957574394	T	T	T
0.9437444242834123	0.2776186105138446	0.4890934949189369	T	T	T
0.9446385314918224	0.4785656460088430	0.4929979878461123	T	T	T
0.9447319705019470	0.6785196086587827	0.4897832194734069	T	T	T
0.9447889531915046	0.8779587274955009	0.4867016652480515	T	T	T
0.0001472507384183	0.0064355900146509	0.4954231369825300	T	T	T
0.9993156510282570	0.2057686786772785	0.4973189026789702	T	T	T
0.9992770968112560	0.4070279502821593	0.5027787585026052	T	T	T
0.0002962921390415	0.6076015065219212	0.5009327370302542	T	T	T
0.0002776435508159	0.8070285476947919	0.4970559091509659	T	T	T
0.1115645858143298	0.0065666890063233	0.4961120394106707	T	T	T
0.1119308458250322	0.2056089119018420	0.4991249060951014	T	T	T
0.1056988381522548	0.4068256997675317	0.5053682619219022	T	T	T
0.1109609750475326	0.6077854662638731	0.5010272015233782	T	T	T
0.1116736570240635	0.8071869357565797	0.4970412681356233	T	T	T
0.2228120370746273	0.0062753147890743	0.4973258110613012	T	T	T
0.2223567755840954	0.2051339109455061	0.5037637827885735	T	T	T
0.2296674708627237	0.4081812169647593	0.5058684142729658	T	T	T
0.2231516446940026	0.6081141810853812	0.4989997326348843	T	T	T
0.2226559555270949	0.8072691429728290	0.4964734346265011	T	T	T
0.3340749578925817	0.0060517339860656	0.4981738948020412	T	T	T
0.3330109677498932	0.2046112400670117	0.5082349246126046	T	T	T
0.3346321913573122	0.4067918415203350	0.5097569811428604	T	T	T
0.3334577897851160	0.6092829607409636	0.5008139494634593	T	T	T
0.3340042324989043	0.8072767568532843	0.4958783238522197	T	T	T
0.4451327953845211	0.0061761321776889	0.4980178567578972	T	T	T
0.4461823511552557	0.2048110203503829	0.5063594981174983	T	T	T
0.4390979496064020	0.4075183895914873	0.5077812500817940	T	T	T
0.4465491891427484	0.6083265222612698	0.4978575307180853	T	T	T
0.4454048057738507	0.8073169516643610	0.4953809245878078	T	T	T

0.5563199581456730	0.0064007385021823	0.4967841538159234	T	T	T
0.5564007510170402	0.2055243254047403	0.5030748013184558	T	T	T
0.5637219758805863	0.4080230666534421	0.5019300508954712	T	T	T
0.5570011373938609	0.6089871697242099	0.4967095412726527	T	T	T
0.5564891988872934	0.8075566361986433	0.4949516668221184	T	T	T
0.6677623588093413	0.0067094671879051	0.4956383411937415	T	T	T
0.6680874262682200	0.2054024618206955	0.4990986714988024	T	T	T
0.6687222687660060	0.4077644623901808	0.5010377290406318	T	T	T
0.6681594613916854	0.6081437899003752	0.4971369930002570	T	T	T
0.6676850368341981	0.8075579296008188	0.4951901788689096	T	T	T
0.7791027286072694	0.0065621887083373	0.4951374289455969	T	T	T
0.7798031499533344	0.2063084758279549	0.4977996518110575	T	T	T
0.7797598516624381	0.4071185985220773	0.4999998576906172	T	T	T
0.7793005625914855	0.6075674351807969	0.4982132871301113	T	T	T
0.7788751200641604	0.8072171433182616	0.4959102636790283	T	T	T
0.8894105760449771	0.0064359112256673	0.4951880493603004	T	T	T
0.8893274267149429	0.2061644574172380	0.4966608506087954	T	T	T
0.8894019204393180	0.4069414443420898	0.5005210708678147	T	T	T
0.8895066717542148	0.6073590566529190	0.4997084531902553	T	T	T
0.8894533117894967	0.8070336251158399	0.4968003054071695	T	T	T
0.0003455869728102	0.0581040565394640	0.3969127055084807	T	T	T
0.9996927431867884	0.2584804174938859	0.3999027784349361	T	T	T
0.9999149688525314	0.4587741525838887	0.4044026035386908	T	T	T
0.0004341073433166	0.6583758648113121	0.4013970838309214	T	T	T
0.0003860585929878	0.8581371486516025	0.3979570751291402	T	T	T
0.1119110985765458	0.0588661296096011	0.3980979289190856	T	T	T
0.1132187234356976	0.2607084775437054	0.4052567615219770	T	T	T
0.1088568747441308	0.4566687232516028	0.4051784428631167	T	T	T
0.1111392749978481	0.6579985906885970	0.4009660373239659	T	T	T
0.1114796520778985	0.8582476268615404	0.3979854691946726	T	T	T
0.2234704976689222	0.0589266276749366	0.3998492110696478	T	T	T
0.2232624685646157	0.2602949317143813	0.4094847779043548	T	T	T
0.2229760561535235	0.4602850411846291	0.4112192558549362	T	T	T
0.2225601582052154	0.6588290191001233	0.3999603016531662	T	T	T
0.2226261137770616	0.8586870007847699	0.3975817674572382	T	T	T
0.3343498601590874	0.0590487603293748	0.4011342636472821	T	T	T
0.3343293237560971	0.2574195819193620	0.4119337209869121	T	T	T
0.3353249346856245	0.4553657351022111	0.4067937821848041	T	T	T
0.3336495789873949	0.6587668386515300	0.3999209859532890	T	T	T
0.3338387716229827	0.8588254608189574	0.3973418631204322	T	T	T
0.4445439253147532	0.0590876102697069	0.4009508158818794	T	T	T
0.4451268485266646	0.2595534226372943	0.4121333499928236	T	T	T
0.4449921803716557	0.4587518369707523	0.4112223657737885	T	T	T
0.4453869475389325	0.6588224805631333	0.3984921620583138	T	T	T
0.4451726204388753	0.8588434556121516	0.3967580528562237	T	T	T
0.5557413528970587	0.0589987728232740	0.3992531079832261	T	T	T
0.5551770399955261	0.2598665022522739	0.4081748687732358	T	T	T
0.5596284074627417	0.4572071615734670	0.4013628049663091	T	T	T
0.5566077547842553	0.6597742220822963	0.3973424272522025	T	T	T
0.5564376058564554	0.8592105992517935	0.3963922473152978	T	T	T
0.6674882911893736	0.0591110085546308	0.3978523645522280	T	T	T
0.6669864768907634	0.2589352892507149	0.4037179056904675	T	T	T
0.6692335330136882	0.4581376164799513	0.4011185466958951	T	T	T
0.6681837493729492	0.6592127694308193	0.3980146351031988	T	T	T

0.6677508404907290	0.8590346490023645	0.3965006191015997	T	T	T
0.7789199472062358	0.0583483319566219	0.3966669598669427	T	T	T
0.7786179885779071	0.2590260919457701	0.4004641972621827	T	T	T
0.7795746551089036	0.4588529743579425	0.4016790414780737	T	T	T
0.7792519827711563	0.6587023698080703	0.3991028119993414	T	T	T
0.7789705540884689	0.8584234675572729	0.3968483230490911	T	T	T
0.8894522646132269	0.0581159822115138	0.3965492318750380	T	T	T
0.8891278330433825	0.2589085718963644	0.3988987180888061	T	T	T
0.8901967855346475	0.4590607753899792	0.4026272311597818	T	T	T
0.8899082420453542	0.6582783065771266	0.4004597467121541	T	T	T
0.8896894135826479	0.8580179669502359	0.3974784236280565	T	T	T
0.0560315188279724	0.1572332226834025	0.4271026821559681	T	T	T
0.0545506315674756	0.3573647100827855	0.4312241761278304	T	T	T
0.0552184496762949	0.5570973426293736	0.4321155293722804	T	T	T
0.0560054966380796	0.7570999749117897	0.4275910687548974	T	T	T
0.0561213865625042	0.9568860810403892	0.4256946129867971	T	T	T
0.1680532784276364	0.1572368236794963	0.4288011190139678	T	T	T
0.1675762311672274	0.3593231989392230	0.4415619544954895	T	T	T
0.1653226490278624	0.5567072582222927	0.4319929736996527	T	T	T
0.1669278406030965	0.7572785822469512	0.4270432465645093	T	T	T
0.1673577750139388	0.9571918820764479	0.4261026599672593	T	T	T
0.2788101713237277	0.1570895631477048	0.4326185082433233	T	T	T
0.2809951969561950	0.3558811870261219	0.4373119251371991	T	T	T
0.2799253348961082	0.5571145215431369	0.4312257306078094	T	T	T
0.2780792900028225	0.7575394581635152	0.4262455749064122	T	T	T
0.2785637425864809	0.9572403448120578	0.4267080084003980	T	T	T
0.3893920535139503	0.1573886275091562	0.4348193176952572	T	T	T
0.3886202561054363	0.3548004231785759	0.4395444410621994	T	T	T
0.3886957049182087	0.5566407813009703	0.4331978553100521	T	T	T
0.3895834454639480	0.7576356959387863	0.4256565185433995	T	T	T
0.3895770882390200	0.9573240442083077	0.4266099183663907	T	T	T
0.5003407630236323	0.1571200354423063	0.4312069367152867	T	T	T
0.5002615057356248	0.3589987206860421	0.4414541490419926	T	T	T
0.5024443007227066	0.5571661477006287	0.4285822739714007	T	T	T
0.5008312222277927	0.7578318344164432	0.4250101928745846	T	T	T
0.5005153498237688	0.9575367663001407	0.4260867761058971	T	T	T
0.6112925251852717	0.1572034236759051	0.4292275114498168	T	T	T
0.6138205614248623	0.3560790354784061	0.4309334006940110	T	T	T
0.6132267614478051	0.5580632247721080	0.4275160774690540	T	T	T
0.6122223197428601	0.7581664388030103	0.4249082053916449	T	T	T
0.6118834826541314	0.9576152622344822	0.4253646073567344	T	T	T
0.7236237476226343	0.1572237622307573	0.4271636108579785	T	T	T
0.7236863493576402	0.3570167664721296	0.4310244184718995	T	T	T
0.7241351244428873	0.5575740551769675	0.4284363639104118	T	T	T
0.7234756376057909	0.7576761075367150	0.4256683734191423	T	T	T
0.7232595039100597	0.9572701520245330	0.4249456301147386	T	T	T
0.8341662153157413	0.1569361518024398	0.4255387659921618	T	T	T
0.8346318428798636	0.3573404429206649	0.4289907448224803	T	T	T
0.8347670455383529	0.5572295211646943	0.4290892057776967	T	T	T
0.8343727934334930	0.7570389317685977	0.4265845566039106	T	T	T
0.8342606717629125	0.9567034064361799	0.4247425655956332	T	T	T
0.9445667804908287	0.1567534101732205	0.4254925431972050	T	T	T
0.9448862484719762	0.3575152922282851	0.4299830577332001	T	T	T
0.9450611422765606	0.5571272611420836	0.4311229064609682	T	T	T

0.9449044379896975	0.7567459406619793	0.4274456243663952	T	T	T
0.9449648369776396	0.9567271225523688	0.4251077609771294	T	T	T
0.9996349172103351	0.1266549555675120	0.5568393799785805	T	T	T
0.9981358356306711	0.3264869879886012	0.5613753060878369	T	T	T
0.9991155192738083	0.5292891846389473	0.5640535313999746	T	T	T
0.0002459893849674	0.7286985801404638	0.5591473997071920	T	T	T
0.0002597666616690	0.9277024510896450	0.5566958635856729	T	T	T
0.1104613132192849	0.1259834659067167	0.5578171453845002	T	T	T
0.1057313878519951	0.3272152897833725	0.5605533930193355	T	T	T
0.1094350306148089	0.5292693767157201	0.5649623514132373	T	T	T
0.1117586756261992	0.7286359975624924	0.5588004055638537	T	T	T
0.1114880691431012	0.9276479245474177	0.5568068172128785	T	T	T
0.2222456623097645	0.1250084819442832	0.5610614977258892	T	T	T
0.2240018142561729	0.3293835698406107	0.5617794612379603	T	T	T
0.2213378342459112	0.5287074702934441	0.5546162330113151	T	T	T
0.2226628549419677	0.7288858664932165	0.5581632368566111	T	T	T
0.2227525187186076	0.9271394133201092	0.5572882520329138	T	T	T
0.3335199790773649	0.1244379776256601	0.5642585584150036	T	T	T
0.3331092223455521	0.3284235188796639	0.5723453234219700	T	T	T
0.3354603442751304	0.5325263794380021	0.5677850395927942	T	T	T
0.3342433839585385	0.7290190462363153	0.5571418637269754	T	T	T
0.3340658166220368	0.9268133460503425	0.5572599917528976	T	T	T
0.4458080242176767	0.1248563479733941	0.5632195365361879	T	T	T
0.4439401181107761	0.3288989127630031	0.5675772129084746	T	T	T
0.4470948242093002	0.5291908599612927	0.5560516493148739	T	T	T
0.4457545640913997	0.7288744337912170	0.5568253308217292	T	T	T
0.4453387236464678	0.9268524199515026	0.5571638173340413	T	T	T
0.5568829520741019	0.1252956296332569	0.5604219759188876	T	T	T
0.5590337635831096	0.3299805227658267	0.5597205656115590	T	T	T
0.5611011036649483	0.5315513535442014	0.5589601912520060	T	T	T
0.5567888609558138	0.7291647091241695	0.5563491436679697	T	T	T
0.5565398566544025	0.9272645352409454	0.5565348038735357	T	T	T
0.6685905924253855	0.1258763453375666	0.5578047405491132	T	T	T
0.6709674726671025	0.3285898659236274	0.5612187219824598	T	T	T
0.6697354248613001	0.5300915373226226	0.5603552793439283	T	T	T
0.6677406667584117	0.7289651276266768	0.5563335148599265	T	T	T
0.6677592133957048	0.9276424898342542	0.5557855201460565	T	T	T
0.7795226745588885	0.1268059461438712	0.5571082475253368	T	T	T
0.7803523257861414	0.3276688147871005	0.5604581361357573	T	T	T
0.7793987809732726	0.5291653207396826	0.5605388631151657	T	T	T
0.7785914198166315	0.7286804860227040	0.5576379775374305	T	T	T
0.7787071289632761	0.9278287084454411	0.5562717855624904	T	T	T
0.8895611330196402	0.1270705445025360	0.5568891263039630	T	T	T
0.8893125847212786	0.3274785598689621	0.5606699784221598	T	T	T
0.8889698197058844	0.5288942068868875	0.5621627739773279	T	T	T
0.8892333651382656	0.7287012088218419	0.5589949389107305	T	T	T
0.8893558850851991	0.9278038388290162	0.5566170735755914	T	T	T
0.0555277102082649	0.0257378274974381	0.5845576211060177	T	T	T
0.0546258102384565	0.2238218576929652	0.5872333511516561	T	T	T
0.0525830474446979	0.4280394913312774	0.5948449027314918	T	T	T
0.0555342356387225	0.6281115294341703	0.5896882888486494	T	T	T
0.0558810044259509	0.8267800640606606	0.5856859227011663	T	T	T
0.1667309777286247	0.0250823939128723	0.5858478997999589	T	T	T
0.1649563951938410	0.2174768351048528	0.5930218770882455	T	T	T

0.1678090132978111	0.4241983070932629	0.5879778108999641	T	T	T
0.1673857265336500	0.6279573762503021	0.5882371870132239	T	T	T
0.1672009939606551	0.8269502366536602	0.5856675805582970	T	T	T
0.2781546484012103	0.0240940282613677	0.5871614486205974	T	T	T
0.2764007905680508	0.2206037589794878	0.5973601555212031	T	T	T
0.2797876264132175	0.4282694933254341	0.5974665553969878	T	T	T
0.2776906238628374	0.6284120094186603	0.5886337872707614	T	T	T
0.2785315731617864	0.8263512431575168	0.5851471785547959	T	T	T
0.3896444416135151	0.0239001446971645	0.5878518130829093	T	T	T
0.3902457957711387	0.2191075803161320	0.5981546218977059	T	T	T
0.3895172692637359	0.4296723263841429	0.5999962630138972	T	T	T
0.3910692635442756	0.6292020032090316	0.5870657395912453	T	T	T
0.3898962392043295	0.8264806230261694	0.5845907676454215	T	T	T
0.5011823797729394	0.0243392805638234	0.5868151060214496	T	T	T
0.5025263544813459	0.2199494378609951	0.5960043760092596	T	T	T
0.5031833665982031	0.4245763946390232	0.5854462282902383	T	T	T
0.5021362673772812	0.6279493609089992	0.5862828533645768	T	T	T
0.5010214216130904	0.8266599720595060	0.5842205510180696	T	T	T
0.6125014111457437	0.0250487773693987	0.5853829981357870	T	T	T
0.6142945685528820	0.2193742078113471	0.5917326241785945	T	T	T
0.6159881100487984	0.4303830103433841	0.5918167556667272	T	T	T
0.6128617291435942	0.6288759109833203	0.5855100977191532	T	T	T
0.6120571564481392	0.8270681087824180	0.5839342990067191	T	T	T
0.7236326514210966	0.0256525707786431	0.5842903219883042	T	T	T
0.7244657776831266	0.2244806127110205	0.5877169945676329	T	T	T
0.7248598709689771	0.4278494897008889	0.5892270845920035	T	T	T
0.7235326231212599	0.6280663735807333	0.5862584870493810	T	T	T
0.7230775106141768	0.8270822024671527	0.5843714262605786	T	T	T
0.8342250573200836	0.0259247158185440	0.5844492900034810	T	T	T
0.8349036212851089	0.2254996968390787	0.5869731663495370	T	T	T
0.8342878472517126	0.4268632229817990	0.5896261676173969	T	T	T
0.8339402232531491	0.6276527982551402	0.5880115367735321	T	T	T
0.8338464543496334	0.8269845267268505	0.5853485631142684	T	T	T
0.9447336550046999	0.0258629606711380	0.5843404650976389	T	T	T
0.9442943247259036	0.2251132816149198	0.5866087987756531	T	T	T
0.9433834599467398	0.4264336822372178	0.5910803793185252	T	T	T
0.9443757881305158	0.6277196370736666	0.5890714233476672	T	T	T
0.9448075454409197	0.8268334012113053	0.5858501332389533	T	T	T
0.2104534943482910	0.2785826493271482	0.7265323591156511	T	T	T
0.3298554348382052	0.2776009600087158	0.7267299878809175	T	T	T
0.4523552105783474	0.2854663353969188	0.7300161349449159	T	T	T
0.5634707696729766	0.2723860932133232	0.7233447780830766	T	T	T
0.1482401744989560	0.3434888407936870	0.7304000260723872	T	T	T
0.2595323142579482	0.3763877232454494	0.7277699973699511	T	T	T
0.3761991578648107	0.3566095914826115	0.7650844014307735	T	T	T
0.4888416062227803	0.3754211658817098	0.7111457333751687	T	T	T
0.6112581574206778	0.3451794381033724	0.7433981657632882	T	T	T
0.2131511391032225	0.4965361825189623	0.7213959309933504	T	T	T
0.2979765318577254	0.4953337636221927	0.7545887287233857	T	T	T
0.4491140824440656	0.4975439373222344	0.7248811383029076	T	T	T
0.5359683770526065	0.4861299586471010	0.7435692481966413	T	T	T
0.1031376503831740	0.4360980400358591	0.7379563650072867	T	T	T
0.6320650527048929	0.4451440347454774	0.7506126056920839	T	T	T
0.1658551873258197	0.4324772059309501	0.8221287409065567	T	T	T

```
  0.3877424622987454  0.4437191141993639  0.8226492655100456   T   T   T
  0.6011662316770841  0.4187441352528146  0.8587587655774305   T   T   T
  0.1616041773515920  0.2972227161708750  0.6337502082241436   T   T   T
  0.2718715377410066  0.3083046950295543  0.6539093040719507   T   T   T
  0.3905473905324076  0.3033687952490335  0.6529958319117916   T   T   T
  0.5071855173326129  0.3048737978521039  0.6448475636584491   T   T   T
  0.6159122463579899  0.2988944334435946  0.6391389882746910   T   T   T
  0.1861544895289844  0.4181427778420266  0.7080316699079773   T   T   T
  0.3240693106420214  0.4191494806674116  0.7556944331744541   T   T   T
  0.4187006750041498  0.4212874482237280  0.7160535179132000   T   T   T
  0.5569189349882052  0.4073734516357674  0.7498396399366674   T   T   T
  0.2727065202665001  0.5083721447678182  0.6412428917303470   T   T   T
  0.5033556692177977  0.5034760563751897  0.6355091909384052   T   T   T
== On-top cluster Pd16-Te21
   1.00000000000000
     31.7040281100000030    0.0000000000000000    0.0000000000000000
      0.0000000000000000   31.5721201999999970    0.0000000000000000
      0.0000000000000000    0.0000000000000000   22.0000000000000000
   W    Te   Pd
    90   201    16
Selective dynamics
Direct
  0.0554912133200615  0.0765732186358627  0.4849611971467238   T   T   T
  0.0546742743542630  0.2764777787751429  0.4901214559019105   T   T   T
  0.0555922370473634  0.4784069666451570  0.4941356482570102   T   T   T
  0.0558066842479469  0.6783685370451342  0.4899115246792592   T   T   T
  0.0557091376897937  0.8774865790354718  0.4858593664719602   T   T   T
  0.1668571084203888  0.0769472216927871  0.4864109076316150   T   T   T
  0.1650265569752563  0.2757995120684341  0.4925921559084706   T   T   T
  0.1649553337409053  0.4794259323558764  0.4950904269602559   T   T   T
  0.1673605354517960  0.6787517878461421  0.4896210098143780   T   T   T
  0.1670055447100884  0.8780088700769659  0.4860567299233525   T   T   T
  0.2781648560238507  0.0773452476809148  0.4887920173928448   T   T   T
  0.2767639974132349  0.2766537802060117  0.4967978829768318   T   T   T
  0.2727431666772702  0.4781003143086843  0.4922542910499439   T   T   T
  0.2779024977943039  0.6794634902163756  0.4887814754964837   T   T   T
  0.2781071486428958  0.8783919536434862  0.4859678454676652   T   T   T
  0.3891391995821317  0.0776906439755932  0.4905693878674701   T   T   T
  0.3883257775715784  0.2752539325071061  0.4957536148052017   T   T   T
  0.3916051138862569  0.4791119992155601  0.4922182539343125   T   T   T
  0.3892103467921370  0.6794112453496359  0.4861464890516764   T   T   T
  0.3890135358596601  0.8784143569682352  0.4858883830656597   T   T   T
  0.5002239714147739  0.0771295815847635  0.4908918043424420   T   T   T
  0.4996787735996041  0.2765732193330745  0.5029508695647722   T   T   T
  0.4962787742494097  0.4778677288317927  0.4869395474904096   T   T   T
  0.5004487616718651  0.6796093761458435  0.4852860554731875   T   T   T
  0.5002838857277219  0.8784152939347001  0.4858557026870428   T   T   T
  0.6117310880126779  0.0772021501637096  0.4902466478577958   T   T   T
  0.6138222710877405  0.2747555470901572  0.4995287444118138   T   T   T
  0.6148427813322959  0.4785634909215044  0.4928400317394766   T   T   T
  0.6114804224968675  0.6790828242492322  0.4846705431764547   T   T   T
  0.6115344954191286  0.8782458363155989  0.4854046130775120   T   T   T
  0.7232326826360875  0.0768296425925677  0.4881832159627549   T   T   T
  0.7243056186719113  0.2764416626984832  0.4991550677737822   T   T   T
```

0.7196945143763346	0.4776441422030372	0.4881670045753104	T	T	T
0.7227992250791447	0.6792442835660784	0.4866391109773703	T	T	T
0.7226912660321040	0.8779854538285694	0.4853320351704462	T	T	T
0.8341915481226542	0.0766960742680387	0.4864912368717718	T	T	T
0.8362502693269500	0.2760704443980819	0.4953899185791219	T	T	T
0.8389959867115639	0.4791842159926435	0.4938800746766590	T	T	T
0.8335588249106379	0.6784636457110972	0.4882010687389731	T	T	T
0.8338416526330967	0.8776486113253988	0.4854442395460605	T	T	T
0.9447880939849089	0.0766244523388887	0.4851368312567531	T	T	T
0.9459427530945690	0.2763280495202254	0.4905909961300925	T	T	T
0.9455988534227884	0.4785199111556878	0.4937056916877892	T	T	T
0.9445687933537450	0.6784435751153873	0.4896328758108039	T	T	T
0.9445859106601971	0.8774260924960470	0.4856213622752261	T	T	T
0.9999306412536683	0.0055781569884555	0.4942590937468316	T	T	T
0.0003722801160022	0.2050408248738449	0.4969141854832274	T	T	T
0.0000865728934013	0.4064850075786124	0.5021881603518953	T	T	T
0.0000031845046927	0.6074315199230245	0.5011417540745847	T	T	T
0.0000274812334640	0.8066495313063013	0.4963319775606373	T	T	T
0.1111264648560810	0.0060143994127297	0.4949171596284104	T	T	T
0.1109541588557245	0.2047676901708345	0.4970834748971221	T	T	T
0.1111923091592410	0.4072988799438374	0.5032207919996707	T	T	T
0.1111412017596726	0.6076945645893201	0.5014764162717957	T	T	T
0.1113835617013505	0.8069195203907226	0.4964928621243220	T	T	T
0.2224809812073972	0.0064019712776262	0.4963044918278047	T	T	T
0.2211675109584668	0.2054156059393132	0.4999533942430235	T	T	T
0.2143357868032569	0.4076865551916432	0.5007778804471330	T	T	T
0.2228339944680573	0.6084451493822078	0.5009172189799130	T	T	T
0.2223746517589228	0.8075311767795281	0.4961474170398555	T	T	T
0.3335625410648256	0.0065808692252784	0.4976375550433411	T	T	T
0.3318102260983672	0.2056227974839261	0.5038853651128617	T	T	T
0.3400544977019410	0.4071671052505804	0.5039092245596375	T	T	T
0.3320942827576056	0.6087501182706567	0.4979113854115951	T	T	T
0.3335013228572713	0.8075421396191064	0.4952526283667402	T	T	T
0.4446350828809296	0.0064687621986540	0.4983022130104069	T	T	T
0.4456012988609878	0.2052769356453631	0.5052055861100914	T	T	T
0.4396839039259353	0.4076744477076820	0.5021053538109443	T	T	T
0.4455719047512286	0.6092815060421840	0.4958420086756981	T	T	T
0.4446968607362177	0.8076733580748118	0.4948778543105914	T	T	T
0.5560650976942445	0.0063081302144684	0.4979883468641909	T	T	T
0.5547233605055256	0.2046822555703763	0.5068412592566411	T	T	T
0.5634997764950175	0.4080627655811333	0.5050994053918293	T	T	T
0.5549789184210625	0.6082912825744020	0.4942330223213605	T	T	T
0.5560115538243620	0.8075114318114344	0.4945053871879501	T	T	T
0.6674702377484371	0.0061490867050582	0.4969651219268302	T	T	T
0.6697309988325013	0.2045242639756631	0.5043733479632692	T	T	T
0.6632473255253262	0.4074912597453286	0.5040573902789676	T	T	T
0.6683218877999734	0.6088918211748415	0.4962657780686159	T	T	T
0.6670403354376084	0.8073283609314048	0.4945554068083809	T	T	T
0.7786929188260497	0.0059069542975843	0.4955700361982789	T	T	T
0.7803520957610339	0.2050484378317953	0.5014461520966901	T	T	T
0.7875931770941648	0.4083708786687262	0.5020544866039602	T	T	T
0.7775164653801421	0.6078950203369144	0.4972864898754846	T	T	T
0.7783922634477946	0.8071028675185837	0.4953169429421815	T	T	T
0.8893224922321690	0.0057364724384588	0.4946231692987354	T	T	T

0.8898382901069992	0.2047266654015759	0.4984379420357030	T	T	T
0.8914668390804407	0.4069965775696660	0.5029727059789847	T	T	T
0.8896872910647161	0.6077033526408196	0.5003666615456974	T	T	T
0.8891573875750891	0.8066841229032653	0.4959111557086086	T	T	T
0.0001725693104385	0.0573596446210990	0.3958503668451638	T	T	T
0.0002054752392860	0.2578378109769238	0.3997994121783769	T	T	T
0.0007135374761313	0.4588289574597991	0.4045148736044274	T	T	T
0.0003499876101743	0.6579323432947172	0.4013904165324635	T	T	T
0.0003113394386957	0.8575917134449864	0.3969628066122063	T	T	T
0.1117198469677926	0.0579577275047891	0.3965423920985571	T	T	T
0.1111194655593107	0.2581872954367394	0.4012715505017750	T	T	T
0.1105415294798120	0.4590949898190085	0.4050395615352267	T	T	T
0.1114648852369425	0.6578672045503854	0.4015281927933593	T	T	T
0.1114576152274019	0.8578869276811135	0.3972903757138840	T	T	T
0.2233292540380103	0.0586234243731251	0.3984042646282532	T	T	T
0.2227677317830969	0.2596736086864396	0.4049580739649568	T	T	T
0.2188057815301515	0.4588608000804298	0.4029860355593811	T	T	T
0.2223363177447810	0.6583707038292252	0.4006354066472134	T	T	T
0.2224704175251016	0.8586758955766790	0.3970715627024952	T	T	T
0.3343050601113319	0.0593944297750736	0.4002880506166990	T	T	T
0.3325292646803978	0.2582606826484742	0.4072915364434401	T	T	T
0.3322639239506289	0.4582450695376086	0.4072494817710447	T	T	T
0.3327203067519146	0.6593133224063024	0.3988255358493958	T	T	T
0.3335333491453912	0.8591113206797650	0.3966801753491019	T	T	T
0.4446760711316103	0.0595043287843634	0.4012976995022061	T	T	T
0.4464803912126727	0.2591187528437054	0.4098886968424733	T	T	T
0.4423714957653037	0.4555961260486324	0.3992240139370460	T	T	T
0.4444996473777930	0.6601491866731599	0.3966678860517288	T	T	T
0.4446299732273148	0.8596154126795088	0.3966890129571212	T	T	T
0.5557798849862159	0.0594232581303906	0.4011318450353917	T	T	T
0.5555115472155506	0.2589892699856459	0.4130157952935562	T	T	T
0.5568942175115846	0.4567682806869127	0.4053975184369833	T	T	T
0.5557412069146596	0.6597199757004346	0.3959316351118837	T	T	T
0.5557360522202434	0.8594987167073250	0.3964363970813441	T	T	T
0.6667314425506798	0.0591376130358277	0.3999148622900227	T	T	T
0.6687123979872954	0.2588486667905435	0.4094333775027214	T	T	T
0.6660129358748915	0.4548036224917513	0.4005267233741224	T	T	T
0.6677191490918781	0.6594445396531922	0.3968160710716152	T	T	T
0.6670651547520818	0.8590530305648678	0.3962207412917486	T	T	T
0.7780629100512958	0.0583963095326983	0.3979170870000603	T	T	T
0.7785105444045232	0.2600983316524812	0.4076142017647436	T	T	T
0.7807701021000502	0.4589712906493364	0.4053229072774012	T	T	T
0.7786840507570298	0.6588697504842649	0.3984934552366696	T	T	T
0.7782749593866252	0.8583799848468664	0.3963691471014653	T	T	T
0.8890913521643654	0.0578157287722652	0.3963544469356725	T	T	T
0.8890155392441678	0.2583766339430381	0.4026855844371715	T	T	T
0.8913813327521354	0.4581655257522526	0.4033966708645348	T	T	T
0.8896542276267351	0.6581654079761927	0.4004414888717350	T	T	T
0.8892760388662706	0.8575911133082865	0.3965483372304001	T	T	T
0.0556171723875226	0.1561180068077822	0.4250370023172551	T	T	T
0.0559388009739015	0.3571573687045455	0.4319616465344905	T	T	T
0.0555981059071191	0.5572364569303769	0.4325886810230734	T	T	T
0.0558334761384029	0.7566544070466106	0.4272505508488504	T	T	T
0.0559034913737234	0.9562346275337948	0.4244729149573419	T	T	T

0.1672098766417721	0.1563215173927429	0.4264806887563811	T	T	T
0.1643420633435777	0.3560685974590992	0.4296062772444632	T	T	T
0.1664107364499714	0.5575970851471480	0.4328251350943619	T	T	T
0.1669148907394047	0.7570336994700732	0.4269829731335299	T	T	T
0.1671822328109518	0.9568758436786853	0.4252097490094499	T	T	T
0.2781434773704323	0.1569441572157094	0.4294743091679754	T	T	T
0.2780826883549672	0.3595427343201497	0.4400291128114851	T	T	T
0.2759317198699056	0.5570530416991168	0.4304472703466015	T	T	T
0.2776199564317555	0.7576705242817684	0.4259505201087813	T	T	T
0.2784507005540393	0.9575537053112322	0.4259068598040448	T	T	T
0.3894680835251502	0.1576985827483189	0.4324998728294539	T	T	T
0.3884908806763046	0.3556220765339236	0.4300825449705759	T	T	T
0.3887314151491276	0.5581297966426125	0.4293553479817498	T	T	T
0.3890246562050999	0.7581655213205752	0.4247573132437357	T	T	T
0.3892672276583520	0.9578413713592798	0.4264936439738760	T	T	T
0.5004448718544046	0.1570124239830883	0.4321258670267732	T	T	T
0.5016076728616164	0.3582141735486765	0.4436456457022335	T	T	T
0.4992731309990344	0.5573521243836778	0.4250998088046053	T	T	T
0.5001873366688584	0.7584357321966259	0.4241633359367765	T	T	T
0.5002822765513500	0.9578999199242192	0.4267258309137107	T	T	T
0.6115692062058740	0.1576115039311257	0.4334929145934423	T	T	T
0.6128663237272645	0.3545529864995185	0.4339032362455935	T	T	T
0.6127887643797578	0.5571575779862189	0.4279838798012339	T	T	T
0.6114795384514173	0.7581343926165461	0.4237977913658661	T	T	T
0.6114477169546644	0.9576685559510676	0.4259937038055512	T	T	T
0.7234502866283614	0.1569337997101750	0.4294773322737986	T	T	T
0.7241392002822588	0.3590058792509619	0.4402908207225738	T	T	T
0.7225455751245137	0.5570293247440744	0.4265261920053542	T	T	T
0.7229752263082896	0.7577916501094153	0.4248329525309946	T	T	T
0.7225394616358867	0.9571086668569150	0.4251800060831772	T	T	T
0.8338962021754743	0.1565065875558951	0.4273127845279135	T	T	T
0.8369202847254392	0.3559266001362261	0.4320983050754156	T	T	T
0.8350964967184070	0.5572099310486472	0.4306413420431944	T	T	T
0.8338853680074469	0.7568534917272720	0.4259792914542496	T	T	T
0.8336868394515883	0.9564105403482548	0.4244575453737651	T	T	T
0.9448313595247714	0.1562399659558334	0.4255377215052918	T	T	T
0.9454115485111240	0.3566318944485692	0.4309520782996637	T	T	T
0.9451995406104354	0.5571361566933852	0.4317787619587068	T	T	T
0.9448453637629157	0.7565764263818843	0.4267840751800953	T	T	T
0.9444632282891011	0.9561346949750976	0.4239667365263117	T	T	T
0.0003115537227119	0.1257617531990909	0.5561083764946856	T	T	T
0.0005705454677369	0.3261460591306801	0.5620063030235167	T	T	T
0.0002701363403699	0.5291177318602058	0.5641330058582710	T	T	T
0.0000745025371063	0.7285794106690113	0.5588609040856888	T	T	T
0.0000246942224622	0.9270986242875089	0.5557338055106287	T	T	T
0.1103354707161852	0.1254654566949042	0.5564815276572213	T	T	T
0.1083261246212249	0.3274153616117699	0.5612489809719609	T	T	T
0.1099478469407976	0.5294334431335422	0.5649141566799500	T	T	T
0.1113304538869461	0.7286796890023159	0.5587256863968552	T	T	T
0.1110031649386187	0.9272916289639217	0.5559536394558440	T	T	T
0.2213769022465754	0.1256084225393356	0.5588073487166143	T	T	T
0.2188319436887016	0.3293899564919138	0.5557950405421136	T	T	T
0.2206309079331922	0.5301244780837822	0.5652802290956794	T	T	T
0.2225943861897670	0.7293320883916048	0.5583615380266773	T	T	T

0.2222441835445390	0.9274439701583580	0.5564996427348560	T	T	T
0.3328132895228814	0.1256248890871326	0.5616275951606735	T	T	T
0.3340732439470776	0.3285547002728044	0.5641241600945194	T	T	T
0.3332611551560867	0.5290988806496031	0.5538025812352997	T	T	T
0.3339864205429766	0.7292590704806432	0.5569190150268247	T	T	T
0.3335648121257499	0.9272759807395043	0.5569345446941296	T	T	T
0.4446707324334435	0.1254194250547745	0.5631848457878555	T	T	T
0.4396425680292286	0.3301867022571046	0.5628088068188194	T	T	T
0.4452259683137850	0.5317378024845342	0.5578757025830194	T	T	T
0.4450094164564642	0.7291077155517380	0.5556383131685450	T	T	T
0.4447692037754753	0.9270432018640159	0.5571678720515311	T	T	T
0.5560127982269588	0.1246386703522716	0.5632926209306266	T	T	T
0.5592272754435523	0.3297563460795661	0.5650888718975302	T	T	T
0.5556973805536968	0.5296045170818303	0.5525741009424441	T	T	T
0.5560679955080439	0.7288498944306767	0.5551922055338825	T	T	T
0.5561612149670311	0.9269969050089487	0.5568831013499960	T	T	T
0.6684977859310576	0.1245764394034633	0.5616226986585384	T	T	T
0.6675924394635366	0.3291924976317104	0.5659742466924729	T	T	T
0.6678021064610986	0.5311727833731531	0.5587404866623843	T	T	T
0.6666908857566864	0.7288018990440710	0.5554347790932164	T	T	T
0.6672888875277573	0.9269553152766898	0.5564312817629371	T	T	T
0.7795549421283774	0.1248901786457092	0.5591468696462913	T	T	T
0.7812485378976450	0.3303204654374537	0.5578066231027577	T	T	T
0.7784129588734445	0.5288353538913573	0.5555243641495421	T	T	T
0.7779567611360582	0.7287238395918467	0.5571544450617917	T	T	T
0.7784051356408869	0.9269977252015269	0.5558112870468209	T	T	T
0.8902741538297450	0.1253280934649746	0.5572400512765051	T	T	T
0.8940252098565877	0.3279837309865148	0.5629969510391128	T	T	T
0.8914155554653624	0.5297572933966911	0.5651207033671215	T	T	T
0.8887846715400907	0.7285350855980529	0.5582713802968222	T	T	T
0.8892676701447416	0.9271009614467071	0.5557945916424636	T	T	T
0.0552332578624798	0.0250501289213457	0.5835519307887099	T	T	T
0.0552988572383873	0.2237242415979092	0.5866900905510567	T	T	T
0.0551128209293016	0.4262604390880943	0.5917445900864676	T	T	T
0.0554295490569996	0.6281706223010177	0.5898198336857651	T	T	T
0.0555803317709811	0.8266256128172915	0.5850640361080872	T	T	T
0.1662049728128707	0.0251481743114498	0.5846357678292162	T	T	T
0.1645829880137151	0.2192134204778567	0.5895318452675072	T	T	T
0.1647938772063091	0.4284493229703912	0.5953506291702000	T	T	T
0.1668568397696787	0.6285666427968372	0.5894186466133872	T	T	T
0.1668097259205805	0.8270522146817770	0.5851699010040274	T	T	T
0.2775525932787235	0.0247960665810250	0.5864119525568139	T	T	T
0.2750069429835584	0.2205377026695328	0.5930801007740163	T	T	T
0.2757735176544283	0.4223507066953497	0.5837478835699375	T	T	T
0.2784609684843379	0.6288391387720532	0.5887561931279029	T	T	T
0.2781731249730397	0.8269811715801654	0.5845625639249452	T	T	T
0.3888723221435005	0.0246809523450125	0.5875232524477662	T	T	T
0.3882222506774106	0.2219674923065157	0.5945778149005548	T	T	T
0.3907418248166350	0.4320169610418728	0.5942864737740831	T	T	T
0.3894296940012703	0.6283667716339554	0.5847439900787385	T	T	T
0.3892101144940065	0.8266988310244302	0.5841458189218419	T	T	T
0.5005095961854285	0.0240796964975823	0.5877634023965100	T	T	T
0.4999059195582485	0.2200515661435878	0.5975971094793956	T	T	T
0.5009791313153550	0.4273388117556953	0.5841213920826686	T	T	T

0.5009976153255795	0.6281922187127036	0.5845031533423708	T	T	T
0.5004986983602043	0.8264408518535188	0.5838635522198389	T	T	T
0.6121353410002398	0.0240255515542170	0.5868519702622630	T	T	T
0.6130347685598679	0.2180681374193725	0.5953799748419832	T	T	T
0.6139577654069474	0.4318052984106926	0.5978018347516122	T	T	T
0.6109791246788701	0.6280574113507255	0.5831905899345603	T	T	T
0.6115129549906214	0.8266633776785244	0.5836767649243333	T	T	T
0.7235822489973275	0.0241543436519054	0.5856180267740686	T	T	T
0.7258901112592351	0.2188645665477621	0.5941111422963262	T	T	T
0.7260952819104902	0.4260114945328987	0.5837764553090398	T	T	T
0.7226514438106922	0.6281916221264789	0.5862403501933410	T	T	T
0.7224938484457358	0.8264679064085709	0.5838524223806411	T	T	T
0.8343530254680502	0.0245684254934727	0.5844688761228516	T	T	T
0.8365369127981027	0.2193803195463908	0.5916818869148108	T	T	T
0.8385152380740127	0.4294145004961077	0.5942244615384205	T	T	T
0.8328811262585526	0.6273017738039149	0.5872712487180789	T	T	T
0.8335960273361462	0.8266061411897186	0.5846020680980953	T	T	T
0.9448748226895056	0.0249815481273465	0.5836205198515730	T	T	T
0.9459539072479470	0.2236455789194671	0.5874783517422055	T	T	T
0.9464216374079354	0.4270368079749338	0.5923196013139426	T	T	T
0.9444947782314992	0.6282202743877974	0.5896216165645467	T	T	T
0.9444949815054132	0.8265842790847696	0.5849800527238348	T	T	T
0.2125866773860454	0.2716324988234494	0.7299373456838597	T	T	T
0.3229236309209209	0.2785386728875466	0.7269668805017137	T	T	T
0.4491350276932615	0.2941875075632818	0.7343325586000289	T	T	T
0.5499211304543536	0.2729145926510475	0.7193721128993358	T	T	T
0.6738450108942508	0.2679251118051024	0.7185285780971594	T	T	T
0.7853921245868245	0.2756913989327863	0.7275750125421276	T	T	T
0.1433326381019271	0.3274118538127075	0.7455747627464050	T	T	T
0.2681775298490313	0.3586009216103704	0.7363644570214888	T	T	T
0.3765376849282721	0.3619472338265297	0.7449242973969973	T	T	T
0.4845526406125805	0.3807093008948529	0.7166797209652482	T	T	T
0.6032765790673722	0.3451815362999988	0.7555307503714824	T	T	T
0.7129065740246743	0.3539196004899359	0.7405418906529811	T	T	T
0.8479422908495042	0.3380967285531068	0.7440384844636075	T	T	T
0.1841767675251294	0.4024462349107165	0.7741143119919763	T	T	T
0.2686719908331607	0.4875637297268556	0.7584329518583619	T	T	T
0.3525114302037121	0.4986081403252351	0.7143010217894805	T	T	T
0.4575116693690929	0.5004430750242004	0.7370255478993432	T	T	T
0.5447245670374643	0.4891513111687648	0.7363935534324277	T	T	T
0.6687185307254161	0.4862512763664124	0.7272732826996161	T	T	T
0.7532060994820874	0.5058044269519272	0.7525832212663479	T	T	T
0.7992943756455277	0.4089240947673747	0.7733908148609507	T	T	T
0.1656856360561214	0.2988927328974643	0.6388679708480003	T	T	T
0.2690451079339120	0.3031674380005920	0.6416968149154717	T	T	T
0.3860268201191261	0.3057588363403587	0.6529520231500673	T	T	T
0.4977292208263447	0.3072193685771222	0.6393149218272127	T	T	T
0.6127339063035995	0.3007952674686618	0.6484850261247470	T	T	T
0.7296183289405853	0.3029586506985997	0.6427468551841792	T	T	T
0.8356998788630831	0.2999802820485206	0.6384373939333260	T	T	T
0.2344186261122470	0.4294813912175050	0.6896586996060831	T	T	T
0.3224125156749500	0.4221623406671919	0.7296648235730108	T	T	T
0.4168564834585666	0.4304078144227417	0.7138784679361767	T	T	T
0.5608841876851990	0.4109828040543255	0.7273768741525742	T	T	T

```
  0.6489142202250758  0.4063423036109570  0.7160781823239175   T   T   T
  0.7353521713686650  0.4296360835905652  0.7071673494886241   T   T   T
  0.2848568085972677  0.5003254933688109  0.6399974073521482   T   T   T
  0.5023012775636462  0.5106452428677338  0.6388512478339748   T   T   T
  0.7234529085351397  0.5089050241975991  0.6411214079891411   T   T   T
== On-top cluster Pd16-Te25
    1.0000000000000000
    31.7040281099999994    0.0000000000000000    0.0000000000000000
     0.0000000000000000   31.5721202000000005    0.0000000000000000
     0.0000000000000000    0.0000000000000000   30.0000000000000000
   W    Te   Pd
    90   205    16
Selective dynamics
Direct
  0.0554990292949259  0.0764878616300088  0.3559307328009556   T   T   T
  0.0547106534536538  0.2754259881253587  0.3607552307015144   T   T   T
  0.0544882176516464  0.4783955090000043  0.3660592658261804   T   T   T
  0.0560025511654239  0.6781668756607544  0.3600672140441284   T   T   T
  0.0557679849528831  0.8773751378574786  0.3563597092930014   T   T   T
  0.1667272637387541  0.0767045897818448  0.3570907600141865   T   T   T
  0.1667804335421602  0.2760149917159537  0.3654876293811031   T   T   T
  0.1619964085001832  0.4777145774852505  0.3653702819681536   T   T   T
  0.1671055569273417  0.6783147899135755  0.3590984705685681   T   T   T
  0.1667645488798092  0.8776320039741278  0.3563166210800955   T   T   T
  0.2781189450844649  0.0767011547806704  0.3581775844357192   T   T   T
  0.2786496091571961  0.2748933042331547  0.3642722685465825   T   T   T
  0.2826547827604999  0.4786558508916090  0.3627620564376817   T   T   T
  0.2781977980020435  0.6791812672467373  0.3581065985267406   T   T   T
  0.2779391053951557  0.8778904332339007  0.3560490743314951   T   T   T
  0.3889329094250223  0.0769903873907715  0.3586504324582758   T   T   T
  0.3883447918755070  0.2749875707844465  0.3650332896404830   T   T   T
  0.3851288841308605  0.4788020502323581  0.3633730775488034   T   T   T
  0.3895824914143389  0.6792159394648480  0.3559903317434002   T   T   T
  0.3891989059705416  0.8781715411232508  0.3556370696072892   T   T   T
  0.5002565163841145  0.0773724104769461  0.3586243455072102   T   T   T
  0.4990843234176594  0.2766510597873860  0.3663938176656391   T   T   T
  0.5070661424018883  0.4794770584594238  0.3588484205006192   T   T   T
  0.5005574067765503  0.6795882100996864  0.3554829621240406   T   T   T
  0.5001021637690747  0.8784863680147882  0.3554492112683781   T   T   T
  0.6111605745681510  0.0771464001713849  0.3584414393102927   T   T   T
  0.6123566155734329  0.2753390845536498  0.3640159864808492   T   T   T
  0.6137038877651539  0.4794809404185157  0.3604840854930658   T   T   T
  0.6116250966860851  0.6798224824316338  0.3555049936667869   T   T   T
  0.6111705351112650  0.8782334962897106  0.3552179710749335   T   T   T
  0.7220312597324702  0.0768865844706901  0.3577968516413559   T   T   T
  0.7212673509123008  0.2755935910453572  0.3643977552076085   T   T   T
  0.7185861559278476  0.4792822984566944  0.3602539718902145   T   T   T
  0.7229902340711483  0.6795010949467746  0.3570637076746052   T   T   T
  0.7226211018784277  0.8780819991438705  0.3557093325643641   T   T   T
  0.8333501308575377  0.0771504730514064  0.3568565240909669   T   T   T
  0.8336555973552158  0.2762602007932505  0.3654303124583483   T   T   T
  0.8379860158341510  0.4777373756206698  0.3639140290081581   T   T   T
  0.8336914250299907  0.6785827573964367  0.3583291246095499   T   T   T
  0.8337242629534846  0.8779287299549859  0.3562717469663441   T   T   T
```

0.9447070785385451	0.0765276488736529	0.3558405286771391	T	T	T
0.9453857562345804	0.2756225299632715	0.3608932836208041	T	T	T
0.9460745730109836	0.4784905900120456	0.3656938543212807	T	T	T
0.9445691778614992	0.6782047799863798	0.3599133475746872	T	T	T
0.9445969161434407	0.8775021739046055	0.3563769164072250	T	T	T
0.0001445775255252	0.0056460237866656	0.3625398955176226	T	T	T
0.9995183308513522	0.2044632435776458	0.3655331390802871	T	T	T
0.0002962161569625	0.4064345135365010	0.3725915641863236	T	T	T
0.0003342292560221	0.6072569679910753	0.3690205100218510	T	T	T
0.0001831873733417	0.8065620827306109	0.3642925206844722	T	T	T
0.1113123115604610	0.0057795712542171	0.3629370204125755	T	T	T
0.1119478507193478	0.2047380023337841	0.3657520203681103	T	T	T
0.1067898539958131	0.4062276691349353	0.3727332065709288	T	T	T
0.1110912297079148	0.6073986703294781	0.3686300054868197	T	T	T
0.1113784575669071	0.8067354457977470	0.3641918171006976	T	T	T
0.2221697124941344	0.0058700212066093	0.3636558552166871	T	T	T
0.2211864607675858	0.2044433837510868	0.3678862826746906	T	T	T
0.2297563866445493	0.4072994236126882	0.3706896626892652	T	T	T
0.2233501472856293	0.6079176834348935	0.3665722155884680	T	T	T
0.2222673315486325	0.8070226924056972	0.3636156244408260	T	T	T
0.3334469434665239	0.0059590147500377	0.3642121960056005	T	T	T
0.3333414865486622	0.2045658116931668	0.3704414880235057	T	T	T
0.3342404094349486	0.4059167397222451	0.3716742728122141	T	T	T
0.3330859438779021	0.6090882064546120	0.3660525278961962	T	T	T
0.3335501210363296	0.8073370022705300	0.3627911620201631	T	T	T
0.4445945261461245	0.0063544808793864	0.3641855673009525	T	T	T
0.4446067020233099	0.2048933374128603	0.3685568360830604	T	T	T
0.4382379618083700	0.4074853227437578	0.3693239233189939	T	T	T
0.4459326308781626	0.6084468492477419	0.3629655182725495	T	T	T
0.4446976916974538	0.8075533568137930	0.3620952039739941	T	T	T
0.5555581859849572	0.0063186981462746	0.3640055175316728	T	T	T
0.5549806756600855	0.2054479742623864	0.3693339950141622	T	T	T
0.5627303564109526	0.4078894752241174	0.3674770696458408	T	T	T
0.5561554655472003	0.6093604759459386	0.3630970907550460	T	T	T
0.5554435113252053	0.8076571533245108	0.3619862166955273	T	T	T
0.6667812689637598	0.0060789238592174	0.3637298148177419	T	T	T
0.6673912794945136	0.2046100429838835	0.3701522976091609	T	T	T
0.6658710742354776	0.4074311073991133	0.3695201636588188	T	T	T
0.6680789456525063	0.6093268874534203	0.3643563783918538	T	T	T
0.6670138955366969	0.8075459733300057	0.3622953674871192	T	T	T
0.7780187152828331	0.0061533971914392	0.3633436993618274	T	T	T
0.7778454582413906	0.2050305543735599	0.3681527351049311	T	T	T
0.7707034265700324	0.4078965904793696	0.3690389368971676	T	T	T
0.7779301725901027	0.6081613083316184	0.3650405769261673	T	T	T
0.7783788187924849	0.8072819775982264	0.3633932653500505	T	T	T
0.8889279519336306	0.0060344015798973	0.3628710665381571	T	T	T
0.8886317773008227	0.2049310050000408	0.3657714121121756	T	T	T
0.8938648834629029	0.4063032845949796	0.3721051840545806	T	T	T
0.8896150520124524	0.6074311572489021	0.3679178266979111	T	T	T
0.8890217754646899	0.8068904758217194	0.3640531602466418	T	T	T
0.0001135254462048	0.0573970145799997	0.2903271920382420	T	T	T
0.0000011824253098	0.2578568215784435	0.2944559197434156	T	T	T
0.0004665920761699	0.4578883750529535	0.3003111033109626	T	T	T
0.0003695373159130	0.6571799474699727	0.2954130517471081	T	T	T

0.0001537600985630	0.8573137639009648	0.2913673365191933	T	T	T
0.1117015098363085	0.0580458857943659	0.2911086715918604	T	T	T
0.1131613099884617	0.2603175529575292	0.2970262325893917	T	T	T
0.1088460234828937	0.4566390598813794	0.2998349458511416	T	T	T
0.1110491752853885	0.6572362337540353	0.2948907611346877	T	T	T
0.1111896757412492	0.8575826363364797	0.2912489775178926	T	T	T
0.2228959328085109	0.0584718285963766	0.2921342190317678	T	T	T
0.2221982955267572	0.2596178460920588	0.2988829036881808	T	T	T
0.2218381059947122	0.4597156349795562	0.3014194248329416	T	T	T
0.2221069986144730	0.6581784419665438	0.2936579259023442	T	T	T
0.2222297379528048	0.8582038947817271	0.2910090067611221	T	T	T
0.3337783202466419	0.0585718704599048	0.2927357736948607	T	T	T
0.3338730608081996	0.2566081998127151	0.2989154896551391	T	T	T
0.3338974076955158	0.4551693679960210	0.2965156201571120	T	T	T
0.3327872928217242	0.6588500980276604	0.2922311131979990	T	T	T
0.3333577265397141	0.8589176323825372	0.2905010880528162	T	T	T
0.4446403139951037	0.0594923763329825	0.2930601962765594	T	T	T
0.4447801489932864	0.2593965383824688	0.2995358680229868	T	T	T
0.4445675391771747	0.4594441205551865	0.2993761162637772	T	T	T
0.4447359832917632	0.6594075871923130	0.2904752745572973	T	T	T
0.4445836898309402	0.8593915034244790	0.2900141885000354	T	T	T
0.5555428260337175	0.0594038994539669	0.2928691506073712	T	T	T
0.5545837198385601	0.2594802619003797	0.2999924750461279	T	T	T
0.5594198253024745	0.4568531333030100	0.2935881774429339	T	T	T
0.5562149290533357	0.6600282923730457	0.2902557484232398	T	T	T
0.5555122081808432	0.8594196843739711	0.2899099126888053	T	T	T
0.6662184066896224	0.0588953940994200	0.2924301503054336	T	T	T
0.6667768006781377	0.2564154134799491	0.2983516873998804	T	T	T
0.6670368070417338	0.4565557348301797	0.2944305522712667	T	T	T
0.6679397753679349	0.6599464486641599	0.2912813482536096	T	T	T
0.6670723577296492	0.8592004199844270	0.2901454541137840	T	T	T
0.7772676582996371	0.0587043500709454	0.2918184710744456	T	T	T
0.7778623658992433	0.2601321917030817	0.2992147540427154	T	T	T
0.7792536884408002	0.4595947090057955	0.2991001941836665	T	T	T
0.7788719873791018	0.6588587611503958	0.2925328895383979	T	T	T
0.7784543826253283	0.8584319446165254	0.2908441697630155	T	T	T
0.8884765933615075	0.0581834570240841	0.2910009310655496	T	T	T
0.8871081222336323	0.2604585490306144	0.2969793545349854	T	T	T
0.8921819296718155	0.4567047144453527	0.2992122416989960	T	T	T
0.8899521078440407	0.6573829083803666	0.2943706642577481	T	T	T
0.8892257393484643	0.8578418038185073	0.2912133178157527	T	T	T
0.0560167813298894	0.1566354995287472	0.3131504678516179	T	T	T
0.0547170519536016	0.3573981523282766	0.3187556247491192	T	T	T
0.0553712362570623	0.5562824604529153	0.3191670359010108	T	T	T
0.0557511662264987	0.7563483881038755	0.3137079017046958	T	T	T
0.0558529336800149	0.9561239330964206	0.3112282696795086	T	T	T
0.1673003804666564	0.1563864357386464	0.3131593197483761	T	T	T
0.1674351659911605	0.3586173894798568	0.3242725632205878	T	T	T
0.1656010611569649	0.5562443836159088	0.3183674740337404	T	T	T
0.1665925245629844	0.7566374262172562	0.3131158207845359	T	T	T
0.1669795532034219	0.9565137140674670	0.3114731015458438	T	T	T
0.2782205161045972	0.1566191863065541	0.3157870446705478	T	T	T
0.2803078674764483	0.3551871263988113	0.3188040918744810	T	T	T
0.2790357101957222	0.5570128644285240	0.3157528473659121	T	T	T

0.2776653470926823	0.7574421012464605	0.3120356925350821	T	T	T
0.2779855707019054	0.9569418849154682	0.3119140777065566	T	T	T
0.3887997956975532	0.1568124514212354	0.3160676443391470	T	T	T
0.3877612666701065	0.3548453872754673	0.3190384361148045	T	T	T
0.3877669197104597	0.5570630419350397	0.3155777530356884	T	T	T
0.3888431828783111	0.7579575120168960	0.3108796435544789	T	T	T
0.3890392655019423	0.9574745377397815	0.3119117818645960	T	T	T
0.5001348479488874	0.1571480889834037	0.3150855298433362	T	T	T
0.4997903208362335	0.3584898556413880	0.3231399761087296	T	T	T
0.5022110490397289	0.5578105224232531	0.3122868728810311	T	T	T
0.5001160974504664	0.7583182008956026	0.3101900739515279	T	T	T
0.5000562015116971	0.9577310520187063	0.3116011780435112	T	T	T
0.6110350006800420	0.1572966220662387	0.3163292556327711	T	T	T
0.6134410611840614	0.3550516121189671	0.3164169922667147	T	T	T
0.6129348424107486	0.5582688623993776	0.3139426271834130	T	T	T
0.6115688285855971	0.7584974728511632	0.3105530711569298	T	T	T
0.6111431669265582	0.9575769201814169	0.3114957808172947	T	T	T
0.7219037194681404	0.1568732296739737	0.3153755274284630	T	T	T
0.7201970690386099	0.3554020073699017	0.3178591887800581	T	T	T
0.7225564843520813	0.5577465301458311	0.3133982421962184	T	T	T
0.7232033438723457	0.7579396039969548	0.3114826336176100	T	T	T
0.7223499747904241	0.9570963278328357	0.3115142328533739	T	T	T
0.8326002496212531	0.1570384559738106	0.3136486393243642	T	T	T
0.8332249770549666	0.3588822330719217	0.3237973016105762	T	T	T
0.8355694018730027	0.5562003692840622	0.3168164076772990	T	T	T
0.8340627506785141	0.7569954751805559	0.3125945700681563	T	T	T
0.8333917860617868	0.9568278513844369	0.3112629489727347	T	T	T
0.9440413110793026	0.1565267459763565	0.3128209826261080	T	T	T
0.9458346464096357	0.3574125882729792	0.3184996867818564	T	T	T
0.9454375608639525	0.5563025686726992	0.3187777841479049	T	T	T
0.9447110987666776	0.7562632080850699	0.3136244654498229	T	T	T
0.9444768201199554	0.9562995877046266	0.3112763452170349	T	T	T
0.0000261769127540	0.1251107487559693	0.4083220572985662	T	T	T
0.0000927556594571	0.3245154796457289	0.4138932490067481	T	T	T
0.0001604908816668	0.5297680969542259	0.4168562775965622	T	T	T
0.0001885024771349	0.7285406733313512	0.4103271168760549	T	T	T
0.0001962853592364	0.9270894604030977	0.4075376701171287	T	T	T
0.1104841164087101	0.1250066062794314	0.4087565640742000	T	T	T
0.1068267260467022	0.3261574163361635	0.4122840010066873	T	T	T
0.1106164539514760	0.5291154980535994	0.4163670622501326	T	T	T
0.1116373614218667	0.7285754354149203	0.4100586646520177	T	T	T
0.1111890891638042	0.9270812504269478	0.4076426404041029	T	T	T
0.2218074653792639	0.1245843743225378	0.4103205682325584	T	T	T
0.2250845306789871	0.3282410166370588	0.4114531163070662	T	T	T
0.2226379064451063	0.5286346572272616	0.4082162380049351	T	T	T
0.2226921847314472	0.7289211812470338	0.4091763834941383	T	T	T
0.2222779309502633	0.9269299455776407	0.4077801664468613	T	T	T
0.3332297328501564	0.1243595314164303	0.4118768024494464	T	T	T
0.3332142834508075	0.3273590046470152	0.4172197178355486	T	T	T
0.3352199027886461	0.5320902300756158	0.4149304813101042	T	T	T
0.3342376883735138	0.7291268210042875	0.4078480416595365	T	T	T
0.3335670454632700	0.9268528794517865	0.4078631722066848	T	T	T
0.4446114175741521	0.1253036738086848	0.4112726278568788	T	T	T
0.4413081864013774	0.3294240491877066	0.4135575466982230	T	T	T

0.4458529073841108	0.5292987975650346	0.4050902671311643	T	T	T
0.4451330077371411	0.7291555878570981	0.4067520298509120	T	T	T
0.4446416853722414	0.9271507496155154	0.4076734950603855	T	T	T
0.5557340226050478	0.1254405465452995	0.4113535988060378	T	T	T
0.5597587106984586	0.3304062744266818	0.4109378688499182	T	T	T
0.5587461223387203	0.5319477505320058	0.4092746984673994	T	T	T
0.5556732048020036	0.7292965762092806	0.4066618434632868	T	T	T
0.5557008916757175	0.9271300585315522	0.4075028053420777	T	T	T
0.6669113568714862	0.1245781669821948	0.4117240461291927	T	T	T
0.6660454212668964	0.3293479858447376	0.4168668364505392	T	T	T
0.6664399799685793	0.5322324222596779	0.4117073406324982	T	T	T
0.6669287683525634	0.7293804818621666	0.4071534802704638	T	T	T
0.6667771130934765	0.9270308839612329	0.4072449463677693	T	T	T
0.7779260889137816	0.1249086602325778	0.4100749801116873	T	T	T
0.7746588529792980	0.3292664126729802	0.4105762924617669	T	T	T
0.7784200686935578	0.5290928708893919	0.4065593308773626	T	T	T
0.7780624712894132	0.7289683946954859	0.4086516328037700	T	T	T
0.7780440494684181	0.9272644174315294	0.4075860679096608	T	T	T
0.8896695962666351	0.1250944549036526	0.4086243629846370	T	T	T
0.8934695894323430	0.3265270857000034	0.4122073776760070	T	T	T
0.8896449213623832	0.5291433721222485	0.4152994692290055	T	T	T
0.8887813465140159	0.7286248605539967	0.4097346682963792	T	T	T
0.8891123538141323	0.9273414021184139	0.4076344115375703	T	T	T
0.0554920438565249	0.0247195182698748	0.4280365379049250	T	T	T
0.0553134588629688	0.2224441175358803	0.4311132437002974	T	T	T
0.0546782837049877	0.4273266421164388	0.4392275369517400	T	T	T
0.0558815628893138	0.6283277480363887	0.4336733324105018	T	T	T
0.0557399958233801	0.8267252398081208	0.4292730071659465	T	T	T
0.1664003397640494	0.0243488846747250	0.4288419893182555	T	T	T
0.1652114036974932	0.2165770971862005	0.4343896585757266	T	T	T
0.1693822510277896	0.4234119247877750	0.4325703540729232	T	T	T
0.1677890656707156	0.6280841336328585	0.4321908925949548	T	T	T
0.1669970018576684	0.8266920903425339	0.4291146119100445	T	T	T
0.2775294254890220	0.0240592389185001	0.4295142354159203	T	T	T
0.2762510168494642	0.2200911955212401	0.4355229304677496	T	T	T
0.2807313545798907	0.4276827580682381	0.4371643281836973	T	T	T
0.2783975637505351	0.6286298712838556	0.4315736107688477	T	T	T
0.2781921297233786	0.8265906370625532	0.4284433791184018	T	T	T
0.3889815965935369	0.0242029420064225	0.4298575289847688	T	T	T
0.3899690910419272	0.2203293897145578	0.4368266014974590	T	T	T
0.3889275108801253	0.4300330433678695	0.4370272972856327	T	T	T
0.3908909121877703	0.6291016921341238	0.4288914760479567	T	T	T
0.3893601347684105	0.8266188896285444	0.4278048575969926	T	T	T
0.5002070445277238	0.0244306980437878	0.4298401522537547	T	T	T
0.4996195536616804	0.2197264565545235	0.4358576428953785	T	T	T
0.5015046197777218	0.4253786851404504	0.4272377543027934	T	T	T
0.5008743954580945	0.6283295224377997	0.4283581600930682	T	T	T
0.5001379714526895	0.8268216475433771	0.4275739271415632	T	T	T
0.6113514799816171	0.0242220106559353	0.4294884557123994	T	T	T
0.6106260535882930	0.2214181832824929	0.4359253893648282	T	T	T
0.6120842556427823	0.4312627813911490	0.4355029873734108	T	T	T
0.6116475642530576	0.6299634555544157	0.4284746004240020	T	T	T
0.6111640183374697	0.8267362681653775	0.4273694342983549	T	T	T
0.7226196815575534	0.0241703453591304	0.4291330579484181	T	T	T

0.7232742077791944	0.2211644963444811	0.4360684983379348	T	T	T
0.7195916828414490	0.4289754936991005	0.4353260338378428	T	T	T
0.7228792404048817	0.6284883478232018	0.4300981375271060	T	T	T
0.7222404948843120	0.8267842250165989	0.4280997520216349	T	T	T
0.8337363199871175	0.0249135062379274	0.4285398916741608	T	T	T
0.8346594184616696	0.2166911894898408	0.4341445786607820	T	T	T
0.8306862670102063	0.4234924104717870	0.4314051813304430	T	T	T
0.8328636844161854	0.6281412662427435	0.4312347011133078	T	T	T
0.8334630574862987	0.8268621569583611	0.4290313161833644	T	T	T
0.9447807599440765	0.0248823525686774	0.4279593917068033	T	T	T
0.9446411805033563	0.2226325628695314	0.4315609966241905	T	T	T
0.9455280000431456	0.4275554746392460	0.4388818335888319	T	T	T
0.9444600955078831	0.6282101229614465	0.4333980565272901	T	T	T
0.9446008337489095	0.8267503909413652	0.4292139563945455	T	T	T
0.2117878431418571	0.2768329469709025	0.5331314127932435	T	T	T
0.3304395541818046	0.2743287099370156	0.5318774939906176	T	T	T
0.4654883466547478	0.2904673050349873	0.5457432165918694	T	T	T
0.5527808507709697	0.2686511542714539	0.5342419119996638	T	T	T
0.6679736258563368	0.2787024643860406	0.5319617601326588	T	T	T
0.7864865570822203	0.2776340585601773	0.5316342540255687	T	T	T
0.1498151047556839	0.3417718749292143	0.5360867266994571	T	T	T
0.2614274740461818	0.3741732935527766	0.5333474041596311	T	T	T
0.3788949736576526	0.3503545498414000	0.5527518373438149	T	T	T
0.4875944163653659	0.3766997509951814	0.5158372682388640	T	T	T
0.6142967776678321	0.3589419606203972	0.5549461893930379	T	T	T
0.7364073062287555	0.3740981651802807	0.5339397205737469	T	T	T
0.8489274236805225	0.3419529091114513	0.5354865327445670	T	T	T
0.1055729503469061	0.4342763318396766	0.5437464817290568	T	T	T
0.8939690168080245	0.4340093505175417	0.5417066312274961	T	T	T
0.2160454980222205	0.4943271959677288	0.5304118890071189	T	T	T
0.3020079171423230	0.4914494886793987	0.5543379886225343	T	T	T
0.4403962032430838	0.5001650966934209	0.5268657425887998	T	T	T
0.5272043437410149	0.4927952972542268	0.5475548552844655	T	T	T
0.7005989626845763	0.4935041807425491	0.5523096422879784	T	T	T
0.7859860671477101	0.4946877917901905	0.5288995981956160	T	T	T
0.1690564297390960	0.4297486850375630	0.6043887080286650	T	T	T
0.3893404215817077	0.4517259214246416	0.5971932901486271	T	T	T
0.6093721236597504	0.4528543154251198	0.5927314741108238	T	T	T
0.8305129110537836	0.4311550314764238	0.6027430519535327	T	T	T
0.1634042543423101	0.2960728660281727	0.4648600459608211	T	T	T
0.2731582347466536	0.3062435419477582	0.4788436220905263	T	T	T
0.3920920173911194	0.3013502806508975	0.4787651390712593	T	T	T
0.5033772607690342	0.3036144167508672	0.4678799322444754	T	T	T
0.6075070450182497	0.3075468766653450	0.4786816299180252	T	T	T
0.7263444368450561	0.3082143775700965	0.4775248054560378	T	T	T
0.8362367189772346	0.2959720826090282	0.4639491329503511	T	T	T
0.1883237281916135	0.4163557855513192	0.5203499187467110	T	T	T
0.3283068247445963	0.4149983496816549	0.5513849418812229	T	T	T
0.4164216434456035	0.4207453563677414	0.5209298226194725	T	T	T
0.5515769620325802	0.4143422523944682	0.5474185344430508	T	T	T
0.6708103121622232	0.4186018348537995	0.5477982405075792	T	T	T
0.8108183552345136	0.4155140772321884	0.5191113132658328	T	T	T
0.2747407931501744	0.5075110912272409	0.4713120481233120	T	T	T
0.5001767308970504	0.5036802631687205	0.4649685840327615	T	T	T

```
  0.7265667387617474  0.5090637892587444  0.4702661965928598   T   T   T
== Embedded cluster I-Pd_2k+1 k=1
   1.00000000000000
     24.6586885454000111    0.0000000000000000    0.0000000000000000
      0.0000000000000000   25.2576961516000082    0.0000000000000000
      0.0000000000000000    0.0000000000000000   30.0000000000007034
   W     Te    Pd
    56   112     3
Selective dynamics
Direct
  0.0711975872586778  0.0989274702559879  0.4926796438998975   T   T   T
  0.2129650857788880  0.0985217073879601  0.4921912035206097   T   T   T
  0.3561624537780247  0.0984061624757576  0.4920126605579230   T   T   T
  0.4999446389299858  0.0977226401661412  0.4924863271451857   T   T   T
  0.6428629377679439  0.0977127685617122  0.4925166562275519   T   T   T
  0.7865567425461319  0.0984664250415769  0.4922864169778197   T   T   T
  0.9296549641659392  0.0986916123166242  0.4925391963696424   T   T   T
  0.0713327893148053  0.3491065870254321  0.4935470842244458   T   T   T
  0.2130139549653227  0.3487520470094516  0.4931467544211217   T   T   T
  0.3539620517994339  0.3478850163296986  0.4935156920515446   T   T   T
  0.4981862023249567  0.3451038723372424  0.4968030505162574   T   T   T
  0.6445314789709848  0.3451758473741204  0.4967766725611604   T   T   T
  0.7886779827820174  0.3480130715649383  0.4935505074925798   T   T   T
  0.9295655464065234  0.3488889018414500  0.4932999674043383   T   T   T
  0.0715866553094460  0.5987664775685736  0.4935316821725129   T   T   T
  0.2134297325533945  0.5989502283866155  0.4936292037938890   T   T   T
  0.3555756768324250  0.5994931974663513  0.4936615747210830   T   T   T
  0.5016161076379264  0.5989973177672906  0.4946706627601788   T   T   T
  0.6413678899059994  0.5991093009665328  0.4948092270726814   T   T   T
  0.7876180684098348  0.5996068260351296  0.4936900519444086   T   T   T
  0.9298770587576991  0.5990077934040997  0.4934863134794658   T   T   T
  0.0714497025915349  0.8487317105622693  0.4931101071645858   T   T   T
  0.2132065373243940  0.8487060030210952  0.4928876290887208   T   T   T
  0.3559607738711199  0.8487400115723343  0.4923656063784849   T   T   T
  0.4996107087546011  0.8488115486648361  0.4921487218797368   T   T   T
  0.6433112859165728  0.8487931109467022  0.4923291478219236   T   T   T
  0.7871997612631443  0.8488065836901371  0.4924268290768155   T   T   T
  0.9297500288841072  0.8488206632273071  0.4928651038562377   T   T   T
  0.0005792738435751  0.0094789683449472  0.4988759446481740   T   T   T
  0.1420292893428359  0.0093802144241297  0.4987716962131901   T   T   T
  0.2842654915000271  0.0095260287822474  0.4986785138497151   T   T   T
  0.4278847407568984  0.0094614815721017  0.4987846676116231   T   T   T
  0.5714893255039307  0.0090359161010065  0.4990626627184210   T   T   T
  0.7150269700526954  0.0094229648990679  0.4989580420549304   T   T   T
  0.8585602473826168  0.0095958161741041  0.4988439954369034   T   T   T
  0.0004722153735257  0.2597613305056181  0.4995387376323944   T   T   T
  0.1420723975109755  0.2596320217244864  0.4994777298951770   T   T   T
  0.2837093303148594  0.2590845090171011  0.4989287321987384   T   T   T
  0.4310133113597999  0.2586970640512080  0.4999795154730851   T   T   T
  0.5713996533116371  0.2588296476399724  0.5021842312022711   T   T   T
  0.7116414045458734  0.2587967984994711  0.5000290444430460   T   T   T
  0.8588728576893789  0.2592140129135851  0.4990928573527517   T   T   T
  0.0005245966846161  0.5095184777419928  0.4998617639903656   T   T   T
  0.1424936735323871  0.5094541719493566  0.4998723885090349   T   T   T
```

0.2838756028073968	0.5098358040311377	0.5002885192173184	T	T	T
0.4266918895669943	0.5111071481391064	0.5019600530852139	T	T	T
0.5714961906945313	0.5131507504688779	0.5048386641833050	T	T	T
0.7161926494764415	0.5112778739622607	0.5020926779202175	T	T	T
0.8590294051843610	0.5099702828468974	0.5003067513381722	T	T	T
0.0007203315939253	0.7595475084863821	0.4996295096158940	T	T	T
0.1423432566008841	0.7595105331937781	0.4997315894356920	T	T	T
0.2845482504310262	0.7598942903144053	0.4996174760021951	T	T	T
0.4280844719627219	0.7600610609885865	0.4993345219689093	T	T	T
0.5715601325323854	0.7604716531977785	0.4995327263542965	T	T	T
0.7149943815784159	0.7601425621986180	0.4995128509066786	T	T	T
0.8585888618969693	0.7599417315507347	0.4995456668399371	T	T	T
0.1418446539878705	0.0746710582537414	0.4269185968549850	T	T	T
0.2847347190852099	0.0748635798691782	0.4267960490386468	T	T	T
0.4281935838678468	0.0748269309206457	0.4270502411548912	T	T	T
0.5714676798188906	0.0742390666369455	0.4271927407667797	T	T	T
0.7146102787419440	0.0748166318562626	0.4272024689037001	T	T	T
0.8579623250692503	0.0749947159215814	0.4271030668883798	T	T	T
0.0003284648369024	0.0748711678395909	0.4269623219761424	T	T	T
0.1419338946053577	0.3248882410045875	0.4276332252201077	T	T	T
0.2842341410051265	0.3246925059004701	0.4273866305559715	T	T	T
0.4281858208620301	0.3261179907801581	0.4294910224545240	T	T	T
0.5713317950580038	0.3256272993065698	0.4315441138982526	T	T	T
0.7145277250597100	0.3261042690932550	0.4294984053353929	T	T	T
0.8584667847562877	0.3248053616819466	0.4274309808870951	T	T	T
0.0005366288275100	0.3251393036899899	0.4277283372041525	T	T	T
0.1428043568876521	0.5748894847496875	0.4280339402974362	T	T	T
0.2851488710770313	0.5748761130226081	0.4282130480161311	T	T	T
0.4291244979422200	0.5745191377988700	0.4295997369932331	T	T	T
0.5716383923928082	0.5740709417913820	0.4288224812122832	T	T	T
0.7139689762609940	0.5746702489992425	0.4297492606836989	T	T	T
0.8581363281283217	0.5749580647734398	0.4282140832420301	T	T	T
0.0006287676640765	0.5747563682965248	0.4279184681464124	T	T	T
0.1423610180152042	0.8243607872871817	0.4275310908086874	T	T	T
0.2846164390264382	0.8244859802963576	0.4273900722148330	T	T	T
0.4278371395091112	0.8245570881522249	0.4271951032632613	T	T	T
0.5716058942909655	0.8246739215928408	0.4271228121004935	T	T	T
0.7151679603724354	0.8245227710040325	0.4273569943863922	T	T	T
0.8584427403516911	0.8247070257973138	0.4273261167862287	T	T	T
0.0005924996572918	0.8244730738851356	0.4275012278505888	T	T	T
0.0711794655060618	0.1974052169172737	0.4476643554490023	T	T	T
0.2127163030190668	0.1972637052280611	0.4472078211415120	T	T	T
0.3570322525756381	0.1980130797214026	0.4481604301107870	T	T	T
0.5005840216463775	0.1968192195637948	0.4482325391278321	T	T	T
0.6421295498148298	0.1968700707086103	0.4483061259367348	T	T	T
0.7856578768734963	0.1979959173590937	0.4483419250177841	T	T	T
0.9297089392431077	0.1972670002076259	0.4473923709564935	T	T	T
0.0715399434982615	0.4470945204688111	0.4483170639855159	T	T	T
0.2136683614046953	0.4473210535282438	0.4483190309828279	T	T	T
0.3564946398575196	0.4483860363887833	0.4495675090735525	T	T	T
0.5020492789418358	0.4468973289529362	0.4563437745884100	T	T	T
0.6410590849868160	0.4469982879036872	0.4563227588416878	T	T	T
0.7863362090080972	0.4485138580415415	0.4496912905072804	T	T	T
0.9292350135074054	0.4474288001947660	0.4483906455983360	T	T	T

0.0715786307943788	0.6970764527547365	0.4482837795702743	T	T	T
0.2137559304832146	0.6972543993908827	0.4484336263337733	T	T	T
0.3564449084447739	0.6978343134765440	0.4484544747816334	T	T	T
0.5003110434958536	0.6976401376936896	0.4486891722222652	T	T	T
0.6429319046164902	0.6977293105236291	0.4488116481602453	T	T	T
0.7866283065753994	0.6979226229603662	0.4485272236013703	T	T	T
0.9294862244315047	0.6973702635049093	0.4482505702483889	T	T	T
0.0713899100524969	0.9467732542724502	0.4473676822972254	T	T	T
0.2134329748168129	0.9469792152469204	0.4473402656259709	T	T	T
0.3564525731586003	0.9474191508245517	0.4474469571635271	T	T	T
0.4997972441750174	0.9475582041203484	0.4474674160159662	T	T	T
0.6432880754654547	0.9473911167190264	0.4475601962064231	T	T	T
0.7865108666537143	0.9475321585554854	0.4474907655177151	T	T	T
0.9292375530004096	0.9471301546015862	0.4473895908376425	T	T	T
0.0003136614551064	0.1611704107798120	0.5443694272215313	T	T	T
0.1425210433455860	0.1608818422558063	0.5441895770295272	T	T	T
0.2848364852274521	0.1606690019914475	0.5433055674438205	T	T	T
0.4284070321287878	0.1598044714859237	0.5437279777556783	T	T	T
0.5713969952156738	0.1589852269876900	0.5448464430573748	T	T	T
0.7142467135847871	0.1599074182322422	0.5438656198082547	T	T	T
0.8578101992052756	0.1606778938199631	0.5437811819415737	T	T	T
0.0001556482323275	0.4113126649329614	0.5450944149517418	T	T	T
0.1426303643273927	0.4111718613535144	0.5450778420293076	T	T	T
0.2839413732919583	0.4107399683147123	0.5453318397126183	T	T	T
0.4246266997836255	0.4122216137220821	0.5448064041262276	T	T	T
0.7179420917079214	0.4123801113378036	0.5449469580484776	T	T	T
0.8586432319256057	0.4109346489339178	0.5453959221603260	T	T	T
0.0006859162263722	0.6614279349862066	0.5449892575889906	T	T	T
0.1426172958152289	0.6612904889185566	0.5450878896340167	T	T	T
0.2847371445939694	0.6616946147660817	0.5448870986031442	T	T	T
0.4282005645418056	0.6617914580575933	0.5442060318289195	T	T	T
0.5714279781879188	0.6629000314365348	0.5452557193267910	T	T	T
0.7149469943080949	0.6618237889176589	0.5442841413139092	T	T	T
0.8585987940537499	0.6617105455498675	0.5448505068899420	T	T	T
0.0003065447050627	0.9113010281271388	0.5443146440510216	T	T	T
0.1425753211892747	0.9111786284766048	0.5442946349179789	T	T	T
0.2851315316168602	0.9111741656556354	0.5437685231362941	T	T	T
0.4280343602207626	0.9107831600117304	0.5433615282450872	T	T	T
0.5713648637832540	0.9103675101055557	0.5435070710144787	T	T	T
0.7149068493312235	0.9106747465853231	0.5435187860526398	T	T	T
0.8578881575124008	0.9111673890978377	0.5438419651689287	T	T	T
0.0714271615329297	0.0334107474420506	0.5644273374000759	T	T	T
0.2133840366296997	0.0333886837895241	0.5641575778956065	T	T	T
0.3562207572205092	0.0334480836927714	0.5638650809993991	T	T	T
0.4996530714862154	0.0325232890777299	0.5642488754293855	T	T	T
0.6432681625209962	0.0326949939239846	0.5643101004260347	T	T	T
0.7866687272788233	0.0333403767951567	0.5640721118743700	T	T	T
0.9293078545984851	0.0334461264593846	0.5644366336013454	T	T	T
0.0713505126080800	0.2833863503815655	0.5652875041878088	T	T	T
0.2135260258391925	0.2832303439498404	0.5649549861989883	T	T	T
0.3562582005111354	0.2818166404723393	0.5641824275357185	T	T	T
0.4994922340419678	0.2779396952864850	0.5692207066067636	T	T	T
0.6432277256729427	0.2781222927071660	0.5692914680084453	T	T	T
0.7862551364561430	0.2820894036520783	0.5642318806491547	T	T	T

```
  0.9290315532813006  0.2832699189469423  0.5650955424806489   T   T   T
  0.0714551520981028  0.5336418718231847  0.5654579651589300   T   T   T
  0.2128272823569494  0.5340692872808324  0.5657343574490650   T   T   T
  0.3538254953914633  0.5355218390381304  0.5661306173029533   T   T   T
  0.4944005953265403  0.5381822157094652  0.5698987277895270   T   T   T
  0.6483263445017936  0.5381135562029200  0.5699871448141390   T   T   T
  0.7891495754321003  0.5357350768389385  0.5661678210124714   T   T   T
  0.9301134405928918  0.5343057612977984  0.5656653131863403   T   T   T
  0.0714781136001689  0.7837771539732638  0.5653119554412251   T   T   T
  0.2136713817273366  0.7839242591378359  0.5651520857133054   T   T   T
  0.3566660877601708  0.7842833159178790  0.5645741414858843   T   T   T
  0.4996841415730043  0.7845966484414999  0.5644568828527242   T   T   T
  0.6433071616653792  0.7844678114007548  0.5646836962570513   T   T   T
  0.7865917901591880  0.7843057870203523  0.5646091447573929   T   T   T
  0.9294092970190676  0.7840315129486773  0.5650569249028446   T   T   T
  0.5712174921742919  0.3956221220770670  0.6506501861316980   T   T   T
  0.5138870895077919  0.3833127725751087  0.5797483941690846   T   T   T
  0.6285308431203489  0.3834102063328920  0.5796382442148876   T   T   T
  0.5712197495704604  0.4708240047360536  0.5876103318553356   T   T   T
== Embedded cluster I-Pd_2k+1 k=2
   1.00000000000000
    24.6586885454000111    0.0000000000000000    0.0000000000000000
     0.0000000000000000   25.2576961516000082    0.0000000000000000
     0.0000000000000000    0.0000000000000000   30.0000000000007034
   W    Te   Pd
    56   112     5
Selective dynamics
Direct
  0.0706292792168228  0.0978694735349835  0.4918094758618083   T   T   T
  0.2129591737433685  0.0970938149499773  0.4908461171405547   T   T   T
  0.3558651849096334  0.0963937386630789  0.4897106916264712   T   T   T
  0.5001638406261343  0.0979937983595515  0.4907184761252432   T   T   T
  0.6444390416913964  0.0963038768557243  0.4897454324224814   T   T   T
  0.7873766321030863  0.0969733210439750  0.4908502729930128   T   T   T
  0.9294703653198656  0.0978089182509755  0.4918082221488776   T   T   T
  0.0707906121380050  0.3478276643112049  0.4948715054131419   T   T   T
  0.2122602309384742  0.3470391046801753  0.4949924214551097   T   T   T
  0.3511273562683448  0.3440391623507299  0.4938689418045141   T   T   T
  0.5001867805223907  0.3384757121086620  0.4929991363275110   T   T   T
  0.6493167919415703  0.3438990756478097  0.4937314432039578   T   T   T
  0.7880160943257271  0.3470534284309683  0.4950033505966293   T   T   T
  0.9292644239443607  0.3478271800522207  0.4948866723914024   T   T   T
  0.0711350145288228  0.5980077898666509  0.4962643429772784   T   T   T
  0.2130455981621310  0.5981127031916528  0.4975351313989778   T   T   T
  0.3539165179859412  0.5995921865389465  0.5002832317889111   T   T   T
  0.5000008573437213  0.5983591281093033  0.4985567078456362   T   T   T
  0.6461084337299449  0.5995687636822715  0.5002922943909858   T   T   T
  0.7869784562870852  0.5980810187589339  0.4975662553760297   T   T   T
  0.9289570514728387  0.5979769747014577  0.4962968913378408   T   T   T
  0.0706705165554686  0.8479129191033918  0.4930840035310402   T   T   T
  0.2129605344602780  0.8481155167612192  0.4924259414723461   T   T   T
  0.3565842617899574  0.8484550936083817  0.4917549891043250   T   T   T
  0.5000602452062677  0.8487741829048635  0.4914895757803919   T   T   T
  0.6435079497002792  0.8483814793784101  0.4918658360924964   T   T   T
```

0.7871065891850572	0.8479588422984136	0.4925033906398338	T	T	T
0.9293812586115769	0.8478617924860136	0.4931128856457277	T	T	T
0.0000213174370941	0.0086413433832527	0.4983164864808498	T	T	T
0.1413240527337738	0.0085943009667134	0.4979068473520529	T	T	T
0.2843395674702157	0.0083387739263390	0.4972352748229490	T	T	T
0.4284292873448409	0.0090122171777276	0.4971791564089879	T	T	T
0.5717995709856551	0.0089629017102310	0.4972234792219077	T	T	T
0.7158797919022826	0.0082228958130247	0.4973034371456745	T	T	T
0.8587751415504655	0.0084458474253953	0.4979604277571548	T	T	T
0.0000226023420869	0.2587662691313180	0.4999724856207899	T	T	T
0.1425277125343134	0.2582543759822086	0.4994099109127790	T	T	T
0.2835643007034724	0.2566660209573343	0.4990642586293528	T	T	T
0.4206363534931635	0.2568743415122177	0.4984411535501502	T	T	T
0.5798968194906706	0.2568348616288751	0.4984020336731253	T	T	T
0.7170569335300284	0.2565540784797636	0.4988545542474582	T	T	T
0.8576842276044080	0.2582210194713107	0.4993670649398905	T	T	T
0.0000255083641120	0.5086747495919965	0.5027984683022741	T	T	T
0.1419180562265715	0.5087746305302702	0.5035186844607284	T	T	T
0.2830701109945398	0.5094940992204283	0.5074093735373675	T	T	T
0.4173951674828684	0.5110659802720657	0.5109737582731591	T	T	T
0.5826429421183901	0.5110939822435885	0.5109492848921289	T	T	T
0.7169771009645859	0.5094473261707886	0.5074078281165786	T	T	T
0.8581484346749886	0.5087696417455705	0.5035636966095084	T	T	T
0.0000407771726085	0.7587109979098148	0.5006252313047802	T	T	T
0.1416838555237809	0.7591067509647000	0.5005765046493890	T	T	T
0.2843510357128554	0.7597968386460137	0.5005649060558522	T	T	T
0.4283797239159182	0.7608677531104946	0.4997537062649743	T	T	T
0.5717201682578512	0.7608481445383191	0.4998028674065308	T	T	T
0.7156491747873533	0.7596979685757717	0.5006000510963304	T	T	T
0.8584277387064992	0.7589981384049609	0.5005639236372943	T	T	T
0.1413351279738229	0.0735107158609060	0.4258099440885035	T	T	T
0.2837847584192622	0.0727615361797808	0.4249129598326313	T	T	T
0.4286507628420401	0.0739979003455662	0.4250727851742470	T	T	T
0.5716120090925053	0.0738529320377824	0.4250990165511437	T	T	T
0.7165098490998675	0.0726742779886453	0.4249846533549916	T	T	T
0.8588910030562279	0.0732242486000007	0.4257926344916153	T	T	T
0.0000877547685248	0.0736635666416307	0.4261066476183562	T	T	T
0.1415376329127364	0.3256768631399784	0.4288206587342268	T	T	T
0.2818645760101393	0.3234166994135959	0.4277721745594769	T	T	T
0.4259943919183805	0.3256862311473910	0.4288209190542930	T	T	T
0.5742172714020589	0.3256492789938256	0.4287548446665465	T	T	T
0.7185702400983580	0.3234534600851968	0.4276360977524892	T	T	T
0.8586378791100641	0.3256420090910699	0.4287914905977261	T	T	T
0.0000307666383292	0.3252440080554628	0.4288163686216909	T	T	T
0.1430327851707437	0.5736299142476425	0.4314333442407504	T	T	T
0.2855665572424740	0.5723490183695079	0.4337586948070610	T	T	T
0.4266229422141365	0.5702703379184615	0.4362185630651138	T	T	T
0.5733938614765784	0.5702793405936120	0.4362085570879537	T	T	T
0.7144440316309300	0.5723199688461180	0.4337611837778343	T	T	T
0.8570354433627143	0.5736272769352437	0.4314646008803443	T	T	T
0.0000355778711965	0.5736961258288824	0.4308924386337848	T	T	T
0.1417565282943168	0.8227371322474782	0.4276706061145529	T	T	T
0.2845810944577007	0.8228336856840889	0.4273562666511206	T	T	T
0.4282855615571484	0.8234610448434884	0.4266731361127291	T	T	T

0.5718699483148963	0.8234580047791862	0.4266900512479067	T	T	T
0.7154448515518164	0.8227548588694965	0.4274258030205040	T	T	T
0.8582809576255472	0.8227363894328961	0.4276876763737211	T	T	T
0.0000407498288830	0.8224401721746895	0.4277877301711838	T	T	T
0.0705177562190248	0.1967916925610331	0.4474317022567460	T	T	T
0.2125801308143523	0.1959419441595864	0.4464603493248107	T	T	T
0.3541840284154599	0.1955602915750358	0.4446719731605534	T	T	T
0.5002422778735059	0.1985224362337193	0.4489305511652026	T	T	T
0.6461871532942085	0.1953532071464524	0.4446802588276882	T	T	T
0.7878653189759320	0.1958081924200681	0.4463086788465678	T	T	T
0.9295955662988361	0.1967002117400750	0.4473619468910727	T	T	T
0.0712030859087783	0.4468219645790394	0.4512174685874317	T	T	T
0.2140814667949011	0.4476922654567751	0.4530431310680557	T	T	T
0.3562303068588070	0.4482806317010112	0.4569914730859535	T	T	T
0.5000319929505093	0.4518616581048172	0.4721292362230388	T	T	T
0.6438305678316962	0.4482428308491417	0.4570172938640389	T	T	T
0.7860212180318928	0.4476706889615472	0.4530614219316077	T	T	T
0.9288552087368880	0.4468302661557866	0.4512377410219500	T	T	T
0.0711230405006801	0.6956040261885447	0.4498528471418272	T	T	T
0.2135348151191757	0.6957733297984456	0.4505916124304629	T	T	T
0.3560474043963791	0.6961291340910422	0.4510060571343479	T	T	T
0.5000461232566292	0.6954178825970949	0.4507288311636166	T	T	T
0.6439759094675658	0.6960824277409268	0.4510256889465881	T	T	T
0.7864957495148652	0.6957034757876168	0.4506291887202941	T	T	T
0.9289974261182459	0.6955571852951428	0.4498420121523624	T	T	T
0.0707535485294353	0.9456838733514464	0.4468863155886449	T	T	T
0.2129208125618788	0.9460977228876259	0.4465460597018768	T	T	T
0.3566828080444041	0.9465846044594617	0.4462638261763066	T	T	T
0.5000787830496958	0.9470380145979624	0.4459924572930585	T	T	T
0.6434255454290314	0.9464924911502836	0.4463347073946138	T	T	T
0.7871649946889602	0.9459170724442980	0.4466187538773552	T	T	T
0.9293010561472093	0.9456206876188546	0.4468869505114624	T	T	T
0.0000594968742525	0.1597981347572805	0.5441035067550243	T	T	T
0.1424846419066644	0.1593118528259413	0.5432114137513353	T	T	T
0.2850328082190280	0.1578806566625930	0.5424439169515499	T	T	T
0.4261501570288645	0.1580973951237747	0.5417040445637420	T	T	T
0.5743369504459015	0.1580608335066951	0.5417323120877858	T	T	T
0.7154578672036284	0.1578241692333912	0.5423507232459130	T	T	T
0.8577330901193280	0.1592911342733865	0.5432121719646229	T	T	T
0.0000139659220879	0.4096973484039469	0.5472228821284500	T	T	T
0.1421016213485158	0.4091644967488082	0.5476697986228088	T	T	T
0.2823533213987185	0.4088148239717038	0.5520622544134957	T	T	T
0.7176818644428276	0.4087072375124303	0.5520460939757339	T	T	T
0.8580415637017361	0.4091944820845871	0.5477584255343575	T	T	T
0.0000613403589035	0.6611419607588427	0.5470410396265626	T	T	T
0.1414845253478740	0.6618053228732077	0.5473027471856449	T	T	T
0.2825308047567823	0.6634681793547149	0.5489106951354058	T	T	T
0.4268120384288380	0.6645207736388018	0.5458116610538776	T	T	T
0.5731839367500574	0.6644851537045868	0.5458381907510688	T	T	T
0.7174570598178707	0.6634399875540188	0.5489364825418318	T	T	T
0.8586318032894308	0.6617118657330574	0.5473640637735437	T	T	T
0.0000363439418111	0.9108484559589459	0.5441985977312475	T	T	T
0.1422776011053103	0.9108311819260057	0.5436097788644814	T	T	T
0.2851832126209564	0.9106754301114574	0.5426960959510861	T	T	T

```
  0.4284620978151690  0.9110484166095357  0.5422766576168316   T   T   T
  0.5716002686041979  0.9110142769399189  0.5423038877216964   T   T   T
  0.7149102474102594  0.9104939116876024  0.5428409582792276   T   T   T
  0.8578023418433911  0.9106824050269778  0.5437163894302651   T   T   T
  0.0711509569457933  0.0329133245091950  0.5638164245794292   T   T   T
  0.2135437988995139  0.0322880806482137  0.5628821141961122   T   T   T
  0.3565021313051361  0.0323367915338225  0.5621996131362016   T   T   T
  0.5001139343599099  0.0328992765805863  0.5625766858189194   T   T   T
  0.6436873901343106  0.0322608929646670  0.5622547767008512   T   T   T
  0.7865793618861522  0.0322349555246539  0.5629423553636846   T   T   T
  0.9289199288437487  0.0328304119434116  0.5638445657314352   T   T   T
  0.0716086372820244  0.2809618746190767  0.5655947081662825   T   T   T
  0.2134199678353591  0.2799873859218239  0.5654218509428239   T   T   T
  0.3544682116333243  0.2800030550497793  0.5659443828249892   T   T   T
  0.5003546679589939  0.2717289211640077  0.5609038077275906   T   T   T
  0.6463954322096893  0.2798537056339058  0.5657910047544475   T   T   T
  0.7869110636478385  0.2799233690471056  0.5654047240991409   T   T   T
  0.9285166688839126  0.2809330644240157  0.5655945279667959   T   T   T
  0.0702702912080775  0.5333033839183545  0.5686138378638231   T   T   T
  0.2097859089533474  0.5347194363001232  0.5706175614093381   T   T   T
  0.3478222759336028  0.5405949235861872  0.5774210639885785   T   T   T
  0.5000016798862117  0.5701334779809255  0.5827231761238506   T   T   T
  0.6522776724717916  0.5405577107060132  0.5774301899076874   T   T   T
  0.7902215107725504  0.5346963843305451  0.5706348351082468   T   T   T
  0.9298384907529164  0.5333127010500924  0.5686540634365167   T   T   T
  0.0709665930727018  0.7841867556497139  0.5659127252577005   T   T   T
  0.2130616399517184  0.7853132569686757  0.5655205471769649   T   T   T
  0.3566169132088423  0.7856004349181483  0.5648255204993261   T   T   T
  0.5000298720701589  0.7860249848395656  0.5647115525175430   T   T   T
  0.6434622568006941  0.7854342010588756  0.5649781494592970   T   T   T
  0.7869864517568467  0.7849894735418523  0.5655171319801755   T   T   T
  0.9291063565322351  0.7840261832234729  0.5659354865035552   T   T   T
  0.4404818939250608  0.3919547311638858  0.6251233283510876   T   T   T
  0.5600317666968351  0.3919390647963482  0.6249409113763794   T   T   T
  0.3914584195787768  0.4134549457968512  0.5469907612737857   T   T   T
  0.4999732169855357  0.4107835030986382  0.5523985901231308   T   T   T
  0.6085220604022209  0.4133838224749702  0.5467011503974111   T   T   T
  0.4426657160854664  0.4898222129831751  0.5972516167505264   T   T   T
  0.5574494064057705  0.4898196887304556  0.5971353679845139   T   T   T
== Embedded cluster I-Pd_2k+1 k=3
   1.00000000000000
    24.6586885454000111    0.0000000000000000    0.0000000000000000
     0.0000000000000000   25.2576961516000082    0.0000000000000000
     0.0000000000000000    0.0000000000000000   30.0000000000007034
   W    Te   Pd
    56   112     7
Selective dynamics
Direct
  0.0691535639683025  0.0962728264822767  0.4896881734936395   T   T   T
  0.2116969406049067  0.0956925613161670  0.4891959932923742   T   T   T
  0.3559644039847960  0.0976443592224724  0.4909749496734988   T   T   T
  0.5001995549027543  0.0966502979850108  0.4907079579942218   T   T   T
  0.6429040248286217  0.0973100205554359  0.4907508363479792   T   T   T
  0.7853412027795921  0.0980371603395407  0.4907338121065747   T   T   T
```

0.9269460718271527	0.0974946581567319	0.4904355166955236	T	T	T
0.0689559227241239	0.3466615471057564	0.4948847479383317	T	T	T
0.2060332596617553	0.3424444151949245	0.4942685057041480	T	T	T
0.3530993042592691	0.3392715203080134	0.4965734209609223	T	T	T
0.5033281739347486	0.3432934843910088	0.5033207622440057	T	T	T
0.6462212625061110	0.3444513587939253	0.4952050911340531	T	T	T
0.7863782107565761	0.3482673056537418	0.4956204365995179	T	T	T
0.9278645796227200	0.3479943275701214	0.4947890279634539	T	T	T
0.0717149598368957	0.5973893531757557	0.4973672294505001	T	T	T
0.2131940196524440	0.5991268700702149	0.4998471827337231	T	T	T
0.3564250298535480	0.5998541937671334	0.5000372629939158	T	T	T
0.5058004822454277	0.5997225185749180	0.5034171265684017	T	T	T
0.6450435821098688	0.5997239107925119	0.4999974556754551	T	T	T
0.7868327545451003	0.5987595450455333	0.4970853880452524	T	T	T
0.9286348065298066	0.5978678223786091	0.4962656555982611	T	T	T
0.0702772956499433	0.8475388860763092	0.4916835623361748	T	T	T
0.2134621596426440	0.8481835079338842	0.4908296858317650	T	T	T
0.3566482622122712	0.8487354545288869	0.4907257202539355	T	T	T
0.5004122668167488	0.8487171399321592	0.4915945631492126	T	T	T
0.6441345429920511	0.8487619231134386	0.4919004527662650	T	T	T
0.7864860677921232	0.8483471246404417	0.4921584532235705	T	T	T
0.9278149405491476	0.8477036100155156	0.4922139124049305	T	T	T
0.9980925829207781	0.0079797758146395	0.4968192590594160	T	T	T
0.1409653060544738	0.0076237802381379	0.4962478452804102	T	T	T
0.2844411283516112	0.0084851928136471	0.4964055456134128	T	T	T
0.4281304712339039	0.0088000401816032	0.4971129847944231	T	T	T
0.5720766554818589	0.0085238649094162	0.4971151998725631	T	T	T
0.7151622708040266	0.0088745673109656	0.4970636361638875	T	T	T
0.8564537847977054	0.0086940363943402	0.4971876832096603	T	T	T
0.9982485173366139	0.2577517570313221	0.4985716724440576	T	T	T
0.1378619630347370	0.2553445621743295	0.4984303732973237	T	T	T
0.2748232344331684	0.2554346949777583	0.4995109913198885	T	T	T
0.4356564043015716	0.2562290541329353	0.5026090782837110	T	T	T
0.5706961836184928	0.2572333409552406	0.5028301998730292	T	T	T
0.7136420033357446	0.2583786401964277	0.4995558614182889	T	T	T
0.8563918421684071	0.2586983230256522	0.4989828958123040	T	T	T
0.0001262365111431	0.5085067264049736	0.5037258805674253	T	T	T
0.1427737619367180	0.5082299487445238	0.5065517223763940	T	T	T
0.2803824596265644	0.5121899935710494	0.5095733671835316	T	T	T
0.4352334341590522	0.5131397753925223	0.5163140279742865	T	T	T
0.5765896358653788	0.5127336966409942	0.5130581279000310	T	T	T
0.7157489748236734	0.5104589203009360	0.5061658634809947	T	T	T
0.8577395705420409	0.5089570582382102	0.5032678228433202	T	T	T
0.9993878283991704	0.7586109949462945	0.5002083121963731	T	T	T
0.1419266798077660	0.7592902699958846	0.4999953303816003	T	T	T
0.2852561865304923	0.7608784176657997	0.4991883102857471	T	T	T
0.4290910087477184	0.7611413437257383	0.5001408513131752	T	T	T
0.5731218782288434	0.7607987418284657	0.5009654466922278	T	T	T
0.7160011464965034	0.7600184758127865	0.5004781911496682	T	T	T
0.8575279210874156	0.7590760468526234	0.5002452868251959	T	T	T
0.1400988790563691	0.0721615594210061	0.4239241961498327	T	T	T
0.2849744659600417	0.0742115908679550	0.4247592726110951	T	T	T
0.4278064917324312	0.0742750009763346	0.4255554767979053	T	T	T
0.5716661285814253	0.0737136449278519	0.4252058816548950	T	T	T

0.7140927763382163	0.0741687938862041	0.4251355490836725	T	T	T
0.8558832982161290	0.0736527607077012	0.4250087025007767	T	T	T
0.9975075591413751	0.0726613035220642	0.4246665153731860	T	T	T
0.1382407709940612	0.3229606516701162	0.4273080652885474	T	T	T
0.2827851966827695	0.3230906481622160	0.4298065921356774	T	T	T
0.4310976930122450	0.3285668833806680	0.4357390460494980	T	T	T
0.5702459134656918	0.3255892402927657	0.4331700097473927	T	T	T
0.7161778718106947	0.3258155574580117	0.4286486184774512	T	T	T
0.8564872623361154	0.3264794572667170	0.4289233863447118	T	T	T
0.9981536398429444	0.3258531306499380	0.4284507068771803	T	T	T
0.1441135516558149	0.5717424844117462	0.4335584908929312	T	T	T
0.2857476698019749	0.5731355352204729	0.4352475050783273	T	T	T
0.4323237796417272	0.5702260005529649	0.4399612398533908	T	T	T
0.5730477125236122	0.5726194464733128	0.4364012720686909	T	T	T
0.7139336132180267	0.5737886248153213	0.4332973662461594	T	T	T
0.8570596708232404	0.5737052757982022	0.4311576118833877	T	T	T
0.0008356075940688	0.5731462285542571	0.4316659287650399	T	T	T
0.1414998006936267	0.8220245448778869	0.4265437503047335	T	T	T
0.2850808733076036	0.8231844742603200	0.4256930890310777	T	T	T
0.4290194933293549	0.8229811425001795	0.4264691007116382	T	T	T
0.5723703644607212	0.8228705708120196	0.4271982675599234	T	T	T
0.7151580744217791	0.8228386864543984	0.4270406028319825	T	T	T
0.8570253972386875	0.8221976048019058	0.4270206611679574	T	T	T
0.9988355360214334	0.8217749419206780	0.4270759832185173	T	T	T
0.0681391157322644	0.1952744430518109	0.4452768157867002	T	T	T
0.2098798255814506	0.1947972258084551	0.4445484814008289	T	T	T
0.3565057737263425	0.1992832688794412	0.4510333232237564	T	T	T
0.5010678106498319	0.1968867380212308	0.4473902370320881	T	T	T
0.6418928429972610	0.1971200366875328	0.4477694492640616	T	T	T
0.7849656232513358	0.1974266335750580	0.4468077766478166	T	T	T
0.9269098747599878	0.1968574922374287	0.4461146759030549	T	T	T
0.0721199591758142	0.4471490491718871	0.4531546323970255	T	T	T
0.2167851383395722	0.4475963584233825	0.4581362083666542	T	T	T
0.3602268617782001	0.4527299735694978	0.4678146586729482	T	T	T
0.5034869650126460	0.4491707224036566	0.4704119596773618	T	T	T
0.6417173765514963	0.4485195441440839	0.4580445736684026	T	T	T
0.7850243978679236	0.4479697818341214	0.4528175187661805	T	T	T
0.9287111181891273	0.4470687818757586	0.4516817808587905	T	T	T
0.0711928222281947	0.6950455471401210	0.4502838069922743	T	T	T
0.2135949099143583	0.6955435196669522	0.4505234493291119	T	T	T
0.3576798562922248	0.6958609830024534	0.4509112740598312	T	T	T
0.5018917795988195	0.6953910330006713	0.4527106626958036	T	T	T
0.6440283653348747	0.6963688681579865	0.4512421757550460	T	T	T
0.7863124485327954	0.6959527951609313	0.4499509133019892	T	T	T
0.9285670340365384	0.6953645406599551	0.4497660316229554	T	T	T
0.0698235089522044	0.9452778952783337	0.4455603816609937	T	T	T
0.2132595913159082	0.9461272647205837	0.4450301641125883	T	T	T
0.3567917480529288	0.9470478123255944	0.4452585755566434	T	T	T
0.5000430555679670	0.9467830068711749	0.4460735585296415	T	T	T
0.6435607056290059	0.9466412722255503	0.4458874804693148	T	T	T
0.7858749765557507	0.9461530897966791	0.4457801090766579	T	T	T
0.9274491361110057	0.9454411055458674	0.4458911340521987	T	T	T
0.9984006197373430	0.1585263915199285	0.5419408573284488	T	T	T
0.1398648272668969	0.1567628761973516	0.5417845580766730	T	T	T

0.2809502091036454	0.1565767307791049	0.5421056377729526	T	T	T
0.4290987955853995	0.1561962559738392	0.5434455745168135	T	T	T
0.5716051838401774	0.1566876799554132	0.5439329126313606	T	T	T
0.7137341227480370	0.1593059027685830	0.5429721955214664	T	T	T
0.8564943086017448	0.1596219890592422	0.5425080577787935	T	T	T
0.9994578458615290	0.4087051591696585	0.5476079897170528	T	T	T
0.1425715785780095	0.4063368002354443	0.5495615540777843	T	T	T
0.7136270058855165	0.4103826788873420	0.5501888247481848	T	T	T
0.8569310626977961	0.4094342414545782	0.5478508643034705	T	T	T
0.9998287254856630	0.6611998160185866	0.5470028670514926	T	T	T
0.1411316300496362	0.6627895423175142	0.5483830194884849	T	T	T
0.2835037563582250	0.6656773482990498	0.5466986055994755	T	T	T
0.4303675799138083	0.6652363808568545	0.5470872048529718	T	T	T
0.5756529853353404	0.6656703965014574	0.5503884160657364	T	T	T
0.7173715819484677	0.6638796043706443	0.5482015226460963	T	T	T
0.8583914100322799	0.6617276281670661	0.5470682424452491	T	T	T
0.9993329038791672	0.9102953400868377	0.5426514709606465	T	T	T
0.1420337394037846	0.9101464499906025	0.5419300273208033	T	T	T
0.2848899802908587	0.9107788922247494	0.5414393932863475	T	T	T
0.4279219024390619	0.9107721284958628	0.5418206506188502	T	T	T
0.5718991441894705	0.9109122035899996	0.5423550163179000	T	T	T
0.7149728019157240	0.9113028634632551	0.5426386381465734	T	T	T
0.8571059021279726	0.9109777335393260	0.5431196206507531	T	T	T
0.0701063525214030	0.0317015066225365	0.5618289282855407	T	T	T
0.2126367597592304	0.0315223721921672	0.5615812733858969	T	T	T
0.3559096888584628	0.0319241567535714	0.5621196791757905	T	T	T
0.5000601213457617	0.0315600505531942	0.5624214075663160	T	T	T
0.6434698579977522	0.0322125386230678	0.5624338426446919	T	T	T
0.7857985852194554	0.0331452393963939	0.5627753240568056	T	T	T
0.9278787967017358	0.0327326697878109	0.5625275813952209	T	T	T
0.0683597076040270	0.2790949526893583	0.5650703404632144	T	T	T
0.2072921725954720	0.2782666961523757	0.5662306346551923	T	T	T
0.3537273942365761	0.2700506564632879	0.5629022218712931	T	T	T
0.5010442764651130	0.2748526951465909	0.5717190321322004	T	T	T
0.6431593492765858	0.2774250729860800	0.5686902716736261	T	T	T
0.7858414023668815	0.2802537281345142	0.5653586978185030	T	T	T
0.9277245882858554	0.2802941209180524	0.5648838348664444	T	T	T
0.0696900675654363	0.5334859714599502	0.5701682862153780	T	T	T
0.2081981661420685	0.5369696405110036	0.5758940570331783	T	T	T
0.3559522269891408	0.5691888189110172	0.5842584527509360	T	T	T
0.5049584663163192	0.5491356172030057	0.5846499345508595	T	T	T
0.6554496951801403	0.5427815744795929	0.5765584868850900	T	T	T
0.7898514211907026	0.5343086299220120	0.5697868343069893	T	T	T
0.9290171744115964	0.5333726470179864	0.5687766523954123	T	T	T
0.0707835354801769	0.7849999226129643	0.5649719605341550	T	T	T
0.2136297337306609	0.7859320066955819	0.5642434091617906	T	T	T
0.3566526261393094	0.7866242271378775	0.5642552868215193	T	T	T
0.5007238542219917	0.7866735629229432	0.5651227986397734	T	T	T
0.6447510589261268	0.7867109561105844	0.5654384650244320	T	T	T
0.7870758871958075	0.7855618976473524	0.5655401459345744	T	T	T
0.9287015026724066	0.7844448455471122	0.5653563864599042	T	T	T
0.3023955056563454	0.3839173730757249	0.6127833300872108	T	T	T
0.4276415187142719	0.3901788620237075	0.6253445189812050	T	T	T
0.5504088567187395	0.3930309557313776	0.6390998843993585	T	T	T

```
  0.2732456442715424  0.4076942217678290  0.5334492509941197   T   T   T
  0.3833698301879250  0.4137974549031992  0.5487073539248615   T   T   T
  0.4945549484362279  0.4209104817791681  0.5584167610248840   T   T   T
  0.6127811659875075  0.3809295528385037  0.5711000310567627   T   T   T
  0.3037804013072483  0.4841500599655510  0.5929419462137032   T   T   T
  0.4163940608938323  0.4914326947429396  0.6037918585386376   T   T   T
  0.5794231924362300  0.4766127516626554  0.5952526395658593   T   T   T
== Embedded cluster I-Pd_2k+1 k=4
   1.00000000000000
     24.6586885454000111    0.0000000000000000    0.0000000000000000
      0.0000000000000000   25.2576961516000082    0.0000000000000000
      0.0000000000000000    0.0000000000000000   30.0000000000007034
   W     Te    Pd
    56   112     9
Selective dynamics
Direct
  0.0692824140143865  0.0953539563925456  0.4884574342432239   T   T   T
  0.2133731307073940  0.0978051910236439  0.4893812403918210   T   T   T
  0.3573474061934497  0.0963819344847218  0.4876798909424730   T   T   T
  0.5014002077000096  0.0977344262036571  0.4891030921960347   T   T   T
  0.6455331318752576  0.0953446998211809  0.4881710654198418   T   T   T
  0.7868619947910721  0.0953637123346442  0.4889162548458879   T   T   T
  0.9278497495288850  0.0953578247243351  0.4891206482572471   T   T   T
  0.0638126983774996  0.3426616760001487  0.4959852917172770   T   T   T
  0.2123263182265858  0.3384141095986183  0.4974620588500491   T   T   T
  0.3574689291268843  0.3454728076935835  0.5038522665532889   T   T   T
  0.5028233445416294  0.3386738025457625  0.4975502202698672   T   T   T
  0.6506721447648867  0.3427563453521268  0.4958308008466046   T   T   T
  0.7877261466524181  0.3463189723818981  0.4978218334814221   T   T   T
  0.9269404861639430  0.3462912774150385  0.4978563315586761   T   T   T
  0.0692010342334143  0.5983817821935663  0.5027697745292566   T   T   T
  0.2138849581033672  0.5992810664212909  0.5030414102745461   T   T   T
  0.3572809871582987  0.6024405397871340  0.5067505428804047   T   T   T
  0.5005137616867126  0.5993862099426649  0.5030539229767735   T   T   T
  0.6452973477491254  0.5984635109198057  0.5029088643123854   T   T   T
  0.7859801485863119  0.5966253679163516  0.5007108185042314   T   T   T
  0.9285539690269011  0.5967126960394217  0.5007724402933452   T   T   T
  0.0703970024300455  0.8475070588768763  0.4906834552361936   T   T   T
  0.2132040788921104  0.8488179656784158  0.4897748940860527   T   T   T
  0.3571206634769841  0.8492404438173781  0.4893298012311683   T   T   T
  0.5011664003493417  0.8488430303550336  0.4895721811669906   T   T   T
  0.6442219448599924  0.8474572632946481  0.4905147703822319   T   T   T
  0.7862015988398345  0.8464637798710941  0.4915653996547983   T   T   T
  0.9282518383319308  0.8464828420545548  0.4916765099742265   T   T   T
  0.9987651532445497  0.0067733707105799  0.4952006185649870   T   T   T
  0.1416496552662035  0.0082297022691499  0.4950358838362533   T   T   T
  0.2850200026295630  0.0089124821588092  0.4945112022210838   T   T   T
  0.4294923429286795  0.0088957831649002  0.4943491315774898   T   T   T
  0.5730681247189208  0.0082655730671135  0.4947009200292571   T   T   T
  0.7158873028019019  0.0067510234876441  0.4948807986416128   T   T   T
  0.8573306302491515  0.0064403874115582  0.4953315307846645   T   T   T
  0.9957157237890625  0.2547623207139083  0.4993834993791174   T   T   T
  0.1334305894467717  0.2555781544209283  0.4998944799278447   T   T   T
  0.2925542618549675  0.2566414573304084  0.5022122909773558   T   T   T
```

0.4227368070842866	0.2566981155716107	0.5021927833992056	T	T	T
0.5812156574874933	0.2555819346829834	0.4997192788356810	T	T	T
0.7187969960673273	0.2548594239107634	0.4992718176309507	T	T	T
0.8572358474617849	0.2562933628623380	0.4994269912889792	T	T	T
0.9997623658554451	0.5077155313695483	0.5098634263077043	T	T	T
0.1351799484593155	0.5108639679878391	0.5130095423295311	T	T	T
0.2953207284862642	0.5124390240608587	0.5179624012449540	T	T	T
0.4191165969338075	0.5124452540886903	0.5179293583557192	T	T	T
0.5794500651577638	0.5108818071859351	0.5130282104764907	T	T	T
0.7146968488134918	0.5077114764896553	0.5098103008060458	T	T	T
0.8573575073314946	0.5076673386306411	0.5081124613653689	T	T	T
0.9991533620596420	0.7584715564244211	0.5009831701368110	T	T	T
0.1417069165023082	0.7604396162783696	0.4999174087195540	T	T	T
0.2849092637181498	0.7619970295249620	0.4998495228826047	T	T	T
0.4294133329519762	0.7620491158460377	0.4998278375699195	T	T	T
0.5726800468465538	0.7605156054486704	0.4999077384933235	T	T	T
0.7154721686863703	0.7583877038267232	0.5009388290092556	T	T	T
0.8572449630767037	0.7576911138875139	0.5013901674101839	T	T	T
0.1423034980259411	0.0741268748111637	0.4235075112675181	T	T	T
0.2844356257888616	0.0749887289604151	0.4231061842783353	T	T	T
0.4300874231730677	0.0750264836514714	0.4229809590121222	T	T	T
0.5724386367927700	0.0742694752495410	0.4231931649176369	T	T	T
0.7163319561201204	0.0718436512812860	0.4226919832423772	T	T	T
0.8575266722819417	0.0711180469293228	0.4231964483575574	T	T	T
0.9985306707222796	0.0717907921018352	0.4229821278132028	T	T	T
0.1403013657765171	0.3248745628662053	0.4312888625193480	T	T	T
0.2883001858527195	0.3282620163428973	0.4349145158022787	T	T	T
0.4267661884548833	0.3284341508215968	0.4349943839070626	T	T	T
0.5743704009205661	0.3248803457005396	0.4310933197800927	T	T	T
0.7190111227120300	0.3235225071749818	0.4292615791528713	T	T	T
0.8573669270294451	0.3264483043273151	0.4306461922688303	T	T	T
0.9956075851520468	0.3233892754744259	0.4292864354970386	T	T	T
0.1423566051144246	0.5711210873173739	0.4388224958982161	T	T	T
0.2876962314108868	0.5701297957246817	0.4417166596934295	T	T	T
0.4268925536838180	0.5701423745382010	0.4416898061743010	T	T	T
0.5721595410767149	0.5713214824419527	0.4389578046741530	T	T	T
0.7140925741897370	0.5709101761204065	0.4365933317821484	T	T	T
0.8572607479007105	0.5717239144289668	0.4356608710645615	T	T	T
0.0002777823193936	0.5708299476392737	0.4365401636105877	T	T	T
0.1416796110406295	0.8211429343490743	0.4255669710303482	T	T	T
0.2850095639656691	0.8223396767204801	0.4251972329215591	T	T	T
0.4292070103885674	0.8222778780285880	0.4250995688187519	T	T	T
0.5727488327793773	0.8211227914645808	0.4255089140097007	T	T	T
0.7155347099291128	0.8194928515620794	0.4263679447859248	T	T	T
0.8572579100566501	0.8191239458998905	0.4271341290054533	T	T	T
0.9989482981789073	0.8198481737358343	0.4265596175076483	T	T	T
0.0678208302396544	0.1951634773932249	0.4447660323783040	T	T	T
0.2140355534734398	0.1995670975398560	0.4498686512025136	T	T	T
0.3575576566935878	0.1977459503404055	0.4446513806902624	T	T	T
0.5009122990897417	0.1994623853583662	0.4495676408110329	T	T	T
0.6470089855941353	0.1952066852739373	0.4445731279282486	T	T	T
0.7877098679520345	0.1948024564336957	0.4450432497441865	T	T	T
0.9268129926452576	0.1947734621985964	0.4451009328629385	T	T	T
0.0730991560954366	0.4468943326555962	0.4601865271062333	T	T	T

0.2172048300920126	0.4528380930945593	0.4721927907446453	T	T	T
0.3571417670295056	0.4499887250649534	0.4655938932517554	T	T	T
0.4973813664977671	0.4529068337030728	0.4722481041179309	T	T	T
0.6414026303553341	0.4471102256240019	0.4599795541360900	T	T	T
0.7854409152699235	0.4466312565781237	0.4570517907783772	T	T	T
0.9292788003292068	0.4466219406065036	0.4569749815996087	T	T	T
0.0703541207674038	0.6938478317119010	0.4521452073599066	T	T	T
0.2137187297468000	0.6945210719606392	0.4525924588637572	T	T	T
0.3572045551806027	0.6957053019869659	0.4531473115110020	T	T	T
0.5005921038177297	0.6946762606724125	0.4526124601588059	T	T	T
0.6440651673001612	0.6937588435597045	0.4523440898495550	T	T	T
0.7862186101998000	0.6931014742292180	0.4522607384768036	T	T	T
0.9283690134694105	0.6931510848371244	0.4523776898380837	T	T	T
0.0707653545253214	0.9450148512488007	0.4440434235364462	T	T	T
0.2134582792103721	0.9467048338181943	0.4432389042308336	T	T	T
0.3571228270676263	0.9472047588365293	0.4431750436979040	T	T	T
0.5011094119780536	0.9467223374410279	0.4430830233719418	T	T	T
0.6439454470804423	0.9449176800261669	0.4437561825212292	T	T	T
0.7865182525650232	0.9434228258343310	0.4440252285061753	T	T	T
0.9282053743667259	0.9434570945792695	0.4442808486989577	T	T	T
0.9985762760702563	0.1557273372367355	0.5418945774319741	T	T	T
0.1392517771301038	0.1561478801610681	0.5414417803463004	T	T	T
0.2875117151781507	0.1560644792311650	0.5417628196107283	T	T	T
0.4276429982371484	0.1560450906292250	0.5416063252741871	T	T	T
0.5755079599477467	0.1561333820858013	0.5412176478074846	T	T	T
0.7160723955763602	0.1557495725691181	0.5416891879157141	T	T	T
0.8572584164582694	0.1565130666818870	0.5417807438964465	T	T	T
0.0014247563082262	0.4053881815696874	0.5525711115893047	T	T	T
0.7130622347246006	0.4055632872907530	0.5524612206467462	T	T	T
0.8572343900466939	0.4070073746048324	0.5518594902154629	T	T	T
0.9980899405739700	0.6629961780614186	0.5509298856441598	T	T	T
0.1400294964722916	0.6657819215446080	0.5484739255012798	T	T	T
0.2844000574777272	0.6685187349098530	0.5502036015244396	T	T	T
0.4301102024328130	0.6685799877896595	0.5502206776477599	T	T	T
0.5743873112602765	0.6658541627654703	0.5485765292416580	T	T	T
0.7166566873951455	0.6629529644721962	0.5509685736126684	T	T	T
0.8571651675930632	0.6614794527994443	0.5500962777628846	T	T	T
0.9996353681118347	0.9099464390695096	0.5414616739037035	T	T	T
0.1422146533988038	0.9109631590789011	0.5404604837004281	T	T	T
0.2853629616153435	0.9116248860771005	0.5395606938388700	T	T	T
0.4290963798618103	0.9115644921359697	0.5393348158580130	T	T	T
0.5723533316373941	0.9110773704038913	0.5401053937166210	T	T	T
0.7149646119295550	0.9099784076651444	0.5412768542365219	T	T	T
0.8572420197472750	0.9097279593798258	0.5418571119657356	T	T	T
0.0703922665469111	0.0308830670962748	0.5605937902676220	T	T	T
0.2136741214539813	0.0317390782963440	0.5602560231974708	T	T	T
0.3573617593193171	0.0314620002274029	0.5595894487542118	T	T	T
0.5010656965923845	0.0317157128331421	0.5599634247681827	T	T	T
0.6443269124534455	0.0308229121964246	0.5602853892490144	T	T	T
0.7863573508494927	0.0303666650061429	0.5608268073481700	T	T	T
0.9281523410374745	0.0302904512189308	0.5610424090229209	T	T	T
0.0657896577837941	0.2770064022297379	0.5669678526071407	T	T	T
0.2119785054784123	0.2681938232330593	0.5635229138782556	T	T	T
0.3576116621517971	0.2768464078853741	0.5722416917611942	T	T	T

```
  0.5030645821511991  0.2683415624859209  0.5636198632501149   T   T   T
  0.6487932502784168  0.2771094090812074  0.5668184811373939   T   T   T
  0.7876203639545679  0.2768155827579163  0.5666555473017493   T   T   T
  0.9267485143296631  0.2767610020745500  0.5666881428541914   T   T   T
  0.0649895859455438  0.5365508443964743  0.5793328270413449   T   T   T
  0.2108044547188715  0.5700979509199723  0.5874274849498492   T   T   T
  0.3571981596808338  0.5548766384378888  0.5895397322798438   T   T   T
  0.5036510298426874  0.5701810752009500  0.5873701992020001   T   T   T
  0.6495570784147464  0.5366320572833336  0.5793825823321019   T   T   T
  0.7871673507202208  0.5328336857007004  0.5738635914203448   T   T   T
  0.9274279377521106  0.5329790580255490  0.5739569715606715   T   T   T
  0.0706875583309957  0.7864996651744084  0.5649525961965062   T   T   T
  0.2131851172034989  0.7884126691763437  0.5642586985427259   T   T   T
  0.3571878202126394  0.7890272872043489  0.5637995788101461   T   T   T
  0.5012100368831411  0.7887040565251827  0.5641471631661442   T   T   T
  0.6438648301341365  0.7865582177391576  0.5648445426993605   T   T   T
  0.7862337517880802  0.7852213727037635  0.5658398020097615   T   T   T
  0.9282544515095601  0.7852407018487156  0.5658952580143704   T   T   T
  0.1687889104776962  0.3832315021694166  0.6147036234241359   T   T   T
  0.2895983046188495  0.3937818598499668  0.6283484600286457   T   T   T
  0.4255527519899373  0.3939566281192656  0.6285141636146181   T   T   T
  0.5462293632313566  0.3834487366677181  0.6150997261192774   T   T   T
  0.1335857869974580  0.4055507235159179  0.5361427510831454   T   T   T
  0.2464956380526967  0.4110897547436088  0.5507827115190250   T   T   T
  0.3575670887916269  0.4257258264925622  0.5593862013944512   T   T   T
  0.4686812941678147  0.4112699440935813  0.5509382538337344   T   T   T
  0.5815856597063788  0.4057596859232440  0.5365514392706574   T   T   T
  0.1624898931285239  0.4827099685157430  0.5951657412813646   T   T   T
  0.2735820786468179  0.4932048744150830  0.6045807551666852   T   T   T
  0.4409687242437558  0.4933151890153121  0.6045655002611120   T   T   T
  0.5521263463443686  0.4828881334712028  0.5953019989825119   T   T   T
== Embedded cluster I-Pd_3k+1 k=1
   1.00000000000000
    24.6586885454000111    0.0000000000000000    0.0000000000000000
     0.0000000000000000   25.2576961516000082    0.0000000000000000
     0.0000000000000000    0.0000000000000000   30.0000000000007034
   W    Te   Pd
    56   112     4
Selective dynamics
Direct
  0.0713773072603434  0.0985239422589886  0.4927750814872652   T   T   T
  0.2131213782719345  0.0982832199597374  0.4923684152907744   T   T   T
  0.3556809280367887  0.0981168079207426  0.4922854005715350   T   T   T
  0.4996104299715196  0.0979895812271176  0.4928047788407961   T   T   T
  0.6431027671237129  0.0980906718973082  0.4927650552725549   T   T   T
  0.7870792317845353  0.0981928935982107  0.4923893558168357   T   T   T
  0.9295575056154159  0.0984760883773269  0.4926375553100964   T   T   T
  0.0713367382790758  0.3490174933695222  0.4934377088170848   T   T   T
  0.2129896043748227  0.3488157544105446  0.4932626420258752   T   T   T
  0.3538578714762479  0.3480036666780017  0.4934615955392619   T   T   T
  0.4926607233649400  0.3451248441315853  0.4947407719245612   T   T   T
  0.6498744878057796  0.3453314063847717  0.4947831014314274   T   T   T
  0.7889398366417362  0.3482908679375041  0.4936860136770670   T   T   T
  0.9297074593265302  0.3489692942428906  0.4933220829030325   T   T   T
```

0.0715934555197942	0.5987466361793515	0.4934950008052214	T	T	T
0.2138765295494514	0.5988618397109309	0.4935947752720666	T	T	T
0.3563351995699457	0.5995631757962774	0.4936275462813586	T	T	T
0.4999765699816310	0.6003632674197115	0.4940678578366147	T	T	T
0.6430590347405245	0.6003360758352950	0.4942064897558371	T	T	T
0.7867550719633046	0.5997192832165058	0.4934852581007936	T	T	T
0.9294009562263977	0.5990395203926868	0.4934277305352977	T	T	T
0.0715340419502983	0.8484841757563857	0.4931029852256263	T	T	T
0.2137330464179330	0.8485817693164712	0.4930159569329185	T	T	T
0.3562872637411786	0.8489242909488333	0.4926169116778852	T	T	T
0.4995602284387654	0.8491900285780771	0.4922991981074602	T	T	T
0.6433653029450442	0.8492015643843871	0.4923255182867825	T	T	T
0.7868370043263299	0.8490066912620151	0.4925307807666710	T	T	T
0.9293077694014791	0.8487227022039959	0.4930027306729636	T	T	T
0.0004217940956914	0.0092656264273087	0.4990295478882875	T	T	T
0.1423243567391271	0.0091405275844316	0.4989325929836839	T	T	T
0.2845486998579612	0.0093014717450056	0.4988587087722517	T	T	T
0.4276285219217945	0.0095040819785421	0.4990097932448779	T	T	T
0.5713700827195957	0.0094677940339575	0.4992735529953068	T	T	T
0.7151969997679897	0.0095761523898944	0.4990308416441292	T	T	T
0.8582836604675741	0.0094049924018023	0.4989772783798872	T	T	T
0.0003789128765865	0.2595727632678121	0.4994411599514487	T	T	T
0.1422803956879367	0.2594992279439927	0.4994602561010621	T	T	T
0.2840812102679334	0.2589234096839459	0.4988432297356770	T	T	T
0.4270374651475496	0.2576889018482966	0.4990971550891137	T	T	T
0.5710786618212310	0.2601075623901113	0.5018279547718077	T	T	T
0.7157720190482421	0.2579270153823159	0.4993218000180914	T	T	T
0.8583542744592564	0.2591219271184171	0.4990723788182548	T	T	T
0.0004142411540114	0.5097350824277355	0.4999680036217110	T	T	T
0.1426613336279408	0.5095863395486070	0.5000480328866262	T	T	T
0.2846654460528603	0.5098442044727821	0.5002957503290417	T	T	T
0.4271655992003009	0.5113702087503273	0.5014355754673997	T	T	T
0.5713859204317453	0.5135496118888652	0.5020348874957483	T	T	T
0.7155285446649794	0.5115200567580758	0.5014186743296455	T	T	T
0.8583603590322043	0.5101484381452923	0.5001662425311729	T	T	T
0.0004349097835668	0.7595261067445462	0.4997713441229666	T	T	T
0.1427314406851487	0.7594506468562151	0.4998548124712627	T	T	T
0.2850463295982378	0.7598671515187193	0.4997081044456561	T	T	T
0.4278357698657773	0.7604463097617247	0.4993699324992262	T	T	T
0.5715090333301184	0.7608084628498067	0.4993394230268612	T	T	T
0.7153156528595813	0.7605568601954645	0.4994587236809024	T	T	T
0.8582048358211342	0.7599757556452610	0.4996502927637783	T	T	T
0.1420048035783875	0.0742822514071604	0.4270087190270853	T	T	T
0.2845597662594356	0.0745061167149361	0.4268983895988344	T	T	T
0.4280077669234481	0.0750751779586323	0.4273286814899274	T	T	T
0.5714042611303635	0.0749681096946477	0.4275533032967986	T	T	T
0.7147855774450070	0.0753035343456925	0.4273965642194107	T	T	T
0.8582923513036308	0.0744964034257238	0.4270682136973755	T	T	T
0.0004966165385723	0.0746160332237444	0.4271382390881139	T	T	T
0.1419191829356560	0.3247113785628069	0.4276592487158598	T	T	T
0.2835056749367931	0.3248019871005005	0.4274020312806177	T	T	T
0.4252428472330594	0.3234084798015157	0.4273856078092901	T	T	T
0.5713464227848246	0.3252884845045916	0.4315607093436744	T	T	T
0.7176804634814626	0.3235855670571817	0.4277172103213312	T	T	T

0.8590800988082243	0.3249049453978292	0.4275443263666137	T	T	T
0.0006677490871217	0.3248415296343698	0.4276728264530115	T	T	T
0.1429013981255607	0.5747399716569471	0.4281253868719111	T	T	T
0.2854500697749114	0.5748648092938939	0.4282939217489346	T	T	T
0.4285736877004455	0.5752247057188296	0.4288398242356936	T	T	T
0.5715917308062454	0.5767329075228758	0.4288512616647133	T	T	T
0.7143481974930691	0.5752271829361516	0.4288400761815749	T	T	T
0.8577673702739360	0.5752370491442270	0.4281328865691853	T	T	T
0.0004915963272926	0.5748084487354517	0.4279555249035549	T	T	T
0.1426960405388149	0.8240855221735156	0.4276784191197874	T	T	T
0.2849661833737950	0.8244684256236461	0.4275099934660357	T	T	T
0.4279502340657193	0.8250582800387795	0.4272441417860099	T	T	T
0.5715719201482198	0.8252496642784111	0.4271936951311666	T	T	T
0.7150359874838980	0.8250047694172483	0.4272405912898799	T	T	T
0.8581923913949604	0.8246474887597868	0.4274285959721585	T	T	T
0.0003885300967457	0.8243313998822466	0.4276627524407982	T	T	T
0.0712408812487980	0.1969280516968004	0.4477917392776100	T	T	T
0.2124080785717572	0.1968425911398793	0.4473050244205672	T	T	T
0.3551427009270302	0.1967549809515272	0.4472198150599383	T	T	T
0.5001204636817079	0.1969230345679805	0.4487365524198851	T	T	T
0.6424921690689827	0.1973391739183887	0.4489356656640150	T	T	T
0.7874868943806772	0.1968321546629864	0.4473603353200655	T	T	T
0.9298354190109545	0.1969533650275608	0.4474935362391652	T	T	T
0.0714854890289418	0.4472252456386417	0.4484537771078227	T	T	T
0.2134585044071704	0.4472869271287151	0.4485112690153824	T	T	T
0.3559755987498884	0.4478392941044277	0.4495278660660081	T	T	T
0.4989836079629401	0.4477089005749684	0.4538639371626185	T	T	T
0.6439483935664104	0.4478398647771453	0.4537234082437767	T	T	T
0.7870200389605364	0.4480539881450207	0.4494831810626858	T	T	T
0.9294827289979071	0.4475236693937342	0.4484566124295729	T	T	T
0.0716150152564705	0.6971626618212241	0.4483856473058661	T	T	T
0.2140071096374887	0.6973109903916114	0.4484618702627010	T	T	T
0.3566378161604964	0.6979265110060228	0.4483120007769307	T	T	T
0.4998165840675644	0.6986484653212084	0.4481976827739154	T	T	T
0.6433879428266590	0.6986447476115973	0.4484545209239308	T	T	T
0.7864831573245160	0.6981909422295145	0.4482549250618876	T	T	T
0.9292352282006993	0.6974313718374721	0.4483584952573756	T	T	T
0.0714162315836884	0.9466809054953564	0.4475229977716519	T	T	T
0.2135244906007632	0.9467749406605040	0.4474871263646516	T	T	T
0.3565047894518973	0.9473665473969725	0.4474806958165393	T	T	T
0.4997117167579034	0.9479930686743011	0.4476557629428277	T	T	T
0.6431333350702599	0.9479285356311014	0.4476989103287269	T	T	T
0.7864692061443630	0.9475209889901609	0.4474998066068907	T	T	T
0.9291099627554744	0.9469746645766176	0.4475150270727758	T	T	T
0.0004370826439188	0.1608901149413555	0.5443447469916893	T	T	T
0.1424924800711102	0.1606547155136991	0.5443145464781218	T	T	T
0.2843685444972082	0.1604929883784606	0.5436962013052900	T	T	T
0.4273818017203104	0.1596476342692355	0.5441977784125329	T	T	T
0.5712261407421388	0.1596460512584139	0.5445825359458047	T	T	T
0.7151389885702263	0.1597202021448697	0.5441744316208731	T	T	T
0.8581252951037166	0.1605691683480729	0.5439235670622615	T	T	T
0.0004467277360102	0.4113855185281238	0.5450719474097557	T	T	T
0.1423634204056619	0.4111753251304502	0.5451047184639238	T	T	T
0.2838721283361729	0.4107724654257554	0.5453091998315506	T	T	T

```
  0.4250228526844993  0.4114502115481269  0.5452332965216626   T   T   T
  0.7176523992818794  0.4116861690371518  0.5451531845031663   T   T   T
  0.8590888473986138  0.4111985374799053  0.5453509235712406   T   T   T
  0.0006273364373251  0.6612828720410002  0.5449247479414877   T   T   T
  0.1427815196340755  0.6611354137916818  0.5450042773925245   T   T   T
  0.2850444463570305  0.6616344874279602  0.5447868077441462   T   T   T
  0.4279828508569190  0.6624566182754702  0.5444725447420384   T   T   T
  0.5715931622939114  0.6630947839884641  0.5444324107240134   T   T   T
  0.7151768639818766  0.6624308580502539  0.5445187817580591   T   T   T
  0.8582458482178629  0.6617151150692115  0.5446995274251005   T   T   T
  0.0002760307612761  0.9109922495087103  0.5443708439891699   T   T   T
  0.1427260565443628  0.9108586745401303  0.5443730402975304   T   T   T
  0.2851890594387800  0.9110992263678511  0.5440719067874358   T   T   T
  0.4280577313840873  0.9110076414963039  0.5437818931615430   T   T   T
  0.5713584029260083  0.9107646243026500  0.5436335358995935   T   T   T
  0.7148634189593321  0.9110626744581579  0.5436627388595818   T   T   T
  0.8577229209550530  0.9110977748459544  0.5441041825708320   T   T   T
  0.0715124535634962  0.0332414346892549  0.5645213177103047   T   T   T
  0.2135164840835949  0.0333007167374866  0.5643152777490682   T   T   T
  0.3561418905188343  0.0332869249279853  0.5642130901655357   T   T   T
  0.4994526908320457  0.0327829857792192  0.5644957300198300   T   T   T
  0.6433356195205667  0.0330062196074591  0.5643813106222335   T   T   T
  0.7866749084848667  0.0332934499771528  0.5642689165954601   T   T   T
  0.9292362332298160  0.0333216802147945  0.5645627516862283   T   T   T
  0.0713520029864943  0.2833188119245306  0.5651198961936108   T   T   T
  0.2135179918601573  0.2831365794361848  0.5648487204161401   T   T   T
  0.3551759633636294  0.2819706747949581  0.5645474315370871   T   T   T
  0.4978004572245471  0.2782702914139679  0.5670737449712618   T   T   T
  0.6444570427027234  0.2782759514779148  0.5669736936015695   T   T   T
  0.7873287376254859  0.2823394557166747  0.5648514042993534   T   T   T
  0.9291259561269385  0.2831805059928217  0.5648852539959156   T   T   T
  0.0714877625628896  0.5339870246304877  0.5655439401488740   T   T   T
  0.2131619298068840  0.5339976024734174  0.5657125508319764   T   T   T
  0.3545471235669386  0.5352446392185679  0.5660248912268732   T   T   T
  0.4961073729923779  0.5376619077017490  0.5679266887023877   T   T   T
  0.6464403218456369  0.5373846797499490  0.5680403766265952   T   T   T
  0.7882946355068702  0.5354725566760707  0.5658682388393302   T   T   T
  0.9297471050488646  0.5342681345354432  0.5655210761667626   T   T   T
  0.0715437868768915  0.7837150302981816  0.5652886817535350   T   T   T
  0.2138905990303897  0.7839016794474140  0.5652425773816632   T   T   T
  0.3567082638654449  0.7844180313194765  0.5648814444168740   T   T   T
  0.4997283429793284  0.7846563791356698  0.5644347811311824   T   T   T
  0.6433596332271636  0.7847632414082306  0.5645014203073735   T   T   T
  0.7866056524447229  0.7845734223233115  0.5648448755870246   T   T   T
  0.9292009889351652  0.7839552152753814  0.5651982417209094   T   T   T
  0.5714521517539602  0.3993775142203856  0.6553533906072829   T   T   T
  0.5090117331843866  0.3788420832394631  0.5894135499396274   T   T   T
  0.6335183371486027  0.3789570814712106  0.5891827364449480   T   T   T
  0.5710188103966964  0.4717196588299852  0.5947448413832356   T   T   T
  0.5711889785118316  0.4111761924253935  0.5211320903827735   T   T   T
== Embedded cluster I-Pd_3k+1 k=2
   1.00000000000000
     24.6586885454000111    0.0000000000000000    0.0000000000000000
      0.0000000000000000   25.2576961516000082    0.0000000000000000
```

```
     0.0000000000000000    0.0000000000000000   30.0000000000007034
   W    Te   Pd
    56   112     7
Selective dynamics
Direct
  0.0709136739877499  0.0986623075110832  0.4925074696904955   T   T   T
  0.2127442815422323  0.0983529702861380  0.4922796080238352   T   T   T
  0.3547145646344779  0.0979046414847083  0.4919970586634100   T   T   T
  0.5001194431199186  0.1014137461122492  0.4933134069634326   T   T   T
  0.6454210284125408  0.0981806905798727  0.4921932947938208   T   T   T
  0.7872792270477166  0.0984601228131206  0.4923407532141301   T   T   T
  0.9291146720183985  0.0986925291276042  0.4925123271348446   T   T   T
  0.0703006477786288  0.3496824042754515  0.4943768543649391   T   T   T
  0.2096859163657432  0.3489180043601232  0.4946737001771526   T   T   T
  0.3500330040515858  0.3469133852639472  0.4933038612847382   T   T   T
  0.4993792103934575  0.3284711341968131  0.4804397259186063   T   T   T
  0.6506885898056802  0.3471772633763777  0.4932772250801778   T   T   T
  0.7903283417772277  0.3490743680877367  0.4946205856967041   T   T   T
  0.9297880672579560  0.3497403525745687  0.4943662823574733   T   T   T
  0.0712018141222612  0.5995906665144729  0.4943302326204294   T   T   T
  0.2129273512814550  0.5994794525396147  0.4949103805995006   T   T   T
  0.3555827784776615  0.5986448824599433  0.4965641357296494   T   T   T
  0.5000127342709365  0.5978015769814180  0.4942124945553202   T   T   T
  0.6442860371625700  0.5987614856265894  0.4963366132060066   T   T   T
  0.7872204239929456  0.5995676361815719  0.4948060730934067   T   T   T
  0.9288134964328498  0.5996084743069191  0.4943088649724700   T   T   T
  0.0711207906024175  0.8489520675726920  0.4929564806768056   T   T   T
  0.2134691964166252  0.8487868562051363  0.4928496090704397   T   T   T
  0.3566785619940762  0.8489421670736629  0.4926760542475194   T   T   T
  0.5000009198393053  0.8493197584446761  0.4921465904398075   T   T   T
  0.6433243444122628  0.8490327768594004  0.4925849609952485   T   T   T
  0.7864874970146437  0.8488689630321060  0.4927985598868159   T   T   T
  0.9288532499953405  0.8489718919673422  0.4929329823795099   T   T   T
  0.9999952224858024  0.0093760359222276  0.4988376739912621   T   T   T
  0.1419876832402930  0.0092517683518646  0.4987559828951186   T   T   T
  0.2843278983764052  0.0091243881140511  0.4988390006158075   T   T   T
  0.4282608330381086  0.0107636094622208  0.4990990731032408   T   T   T
  0.5718235192894185  0.0108439143079627  0.4990985362393894   T   T   T
  0.7156559803539447  0.0092722214867144  0.4988436574029290   T   T   T
  0.8580075673459427  0.0092998375550398  0.4987524419166155   T   T   T
  0.0000981919372633  0.2597409525472434  0.4994728510743036   T   T   T
  0.1408093591468242  0.2592636306490815  0.4993695977888412   T   T   T
  0.2823260798422876  0.2590192908805465  0.4996721450084723   T   T   T
  0.4154569211812426  0.2593698817905061  0.4979461636591612   T   T   T
  0.5845334647398014  0.2599573404325502  0.4982396496901637   T   T   T
  0.7180723343973001  0.2591652764871542  0.4997527730471953   T   T   T
  0.8593330674442026  0.2593688898436289  0.4993482034464962   T   T   T
  0.0000864274146021  0.5106101609409236  0.5011393533363432   T   T   T
  0.1420971804552034  0.5106370986942597  0.5014502746453336   T   T   T
  0.2841914288757019  0.5106493559082683  0.5025654086674448   T   T   T
  0.4232017514380708  0.5087983318620495  0.5035802275217658   T   T   T
  0.5769294376975467  0.5087047249052788  0.5032895921641461   T   T   T
  0.7160125289376956  0.5108827958795845  0.5025727508779934   T   T   T
  0.8580086255573713  0.5106966441145475  0.5013534238249711   T   T   T
```

0.0000030180426671	0.7600829249683144	0.5000237328725542	T	T	T
0.1421345895922794	0.7600805076311196	0.5000310466173605	T	T	T
0.2850271509435710	0.7598857268323224	0.5001047853212643	T	T	T
0.4283012044787268	0.7603168975196478	0.4996958851936771	T	T	T
0.5716582244628193	0.7603764574957523	0.4995971079342026	T	T	T
0.7149967841448666	0.7599582879674633	0.5000062068458921	T	T	T
0.8578487559241031	0.7601363805397151	0.4999950419071986	T	T	T
0.1416951385283025	0.0744735016329883	0.4268603936919035	T	T	T
0.2837523048083374	0.0738676136449793	0.4266555471955838	T	T	T
0.4287993217621265	0.0770850051050044	0.4277836266986344	T	T	T
0.5715410339452313	0.0772456615339808	0.4278628559325802	T	T	T
0.7161840772144532	0.0742225471300251	0.4267313459374485	T	T	T
0.8583049156773349	0.0745286343659924	0.4268953858862819	T	T	T
0.0000001594226037	0.0745935087511364	0.4269489228790340	T	T	T
0.1403016601034952	0.3255372698700827	0.4282108152301089	T	T	T
0.2794906415747412	0.3250811594872354	0.4281179566494843	T	T	T
0.4173604554973393	0.3200639824638252	0.4226451097593790	T	T	T
0.5823493380760107	0.3206110648464193	0.4231149696028229	T	T	T
0.7207041477162410	0.3248709147277596	0.4279582170931225	T	T	T
0.8597847423805850	0.3256617058956081	0.4281929556555076	T	T	T
0.0000592277290166	0.3260740121331921	0.4282508667092883	T	T	T
0.1425303558538471	0.5753455973926009	0.4290579852783175	T	T	T
0.2850312694839576	0.5750970176737017	0.4302857558443115	T	T	T
0.4267847511471872	0.5728425749823769	0.4310015893588231	T	T	T
0.5730126472799694	0.5730093048562044	0.4307944722055165	T	T	T
0.7150178641971581	0.5751421967184424	0.4302198341619086	T	T	T
0.8575897740946621	0.5754638333045595	0.4289400419076161	T	T	T
0.0000182542283546	0.5751424768664188	0.4289066199236758	T	T	T
0.1423590700183590	0.8243432178977100	0.4275711626455186	T	T	T
0.2851372939278113	0.8242844400151319	0.4277012207913876	T	T	T
0.4282092344655588	0.8244518081792040	0.4273621731467352	T	T	T
0.5717516213621093	0.8246428275929472	0.4272882066064378	T	T	T
0.7148399791994199	0.8243754182338088	0.4276181464353064	T	T	T
0.8576352774554304	0.8243783305320479	0.4275329096698948	T	T	T
0.9999997206592290	0.8243567032355087	0.4276294549641204	T	T	T
0.0704296522453347	0.1970983830601162	0.4472871873928000	T	T	T
0.2120522988373430	0.1970876718549948	0.4473782939084727	T	T	T
0.3520756805311134	0.1957985391665651	0.4456056776538537	T	T	T
0.5001890045858918	0.2040764381327459	0.4498892067197114	T	T	T
0.6479442329878327	0.1962073577668665	0.4458630697506081	T	T	T
0.7880294577536121	0.1971217642537070	0.4473871521768771	T	T	T
0.9296406290786633	0.1971266380824191	0.4472804836640877	T	T	T
0.0710905410670513	0.4481469974881516	0.4497411806692450	T	T	T
0.2129352872531702	0.4482353753386005	0.4505684174296798	T	T	T
0.3551935353338685	0.4478453772820375	0.4516354452088810	T	T	T
0.4999502856248670	0.4463618096666451	0.4587206077453858	T	T	T
0.6451167867585780	0.4480737134704041	0.4514814610967182	T	T	T
0.7870659577019870	0.4483821459853989	0.4504685869790935	T	T	T
0.9291620750242395	0.4481998990886063	0.4497433875492974	T	T	T
0.0712278340436634	0.6976065553253029	0.4488428032285534	T	T	T
0.2137335062813868	0.6974548957143739	0.4492536782704781	T	T	T
0.3566083705305438	0.6967096116248007	0.4497313343829656	T	T	T
0.4999560588155937	0.6969544696062442	0.4490484661474445	T	T	T
0.6433817952919672	0.6968337433079579	0.4496248717886283	T	T	T

0.7862704647437254	0.6975481574233121	0.4491584926316822	T	T	T
0.9287934025608720	0.6976343791669252	0.4488310625154943	T	T	T
0.0712004290697985	0.9469672242541749	0.4473269968064018	T	T	T
0.2134350670610313	0.9469598191910252	0.4474069672645143	T	T	T
0.3569514733212814	0.9478087589263940	0.4479524680386935	T	T	T
0.5000247928511953	0.9489500016011632	0.4475991711130531	T	T	T
0.6430377897573741	0.9479270382701999	0.4478908897043303	T	T	T
0.7864882580410422	0.9471401296705753	0.4473797023541051	T	T	T
0.9288012013464528	0.9469896949391843	0.4473244713041054	T	T	T
0.0000628146977785	0.1609949683299660	0.5441266153457709	T	T	T
0.1417571390496642	0.1606835571371260	0.5440582650809518	T	T	T
0.2838636934015948	0.1600485435896003	0.5439020011527315	T	T	T
0.4251069055608466	0.1610269325005664	0.5434814462019096	T	T	T
0.5749512698829974	0.1613450404379769	0.5435192230084238	T	T	T
0.7163444375921010	0.1603010390604035	0.5441816093985340	T	T	T
0.8583862995279142	0.1608287866155429	0.5439998604454301	T	T	T
0.0001039838465665	0.4120333339143491	0.5465093841801523	T	T	T
0.1411068798791199	0.4116399173811421	0.5469048437099506	T	T	T
0.2824920788136175	0.4118675053684701	0.5468608015505595	T	T	T
0.7177741196766605	0.4122012693902435	0.5469708514878857	T	T	T
0.8589993231677816	0.4117595586374872	0.5468298673105602	T	T	T
0.0000105720662812	0.6619409591217966	0.5454783784156040	T	T	T
0.1421473734828977	0.6622477002336634	0.5457520712413859	T	T	T
0.2845847697397876	0.6620935476781598	0.5465707031899820	T	T	T
0.4283485112198220	0.6618125492116437	0.5447357866728476	T	T	T
0.5715904613646496	0.6618682707766150	0.5446543227833552	T	T	T
0.7154900540828036	0.6620759144476545	0.5463680407956816	T	T	T
0.8579277276194067	0.6623325012408483	0.5457022604063128	T	T	T
0.9999891886278164	0.9112548138248885	0.5441402389968073	T	T	T
0.1424914457721745	0.9111159022336749	0.5441611924559802	T	T	T
0.2852054981999790	0.9108549140460640	0.5440041254258080	T	T	T
0.4285548630386429	0.9114989615465007	0.5433990522555696	T	T	T
0.5714740022572971	0.9115441803270439	0.5433311148838598	T	T	T
0.7147125243795780	0.9109571918709645	0.5439641930696851	T	T	T
0.8574877666946995	0.9111435243631650	0.5441132649553071	T	T	T
0.0711711211763112	0.0333386845612866	0.5643021914328027	T	T	T
0.2133095031063654	0.0333020527658608	0.5642005197771897	T	T	T
0.3561308538645889	0.0333364377556419	0.5641453645506910	T	T	T
0.5000102013525809	0.0350794167271250	0.5641277886831554	T	T	T
0.6438605547353197	0.0333486272589374	0.5641889176174297	T	T	T
0.7867052089810923	0.0333268898745451	0.5642052425386480	T	T	T
0.9288336623774065	0.0333407076371791	0.5642953726446888	T	T	T
0.0706813280438147	0.2829752378992287	0.5654923167948044	T	T	T
0.2113164013599411	0.2825201210831893	0.5655442940791575	T	T	T
0.3526546997095520	0.2815778949343488	0.5679146458824805	T	T	T
0.4999512344212569	0.2780582189526033	0.5583103967492520	T	T	T
0.6477251998574769	0.2821030997333239	0.5679424502834329	T	T	T
0.7889814743439566	0.2827594072329288	0.5655804860188248	T	T	T
0.9295645826591594	0.2830041007116560	0.5654786441936492	T	T	T
0.0708955525677877	0.5350709319107430	0.5667115168926117	T	T	T
0.2123600471619353	0.5353737843420615	0.5673496192751796	T	T	T
0.3525771383498533	0.5341817565933488	0.5710428023079341	T	T	T
0.4994909035318956	0.5515811334629811	0.5733411769171648	T	T	T
0.6473367215111905	0.5346299997329338	0.5708650281406848	T	T	T

```
  0.7877931768555120  0.5355496499817712  0.5673410263774433   T   T   T
  0.9292273426057615  0.5350299625919931  0.5666636009488367   T   T   T
  0.0711562627051024  0.7844660264308096  0.5654178300247087   T   T   T
  0.2137433522381038  0.7845003244901152  0.5652489418865846   T   T   T
  0.3568995722682904  0.7844365023029983  0.5649693368275710   T   T   T
  0.5000171457412863  0.7851483969748081  0.5644953640178703   T   T   T
  0.6430671558395477  0.7845263604608671  0.5648554390376973   T   T   T
  0.7862262892403808  0.7845280787690814  0.5652078871625672   T   T   T
  0.9288244774478791  0.7845039383675533  0.5653844392844096   T   T   T
  0.4333256041722512  0.3996224609854344  0.6542641847501188   T   T   T
  0.5677482902383582  0.4019422079117219  0.6548509791028098   T   T   T
  0.3706126915466799  0.3831192401271095  0.5874378807603200   T   T   T
  0.5014817786911240  0.3736466016175352  0.5927702738317356   T   T   T
  0.6296386663965416  0.3835162471650638  0.5877796211177486   T   T   T
  0.4389778588482975  0.4719665084640417  0.5907853038599626   T   T   T
  0.5606998389482213  0.4724477751267249  0.5898470850652132   T   T   T
  0.4391563636850694  0.4015100243991630  0.5224837921155594   T   T   T
  0.5622083577670981  0.4012763035196242  0.5217128064766349   T   T   T
== Embedded cluster I-Pd_3k+1 k=3
   1.00000000000000
     24.6586885454000111    0.0000000000000000    0.0000000000000000
      0.0000000000000000   25.2576961516000082    0.0000000000000000
      0.0000000000000000    0.0000000000000000   30.0000000000007034
   W     Te    Pd
    56   112    10
Selective dynamics
Direct
  0.0700215662185416  0.0984320902402171  0.4922675459840353   T   T   T
  0.2121798789174001  0.0981775161290955  0.4921351981130641   T   T   T
  0.3560016918258596  0.0998626754693673  0.4927111801268565   T   T   T
  0.5024393899795920  0.0974579786066325  0.4908327081315701   T   T   T
  0.6445192815946803  0.0982317227339741  0.4916424710556565   T   T   T
  0.7865895432893667  0.0989426076906523  0.4921718861684397   T   T   T
  0.9283817464949610  0.0986951408418314  0.4922102509980454   T   T   T
  0.0662124203553070  0.3484739297767371  0.4947120048337163   T   T   T
  0.2043965425051610  0.3469450815690597  0.4946229979359887   T   T   T
  0.3615185017058354  0.3254693859814238  0.4785870048931903   T   T   T
  0.4971806198557088  0.3435971021203184  0.4897053270150914   T   T   T
  0.6493481226375182  0.3485442531674101  0.4957062272828289   T   T   T
  0.7896869551208783  0.3491629109101509  0.4948185499454699   T   T   T
  0.9282899817085772  0.3495208327299687  0.4945261068602929   T   T   T
  0.0689547255971709  0.6007519957562004  0.4955885639606584   T   T   T
  0.2110740867973021  0.5970174419119555  0.4957221502612371   T   T   T
  0.3524351723844250  0.5996457664201283  0.4999632134987044   T   T   T
  0.5108154801667851  0.5989858897927317  0.4974010795099307   T   T   T
  0.6473192039727915  0.6013316157520562  0.4963309228999675   T   T   T
  0.7880948397723845  0.6010678630492876  0.4956347061044755   T   T   T
  0.9291735625984696  0.6006889167511297  0.4956973423486583   T   T   T
  0.0715902474349545  0.8493694368481981  0.4928278033052500   T   T   T
  0.2142802432854140  0.8489373798301343  0.4926011689580705   T   T   T
  0.3572109548625320  0.8484769380530066  0.4924504128630211   T   T   T
  0.5003440039293650  0.8490299738340388  0.4921728268245746   T   T   T
  0.6440964007167033  0.8497424407662462  0.4921594481695700   T   T   T
  0.7872983921846082  0.8499083674809118  0.4925172346784513   T   T   T
```

0.9293937704604359	0.8497619489452096	0.4927590972993770	T	T	T
0.9996756003423722	0.0095707689480386	0.4986720406954110	T	T	T
0.1418853879163063	0.0094405277385241	0.4986962732326553	T	T	T
0.2849937639195949	0.0100778929411247	0.4988133843093005	T	T	T
0.4280434359701417	0.0100865016976298	0.4984376333352893	T	T	T
0.5725874259427904	0.0093093233633692	0.4982955858876665	T	T	T
0.7156091595742281	0.0097256365007957	0.4984175192975986	T	T	T
0.8578383147441159	0.0098558484922967	0.4984692582069230	T	T	T
0.9977765557079030	0.2589982151285106	0.4995827804436752	T	T	T
0.1377667392538477	0.2588387862092107	0.4998934969110491	T	T	T
0.2726402521346829	0.2604276818514294	0.4986053157436968	T	T	T
0.4405426183572914	0.2537087903840719	0.4937522230426910	T	T	T
0.5749511098182131	0.2606475138295147	0.4992076092571537	T	T	T
0.7148257896712071	0.2591357796654984	0.4998324099004515	T	T	T
0.8577804658957228	0.2593915680933430	0.4994406739283548	T	T	T
0.9977507676129866	0.5108403863891532	0.5021965163411383	T	T	T
0.1328558720289420	0.5108322811325398	0.5026749952018670	T	T	T
0.2978595664648872	0.5045180214917697	0.5044401540117625	T	T	T
0.4126890318940878	0.5072852200801442	0.5053358149441458	T	T	T
0.5845024168305167	0.5123677505801176	0.5057026637820125	T	T	T
0.7186873562240207	0.5121035766492233	0.5030792852746117	T	T	T
0.8585027421616438	0.5111202419114106	0.5019456855806981	T	T	T
0.0001775659830337	0.7609901020628792	0.5003207762403111	T	T	T
0.1423924712036739	0.7602231911069388	0.5001926235208790	T	T	T
0.2848885328727332	0.7598738504748139	0.5005615887122412	T	T	T
0.4292092384397565	0.7595353831637973	0.4999826865836883	T	T	T
0.5731236887326031	0.7615229065258772	0.5002122435358407	T	T	T
0.7161639637331223	0.7614924242960570	0.5000048693131245	T	T	T
0.8584162717307159	0.7612205255107128	0.5002247453525092	T	T	T
0.1411370901304418	0.0744832986559912	0.4266859720111068	T	T	T
0.2847533672024408	0.0761368374325100	0.4271715636566530	T	T	T
0.4277771908951279	0.0764137817329869	0.4270658804999208	T	T	T
0.5738523714495817	0.0730243682222906	0.4258157209643569	T	T	T
0.7159300006187126	0.0748198916925393	0.4263864366889301	T	T	T
0.8573685987952632	0.0752196595640547	0.4266048138171074	T	T	T
0.9991823351796271	0.0746128222090224	0.4265945779926581	T	T	T
0.1362725856845625	0.3251068940305558	0.4282655837488864	T	T	T
0.2738257036089599	0.3210819630140778	0.4243389707675386	T	T	T
0.4386873152486792	0.3148935618736131	0.4169730429083460	T	T	T
0.5773079110539506	0.3276029933100619	0.4293098787292655	T	T	T
0.7179335313546907	0.3260548294042159	0.4291980961946066	T	T	T
0.8584182031183274	0.3256548310336327	0.4282094854936838	T	T	T
0.9976336298364622	0.3250474315552231	0.4281323609063059	T	T	T
0.1403317866669447	0.5735405305659965	0.4300908080118939	T	T	T
0.2850195024406408	0.5714167049139051	0.4331282203311633	T	T	T
0.4305743250856622	0.5748768126371780	0.4376751356390290	T	T	T
0.5770639243004342	0.5727748458478080	0.4307817681159959	T	T	T
0.7171771214971232	0.5758695795334575	0.4302792476822077	T	T	T
0.8583848063059888	0.5763055630872184	0.4300005156824787	T	T	T
0.9991805974351493	0.5754862933025513	0.4298128612332252	T	T	T
0.1428590679395477	0.8241432914638011	0.4276128018474644	T	T	T
0.2856979192570296	0.8235822223723722	0.4275943929918357	T	T	T
0.4287122589623842	0.8233853565547810	0.4273578888480384	T	T	T
0.5721921556206884	0.8248840594384618	0.4272018199330816	T	T	T

0.7158156648183491	0.8250601413658338	0.4272150736188933	T	T	T
0.8583619727296644	0.8248623501791079	0.4273539547465003	T	T	T
0.0004717866775663	0.8247577633253806	0.4275189506931785	T	T	T
0.0687980009552288	0.1968100546254502	0.4470788653993177	T	T	T
0.2086101121508777	0.1966059380664768	0.4463573633342475	T	T	T
0.3542416006119654	0.2011847067622640	0.4487136260493236	T	T	T
0.5074476659495721	0.1939908503857364	0.4429158766968546	T	T	T
0.6453845283541900	0.1974065834744551	0.4470722794855009	T	T	T
0.7864256579878467	0.1977395523338998	0.4476233974608540	T	T	T
0.9278654107188322	0.1971280845646639	0.4470374573952797	T	T	T
0.0682381585801995	0.4480877309092601	0.4494738383044768	T	T	T
0.2133183163081197	0.4505673476568078	0.4589721106060303	T	T	T
0.3566771022922161	0.4422564619447199	0.4494185621872517	T	T	T
0.4994648523766589	0.4518691535510437	0.4680990174636230	T	T	T
0.6475805185926146	0.4486699465749863	0.4527460735117813	T	T	T
0.7879136361414922	0.4487768149895950	0.4505224624821537	T	T	T
0.9286920176102859	0.4482438160882700	0.4498969928441962	T	T	T
0.0710870099246027	0.6977884697913790	0.4492554350279853	T	T	T
0.2134864952295017	0.6960533969538614	0.4505174979299206	T	T	T
0.3562540825321577	0.6956978906522853	0.4509249829009367	T	T	T
0.5028810023581051	0.6966548273007986	0.4508680076708328	T	T	T
0.6448808103203997	0.6986018487123435	0.4494869524148994	T	T	T
0.7873074548478113	0.6985410057993020	0.4494058867504388	T	T	T
0.9292650254315973	0.6981031594314456	0.4495146207915450	T	T	T
0.0713131729889919	0.9473129976912850	0.4472219460133217	T	T	T
0.2140134653788827	0.9476850025521831	0.4475442527324314	T	T	T
0.3567698724223770	0.9477295065836069	0.4475638327556772	T	T	T
0.4999916978203315	0.9477983439459311	0.4473888096032814	T	T	T
0.6440593296438932	0.9477880184344177	0.4470432701919808	T	T	T
0.7867008386832863	0.9477886987969325	0.4469670955763031	T	T	T
0.9289055912686621	0.9475151078768511	0.4470046653580664	T	T	T
0.9987777877742229	0.1605834600755966	0.5441648865341627	T	T	T
0.1405679909303141	0.1598532734738606	0.5443477215817694	T	T	T
0.2823620908271235	0.1609830523062197	0.5434126659460312	T	T	T
0.4312533003952486	0.1585838577175010	0.5428552548787049	T	T	T
0.5729482992807241	0.1599768826232271	0.5427055641717282	T	T	T
0.7149629608433971	0.1602029252173854	0.5440638548498477	T	T	T
0.8574138695806144	0.1608190744542245	0.5440554580952622	T	T	T
0.9978416629247168	0.4117040837325748	0.5473223137843134	T	T	T
0.1357749155461317	0.4125712439746110	0.5477420519460734	T	T	T
0.7192670481622985	0.4129570589103917	0.5480823395259732	T	T	T
0.8581754128565702	0.4117361222137049	0.5471934930153777	T	T	T
0.9997421437172725	0.6639643502994652	0.5469896313754804	T	T	T
0.1420664582726723	0.6624023621734278	0.5462927082993377	T	T	T
0.2820446245290472	0.6624794734003064	0.5487847446416687	T	T	T
0.4313675129807569	0.6606254149570245	0.5454586990612458	T	T	T
0.5758626697716555	0.6646779891525553	0.5475838095441445	T	T	T
0.7170996393551506	0.6647655173730123	0.5469017694982040	T	T	T
0.8584135991535606	0.6639136602155680	0.5468049436304424	T	T	T
0.0005042704510268	0.9117502356938526	0.5440162938278255	T	T	T
0.1430122642668340	0.9113503197256877	0.5438928927893625	T	T	T
0.2859526074952431	0.9111518298477451	0.5435524987238848	T	T	T
0.4286582199970297	0.9111737852779034	0.5432252360054065	T	T	T
0.5719796164788744	0.9111700374462322	0.5432071894993716	T	T	T

```
  0.7152762991178696  0.9117407165261715  0.5434983118479421   T   T   T
  0.8580788687219658  0.9120419008360950  0.5438368366087527   T   T   T
  0.0710529779469390  0.0333637150062419  0.5642170435245434   T   T   T
  0.2134651349080819  0.0333411933376991  0.5641159702782842   T   T   T
  0.3568216895047615  0.0339251016672463  0.5639053649872908   T   T   T
  0.5004790136496083  0.0332923548193207  0.5632552421052558   T   T   T
  0.6437621799638757  0.0334614252066434  0.5636818261475501   T   T   T
  0.7866081820547673  0.0336766691096116  0.5639745331539652   T   T   T
  0.9286632397198096  0.0334801375632143  0.5641526397017068   T   T   T
  0.0677036892001844  0.2824235216711354  0.5660722398361786   T   T   T
  0.2085364130273370  0.2798017754841020  0.5684825512049331   T   T   T
  0.3578206812998461  0.2780797753916757  0.5572142370610551   T   T   T
  0.5012533979968427  0.2797544819653069  0.5645531241841258   T   T   T
  0.6443201878752087  0.2791767143522835  0.5672679711331569   T   T   T
  0.7867451311895385  0.2823652944409979  0.5654072482767343   T   T   T
  0.9277003873493322  0.2828280653781805  0.5657122340645768   T   T   T
  0.0663945996243793  0.5375639401233254  0.5693007715630150   T   T   T
  0.2065128045149226  0.5361401127800186  0.5673975976465545   T   T   T
  0.3541314148615601  0.5459780228752464  0.5795851293167812   T   T   T
  0.5058803158219651  0.5435558186771303  0.5716295771350006   T   T   T
  0.6541052752648602  0.5406368335925046  0.5724021617743261   T   T   T
  0.7899650595982659  0.5366089716686496  0.5680868188009767   T   T   T
  0.9283131970507221  0.5356003760880933  0.5679914458916594   T   T   T
  0.0716517412351556  0.7853518078644643  0.5655318609164425   T   T   T
  0.2138232841883796  0.7854401411434567  0.5653801959891575   T   T   T
  0.3575673011659116  0.7838266588604867  0.5649510711256245   T   T   T
  0.5006953524265816  0.7853281430939858  0.5648814736750626   T   T   T
  0.6446023497536733  0.7865395212152151  0.5650831224906709   T   T   T
  0.7871174254831815  0.7863379743587550  0.5653324843428067   T   T   T
  0.9292602603446224  0.7859727550155453  0.5655583906741610   T   T   T
  0.2871273033047599  0.3999494618320473  0.6525149789180049   T   T   T
  0.4268359473462145  0.4044410066290730  0.6537046094601394   T   T   T
  0.5581630751871285  0.3936838790458884  0.6481065825680916   T   T   T
  0.2245977975089217  0.3811391629418193  0.5856873594667706   T   T   T
  0.3586998665922517  0.3739735723103373  0.5943851814554073   T   T   T
  0.4866471281546476  0.3819299664995036  0.5835151351989945   T   T   T
  0.6332824143963822  0.3808155551238961  0.5896719018877618   T   T   T
  0.2821737934168343  0.4694534799532799  0.5872719116009996   T   T   T
  0.4314624822307049  0.4737357126741466  0.5871005388530492   T   T   T
  0.5787224633254312  0.4749274602744383  0.5975377405825042   T   T   T
  0.2980977586493276  0.3969170555170033  0.5197054685334961   T   T   T
  0.4112311044161557  0.4020589741674193  0.5226319697463944   T   T   T
  0.5671191284825966  0.4099522122853800  0.5284367703893199   T   T   T
== Embedded cluster I-Pd_3k+1 k=4
   1.00000000000000
     24.6586885454000111    0.0000000000000000    0.0000000000000000
      0.0000000000000000   25.2576961516000082    0.0000000000000000
      0.0000000000000000    0.0000000000000000   30.0000000000007034
   W    Te   Pd
    56   112    13
Selective dynamics
Direct
  0.0695150255048044  0.0977012083564880  0.4920091221951464   T   T   T
  0.2120414255120010  0.0973707620905746  0.4915731401768551   T   T   T
```

0.3570295781749290	0.0953168470336304	0.4896603150095659	T	T	T
0.5023050468465010	0.0974194609172329	0.4914181144725016	T	T	T
0.6449292290866699	0.0979080651559418	0.4918757591758011	T	T	T
0.7864014925747603	0.0988715836300473	0.4922437314145170	T	T	T
0.9279418403342587	0.0987743261144265	0.4923128233076315	T	T	T
0.0637900941836516	0.3469015324578058	0.4956139387574700	T	T	T
0.2211987775157668	0.3383442106496737	0.4895125510319259	T	T	T
0.3572298728149867	0.3382478828903802	0.4869336170796282	T	T	T
0.4935212708166437	0.3381693752688445	0.4893396963708999	T	T	T
0.6507251367924486	0.3469354939426342	0.4951985326802393	T	T	T
0.7886168209414053	0.3495692258957690	0.4951003147170167	T	T	T
0.9260126108795940	0.3494594637317071	0.4952248149758948	T	T	T
0.0686855588584781	0.6001407553967396	0.4975841330817314	T	T	T
0.2117412006939355	0.5998631079567026	0.4960644395154620	T	T	T
0.3565964015832320	0.6016439200857489	0.5009964287560396	T	T	T
0.5043969413801125	0.5999246189536481	0.4958687784832579	T	T	T
0.6456945465573070	0.6001892399406947	0.4972001361681108	T	T	T
0.7870494751639796	0.6003254492139698	0.4953797521640176	T	T	T
0.9276738661329816	0.6002673814017836	0.4955161241546868	T	T	T
0.0699175281730940	0.8492367949722459	0.4920292374135108	T	T	T
0.2131902046922740	0.8490714427905710	0.4915855768729756	T	T	T
0.3573281730367362	0.8489583043800020	0.4917281456466355	T	T	T
0.5013276432111721	0.8491876065136794	0.4914651014438060	T	T	T
0.6444204141171456	0.8494373195194591	0.4918242408212519	T	T	T
0.7863647986595292	0.8495997074164998	0.4923077070946957	T	T	T
0.9278549130895593	0.8495171391070692	0.4924334517362581	T	T	T
0.9987003931748245	0.0094222008331160	0.4985021278394926	T	T	T
0.1408605650902445	0.0090891135600809	0.4985734943165153	T	T	T
0.2847679826899953	0.0086315736602593	0.4982994928920820	T	T	T
0.4293909249806859	0.0086958844145475	0.4982964930107343	T	T	T
0.5734928536991940	0.0092839700400055	0.4984259303435202	T	T	T
0.7156825292633801	0.0095776671069939	0.4984904840922139	T	T	T
0.8572412587377403	0.0097900104760375	0.4984922483537821	T	T	T
0.9985302402585939	0.2585061335211242	0.5001273620739508	T	T	T
0.1352628244118126	0.2599213897028330	0.5005521120243492	T	T	T
0.2915935390955712	0.2533866401538350	0.4944311131540252	T	T	T
0.4228241007811966	0.2533304363190138	0.4943737324586097	T	T	T
0.5792001162372424	0.2599872005873471	0.5002908658297958	T	T	T
0.7160042004984724	0.2586223944730718	0.4999026396029508	T	T	T
0.8572616211572981	0.2594312481196248	0.4989644333012505	T	T	T
0.9979117490533387	0.5113663377435282	0.5039799516499224	T	T	T
0.1337250519759926	0.5110501974137496	0.5064393768022026	T	T	T
0.2945528580084203	0.5123254308384962	0.5088441016385605	T	T	T
0.4195885801758686	0.5123347061707825	0.5088641088848564	T	T	T
0.5810036334349692	0.5108487051099880	0.5059577807031062	T	T	T
0.7165478530288945	0.5114045018493758	0.5035527606101381	T	T	T
0.8573911421198624	0.5110874011881906	0.5022775848663731	T	T	T
0.9987051222005824	0.7606593656112366	0.4997961080166921	T	T	T
0.1412353722574992	0.7611904827997824	0.4995096658745165	T	T	T
0.2852228891339078	0.7612767060714948	0.4998311730973863	T	T	T
0.4295226015812800	0.7611099351866836	0.4996602419565756	T	T	T
0.5733575969884985	0.7613717961856513	0.4991740005415576	T	T	T
0.7157636650249547	0.7606457141484976	0.4995371324759190	T	T	T
0.8572007406496494	0.7607661408898219	0.4997693908877421	T	T	T

0.1406698489063475	0.0738600208364455	0.4264399011431619	T	T	T
0.2833856931069593	0.0727701873089285	0.4258737792357314	T	T	T
0.4306933997881028	0.0727013801129451	0.4258643120339425	T	T	T
0.5737739696412466	0.0741060949613095	0.4263189019282765	T	T	T
0.7159476588711765	0.0747871510388283	0.4264171963659193	T	T	T
0.8571223092131629	0.0751217358543480	0.4266325305915146	T	T	T
0.9984827414082793	0.0747942072786793	0.4264942129662570	T	T	T
0.1390640026190200	0.3254406250686560	0.4299634662379402	T	T	T
0.2860433729723140	0.3174502307015458	0.4206507743362700	T	T	T
0.4283801350218268	0.3175629675533410	0.4206042509965813	T	T	T
0.5754380269981917	0.3253613778460939	0.4295406471352254	T	T	T
0.7183385291536895	0.3246099896167529	0.4284539308402100	T	T	T
0.8574161123756006	0.3259608810490212	0.4280946550167722	T	T	T
0.9964384785273710	0.3244863645302742	0.4287170125936752	T	T	T
0.1402339849524228	0.5722388656292107	0.4325926898500728	T	T	T
0.2865385078938974	0.5734682548555524	0.4346917375425297	T	T	T
0.4281792581060527	0.5737597284893684	0.4353472836165317	T	T	T
0.5747912323567462	0.5720139173072005	0.4318827965074148	T	T	T
0.7154667626398389	0.5751381346071669	0.4307565938003622	T	T	T
0.8574734949411978	0.5757042084381555	0.4297469313964401	T	T	T
0.9994208309909786	0.5748639131297186	0.4309682698696114	T	T	T
0.1415187911924675	0.8244860314826168	0.4266629969554064	T	T	T
0.2853334210393658	0.8241996686145974	0.4267902439749045	T	T	T
0.4291185030283836	0.8242569615004325	0.4267349520579327	T	T	T
0.5729113441636864	0.8248244535852062	0.4263641467618961	T	T	T
0.7155432224611459	0.8245200226431419	0.4267952294621774	T	T	T
0.8571254470410490	0.8245906100118028	0.4269883864271690	T	T	T
0.9987481696180234	0.8243827824271276	0.4269756609527525	T	T	T
0.0686323212650142	0.1971112156865155	0.4472183535429722	T	T	T
0.2104447199034619	0.1967630220593777	0.4485523511941464	T	T	T
0.3570479972467525	0.1907462942004108	0.4401502190977487	T	T	T
0.5038166330817818	0.1967894959562635	0.4483939927764730	T	T	T
0.6458371183070790	0.1972270777270079	0.4470104969135822	T	T	T
0.7864892515214208	0.1973425968480904	0.4467643529442135	T	T	T
0.9278542312150994	0.1971923011225576	0.4469574148676463	T	T	T
0.0690055505342924	0.4479437594341053	0.4543542509770837	T	T	T
0.2160589468733314	0.4505692855954817	0.4660081900882340	T	T	T
0.3571302043806580	0.4501098404745378	0.4555085624747657	T	T	T
0.4983408685849132	0.4506045082091235	0.4659749944606706	T	T	T
0.6456832539181151	0.4477296433448040	0.4537138517312613	T	T	T
0.7866320226946800	0.4486446885996888	0.4511538834013613	T	T	T
0.9282683422831762	0.4485114668392813	0.4513475341922874	T	T	T
0.0701121305604724	0.6972137130925653	0.4495448588816455	T	T	T
0.2131643287239734	0.6976097931161973	0.4498825035059542	T	T	T
0.3572285423721038	0.6971686326009477	0.4508200323593318	T	T	T
0.5016217631699745	0.6976051250679380	0.4495732022402934	T	T	T
0.6443795377731240	0.6973091694217635	0.4492011861404481	T	T	T
0.7865273866428402	0.6977339826251656	0.4488215481667960	T	T	T
0.9281785082030406	0.6977580385169420	0.4489356947540665	T	T	T
0.0701200633551913	0.9475375949734526	0.4469593910031438	T	T	T
0.2130610950589840	0.9475069235261487	0.4470172510153760	T	T	T
0.3571145060364835	0.9473803239491835	0.4469709208823390	T	T	T
0.5012694780923510	0.9477630871330500	0.4469831340529343	T	T	T
0.6442622717090285	0.9477781322906199	0.4468248127183170	T	T	T

0.7864002700864452	0.9476770410402782	0.4467737837014055	T	T	T
0.9280749159542301	0.9474423782539074	0.4468184310015767	T	T	T
0.9992616784479216	0.1598045544894833	0.5445113561829225	T	T	T
0.1406068284698401	0.1594263342098378	0.5434723426264934	T	T	T
0.2868647353673758	0.1572283446820852	0.5417707167296709	T	T	T
0.4274552424570815	0.1570503370514845	0.5416379674419733	T	T	T
0.5739475422895096	0.1595538187395259	0.5433646006992645	T	T	T
0.7152300360934302	0.1600099866490575	0.5444089935936191	T	T	T
0.8570785461954895	0.1611716810308289	0.5437844596051455	T	T	T
0.9959781206004583	0.4123167830191423	0.5489481858317813	T	T	T
0.7183057860784287	0.4124758825410320	0.5483707788272196	T	T	T
0.8572172932881942	0.4114941587311032	0.5479484924769412	T	T	T
0.9981972050951934	0.6638044426094536	0.5470281251494851	T	T	T
0.1406388393804054	0.6644272738559536	0.5456885900903999	T	T	T
0.2834874283594075	0.6646905173425699	0.5470334471815036	T	T	T
0.4311995196373927	0.6641901093973323	0.5464776047218444	T	T	T
0.5742844448103088	0.6648134972748313	0.5456447345926896	T	T	T
0.7162232582524458	0.6637427248551161	0.5468268513050999	T	T	T
0.8573265421289999	0.6637698147165672	0.5463034587268542	T	T	T
0.9992615901155179	0.9114003460243417	0.5436435738388905	T	T	T
0.1418630143255230	0.9108232740378119	0.5431653934033984	T	T	T
0.2853094386104356	0.9104105465508507	0.5427569871951347	T	T	T
0.4291245914842888	0.9104797018942332	0.5428890706557309	T	T	T
0.5723918272527307	0.9110141918246708	0.5430345237483022	T	T	T
0.7149803710471194	0.9115940646396931	0.5435865896937686	T	T	T
0.8570626954561502	0.9118768130256433	0.5437705248864714	T	T	T
0.0702147674866678	0.0329281951715612	0.5641598373084967	T	T	T
0.2132495972562025	0.0329581406622957	0.5636193285389605	T	T	T
0.3570755365605135	0.0325805955059816	0.5631986648142778	T	T	T
0.5009943661107252	0.0329931394477741	0.5634608201378825	T	T	T
0.6440118654929902	0.0332871923516791	0.5640462165214789	T	T	T
0.7863651055521417	0.0336037197723251	0.5641601032994550	T	T	T
0.9281511721693306	0.0334462037031935	0.5642088678111402	T	T	T
0.0685850260887971	0.2802979957849299	0.5687170542415200	T	T	T
0.2162136047339081	0.2752054165661843	0.5610455318980928	T	T	T
0.3572903945127748	0.2784032897629251	0.5629587561736632	T	T	T
0.4984016978848999	0.2751142641479754	0.5608976568627247	T	T	T
0.6458765781350287	0.2804961566024951	0.5685013520537743	T	T	T
0.7873526660936170	0.2822981101844703	0.5655661148516763	T	T	T
0.9271060362448533	0.2822553420748539	0.5657000745723149	T	T	T
0.0647247099908943	0.5363973198221204	0.5731170967984315	T	T	T
0.2084790562854193	0.5559357391916792	0.5747376634180053	T	T	T
0.3571988403161535	0.5419596634239177	0.5817573080993460	T	T	T
0.5063471440176153	0.5557754950633556	0.5744121799478520	T	T	T
0.6498344358802940	0.5364457630388281	0.5727335036746227	T	T	T
0.7878549561131256	0.5365638824711172	0.5681859169255573	T	T	T
0.9265908705879986	0.5366172773629648	0.5684937125090163	T	T	T
0.0702318256577620	0.7857637999459525	0.5648399071724717	T	T	T
0.2131673350284032	0.7862729386288417	0.5644642462102866	T	T	T
0.3575193805491135	0.7855197132904915	0.5645294552353732	T	T	T
0.5015316304098070	0.7861442830829386	0.5642695910203086	T	T	T
0.6443077854626287	0.7858880647537381	0.5645140523097187	T	T	T
0.7862527891407618	0.7858711810270731	0.5650586290733887	T	T	T
0.9280694995357003	0.7857740761472010	0.5651959638919346	T	T	T

```
  0.1547163887288533  0.3941016165354805  0.6506173025432452   T   T   T
  0.2863715813810152  0.4050486718301815  0.6520394130030062   T   T   T
  0.4288880413724732  0.4054047866107185  0.6522972090014992   T   T   T
  0.5602374063510350  0.3940871288616374  0.6505510822086497   T   T   T
  0.0834718014613162  0.3822139432528486  0.5897171935068458   T   T   T
  0.2238788435739119  0.3752900647627775  0.5859276103864959   T   T   T
  0.3578875059860510  0.3771446824790347  0.5944215468587338   T   T   T
  0.4909146207952276  0.3751749914808111  0.5859748304517047   T   T   T
  0.6311437406747522  0.3822580652817745  0.5894350583619461   T   T   T
  0.1526715823929166  0.4729803481591821  0.5940694647547695   T   T   T
  0.2699257336550611  0.4784326028494797  0.5925178627213148   T   T   T
  0.4447922848253730  0.4783773191813039  0.5923083034464254   T   T   T
  0.5621936521870623  0.4729026595166280  0.5937794316146588   T   T   T
  0.1480427640098224  0.4052181252877448  0.5249567780916196   T   T   T
  0.2954748115630190  0.4081240794304336  0.5273352261520184   T   T   T
  0.4195507787743648  0.4080854400832604  0.5270390537448866   T   T   T
  0.5665401427021703  0.4050752318472510  0.5247242675273088   T   T   T
== Embedded cluster II-Pd_2k+1 k=1
   1.00000000000000
    24.6586885454000111    0.0000000000000000    0.0000000000000000
     0.0000000000000000   25.2576961516000082    0.0000000000000000
     0.0000000000000000    0.0000000000000000   30.0000000000007034
   W    Te   Pd
    56   112     3
Selective dynamics
Direct
  0.0713495497344659  0.0980925256261591  0.4911127380333501   T   T   T
  0.2133393458454139  0.0979652948891090  0.4912193551677518   T   T   T
  0.3552738067881613  0.0979017613796217  0.4919855656015175   T   T   T
  0.4997859469815750  0.0979558442096839  0.4934327932032553   T   T   T
  0.6430966962759246  0.0979660357594418  0.4934504536356460   T   T   T
  0.7874228603563892  0.0979479813056741  0.4920885434502850   T   T   T
  0.9292418548691123  0.0980203102180403  0.4913127297793918   T   T   T
  0.0714358816414249  0.3487164974492640  0.4923778410680318   T   T   T
  0.2130301559586479  0.3486257206487066  0.4931916769498172   T   T   T
  0.3528183011928637  0.3468239923272730  0.4953258576643990   T   T   T
  0.4895988125058919  0.3454880126063405  0.5020875466347531   T   T   T
  0.6531471732481953  0.3456608316463198  0.5020401426675608   T   T   T
  0.7898881722733796  0.3468467776216635  0.4952496962370044   T   T   T
  0.9298245537587129  0.3485663670169128  0.4930782850221284   T   T   T
  0.0715505071243461  0.5986128299649208  0.4936540496396090   T   T   T
  0.2141101519067704  0.5987814529721071  0.4946616508821193   T   T   T
  0.3558660455352990  0.5991433767521918  0.4965045773842355   T   T   T
  0.4973683296340631  0.6016523965560786  0.4949698708365325   T   T   T
  0.6454751349696904  0.6015748478629953  0.4947702818610978   T   T   T
  0.7872406453064683  0.5990746103128443  0.4962585110635841   T   T   T
  0.9289497599862518  0.5987896830728496  0.4944770111485931   T   T   T
  0.0714132651172654  0.8484349229646798  0.4925410800402233   T   T   T
  0.2139141344377478  0.8485421678618467  0.4925826301619954   T   T   T
  0.3561141588434426  0.8489886558647159  0.4925150923362299   T   T   T
  0.4993505401327163  0.8492624618163693  0.4920452926640933   T   T   T
  0.6437209128528748  0.8492700555331616  0.4921047966110463   T   T   T
  0.7869597117498132  0.8490050001264143  0.4925853902496251   T   T   T
  0.9290876969542745  0.8485433686078421  0.4925421577320847   T   T   T
```

0.0001288903086398	0.0091250867227424	0.4980403610668931	T	T	T
0.1425292427892702	0.0091152774765493	0.4979420726200069	T	T	T
0.2845328440606552	0.0091687529069967	0.4982501508361848	T	T	T
0.4274303566746260	0.0094862728767102	0.4987243542891830	T	T	T
0.5715828721217352	0.0093683957390038	0.4992505953284970	T	T	T
0.7155163726961963	0.0094455811555517	0.4988951152668521	T	T	T
0.8582028810819952	0.0092150183676774	0.4983158553875803	T	T	T
0.0000715291428271	0.2591249851519996	0.4978358108188565	T	T	T
0.1427316588501064	0.2590821243734179	0.4978319656807849	T	T	T
0.2841310923758606	0.2586230305722603	0.4989085523647935	T	T	T
0.4257796213403410	0.2570587192585308	0.5022591022206445	T	T	T
0.5714795861848813	0.2592846664767325	0.5084326796835508	T	T	T
0.7168744055973522	0.2571215555213360	0.5022419125087740	T	T	T
0.8585417787129394	0.2586159257658968	0.4989110210332501	T	T	T
0.9998795675132913	0.5095929378031534	0.5002305056921758	T	T	T
0.1429853863326370	0.5096051581553106	0.5003686648288704	T	T	T
0.2852168890961814	0.5094680417991266	0.5024003134429068	T	T	T
0.4251874197168467	0.5098751538350084	0.5058056594754643	T	T	T
0.5710934618793361	0.5188331232352452	0.5035869984686321	T	T	T
0.7178558271280362	0.5099137764233791	0.5057360538932196	T	T	T
0.8577283923635458	0.5093754104292477	0.5021846531090801	T	T	T
0.0003773830897459	0.7594622948488088	0.4995775725858789	T	T	T
0.1428014096257269	0.7595041251208045	0.4996961704307429	T	T	T
0.2849092507393615	0.7597937025472915	0.4999845694881537	T	T	T
0.4272178280515567	0.7607715658360831	0.5000456521048133	T	T	T
0.5715502304716609	0.7612916311107274	0.4993331471485395	T	T	T
0.7158787732017379	0.7608202172138058	0.4999928700088864	T	T	T
0.8581065215591592	0.7598052977658860	0.4998902555398442	T	T	T
0.1424645626433578	0.0738523562996527	0.4256301906222916	T	T	T
0.2849907431696335	0.0739407803775179	0.4260253610412647	T	T	T
0.4283989803229059	0.0756835332673209	0.4274315096768636	T	T	T
0.5714440195474247	0.0751761018082689	0.4279106259552762	T	T	T
0.7143439580023004	0.0754607219148748	0.4275025426026013	T	T	T
0.8576325216103866	0.0739438298234012	0.4260660389254885	T	T	T
0.0001143118387490	0.0739434453179134	0.4258092267250553	T	T	T
0.1426977718006648	0.3256635817923189	0.4268475798626118	T	T	T
0.2843509442220896	0.3252439109232749	0.4277697679132920	T	T	T
0.4270543024287786	0.3235419427707092	0.4312525859661597	T	T	T
0.5712998531317797	0.3286704685884084	0.4414507279877968	T	T	T
0.7155764963796123	0.3235784045201238	0.4312200352214908	T	T	T
0.8584053250647441	0.3250479236699851	0.4277188233016684	T	T	T
0.0002916639869661	0.3254797450601891	0.4267427845793134	T	T	T
0.1434485388349297	0.5749396524792897	0.4286397408768502	T	T	T
0.2861329446790033	0.5743207468039579	0.4302452165767393	T	T	T
0.4268052961418173	0.5717826158383905	0.4316850049866690	T	T	T
0.5713399421739139	0.5783678178111741	0.4300283790651327	T	T	T
0.7161076135584672	0.5717227355242979	0.4315650042587518	T	T	T
0.8570115467776679	0.5741237775959482	0.4300225799002991	T	T	T
0.9998157993747359	0.5750608891419801	0.4285790585796355	T	T	T
0.1426786074083394	0.8238894877253671	0.4273581403885706	T	T	T
0.2850909867426314	0.8238540182729757	0.4272879660938105	T	T	T
0.4276960447174982	0.8245096458604891	0.4272237039982711	T	T	T
0.5716408474957528	0.8252992791505777	0.4270775643784786	T	T	T
0.7154045502040968	0.8245963061562259	0.4272670442825560	T	T	T

0.8578713211695664	0.8239521839188333	0.4272976958927569	T	T	T
0.0002209500592297	0.8239058434289550	0.4272232753522394	T	T	T
0.0713296356811063	0.1968074068855893	0.4460139719187358	T	T	T
0.2135380086684856	0.1967707045231985	0.4460462082320115	T	T	T
0.3561724366228724	0.1968149667578354	0.4478486347484888	T	T	T
0.5013392503495763	0.1986369713888950	0.4523202103957462	T	T	T
0.6414556084861509	0.1986472495730267	0.4522474683274286	T	T	T
0.7863392501259810	0.1967602502774731	0.4478447852809192	T	T	T
0.9290458552823603	0.1967771767747054	0.4460814611377957	T	T	T
0.0714670366463039	0.4478467061506911	0.4484979011332947	T	T	T
0.2144369690648913	0.4477930676569609	0.4493030696715288	T	T	T
0.3566338172807242	0.4469114097383743	0.4526059292396977	T	T	T
0.4990103818493922	0.4477904571889940	0.4580734859028803	T	T	T
0.6437556751279350	0.4477834341924365	0.4581386552555049	T	T	T
0.7861586326058946	0.4468862712097866	0.4524856233407619	T	T	T
0.9283924972548659	0.4476526223071556	0.4491389216118666	T	T	T
0.0716355150728676	0.6970994822353368	0.4483789528714670	T	T	T
0.2142256812866704	0.6969366819487970	0.4487999149976218	T	T	T
0.3565948530320304	0.6968441750762406	0.4495927009027408	T	T	T
0.4987879792453053	0.6988170169244411	0.4488374901205957	T	T	T
0.6441812172047328	0.6988628909957396	0.4487144048836231	T	T	T
0.7864210130300161	0.6968865318081304	0.4493736954099402	T	T	T
0.9288295774847426	0.6969765193330434	0.4487645371920538	T	T	T
0.0713676807512459	0.9465799501171921	0.4469174015414192	T	T	T
0.2138065852936499	0.9466025707226838	0.4468100505857573	T	T	T
0.3565266197615335	0.9472456194692582	0.4470183473618681	T	T	T
0.4997738766281596	0.9481732340895268	0.4474178888176414	T	T	T
0.6433110026056837	0.9479684175385971	0.4475172478009060	T	T	T
0.7863965975548131	0.9472461220557757	0.4471140383258282	T	T	T
0.9287599381871918	0.9466460605531453	0.4468677195738159	T	T	T
0.0003304255095005	0.1605657153382083	0.5425851932468153	T	T	T
0.1422158428356194	0.1604753531105860	0.5425145620004644	T	T	T
0.2837004763415147	0.1598742117189578	0.5431614347537601	T	T	T
0.4266413262705326	0.1578861824746015	0.5453099735521081	T	T	T
0.5714351580478346	0.1571298357832397	0.5463061714819528	T	T	T
0.7160633092105985	0.1580760340492341	0.5454133920660714	T	T	T
0.8588408189943105	0.1599523896956536	0.5433292982877840	T	T	T
0.0007945210471827	0.4106731337491750	0.5445940750400546	T	T	T
0.1419306702603393	0.4106356407552919	0.5446095246229923	T	T	T
0.2823609981992359	0.4101212110476711	0.5466199292129856	T	T	T
0.4161862877123297	0.4067320236940658	0.5561311423486128	T	T	T
0.7264097168843363	0.4068308558484126	0.5561028987454085	T	T	T
0.8603854885328394	0.4100827336309293	0.5465855372077493	T	T	T
0.0006652214649646	0.6614958268069980	0.5450725442953430	T	T	T
0.1424460991472094	0.6615228078425137	0.5451606214509104	T	T	T
0.2842996683324703	0.6622541317659606	0.5464383883106385	T	T	T
0.4270126315997610	0.6631944654149371	0.5466562072203749	T	T	T
0.5715422651279601	0.6637060937011248	0.5438451774917926	T	T	T
0.7159273255407819	0.6631120513945278	0.5462688212094214	T	T	T
0.8587226896915807	0.6621936987223027	0.5461083833350734	T	T	T
0.0002258667920527	0.9111824357727161	0.5436286124236660	T	T	T
0.1425508703702733	0.9110942134906759	0.5435488120601228	T	T	T
0.2848658694812713	0.9112726123853873	0.5437260489342380	T	T	T
0.4278726452417084	0.9111358503704787	0.5434261695119731	T	T	T

```
  0.5714711985009907  0.9106411621923169  0.5433175681617132   T   T   T
  0.7151480458727221  0.9111289584511353  0.5435687501375605   T   T   T
  0.8580795473223914  0.9112157171392944  0.5437728640087605   T   T   T
  0.0714231030344997  0.0334695622476063  0.5634006475293249   T   T   T
  0.2133288583451526  0.0333788191646664  0.5635839981366214   T   T   T
  0.3558972226002313  0.0329693093754173  0.5638996267049374   T   T   T
  0.4994192984387941  0.0318900282124476  0.5643572849776142   T   T   T
  0.6436752922169179  0.0321404311756341  0.5645174439829356   T   T   T
  0.7870708930038951  0.0330237415335078  0.5640312198044689   T   T   T
  0.9292498251368541  0.0334983178979421  0.5636605114170894   T   T   T
  0.0714165317375757  0.2823689783157762  0.5634008458421128   T   T   T
  0.2124778308401453  0.2820481274165959  0.5642529933131479   T   T   T
  0.3529429627692969  0.2804590073031357  0.5665320582386463   T   T   T
  0.4947072656407682  0.2752405284943170  0.5726516361027930   T   T   T
  0.6481743331637756  0.2754659760210308  0.5727189205902492   T   T   T
  0.7897090458360890  0.2805710869206768  0.5664809357183285   T   T   T
  0.9303037297950282  0.2821749439193108  0.5641938279103100   T   T   T
  0.0713020657627183  0.5334964598612534  0.5655227349566155   T   T   T
  0.2124900943959019  0.5338916389322190  0.5667835465159251   T   T   T
  0.3535809644086281  0.5355719904045895  0.5697182444428318   T   T   T
  0.4982015820559995  0.5432674417552328  0.5705308900125502   T   T   T
  0.6445816612556676  0.5433334906900560  0.5703131137887600   T   T   T
  0.7895875544599482  0.5355945469443905  0.5695708137109375   T   T   T
  0.9301489996348286  0.5339667201756488  0.5665795764332773   T   T   T
  0.0715455581876600  0.7841306177656101  0.5649557968620615   T   T   T
  0.2136770302992956  0.7845439480585622  0.5652033731782522   T   T   T
  0.3561138869246422  0.7852925393830756  0.5652774415176312   T   T   T
  0.4997878946019125  0.7854689085318438  0.5645104136968128   T   T   T
  0.6433616746252685  0.7855541934537077  0.5645320167183074   T   T   T
  0.7869835479014906  0.7851409056205573  0.5653026791614912   T   T   T
  0.9294059114647094  0.7844376181835898  0.5650509377257230   T   T   T
  0.5717142670105197  0.4258833321639595  0.6209681049275799   T   T   T
  0.5713270388795646  0.3488461018457369  0.5639034605507108   T   T   T
  0.6272460897464497  0.4409282103670132  0.5462351844964531   T   T   T
  0.5154410908518819  0.4406772205157871  0.5462808815235793   T   T   T
== Embedded cluster II-Pd_2k+1 k=2
   1.00000000000000
    24.6586885454000111    0.0000000000000000    0.0000000000000000
     0.0000000000000000   25.2576961516000082    0.0000000000000000
     0.0000000000000000    0.0000000000000000   30.0000000000007034
   W    Te   Pd
    56   112    5
Selective dynamics
Direct
  0.0706084917414598  0.0980453232180539  0.4910244418968887   T   T   T
  0.2120959395481151  0.0980884712056469  0.4920431606398998   T   T   T
  0.3556907364412689  0.0977738875878600  0.4936515045115596   T   T   T
  0.5001193013120289  0.0997649897422594  0.4959443209800468   T   T   T
  0.6444839663954622  0.0976722301402878  0.4935836846542213   T   T   T
  0.7880936759590895  0.0980447360702266  0.4920244272953598   T   T   T
  0.9296166873178242  0.0980536628677664  0.4910246130682913   T   T   T
  0.0698484501569352  0.3489840400794215  0.4948402355619407   T   T   T
  0.2089251239564019  0.3478560247194366  0.4964767710658179   T   T   T
  0.3450664753116532  0.3471619971114211  0.5010942476890983   T   T   T
```

0.5000253239856294	0.3365664546614102	0.4999348952977317	T	T	T
0.6550330083923489	0.3471427197965511	0.5011381565292400	T	T	T
0.7911812454512526	0.3477788525292721	0.4965592450016565	T	T	T
0.9303076269450613	0.3489332015288326	0.4948512431759554	T	T	T
0.0703984338091748	0.5991471397965663	0.4962578319137293	T	T	T
0.2115204992705317	0.5989080507901091	0.4971079397844705	T	T	T
0.3607343415171812	0.5997332016172331	0.4949042212680875	T	T	T
0.5000129602146284	0.6045048599797096	0.4936391358138462	T	T	T
0.6394134228587787	0.5996989140522340	0.4948144348484498	T	T	T
0.7886070210597630	0.5989180120101908	0.4971003030251530	T	T	T
0.9297092061580227	0.5991516300529705	0.4962678924141363	T	T	T
0.0708855515983817	0.8484599544643771	0.4925128907677576	T	T	T
0.2129299734942296	0.8487024111524116	0.4926215403643038	T	T	T
0.3562737342897662	0.8496328829268970	0.4919681326821184	T	T	T
0.5000556022047628	0.8504985255213301	0.4915611896934995	T	T	T
0.6439058361920001	0.8495603973530221	0.4918371328348847	T	T	T
0.7873236493620931	0.8486219033188479	0.4924719253461789	T	T	T
0.9293907551492077	0.8484672160607452	0.4924533427850700	T	T	T
0.0001403543049359	0.0088884015265859	0.4973758226420596	T	T	T
0.1414696220669474	0.0090313421916858	0.4979022140978949	T	T	T
0.2840867430621985	0.0094550312959750	0.4988933487792856	T	T	T
0.4282189818183224	0.0102477636554756	0.4998092384073519	T	T	T
0.5719930976814008	0.0101724235661184	0.4997823234726773	T	T	T
0.7162231108437808	0.0093651112535881	0.4988873193067703	T	T	T
0.8587351957430112	0.0090211654315470	0.4978534629930468	T	T	T
0.0002345560680615	0.2590425639913402	0.4986227364711534	T	T	T
0.1405165172781611	0.2589221052044642	0.4994261810495515	T	T	T
0.2815939062144224	0.2578913207557348	0.5026176931587791	T	T	T
0.4187468370853915	0.2590470195853863	0.5079946517233964	T	T	T
0.5813621910710948	0.2589838657451181	0.5080369034630575	T	T	T
0.7185962665344371	0.2578548991960127	0.5027014242255665	T	T	T
0.8596704886872777	0.2589068189508812	0.4994569829497287	T	T	T
0.0000433781845572	0.5097894246384049	0.5027315103367533	T	T	T
0.1408086653958200	0.5097743451460853	0.5038337435225320	T	T	T
0.2824215776916798	0.5100915428371547	0.5070672978891299	T	T	T
0.4358888163686337	0.5152469824437690	0.5021224267466755	T	T	T
0.5640346413789307	0.5152380805735910	0.5021002666858514	T	T	T
0.7177198885867271	0.5100896736251880	0.5070763879332368	T	T	T
0.8592437113030438	0.5097498889456333	0.5038567428397369	T	T	T
0.0001392946570511	0.7595216031015724	0.5000326911095146	T	T	T
0.1420153706069708	0.7597358966797754	0.5001798076262640	T	T	T
0.2846833953373767	0.7601894211261551	0.4999299166440972	T	T	T
0.4280695131453345	0.7622267070821291	0.4989613933351437	T	T	T
0.5719830077964256	0.7621889785570238	0.4989032605844814	T	T	T
0.7155245016615703	0.7601764510087271	0.4997796520172422	T	T	T
0.8582674756581242	0.7597337397915686	0.5001144052411369	T	T	T
0.1422243131173150	0.0740239247685451	0.4257546528866702	T	T	T
0.2851524233155120	0.0753561909436674	0.4274278900504628	T	T	T
0.4293602205108338	0.0778335371982117	0.4292952601545455	T	T	T
0.5708070961522965	0.0777431231709423	0.4292157776595178	T	T	T
0.7150852811165762	0.0752188103417058	0.4273897668122552	T	T	T
0.8580000313062752	0.0740122677706800	0.4257329803264003	T	T	T
0.0001310181858292	0.0741416308609066	0.4252007900456771	T	T	T
0.1407481016566391	0.3264936529177915	0.4289407304742163	T	T	T

0.2812805777503882	0.3241393379615986	0.4314953258525157	T	T	T
0.4225590863931178	0.3253658796772536	0.4372981771081893	T	T	T
0.5775575853283544	0.3253518725478024	0.4373566327740151	T	T	T
0.7187991320632552	0.3240578299753572	0.4315679033733968	T	T	T
0.8592905219887980	0.3265072019300336	0.4289719734359153	T	T	T
0.0000548648353448	0.3265617264827272	0.4281778150581709	T	T	T
0.1419821810401343	0.5738637427416332	0.4311420564037723	T	T	T
0.2852809594698465	0.5717113771042834	0.4337163768244550	T	T	T
0.4296495165203196	0.5757058752625642	0.4282197276737270	T	T	T
0.5704001047009378	0.5756700706909644	0.4281937195234538	T	T	T
0.7149534264697527	0.5715893109683451	0.4336461938991183	T	T	T
0.8581297625506263	0.5738340669097792	0.4311649555947445	T	T	T
0.0000172612412506	0.5746053411201425	0.4304949664100546	T	T	T
0.1422274299071328	0.8235528701915554	0.4273639884708225	T	T	T
0.2846187107789684	0.8240450389101520	0.4273939718944769	T	T	T
0.4281207618030398	0.8263123770113091	0.4265146411995220	T	T	T
0.5719478989686891	0.8262898441679045	0.4264641332207803	T	T	T
0.7155615589096528	0.8240415398518703	0.4272282061411472	T	T	T
0.8581861001480993	0.8234723119712520	0.4272330275987494	T	T	T
0.0001879157655148	0.8233492836626070	0.4270762011116308	T	T	T
0.0708797327444374	0.1969486726945913	0.4459785464008028	T	T	T
0.2127650936073225	0.1971627257742652	0.4479888096687782	T	T	T
0.3553493716186366	0.1982400348262601	0.4508075751375741	T	T	T
0.5000317295390488	0.2031039202204274	0.4583386507591861	T	T	T
0.6448473683047459	0.1982110368306306	0.4509091587517620	T	T	T
0.7874277045015293	0.1971938998166681	0.4479818242658378	T	T	T
0.9294664767484029	0.1969668856304318	0.4461291067643750	T	T	T
0.0709692209958753	0.4480111666898655	0.4509310737020752	T	T	T
0.2131423469558799	0.4480164716536845	0.4540211089716163	T	T	T
0.3558974124699945	0.4478248544798862	0.4575998966688321	T	T	T
0.5000077212061123	0.4446864762979482	0.4556981813575034	T	T	T
0.6441845670504236	0.4478619942192779	0.4576330277398509	T	T	T
0.7869175530531087	0.4479674111898855	0.4540992082625931	T	T	T
0.9290982675067629	0.4479752687439524	0.4509674663815592	T	T	T
0.0713261254225884	0.6964500656587034	0.4494576544748043	T	T	T
0.2135599739789135	0.6962834229119378	0.4498266776138939	T	T	T
0.3569601090905309	0.6978205731700143	0.4492303900020703	T	T	T
0.5000057993796099	0.7012740229072418	0.4472686996685044	T	T	T
0.6431619754159050	0.6978329405973795	0.4491614412256379	T	T	T
0.7866478535046850	0.6962759774890375	0.4497711407671635	T	T	T
0.9289216743181947	0.6964360605264096	0.4494209064120087	T	T	T
0.0712131781819187	0.9461914429698040	0.4464344995040495	T	T	T
0.2135840971752605	0.9469031450307328	0.4470101416336533	T	T	T
0.3568028624836757	0.9485751688837254	0.4476890106911818	T	T	T
0.5000554482255306	0.9496946235287983	0.4477177069763544	T	T	T
0.6433856252909975	0.9485382481367122	0.4476699863126800	T	T	T
0.7866343890357278	0.9468107914260567	0.4469603964808208	T	T	T
0.9289969561527800	0.9462141846455905	0.4463702161853794	T	T	T
0.0001315960123500	0.1603962343890078	0.5428538268504297	T	T	T
0.1406880436043937	0.1599238754666145	0.5433303207348318	T	T	T
0.2826653066319506	0.1581571416547200	0.5455138020712802	T	T	T
0.4249189604866492	0.1577462708678607	0.5477806427772911	T	T	T
0.5753668305179402	0.1576047783721224	0.5478379730708857	T	T	T
0.7174425900383737	0.1580945002808997	0.5454369700325506	T	T	T

0.8594627251433912	0.1599116373010391	0.5433279924129397	T	T	T
0.0000465380553088	0.4110902389585705	0.5474252345202018	T	T	T
0.1395205213462705	0.4106608011268472	0.5484798158212840	T	T	T
0.2760651568539763	0.4085639199614597	0.5556091472833626	T	T	T
0.7239884750415505	0.4085714892125378	0.5556144464754272	T	T	T
0.8605746066526101	0.4106150883192352	0.5485028321736356	T	T	T
0.0000696608270412	0.6625216570544848	0.5471151619395084	T	T	T
0.1416877622153815	0.6624716045992495	0.5472277569303595	T	T	T
0.2862474564953666	0.6615644143301774	0.5452788544374826	T	T	T
0.4291323095646675	0.6656901712228422	0.5454530791354550	T	T	T
0.5709571081003189	0.6656455585741460	0.5453991749218287	T	T	T
0.7138496285882325	0.6616057581729956	0.5451682667986069	T	T	T
0.8584390946595351	0.6625034845795710	0.5472127410570079	T	T	T
0.0000581095903827	0.9114085257551283	0.5435649098339446	T	T	T
0.1419970073225799	0.9111699765068263	0.5436385029013959	T	T	T
0.2847258405118079	0.9110137119221274	0.5435842926342974	T	T	T
0.4282891449077431	0.9112553967266667	0.5434371660958772	T	T	T
0.5720686324729973	0.9111541324076273	0.5434010065987928	T	T	T
0.7155241616137329	0.9107761730745255	0.5434835188738364	T	T	T
0.8581546822391383	0.9111343816913279	0.5435243460769975	T	T	T
0.0706323898311524	0.0332103331436005	0.5632283567879135	T	T	T
0.2123985147410427	0.0328201721585703	0.5638598946229973	T	T	T
0.3555883180305260	0.0319023754011036	0.5648399872666147	T	T	T
0.5001540114335599	0.0318698062054118	0.5655801190649480	T	T	T
0.6447054246240107	0.0318632269480952	0.5647955621190446	T	T	T
0.7879023945840574	0.0328047755563518	0.5638630995082540	T	T	T
0.9295631796718928	0.0332166888065309	0.5631954500448312	T	T	T
0.0698800635800851	0.2813848274811355	0.5653733752372891	T	T	T
0.2089904836455500	0.2808667777400030	0.5670930244108848	T	T	T
0.3468901024554640	0.2776500630383648	0.5743345910319237	T	T	T
0.5000521181537008	0.2679425570541381	0.5728676439124815	T	T	T
0.6531827112481871	0.2776634443106272	0.5743400995970227	T	T	T
0.7912684192677070	0.2807734589157433	0.5672058240328097	T	T	T
0.9305356692393383	0.2811865690819834	0.5653010147233917	T	T	T
0.0698830915106793	0.5349894286865428	0.5690046418199952	T	T	T
0.2106483050751365	0.5362465669901303	0.5706349163593852	T	T	T
0.3589625805330809	0.5406200333126111	0.5695681470483791	T	T	T
0.4999967316785941	0.5458860008267020	0.5700598619334943	T	T	T
0.6410027158066330	0.5406522032943544	0.5695053646797222	T	T	T
0.7893688730411151	0.5362559637058054	0.5706621889710199	T	T	T
0.9301647874342100	0.5349767712999787	0.5689907637107118	T	T	T
0.0710876109461717	0.7847805678638380	0.5653545670140023	T	T	T
0.2137258309883465	0.7844831902631449	0.5652460739988191	T	T	T
0.3566208701877711	0.7859296106423260	0.5644483418618867	T	T	T
0.5000419175075224	0.7865545178669830	0.5638594556567780	T	T	T
0.6435118386117687	0.7859029293876321	0.5643024347035070	T	T	T
0.7865179080150501	0.7844698262496440	0.5651308310109968	T	T	T
0.9291149241236493	0.7848261113113612	0.5653336993854964	T	T	T
0.4407791161945592	0.4236865549456797	0.6218566307037922	T	T	T
0.5592842150092703	0.4236902595092775	0.6218566120498146	T	T	T
0.3789734000314059	0.4406087514310373	0.5452159926782089	T	T	T
0.4999877122286978	0.4309230671103462	0.5448702885991344	T	T	T
0.6210790596366623	0.4406184954183570	0.5452124226879549	T	T	T
0.4269369173338753	0.3476614720585453	0.5647423102505010	T	T	T

```
  0.5730923143113552  0.3476871397162589  0.5647163923242078   T   T   T
== Embedded cluster II-Pd_2k+1 k=3
   1.00000000000000
    24.6586885454000111    0.0000000000000000    0.0000000000000000
     0.0000000000000000   25.2576961516000082    0.0000000000000000
     0.0000000000000000    0.0000000000000000   30.0000000000007034
   W    Te   Pd
    56   112     7
Selective dynamics
Direct
  0.0692411351601102  0.0978155217182703  0.4923871510438400   T   T   T
  0.2115872295269315  0.0984596093939470  0.4936583581135195   T   T   T
  0.3562995532804079  0.0975225738572613  0.4951591093026028   T   T   T
  0.5016679094858800  0.0972580836989804  0.4950825586426815   T   T   T
  0.6450605953885914  0.0978099668762359  0.4934269597901413   T   T   T
  0.7878344095827465  0.0981557657007513  0.4924915845666107   T   T   T
  0.9287153771889856  0.0981399931726278  0.4922096622544906   T   T   T
  0.0674496157185228  0.3481096284571042  0.4981340343760945   T   T   T
  0.2081345771397042  0.3450073797360609  0.5009312960033089   T   T   T
  0.3712297203753759  0.3521430637536682  0.4956045634911480   T   T   T
  0.4880531391637099  0.3457893687498485  0.4980941958384923   T   T   T
  0.6517325429956066  0.3466217891834576  0.5012192085701723   T   T   T
  0.7899894459475172  0.3475254510103676  0.4976982999336262   T   T   T
  0.9290549676940661  0.3487600524717309  0.4972573640294933   T   T   T
  0.0681020657381893  0.5990626282562075  0.4983122959269508   T   T   T
  0.2137244950255665  0.5976533972755497  0.4949970443066426   T   T   T
  0.3607985322587008  0.6050224974096619  0.4952723162584975   T   T   T
  0.4976923323812246  0.6054005196882211  0.4948098589682824   T   T   T
  0.6410931820110034  0.5992616403067188  0.4948556746648932   T   T   T
  0.7876924581573997  0.5986168790615154  0.4986262564905997   T   T   T
  0.9284096105528159  0.5988137711843609  0.4986601630932384   T   T   T
  0.0699239166064990  0.8483755581316685  0.4925781748156615   T   T   T
  0.2125462780497466  0.8492067165032054  0.4919287515946554   T   T   T
  0.3559559508259654  0.8506160535673369  0.4915929208927938   T   T   T
  0.5006957145469598  0.8508766639068706  0.4912450160902219   T   T   T
  0.6444775886793277  0.8493887212990039  0.4914412297885735   T   T   T
  0.7868558484163342  0.8483789977545840  0.4921538704538150   T   T   T
  0.9282237193735274  0.8483737432682930  0.4924672989348182   T   T   T
  0.9988073146150859  0.0086823762804767  0.4979763035303646   T   T   T
  0.1406450294533818  0.0092567245392677  0.4985709975495392   T   T   T
  0.2843690487023030  0.0099002611055110  0.4998367979652846   T   T   T
  0.4283334164673682  0.0096915134254068  0.5003178512927231   T   T   T
  0.5728932874587590  0.0094199610559764  0.4993713739931865   T   T   T
  0.7166876254295919  0.0092202504792636  0.4983732076561494   T   T   T
  0.8580473818278421  0.0089632252987405  0.4980524893498195   T   T   T
  0.9983066594785803  0.2588626121021898  0.5017569125686359   T   T   T
  0.1349728242351179  0.2581390025881629  0.5030064377282970   T   T   T
  0.2910562622594915  0.2583637794249074  0.5070050315824017   T   T   T
  0.4276115939505511  0.2580024360303796  0.5059480252490938   T   T   T
  0.5726156640839659  0.2585879686825707  0.5075819163421030   T   T   T
  0.7172662233162403  0.2575914768370532  0.5030319501680827   T   T   T
  0.8586929840008911  0.2586790339730434  0.5011474676975322   T   T   T
  0.9977456080808647  0.5094366447386627  0.5062723610382986   T   T   T
  0.1334997200894050  0.5098707707232543  0.5071477079219555   T   T   T
```

0.3008638945688832	0.5141174469922090	0.5031937744912605	T	T	T
0.4291589066033037	0.5166436927920284	0.5032235832021905	T	T	T
0.5597273317687917	0.5153708939450451	0.5029491073713293	T	T	T
0.7186331304048329	0.5091816585430931	0.5079727432171069	T	T	T
0.8577819410263876	0.5092348172386064	0.5063365005499976	T	T	T
0.9990208006755754	0.7594107735289252	0.5005280585983948	T	T	T
0.1413365781946529	0.7596666098607117	0.5000106784662739	T	T	T
0.2842617453742213	0.7615858858750596	0.4990899085106391	T	T	T
0.4283168389218648	0.7639419984493298	0.4988752906452422	T	T	T
0.5724146045322226	0.7623293978643690	0.4986449749619340	T	T	T
0.7154277302522450	0.7598780038012374	0.4996087260605249	T	T	T
0.8575185429691752	0.7593018612591084	0.5003587561026682	T	T	T
0.1416814442855039	0.0758410767068655	0.4272365588527965	T	T	T
0.2847873501899615	0.0765209937045699	0.4289455375168625	T	T	T
0.4288688019967842	0.0769071457843824	0.4297242102005082	T	T	T
0.5723152306607493	0.0758120834563881	0.4284326348563154	T	T	T
0.7156219612784629	0.0758441064695355	0.4271707968867710	T	T	T
0.8579442379221491	0.0746707795301964	0.4263506654035014	T	T	T
0.9992165613113659	0.0744155356671914	0.4262979889042330	T	T	T
0.1408773491637113	0.3255618133574951	0.4330480647101611	T	T	T
0.2866185096869134	0.3287312410327317	0.4390949126138907	T	T	T
0.4289455407359957	0.3133992044720424	0.4276229594892230	T	T	T
0.5718465252452816	0.3275045672877668	0.4395775749428110	T	T	T
0.7169988520078641	0.3246011845343234	0.4324943341061420	T	T	T
0.8589359738232991	0.3262135507653585	0.4306181387254450	T	T	T
0.9993796648426128	0.3257126960293336	0.4308966053262108	T	T	T
0.1398751873747231	0.5711513825850830	0.4331127879363927	T	T	T
0.2876634183667839	0.5761026501129616	0.4310955749298938	T	T	T
0.4287970258364428	0.5782396044673482	0.4285261533819682	T	T	T
0.5692370964683150	0.5761557488205095	0.4299067187030552	T	T	T
0.7157220962876583	0.5702962042749531	0.4340430501103230	T	T	T
0.8576628258415168	0.5728493025331859	0.4332926045052843	T	T	T
0.9984515023505206	0.5727582327792492	0.4329592046583227	T	T	T
0.1411705829172152	0.8233635286407304	0.4272454893070370	T	T	T
0.2842528097785840	0.8253915403236928	0.4265605328950832	T	T	T
0.4281663801633164	0.8276511239730381	0.4262615260730739	T	T	T
0.5723110550579159	0.8262613722892990	0.4260927273181430	T	T	T
0.7155920611271068	0.8233847306619116	0.4266661558213163	T	T	T
0.8576655387095093	0.8226385522296208	0.4270869000951994	T	T	T
0.9993063280802136	0.8228876358502467	0.4273120263284113	T	T	T
0.0691589511905747	0.1972463496390013	0.4478296452916258	T	T	T
0.2140588717385395	0.2007839453091641	0.4542153815844156	T	T	T
0.3575738385063567	0.1974802947632399	0.4525146613756142	T	T	T
0.5008744634870875	0.1976643622412355	0.4548930266397100	T	T	T
0.6430512841302697	0.1986809364711219	0.4524127243321258	T	T	T
0.7875361932734808	0.1974731849871221	0.4489140534620976	T	T	T
0.9295020114928947	0.1971511458300157	0.4478349302925229	T	T	T
0.0692678612278589	0.4474850047313836	0.4538149098675642	T	T	T
0.2157947113513666	0.4511854890866421	0.4642135120452844	T	T	T
0.3620325269486532	0.4496216904092136	0.4516957742158689	T	T	T
0.4963807006831477	0.4477095328189136	0.4552155538141374	T	T	T
0.6420671074653062	0.4489166466734825	0.4602945095165704	T	T	T
0.7867917396718344	0.4475186142117179	0.4550220991824810	T	T	T
0.9284553949401839	0.4478048927221807	0.4535846037281269	T	T	T

0.0700600559333619	0.6955423703980205	0.4503704593823214	T	T	T
0.2128776726467879	0.6964817868427561	0.4498767147112797	T	T	T
0.3570637942334491	0.7012211312220111	0.4479872331228589	T	T	T
0.4997277745978140	0.7018235304371097	0.4475294806311985	T	T	T
0.6435430066308087	0.6971720250637905	0.4491579810558967	T	T	T
0.7864743566666414	0.6951852552638806	0.4501966170161129	T	T	T
0.9283477300038395	0.6951734408847859	0.4505351734882494	T	T	T
0.0705100448933322	0.9466029781221166	0.4467363365224286	T	T	T
0.2133669879410829	0.9481647354266776	0.4473715742691620	T	T	T
0.3564131974709643	0.9494919727084519	0.4479436028205441	T	T	T
0.5001763808071539	0.9497247560128441	0.4475813021949134	T	T	T
0.6441032466787580	0.9482312554520039	0.4470173846345605	T	T	T
0.7868409489478164	0.9466818922098175	0.4465136388111191	T	T	T
0.9283602675703271	0.9462298270410854	0.4463097467425652	T	T	T
0.9987600800502663	0.1594150830268963	0.5450967797101249	T	T	T
0.1388623636811966	0.1589482258508919	0.5457823047968374	T	T	T
0.2834743429091184	0.1565152743839259	0.5465562730760007	T	T	T
0.4294439696970016	0.1551797436402105	0.5493568493954762	T	T	T
0.5744960080352571	0.1566909055428296	0.5468945540900415	T	T	T
0.7168731640263133	0.1585824010085366	0.5459216713899820	T	T	T
0.8582990425566284	0.1595628240605585	0.5447951451620746	T	T	T
0.9987582636357480	0.4103301062431262	0.5509778583202378	T	T	T
0.1385407827818840	0.4096684236079172	0.5544569878218595	T	T	T
0.7211502876086577	0.4081613911400723	0.5557990315430847	T	T	T
0.8588073690582714	0.4099768554446785	0.5505620263382924	T	T	T
0.9986597413920590	0.6633545645388325	0.5491038769348178	T	T	T
0.1429418586603930	0.6615376642490621	0.5464431604251454	T	T	T
0.2854383242945435	0.6638269617713578	0.5447017238198730	T	T	T
0.4289737857346418	0.6689326086505362	0.5466701356651986	T	T	T
0.5710820587923884	0.6653588489981092	0.5447852089261487	T	T	T
0.7132825482744889	0.6617864474707650	0.5458591377041656	T	T	T
0.8571920836801870	0.6628648486671871	0.5487744330947266	T	T	T
0.9992196157199251	0.9108831627229579	0.5436503218454086	T	T	T
0.1415070654186366	0.9108817910791935	0.5434423764516511	T	T	T
0.2841374250998156	0.9109670096298823	0.5435345014941786	T	T	T
0.4283905617822715	0.9109157267561228	0.5433922137901016	T	T	T
0.5726800936102854	0.9109023550366852	0.5431653338321801	T	T	T
0.7156373658209485	0.9108502313338868	0.5430999101361069	T	T	T
0.8575270574290212	0.9109935047171723	0.5435015707650911	T	T	T
0.0694786248721510	0.0320771270468488	0.5639648819077705	T	T	T
0.2116899116463502	0.0323519360184621	0.5645783058158491	T	T	T
0.3560650711054455	0.0309701525645712	0.5658887411966307	T	T	T
0.5013080849740569	0.0305863882515874	0.5656018222812763	T	T	T
0.6453783309954469	0.0316785507025722	0.5643179331768355	T	T	T
0.7873857948861810	0.0326252667143975	0.5640140657802648	T	T	T
0.9285121881906171	0.0325002382305603	0.5638997934019538	T	T	T
0.0670969608819458	0.2812969243988059	0.5695941558283673	T	T	T
0.2097385755201258	0.2764765378171374	0.5687968741001974	T	T	T
0.3557345815123218	0.2583520383121147	0.5785040718194598	T	T	T
0.5018137444550110	0.2688550353783075	0.5764484807509652	T	T	T
0.6482283549749872	0.2783543847052103	0.5718980623644041	T	T	T
0.7889675157547918	0.2803437823955062	0.5682822475974136	T	T	T
0.9282064143401250	0.2811522546678011	0.5678779000269745	T	T	T
0.0675962326066243	0.5379806802778757	0.5730471002727675	T	T	T

```
  0.2167495363877583  0.5361965658600807  0.5673119115661457   T   T   T
  0.3617119046904798  0.5467223696327589  0.5715402443738500   T   T   T
  0.4975417144346688  0.5466876551999188  0.5708164181084563   T   T   T
  0.6393322216057966  0.5415848989831181  0.5693020689243270   T   T   T
  0.7876503231775334  0.5366900159651382  0.5728247972644429   T   T   T
  0.9280363872775420  0.5354646324358687  0.5719092558314710   T   T   T
  0.0706227693903996  0.7844523467696556  0.5653867488095787   T   T   T
  0.2130767532434741  0.7856246774495304  0.5645639592731685   T   T   T
  0.3560909556040425  0.7867081500814589  0.5640310839293717   T   T   T
  0.5007729862412120  0.7874029007509471  0.5637550439210505   T   T   T
  0.6436964059249756  0.7860592215747447  0.5641408913993577   T   T   T
  0.7859835917313662  0.7846986056184768  0.5651081093579091   T   T   T
  0.9281961749820268  0.7852579620478991  0.5655979639121198   T   T   T
  0.2999190653495010  0.4186453952201714  0.6220502848917471   T   T   T
  0.4368773866253152  0.4238230880098495  0.6233432201970336   T   T   T
  0.5616909205455912  0.4224529358826284  0.6222084346296169   T   T   T
  0.2479847857231130  0.4359789698304086  0.5475602653362701   T   T   T
  0.3713780091862357  0.4367515791827392  0.5479967835107941   T   T   T
  0.4891124044767484  0.4355058292567237  0.5457253952339971   T   T   T
  0.6169814596293827  0.4416019968417137  0.5466782099194810   T   T   T
  0.3048774360239932  0.3470512417620845  0.5609754607793174   T   T   T
  0.4214137503931742  0.3412951873625977  0.5726672683076329   T   T   T
  0.5632574065810871  0.3509102973684065  0.5599640120699579   T   T   T
== Embedded cluster II-Pd_2k+1 k=4
   1.00000000000000
    24.6586885454000111    0.0000000000000000    0.0000000000000000
     0.0000000000000000   25.2576961516000082    0.0000000000000000
     0.0000000000000000    0.0000000000000000   30.0000000000007034
   W    Te   Pd
    56   112     9
Selective dynamics
Direct
  0.0716580088063257  0.0983649774211598  0.4932493638990857   T   T   T
  0.2133876662473553  0.0977766859371925  0.4944364951278388   T   T   T
  0.3572567536773599  0.0990059447826189  0.4955900908746971   T   T   T
  0.5012366835856401  0.0978700786340231  0.4942531125647485   T   T   T
  0.6430270426146845  0.0984216799747078  0.4931779064933033   T   T   T
  0.7864657142912080  0.0975891569303520  0.4915085191697524   T   T   T
  0.9279760406152000  0.0975990767206514  0.4914943087253409   T   T   T
  0.0629877327994943  0.3474193400675252  0.5015499998039811   T   T   T
  0.2145800794171589  0.3453221214244972  0.5022536461822923   T   T   T
  0.3574231125850154  0.3399607587741595  0.4981407942189278   T   T   T
  0.4995280260516964  0.3454865174505574  0.5018354631349182   T   T   T
  0.6515323279495649  0.3475338990980236  0.5010473137948706   T   T   T
  0.7882852258299499  0.3474310587517176  0.4973214809146228   T   T   T
  0.9261548058897935  0.3473446515987247  0.4975634226516492   T   T   T
  0.0732556442803105  0.5982350150742106  0.4958468085361122   T   T   T
  0.2187833888306033  0.6035774041580652  0.4968127777674778   T   T   T
  0.3572557670630085  0.6016059751001814  0.4981196695745703   T   T   T
  0.4958852464838592  0.6036880712841257  0.4966943944781455   T   T   T
  0.6412192529855126  0.5983969731747936  0.4955190801804866   T   T   T
  0.7873555456308923  0.5990158801094173  0.4997084969556296   T   T   T
  0.9271838993733398  0.5989786427777676  0.4998400280701349   T   T   T
  0.0716172226660213  0.8489039307581216  0.4914002995452040   T   T   T
```

0.2144321566005616	0.8500810719207146	0.4915900218102585	T	T	T
0.3574726794159455	0.8509151223831943	0.4917481747423550	T	T	T
0.5003311276147397	0.8500840051853203	0.4916405855225957	T	T	T
0.6432995578411056	0.8489578911376028	0.4914666570652260	T	T	T
0.7861862504122654	0.8482694580018392	0.4917744050075931	T	T	T
0.9286644238872771	0.8481755250117261	0.4917302082039823	T	T	T
0.9997279413259021	0.0091372934053224	0.4981361286748560	T	T	T
0.1426685936303969	0.0093418492585457	0.4989233530938233	T	T	T
0.2857026221620295	0.0100526060255679	0.4997252791271140	T	T	T
0.4291122252152565	0.0101549675505692	0.4997464396609114	T	T	T
0.5721451186343762	0.0094094887104779	0.4990031459798970	T	T	T
0.7150626745630478	0.0091441404810458	0.4981319145345868	T	T	T
0.8573850486751525	0.0086965703598763	0.4977202207507897	T	T	T
0.9975767994718866	0.2578807058683294	0.5026241938226474	T	T	T
0.1413951459039722	0.2602104167086361	0.5087229968736041	T	T	T
0.2787585737186506	0.2589372296083698	0.5071373051187613	T	T	T
0.4356479180379880	0.2590359110034022	0.5068614272912795	T	T	T
0.5729330385874541	0.2603685895202369	0.5082210273120927	T	T	T
0.7169295077798185	0.2579430881974677	0.5022529688119742	T	T	T
0.8571744150809105	0.2583296829213403	0.5006681167613835	T	T	T
0.9940670485227547	0.5092219838783804	0.5089785459446932	T	T	T
0.1566868496946319	0.5149224461719824	0.5056135585822039	T	T	T
0.2881529632044622	0.5126529166876899	0.5064088325493585	T	T	T
0.4264033185089403	0.5127652370814143	0.5062991189180585	T	T	T
0.5577163437868816	0.5149053766911452	0.5052848428745579	T	T	T
0.7206735928504078	0.5092653552681701	0.5086487254107045	T	T	T
0.8573264470935804	0.5092408425031933	0.5079073149991321	T	T	T
0.0002376496265000	0.7597779335143610	0.4997670997669815	T	T	T
0.1430518958056595	0.7610785160065567	0.4989663821678965	T	T	T
0.2864968699722413	0.7626521026092159	0.4995399222720193	T	T	T
0.4282330444584961	0.7626187498664738	0.4995225739178155	T	T	T
0.5717683099034109	0.7611670312987311	0.4990203128683330	T	T	T
0.7146255411977797	0.7598647272081822	0.4997871129852362	T	T	T
0.8573305049679125	0.7594335724686594	0.5003572962167556	T	T	T
0.1430485163162074	0.0757836381579190	0.4278208906917237	T	T	T
0.2861914714957371	0.0778226668653314	0.4292418378748821	T	T	T
0.4283449619450916	0.0778602553222483	0.4292012566029491	T	T	T
0.5716656899224284	0.0755853518747965	0.4277844074580656	T	T	T
0.7137481339057334	0.0751679246122074	0.4268565997798970	T	T	T
0.8571371966503541	0.0738267704015364	0.4256963945616731	T	T	T
0.0008257852399530	0.0751279051598369	0.4268629707955390	T	T	T
0.1396557166499240	0.3266927648395357	0.4383893200440296	T	T	T
0.2827061130415638	0.3209121222224435	0.4336878155257677	T	T	T
0.4315353649322247	0.3208526922080259	0.4332599725993927	T	T	T
0.5746806853542982	0.3271716359424792	0.4380337171701460	T	T	T
0.7161033787385714	0.3256070724858271	0.4318631167080387	T	T	T
0.8573253005308534	0.3263121813702036	0.4303915358098415	T	T	T
0.9985159363750031	0.3255325895718087	0.4322933656049104	T	T	T
0.1465151703548058	0.5747355041060357	0.4319972242075452	T	T	T
0.2880262222287056	0.5740265234631136	0.4321233110400043	T	T	T
0.4263921912945087	0.5742926036856972	0.4320999720646409	T	T	T
0.5679352647412612	0.5748175483749910	0.4317370210963358	T	T	T
0.7160611404715290	0.5703260238752238	0.4346083561616997	T	T	T
0.8572718800850968	0.5723743759220872	0.4343540519026883	T	T	T

0.9984859129631543	0.5702097574564559	0.4348306189204695	T	T	T
0.1432509229150379	0.8248525352855429	0.4262957859429952	T	T	T
0.2861517483301961	0.8259540611398171	0.4264862292783118	T	T	T
0.4287378753875690	0.8258735521015778	0.4264674582970202	T	T	T
0.5716851768660500	0.8249193238460053	0.4263743879661571	T	T	T
0.7148679414710349	0.8231553324449755	0.4266712499397903	T	T	T
0.8574202274475898	0.8224252904184323	0.4268486972815556	T	T	T
0.0000689269886254	0.8230778528036683	0.4266439750637262	T	T	T
0.0727795173382200	0.1990224382137965	0.4521069299432901	T	T	T
0.2112438964706495	0.1986144724842760	0.4530826774319767	T	T	T
0.3570882482874735	0.2005607876945189	0.4564143360872898	T	T	T
0.5030346861298788	0.1986785999713701	0.4526584476726206	T	T	T
0.6416973467355492	0.1989968998712207	0.4518019320139219	T	T	T
0.7857631227147001	0.1971475587126735	0.4479782969031907	T	T	T
0.9286095915870679	0.1972598763537431	0.4481933443772931	T	T	T
0.0723446271498429	0.4502771009185333	0.4610884507617355	T	T	T
0.2176162244470246	0.4460804682386519	0.4585225429682220	T	T	T
0.3572063850662283	0.4437091400861991	0.4608359075686074	T	T	T
0.4966306471954148	0.4463062116134810	0.4581406056664727	T	T	T
0.6421049256049955	0.4504952783961476	0.4608075774936136	T	T	T
0.7869963391965871	0.4481862312792955	0.4550260265011328	T	T	T
0.9278548516978397	0.4481694131191531	0.4552121735826510	T	T	T
0.0716848336527871	0.6960489830374813	0.4496996510382607	T	T	T
0.2157089802073877	0.6993268086486024	0.4484989894419070	T	T	T
0.3572887631802937	0.6986565385433341	0.4497488253249555	T	T	T
0.4989882353456224	0.6994832610660636	0.4485278715696925	T	T	T
0.6431564298185086	0.6963368862443534	0.4495801003908521	T	T	T
0.7862608128229973	0.6950310639774666	0.4507368884554347	T	T	T
0.9285173960100328	0.6949619233916834	0.4508217279557565	T	T	T
0.0716101616233796	0.9477000877826548	0.4466402538969345	T	T	T
0.2146849997351867	0.9488258395066248	0.4471760027812671	T	T	T
0.3574056134439484	0.9498210624084676	0.4474215745185502	T	T	T
0.5001096512563632	0.9488960198993691	0.4472783787973917	T	T	T
0.6432228483841221	0.9476486572759905	0.4467141026911605	T	T	T
0.7860144236749934	0.9467705405704612	0.4464382295802901	T	T	T
0.9288880890124346	0.9467494251881380	0.4464305173472937	T	T	T
0.9983732596683033	0.1581166578634405	0.5449583570615484	T	T	T
0.1412449552930937	0.1574498581680184	0.5477290129701454	T	T	T
0.2829026943012525	0.1567475320989902	0.5483376809346946	T	T	T
0.4316712178967901	0.1569031414746461	0.5480833388774318	T	T	T
0.5733315159666852	0.1575674511951278	0.5473500866377602	T	T	T
0.7162428423008474	0.1582899858754959	0.5449036450358298	T	T	T
0.8570950939816867	0.1587255777954710	0.5437435260417582	T	T	T
0.9923183440256301	0.4084582879123220	0.5566261934983365	T	T	T
0.7220833761623185	0.4085872998868557	0.5562640575682675	T	T	T
0.8571017059745750	0.4092527763704222	0.5512312665095119	T	T	T
0.0016002407348884	0.6622210993688520	0.5467883876035085	T	T	T
0.1439884929899775	0.6643948032811308	0.5456197124148126	T	T	T
0.2872377739126966	0.6673393705953979	0.5477840084914347	T	T	T
0.4275131264713676	0.6673242265392011	0.5477914483309716	T	T	T
0.5708130154014754	0.6644064354591187	0.5454934311047727	T	T	T
0.7129724457215967	0.6622426310158487	0.5466107739668490	T	T	T
0.8572400776620567	0.6636244835157461	0.5498025438698042	T	T	T
0.9999797536938623	0.9106004186958909	0.5425753810844858	T	T	T

```
  0.1424819378479392  0.9108591870115070  0.5431670602700490   T   T   T
  0.2855741558203180  0.9115887893258757  0.5434003769042859   T   T   T
  0.4292902038011740  0.9116614274903654  0.5435068796253616   T   T   T
  0.5723426579725339  0.9108898305963542  0.5431706472932869   T   T   T
  0.7148934645632825  0.9107120227090686  0.5426612186714647   T   T   T
  0.8574854984397005  0.9105005465859933  0.5428327464633591   T   T   T
  0.0705585559127018  0.0320401799528765  0.5640934393380954   T   T   T
  0.2134379208032008  0.0312193550972010  0.5652486721943871   T   T   T
  0.3573849547125499  0.0314179313051085  0.5656375822327406   T   T   T
  0.5013159705057482  0.0316321468476742  0.5652619668941901   T   T   T
  0.6442914436779414  0.0321972025750467  0.5641187993684573   T   T   T
  0.7865604341432376  0.0325442399932313  0.5634202818555910   T   T   T
  0.9282322554134388  0.0325817579157116  0.5634209834077596   T   T   T
  0.0645999556692497  0.2764903920923127  0.5727798267161117   T   T   T
  0.2126136737693758  0.2733722633434570  0.5796246156124123   T   T   T
  0.3572565753361810  0.2640603359963765  0.5721896785940271   T   T   T
  0.5020098545183135  0.2733196733890963  0.5792188212251601   T   T   T
  0.6497966224431715  0.2765921599449624  0.5722554388744736   T   T   T
  0.7879898968711733  0.2798370396444367  0.5677607734434237   T   T   T
  0.9261626082905351  0.2795811426899167  0.5679871769986546   T   T   T
  0.0745467708483136  0.5412637620087205  0.5704532956327164   T   T   T
  0.2198468562478767  0.5460753861671925  0.5732179569498757   T   T   T
  0.3573032729722444  0.5394720287250437  0.5735106651921498   T   T   T
  0.4948855225075010  0.5460763184922027  0.5730414189596239   T   T   T
  0.6401715528582029  0.5412830349655946  0.5699818482473110   T   T   T
  0.7877680820585751  0.5382696641805448  0.5744223850960829   T   T   T
  0.9266693205925325  0.5381711476256582  0.5745674479089434   T   T   T
  0.0717210293340113  0.7858191310421661  0.5642954072086281   T   T   T
  0.2140005473244739  0.7864271179101791  0.5642164262584257   T   T   T
  0.3573949455752629  0.7881921390474680  0.5648263781250001   T   T   T
  0.5006752642723792  0.7862964555017946  0.5642483687984493   T   T   T
  0.6431469298247411  0.7858598356287902  0.5643520769385569   T   T   T
  0.7857054423524646  0.7851272125076375  0.5649073033139719   T   T   T
  0.9290978198637873  0.7850602924621031  0.5648523049675291   T   T   T
  0.1541758156245930  0.4253796076317078  0.6252032438797680   T   T   T
  0.2728998954161039  0.4210692631446416  0.6265800246051013   T   T   T
  0.4421024962765454  0.4210416454589817  0.6266339762791869   T   T   T
  0.5607634850176830  0.4251516260915257  0.6249964811611434   T   T   T
  0.0953986835135905  0.4415893379787690  0.5476628091300930   T   T   T
  0.2203859984657392  0.4342393986278360  0.5497156984558682   T   T   T
  0.3577302700918323  0.4335144883231877  0.5498579997334435   T   T   T
  0.4940913492497724  0.4343104437587832  0.5495018411546954   T   T   T
  0.6190669537449177  0.4416314608958491  0.5472654976902593   T   T   T
  0.1432895901933716  0.3503817565647565  0.5670460218517126   T   T   T
  0.2924185503182340  0.3491415967731072  0.5683984306550199   T   T   T
  0.4223268830247477  0.3489594295644176  0.5685932767631853   T   T   T
  0.5710014863695598  0.3504719787244815  0.5663787981389445   T   T   T
== Embedded cluster II-Pd_3k+1 k=1
   1.00000000000000
    24.6586885454000111    0.0000000000000000    0.0000000000000000
     0.0000000000000000   25.2576961516000082    0.0000000000000000
     0.0000000000000000    0.0000000000000000   30.0000000000007034
   W    Te   Pd
    56   112     4
```

```
Selective dynamics
Direct
  0.0714531950879982  0.0984021376966546  0.4919282543918378   T   T   T
  0.2133778911499463  0.0982007348017291  0.4917340043377954   T   T   T
  0.3552008991837129  0.0980391705297327  0.4917375650407406   T   T   T
  0.4998104024782064  0.0988294738331843  0.4936841732878280   T   T   T
  0.6429529226655919  0.0989742706792679  0.4937649630449433   T   T   T
  0.7875398874691351  0.0981553868018950  0.4919103410742561   T   T   T
  0.9294884670044242  0.0983259740422052  0.4918491466911784   T   T   T
  0.0714925441473128  0.3490994369442036  0.4932304306829376   T   T   T
  0.2130839997018922  0.3490917300710985  0.4933656832536887   T   T   T
  0.3536734242467166  0.3483218074593162  0.4940052338044398   T   T   T
  0.4902875413277102  0.3447075795849682  0.4968256251191436   T   T   T
  0.6523916439694545  0.3446329852740684  0.4966026746143000   T   T   T
  0.7891954033469535  0.3483595003468483  0.4940167778616500   T   T   T
  0.9299356695392897  0.3490950895446886  0.4933061681539546   T   T   T
  0.0714866123720614  0.5989134634551234  0.4938274428063861   T   T   T
  0.2144951638984301  0.5990159321567230  0.4936603157144620   T   T   T
  0.3572691636138436  0.5996081409105102  0.4932920475475861   T   T   T
  0.4980967888062521  0.6011475434437876  0.4926545115421912   T   T   T
  0.6450280427851834  0.6011745204907312  0.4927732410512255   T   T   T
  0.7857828702665479  0.5996532483375537  0.4933574739981512   T   T   T
  0.9284573705235735  0.5990893675721907  0.4938259294784349   T   T   T
  0.0714489580203562  0.8483930015903188  0.4929830302301001   T   T   T
  0.2139085469255267  0.8485929069079456  0.4928859599927449   T   T   T
  0.3565180791709643  0.8492049388861534  0.4925451887164827   T   T   T
  0.4996052206939777  0.8496120212767866  0.4922122141915148   T   T   T
  0.6430792548980701  0.8497098062045662  0.4923508893920778   T   T   T
  0.7863301231717347  0.8493249129643119  0.4925634323267638   T   T   T
  0.9288727954002107  0.8485956302969085  0.4929197408011725   T   T   T
  0.0003539505941017  0.0092149165237784  0.4985114429273896   T   T   T
  0.1423369700074195  0.0091568988256442  0.4985361047308152   T   T   T
  0.2844006803838500  0.0092637266381192  0.4984814968123552   T   T   T
  0.4277175921212554  0.0099406599142371  0.4988105606130275   T   T   T
  0.5712175922670658  0.0101200881422841  0.4995873723722739   T   T   T
  0.7149933334379103  0.0100374329651651  0.4989743906279460   T   T   T
  0.8581884983654435  0.0093789229683023  0.4985804251798996   T   T   T
  0.0005444624218246  0.2595593939264384  0.4986692556250901   T   T   T
  0.1425203543607462  0.2595535132660522  0.4986787032905042   T   T   T
  0.2840177288402550  0.2593618076389030  0.4987938793505304   T   T   T
  0.4230564334270018  0.2576606965794004  0.4995494784646287   T   T   T
  0.5711822340079936  0.2608920728977152  0.5067357371259166   T   T   T
  0.7199436946020363  0.2577946875551614  0.4996253935589026   T   T   T
  0.8587911868752753  0.2594181620668340  0.4989422128443844   T   T   T
  0.0000948949849058  0.5099527601388731  0.5004277426799689   T   T   T
  0.1429345979226968  0.5098970598782107  0.5004785784095824   T   T   T
  0.2857045801289428  0.5101562361362572  0.5004048358375403   T   T   T
  0.4277356943137412  0.5116284305039255  0.5003299709486621   T   T   T
  0.5716466749956045  0.5167876190008435  0.4981250137037792   T   T   T
  0.7153025309157005  0.5115522657917068  0.5002118368919929   T   T   T
  0.8572430704448157  0.5101838766467561  0.5004532647172711   T   T   T
  0.0001592520380683  0.7595125985568780  0.4998736637042411   T   T   T
  0.1428768263355019  0.7594887969995137  0.4998407644222726   T   T   T
  0.2853091908298279  0.7599300429833474  0.4995776108309286   T   T   T
```

0.4278069504711796	0.7608806325956730	0.4992858536264552	T	T	T
0.5714746774393337	0.7609388192982697	0.4987755574199180	T	T	T
0.7150924578868437	0.7609905066877218	0.4994386254531064	T	T	T
0.8574770330392429	0.7600276129513148	0.4997417423601176	T	T	T
0.1423401446848545	0.0740451879711968	0.4263953396506030	T	T	T
0.2846449811893073	0.0739387047719493	0.4261637030742171	T	T	T
0.4286832100824075	0.0763541187488471	0.4274558855293762	T	T	T
0.5714674132049374	0.0763061953138256	0.4282678480247592	T	T	T
0.7140807684127362	0.0763232506216947	0.4276030935278415	T	T	T
0.8582210425327624	0.0740521010439377	0.4264130962542091	T	T	T
0.0005073967803706	0.0743546441457400	0.4263834209738819	T	T	T
0.1423952307105417	0.3259774197409004	0.4274495807776952	T	T	T
0.2839769326964495	0.3255981012786766	0.4276192775191853	T	T	T
0.4252779179893922	0.3232713887919453	0.4281978417127375	T	T	T
0.5712077179017419	0.3250231412938814	0.4360444608022430	T	T	T
0.7176849306043852	0.3232979784523849	0.4280880951641985	T	T	T
0.8588650610736664	0.3254775558214448	0.4276758821025366	T	T	T
0.0006520522498986	0.3258057829947146	0.4274050679609418	T	T	T
0.1429443189687630	0.5748071432623790	0.4284673169728827	T	T	T
0.2857423341176007	0.5749014982370338	0.4282234128857235	T	T	T
0.4280317283116248	0.5753587570167328	0.4273526060300893	T	T	T
0.5716755575451425	0.5808834239192388	0.4270075680197254	T	T	T
0.7150872094436563	0.5756143684336177	0.4274110205544418	T	T	T
0.8572867382257908	0.5750000670524994	0.4283050662311849	T	T	T
0.9999937262901355	0.5751152629910635	0.4285283147624147	T	T	T
0.1426922908936289	0.8240362344049806	0.4276493524178793	T	T	T
0.2851969063156988	0.8245987250859734	0.4274133570050340	T	T	T
0.4280770930257515	0.8254510920608368	0.4270861700164399	T	T	T
0.5714636179245478	0.8258614503569324	0.4270214937258036	T	T	T
0.7146051734636211	0.8254879243630786	0.4272512343983753	T	T	T
0.8575945425094189	0.8245194871293710	0.4274462077324407	T	T	T
0.0001264268990335	0.8242059677246738	0.4276602068078858	T	T	T
0.0714862716477609	0.1971735213290453	0.4468124775567625	T	T	T
0.2133175765949150	0.1970192667461179	0.4466027947083558	T	T	T
0.3548926828718708	0.1965143570359020	0.4462806653006848	T	T	T
0.5008560880558490	0.1989740796460220	0.4515403355513158	T	T	T
0.6419502140125725	0.1991596194349224	0.4517067779344009	T	T	T
0.7878486591805481	0.1966233557218869	0.4464486179191061	T	T	T
0.9296383685740692	0.1971128616851189	0.4468591296228543	T	T	T
0.0715820283850284	0.4478694081789145	0.4488797323414909	T	T	T
0.2139072459947472	0.4479002304959428	0.4489287156910246	T	T	T
0.3565660282963209	0.4473894342599101	0.4495553571733718	T	T	T
0.4992621749087118	0.4461956884063727	0.4530267138610127	T	T	T
0.6436122545388482	0.4463003907155160	0.4528041777335544	T	T	T
0.7865818548076662	0.4474223841239758	0.4494597474739968	T	T	T
0.9291404270918892	0.4479132405178706	0.4489256529090264	T	T	T
0.0714589442253020	0.6971547128317176	0.4486728668358454	T	T	T
0.2141775036638632	0.6973326875186664	0.4484139341640876	T	T	T
0.3568710431118477	0.6980788556433234	0.4481212885832866	T	T	T
0.4991515985189235	0.6993966446306498	0.4476467712025258	T	T	T
0.6438148765942002	0.6994179387391011	0.4476973703244025	T	T	T
0.7861023549800664	0.6982244273401890	0.4481497333913753	T	T	T
0.9287208934701764	0.6974198471818510	0.4486988603276169	T	T	T
0.0713787459605481	0.9465441252707459	0.4473551434797878	T	T	T

0.2136125491228051	0.9466386017281979	0.4472322283116562	T	T	T
0.3566651279131801	0.9474236218695670	0.4473302936562972	T	T	T
0.4999503772560069	0.9485057833533291	0.4475397603525970	T	T	T
0.6428133977606424	0.9485002635901656	0.4477237677274536	T	T	T
0.7860143067134931	0.9476850451877484	0.4473932052435366	T	T	T
0.9287887195194294	0.9466907269550821	0.4473395558423679	T	T	T
0.0005254027725996	0.1610407827980226	0.5433832769137428	T	T	T
0.1425163156143323	0.1608921028086419	0.5434148382517136	T	T	T
0.2841378226107941	0.1606195515846110	0.5431193024279107	T	T	T
0.4257571992728497	0.1594364719705733	0.5443015138818257	T	T	T
0.5713269394814887	0.1593124484871186	0.5464833835175578	T	T	T
0.7170077052112288	0.1596118965550409	0.5444682973386583	T	T	T
0.8587053969241150	0.1606959034633550	0.5433921558571756	T	T	T
0.0005458742601647	0.4114011151218541	0.5452968868199061	T	T	T
0.1423952265953867	0.4112676209973927	0.5453343030148715	T	T	T
0.2843566747773773	0.4114311029367445	0.5456531126223554	T	T	T
0.4285474372189883	0.4126333656433773	0.5472495637327988	T	T	T
0.7141847012169379	0.4125051932650486	0.5470713245048512	T	T	T
0.8585927136843027	0.4113835876244202	0.5456215156757978	T	T	T
0.0000652509281165	0.6612402976788653	0.5451324025048235	T	T	T
0.1430204896618091	0.6612002600622707	0.5450313255576473	T	T	T
0.2858324512734440	0.6616864224875368	0.5446832408742940	T	T	T
0.4280861205961872	0.6627722562398883	0.5446048009371385	T	T	T
0.5715299485103981	0.6625497802292853	0.5429390569620198	T	T	T
0.7149919903864382	0.6627018493414788	0.5447296033399601	T	T	T
0.8570820191369868	0.6617649834362048	0.5448604956110812	T	T	T
0.0001008907590174	0.9110741229018039	0.5441165460266149	T	T	T
0.1426607348635250	0.9109634539551166	0.5441234691537257	T	T	T
0.2852164869581262	0.9112359361061110	0.5439607768771871	T	T	T
0.4281662761338117	0.9115069090632303	0.5436447467335158	T	T	T
0.5712590343090733	0.9112790674971590	0.5438040417988388	T	T	T
0.7146143570566394	0.9116037446043295	0.5436546866724016	T	T	T
0.8575400069806480	0.9112831327167472	0.5439680217681560	T	T	T
0.0713548838630629	0.0334011127878768	0.5640328205777460	T	T	T
0.2134015675206062	0.0334140030239152	0.5639989142643783	T	T	T
0.3559786823118367	0.0333071712950322	0.5639341138101321	T	T	T
0.4991144075916863	0.0328956689699891	0.5647621883738356	T	T	T
0.6433644440617878	0.0329596092879587	0.5647138573586462	T	T	T
0.7867199442099959	0.0333626696592789	0.5640699196820333	T	T	T
0.9290903044541843	0.0334650076815448	0.5639929193589466	T	T	T
0.0714737151353109	0.2828471508325444	0.5644459389436788	T	T	T
0.2129458804604821	0.2828526990739477	0.5646071772073368	T	T	T
0.3532382526808022	0.2827044321045369	0.5655773125653725	T	T	T
0.4919857360292726	0.2756710386079443	0.5697046106059015	T	T	T
0.6505861382860405	0.2757055652111282	0.5695786029752512	T	T	T
0.7896271884458410	0.2829666191870149	0.5657199759660999	T	T	T
0.9299927682271376	0.2829583593890868	0.5645375849701122	T	T	T
0.0715152039112182	0.5341462969690961	0.5658288470449665	T	T	T
0.2141845498677418	0.5342662237534440	0.5657657789224035	T	T	T
0.3567805010118210	0.5349230260966124	0.5658065066510591	T	T	T
0.5009787102621251	0.5342985947575845	0.5657966367614821	T	T	T
0.6421867843565631	0.5339544866658155	0.5658493632214948	T	T	T
0.7861294037886033	0.5349105871887899	0.5657859404936754	T	T	T
0.9287474212410496	0.5340830118494223	0.5658030056465451	T	T	T

```
  0.0715416657140674  0.7836531440666138  0.5651869045653810   T   T   T
  0.2141939951624446  0.7839166308974485  0.5651250825390677   T   T   T
  0.3567630553526170  0.7846635960967089  0.5648371551078066   T   T   T
  0.4999565744175518  0.7847534273479572  0.5641899509692413   T   T   T
  0.6428663752886177  0.7849444233952249  0.5643311279593863   T   T   T
  0.7861418675650530  0.7850549653114680  0.5649476068142814   T   T   T
  0.9288058517134167  0.7839352161430636  0.5652022102302713   T   T   T
  0.5712794067128611  0.3878445914281463  0.6557529154310640   T   T   T
  0.5712598967391085  0.3409610027394591  0.5783987557993110   T   T   T
  0.6276479676158617  0.4355609414202856  0.5957314342484795   T   T   T
  0.5153616637468604  0.4358930506221318  0.5956102744364945   T   T   T
  0.5714471151136232  0.4169577298969211  0.5198576319227792   T   T   T
== Embedded cluster II-Pd_3k+1 k=2
   1.00000000000000
     24.6586885454000111    0.0000000000000000    0.0000000000000000
      0.0000000000000000   25.2576961516000082    0.0000000000000000
      0.0000000000000000    0.0000000000000000   30.0000000000007034
   W     Te    Pd
    56   112     7
Selective dynamics
Direct
  0.0700633590886485  0.0984277711727274  0.4940678290638749   T   T   T
  0.2107983279735320  0.0980169047366587  0.4945009511384918   T   T   T
  0.3528652936770766  0.0970337618024984  0.4952332388312735   T   T   T
  0.5000236734359829  0.1018604267492488  0.4997031155495529   T   T   T
  0.6472101374639875  0.0970771904683106  0.4952980223343387   T   T   T
  0.7893074162271433  0.0980609859593416  0.4945354367293408   T   T   T
  0.9300243748007374  0.0984036128822523  0.4940542476267205   T   T   T
  0.0693270556747001  0.3497656442744624  0.4984312490408543   T   T   T
  0.2067460723074761  0.3484589620734164  0.4987480726135276   T   T   T
  0.3430448986071105  0.3478123924898839  0.4991617274264442   T   T   T
  0.5000337966721341  0.3083991345475733  0.4902805050642851   T   T   T
  0.6570807898765271  0.3478251852002500  0.4992605204513831   T   T   T
  0.7934188894956842  0.3484428741461440  0.4988090516092344   T   T   T
  0.9309384246592046  0.3498215389674951  0.4985069308413759   T   T   T
  0.0702628529209026  0.6000604495466194  0.4979914762578977   T   T   T
  0.2093355231646740  0.6004603617832808  0.4986820529475021   T   T   T
  0.3625118107798063  0.6021818671808802  0.4935465760827336   T   T   T
  0.5000273672714433  0.6090215720932342  0.4905631987256467   T   T   T
  0.6375895080702173  0.6021725162608766  0.4935650658951838   T   T   T
  0.7906790056991413  0.6004462596908988  0.4986684668723010   T   T   T
  0.9297767776655671  0.6000599374757138  0.4979872422963725   T   T   T
  0.0706790676923120  0.8489011027608461  0.4933926113795978   T   T   T
  0.2122107824478792  0.8490874276987024  0.4933708568306509   T   T   T
  0.3555848759127298  0.8510479413810944  0.4931054162667631   T   T   T
  0.5000232793110770  0.8525796331916761  0.4926388769049769   T   T   T
  0.6444787028070178  0.8510627888797073  0.4931406939224105   T   T   T
  0.7878204142897456  0.8491243652313580  0.4934031753221940   T   T   T
  0.9293648536714297  0.8489120469060787  0.4934290363928271   T   T   T
  0.0000449182456936  0.0089326591893241  0.4992906287206369   T   T   T
  0.1406443848666431  0.0090286545524630  0.4997148226620125   T   T   T
  0.2824087164253442  0.0093521331337191  0.5006311614578788   T   T   T
  0.4284208796166921  0.0116379736910811  0.5020899484024255   T   T   T
  0.5716610959325048  0.0116592901888115  0.5021225155079334   T   T   T
```

0.7176020146589932	0.0093954962232452	0.5006435550876014	T	T	T
0.8594094070130289	0.0090445101915134	0.4997243832251493	T	T	T
0.0000734009009971	0.2595896893602891	0.5022673511456541	T	T	T
0.1392637960743877	0.2590362674898262	0.5023149658657783	T	T	T
0.2783330646532018	0.2574306937761640	0.5037841904441069	T	T	T
0.4113584335088013	0.2549562883885187	0.5078844177453850	T	T	T
0.5887248933537647	0.2549571138822612	0.5079764831496121	T	T	T
0.7218409038480240	0.2574665780187916	0.5039266895994680	T	T	T
0.8609779946770174	0.2590989408921586	0.5024588016316924	T	T	T
0.0000835446259447	0.5108718632709393	0.5049901991251128	T	T	T
0.1400046496052419	0.5107811643964952	0.5058589173894571	T	T	T
0.2787892781544837	0.5122309079757243	0.5081390145188857	T	T	T
0.4391167025502927	0.5168333996905572	0.4956430910753641	T	T	T
0.5610269133385951	0.5168423520204267	0.4956816327818506	T	T	T
0.7213267337417568	0.5121548107326298	0.5081003201715851	T	T	T
0.8601106194902717	0.5107822826511841	0.5058584462979001	T	T	T
0.0000220559940779	0.7601888977021874	0.5010186695759153	T	T	T
0.1416007408096702	0.7603812130888323	0.5011471747829903	T	T	T
0.2843692096872273	0.7612603404619912	0.5005384100974362	T	T	T
0.4279104627380969	0.7645239260140466	0.4993916464579175	T	T	T
0.5721330500120263	0.7645332289566351	0.4994040920894572	T	T	T
0.7156872302905392	0.7612860326453159	0.5005638716713718	T	T	T
0.8584319508462561	0.7604078233599058	0.5011635658988557	T	T	T
0.1409560431371824	0.0749156138168129	0.4281995406858888	T	T	T
0.2826900648752874	0.0748596820892285	0.4289804961512833	T	T	T
0.4291738586296484	0.0798900536526769	0.4322658799880002	T	T	T
0.5709095137579768	0.0798746198600321	0.4322980081113489	T	T	T
0.7173729090938200	0.0749794043788008	0.4290112676934423	T	T	T
0.8591194461617909	0.0749179283600104	0.4281879681762376	T	T	T
0.0000498070430683	0.0750585998510413	0.4278535046865273	T	T	T
0.1385269261108032	0.3264994409388797	0.4316689041094388	T	T	T
0.2761898178177237	0.3233440996203423	0.4325889086013960	T	T	T
0.4151745221919930	0.3169284256239597	0.4319495210572604	T	T	T
0.5849645273862096	0.3169072866379801	0.4320787437264406	T	T	T
0.7238867097459076	0.3233438520406380	0.4326798406782529	T	T	T
0.8616723889770614	0.3264822946394256	0.4317569153672925	T	T	T
0.0000796021778078	0.3269616425560231	0.4316808622617893	T	T	T
0.1410963551515038	0.5742433068507070	0.4325369075284231	T	T	T
0.2835360403330604	0.5739338327478927	0.4350831652502510	T	T	T
0.4288087684153787	0.5833343154198136	0.4251794316057838	T	T	T
0.5712442097118882	0.5833452641037337	0.4252181630693864	T	T	T
0.7164715973477263	0.5738955059786712	0.4350663035999053	T	T	T
0.8589235202390980	0.5742347257141809	0.4325385297213359	T	T	T
0.0000105975153901	0.5748869746366712	0.4323195124228559	T	T	T
0.1417101556722827	0.8237525972040762	0.4279530425971187	T	T	T
0.2841975562215188	0.8255408127823456	0.4281465373340014	T	T	T
0.4278467970313099	0.8294086294762928	0.4275570583156346	T	T	T
0.5722482939942695	0.8294464402483125	0.4275911089002655	T	T	T
0.7158488501607478	0.8255652839325851	0.4281629246802937	T	T	T
0.8583229327054133	0.8238283359105805	0.4279875114290446	T	T	T
0.9999983150435231	0.8239073648893515	0.4279633378104973	T	T	T
0.0697094308739934	0.1971278114214478	0.4491592600370289	T	T	T
0.2098805057936213	0.1970020744249613	0.4497975794574650	T	T	T
0.3502973596846660	0.1959979599905258	0.4504341464632867	T	T	T

0.5000123802076974	0.2041823023671476	0.4563554961788401	T	T	T
0.6498234906091302	0.1960089011311320	0.4505317355750287	T	T	T
0.7903168877659981	0.1971303104348347	0.4500002958765804	T	T	T
0.9303924891008160	0.1971238099837842	0.4491976125246941	T	T	T
0.0706365903436690	0.4483282591839210	0.4536621933393089	T	T	T
0.2117459970195889	0.4488727169620065	0.4556188740405991	T	T	T
0.3515279536056081	0.4509616025296456	0.4543097666701240	T	T	T
0.5000802979635618	0.4690744746942055	0.4298815840109957	T	T	T
0.6485840387223650	0.4508919405988946	0.4542778157326041	T	T	T
0.7885166978252901	0.4488032688813006	0.4555640387736306	T	T	T
0.9295686705535140	0.4483200371548444	0.4536886955235139	T	T	T
0.0710502601811001	0.6967262550218120	0.4506750770465289	T	T	T
0.2127634913146157	0.6969423396551333	0.4508655018643970	T	T	T
0.3564247003421663	0.7005654450260023	0.4491867594663546	T	T	T
0.5000174520658414	0.7061006146187429	0.4462963750668579	T	T	T
0.6436301451677129	0.7005978733520579	0.4492194830094577	T	T	T
0.7872752230684866	0.6969492090485924	0.4508668468883245	T	T	T
0.9289658076969771	0.6967343862812289	0.4506859953443066	T	T	T
0.0709412067665146	0.9467008288559898	0.4474783021061230	T	T	T
0.2127149004466763	0.9474073766941433	0.4481199441891338	T	T	T
0.3565724613736978	0.9500697475384833	0.4496324598536879	T	T	T
0.5000392704779526	0.9519359024655929	0.4493030734938613	T	T	T
0.6434389902030391	0.9500620168035389	0.4496397689657176	T	T	T
0.7873010314007562	0.9474537000540096	0.4481781164608741	T	T	T
0.9291138092248697	0.9467009382588171	0.4475381774271071	T	T	T
0.0000511318775559	0.1606088783360967	0.5464961964588683	T	T	T
0.1402211210505776	0.1600819312124107	0.5466090339427222	T	T	T
0.2813350819667182	0.1588473264084866	0.5474247275167298	T	T	T
0.4217049314073891	0.1567860178473008	0.5507877845293664	T	T	T
0.5783368670885523	0.1567778677476853	0.5508177876957749	T	T	T
0.7188669758961415	0.1588666988919550	0.5475613112044494	T	T	T
0.8600342590953168	0.1600316373687252	0.5466787950242160	T	T	T
0.0001966879015296	0.4126813052324630	0.5507074068590251	T	T	T
0.1395319289806277	0.4121229766926537	0.5515397799096139	T	T	T
0.2754577943926849	0.4104528894994201	0.5561371743558277	T	T	T
0.7246629436266189	0.4104518848528082	0.5561785735202882	T	T	T
0.8606033252332255	0.4121312271856252	0.5515571167988852	T	T	T
0.0000132311197509	0.6634476128944026	0.5486665104959060	T	T	T
0.1411579613522701	0.6636610853928135	0.5494235702728973	T	T	T
0.2866895248052683	0.6620479534159674	0.5454370797268915	T	T	T
0.4295110290785485	0.6671993483888442	0.5447698352615292	T	T	T
0.5705306587340759	0.6672123859141299	0.5447772279489768	T	T	T
0.7133735097532918	0.6620695061267199	0.5454145836634227	T	T	T
0.8588949565530390	0.6636641643433568	0.5494089121581708	T	T	T
0.0000352120253740	0.9111946253964656	0.5447197068481502	T	T	T
0.1416301186328044	0.9109906139574322	0.5448295603871657	T	T	T
0.2837327958699272	0.9109507119032807	0.5449813811356428	T	T	T
0.4279155192742879	0.9122410996231535	0.5449120699900557	T	T	T
0.5720922134111269	0.9122409319756634	0.5449190113258313	T	T	T
0.7163125687494721	0.9110040383576495	0.5450222633782439	T	T	T
0.8584183843191675	0.9110349366323419	0.5448730802986811	T	T	T
0.0702488944371452	0.0323485245082001	0.5654906108670906	T	T	T
0.2111870572720100	0.0322012064943698	0.5660086897286178	T	T	T
0.3543187976781865	0.0313963096720017	0.5665772583678422	T	T	T

```
  0.5000054943279589  0.0326025991467891  0.5681953404499689   T   T   T
  0.6457505624667276  0.0314182645133037  0.5666214763684364   T   T   T
  0.7888345839189240  0.0322027237510067  0.5659997503843215   T   T   T
  0.9298250507822136  0.0323397328281167  0.5654770287610648   T   T   T
  0.0697128140032259  0.2821272381570840  0.5689907053147949   T   T   T
  0.2083646132435046  0.2817313892764426  0.5695847974483984   T   T   T
  0.3459201918762647  0.2858063765172705  0.5738868720632461   T   T   T
  0.5000516293653826  0.2620358659547445  0.5717742374808341   T   T   T
  0.6541882297496201  0.2858289005041183  0.5739855695551108   T   T   T
  0.7918442513474099  0.2817674508219171  0.5697131763342015   T   T   T
  0.9305903731735622  0.2822139257060169  0.5690973875198813   T   T   T
  0.0697247684569095  0.5365046169841863  0.5711235719235923   T   T   T
  0.2095541608790821  0.5385967381536857  0.5726162770122358   T   T   T
  0.3590768674594862  0.5429525758028074  0.5671439790286660   T   T   T
  0.5000182306997918  0.5477790092334690  0.5653207683906998   T   T   T
  0.6410835692782572  0.5429251535739759  0.5671470037945852   T   T   T
  0.7905251113760898  0.5385687534986520  0.5726045396384533   T   T   T
  0.9304069938922256  0.5365033068561251  0.5711089169385076   T   T   T
  0.0708752539952046  0.7853964251020642  0.5663533081061729   T   T   T
  0.2134151442339467  0.7848072210800927  0.5660315741388461   T   T   T
  0.3561721320365111  0.7865764136817471  0.5651773235370469   T   T   T
  0.5000087241312494  0.7878449439285623  0.5644099986257305   T   T   T
  0.6438652517780452  0.7865748817109497  0.5652008909447122   T   T   T
  0.7866192486490953  0.7848530930855949  0.5660614512832153   T   T   T
  0.9291947196717329  0.7853622750450837  0.5663580908156384   T   T   T
  0.4288012687374980  0.4234228118705481  0.6149309959181695   T   T   T
  0.5712261636316753  0.4234150399083508  0.6149182152468352   T   T   T
  0.3755752412060710  0.4444388964981750  0.5418277522760908   T   T   T
  0.5000311233931206  0.4414433455347313  0.5519072270694274   T   T   T
  0.6245389918012423  0.4444283466341293  0.5418569752836723   T   T   T
  0.4380008750872361  0.3461193975165467  0.5579620698696621   T   T   T
  0.5620625247629388  0.3461187533681484  0.5579892102002825   T   T   T
  0.4445545608582255  0.4020259531939186  0.4778334670438286   T   T   T
  0.5555693136645993  0.4020355831742095  0.4778724602702988   T   T   T
== Embedded cluster II-Pd_3k+1 k=3
   1.00000000000000
    24.6586885454000111    0.0000000000000000    0.0000000000000000
     0.0000000000000000   25.2576961516000082    0.0000000000000000
     0.0000000000000000    0.0000000000000000   30.0000000000007034
   W    Te   Pd
    56   112    10
Selective dynamics
Direct
  0.0680664072728176  0.0983104665865370  0.4964362149680788   T   T   T
  0.2090385845568951  0.0981046456061839  0.4977583824661981   T   T   T
  0.3468901748466916  0.0954788993785870  0.4983211090498698   T   T   T
  0.5103342796584237  0.1005094534761697  0.5022981057716733   T   T   T
  0.6497674886875653  0.0966362493206197  0.4962909235069534   T   T   T
  0.7892624347151288  0.0977376468684952  0.4953643969885359   T   T   T
  0.9287813502834324  0.0982107061510433  0.4953320855680223   T   T   T
  0.0655928515360450  0.3493072150437046  0.5002365652512644   T   T   T
  0.1991495992381254  0.3488312979040599  0.5011021791211746   T   T   T
  0.3594714103060533  0.3365664691816488  0.5029079622486125   T   T   T
  0.4990328517966307  0.3067412653006906  0.4899969728125229   T   T   T
```

0.6567566339397192	0.3477731474620278	0.4998475424098461	T	T	T
0.7923995677388608	0.3486054613309005	0.5000624311959335	T	T	T
0.9289674197042872	0.3501631490651237	0.5002558925383344	T	T	T
0.0656354015242447	0.6006904363413488	0.5009811497856308	T	T	T
0.2218558444760008	0.6022648601708372	0.4947978091179184	T	T	T
0.3598102016429496	0.6091430869936365	0.4914343818299656	T	T	T
0.4980302284561267	0.6095466460731127	0.4914254202093142	T	T	T
0.6383285448216405	0.6020552274623657	0.4945479635620113	T	T	T
0.7890645555014870	0.6001827781387131	0.4996865027626290	T	T	T
0.9280559442904565	0.6000303329283155	0.4999541961031585	T	T	T
0.0702119836447023	0.8486743178591174	0.4949408605516962	T	T	T
0.2120162343165944	0.8502104892857222	0.4946283257708531	T	T	T
0.3564460856583176	0.8540576069772646	0.4952244024273477	T	T	T
0.5009948380336903	0.8536896902104559	0.4947026440571440	T	T	T
0.6451692950401592	0.8503089588873786	0.4942724735218437	T	T	T
0.7877404334415614	0.8486194013115119	0.4944187636361940	T	T	T
0.9289859043066098	0.8486465895300899	0.4946581643426225	T	T	T
0.9988925388424338	0.0087131322944047	0.5010732911580013	T	T	T
0.1392453730444147	0.0092971429911336	0.5022879419029388	T	T	T
0.2784372935477629	0.0091468823505133	0.5033579743121673	T	T	T
0.4327102353758580	0.0142794359589092	0.5071385453819164	T	T	T
0.5757013991084980	0.0104092413376623	0.5037760159631426	T	T	T
0.7183095185440953	0.0084756938013101	0.5011294346680358	T	T	T
0.8587736737038307	0.0085677231453797	0.5005026190359843	T	T	T
0.9977786604416204	0.2594912762327731	0.5037302126047599	T	T	T
0.1362425146055657	0.2580666347444071	0.5052335142651799	T	T	T
0.2803517794547948	0.2576630353711832	0.5115402850943580	T	T	T
0.4143051462273704	0.2475968578918504	0.5074967252736601	T	T	T
0.5900556264674569	0.2553287057874211	0.5087852434447369	T	T	T
0.7214999850850731	0.2576024840502425	0.5048796487278442	T	T	T
0.8594533040620199	0.2590685359739506	0.5035660141234278	T	T	T
0.9979624819934713	0.5110364549551868	0.5080914527132437	T	T	T
0.1366138375240822	0.5136005168495834	0.5102160850750874	T	T	T
0.2967819852483680	0.5168122894780260	0.4971904706602543	T	T	T
0.4307107886701981	0.5195499887135048	0.4936821276295613	T	T	T
0.5602357576442640	0.5170475518491854	0.4957429158805548	T	T	T
0.7195912089610047	0.5118535864820282	0.5084205370980179	T	T	T
0.8584545781443335	0.5108133325776472	0.5073534622273360	T	T	T
0.9992151311547800	0.7602922629523787	0.5027944742819822	T	T	T
0.1418701431197950	0.7604488200198047	0.5017843438882400	T	T	T
0.2850754455542081	0.7647583667608563	0.5010785754182768	T	T	T
0.4285443959646374	0.7669756663076613	0.5006218260830734	T	T	T
0.5720363769719713	0.7646372927670886	0.5009854682336861	T	T	T
0.7158001309925851	0.7607755016683402	0.5017842317535266	T	T	T
0.8580240928045704	0.7601360144854692	0.5024151381324757	T	T	T
0.1395018904618181	0.0755439004320601	0.4309212610304949	T	T	T
0.2797543134742749	0.0731995595256610	0.4308688002478042	T	T	T
0.4307194266578674	0.0815278875811676	0.4386969216376698	T	T	T
0.5744415369706648	0.0770908804831489	0.4325668155132836	T	T	T
0.7183936391625364	0.0740753975719009	0.4293970825021807	T	T	T
0.8588675304043087	0.0745452208186405	0.4289263979198606	T	T	T
0.9992336984557862	0.0746232674264464	0.4295248359071167	T	T	T
0.1348731351875047	0.3229077604010519	0.4334333010567116	T	T	T
0.2781257321173599	0.3186603567191897	0.4382636313115532	T	T	T

0.4190806556386257	0.3127817370066098	0.4287333534475520	T	T	T
0.5843044946855059	0.3166889005936566	0.4326464750810892	T	T	T
0.7234589879896215	0.3232016992266977	0.4333757256136768	T	T	T
0.8605445127263235	0.3267153183386199	0.4330142046530237	T	T	T
0.9975038664076212	0.3270248129689073	0.4331167278825793	T	T	T
0.1407732719190289	0.5754396609943362	0.4374637297549226	T	T	T
0.2878641881932753	0.5822707761087583	0.4264884636675980	T	T	T
0.4288333132380207	0.5893902347104680	0.4243566457614100	T	T	T
0.5708638561661241	0.5850867981795721	0.4266604082860982	T	T	T
0.7160686791882779	0.5738314531616491	0.4352770546185568	T	T	T
0.8583070713757711	0.5743438340840885	0.4340237992726738	T	T	T
0.9986334516087393	0.5740786126732209	0.4344273981071928	T	T	T
0.1414694781344513	0.8244838502386450	0.4293349151268228	T	T	T
0.2851938141517358	0.8303952762773426	0.4295219065103247	T	T	T
0.4283553817391061	0.8328876675875939	0.4293830149014291	T	T	T
0.5722471003689400	0.8296317869074210	0.4290904753713732	T	T	T
0.7160470406547237	0.8247656692136532	0.4291120354962064	T	T	T
0.8583665455179315	0.8233183602180848	0.4290707603835520	T	T	T
0.9998367037341014	0.8234138921112880	0.4293934807174385	T	T	T
0.0679704142827493	0.1966242834610375	0.4513656014058481	T	T	T
0.2104061821001896	0.1975491301956470	0.4554580627747903	T	T	T
0.3473029328164329	0.1928675016479186	0.4536268552392626	T	T	T
0.5041368366418827	0.2013452679442352	0.4577961468895344	T	T	T
0.6506997528252284	0.1956268283472511	0.4513818293807818	T	T	T
0.7897655572260815	0.1966705333261061	0.4507501007138811	T	T	T
0.9288541727810122	0.1969054942265142	0.4502973586439559	T	T	T
0.0688977307617441	0.4492086250201988	0.4576057914186428	T	T	T
0.2018229704629600	0.4515343652282409	0.4529386748001482	T	T	T
0.3615913078362722	0.4663548234444356	0.4347714474921856	T	T	T
0.5001810119498049	0.4706075449291574	0.4309870331603616	T	T	T
0.6472725109578229	0.4503400450299803	0.4539421522787970	T	T	T
0.7876011494113742	0.4485917054654196	0.4564619407902373	T	T	T
0.9286157054406680	0.4486041527389952	0.4556291538570166	T	T	T
0.0697992900216263	0.6964921887787779	0.4525491773898423	T	T	T
0.2147144023654904	0.7004501238937610	0.4501765381678368	T	T	T
0.3571498735637932	0.7070969436154619	0.4476138026138320	T	T	T
0.5000710102973094	0.7073560082026251	0.4476635294682302	T	T	T
0.6435216327759405	0.7006930868018690	0.4504240712355244	T	T	T
0.7870980838305324	0.6967593236996680	0.4519046450747906	T	T	T
0.9287827761868570	0.6965694037971687	0.4521811027682716	T	T	T
0.0703622226744496	0.9467489649595704	0.4494526724337926	T	T	T
0.2109948011430913	0.9484044403087244	0.4495331905069571	T	T	T
0.3576121808343500	0.9540332721262360	0.4538318299303062	T	T	T
0.5006711222513850	0.9533410869557192	0.4520191113458244	T	T	T
0.6446361483580384	0.9487095674939161	0.4499743796297094	T	T	T
0.7878926077697563	0.9464635640459743	0.4485940979710716	T	T	T
0.9292441321496236	0.9462399478945500	0.4486748246497237	T	T	T
0.9978131718951605	0.1604734644138623	0.5482581629774924	T	T	T
0.1381635984982076	0.1593635842815118	0.5499548182561912	T	T	T
0.2808543217139160	0.1565610740160726	0.5521744671158546	T	T	T
0.4255096843430761	0.1496566287040653	0.5511756401856910	T	T	T
0.5831089077565623	0.1570110690387193	0.5535520550612888	T	T	T
0.7196267946131564	0.1589672317972922	0.5489897828237379	T	T	T
0.8588403891004580	0.1601042406069510	0.5477948464252357	T	T	T

0.9974429704299553	0.4124574898854373	0.5534543575684161	T	T	T
0.1328738460950501	0.4102242764316580	0.5580615171431726	T	T	T
0.7228987519820516	0.4105003726815347	0.5566693673494837	T	T	T
0.8587880415938395	0.4124278752082299	0.5532387709705248	T	T	T
0.9981357181252620	0.6639602818192016	0.5516411768364666	T	T	T
0.1445610979334683	0.6613651966669414	0.5462549032008595	T	T	T
0.2882800374102744	0.6666226559909991	0.5460201894225979	T	T	T
0.4287092145679006	0.6694525844881980	0.5451418853283997	T	T	T
0.5701857174567593	0.6664487389374664	0.5455589966529300	T	T	T
0.7131789900724319	0.6620757899486301	0.5468403872398360	T	T	T
0.8576841157758570	0.6637653565490985	0.5506386942840394	T	T	T
0.9995148860109518	0.9107696931824989	0.5463805758140298	T	T	T
0.1408501081271578	0.9107952070750756	0.5467190582265430	T	T	T
0.2826948061435011	0.9115847361193340	0.5475308445370158	T	T	T
0.4295557454134711	0.9138724268777090	0.5473494656359872	T	T	T
0.5742537614756932	0.9118632658465580	0.5467644755618937	T	T	T
0.7168364857916327	0.9106385355628630	0.5459499166133214	T	T	T
0.8581992164653337	0.9108455206448989	0.5458663981144080	T	T	T
0.0683968655227841	0.0321150167971939	0.5678518148669036	T	T	T
0.2089597848564652	0.0323434959389520	0.5694562598950607	T	T	T
0.3534442471551949	0.0303620395773097	0.5690709783831565	T	T	T
0.5059068446018284	0.0324696442717561	0.5718996327079120	T	T	T
0.6487344218814423	0.0315418674459438	0.5682346915948554	T	T	T
0.7888607556815987	0.0316933106226207	0.5668920984261600	T	T	T
0.9284972981019303	0.0321487705630825	0.5668422084104565	T	T	T
0.0663882055049582	0.2821194070466284	0.5711105511130682	T	T	T
0.2047303979391673	0.2800448275685259	0.5743556691682734	T	T	T
0.3548621599319111	0.2757062858222002	0.5805475143171379	T	T	T
0.5043113140561460	0.2591525251082517	0.5714017077971713	T	T	T
0.6541566162371181	0.2874975224692980	0.5750763809124719	T	T	T
0.7907451890285987	0.2819881414232996	0.5711095221269710	T	T	T
0.9284075241746129	0.2818310315587250	0.5705637185999309	T	T	T
0.0672044932842219	0.5389750851201779	0.5751475966090280	T	T	T
0.2174024300621722	0.5448259912620598	0.5691441441597405	T	T	T
0.3618539991350579	0.5442681772953295	0.5636346694805598	T	T	T
0.4973358491301572	0.5454004349261240	0.5646502128440194	T	T	T
0.6402128714513537	0.5426697916456922	0.5681331695455127	T	T	T
0.7881790657841959	0.5382246986279099	0.5736112650938298	T	T	T
0.9276523470638137	0.5370350049330949	0.5734196371185326	T	T	T
0.0711648483922442	0.7842727465389079	0.5674906692945818	T	T	T
0.2131399280764718	0.7854509481602585	0.5667912186799596	T	T	T
0.3566748523716851	0.7886008065512006	0.5663949040662621	T	T	T
0.5005332636388332	0.7887237129472211	0.5663204319898023	T	T	T
0.6441277315300016	0.7862370952527229	0.5665959910880506	T	T	T
0.7864739763672486	0.7848586804189958	0.5673448901081686	T	T	T
0.9284157165496876	0.7856436625876757	0.5678365842713485	T	T	T
0.2986600850808130	0.4308847825058473	0.6164262941978566	T	T	T
0.4215768866018047	0.4223382722973756	0.6180417495916505	T	T	T
0.5659199174162781	0.4259474290479679	0.6126642360251048	T	T	T
0.2320355753045870	0.4478858792541212	0.5440300753772946	T	T	T
0.3659725272906387	0.4341417085755022	0.5430815235539638	T	T	T
0.4919414854080403	0.4420425414249581	0.5481499597832642	T	T	T
0.6224222652746482	0.4451139102604855	0.5412572076335240	T	T	T
0.2834937999367838	0.3527124915547752	0.5626986230729247	T	T	T

```
  0.4419279202745108  0.3453863531948076  0.5623824412654054   T   T   T
  0.5603915238516654  0.3475356259098665  0.5574793110051062   T   T   T
  0.2937569077558982  0.4099115706732908  0.4781133890915223   T   T   T
  0.4368954188365001  0.4032976106828086  0.4755104022051277   T   T   T
  0.5528071883156830  0.4020114113544510  0.4778816914154650   T   T   T
== Embedded cluster II-Pd_3k+1 k=4
   1.00000000000000
    24.6586885454000111    0.0000000000000000    0.0000000000000000
     0.0000000000000000   25.2576961516000082    0.0000000000000000
     0.0000000000000000    0.0000000000000000   30.0000000000007034
   W    Te   Pd
    56   112    13
Selective dynamics
Direct
  0.0684874192833047  0.0976571472173669  0.4994949180516463   T   T   T
  0.2083972954313702  0.0958819109874425  0.4999996580309843   T   T   T
  0.3484447494415015  0.0991784822455377  0.5034979298123704   T   T   T
  0.5123853229272848  0.0949084963455758  0.4987599907549627   T   T   T
  0.6485708869707054  0.0979999200029433  0.4984186801990519   T   T   T
  0.7882603982947644  0.0981947906799106  0.4978519841527399   T   T   T
  0.9273280233312876  0.0979691648928785  0.4981615956966675   T   T   T
  0.0570412256236979  0.3491422872117419  0.5020719353077561   T   T   T
  0.2155151442872886  0.3376485922512263  0.5035540645171048   T   T   T
  0.3573472412207646  0.3055135793857708  0.4905034214352120   T   T   T
  0.4990387547801974  0.3365502943528029  0.5022855231470595   T   T   T
  0.6572412888848981  0.3491153146307779  0.5010125554155187   T   T   T
  0.7896591964722176  0.3493892226659847  0.5008593569096085   T   T   T
  0.9248539655542388  0.3492947339383906  0.5013799906036537   T   T   T
  0.0791063314201286  0.6016754282730248  0.4960654617548412   T   T   T
  0.2176714238319891  0.6091593524301760  0.4923276021029085   T   T   T
  0.3577132974013595  0.6102504071270866  0.4920355931012931   T   T   T
  0.4968527662299804  0.6092943022923405  0.4918487383848945   T   T   T
  0.6354912001806816  0.6020650263940671  0.4956415491968530   T   T   T
  0.7893607164320138  0.6007423392443172  0.5032783833599472   T   T   T
  0.9245566035893747  0.6006407490222950  0.5036218702156386   T   T   T
  0.0695681068729403  0.8497779374003842  0.4968266758390083   T   T   T
  0.2122864907767086  0.8521571255279929  0.4957719298775959   T   T   T
  0.3571854150079127  0.8543126884948545  0.4955609409350771   T   T   T
  0.5014979060063409  0.8540509603291194  0.4960445447884819   T   T   T
  0.6450648039380434  0.8500502665180932  0.4958840467794564   T   T   T
  0.7864938781975761  0.8486145278156527  0.4970233970586855   T   T   T
  0.9279448891207113  0.8485853741441916  0.4975156399795221   T   T   T
  0.9980249756988143  0.0089552898045031  0.5044058980099311   T   T   T
  0.1390665580361884  0.0086368799324630  0.5050842684907880   T   T   T
  0.2822655497889107  0.0100303776474229  0.5058125291133304   T   T   T
  0.4260009251744359  0.0139797080102750  0.5082470112341022   T   T   T
  0.5801541773590614  0.0086616690240090  0.5039059629878325   T   T   T
  0.7178081283443946  0.0090703314451089  0.5035720536640137   T   T   T
  0.8575273253012962  0.0086693006008796  0.5037740664045982   T   T   T
  0.9951139609933014  0.2579074101314205  0.5060637076517859   T   T   T
  0.1379472535744532  0.2578310891274850  0.5126208649639330   T   T   T
  0.2707092755173656  0.2492161576900333  0.5092762265015608   T   T   T
  0.4435940120009676  0.2476771668537309  0.5077865279076212   T   T   T
  0.5775062201270744  0.2579190398495130  0.5115059909986098   T   T   T
```

0.7199951236659865	0.2582186581636416	0.5054258964074370	T	T	T
0.8575785062269562	0.2591743874398629	0.5046128944080909	T	T	T
0.9942608465081249	0.5132999574278806	0.5117749246674825	T	T	T
0.1554494958829846	0.5168714451338945	0.4981015450686434	T	T	T
0.2898568023961987	0.5203302568695981	0.4943071364244012	T	T	T
0.4247016591071350	0.5201116352479440	0.4939164077397740	T	T	T
0.5590440799216663	0.5168477463302955	0.4974585737782800	T	T	T
0.7194796579741762	0.5133947056346168	0.5109864425043641	T	T	T
0.8570976247048221	0.5112723340258080	0.5111232030107472	T	T	T
0.9995468802530496	0.7602895417936800	0.5043420518171534	T	T	T
0.1417559911476065	0.7635287443187125	0.5024231740785408	T	T	T
0.2857576379637169	0.7667157499721268	0.5013131921214040	T	T	T
0.4292397728091344	0.7674433695086177	0.5013149856491903	T	T	T
0.5722905593097718	0.7644676977569719	0.5019069457910490	T	T	T
0.7147638807294238	0.7604693842535734	0.5036083576130530	T	T	T
0.8572072728693774	0.7605717683309138	0.5056221369028915	T	T	T
0.1388160011706835	0.0733453220933586	0.4333421398326956	T	T	T
0.2825793965684436	0.0771073173200061	0.4346896058846266	T	T	T
0.4279762819995555	0.0804470323963292	0.4394590581826473	T	T	T
0.5788285949820952	0.0722606610919910	0.4310920002479021	T	T	T
0.7179473189741314	0.0748105651885964	0.4318216008232886	T	T	T
0.8580542469390512	0.0736304193127319	0.4317494396133542	T	T	T
0.9986244278007930	0.0748655767368432	0.4328049472062465	T	T	T
0.1352664158211013	0.3188793269779547	0.4390315734100281	T	T	T
0.2760896169389701	0.3133447751938858	0.4304208676569462	T	T	T
0.4377209872274896	0.3119018821972412	0.4294623463380296	T	T	T
0.5790575676578543	0.3185276606483233	0.4376711479356528	T	T	T
0.7212171732590896	0.3228710082410923	0.4334374151214559	T	T	T
0.8574922515388381	0.3263768173641194	0.4337265251228001	T	T	T
0.9935252892227020	0.3227106598181371	0.4342084777015698	T	T	T
0.1454843867160512	0.5824198161190289	0.4275839768325392	T	T	T
0.2871950349518207	0.5903787575195129	0.4255666929620521	T	T	T
0.4275005258147663	0.5901442289352762	0.4251183498277454	T	T	T
0.5691461629979858	0.5827698763012128	0.4271457872448169	T	T	T
0.7163307825519212	0.5757714754015089	0.4383064434254237	T	T	T
0.8571801985986318	0.5740038070131321	0.4368451805181280	T	T	T
0.9979987935816491	0.5752482930311973	0.4389169395632226	T	T	T
0.1407386966618144	0.8282259706859663	0.4304904864982506	T	T	T
0.2850473781475696	0.8324769894272189	0.4299911208182432	T	T	T
0.4295015890518876	0.8333512567030188	0.4300462171129908	T	T	T
0.5725878772480281	0.8301959362115161	0.4303384666885960	T	T	T
0.7158960480155058	0.8243431764676311	0.4309668139812136	T	T	T
0.8574532641707926	0.8233218435051928	0.4319568864620125	T	T	T
0.9986540312879396	0.8241010313054640	0.4317483159524275	T	T	T
0.0685930997943077	0.1969005068376001	0.4565863171340682	T	T	T
0.2052023896577847	0.1933290116427817	0.4550857054201488	T	T	T
0.3558243307091426	0.1989893568835068	0.4581931764313006	T	T	T
0.5110166847894486	0.1921785722766984	0.4541695941552382	T	T	T
0.6471068833019449	0.1969467594637646	0.4553402220417326	T	T	T
0.7882518865177364	0.1959296709210960	0.4519308580827647	T	T	T
0.9270923555452963	0.1958210451188929	0.4522337773194779	T	T	T
0.0590708996143965	0.4522568781107945	0.4536360536804915	T	T	T
0.2201990264094921	0.4665146540317536	0.4358732590040995	T	T	T
0.3570537525675382	0.4702949925281391	0.4329654978884836	T	T	T

0.4942160298352720	0.4664573605674995	0.4352843324106088	T	T	T
0.6548827193499742	0.4524765715205351	0.4527855278558176	T	T	T
0.7878771613566330	0.4497993806135164	0.4586834554683527	T	T	T
0.9265809342274963	0.4495181146175917	0.4593052594759422	T	T	T
0.0712003398043700	0.6998402956689784	0.4519160643114613	T	T	T
0.2141375026561048	0.7068071545797072	0.4485308489927512	T	T	T
0.3576075451954615	0.7084982428147427	0.4485535259574412	T	T	T
0.5001177340981608	0.7073768255664126	0.4480918077350063	T	T	T
0.6427505367885414	0.7003039627435612	0.4513308266314467	T	T	T
0.7870075447046155	0.6966098804649345	0.4549408081704474	T	T	T
0.9274257065268452	0.6964588023205373	0.4553019280780666	T	T	T
0.0690581204587995	0.9476566003610974	0.4521516851097365	T	T	T
0.2120438134574302	0.9500396903346717	0.4523535576826800	T	T	T
0.3571914533939811	0.9538480741472377	0.4533633663241853	T	T	T
0.5007125504190629	0.9539449975949913	0.4545053932297434	T	T	T
0.6469948246362400	0.9477646114007412	0.4501653004706443	T	T	T
0.7871931523768522	0.9465558475382956	0.4513296298821298	T	T	T
0.9281642094138653	0.9464463043813151	0.4521033921053610	T	T	T
0.9970484413400837	0.1596708639166934	0.5512166205589992	T	T	T
0.1386679214058764	0.1571677894747071	0.5533603902198453	T	T	T
0.2777897180158630	0.1534408208499721	0.5573434852675077	T	T	T
0.4343364459987031	0.1486700358723504	0.5510197570995317	T	T	T
0.5769996255179964	0.1568169878628253	0.5528121425908703	T	T	T
0.7180844584760883	0.1598870005462611	0.5508125051030262	T	T	T
0.8575977563914338	0.1606055229504178	0.5500007368824076	T	T	T
0.9914353835239251	0.4102077279117153	0.5596360853101282	T	T	T
0.7223324163354774	0.4102104300990975	0.5585823786573041	T	T	T
0.8568821538445681	0.4114041774662926	0.5554320876474110	T	T	T
0.0027034803130042	0.6612733773828997	0.5490092047661035	T	T	T
0.1459451716737668	0.6658152928055030	0.5473136324111398	T	T	T
0.2869695595020114	0.6692699524641366	0.5455916721228899	T	T	T
0.4282529566782885	0.6697615861691252	0.5454490194619263	T	T	T
0.5683286070724655	0.6665093106164051	0.5466623642408270	T	T	T
0.7113317392758476	0.6617549087757052	0.5484812979256282	T	T	T
0.8569251007596387	0.6640768370092552	0.5553854772667003	T	T	T
0.9986416392411884	0.9104942437039774	0.5492060886114423	T	T	T
0.1406549080630851	0.9105491812197422	0.5490634141394831	T	T	T
0.2840931142019921	0.9118114568371767	0.5485765239268323	T	T	T
0.4288080570045541	0.9137449183320113	0.5484288119436529	T	T	T
0.5749177792292304	0.9117180902172651	0.5486617769905321	T	T	T
0.7158327823722412	0.9108043162126960	0.5483290929830783	T	T	T
0.8570558274651899	0.9104955851499154	0.5488814375077694	T	T	T
0.0681621044166746	0.0316385456292880	0.5708551215066776	T	T	T
0.2100801189686256	0.0300700027727207	0.5714984788560248	T	T	T
0.3532667917403179	0.0316586257513413	0.5733356366189956	T	T	T
0.5055333392118200	0.0304085687539098	0.5698805992128968	T	T	T
0.6482628264276437	0.0327642205608715	0.5705493925077674	T	T	T
0.7875569814747653	0.0327397061322801	0.5697706353646088	T	T	T
0.9274022189062954	0.0325472857229803	0.5700164237237393	T	T	T
0.0626903885409472	0.2807949873634177	0.5755146156868921	T	T	T
0.2116099184781882	0.2779554045998646	0.5821960846227721	T	T	T
0.3584093643543595	0.2566959021390783	0.5719658349182309	T	T	T
0.5032191982772640	0.2763231167994311	0.5805937043419773	T	T	T
0.6521567890097775	0.2807264226907561	0.5745771693357237	T	T	T

0.7890387153957070	0.2819436770197927	0.5717756563521033	T	T	T
0.9257011559663404	0.2818130623991786	0.5721297761810421	T	T	T
0.0756752923978981	0.5447037394352817	0.5704105860123723	T	T	T
0.2209312592579180	0.5447914340732423	0.5643605759276761	T	T	T
0.3574538646881608	0.5433304494815766	0.5640970333654043	T	T	T
0.4936346303920172	0.5446692889362472	0.5637605784335923	T	T	T
0.6383221410223626	0.5445934377105991	0.5697987341166016	T	T	T
0.7869391487101807	0.5388349877760678	0.5774115657313904	T	T	T
0.9264782034135827	0.5390361389965399	0.5779000346799809	T	T	T
0.0711670667103825	0.7851817594083971	0.5689914261137595	T	T	T
0.2139372015892704	0.7872093829771292	0.5673602951732554	T	T	T
0.3573755375812939	0.7894328411114030	0.5670515964426815	T	T	T
0.5008955257615203	0.7885026920512958	0.5672337815098977	T	T	T
0.6433187815977528	0.7855595444522812	0.5682384800668078	T	T	T
0.7849268943603097	0.7845420118018462	0.5697819148676677	T	T	T
0.9290310280373142	0.7845145986192981	0.5702536150599143	T	T	T
0.1589404318682183	0.4325944102564087	0.6166575116800099	T	T	T
0.2826072177963518	0.4232034690577857	0.6160479531313102	T	T	T
0.4325995650813481	0.4231531571914650	0.6156882523874737	T	T	T
0.5553823319717385	0.4317278896602826	0.6162502699683202	T	T	T
0.0901784723897968	0.4481051602319210	0.5447688865476192	T	T	T
0.2239592933026668	0.4344875294231380	0.5426095795935403	T	T	T
0.3576436874136041	0.4412047204562540	0.5468369255692925	T	T	T
0.4908894353289059	0.4339333643176315	0.5418390581097701	T	T	T
0.6236704631650944	0.4479898756695473	0.5439365568379224	T	T	T
0.1409174084985986	0.3539661169367164	0.5642699836238665	T	T	T
0.2999764602559920	0.3454332841785948	0.5617496258991528	T	T	T
0.4159777887969765	0.3453489893752197	0.5610376573260606	T	T	T
0.5735493205885276	0.3534622688996325	0.5634836475405275	T	T	T
0.1510427977918229	0.4107706122328045	0.4779773954128674	T	T	T
0.2968273038257952	0.4025273327781805	0.4772398559907284	T	T	T
0.4175984227025561	0.4019935781655953	0.4765159407440284	T	T	T
0.5632279770218477	0.4101594435253044	0.4771275325888114	T	T	T
